# London Dispersion Interactions Rather than Steric Hindrance Determine the Enantioselectivity of the Corey–Bakshi–Shibata Reduction

**DOI:** 10.1002/anie.202012760

**Published:** 2021-01-28

**Authors:** Christian Eschmann, Lijuan Song, Peter R. Schreiner

**Affiliations:** ^1^ Institute of Organic Chemistry Justus Liebig University 35392 Giessen Germany; ^2^ Current address: Shenzhen Bay Laboratory Shenzhen 518055 China

**Keywords:** asymmetric catalysis, borane reduction, CBS reduction, noncovalent interactions, steric repulsion

## Abstract

The well‐known Corey–Bakshi–Shibata (CBS) reduction is a powerful method for the asymmetric synthesis of alcohols from prochiral ketones, often featuring high yields and excellent selectivities. While steric repulsion has been regarded as the key director of the observed high enantioselectivity for many years, we show that London dispersion (LD) interactions are at least as important for enantiodiscrimination. We exemplify this through a combination of detailed computational and experimental studies for a series of modified CBS catalysts equipped with dispersion energy donors (DEDs) in the catalysts and the substrates. Our results demonstrate that attractive LD interactions between the catalyst and the substrate, rather than steric repulsion, determine the selectivity. As a key outcome of our study, we were able to improve the catalyst design for some challenging CBS reductions.

## Graphical abstract

London dispersion (LD) interactions facilitate the enantioselectivity in the Corey–Bakshi–Shibata (CBS) reduction. Employing a combination of computational and experimental studies, we provide a modern view on the origin of enantioselectivity in this powerful organocatalyzed reaction. The results demonstrate that attractive LD interactions between the catalyst and the substrate rather than steric repulsion determine the selectivity.

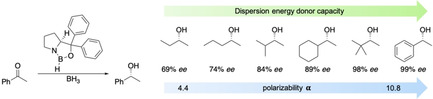

## Introduction

The detailed understanding of reaction mechanisms and the origin of enantioselectivity is essential for successful catalyst design. Enantioselectivity imparted by chiral, small‐molecule catalysts is rationalized typically by preferential steric destabilization derived from the repulsive part of the van der Waals (vdW) potential because it can be readily understood and taught with hard‐sphere classical mechanics models. In contrast, London Dispersion (LD), the attractive part of the vdW potential,^[1]^ is often neglected in mechanistic considerations and for catalyst design.^[2]^ However, for a detailed understanding of a given catalytic system, all interactions must be considered, even though we are just learning how to conceptualize this for reaction planning.^[3]^ Fortunately, modern computational techniques like dispersion corrected density functional theory (DFT) now allow a detailed analysis of all factors contributing to transition state stabilization for a much better understanding of catalyst design.^[4]^ Here we chose the Corey–Bakshi–Shibata (CBS) reduction, which is a versatile method for the enantioselective reduction of prochiral ketones by oxazaborolidines (OXB), achieving high selectivities and yields^[5]^ to demonstrated that *all* steric factors, attraction and repulsion, have to be taken into account to arrive at a balanced description of the mechanism and to design new, more selective catalysts.

**Scheme 1:**
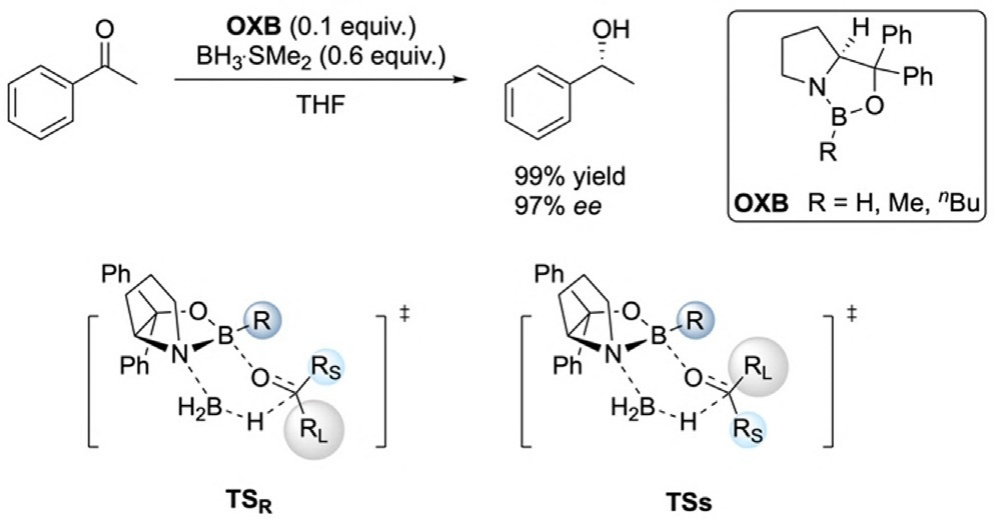
CBS reduction of acetophenone and proposed transition structures for hydride transfer, favoring the (*R*)‐product on the basis of minimizing the steric repulsion between “**R**” and “**R_L_**”.

Corey's widely accepted mechanistic model bases stereoselection exclusively on steric repulsion between the boron substituent **R** on the catalyst and the large **R_L_** and small **R_S_** substituents of the ketone in a six‐membered boat‐like transition state (Scheme 1).[^5f^, ^6^] With this model, one can qualitatively predict the enantiofacial discrimination of numerous substrates. However, this model of steric destabilization does not offer a satisfying explanation for the selectivity and reactivity of some substrates. For example, the reduction of trichloroacetophenone predominantly generates the (*R*)‐enantiomer.^[6a]^ This implies that the large phenyl group (R_L_) faces the boron substituent in the favored transition state, which is in contrast to Corey's standard model depicted in Scheme 1. In the reduction of cyclopropyl isopropyl ketone (1‐cyclopropyl‐2‐methylpropan‐1‐one) one would assume poor selectivity, because both substituents are similar in steric size. Nonetheless, the reduction delivers the (*R*)‐enantiomer with a selectivity of 91 % *ee*.^[5f]^ Similarly, a high *ee* (81 %) was also found for *p*‐methoxy‐*p*′‐nitrobenzophenone with two groups of similar size.^[5f]^ In these two cases, the cyclopropyl substituent and the *p*‐methoxyphenyl group act as R_L_, respectively, thereby demonstrating that other factors must also play an important role in the transition state structure. Furthermore, despite bearing bulky groups, there are substrates that do not deliver high selectivity, e.g., unbranched aliphatic ketones.^[7]^

There are several reports on the stereoselection of the CBS reduction trying to shed light on the origin of its enantioselectivity. In 1993 Liotta et al. used the MNDO semiempirical approach to suggest that the reduction is more likely to occur via a chair‐like transition state. In addition, the carbinol phenyl substituents of the catalyst are required to lie parallel to the **R_L_** substituent to minimize steric repulsion.^[8]^ Meyer et al. investigated the role of steric repulsion in the transition structures of the reduction by determining kinetic isotope effects (KIE), as the C‐D bond is effectively shorter than the C−H bond, resulting in inverse ^2^H KIEs for reactions in which steric repulsion increases in the transition structure.^[9]^ They concluded that Corey's steric reasoning is too simplistic, because in the reduction of acetophenone the chair‐like transition state prevails, with the boron substituent only playing a minor role.^[10]^ In a recent theoretical study, Lachtar et al. suggested that the origin of the enantioselectivity for the oxazaborolidine catalyzed reduction of ketimines can be traced back to noncovalent interactions in the preferred transition structure.^[11]^ However, by replacing the phenyl groups of the catalyst by hydrogens, they used a computationally reduced model that neglects major parts of these key noncovalent interactions. Furthermore, their B3LYP/6‐31G(d,p) computations do not include dispersion corrections.

Herein, we aim at bringing together experimental and computational studies geared towards understanding a reaction whose stereochemical outcome was classically interpreted as being derived solely on the basis of steric repulsion. We demonstrate that a more detailed and hence more powerful mechanistic reasoning emerges when all interactions are taken into account and we gauge the role of attractive LD stabilization in this particular reaction.

## Results and Discussion

This section is organized in three parts. First, a comprehensive computational investigation of the various noncovalent interactions (NCI) in the transition structures provides a contemporary view of the origin of the enantioselectivity in the CBS reduction. Then we show how these insights help in the design of new catalysts to improve enantioselectivity, especially for some challenging substrates. Finally, we provide an experimental validation of our improved understanding of catalyst design.

### Reconsidering Steric Effects

**Figure 1:**
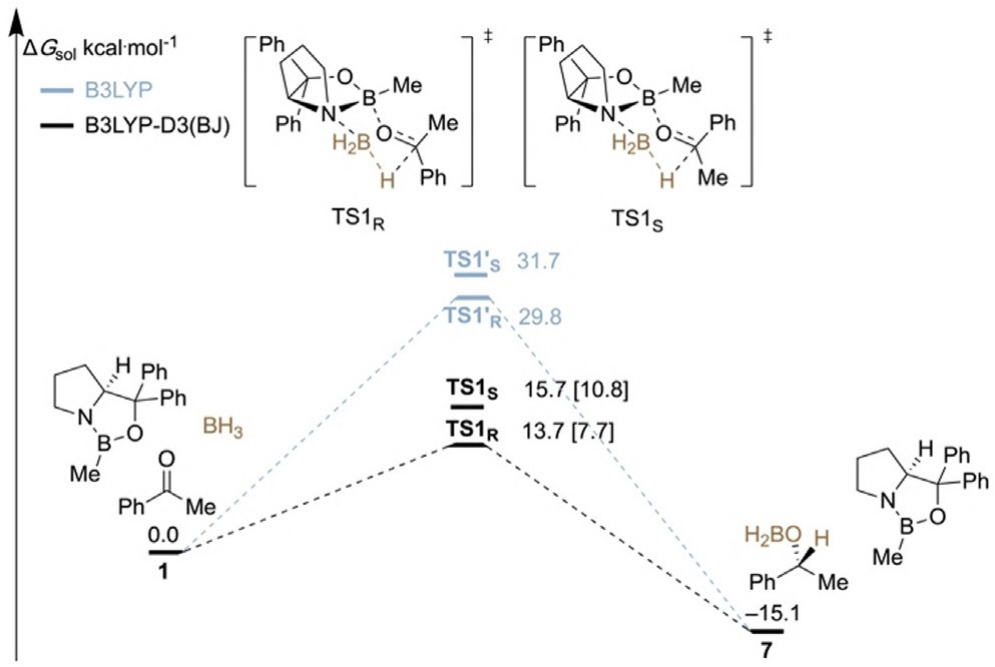
Potential energy surface displaying the free energies (ΔG275sol ) of the CBS pathway with (black) and without dispersion (gray) corrections at 2 °C (for a more detailed PES, see the Supporting Information, Figure S2). Level of theory: B3LYP‐D3(BJ)/6–311+G(d,p)‐SMD(THF)//B3LYP‐D3(BJ)/6–311G(d,p). The free energies in brackets are based on electronic DLPNO‐CCSD(T)/cc‐pVTZ single‐point energy (corrected for ZPVE).

None of the previously reported computational mechanistic studies include LD corrections,[^8^, ^9^, ^10^, ^11^, ^12^] which are needed to strike a proper balance between repulsive and attractive noncovalent interactions. As this is the very concept of an “equilibrium structure”, we set out to determine the role LD plays in the CBS reduction. We first computed the reaction pathway for the reduction of acetophenone using a comparison of B3LYP vs. B3LYP‐D3(BJ) with appropriate basis sets and solvent inclusion (see Computational Details below); the difference should provide a good estimate of the role dispersion plays (Figure 1). A detailed potential energy surface (PES) of the complete reaction pathway and higher‐level single‐point energy computations with DLPNO‐CCSD(T)^[13]^ (domain‐based local pair natural orbital CCSD(T)) for the key step are provided in the Supporting Information (Supporting Information, Figure S1).

In this simplified PES, we start with the catalyst, the reducing agent, and acetophenone as reference **1**. The hydride transfer determining enantioselectivity occurs via two diastereomeric transition structures. If LD is not taken into account (color‐coded in gray), the transition structures **TS1′_S_** and **TS1′_R_** are found to be very high in free energy exhibiting barriers of 29.8 kcal mol^−1^ and 31.7 kcal mol^−1^, respectively. These energy barriers are too high for such a fast reaction at 25 °C. After inclusion of LD (color‐coded in black), the relative energies of the transition structures **TS1_R_** and **TS1_S_** are notably lower with barriers of only 13.7 kcal mol^−1^ and 15.7 kcal mol^−1^, respectively, which is much more reasonable for a catalyzed reaction that proceeds quickly at room temperature. The calculated enantioselectivity of the reduction, which is expressed in the energy difference between the transition structures (ΔΔ*G* ^≠^) for hydride transfer, is −2.0 kcal mol^−1^ and thereby consistent with previously published experimental results (−2.2 kcal mol^−1^).^[5b]^ We computed the transition structures in solvent within the limitations of an SCRF model. The energy difference of **TS1_R_** and **TS1_S_** in gas phase is 4.0 kcal mol^−1^, while it is 2.0 kcal mol^−1^ in THF. As expected, the LD interactions are attenuated by the interaction with the solvent but, more importantly, they do not vanish. Catalyst regeneration and release of the boronate **7** is exergonic by −15.1 kcal mol^−1^. For comparison, we also added DLPNO‐CCSD(T)/cc‐pVTZ single‐point energies on the DFT‐optimized geometries (and ZPVE corrections) of **TS1_S_** and **TS1_R_**, which are 7.7 kcal mol^−1^ and 10.8 kcal mol^−1^, respectively. This indicates that the dispersion‐corrected energy barrier is more reasonable and that more complete inclusion of electron correlation effects emphasize the importance of LD.

**Figure 2:**
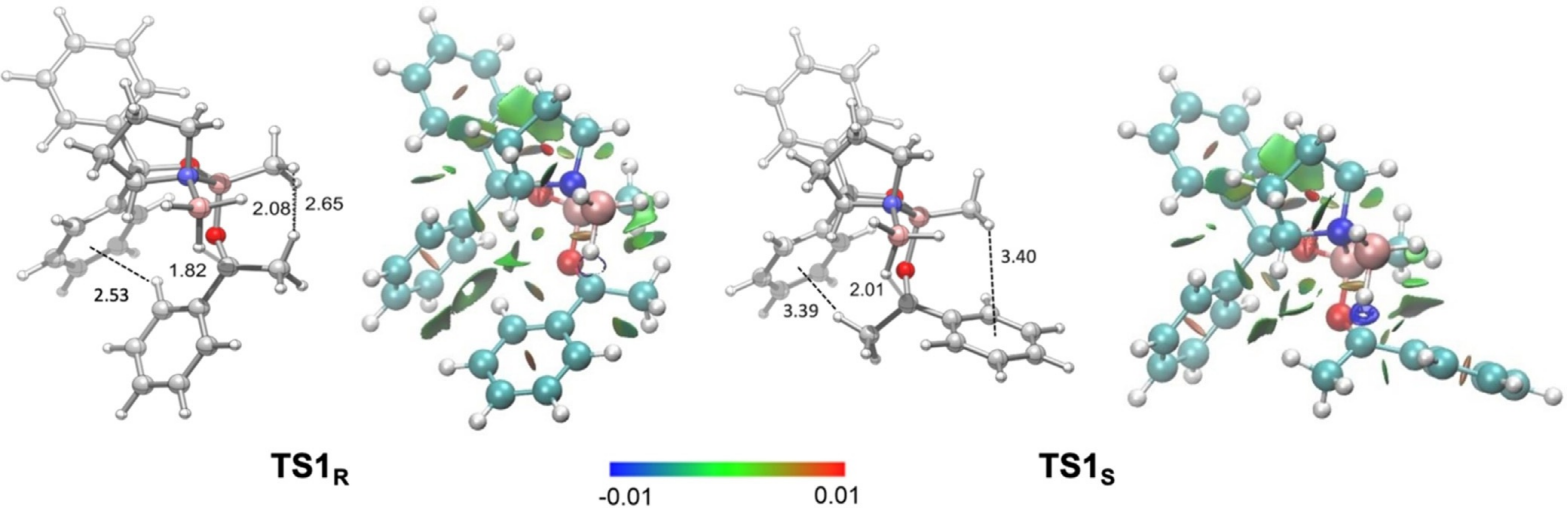
Selectivity determining transition structures for hydride transfer in the CBS‐reduction of acetophenone with (*S*)‐1‐methyl‐3,3‐diphenylhexahydropyrrolo[1,2‐*c*][1,3,2]oxazaborole as the catalyst. Selected bond distances in Å and noncovalent interaction (NCI) plots (s=0.5 a.u., −0.01 < r < +0.01 a.u.). Color code: repulsion (red), strong attraction (blue), weak noncovalent interactions (green). The transition structures were optimized at B3LYP‐D3(BJ)/6–311G(d,p).

A closer look at the geometries of the transition structures **TS1_R_** and **TS1_S_** reveals chair‐like conformations (Figure 2), which are 3.8 kcal mol^−1^ lower in energy, than the corresponding boat‐like conformations suggested by Corey (Supporting Information, Figure S1). Thereby, the catalyst binds to the ketone at the lone pair facing the small substituent (**R_S_**) *anti* to the electron‐rich substituent as it is also described in Corey's model. NCI plots indicate some differences of the noncovalent interactions between catalyst and substrate in the two transition structure conformations.^[14]^ Contrary to Corey's model no steric destabilization (repulsion is color‐coded red in the NCI plot) by hydrogen‐hydrogen contacts can be found in less favored **TS1_S_**. In fact, the bond distances of around 2.5 Å in the preferred transition structure **TS1_R_** lead to stabilizing σ‐π LD interactions^[15]^ between acetophenone and the phenyl groups of the catalyst, as visualized by the green areas in the NCI plot. Additionally, the methyl substituent of the substrate interacts favorably with the boron substituent of the catalyst. In the less favored transition structure **TS1_S_** the long distance between substituents of substrate and catalyst prevent optimal interactions. These computations suggest LD interactions to be important for enantiodiscrimination. We additionally employed LD potential maps developed by Pollice and Chen to visualize these LD interactions (Supporting Information, Figure S2).^[16]^ These confirm the conclusions drawn from the qualitative NCI analysis.

To examine the general effect of LD interactions on the enantioselectivity, three literature examples with various substrates and catalysts were also studied (Table 1). In the reduction of cyclohexyl methyl ketone (1‐cyclohexylethanone, entry 2) the moderate enantioselectivity (85 % *ee*) is likely due to decreased LD interactions of the cyclohexyl with the phenyl group of the catalyst.^[5a]^ Similarly, replacing the phenyl substituents with a spirocyclopentyl group in the catalyst leads to diminished enantioselectivity of 67 % *ee*.^[17]^ This implies that the LD interactions of the phenyl groups in the catalyst are crucial for enantioselectivity. Note that the selectivities obtained from our LD corrected computations (B3LYP‐D3(BJ)) are in better agreement with the experimental *ee* values (Table 1) than the uncorrected values.[^5a^, ^17^]

**Table 1:** Activation free energy (at the temperature of the experiment) differences in kcal mol^−1^: experiment vs. theory. Level of theory: B3LYP‐D3(BJ)/6–311+G(d,p)‐SMD//B3LYP‐D3(BJ)/6–311G(d,p). 
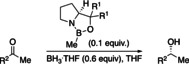

R^1^	R^2^	Config *ee* [%]	ΔΔG≠exp	ΔΔG≠sol (without D3)	ΔΔG≠sol (with D3)
Ph	Ph	*R* 97	2.2 (2 °C)	1.9	2.0
Ph	Cy	*R* 85	1.3 (−10 °C)	0.9	1.1
‐(CH_2_)_4_‐	Ph	*R* 67	1.0 (23 °C)	2.4	1.2

**Figure 3:**
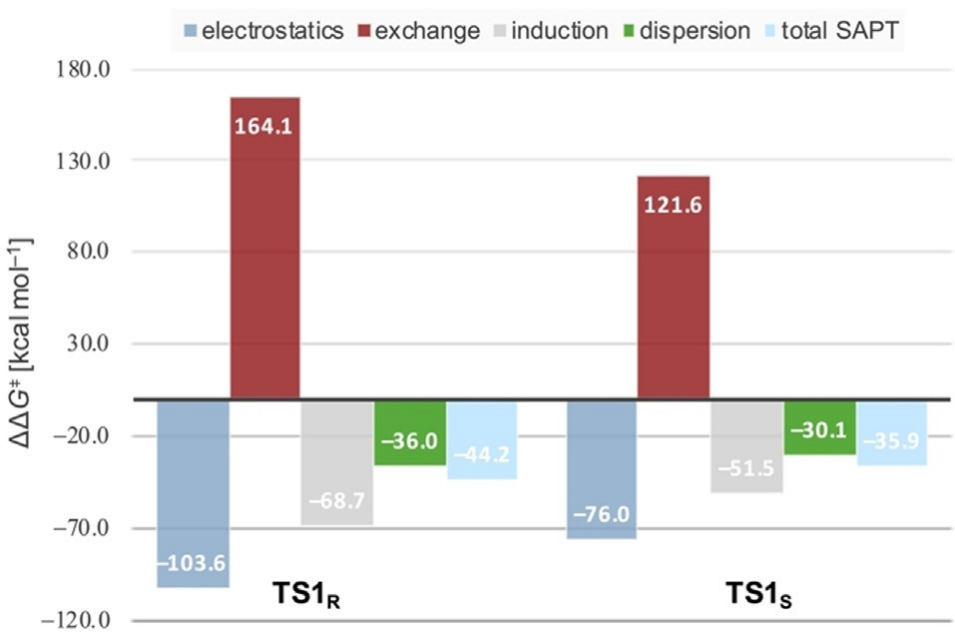
SAPT0 analysis of the transition structures **TS1_R_** and **TS1_S_** in the CBS reduction of acetophenone. Level of theory: SAPT0/jun‐cc‐pvdz. For a plot of relative energies and more information, see the Supporting Information.

SAPT0 (Symmetry Adapted Perturbation Theory) was employed to analyze the different energetic contributions of the interactions between substrate and catalyst in the transition structures (Figure 3).^[18]^ The components include electrostatics, exchange, induction, and LD energies. The electrostatic term arises from the large Coulomb interactions between the Lewis acid and Lewis base sites (carbonyl and boryl as well as amino and boryl groups). In the transition structures, electrostatics and induction dominate the interactions but they are counterbalanced by a large exchange term (i.e., Pauli repulsion), indicating significant steric repulsion. However, the larger exchange energy in favored **TS1_R_** disagrees with Corey's model, in which the larger exchange term should favor **TS1_S_**. Therefore, the selectivity is not determined by steric repulsion (alone). Although LD is a small part of the total interaction energy, it decisively contributes. The LD energy preference for **TS1_R_** is 5.9 kcal mol^−1^, which is in good agreement with the experimentally observed high selectivity.[^5a^, ^5b^] Thus, our computational results strongly suggest that LD interactions between catalyst and substrate also determine the enantioselectivity.

### Improving Catalyst Design with Dispersion Energy Donors

Based on our new understanding of the origin of enantioselectivity in the CBS reduction, we hypothesized that higher enantioselectivity can be achieved by modifying the catalyst with dispersion energy donors (DEDs)[^2c^, ^15b^] that enable favorable substrate‐catalyst interactions through increasing polarizability. The catalyst modifications involve on the one hand the boron substituent and the carbinol substituents on the other.

First, we investigated the effect of the substituent at boron (Table 2); DEDs including ^*i*^Pr, ^*t*^Bu, Cy, CH_2_Cy, *c*‐C_5_H_9_ were employed. In the reduction of cyclohexyl ketone LD interactions are the highest using CH_2_Cy with a ΔΔ*G* ^≠^ of 3.5 kcal mol^−1^ (entry 1). As expected for large and highly polarizable groups, Cy and ^*t*^Bu should also deliver significant enantiodiscrimination (entries 2 and 4). For *tert*‐butyl methyl ketone, only the ^*t*^Bu and Me substituents show high selectivity (entries 8 and 9). However, our experimental results demonstrate that the selectivity does not change much as compared to the original catalyst when using CH_2_Cy and Cy groups (Supporting Information, Figure S3). This implies that the interactions between substrate and the substituent at boron on the catalyst only have a subtle effect on the enantioselectivity.

**Table 2:** The energy difference between the transition structures for hydride transfer for computed substrates and catalysts at 25 °C. Level of theory: B3LYP‐D3(BJ)/6–311+G(d,p)‐SMD// B3LYP‐D3(BJ)/6–311G(d,p). 
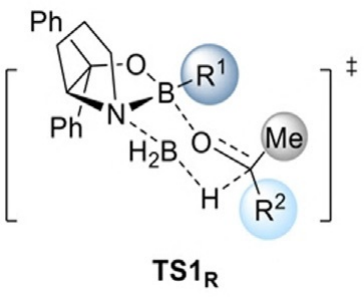

Entry	R^1^	R^2^	ΔΔG≠sol (without D3)	ΔΔG≠sol (with D3)
1	CH_2_Cy	Cy	1.0	3.5
2	Cy	Cy	−0.4	2.3
3	*c*‐C_5_H_9_	Cy	−2.2	−0.2
4	^*t*^Bu	Cy	0.1	2.0
5	^*i*^Pr	Cy	1.2	0.9
6	Cy	^*t*^Bu	−0.5	−1.1
7	*c*‐C_5_H_9_	^*t*^Bu	−0.3	0.1
8	^*t*^Bu	^*t*^Bu	1.7	2.3
9	Me	^*t*^Bu	4.1	4.2

The variation of carbinol substituents of the catalyst leads to much larger changes of enantioselectivity. Replacing the phenyl groups with aliphatic DEDs, e.g., Me, ^*i*^Pr, and ^*n*^Bu show comparable or even reduced selectivity relative to the original catalyst with its unsubstituted phenyl groups.^[6b]^

**Figure 4:**
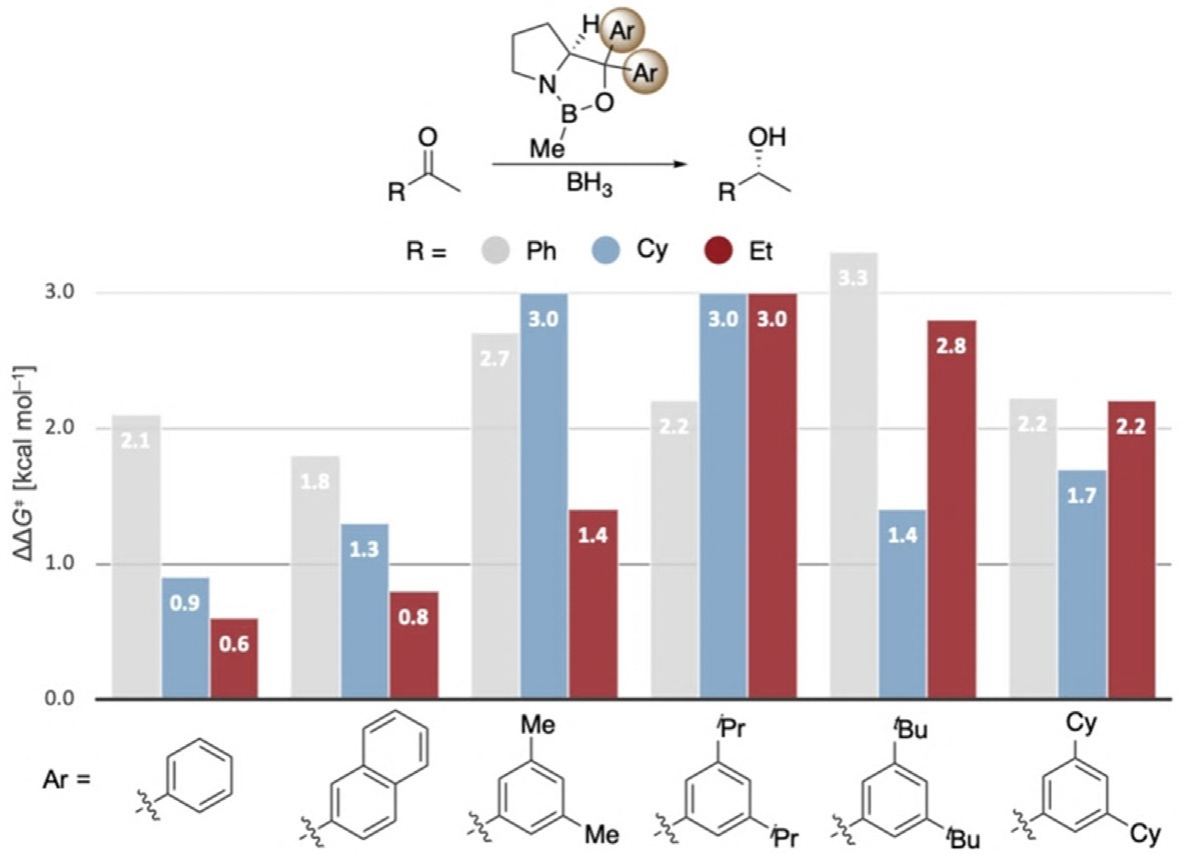
Computed enantioselectivities (expressed through ΔΔ*G* ^≠^) in the reduction of acetophenone, cyclohexyl methyl ketone, and 2‐butanone using different Ar groups on the catalyst at 25 °C. Level of theory: B3LYP‐D3(BJ)/6–311+G(d,p)‐SMD// B3LYP‐D3(BJ)/6–311G(d,p).

The introduction of DEDs in the *meta*‐positions of the aryl groups of the catalyst results in comparably high or even slightly higher enantioselectivities relative to the original catalyst in the reduction of acetophenone (Figure 4).

**Figure 5:**
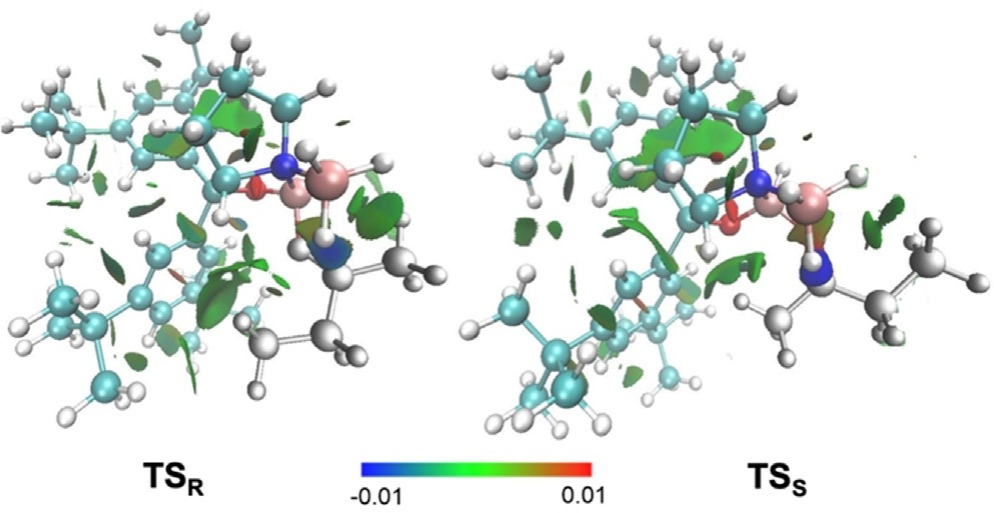
NCI plots (s=0.5 a.u., −0.01 < r < +0.01 a.u.) of the transition structures in the reduction of 2‐butanone using 3,5‐^*t*^Bu_2_Ph as carbinol substituent on the catalyst. Color code: repulsion (red), strong attraction (blue), weak noncovalent interactions (green). The transition structures were optimized at B3LYP‐D3(BJ)/6–311G(d,p).

The intermolecular stabilization by all‐*meta* substitution in dispersion‐driven systems has been demonstrated recently, e.g., in the stabilization of molecular dimers^[19]^ and in the catalytic hydroamination of olefins.^[3d]^ Here we also find that DEDs in *meta*‐aryl positions provide additional attractive interactions with, e.g., the ethyl substituent of 2‐butanone, as indicated in the NCI plots (Figure 5). Again, more stabilizing interactions (−2.8 kcal mol^−1^) between aryl groups on the catalyst and the ethyl group in the substrate are found in the preferred **TS_R_**.

**Figure 6:**
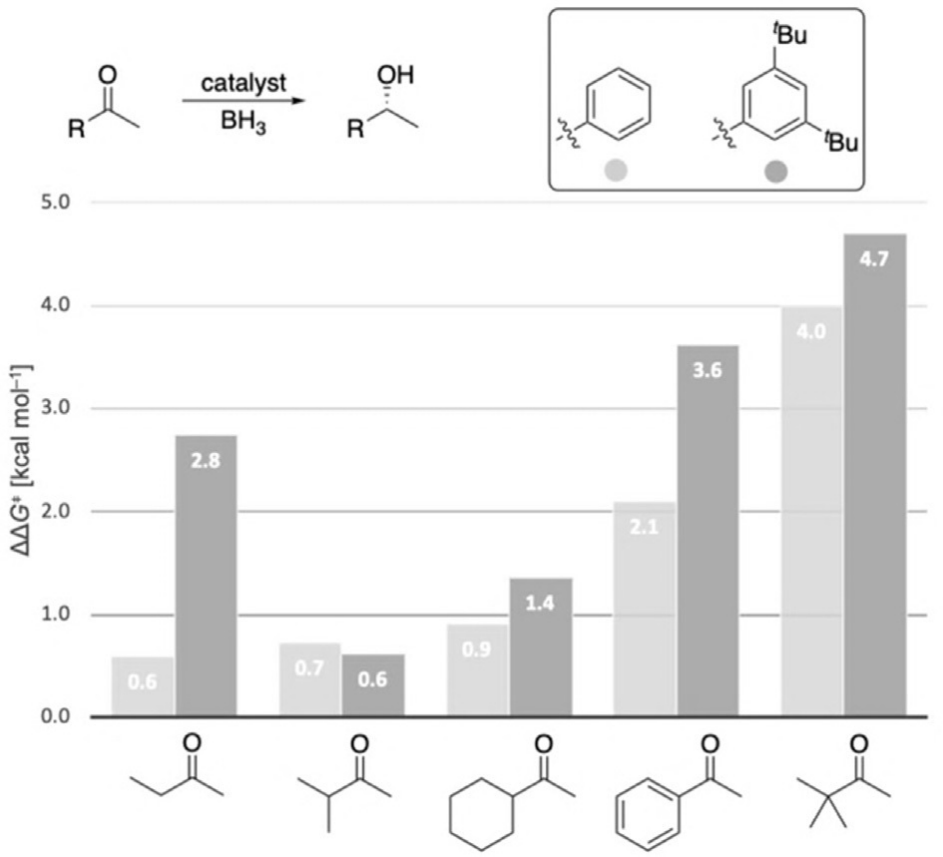
Computed enantioselectivities (expressed as ΔΔ*G* ^≠^ values) for the reduction of various ketones employing the 3,5‐ ^*t*^Bu_2_Ph catalyst compared to the original CBS catalyst at 25 °C. Level of theory: B3LYP‐D3(BJ)/6–311G+(d,p)‐SMD// B3LYP‐D3(BJ)/6–311G(d,p).

To explore the general potential of aryl‐substituted catalysts computationally, the 3,5‐^*t*^Bu_2_Ph catalyst was computed in the reduction of various substrates (Figure 6). The modified catalyst shows comparable or improved enantioselectivity, especially for substrates yielding low enantioselectivity with the original catalyst, e.g., 2‐butanone and cyclohexyl methyl ketone. For 2‐butanone, ΔΔ*G* ^≠^ improves from 0.6 to 2.8 kcal mol^−1^, implying a theoretical change in *ee* from 47 % to 98 %.

### Experimental Validation

**Figure 7:**
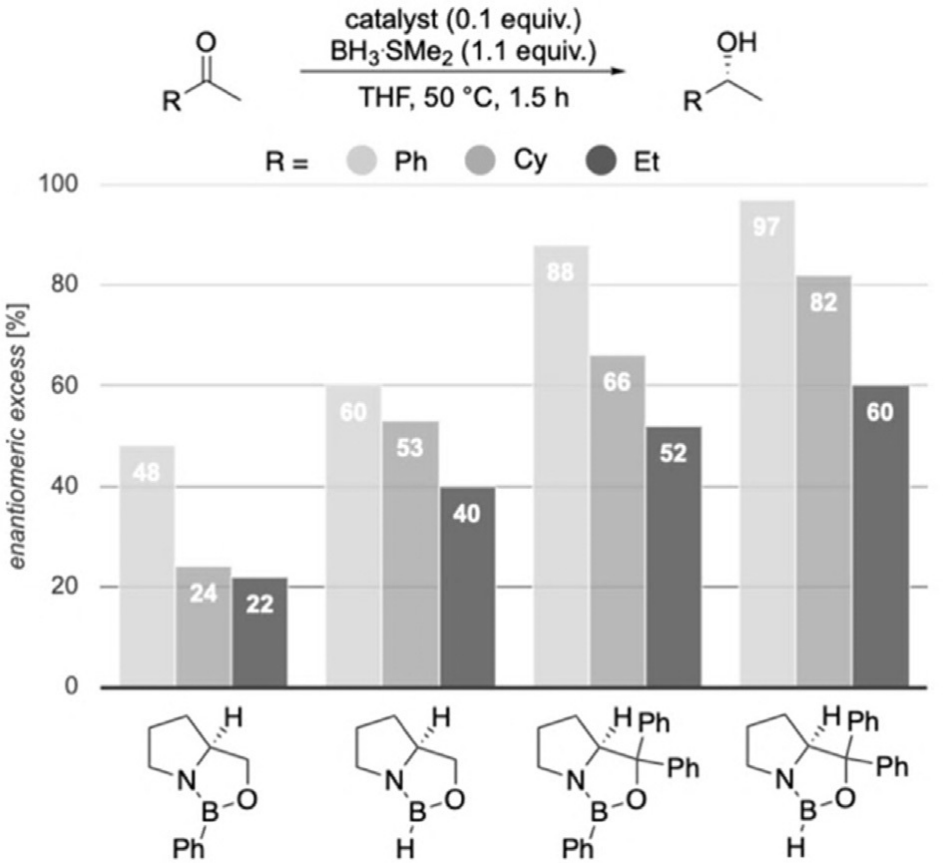
Reduction of prochiral ketones employing modified CBS catalysts.

To examine our computational predictions, we performed an experimental validation employing various catalysts and substrates. We started with comparing the effects of changing the substituents in the catalyst at the carbinol and boron positions. We compared the original CBS catalyst to three modified versions in the reduction of three ketones bearing aromatic, branched or unbranched alkyl substituents. We employed Corey's standard protocol using 10 mol % of catalyst, 1.1 equivalents of reducing agent in THF at 50 °C for 1.5 h (Figure 7).^[6b]^ We chose slightly elevated temperatures, as for borane reductions there is an increase in selectivity with increasing temperature up to 30–50 °C (Supporting Information, Table S2).^[20]^ This resulted in nearly quantitative yields. As expected, by changing the carbinol substituent of the catalyst from hydrogen to phenyl, the selectivity increases.

These initial findings support our proposal that the carbinol substituents are key for enantiofacial discrimination due to LD interactions with the substrate. Furthermore, when replacing the hydrogen at boron with a phenyl group, we observe a decrease in enantioselectivity. This disagrees with the steric repulsion hypothesis, where (*R*)‐selectivity should improve with increasing steric size of the substituent at boron.^[6b]^ Consistent with our computations (Figures 2 and 3), we relate this to stabilizing LD interactions between substrate and the phenyl group at boron in less favored **TS_S_** (Figure 2). This does not exclude the notion that catalysts bearing a phenyl group at boron are probably weaker Lewis acids and less effective, and the lower selectivity might also be a result of a more prominent unselective background reaction.

**Figure 8:**
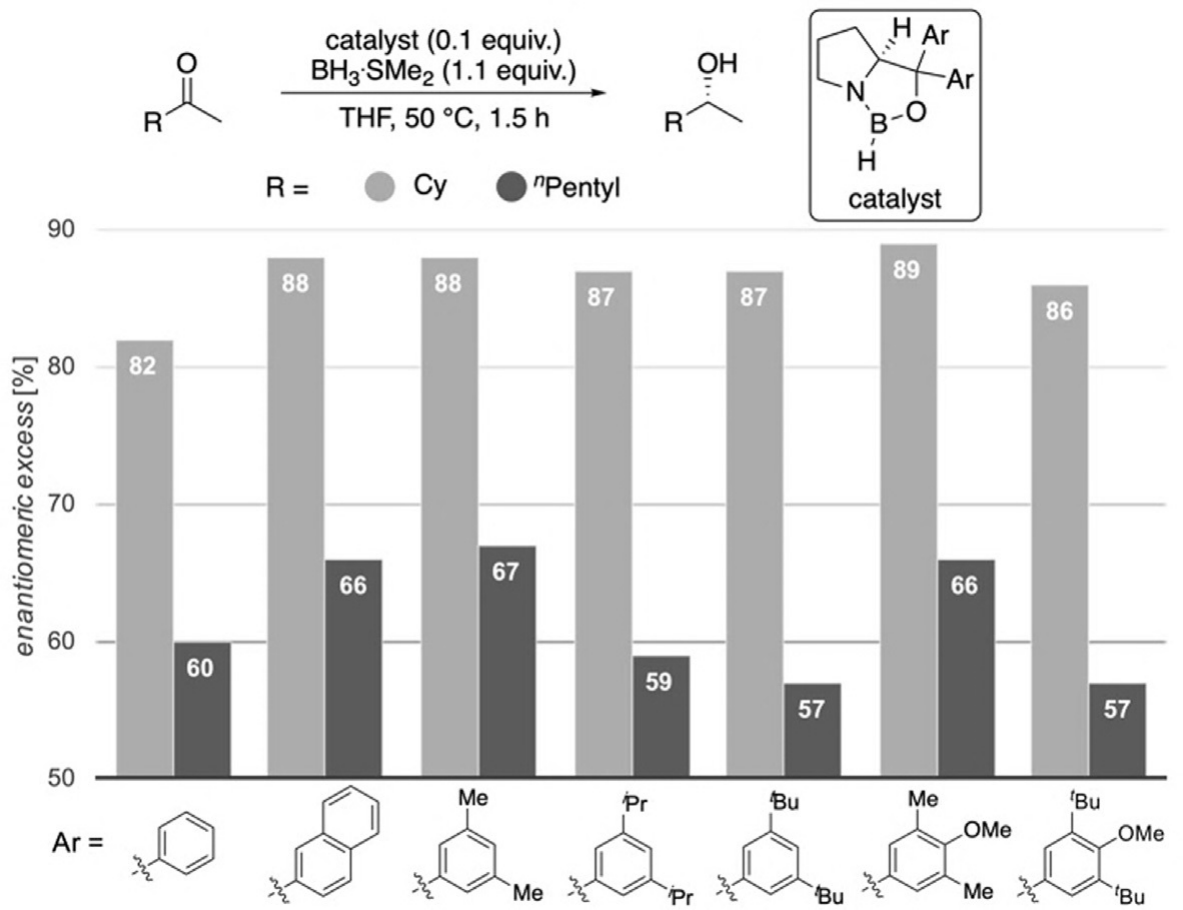
CBS reductions employing modified catalysts with DEDs in the catalyst's carbinol position.

**Figure 9:**
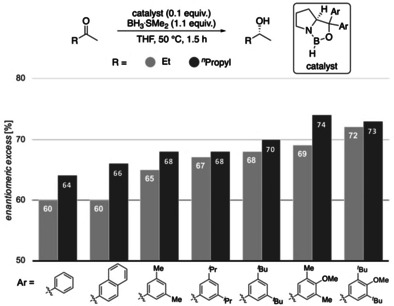
CBS reductions employing modified catalysts with DEDs in the catalyst's carbinol position for challenging substrates. Corey's original catalyst is the first entry (Ar=phenyl).

Next, we experimentally probed the computationally predicted effects of the carbinol substituents shown in Figure 4. We employed catalysts with aryl groups bearing additional DEDs to check whether the LD interactions increase and thereby increase enantioselectivity (Figures 8 and 9). In all cases, at 50 °C after 1.5 h the reduction results in near quantitative yields. In the reduction of cyclohexyl methyl ketone all modified catalysts achieve higher selectivities due to additional LD stabilizations. As LD through the *meta*‐substituent seems to be maximized at methyl already, we decided to test the 4‐OMe‐3,5‐Me_2_Ph and 4‐OMe‐3,5‐^*t*^Bu_2_Ph catalysts that should be even more polarizable due to electron donation from the methoxy group. Indeed, the best selectivities were achieved with the 3,5‐Me_2_Ph‐ and 4‐OMe‐3,5‐Me_2_Ph catalysts. The selectivities with 3,5‐^*i*^Pr_2_Ph, 3,5‐^*t*^Bu_2_Ph and 4‐OMe‐3,5‐^*t*^Bu_2_Ph are slightly lower, as the substituents are getting too bulky for cyclohexyl ketone; the results are similar for the reduction of 2‐heptanone. While the overall selectivity is lower due to entropic penalty of the linear alkyl chain,^[21]^ we observed the best selectivities with 3,5‐Me_2_Ph and 4‐OMe‐3,5‐Me_2_Ph. These experimental results fit the qualitative expectation from our improved model and confirm our computations of Figure 4, as the 3,5‐Me_2_Ph catalyst was the also the best computed catalyst for cyclohexyl ketone.

Importantly, in the challenging reductions of smaller *n*‐alkyl ketones, we achieve a steady increase in selectivity from 60 % up to 72 % *ee* for 2‐butanone and 64 % up to 74 % *ee* for 2‐pentanone by increasing DEDs and adding further electron‐donor groups (4‐OMe‐3,5‐Me_2_Ph, 4‐OMe‐3,5‐^*t*^Bu_2_Ph) to the catalyst (Figure 9). These results also fit qualitatively to the computations of Figures 4 and 5, as computations suggest the 3,5‐^*i*^Pr_2_Ph and 3,5‐^*t*^Bu_2_Ph derivatives to be the best performing catalysts (the 4‐OMe‐3,5‐Me_2_Ph, 4‐OMe‐3,5‐^*t*^Bu_2_Ph catalysts have not been computed). Note that all newly designed catalysts show improvement over Corey's original catalyst.

The trend of increasing selectivities in the experiments is consistent with our computational results but the absolute selectivities differ. While computations suggest an increase of selectivity of up to 98 % *ee* by introducing DEDs, the experimentally observed improvement is more moderate. There may be several reasons for this. First, the mechanism is more complex than accounted for in the computations. Second, the reduction features also a non‐negligible background reaction with BH_3_, which could have a larger impact on the modified catalysts, as the activity of these is lower compared to the original catalyst, because the EDG in the carbinol position reduces the Lewis acidity at boron.^[22]^ Third, the computations are not accurate enough as compared to highest level ab initio computations. This is certainly true but we are pleased to see trends with predictability leading to improved catalyst performance, in particular, for the most challenging of substrates.

**Figure 10:**
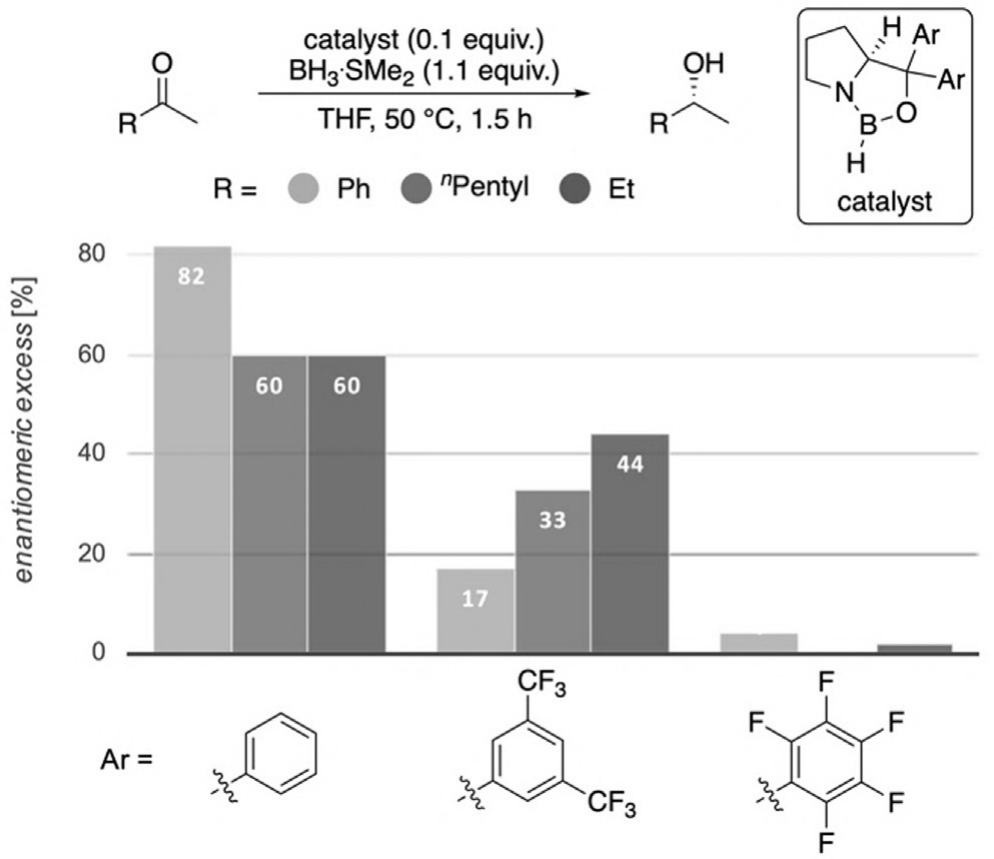
Reductions employing phenyl‐substituted and fluorinated catalysts relative to Corey's original catalyst (Ar=phenyl).

In order to also provide some counter examples, we included catalysts with 3,5‐(CF_3_)_2_Ph and C_6_F_5_ carbinol substituents and found that the fluorinated catalysts are much less selective (Figure 10), despite their high steric demand and significant activation of the boron Lewis acid. These results are in accord with the computed values (Supporting Information, Table S1) and suggest weakened LD interactions between the fluorinated aryl groups and the substrates, as we decrease attractive σ‐π interactions through the strongly electron withdrawing fluorine substituents.[^15c^, ^23^]

Moreover, this is consistent with the reduction of special substrates like pentafluorobenzophenone and *p*‐methoxy‐*p*‐nitro‐benzophenone (Table 3). In all cases, the enantiomer maximizing the attractive σ‐π interactions in the TS is favored. Also, the σ‐π interaction of a C_6_F_5_ substituent to a phenyl ring is lower than the σ‐π of two phenyl groups (entry 1).^[15c]^ For *p*‐methoxy‐*p*‐nitro‐benzophenone the electron‐rich aryl group (4‐OMe‐C_6_H_4_) provides the stronger interaction with the catalyst (entries 2 and 3). In the case of cyclopropyl isopropyl ketone (entry 4), the catalyst interacts with the more π‐electron rich cyclopropyl substituent favoring the *R* enantiomer. With trichloroacetophenone (entries 5 and 6), the more attractive σ‐π interaction results in *R* selectivity, as chloromethanes strongly interact with aryl rings due to LD (Supporting Information, Table S4).^[23a]^

**Table 3:** Reductions employing some challenging ketones. For further details, see the Supporting Information. 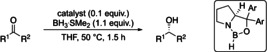

Entry	R^1^	R^2^	Cat. Ar	Config^[a]^ *ee* [%]
1	Ph	C_6_F_5_	Ph	*S* 92
2	4‐OMe‐C_6_H_4_	4‐NO_2_‐C_6_H_4_	Ph	*R* 56
3	4‐OMe‐C_6_H_4_	4‐NO_2_‐C_6_H_4_	4‐OMe‐3,5‐Me_2_Ph	*R* 62
4	*c*‐Pr	^*i*^Pr	Ph	*R* 91^[b]^
5	CCl_3_	Ph	Ph	*R* 27
6	CCl_3_	Ph	4‐OMe‐3,5‐Me_2_Ph	*R* 45

[a] Abs. configuration is based upon measurement of rotation and comparison with literature or computed values (Supporting Information). [b] Reaction as reported by Corey et al. with 15 mol % of catalyst and catecholborane as reducing agent at −78 °C.^[5f]^

**Figure 11:**
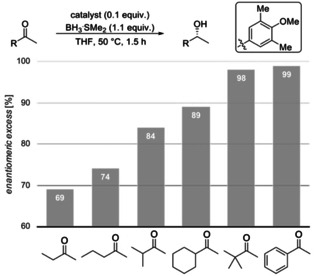
Reductions of various substrates employing our new modified CBS catalyst.

**Figure 12:**
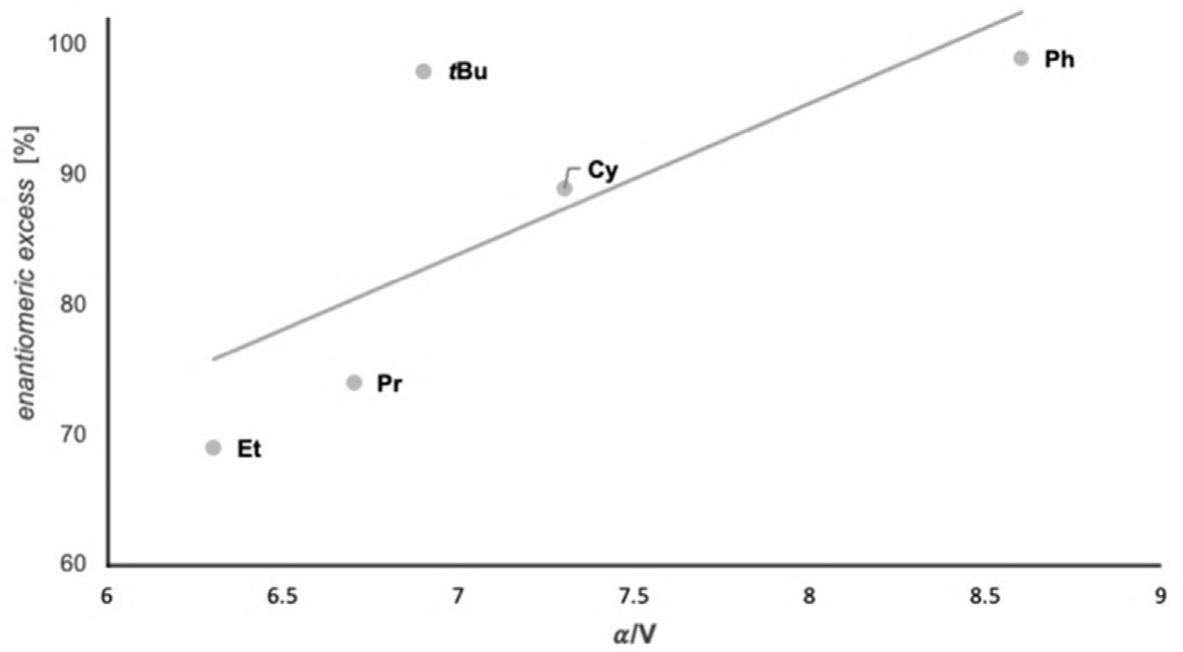
Increasing polarizability per volume *α*/V of the substrates typically leads to higher enantioselectivites in the reduction with a given catalyst (4‐OMe‐3,5‐Me_2_Ph). Computed values of polarizability (*α*) and volume (V) of the corresponding substituent. Level of theory: PBE0/aug‐cc‐pVDZ//B3LYP‐D3(BJ)/6–311G(d,p).^[24]^

Figure 11 summarizes the results for the reductions of a variety of ketones with our best modified catalyst. These data also indicate that enantioselectivities increase with the computed polarizabilities per volume *α*/V, resulting in a higher interaction energy of the substituent with the catalyst (Figure 12).^[15a]^

**Figure 13:**
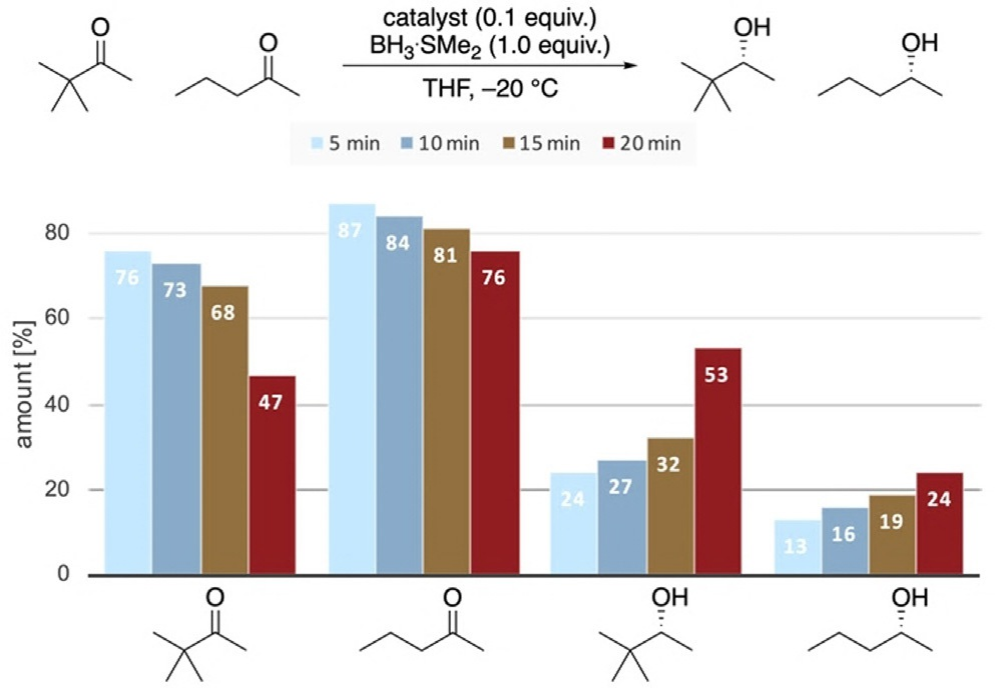
Ketone‐to‐alcohol ratios in the competitive reduction of *tert*‐butyl ketone and 2‐pentanone after the given reaction times.

These findings are confirmed by a competitive rate analysis in the reduction of 2‐pentanone and *tert*‐butyl methyl ketone in the same reaction flask (Figure 13). After the given reaction times, we took a small sample of the reaction mixture, quenched it in citric acid, and analyzed the conversion after work up. We chose *tert*‐butyl methyl ketone and 2‐pentanone, as they are reduced with quite different selectivities (Figure 11). While *tert*‐butyl methyl ketone is reduced in excellent selectivity, the reaction should proceed slowly because the neopentyl position is traditionally viewed as highly sterically encumbered, thereby hampering the attack of nucleophiles.^[25]^ Remarkably, the consumption of *tert*‐butyl methyl ketone occurs at a higher reaction rate than that of 2‐pentanone. Computations show that the complex of the catalyst with *tert*‐butyl methyl ketone has a similar energy as that with 2‐pentanone (Supporting Information, Figure S6). This implies that electrostatic interactions with catalyst are similar, and electronic effects are not significant. We conclude that stabilizing LD interactions in the TS are at work because the rate of the sterically more demanding substrate is higher.

## Conclusion

We present a combined computational and experimental exploration of the origin of the enantioselectivities in CBS reductions. Contrary to the current hypothesis that makes steric repulsion solely responsible for enantioselection, our computations reveal the presence of stabilizing noncovalent interactions in the hydride transfer transition structure. NCI plots qualitatively aided in visualizing these intermolecular interactions particularly between the substrate and the phenyl substituents of the catalyst. A quantitative SAPT analysis suggests that LD interactions tip the balance in favor of attractive noncovalent steric interactions to achieve high enantioselectivity.

Catalysts bearing DEDs in the *meta*‐positions of the aryl groups increase the enantioselectivity for different substrates, as confirmed computationally and experimentally. More polarizable substrates lead to stronger LD interactions with the catalyst and therefore to higher enantioselectivities as well as faster reaction rates. If steric repulsion were the chief selector, the rates would diminish with increasing selectivity—the opposite is the case. Even though the overall positive effect on enantioselectivity through the addition of DEDs is moderate, it provides strong evidence that the success of the CBS reduction is due to an excellent balance of attractive and repulsive steric interactions, with LD interactions being key to rationalizing the experimental findings. Our study therefore emphasizes that attractive LD interactions can and should be used as a modern catalyst design principle.

## Computational Methods

All computations were performed with the Gaussian16 or ORCA^[26]^ program suite. Geometries were optimized with dispersion corrections [DFT‐D3^[4a]^(BJ)^[4b]^] and without dispersion corrections in conjunction with the B3LYP functional combining a 6–311G(d,p) basis set. Vibrational frequencies were computed for each optimized structure to verify the stationary structures as minima or saddle points. Solvent effects were included by single‐point energy computations with the SMD model^[27]^ at the same level as for the optimized geometry. Higher level single‐point energies were computed by the domain‐based local pair natural orbital CCSD(T) (labeled DLPNO‐CCSD(T)) method with a cc‐pVTZ basis set. The SAPT analysis was performed at the SAPT0/jun‐cc‐pvdz level of theory on the optimized geometries^[28]^ utilizing the PSI4 code.^[29]^ Conformational analyses were performed using xtb (version 5.8) employing GFN2‐xTB by simulated annealing molecular dynamics (MD) simulations in the gas phase.^[30]^ All energies discussed are Gibbs free relative energies at 298.15 K and 1 atm in kcal mol^−1^ unless noted otherwise. Effects of zero‐point vibrational energy (ZPVE) corrections are included.

## Conflict of interest

The authors declare no conflict of interest.

## Supporting information

As a service to our authors and readers, this journal provides supporting information supplied by the authors. Such materials are peer reviewed and may be re‐organized for online delivery, but are not copy‐edited or typeset. Technical support issues arising from supporting information (other than missing files) should be addressed to the authors.

Supplementary
