# Supplementary material for: London Dispersion Interactions Rather than Steric Hindrance Determine the Enantioselectivity of the Corey–Bakshi–Shibata Reduction
Source: Angew Chem Int Ed Engl. 2021 Jan 28;60(9):4823–32. doi: 10.1002/anie.202012760 (PMC7986100; doi:10.1002/anie.202012760)
Supplement: Supplementary file 1 — Supplementary [file ANIE-60-4823-s001.pdf]

Angewandte Chemie
Eine Zeitschrift der Gesellschaft Deutscher Chemiker

Supporting Information

London Dispersion Interactions Rather than Steric Hindrance Determine the Enantioselectivity of the Corey–Bakshi–Shibata Reduction

*Christian Eschmann+, Lijuan Song+, and Peter R. Schreiner**

anie_202012760_sm_miscellaneous_information.pdf

Table of Contents

1. General Information 2
2. Analytical Methods 2
3. Potential Energy Hypersurface 3
4. Symmetry-Adapted Perturbation Theory (SAPT0) 5
5. London Dispersion Maps 6
5. General Procedure for the Oxazaborolidine (OXB) Catalyzed Reduction 7
6. Experimental Evaluation of Reaction Conditions 8
7. Experimental Evaluation of the Boron Substituent 9
8. Computational Evaluation of Fluorinated Catalysts 10
9. Evaluation of Special Substrates 10
10. Supplementary Computations to the Competitive Reduction 12
11. Synthetic Procedures and Analytical Data 13
   11.1. GC Analytics of Alcohols 13
   11.2. Synthesis of Substrates 17
   11.3. Miscellaneous 19
   11.4. Synthesis of Catalyst Precursors 21
12. NMR Spectra 41
13. Computational Data 63
14. References 244

1. General Information

Unless otherwise noted, chemicals were purchased from Acros Organics, TCI, Alfa Aesar, Lancaster, Merck, or Fluka at the highest purity grade available and were used without further purification. All solvents were distilled prior to use. Toluene, THF, and CH_2Cl_2 were distilled from appropriate drying agents prior to use and stored under argon atmosphere. All catalytic reactions were carried out under argon atmosphere employing oven- and flame-dried glassware. Column chromatography was conducted using Macherey Nagel silica gel 60 (0.040 – 0.063 mm),

2. Analytical Methods

Thin Layer Chromatography (TLC) was performed on silica coated plates (Macherey Nagel, silica 60 F254) with detection by UV-light (λ = 254 nm) and/or by staining with a with a cerium ammonium molybdate solution [CAM] followed by heat treatment.

CAM-staining solution: cerium sulfate tetrahydrate (1.00 g), ammonium molybdate (25.0 g) and concentrated sulfuric acid (25.0 mL) in water (250 mL).

Nuclear Magnetic Resonance Spectra were recorded at room temperature either on a *Bruker* AV-400 or a AV-400HD. ^{1}H NMR spectra were referenced to the residual proton signal of $CDCl_3$ (δ = 7.26 ppm) or CD_2Cl_2 (δ = 5.32 ppm). ^{13}C NMR spectra were referenced to the ^{13}C-D triplet of $CDCl_3$ (δ = 77.16 ppm) or CD_2Cl_2 (δ = 53.84 ppm). The following abbreviations for single multiplicities were used: m-multiplet, s-singlet, d-doublet, t-triplet, q-quartet, quint-quintet, sept-septet, dd-doublet of doublets, dt-doublet of triplets, qd-quartet of doublets.

High resolution mass spectrometry (HRMS) was performed employing a Thermo Scientific LTQ FT Ultraspectrometer (ESI) using methanol solutions of the respective compounds.

Chiral Gas Chromatography (GC): Enantioselectivities were determined by chiral stationary phase GC analyses on Hewlett Packard 5890 or 6890 gas chromatographs, respectively.

3. Potential Energy Hypersurface

We computed the reaction pathway for the reduction of acetophenone using a comparison of B3LYP/6-311G(d,p) and B3LYP-D3(BJ)/6-311G(d,p) (Fig. S1) to estimate the dispersion correction. ΔG_{298K} values are discussed unless noted otherwise. The conformational analysis was performed manually. There are four conformations in the hydride transfer step, regarding the direction of the coordinated oxygen lone pair (chair-like vs. boat-like) and the geometry (R vs S) as in Figure S2. The chair-like transition state with the oxygen lone pair *anti* to the larger group (Ph) is favored. In addition, for those substrates or catalysts with more than one conformation, a conformational search was carried out using xtb employing GFN2-xTB with simulated annealing molecular dynamics (MD) simulations.

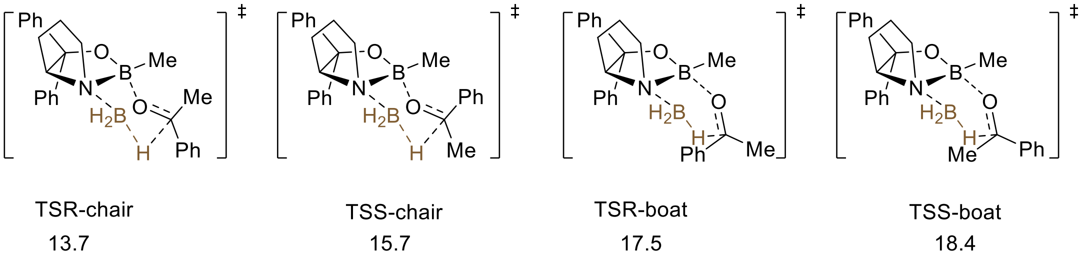

Figure S1. Conformations for the hydride transfer step.

Oxazaborolidine, BH_3, and acetophenone were used as reference point **1** at 0.0 kcal mol^{-1}. Coordination of one solvent molecule (THF) would form complex **1'**, which is higher in free energy. Initial coordination of BH_3 to the oxazaborolidine leads to a 5.5 kcal mol^{-1} more stable complex **2**. Coordination of the ketone to give complex **3** is endergonic and exhibits an energy barrier of 15.0 kcal mol^{-1}. Here a solvent molecule (THF) competes with acetophenone to form **2'**. The subsequent hydride transfer is the most important step in the reaction, which determines enantioselectivity. Without dispersion correction **TS1'$_S$** and **TS1'$_R$** are found to be very high in energy exhibiting relative energies of 29.8 kcal mol^{-1} and 31.7 kcal mol^{-1} respectively, related to the starting point **1**. These energy barriers seem to be too high for such a fast catalyzed reaction at temperatures of 298 K. Including dispersion correction, the relative energies of all intermediates and transition structures decrease, presenting larger stabilizing intermolecular interactions. Especially the transition structures **TS1$_R$** and **TS1$_S$** are notably lower in energy with barriers of 13.7 kcal mol^{-1} and 15.7 kcal mol^{-1} respectively, which seem much more reasonable for a catalyzed reaction. The calculated enantioselectivity of the reduction, which is expressed in the energy difference between the transition

structures ($\Delta\Delta G^{\ddagger}$) of the hydride transfer, is −2.0 kcal mol^{-1} and thereby consistent with previously published experimental results (−2.2 kcal mol^{-1}).[1] After the hydride transfer, intermediate **4R** with an open ring structure forms. There are two possible pathways for product release **7** and catalyst regeneration. The direct release *via* **TS2$_R$** towards **5R** is less preferred (+3.5 kcal mol^{-1}) than *via* **TS3$_R$** by addition of an additional equivalent of borane to **6R**.

When employing DLPNO-CCSD(T)/cc-pVTZ to compute the electronic energies of **TS1$_S$** and **TS1$_R$**, the free energies (corrected for ZPVE) of the TS are 7.7 kcal mol^{-1} and 10.8 kcal mol^{-1}, respectively. This indicates the dispersion-corrected energy barrier is more reasonable and that more complete inclusion of electron correlation effects emphasize the importance of LD even more.

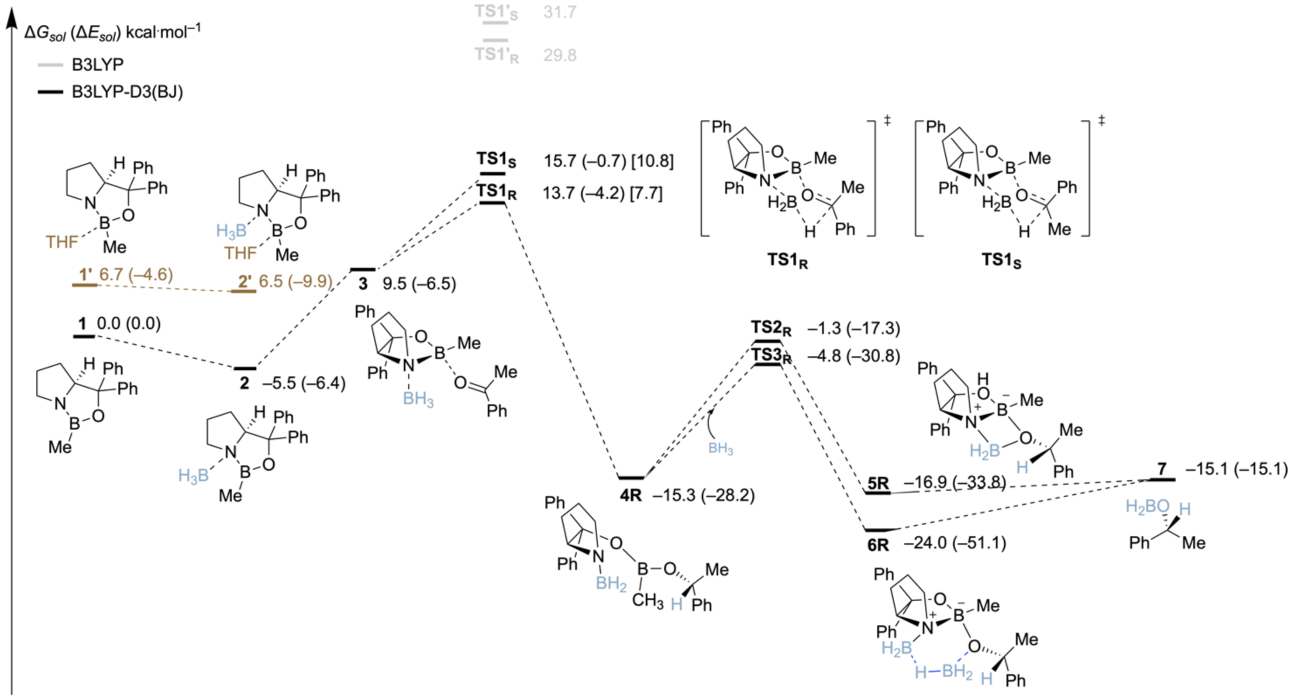

Figure S2. Potential energy surface containing free energies ($\Delta G_{\text{sol}}^{275}$) electronic energies ($\Delta E_{\text{sol}}$ in parentheses) of the CBS pathway with and without dispersion corrections. Level of theory: B3LYP-D3(BJ)/6-311+G(d,p)-SMD(THF)// B3LYP-D3(BJ)/6-311G(d,p). The free energies in brackets are based on electronic DLPNO-CCSD(T)/cc-pVTZ single-point energy (corrected for ZPVE).

4. Symmetry-Adapted Perturbation Theory (SAPT0)

SAPT(0) analysis was employed to determine the noncovalent interaction energies of the transition structures. The structures for SAPT0 were optimized at B3LYP-D3(BJ)/6-311G(d,p). Then the SAPT module of the PSI4 code was employed in conjunction with a jun-cc-pvdz basis set.

Table S1. SAPT0 analysis of the TS of the CBS reduction of acetophenone.

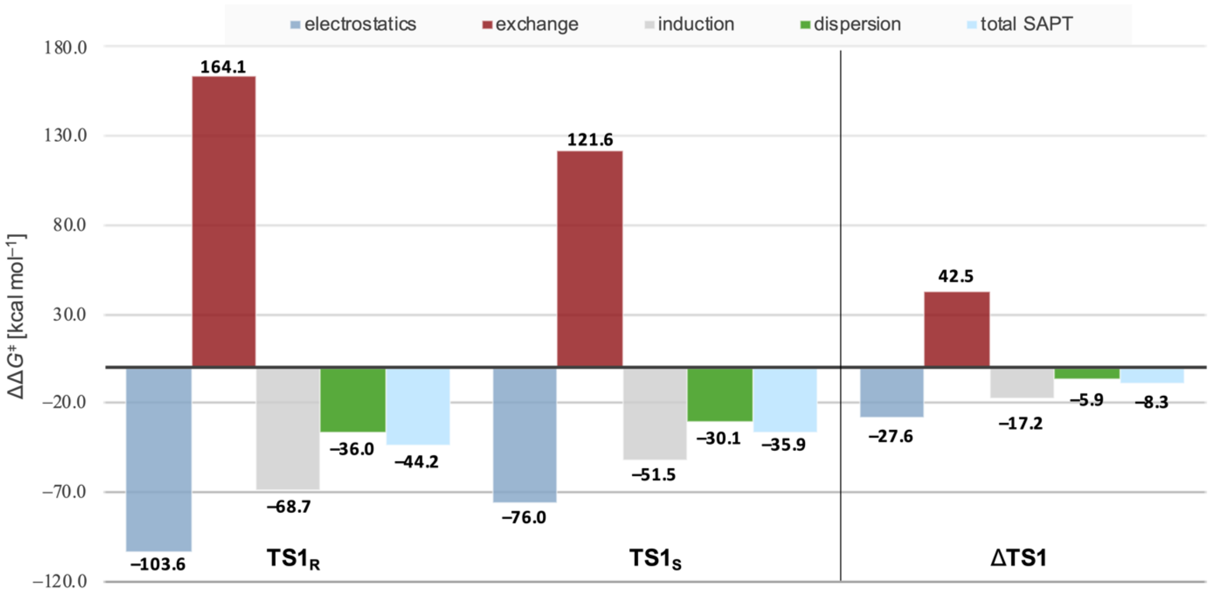

	$TS1_R$	$TS1_S$	$\Delta TS1_R$
electrostatics	-103.6	-76.0	-27.6
exchange	164.1	121.6	42.5
induction	-68.7	-51.5	-17.2
dispersion	-36.0	-30.1	-5.9
total SAPT	-44.2	-35.9	-8.3

All energies are provided in kcal mol^{-1}

5. London Dispersion Maps

Besides NCI plots (Fig. 2) we used recent developed LD potential maps by Pollice and Chen to directly visualize LD interactions.[2] Attractive LD interactions are indicated with red areas, which are observed intermolecularly between catalyst and substrate. Larger red areas, indicating stronger LD interactions can be observed in the favored **TS1$_R$** between the phenyl group of the catalyst and the phenyl group of the substrate (Fig. S3). These computations qualitatively confirm the NCI plots (Fig. 2) and the SAPT analysis (Fig. 3) and also suggest that LD interactions are important for enantiodiscrimination.

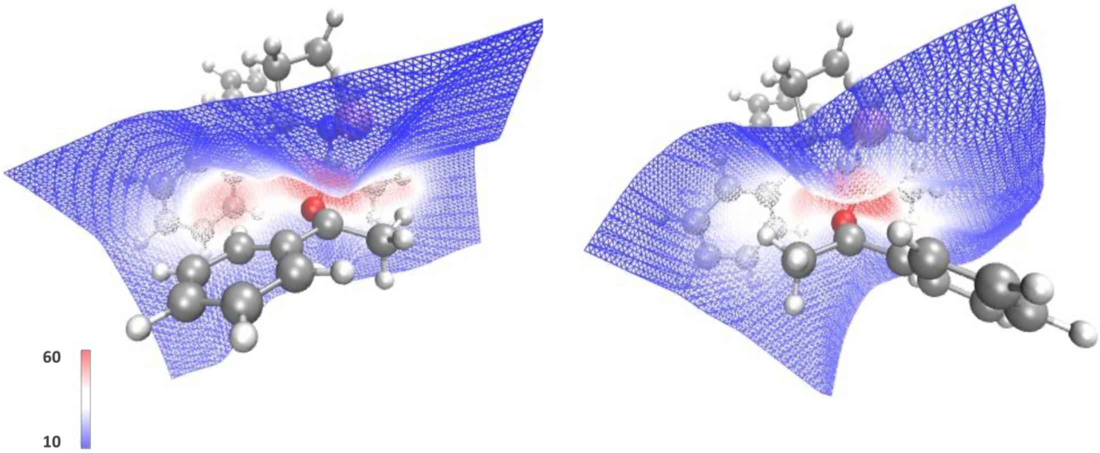

TS1$_R$ **TS1$_S$**

Figure S3. London dispersion maps (values are in kcal$^{0.5}$ mol$^{-0.5}$) of the transition structures **TS1$_R$** and **TS1$_S$** in the reduction of acetophenone. Red areas show more stabilizing LD interactions.

5. General Procedure for the Oxazaborolidine (OXB) Catalyzed Reduction

The respective amino alcohol as catalyst precursor (0.040 mmol, 0.1 equiv.) was placed in a flame dried Schlenk tube under Ar. Anhydrous solvent (1 mL) and $BH_3{\cdot}SMe_2$ (0.440 mmol, 1.1 equiv.) were added and the reaction was stirred at 50 °C for 30 min. A solution of the ketone (0.400 mmol, 1.0 equiv. in 0.5 mL THF) was added *via* a syringe pump within 30 min. The reaction mixture was stirred for another 60 min at 50 °C. The reaction was quenched by addition of 6.0 mL citric acid [0.5 M] and the mixture was extracted with EtOAc (3 × 5 mL). The combined organic layers were washed with brine, dried over Na_2SO_4, filtered, and the solvent was carefully removed under reduced pressure. Alcohols whose enantiomers could not be separated by chiral GC, were directly derivatized either by addition of Ac_2O (0.042 mL, 0.440 mmol, 1.1 equiv.), DMAP (0.005 g, 0.044 mmol, 0.1 equiv.), and Et_3N (0.041 mL, 0.440 mmol, 1.1 equiv.) or benzoyl chloride (0.051 mL, 0.440 mmol, 1.1 equiv.).

6. Experimental Evaluation of Reaction Conditions

We started our investigation employing the 3,5-*t*Bu_2Ph catalyst in the reduction of 2-butanone (Tab. S2). In all cases, at 50 °C after 1.5 h the reduction resulted in near quantitative yields. We chose a slightly elevated temperature, because the boron reduction often proceeds with better enantioselectivity at higher temperatures (entries 1 and 2).[3] We could not improve the enantioselectivity by changing the solvent to PhMe or CH_2Cl_2 (entries 3 and 4) or by lowering the concentration (entry 5). Furthermore, there was no difference in selectivity when using BH_3·THF instead of BH_3·SMe_2 (entries 2 and 6). The best selectivity was achieved when adding the ketone slowly *via* a syringe pump to the reducing mixture (entries 2, 7, and 8).

Table S2. Investigation of the reaction conditions.

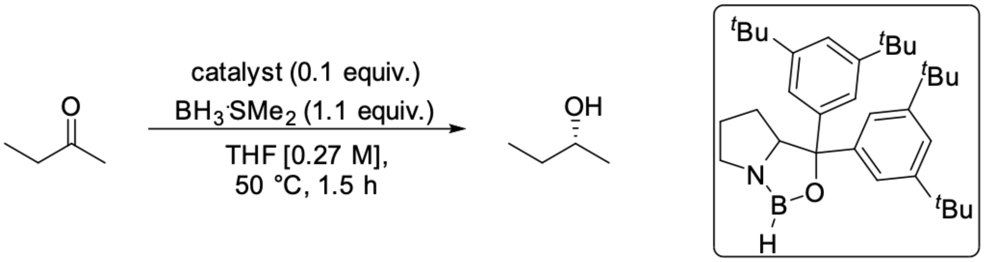

Entry	Solvent	*ee* [%]
**1**[a]	THF	53
**2**	THF	67
**3**	PhMe	67
**4**	CH_2Cl_2	42
**5**[b]	THF	64
**6**[c]	THF	67
**7**[d]	THF	60
**8**[e]	THF	44

[a] Reaction performed at 0 °C.
[b] THF [0.1 M].
[c] BH_3·THF used as reducing agent.
[d] Without syringe pump.
[e] BH_3·SMe_2 added *via* syringe pump.

7. Experimental Evaluation of the Boron Substituent

We investigated the effect of the substituent at boron computationally (Tab. 2) and experimentally (Fig. S4). Computations suggest that in the reduction of cyclohexyl methyl ketone LD interactions can be increased using Cy or CH_2Cy with selectivities ($\Delta\Delta G^{\ddagger}$) of 2.3 kcal mol^{-1} and 3.5 kcal mol^{-1} respectively (Tab. 2 entries 1 and 2). We synthesized the corresponding oxazaborolidines *in situ via* azeotropic distillation following a common literature protocol with a Dean-Stark trap.[4] We employed the catalysts in the reduction of three different ketones. The results show that the selectivity does not change much as compared to the original catalyst when using CH_2Cy and Cy groups (Fig. S4). This implies that the interactions between substrate and the substituent at boron on the catalyst only have a subtle effect on the enantioselectivity.

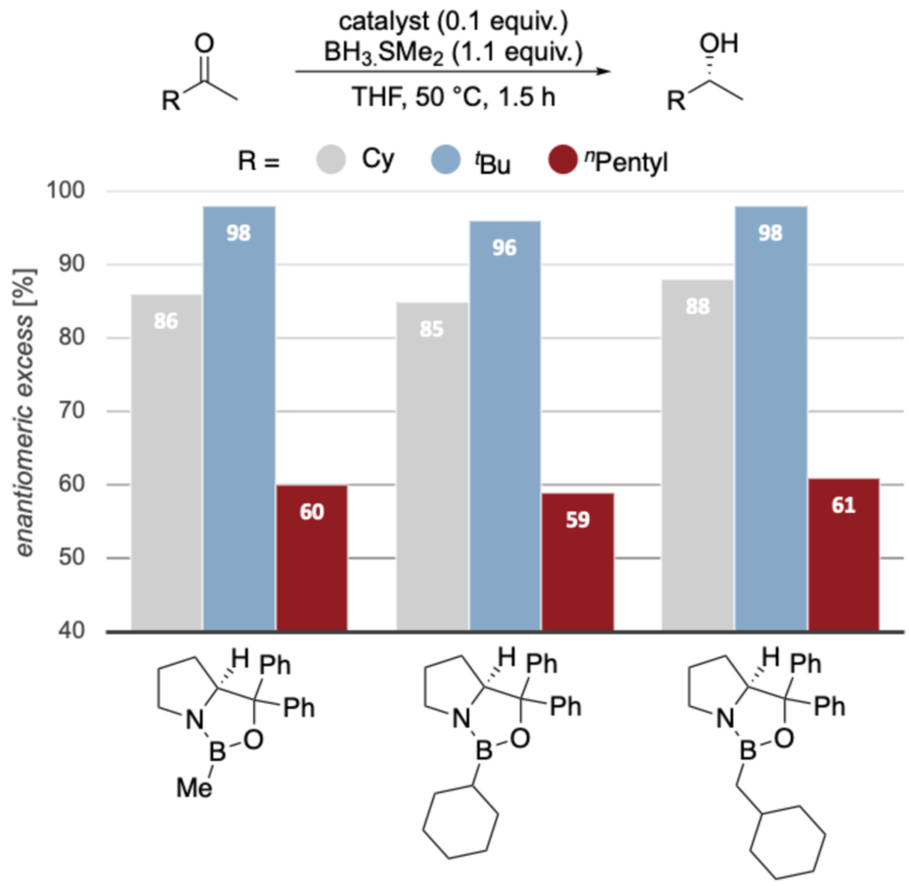

Figure S4. Reductions employing modified CBS catalysts with different boron substituents.

8. Computational Evaluation of Fluorinated Catalysts

We also employed catalysts with 3,5-$(CF_3)_2$Ph and C_6F_5 carbinol substituents and found experimentally that the fluorinated catalysts are less selective (Fig. 10). These results are in accord with computed values (Tab. S2), as attractive $\sigma - \pi$ interactions decrease through strongly electron withdrawing fluorine substituents.[5]

Table S2. Calculated enantioselectivities ($\Delta\Delta G^{\ddagger}_{\text{sol}}$) in the reduction of 2-butanone employing fluorinated catalysts. Level of theory: B3LYP-D3(BJ)/6-311G(d,p).

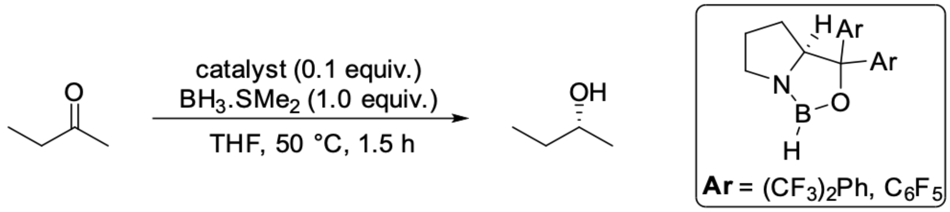

Entry	Catalyst	$\Delta\Delta G^{\ddagger}_{sol}$ (with D3)
**1**	CF_3	0.2
**2**	C_5F_6	0.3

$\Delta\Delta G^{\ddagger}_{\text{sol}} = \Delta\Delta G^{\ddagger}_{\text{sol}}(\text{TS}_\text{S}) - \Delta\Delta G^{\ddagger}_{\text{sol}}(\text{TS}_\text{R})$, at reaction temp.; (kcal mol^{-1})

9. Evaluation of Special Substrates

In the reduction of pentafluorobenzophenone we achieved 92% *ee* for the (*S*) enantiomer (Fig. S5). This is consistent with our results for the fluorinated catalysts (Tab S2) as we still have attractive T-shaped $\sigma - \pi$ interactions between the phenyl of substrate and catalyst. Accordingly the $\sigma - \pi$ interaction of a C_6F_5 substituent to a phenyl is meant to be lower than the $\sigma - \pi$ of two phenyl groups.[5b] The bulky and electron deficient pentafluorophenyl takes the position of the former Me group, which is in contrast to the traditional model based on steric repulsion.

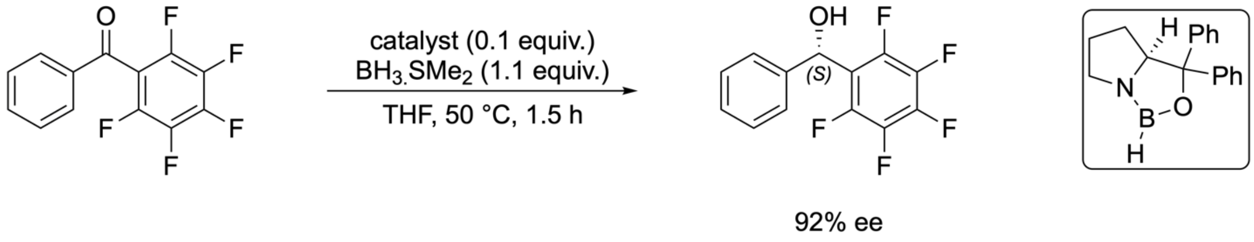

Figure S5. Reduction of pentafluorobenzophenone.

The reduction of *p*-methoxy-*p'*-nitrobenzophenone yielded the (*R*) enantiomer in moderate selectivities (Tab S3). The electron enriched aryl group (4-OMe-C_6H_4) provides the higher interaction with the catalyst. These results are consistent with the reduction of pentafluoro-benzophenone as both electron deficient aryl groups (pentafluorophenyl, *p'*-nitrophenyl) point away from the catalysts' phenyl groups because of a weakened noncovalent interaction.

Table S3. Reduction of *p*-methoxy-*p'*-nitrobenzophenone

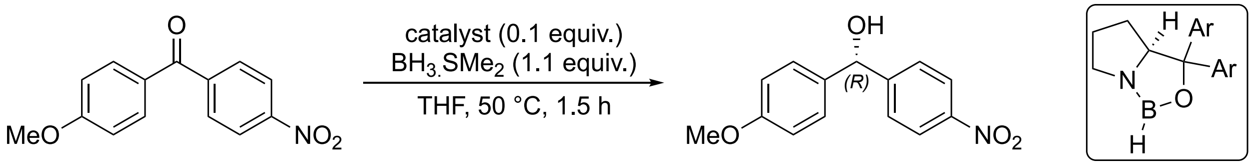

Entry	Catalyst Ar	*ee* [%]
**1**	Ph	(*R*) 56
**2**	4-OMe-3,5-Me_2Ph	(*R*) 62

In the reduction of trichloroacetophenone employing catecholborane as reducing agent at –78 °C Corey achieved predominantly the (*R*) enantiomer.[6] Comparing this to acetophenone, the geometries of the **TS1$_R$** and **TS1$_S$** (Fig. 2) are swapped, as for stereochemistry the trichloromethyl has the higher priority than phenyl. When using the standard conditions with $BH_3 \cdot SMe_2$ we also obtained the (*R*) enantiomer but with lower selectivity (Tab. S4 entry 1). With the modified version of the CBS catalyst (entry 3), the selectivity increased to 45% *ee*. Our computations suggest a preference for the (*R*) enantiomer (entry 2), because of stabilizing interactions between the trichloromethyl group with the π-system of the catalyst, originating from London dispersion (LD).[5c]

Table S4. Reduction of 2,2,2-Trichloro-1-phenylethanone

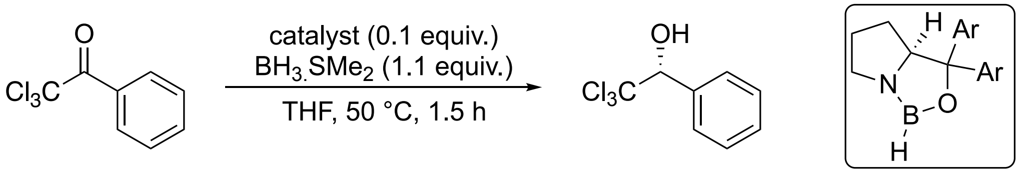

Entry	Catalyst Ar	*ee* [%]
**1**	Ph	(*R*) 27
**2**	Ph[a]	(*R*) 18
**3**	4-OMe-3,5-Me_2Ph	(*R*) 45

[a] Selectivity computed at B3LYP-D3(BJ)/6-311G(d,p)

10. Supplementary Computations to the Competitive Reduction

In a competitive experiment we observed that *t*Bu methyl ketone is reduced faster than 2-pentanone (Fig 13). We conclude that stabilizing interactions in the TS are responsible for that rate difference, because reduction at the neopentyl position of *t*Bu ketone should proceed with a lower rate. The tBu ketone is more electron-rich, so it may form a stronger interaction with the catalyst. However, our computations show that the two catalyst-ketone complexes have very similar binding energies. This implies that differential electronic effects are not significant. As *t*Bu ketone is also reduced with higher selectivity (Fig. 11) and possesses a higher polarizability per Volume α/V (Fig. 12), we conclude that stabilizing LD interactions in the TS must be responsible for these observations.

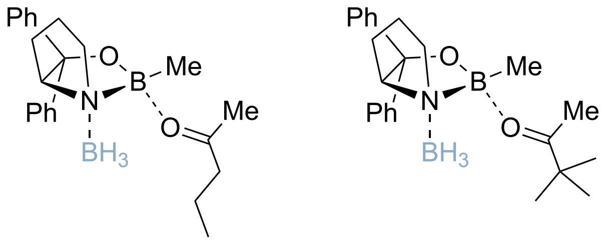

7.8 kcal mol^{-1} 7.4 kcal mol^{-1}

Fig. S6 Computed energies of the complex structures. Level of theory: B3LYP-D3(BJ)/6-311G(d,p).

Furthermore, the SAPT analysis of the transition structures suggests, that the tBu ketone has higher dispersion energy than 2-pentanone, therefor leading to a lower total SAPT.

Table S5 Computed energies of the complex structures. Level of theory: B3LYP-D3(BJ)/6-311G(d,p).

Transition structures	Electrostatics	Exchange	Induction	Dispersion	Total SAPT
2-pentanone TS_R	−94.3	142.8	−60.7	−30.8	−43.0
tBu ketone TS_R	−97.1	147.7	−62.4	−32.5	−44.2

All energies are provided in kcal mol^{-1}; Level of theory: SAPT0/jun-cc-pvdz.

11. Synthetic Procedures and Analytical Data

11.1. GC Analytics of Alcohols

2-Butanol

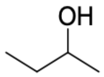

Enantioselectivity was determined by investigation of the benzoylated alcohol *via* chiral GC employing a 30 m FS-Hydrodex β-6TBDM column (Macherey Nagel).

T (Injector + Detector) = 250 °C

Splitflow = 80 mL min^{-1}

Precolumn pressure = 0.8 bar

Conditions: 100 °C – 140 °C, 2 °C min^{-1}

Retention Times: (*R*) = 14.3 min; (*S*) = 14.5 min

2-Pentanol

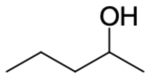

Enantioselectivity was determined by investigation of the benzoylated alcohol *via* chiral GC employing a 30 m FS-Hydrodex β-6TBDM column (Macherey Nagel).

T (Injector + Detector) = 250 °C

Splitflow = 80 mL min^{-1}

Precolumn pressure = 0.8 bar

Conditions: 80 °C, 10 min; 80 – 120 °C, 0.5 °C min^{-1}

Retention Times: (*R*) = 63.2 min; (*S*) = 64.0 min

2-Heptanol

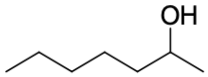

Enantioselectivity was determined by investigation of the acylated alcohol *via* chiral GC employing a 30 m FS-Hydrodex β-6TBDM column (Macherey Nagel).

T (Injector + Detector) = 250 °C

Splitflow = 80 mL min^{-1}

Precolumn pressure = 0.8 bar

Conditions: 100 °C – 120 °C, 2 °C min^{-1}

Retention Times: (*R*) = 4.2 min; (*S*) = 4.4 min

3-Methyl-2-butanol

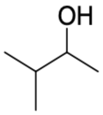

Enantioselectivity was determined *via* chiral GC employing a 30 m FS-Hydrodex β-6TBDM column (Macherey Nagel).

T (Injector + Detector) = 250 °C

Splitflow = 80 mL min^{-1}

Precolumn pressure = 0.8 bar

Conditions: 80 °C, 10 min

Retention Times: (*R*) = 5.8 min; (*S*) = 6.1 min

3,3-Dimethyl-2-butanol

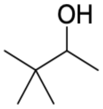

Enantioselectivity was determined *via* chiral GC employing a 30 m FS-Hydrodex β-6TBDM column (Macherey Nagel).

T (Injector + Detector) = 250 °C

Splitflow = 80 mL min^{-1}

Precolumn pressure = 0.8 bar

Conditions: 60 °C, 10 min

Retention Times: (*R*) = 5.3 min; (*S*) = 5.5 min

1-Cyclohexylethanol

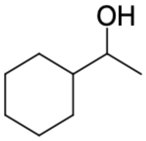

Enantioselectivity was determined *via* chiral GC employing a 30 m FS-Hydrodex β-TBDAc column (Macherey Nagel).

T (Injector + Detector) = 250 °C

Splitflow = 80 mL min^{-1}

Precolumn pressure = 0.8 bar

Conditions: 80 °C, 20 min; 80 – 120 °C, 2 °C min^{-1}

Retention Times: (*R*) = 26.9 min; (*S*) = 27.4 min

1-Phenylethanol

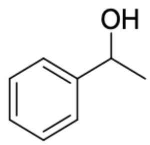

Enantioselectivity was determined *via* chiral GC employing a 30 m FS-Hydrodex β-6TBDM column (Macherey Nagel).

T (Injector + Detector) = 250 °C

Splitflow = 80 mL min^{-1}

Precolumn pressure = 0.8 bar

Conditions: 100 °C, 20 min

Retention Times: (*R*) = 10.8 min; (*S*) = 11.2 min

2,2,2-Trichloro-1-phenylethanol

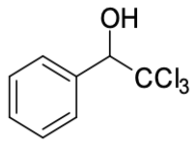

Enantioselectivity was determined by investigation of the acylated alcohol *via* chiral GC employing a 30 m FS-Hydrodex β-6TBDM column (Macherey Nagel).

T (Injector + Detector) = 250 °C

Splitflow = 80 mL min^{-1}

Precolumn pressure = 0.8 bar

Conditions: 100 °C – 190 °C, 2 °C min^{-1}

Retention Times: (*R*) = 26.5 min; (*S*) = 26.7 min

$[\alpha]_D^{24}$ = −18.4 ([0.77 M] in $CHCl_3$) 40% *ee* (*R*) (lit.[7] $[\alpha]_D^{20}$ = −27.0 ([1.0 M] in EtOH) 56% *ee* (*R*))

Computed value with Boltzmann weighted conformers:

(*R*) $[\alpha]_D^{25}$ = −56.4 (in $CHCl_3$) [B3LYP/6-311G(d,p)]

2,3,4,5,6-Pentafluoro-α-phenylbenzenemethanol

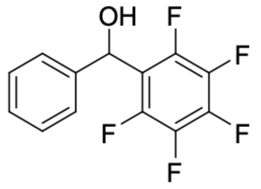

Enantioselectivity was determined *via* chiral HPLC employing a Chiralpak IC column (Daicel).

Detector (UV/VIS) = 254 nm

Flow = 1.0 mL min^{-1}

Eluent ^{n}hexane (97%), ^{i}PrOH (3%)

Retention Times: (*R*) = 5.3 min; (*S*) = 6.0 min

$[\alpha]_D^{20}$ = −42.2 ([0.78 M] in $CHCl_3$) 92% *ee* (*S*) (lit.[8] $[\alpha]_D^{20}$ = −45.0 ([1.90 M] in $CHCl_3$) 99% *ee* (*S*))

Computed value with Boltzmann weighted conformers

(*S*) $[\alpha]_D^{25}$ = −42.6 (in $CHCl_3$) [B3LYP/6-311G(d,p)]

4-Methoxyphenyl-4-nitrophenylmethanol

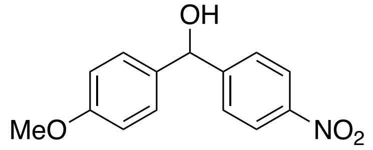

Enantioselectivity was determined *via* chiral HPLC employing a Chiralpak IA column (Daicel).

Detector (UV/VIS) = 270 nm

Flow = 1.0 mL min^{-1}

Eluent ^{n}hexane (75%), EtOAc (25%)

Retention Times: (*S*) = 9.5 min; (*R*) = 11.2 min

$[\alpha]_D^{24}$ = +48.5 ([0.50 M] in EtOH) 62% *ee* (*R*) (lit.[8] $[\alpha]_D^{20}$ = +43.1 ([1.14 M] in $CHCl_3$) 79% *ee* (*R*))

11.2. Synthesis of Substrates

2,3,4,5,6-Pentafluoro-α-phenylbenzenemethanol

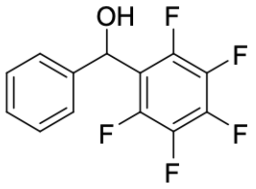

To a solution of pentafluoroiodobenzene (3.29 g, 11.2 mmol, 1.0 equiv.) in dry Et_2O under Ar at −78 °C, ^{n}BuLi in hexane [1.6 M] (7.00 mL, 11.2 mmol, 1.0 equiv.) was added dropwise. After stirring at −78 °C for 2 h benzaldehyde (1.13 mL, 11.2 mmol, 1.0 equiv.) was added and the solution was stirred at −78 °C for 3 h. Saturated NH_4Cl solution was added and the mixture was extracted with EtOAc (3 × 70 mL). The combined organic layers were washed with brine, dried over Na_2SO_4, filtered, and the solvent was removed under reduced pressure, to yield 2,3,4,5,6-Pentafluoro-α-phenylbenzenemethanol as colorless oil (2.71 g, 9.88 mmol, 89%). The product was used without further purification.

^{1}H NMR (400 MHz, $CDCl_3$): δ/ppm = 7.43 – 7.29 (m, 5H), 6.25 (s, 1H), 2.64 (s, 1H).

^{13}C NMR (101 MHz, $CDCl_3$): δ/ppm = 146.0 (m, CF), 143.5 (m, CF), 142.4 (m, CF), 140.7 (C), 139.0 (m, CF), 136.6 (m, CF), 128.9 (CH), 128.5 (CH), 125.5 (CH), 67.8 (CHOH).

The NMR spectra are in accordance with those reported in the literature.[8]

2,3,4,5,6-Pentafluorobenzophenone

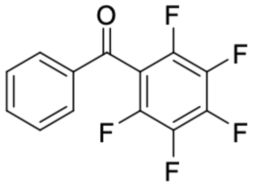

To a solution of 2,3,4,5,6-Pentafluoro-α-phenylbenzenemethanol (1.00 g, 3.65 mmol, 1.00 equiv.) in 10 mL CH_2Cl_2 was added pyridinium chlorochromate (PCC) (1.18 g, 5.47 mmol, 1.50 equiv.). The suspension was stirred at r.t. for 2 h, then filtered through a pad of Celite and washed with small portions of Et_2O. The solvent was removed under reduced pressure and the crude product was purified by column chromatography (silica, Hex/EtOAc = 40/1), to yield 2,3,4,5,6-Pentafluorobenzophenone as a colorless oil (0.721 g, 2.65 mmol, 73%).

TLC: *R*f = 0.21 (Hex/EtOAc = 40/1) [UV, CAM].

^{1}H NMR (400 MHz, $CDCl_3$): δ/ppm = 7.85 (dd, J = 8.3, 1.3 Hz, 2H), 7.72 – 7.65 (m, 1H), 7.57 – 7.49 (m, 2H).

^{13}C NMR (101 MHz, $CDCl_3$): δ/ppm = 185.4 (C), 145.2 (m, CF), 143.9 (m, CF), 142.7 (m, CF), 139.1 (m, CF), 136.1 (C), 135.2 (CH), 129.9 (CH), 129.2 (CH).

HRMS (ESI): calcd for $C_{13}H_5F_5O$ $[M+H]^+$: 273.0333; found: 273.0334

The NMR spectra are in accordance with those reported in the literature.[9]

2,2,2-Trichloro-1-phenylethanone

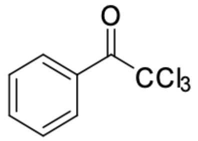

A solution of TCCA (9.30 g, 40.0 mmol, 2.0 equiv.) and acetophenone (2.36 g, 20.0 mmol, 1.0 equiv.) in 40 mL acetic acid was refluxed for 5 hr. After cooling, the precipitate was filtered and washed with acetic acid, the filtrate was diluted with water, and the mixture extracted with 4 x 40 mL pentane. The pentane solution was washed with brine and dried over Na_2SO_4. After filtration, the pentane was removed under reduced pressure and the residue purified by column chromatography (silica, Hex/EtOAc = 1/0 → 40/1), to yield 2,2,2-Trichloro-1-phenylethanone as a colorless liquid (3.89 g, 17.4 mmol, 87%).

TLC: *R*f = 0.20 (Hex) [UV].

^{1}H NMR (400 MHz, $CDCl_3$): δ/ppm = 8.28 – 8.25 (m, 2H), 7.68 – 7.62 (m, 1H), 7.53 – 7.47 (m, 2H).

^{13}C NMR (101 MHz, $CDCl_3$): δ/ppm = 181.4 (C), 134.4 (CH), 131.7 (CH), 129.3 (C), 128.6 (CH), 95.6 (C).

The NMR spectra are in accordance with those reported in the literature.[10]

11.3. Miscellaneous

4-Bromo-2,6-diisopropylaniline

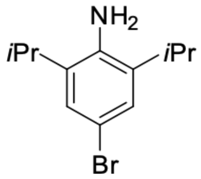

A solution of Br_2 (2.1 ml, 41.0 mmol, 1.05 equiv.) in CH_2Cl_2 /MeOH (100 ml, 1/1) was added to a stirring solution of 2,6-diisopropylaniline (7.4 ml, 39.0 mmol, 1.0 equiv.) in CH_2Cl_2/MeOH (200 mL, 1/1) at r.t. over 2 h. The red solution was stirred for 24 h. The solvents were evaporated, and the resultant red solid was washed with PE 35-70 and further recrystallized from CH_2Cl_2/PE 35-70 to yield a colorless crystalline solid. 100 mL of CH_2Cl_2 were added and the organic phase was washed three times with small portions of 2 M aqueous sodium hydroxide solution and with brine. The organic phase was dried over Na_2SO_4, filtered and the solvent was removed under reduced pressure to yield the title compound as a colorless liquid (9.98 g, 38.9 mmol, 95%).

^{1}H NMR (400 MHz, $CDCl_3$): δ/ppm = 7.11 (s, 2H), 3.71 (s, 2H), 2.88 (hept, *J* = 6.8 Hz, 2H), 1.25 (d, *J* = 6.8 Hz, 12H).

^{13}C NMR (101 MHz, $CDCl_3$): δ/ppm = 139.4 (C), 134.8 (C), 125.9 (CH), 111.3 (C), 28.2 (CH), 22.4 (CH_3).

The NMR spectra are in accordance with those reported in the literature.[11]

1-Bromo-3,5-diisopropylbenzene

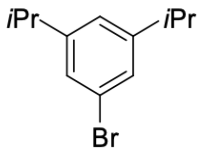

Sodium nitrite (4.45 g, 64.5 mmol, xxx equiv.) was added in portions to a suspension of 4-Bromo-2,6-diisopropylaniline (6.6 g, 26.0 mmol, 1.00 equiv.) in HCl [2 M] (70 mL) at –5 °C. The reaction was allowed to react for 10 min at –5 °C and then 50% H_3PO_2 (30 mL, 258 mmol, 10.0 equiv.) was added. The reaction mixture was left at 4 °C for 24 h and then at room temperature for 24 h. The aqueous layer was extracted with Et_2O (3 × 100 mL). The combined organic layers were dried over Na_2SO_4, filtered and the solvent was removed under reduced pressure to yield 1-Bromo-3,5-diisopropylbenzene as a light red liquid (6.08 g, 36.9 mmol, 97%).

TLC: *R*f = 0.35 (Hex/EtOAc = 3/1) [UV, CAM].

^{1}H NMR (400 MHz, $CDCl_3$): δ/ppm = 7.18 (d, *J* = 1.5 Hz, 2H), 7.00 – 6.96 (m, 1H), 2.85 (hept, *J* = 6.9 Hz, 2H), 1.23 (d, *J* = 6.9 Hz, 12H).

^{13}C NMR (101 MHz, $CDCl_3$): δ/ppm = 151.2 (C), 127.0 (CH), 123.9 (CH), 122.5 (C), 34.2 (CH), 24.0 (CH_3).

HRMS (ESI): calcd for $C_{12}H_{18}Br$ $[M+H]^+$: 241.0586; found: 241.0584.

The NMR spectra are in accordance with those reported in the literature.[12]

11.4. Synthesis of Catalyst Precursors

General Procedure 1: Grignard Addition to Benzylprolinesters

To a suspension of magnesium (2.50 equiv.) in anhydrous THF under Ar was added a crystal of iodine and stirred at room temperature for 30 min. Then 5% of the solution of aryl bromide (2.50 equiv.) in THF was added and the reaction mixture was warmed to 50 °C. As soon as the color changed from brown to pale yellow, the remaining solution of aryl bromide was added dropwise *via* an addition funnel (0.1 mL min^{-1}). The reaction mixture was refluxed for 1 h and then cooled to 0 °C.

To the arylmagnesium bromide suspension, a solution of (*S*)-methyl-1-benzylpyrrolidin-2-carboxylate (1.00 equiv.) in THF was added dropwise (0.1 mL min^{-1}). The reaction mixture was stirred for the appropriate amount of time, monitored by TLC analysis. At 0 °C, saturated NH_4Cl solution was added to quench the reaction. The mixture was then extracted three times with ethyl acetate. The combined organic layers were washed with brine, dried over Na_2SO_4, filtered, and the solvent was removed under reduced pressure. The crude product was purified by column chromatography.

General Procedure 2: Hydrogenolysis of Benzyl Group

The benzyl-protected prolinol was dissolved in methanol and palladium on charcoal (10–20 wt%) was added. The reaction flask was purged with hydrogen gas. After stirring 24 h at room temperature the reaction mixture was filtered through a pad of Celite and washed with small portions of methanol. The solvent was removed under reduced pressure, and the residue was dissolved in EtOAc and washed with 2 M aqueous sodium hydroxide solution (3 × 50 mL) and with brine, dried over Na_2SO_4, filtered, and the solvent was removed under reduced pressure. The crude product was purified by column chromatography.

(*S*)-Methyl-1-benzylpyrroldine-2-carboxylate

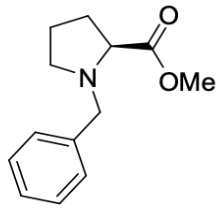

Esterification: To a solution of L-proline (5.00 g, 43.4 mmol, 1.00 equiv.) in methanol (40 mL, 1 M) at 0 °C was added dropwise thionyl chloride (7.87 mL, 109 mmol, 2.50 equiv.) and stirred for 20 h at room temperature. Methanol was removed under reduced pressure and toluene (50 mL) was added and removed under reduced pressure three times. The crude material was used without further purification for the next step

Benzylation: The crude material was dissolved in dichloromethane (40 mL, 1 M) and DiPEA (14.0 mL, 109 mmol, 2.50 equiv.) was added. The formed precipitate was filtered and washed with small portions of CH_2Cl_2. The solvent was removed under reduced pressure and the residue was suspended in Et_2O (50 mL), cooled to 0 °C and benzyl bromide (5.67 mL, 47.8 mmol, 1.10 equiv.) was slowly added dropwise. After stirring for 24 h at room temperature the reaction mixture was filtered, washed with small portions of Et_2O, and the solvent was removed under reduced pressure. After column chromatography (silica, Hex/EtOAc = 3/1), (*S*)-methyl-1-benzylpyrrolidin-2-carboxylate (8.1 g, 36.9 mmol, 95%) was obtained as an orange oil.

TLC: *R*f = 0.35 (Hex/EtOAc = 3/1) [CAM].

^{1}H NMR (400 MHz, $CDCl_3$): δ/ppm = 7.28 – 7.11 (m, 5H), 3.80 (d, *J* = 12.8 Hz, 1H), 3.55 (s, 3H), 3.49 (d, *J* = 12.8, 1.4 Hz, 1H), 3.17 (dd, *J* = 8.9, 6.2 Hz, 1H), 2.96 (td, *J* = 8.6, 2.6 Hz, 1H), 2.35 – 2.27 (m, 1H), 2.11 – 1.98 (m, 1H), 1.94 – 1.74 (m, 2H), 1.74 – 1.63 (m, 1H).

^{13}C NMR (101 MHz, $CDCl_3$): δ/ppm = 174.5 (C), 138.3 (C), 129.2 (CH), 128.2 (CH), 127.1 (CH), 65.3 (CH), 58.7 (CH_2), 53.3 (CH_2), 51.7 (CH_3), 29.4 (CH_2), 23.9 (CH_2).

The NMR spectra are in accordance with those reported in the literature.[13]

(*S*)-(1-Benzylpyrrolidin-2-yl)diphenylmethanol

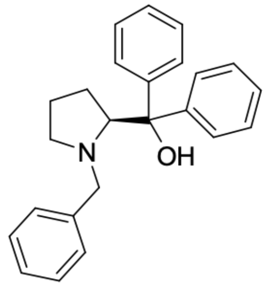

Using GP1, (*S*)-Methyl-1-benzylpyrrolidin-2-carboxylate (0.750 g, 3.42 mmol, 1.00 equiv.) was converted with bromobenzene (0.895 mL, 1.34 g, 8.55 mmol, 2.50 equiv.) and magnesium (0.208 g, 8.55 mmol, 2.50 equiv.) in 20 h at room temperature. After column chromatography (silica, Hex/EtOAc = 1/0 → 9/1), (*S*)-(1-benzylpyrrolidin- 2-yl)bis(3,5-dimethylphenyl)methanol (1.09 g, 3.18 mmol, 93%) was obtained as an orange foam and directly used in the next step.

TLC: *R*f = 0.45 (Hex/EtOAc = 1/1) [UV, CAM].

(*S*)-Diphenyl)(pyrrolidin-2-yl)methanol

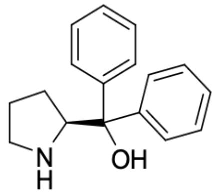

Using GP2, (*S*)-(1-Benzylpyrrolidin-2-yl)diphenylmethanol (1.09 g, 3.18 mmol, 1.00 equiv.) was converted with palladium on charcoal (0.674 g, 10 wt%, 0.20 equiv.) under hydrogen atmosphere in 24 h. After column chromatography (silica, Hex/EtOAc = 1/0 → 9/1) (*S*)-Diphenyl)(pyrrolidin-2-yl)methanol (0.699 g, 2.76 mmol, 87%) was obtained as a pale yellow solid.

TLC: *R*f = 0.11 (Hex/EtOAc = 1/1) [UV, CAM].

^{1}H NMR (400 MHz, $CDCl_3$): δ/ppm = 7.61 – 7.55 (m, 2H), 7.51 – 7.46 (m, 2H), 7.33 – 7.24 (m, 5H), 7.20 – 7.13 (m, 2H), 4.29 (t, *J* = 7.6 Hz, 1H), 3.05 – 2.89 (m, 2H), 1.80 – 1.51 (m, 4H).

^{13}C NMR (101 MHz, $CDCl_3$): δ/ppm = 148.0 (C), 145.4 (C), 128.4 (CH), 128.1 (CH), 126.7 (CH), 126.5 (CH), 126.0 (CH), 125.6 (CH), 77.3 (COH), 64.7 (CH), 46.9 (CH_2), 26.5 (CH_2), 25.6 (CH_2).

HRMS (ESI): calcd for $C_{17}H_{20}NO$ $[M+H]^+$: 254.1539; found: 254.1540

The NMR spectra are in accordance with those reported in the literature.[14]

(*S*)-(1-Benzylpyrrolidin-2-yl)bis(3,5-dimethylphenyl)methanol

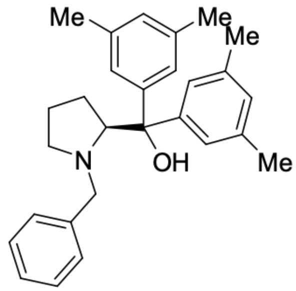

Using GP1, (*S*)-Methyl-1-benzylpyrrolidin-2-carboxylate (2.66 g, 12.1 mmol, 1.00 equiv.) was converted with 1-Bromo-3,5-dimethylbenzene (4.19 mL, 5.61 g, 30.33 mmol, 2.50 equiv.) and magnesium (0.737 g, 30.33 mmol, 2.50 equiv.) in 20 h at room temperature. After column chromatography (silica, Hex/EtOAc = 1/0 → 10/1), (*S*)-(1-benzylpyrrolidin- 2-yl)bis(3,5-dimethylphenyl)methanol (3.23 g, 8.13 mmol, 67%) was obtained as an orange oil.

TLC: *R*f = 0.30 (Hex/EtOAc = 10/1) [UV, CAM].

^{1}H NMR (400 MHz, $CDCl_3$): δ/ppm = 7.31 – 7.28 (m, 2H), 7.25 – 7.16 (m, 5H), 7.09 – 7.02 (m, 2H), 6.80 (s, 1H), 6.71 (s, 1H), 4.77 (s, 1H), 3.89 (dd, *J* = 9.5, 4.5 Hz, 1H), 3.18 – 2.97 (m, 2H), 2.95 – 2.88 (m, 1H), 2.30 (s, 6H), 2.25 (s, 6H), 2.17 (s, 2H), 2.04 – 1.87 (m, 1H), 1.83 – 1.71 (m, 1H).

^{13}C NMR (101 MHz, $CDCl_3$): δ/ppm = 148.0 (C), 146.6 (C), 140.1 (C), 137.5 (C), 137.4 (C), 128.8 (CH), 128.2 (CH), 128.2 (CH), 128.0 (CH), 126.9 (CH), 123.7 (CH), 123.6 (CH), 78.2 (COH), 70.9 (CH), 60.8 (CH_2), 55.8 (CH_2), 30.0 (CH_2), 24.5 (CH_2), 21.8 (CH_3), 21.7 (CH_3).

HRMS (ESI): calcd for $C_{28}H_{34}NO$ $[M+H]^+$: 400.2635; found: 400.2638.

The NMR spectra are in accordance with those reported in the literature.[13]

(*S*)-Bis(3,5-dimethylphenyl)(pyrrolidin-2-yl)methanol

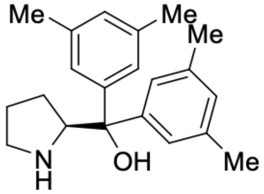

Using GP2, (*S*)-(1-Benzylpyrrolidin-2-yl)diphenylmethanol (3.23 g, 8.13 mmol, 1.00 equiv.) was converted with palladium on charcoal (1.72 g, 10 wt%, 0.20 equiv.) under hydrogen atmosphere in 24 h. After column chromatography (silica, Hex/EtOAc = 1/0 → 10/1) (*S*)-Diphenyl)(pyrrolidin-2-yl)methanol (2.31 g, 7.46 mmol, 92%) was obtained as a pale yellow solid.

TLC: *R*f = 0.21 (Hex/EtOAc = 10/1) [UV, CAM].

^{1}H NMR (400 MHz, $CDCl_3$): δ/ppm = 7.18 (s, 2H), 7.10 (s, 2H), 6.80 (s, 2H), 4.23 (t, *J* = 7.7 Hz, 1H), 3.05 – 2.97 (m, 1H), 2.97 – 2.89 (m, 1H), 2.28 (d, *J* = 5.1 Hz, 12H), 1.79 – 1.67 (m, 2H), 1.67 – 1.53 (m, 2H).

^{13}C NMR (101 MHz, $CDCl_3$): δ/ppm = 148.2 (C), 145.4 (C), 137.7 (C), 137.4 (C), 128.3 (CH), 128.2 (CH), 123.8 (CH), 123.4 (CH), 77.4 (COH), 64.7 (CH), 46.87 (CH_2), 26.4 (CH_2), 25.6 (CH_2), 21.7 (CH_3).

HRMS (ESI): calcd for $C_{21}H_{28}NO$ $[M+H]^+$: 310.2165; found: 310.2168.

The NMR spectra are in accordance with those reported in the literature.[13]

(*S*)-(1-Benzylpyrrolidin-2-yl)bis(3,5-diisopropylphenyl)methanol

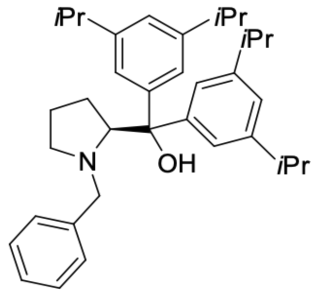

Using GP1, (*S*)-Methyl-1-benzylpyrrolidin-2-carboxylate (0.848 g, 3,87 mmol, 1.00 equiv.) was converted with 1-Bromo-3,5-diisopropylbenzene (2.33 g, 9.68 mmol, 2.50 equiv.) and magnesium (0.235 g, 9.68 mmol, 2.50 equiv.) in 20 h at room temperature. After column chromatography (silica, Hex/EtOAc = 10/1), (*S*)-(1-Benzylpyrrolidin-2-yl)bis(3,5-diisopropyl-phenyl)methanol (0.603 g, 1.18 mmol, 31%) was obtained as a colorless foam.

TLC: *R*f = 0.20 (Hex/EtOAc = 10/1) [UV, CAM].

^{1}H NMR (600 MHz, $CDCl_3$): δ/ppm 7.49 (d, *J* = 1.6 Hz, 2H), 7.31 (d, *J* = 1.7 Hz, 2H), 7.25 – 7.17 (m, 3H), 7.05 – 7.01 (m, 2H), 6.90 – 6.79 (m, 2H), 4.88 (s, 1H), 4.00 (dd, *J* = 9.4, 4.8 Hz, 1H), 3.13 (dd, *J* = 125.9, 12.6 Hz, 2H), 2.96 – 2.82 (m, 5H), 2.38 (td, *J* = 9.5, 6.6 Hz, 1H), 1.97 – 1.88 (m, 1H), 1.78 – 1.53 (m, 3H), 1.26 (d, *J* = 6.9, 1.1 Hz, 12H), 1.21 (d, *J* = 7.0 Hz, 12H).

^{13}C NMR (151 MHz, $CDCl_3$): δ/ppm = 148.4 (C), 148.2 (C), 148.2 (C), 146.7, 140.3, 128.8 (CH), 128.1 (CH), 126.8 (C), 122.8 (C), 122.3 (C), 121.5 (CH), 121.2 (CH), 78.3 (COH), 71.6 (CH), 60.6 (CH_2), 55.8 (CH_2), 34.5 (CH), 34.4 (CH), 29.8 (CH_2), 24.4 (CH_3), 24.3 (CH_3), 24.2 (CH_3), 24.1 (CH_3).

HRMS (ESI): calcd for $C_{36}H_{50}NO$ $[M+H]^+$: 512.3887; found: 512.3884.

(*S*)-Bis(3,5-diisopropylphenyl)(pyrrolidin-2-yl)methanol

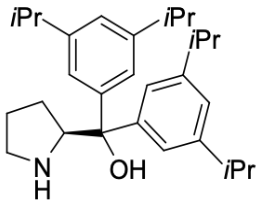

Using GP2, (*S*)-(1-Benzylpyrrolidin-2-yl)bis(3,5-diisopropylphenyl)methanol (0.603 g, 1.18 mmol, 1.00 equiv.) was converted with palladium on charcoal (0.232 g, 10 wt%, 0.20 equiv.) under hydrogen atmosphere in 24 h. After column chromatography (silica, Hex/EtOAc = 3/1) (S)-Bis(3,5-diisopropylphenyl)(pyrrolidin-2-yl)methanol (0.294 g, 0.697 mmol, 59%) was obtained as a colorless solid.

TLC: *R*f = 0.15 (Hex/EtOAc = 3/1) [UV, CAM].

^{1}H NMR (400 MHz, $CDCl_3$): δ/ppm = 7.28 (d, *J* = 1.6 Hz, 2H), 7.20 (d, *J* = 1.7 Hz, 2H), 6.87 (dt, *J* = 6.2, 1.8 Hz, 2H), 4.24 (t, *J* = 7.7 Hz, 1H), 3.05 – 2.91 (m, 2H), 2.86 (dq, *J* = 13.3, 6.8 Hz, 4H), 1.79 – 1.52 (m, 5H), 1.25 – 1.17 (m, 24H).

^{13}C NMR (101 MHz, $CDCl_3$): δ/ppm = 148.5 (C), 148.1 (C), 145.4 (C), 122.5 (CH), 122.5 (CH), 121.7 (CH), 121.3 (CH), 77.4 (COH), 65.4 (CH), 46.9 (CH_2), 34.4 (CH), 26.5 (CH_2), 25.6 (CH_2), 24.5 (CH_3), 24.3 (CH_3), 24.2 (CH_3), 24.1 (CH_3).

HRMS (ESI): calcd for $C_{29}H_{44}NO$ $[M+H]^+$: 421.3417; found: 421.3415.

(*S*)-(1-Benzylpyrrolidin-2-yl)bis(3,5-di-^{t}buylphenyl)methanol

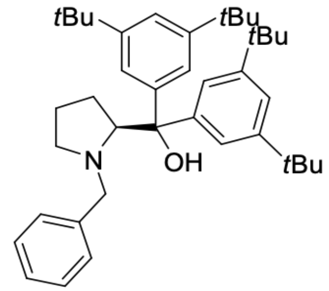

Using GP1, (*S*)-Methyl-1-benzylpyrrolidin-2-carboxylate (0.700 g, 3.19 mmol, 1.00 equiv.) was converted with 1-Bromo-3,5-di-^{t}butylbenzene (2.15 g, 7.98 mmol, 2.50 equiv.) and magnesium (0.194 g, 7.98 mmol, 2.50 equiv.) in 20 h at room temperature. After column chromatography (silica, Hex/EtOAc = 1/0 → 20/1), (*S*)-(1-Benzylpyrrolidin-2-yl)bis(3,5-di-^{t}buylphenyl)methanol (1.06 g, 1.88 mmol, 59%) was obtained as a colorless foam.

TLC: *R*f = 0.20 (Hex/EtOAc = 20/1) [UV, CAM].

^{1}H NMR (400 MHz, $CDCl_3$): δ/ppm = 7.61 (d, *J* = 1.8 Hz, 2H), 7.42 (d, *J* = 1.8 Hz, 2H), 7.17 – 7.03 (m, 5H), 6.96 – 6.86 (m, 2H), 5.12 (s, 2H), 3.94 (dd, *J* = 9.3, 4.7 Hz, 1H), 3.12 (d, *J* = 12.6 Hz, 1H), 2.92 (d, *J* = 12.7 Hz, 1H), 2.88 – 2.80 (m, 1H), 2.28 (td, *J* = 9.2, 6.9 Hz, 1H), 1.88 – 1.72 (m, 1H), 1.69 – 1.47 (m, 3H), 1.23 (d, *J* = 18.1 Hz, 36H).

^{13}C NMR (101 MHz, $CDCl_3$): δ/ppm = 150.1 (C), 149.9 (C), 147.6 (C), 146.1 (C), 140.3 (C), 128.7 (CH), 128.1 (CH), 126.8 (CH), 120.3 (CH), 119.9 (CH), 119.8 (CH), 78.4 (COH), 71.9 (CH), 60.5 (CH_2), 55.8 (CH_2), 53.5 (CH_2), 35.1 (C), 35.0 (C), 31.8 (CH_3), 31.7 (CH_3), 29.8 (CH_3), 24.3 (CH_2).

HRMS (ESI): calcd for $C_{40}H_{57}NONa$ $[M+Na]^+$: 590.4332; found: 590.4335.

(*S*)-Bis(3,5-di-^{t}butylphenyl)(pyrrolidin-2-yl)methanol

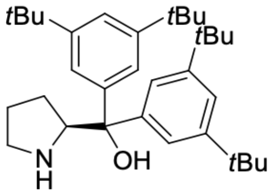

Using GP2, (*S*)-(1-Benzylpyrrolidin-2-yl)bis(3,5-di^{t}buylphenyl)methanol (1.06 g, 1.88 mmol, 1.00 equiv.) was converted with palladium on charcoal (0.397 g, 10 wt%, 0.20 equiv.) under hydrogen atmosphere in 24 h. After column chromatography (silica, Hex/EtOAc 3/1) (*S*)-Bis(3,5-di-^{t}butylphenyl)(pyrrolidin-2-yl)methanol (0.794 g, 1.66 mmol, 89%) was obtained as a colorless solid.

TLC: *R*f = 0.18 (Hex/EtOAc = 3/1) [UV, CAM].

^{1}H NMR (400 MHz, CD_2Cl_2): δ/ppm = 7.50 (d, *J* = 1.8 Hz, 2H), 7.39 (d, *J* = 1.8 Hz, 2H), 7.26 (t, *J* = 1.8 Hz, 1H), 7.22 (t, *J* = 1.8 Hz, 1H), 4.44 (t, *J* = 7.8 Hz, 1H), 2.85 (s, 2H), 1.78 – 1.67 (m, 2H), 1.65 – 1.54 (m, 2H), 1.30 (d, *J* = 9.2 Hz, 36H).

^{13}C NMR (101 MHz, CD_2Cl_2): δ/ppm = 151.1 (C), 150.7 (C), 145.3 (C), 121.3 (CH), 120.7 (CH), 120.2 (CH), 120.0 (CH), 78.2 (COH), 65.9 (CH), 47.3 (CH_2), 35.4 (C), 31.9 (CH_3), 31.8 (CH_3), 27.0 (CH_2), 26.0 (CH_2).

HRMS (ESI): calcd for $C_{33}H_{53}NO$ $[M+H]^+$: 478.4044; found: 478.4044.

The NMR spectra are in accordance with those reported in the literature.[15]

(*S*)-(1-Benzylpyrrolidin-2-yl)bis(3,5-dimethyl-4-Methoxy-phenyl)methanol

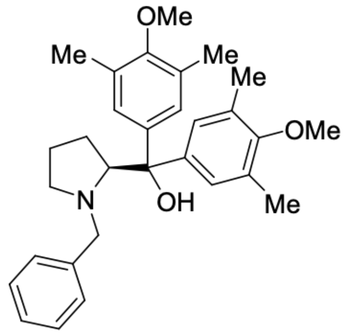

Using GP1, (*S*)-Methyl-1-benzylpyrrolidin-2-carboxylate (2.00 g, 9.66 mmol, 1.00 equiv.) was converted with 1-Bromo-3,5-dimethyl-4-methoxybenzene (6.50 g, 24.15 mmol, 2.50 equiv.) and magnesium (0.587 g, 24.15 mmol, 2.50 equiv.) in 20 h at room temperature. After column chromatography (silica, Hex/EtOAc = 10/1), (*S*)-(1-Benzylpyrrolidin-2-yl)bis(3,5-dimethyl-4-Methoxy-phenyl)methanol (1.50 g, 3.26 mmol, 34%) was obtained as a colorless oil.

TLC: *R*f = 0.16 (Hex/EtOAc = 10/1) [UV, CAM].

^{1}H NMR (400 MHz, $CDCl_3$): δ/ppm = 7.31 – 7.15 (m, 7H), 7.05 – 7.00 (m, 2H), 4.71 (s, 1H), 3.83 (dd, *J* = 9.5, 4.4 Hz, 1H), 3.68 (s, 3H), 3.58 (s, 3H), 3.08 (dd, 2H), 2.96 – 2.90 (m, 1H), 2.35 (td, *J* = 9.3, 7.3 Hz, 1H), 2.27 (s, 6H), 2.21 (s, 6H), 2.01 – 1.87 (m, 1H), 1.81 – 1.69 (m, 1H), 1.67 – 1.54 (m, 2H).

^{13}C NMR (101 MHz, $CDCl_3$): δ/ppm = 155.5 (C), 155.4 (C), 143.2 (C), 141.9 (C), 140.1 (C), 130.2 (CH), 130.2 (CH), 128.7 (CH), 128.2 (CH), 126.9 (CH), 126.3 (CH), 126.2 (CH), 77.7 (COH), 71.1 (CH), 60.8 (CH_2), 59.8 (CH_3), 59.7 (CH_3), 55.9 (CH_2), 30.0 (CH_2), 24.5 (CH_2), 16.6 (CH_3), 16.5 (CH_3).

HRMS (ESI): calcd for $C_{30}H_{38}NO_3$ $[M+H]^+$: 460.2846; found: 460.2848.

(*S*)-Bis(3,5-dimethyl-4-Methoxy-phenyl)(pyrrolidin-2-yl)methanol

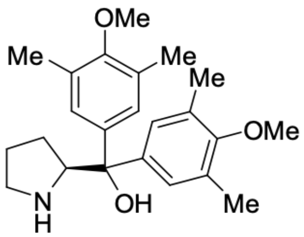

Using GP2, (*S*)-(1-Benzylpyrrolidin-2-yl)bis(3,5-dimethyl-4-Methoxy-phenyl)methanol (1.37 g, 2.98 mmol, 1.00 equiv.) was converted with palladium on charcoal (0.632 g, 10 wt%, 0.20 equiv.) under hydrogen atmosphere in 24 h. After column chromatography (silica, Hex/EtOAc = 1/1 → 0/1) (*S*)-Bis(3,5-dimethyl-4-Methoxy-phenyl)(pyrrolidin-2-yl)methanol (0.717 g, 1.94 mmol, 65%) was obtained as a light orange solid.

TLC: *R*f = 0.11 (Hex/EtOAc = 1/1) [UV, CAM].

^{1}H NMR (400 MHz, CD_2Cl_2): δ/ppm = 7.19 (s, 2H), 7.11 (s, 2H), 4.23 (t, *J* = 7.7 Hz, 1H), 3.66 (d, *J* = 1.5 Hz, 6H), 3.02 – 2.88 (m, 2H), 2.24 (d, *J* = 5.5 Hz, 12H), 1.71 (p, *J* = 7.1 Hz, 2H), 1.60 – 1.51 (m, 2H).

^{13}C NMR (101 MHz, CD_2Cl_2): δ/ppm = 156.0 (C), 155.9 (C), 144.1 (C), 141.6 (C), 130.9 (C), 130.6 (C), 126.6 (CH), 126.2 (CH), 76.9 (COH), 64.9 (CH), 60.0 (CH_3), 47.3 (CH_2), 26.7 (CH_2), 26.1 (CH_2), 16.7 (CH_3), 16.6 (CH_3).

HRMS (ESI): calcd for $C_{23}H_{32}NO_3$ $[M+H]^+$: 370.2378; found: 370.2379.

The NMR spectra are in accordance with those reported in the literature.[15]

(*S*)-Bis(3,5-di-^{t}butyl-4-Methoxy-phenyl)(pyrrolidin-2-yl)methanol

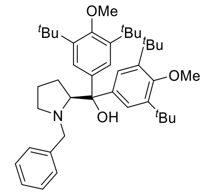

Using GP1, (*S*)-Methyl-1-benzylpyrrolidin-2-carboxylate (0.750 g, 3.42 mmol, 1.00 equiv.) was converted with 1-Bromo-3,5-di-^{t}butyl-4-methoxybenzene (2.56 g, 8.55 mmol, 2.50 equiv.) and magnesium (0.208 g, 8.55 mmol, 2.50 equiv.) in 20 h at room temperature. After column chromatography (silica, CH_2Cl_2), (*S*)-Bis(3,5-di-^{t}butyl-4-Methoxy-phenyl)(pyrrolidin-2-yl)methanol (1.65 g, 2.63 mmol, 77%) was obtained as an orange oil, which was directly used in the next step.

TLC: *R*f = 0.3 (CH_2Cl_2 = 1/1) [UV, CAM].

(*S*)-Bis(3,5-di-*t*butyl-4-Methoxy-phenyl)(pyrrolidin-2-yl)methanol

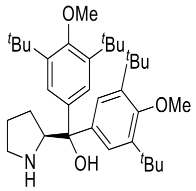

Using GP2, (*S*)-(1-Benzylpyrrolidin-2-yl)bis(3,5-di-*t*butyl-4-Methoxy-phenyl)methanol (0.661 g, 1.05 mmol, 1.00 equiv.) was converted with palladium on charcoal (0.223 g, 10 wt%, 0.20 equiv.) under hydrogen atmosphere in 24 h. After column chromatography (silica, CH_2Cl_2/MeOH = 40/1) (*S*)-Bis(3,5-di-*t*butyl-4-methoxy-phenyl)(pyrrolidin-2-yl)methanol (0.298 g, 0.554 mmol, 53%) was obtained as a colorless solid.

TLC: *R*f = 0.10 (CH_2Cl_2/MeOH = 40/1) [UV, CAM].

^{1}H NMR (400 MHz, $CDCl_3$): δ/ppm = 7.37 (s, 2H), 7.24 (s, 2H), 4.23 – 4.11 (m, 1H), 3.56 (d, *J* = 8.1 Hz, 6H), 2.83 – 2.71 (m, 2H), 1.69 – 1.58 (m, 3H), 1.53 – 1.42 (m, 1H), 1.34 (s, 18H), 1.31 (s, 18H), 1.21 – 1.14 (m, 1H).

^{13}C NMR (101 MHz, $CDCl_3$): δ/ppm = 158.2 (C), 157.8 (C), 143.0 (C), 142.6 (C), 139.0 (C), 124.3 (CH), 124.0 (CH), 77.4 (COH), 65.7 (CH), 64.2 (CH_3), 46.8 (CH_2), 36.0 (CH_3), 32.3 (CH_3), 26.7 (CH_2), 25.4 (CH_2).

HRMS (ESI): calcd for $C_{35}H_{56}NO_3$ $[M+H]^+$: 538.4255; found: 538.4252.

The NMR spectra are in accordance with those reported in the literature.[16]

(*S*)-(1-Benzylpyrrolidin-2-yl)dinaphthylmethanol

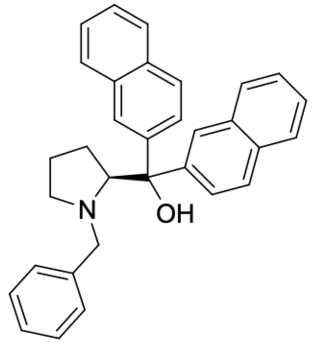

Using GP1, (*S*)-Methyl-1-benzylpyrrolidin-2-carboxylate (2.12 g, 9.66 mmol, 1.00 equiv.) was converted with 2-bromonaphthalene (5.00 g, 24.15 mmol, 2.50 equiv.) and magnesium (0.587 g, 24.15 mmol, 2.50 equiv.) in 20 h at room temperature. After column chromatography (silica, Hex/EtOAc = 80/1 → 10/1), (*S*)-(1-Benzylpyrrolidin-2-yl)dinaphthyl-methanol (1.85 g, 4.78 mmol, 49%) was obtained as a colorless solid.

TLC: *R*f = 0.19 (Hex/EtOAc = 40/1) [UV, CAM].

^{1}H NMR (400 MHz, $CDCl_3$): δ/ppm = 8.34 (s, 1H), 8.11 (s, 1H), 7.89 – 7.66 (m, 8H), 7.52 – 7.29 (m, 4H), 7.19 (s, 3H), 7.03 – 6.95 (m, 2H), 5.25 (s, 1H), 4.24 (dd, *J* = 9.4, 4.8 Hz, 1H), 3.18 (dd, *J* = 89.1, 12.6 Hz, 2H), 2.97 (d, *J* = 18.5 Hz, 1H), 2.48 – 2.37 (m, 1H), 2.02 (s, 1H), 1.90 (s, 1H), 1.64 (s, 2H).

^{13}C NMR (101 MHz, $CDCl_3$): δ/ppm = 145.4 (C), 144.0 (C), 139.6 (C), 133.4 (C), 133.4 (C), 132.2 (C), 128.7 (CH), 128.4 (CH), 128.3 (CH), 128.2 (CH), 128.0 (CH), 127.9 (CH), 127.6 (CH), 127.6 (CH), 127.0 (CH), 126.2 (CH), 126.0 (CH), 125.8 (CH), 125.8 (CH), 124.6 (CH), 124.5 (CH), 124.3 (CH), 124.1 (CH), 78.5 (COH), 70.2 (CH), 60.7 (CH_2), 55.7 (CH_2), 30.1 (CH_2), 24.3 (CH_2).

HRMS (ESI): calcd for $C_{32}H_{30}NO$ $[M+H]^+$: 444.2322; found: 444.2322.

(*S*)-Dinaphthalenyl-2-pyrrolidinemethanol

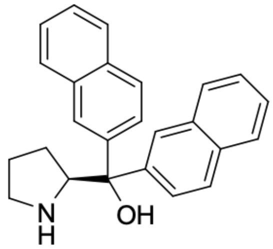

Using GP2, (*S*)-(1-Benzylpyrrolidin-2-yl)dinaphthylmethanol (1.85 g, 4.78 mmol, 1.00 equiv.) was converted with palladium on charcoal (0.883 g, 10 wt%, 0.20 equiv.) under hydrogen atmosphere in 24 h. After column chromatography (silica, Hex/EtOAc = 1/1) (*S*)-Bis(3,5-dimethyl-4-methoxy-phenyl)(pyrrolidin-2-yl)methanol (0.665 g, 1.88 mmol, 39%) was obtained as a colorless solid.

TLC: *R*f = 0.19 (Hex/EtOAc = 1/1) [UV, CAM].

^{1}H NMR (400 MHz, $CDCl_3$): δ/ppm = 8.16 (d, *J* = 1.8 Hz, 2H), 7.91 (td, *J* = 8.1, 1.5 Hz, 2H), 7.83 – 7.69 (m, 5H), 7.63 (dd, *J* = 8.6, 1.8 Hz, 1H), 7.55 – 7.41 (m, 4H), 4.53 (t, *J* = 7.1 Hz, 1H), 3.12 – 2.91 (m, 2H), 1.86 – 1.71 (m, 3H), 1.69 – 1.58 (m, 1H).

^{13}C NMR (101 MHz, $CDCl_3$): δ/ppm = 145.4 (C), 142.7 (C), 133.3 (C), 133.2 (C), 132.4 (C), 132.3 (C), 128.4 (CH), 128.3 (CH), 128.2 (CH), 127.8 (CH), 127.6 (CH), 126.1 (CH), 126.0 (CH), 125.9 (CH), 125.7 (CH), 125.4 (CH), 124.5 (CH), 124.1 (CH), 123.9 (CH), 77.6 (COH), 64.1 (CH), 46.9 (CH_2), 26.6 (CH_2), 25.7 (CH_2).

HRMS (ESI): calcd for $C_{25}H_{24}NO$ $[M+H]^+$: 354.1853; found: 354.1854.

The NMR spectra are in accordance with those reported in the literature.[14]

(*S*)-(1-Benzylpyrrolidin-2-yl)bis(3,5-bistrifluoromethylphenyl)methanol

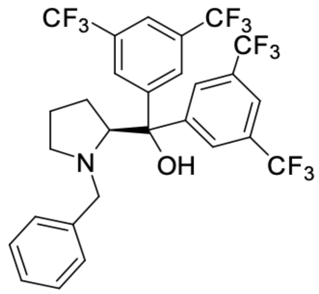

Using GP1, (*S*)-Methyl-1-benzylpyrrolidin-2-carboxylate (0.500 g, 2.28 mmol, 1.00 equiv.) was converted with 1-Bromo-3,5-bis(trifluoromethyl)benzene (1.67 g, 5.70 mmol, 2.50 equiv.) and magnesium (0.139 g, 5.70 mmol, 2.50 equiv.) in 20 h at room temperature. After column chromatography (silica, Hex/EtOAc = 80/1 → 10/1), (*S*)-(1-Benzylpyrrolidin-2-yl)dinaphthyl-methanol (0.688 g, 1.12 mmol, 49%) was obtained as a colorless solid, which was directly used in the next step.

TLC: *R*f = 0.25 (Hex/EtOAc = 10/1) [UV, CAM]

(*S*)-Bis(3,5-bistrifluoromethylphenyl)(pyrrolidin-2-yl)methanol

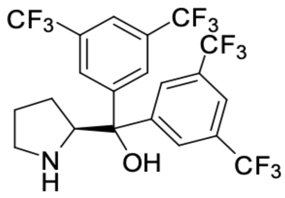

Using GP2, (*S*)-(1-Benzylpyrrolidin-2-yl)bis(3,5-bistrifluoromethylphenyl)methanol (0.688 g, 1.12 mmol, 1.00 equiv.) was converted with palladium on charcoal (0.237 g, 10 wt%, 0.20 equiv.) under hydrogen atmosphere in 24 h. After column chromatography (silica, Hex/EtOAc = 10/1) (*S*)-Bis(3,5-dimethyl-4-methoxy-phenyl)(pyrrolidin-2-yl)methanol (0.546 g, 1.04 mmol, 93%) was obtained as a colorless solid.

TLC: *R*f = 0.20 (Hex/EtOAc = 5/1) [UV, CAM].

^{1}H NMR (400 MHz, $CDCl_3$): δ/ppm = 8.05 (s, 2H), 7.96 (s, 2H), 7.76 (d, *J* = 4.4 Hz, 2H), 5.06 (dd, *J* = 8.7, 4.2 Hz, 0H), 4.35 (t, *J* = 7.7 Hz, 1H), 3.12 – 3.00 (m, 2H), 1.87 – 1.72 (m, 2H), 1.66 – 1.46 (m, 2H).

^{13}C NMR (101 MHz, $CDCl_3$): δ/ppm = 149.6 (C), 147.1 (C), 132.1 (qd, *J* = 33.4, 25.5 Hz, C), 127.4 (CH), 126.0 (dd, *J* = 37.7, 4.0 Hz, C), 124.7 (CH), 121.6 (dt, *J* = 19.3, 3.8 Hz), 119.3 (CH), 76.8 (COH), 65.0 (CH), 47.9 (CH_2), 27.9 (CH_2), 25.2 (CH_2).

HRMS (ESI): calcd for $C_{21}H_{16}F_{12}NO$ $[M+H]^+$: 526.1035; found: 526.1036.

The NMR spectra are in accordance with those reported in the literature.[17]

(*S*)-(1-Benzylpyrrolidin-2-yl)dipentafluorophenylmethanol

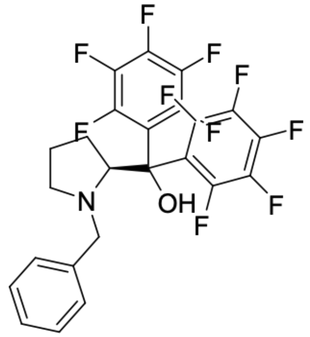

To a solution of pentafluoroiodobenzene (1.37 mL, 10.3 mmol, 3.0 equiv.) in 25 mL dry Et_2O under Ar at −78 °C, ^{n}BuLi in hexane [1.6 M] (6.00 mL, 9.6 mmol, 2.80 equiv.) was added dropwise. After stirring at −78 °C for 2 h (*S*)-Methyl-1-benzylpyrrolidin-2-carboxylate (0.670 mL, 3.43 mmol, 1.0 equiv.) were added and the solution was stirred at −78 °C for 3 h. Saturated NH_4Cl solution was added and the mixture was extracted with EtOAc (3 × 70 mL). The combined organic layers were washed with brine, dried over Na_2SO_4, filtered, and the solvent was removed under reduced pressure. After column chromatography (silica, Hex/EtOAc = 10/1) (*S*)-(1-Benzylpyrrolidin-2-yl)dipentafluoro-phenylmethanol was obtained as a colorless solid (1.02 g, 1.95 mmol, 57%).

TLC: *R*f = 0.49 (Hex/EtOAc = 10/1) [UV, CAM].

^{1}H NMR (400 MHz, $CDCl_3$): δ/ppm = 7.38 – 7.16 (m, 3H), 7.15 – 7.08 (m, 2H), 4.90 (s, 1H), 4.06 – 3.98 (m, 1H), 3.30 (dd, *J* = 88.6, 13.1 Hz, 2H), 3.05 (dt, *J* = 8.8, 3.5 Hz, 1H), 2.45 – 2.33 (m, 1H), 2.33 – 2.19 (m, 1H), 2.02 – 1.91 (m, 1H), 1.81 – 1.67 (m, 2H).

^{13}C NMR (101 MHz, $CDCl_3$): δ/ppm = 146.3 (d, *J* = 53.2 Hz, CF), 144.0 (d, *J* = 51.3 Hz, CF), 142.3 (m, CF), 139.2 (m, CF), 136.7 (m, CF), 128.7 (CH), 128.0 (CH), 127.5 (CH), 118.5 (m, C), 116.7 (m, C), 80.0 (COH), 70.2 (CH), 62.3 (CH_2), 56.5 (CH_2), 30.1 (CH_2), 25.4 (CH_2).

HRMS (ESI): calcd for $C_{24}H_{16}F_{10}NO$ $[M+H]^+$: 524.1065; found: 524.1064.

(*S*)-Dipentafluorophenyl(pyrrolidin-2-yl)methanol

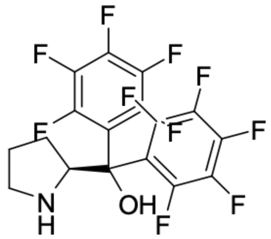

Using GP2, (*S*)-(1-Benzylpyrrolidin-2-yl)dipentafluorophenylmethanol (1.02 g, 1.95 mmol, 1.00 equiv.) was converted with palladium on charcoal (0.413 g, 10 wt%, 0.20 equiv.) under hydrogen atmosphere in 24 h. After column chromatography (silica, Hex/EtOAc = 40/1) and further crystallization from ^{n}heptane (*S*)-Dipentafluorophenyl(pyrrolidin-2-yl)methanol (0.507 g, 1.17 mmol, 60%) was obtained as a colorless solid.

TLC: *R*f = 0.22 (Hex/EtOAc = 40/1) [UV, CAM].

^{1}H NMR (400 MHz, $CDCl_3$): δ/ppm = 4.16 (t, *J* = 7.1 Hz, 1H), 3.62 – 3.50 (m, 1H), 3.26 – 3.15 (m, 1H), 2.93 (s, 1H), 2.29 – 1.92 (m, 4H).

^{13}C NMR (101 MHz, $CDCl_3$): δ/ppm = 146.0 (m, CF), 145.2 (m, CF), 144.3 (m, CF), 143.5 (m, CF), 141.8 (m, CF), 137.1 (m, CF), 136.4 (m, CF), 135.9 (m, CF), 134.8 (m, CF), 133.3 (m, CF), 117.9 (m, C), 117.0 (m, C), 80.1 (COH), 78.9 (CH), 52.2 (CH_2), 26.9 (CH_2), 24.7 (CH_2).

HRMS (ESI): calcd for $C_{17}H_{10}F_{10}NO$ $[M+H]^+$: 434.0597; found: 434.0599.

(*S*)-Prolinol

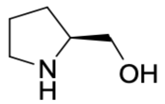

To a suspension of $LiAlH_4$ (0.850 g, 22.4 mmol, 1.6 equiv.) in dry THF was added (*S*)-Proline (1.61 g, 14.0 mmol, 1.0 equiv.) in small portions. After refluxing for 2 h, the reaction was stopped by careful addition of potassium hydroxide solution (0.4 g in 1.6 mL dest. H_2O). The mixture was then refluxed for 15 min and the hot solution was filtered. The precipitate was refluxed with THF for an additional hour and filtered once more. The combined filtrates were concentrated under reduced pressure and used without further purification in quantitative yield (1.41 g, 14.0 mmol, 99%).

^{1}H NMR (400 MHz, $CDCl_3$): δ/ppm = 3.61 – 3.50 (m, 1H), 3.39 – 3.28 (m, 2H), 3.09 (s, 2H), 3.01 – 2.85 (m, 2H), 1.90 – 1.63 (m, 3H), 1.50 – 1.37 (m, 1H).

^{13}C NMR (101 MHz, $CDCl_3$): δ/ppm = 64.6 (CH_2OH), 59.7 (CH), 46.4 (CH_2), 27.7 (CH_2), 26.1 (CH_2).

HRMS (ESI): calcd for $C_5H_{11}NO$ $[M+H]^+$: 102.0919; found: 102.0915.

The NMR spectra are in accordance with those reported in the literature.[18]

12. NMR Spectra

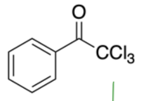

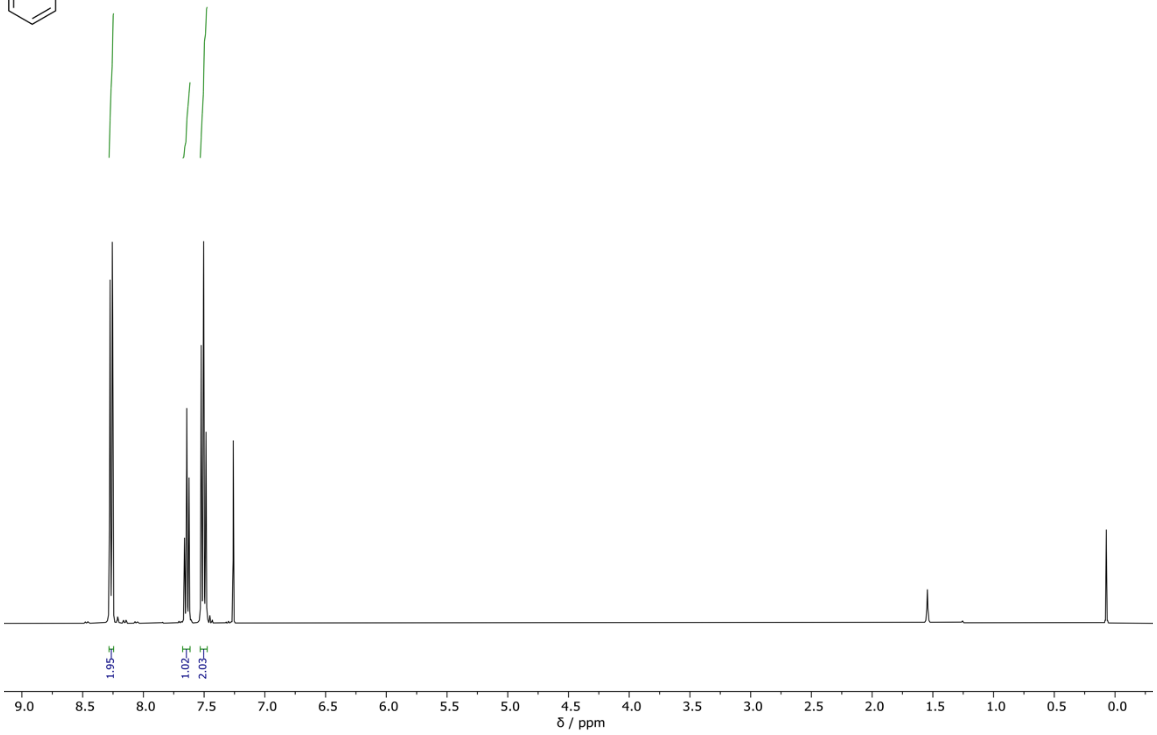

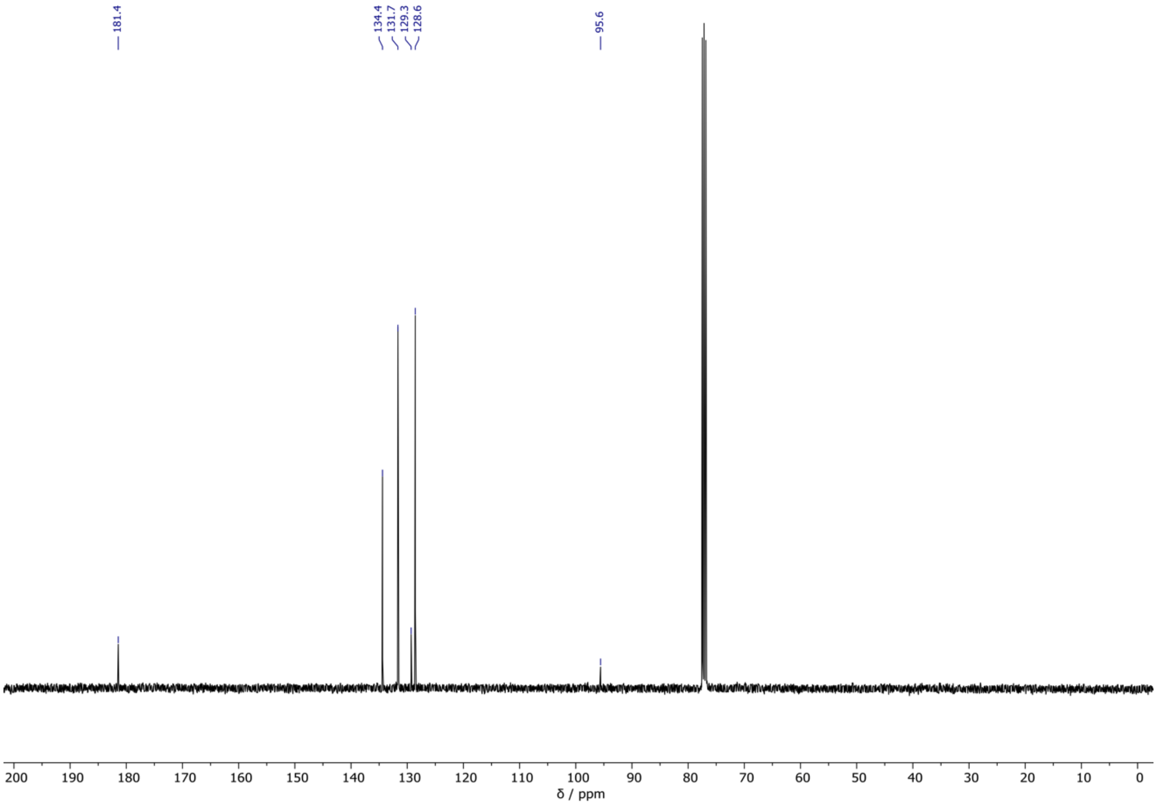

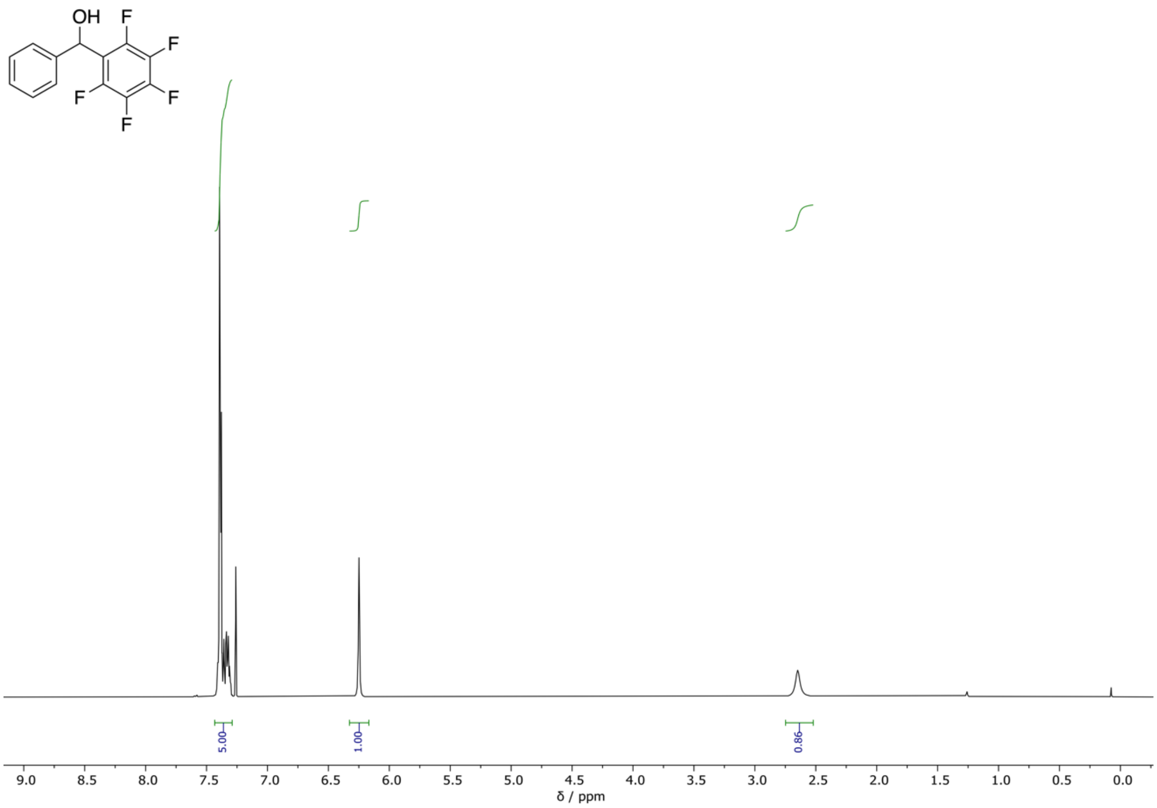
OH
F
F
F
F
F
5.00
1.00
0.86
δ / ppm

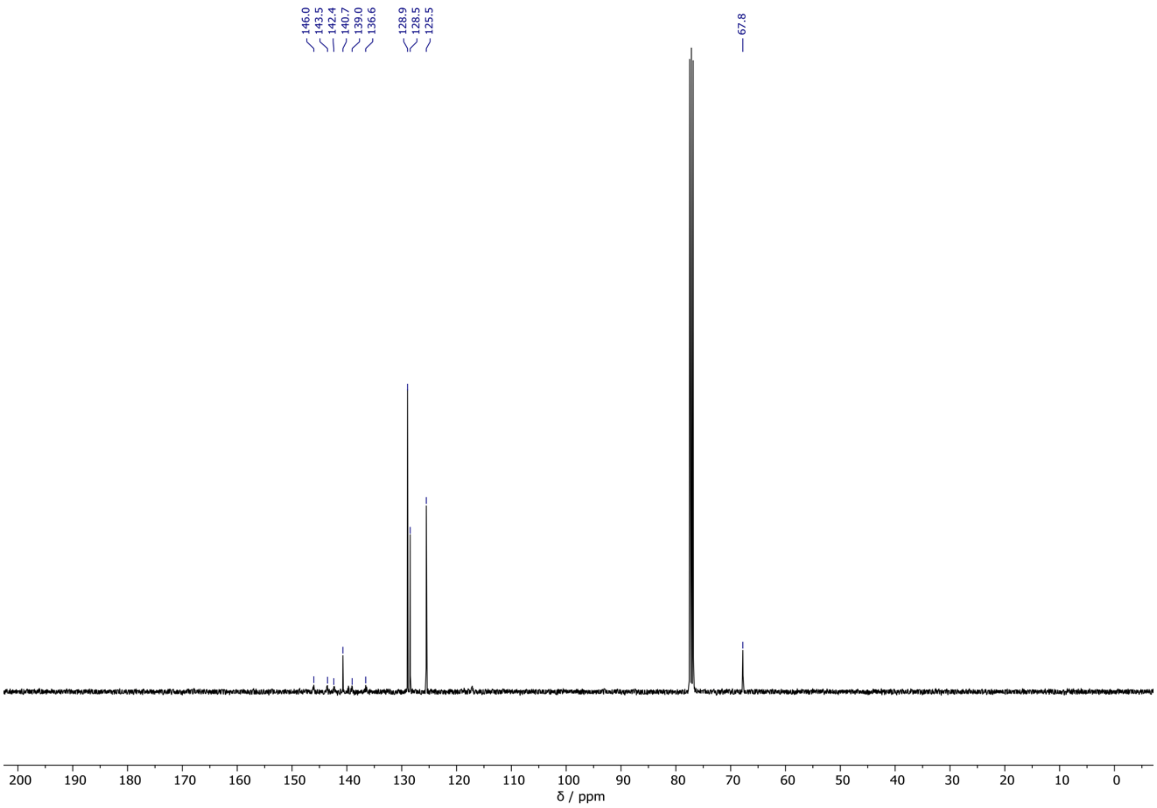
146.0
143.5
142.4
140.7
139.0
136.6
128.9
128.5
125.5
67.8
δ / ppm

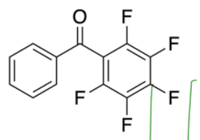

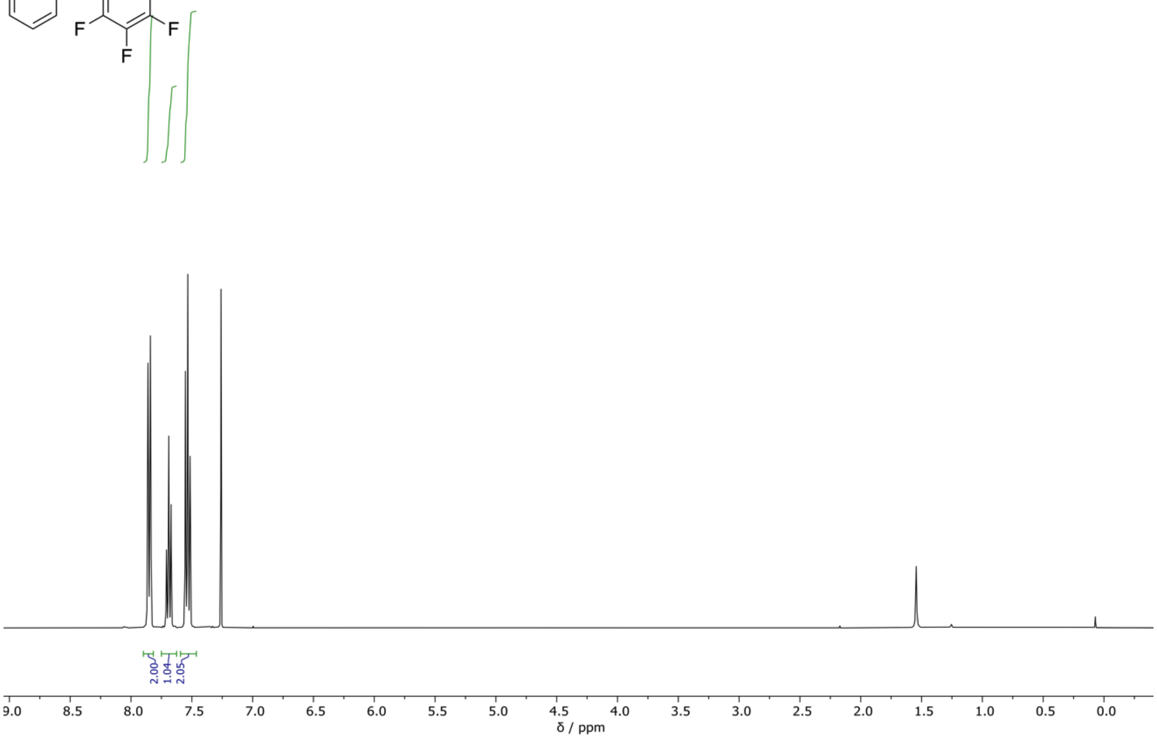
2.00
1.04
2.05
9.0
8.5
8.0
7.5
7.0
6.5
6.0
5.5
5.0
4.5
4.0
3.5
3.0
2.5
2.0
1.5
1.0
0.5
0.0
δ / ppm

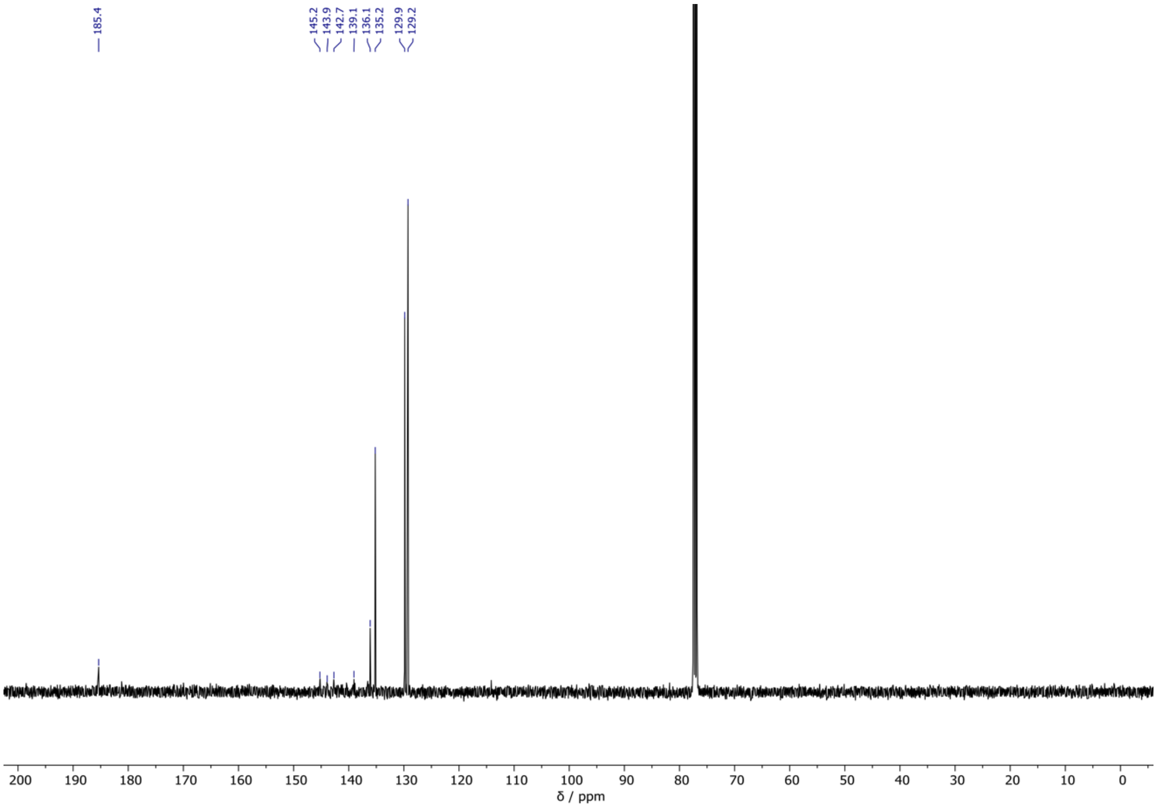
185.4
145.2
143.9
142.7
139.1
136.1
135.2
129.9
129.2
200
190
180
170
160
150
140
130
120
110
100
90
80
70
60
50
40
30
20
10
0
δ / ppm

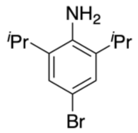
NH2
iPr
iPr
Br

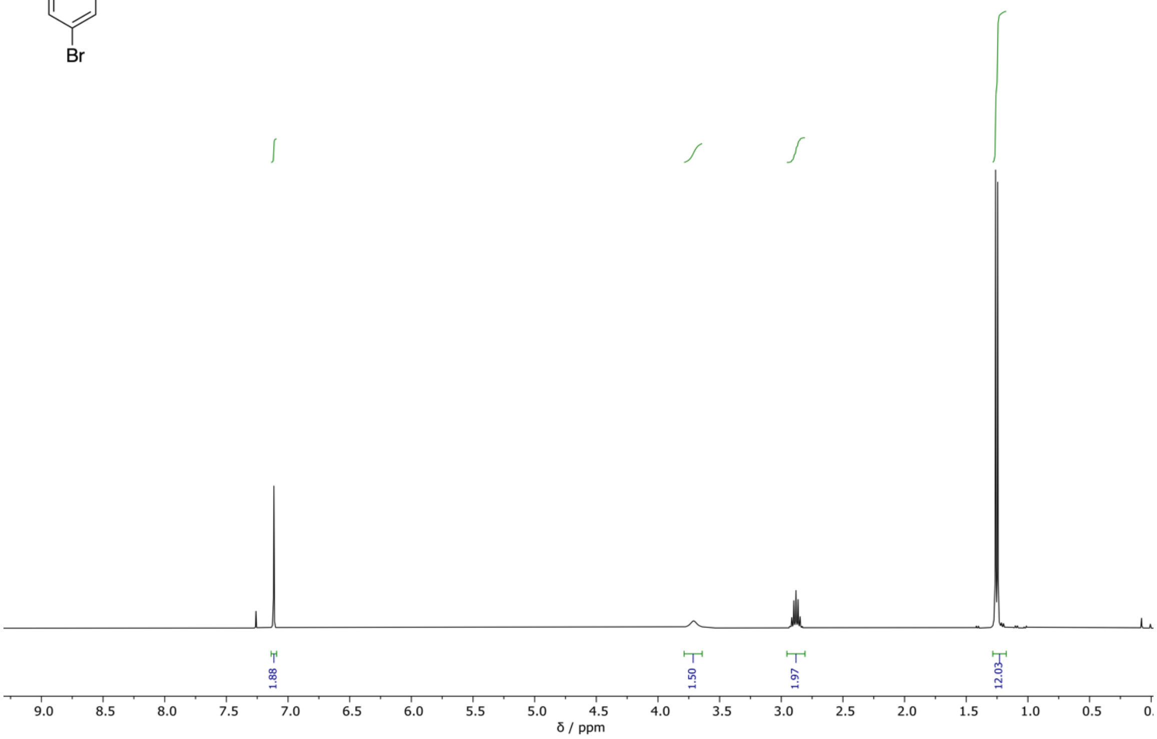
1.88
1.50
1.97
12.03
9.0 8.5 8.0 7.5 7.0 6.5 6.0 5.5 5.0 4.5 4.0 3.5 3.0 2.5 2.0 1.5 1.0 0.5
δ / ppm

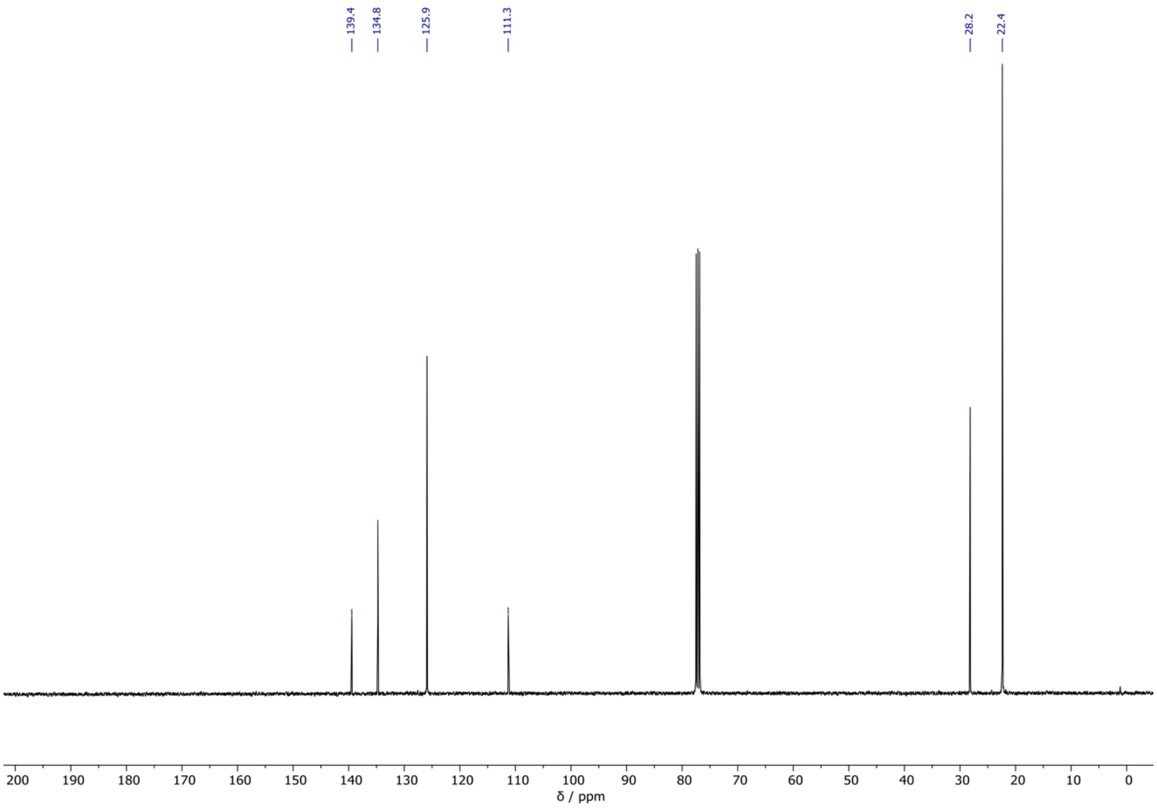
139.4
134.8
125.9
111.3
28.2
22.4
200 190 180 170 160 150 140 130 120 110 100 90 80 70 60 50 40 30 20 10 0
δ / ppm

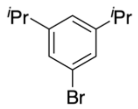
iPr
iPr
Br

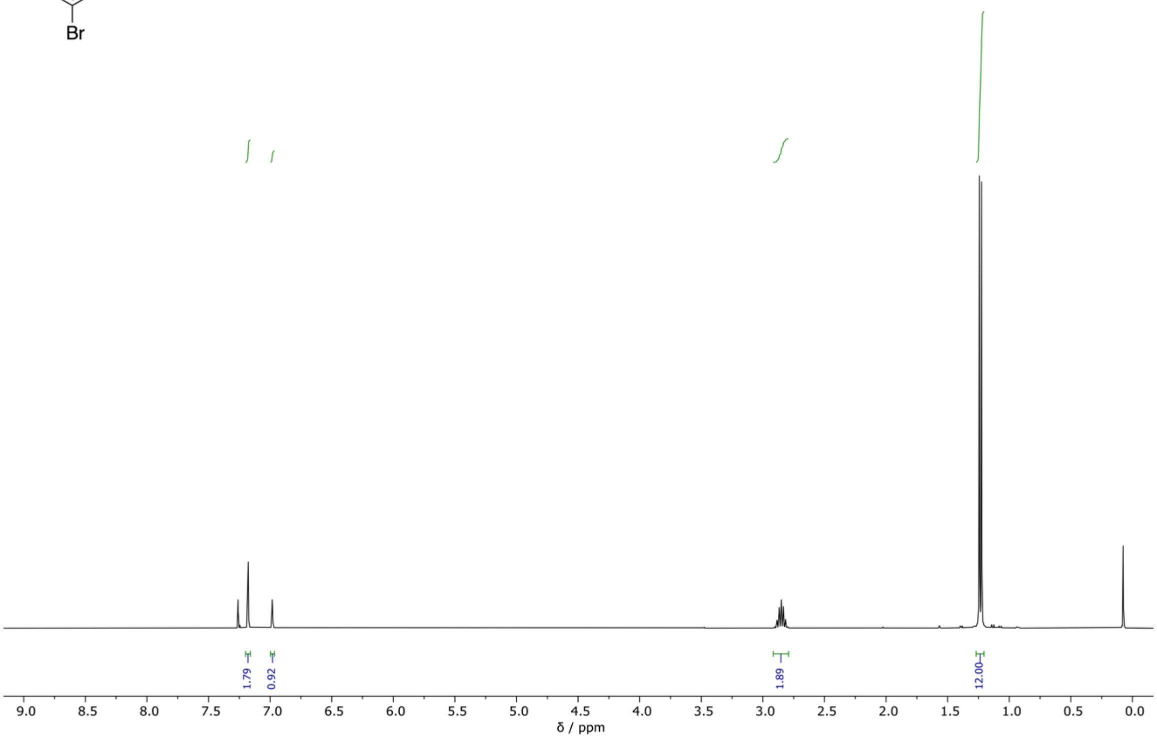
1.79
0.92
1.89
12.00
9.0
8.5
8.0
7.5
7.0
6.5
6.0
5.5
5.0
4.5
4.0
3.5
3.0
2.5
2.0
1.5
1.0
0.5
0.0
δ / ppm

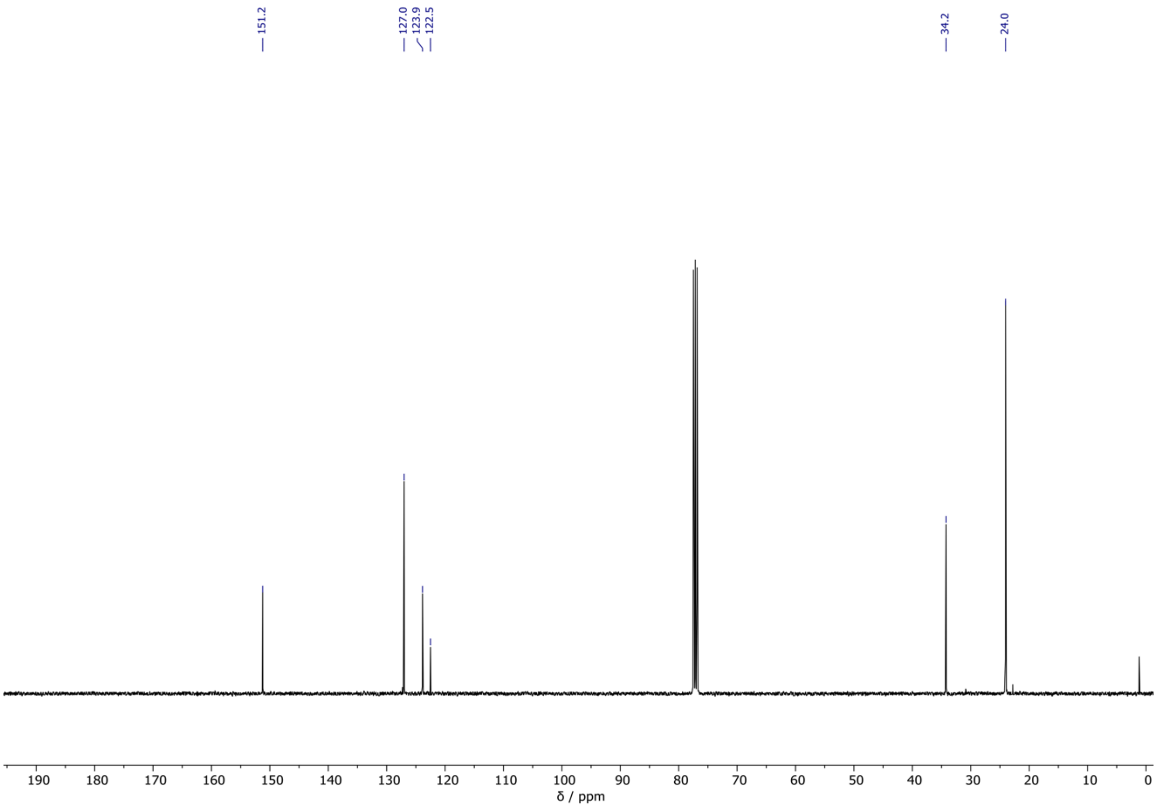
151.2
127.0
123.9
122.5
34.2
24.0
190
180
170
160
150
140
130
120
110
100
90
80
70
60
50
40
30
20
10
0
δ / ppm

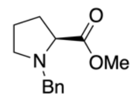
O
OMe
N
Bn

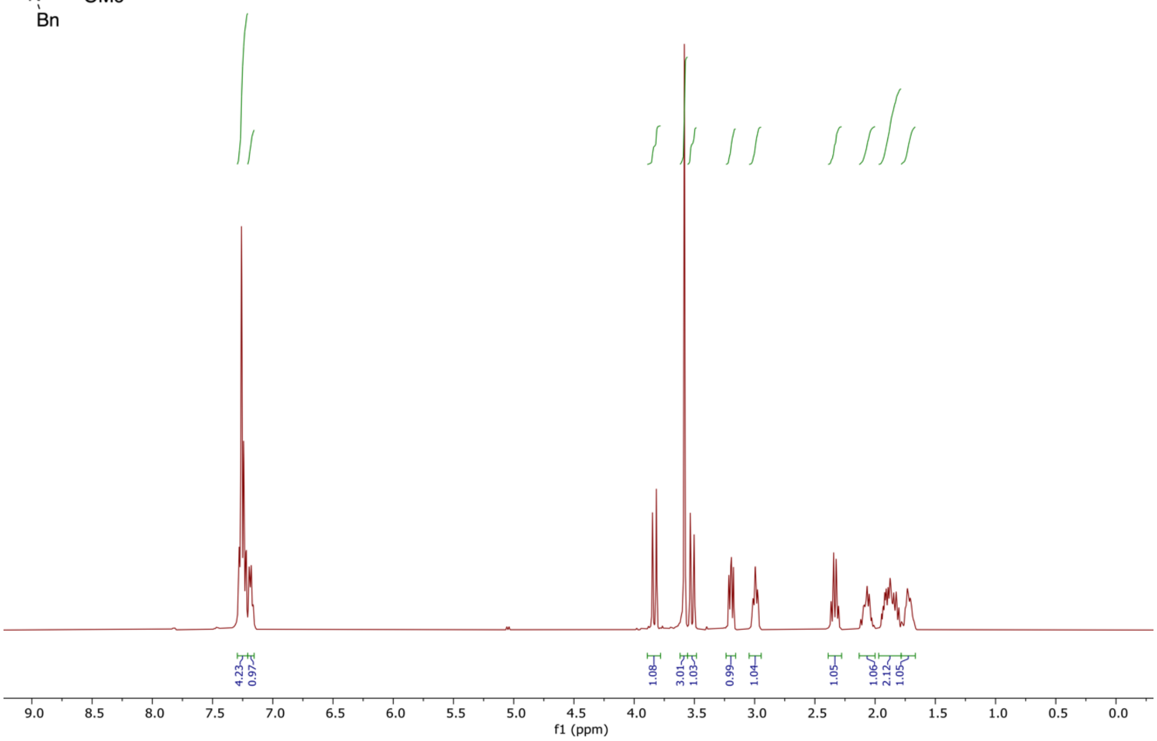
4.23
0.97
1.08
3.01
1.03
0.99
1.04
1.05
1.06
2.12
1.05
f1 (ppm)

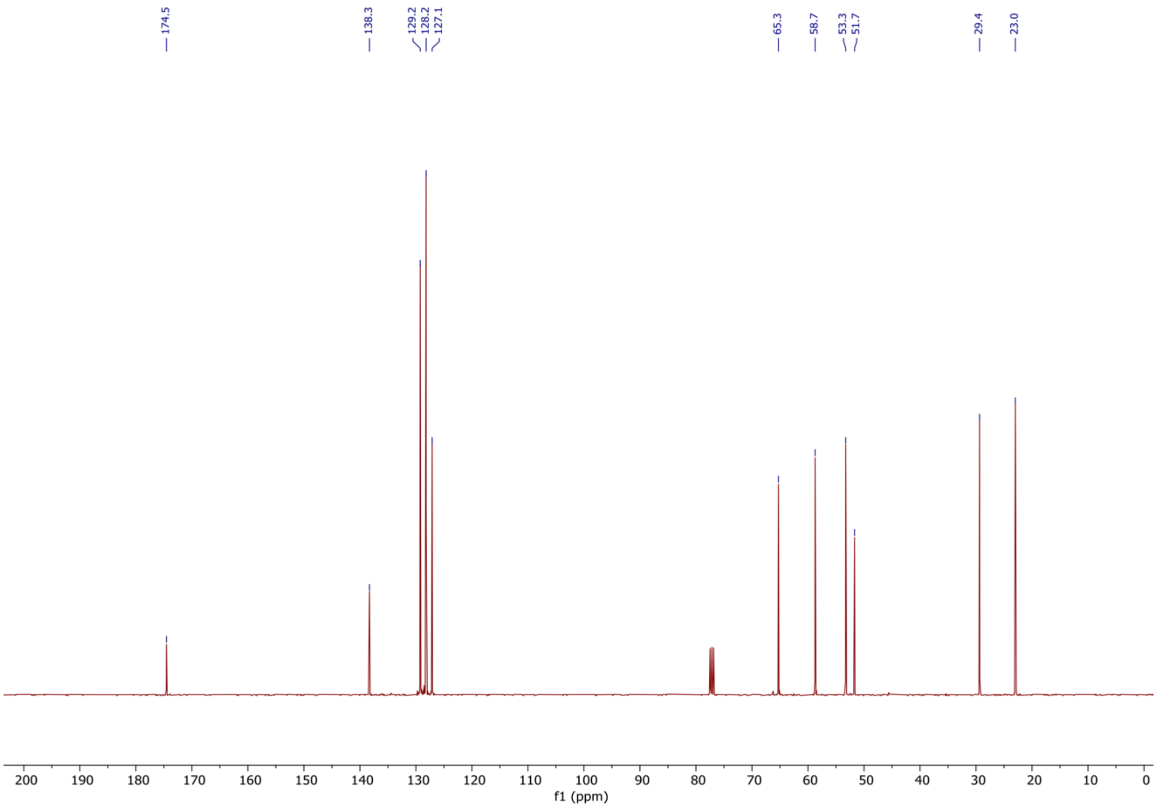
174.5
138.3
129.2
128.2
127.1
65.3
58.7
53.3
51.7
29.4
23.0
f1 (ppm)

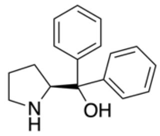
N
H
OH

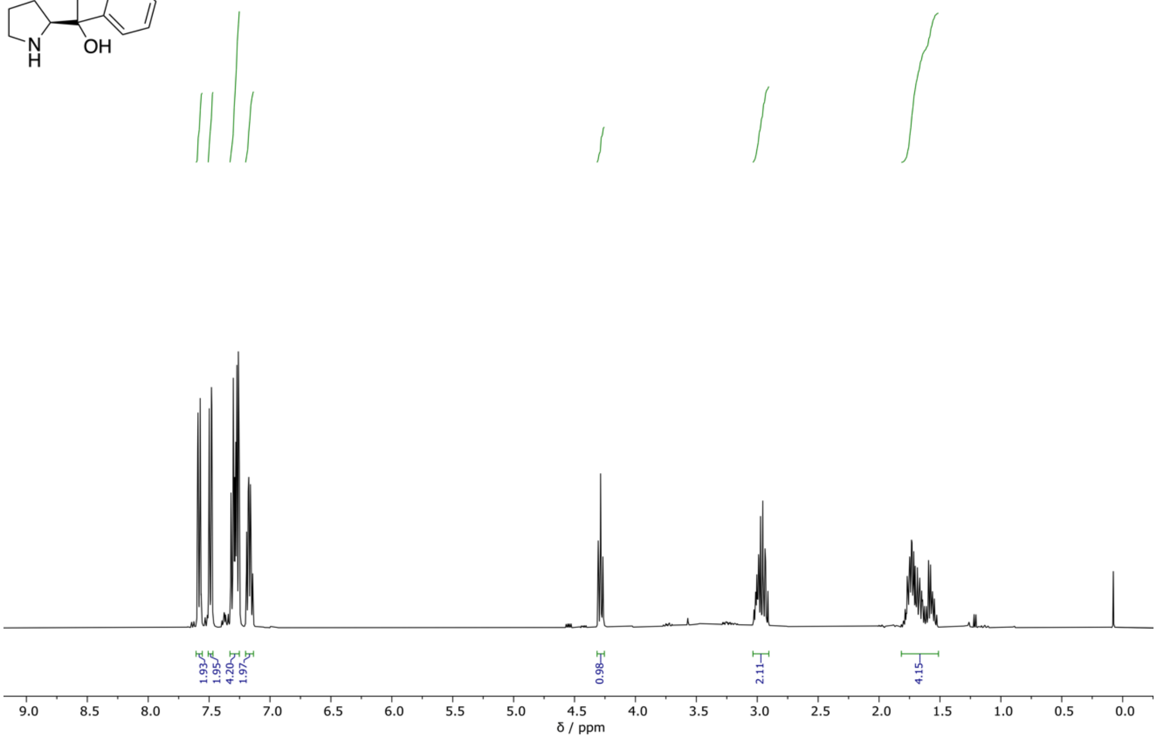
1.93
1.95
4.20
1.97
0.98
2.11
4.15
9.0
8.5
8.0
7.5
7.0
6.5
6.0
5.5
5.0
4.5
4.0
3.5
3.0
2.5
2.0
1.5
1.0
0.5
0.0
δ / ppm

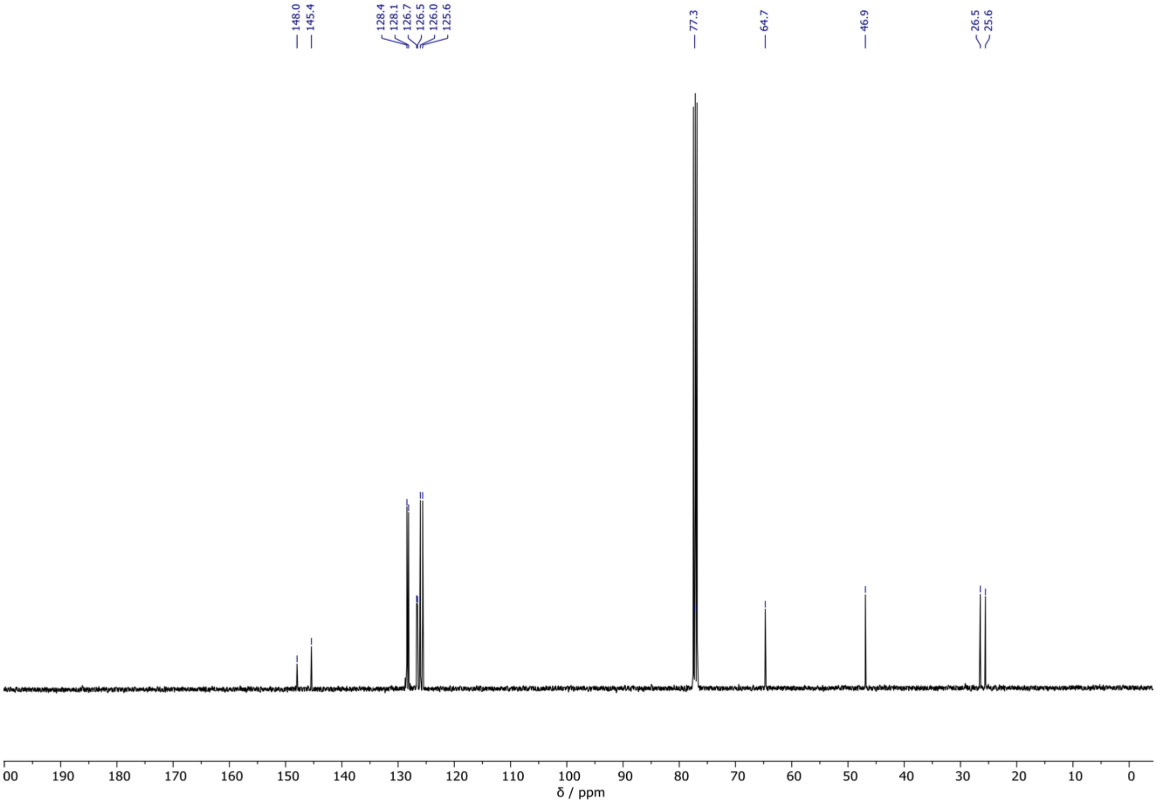
148.0
145.4
128.4
128.1
126.7
126.5
126.0
125.6
77.3
64.7
46.9
26.5
25.6
00
190
180
170
160
150
140
130
120
110
100
90
80
70
60
50
40
30
20
10
0
δ / ppm

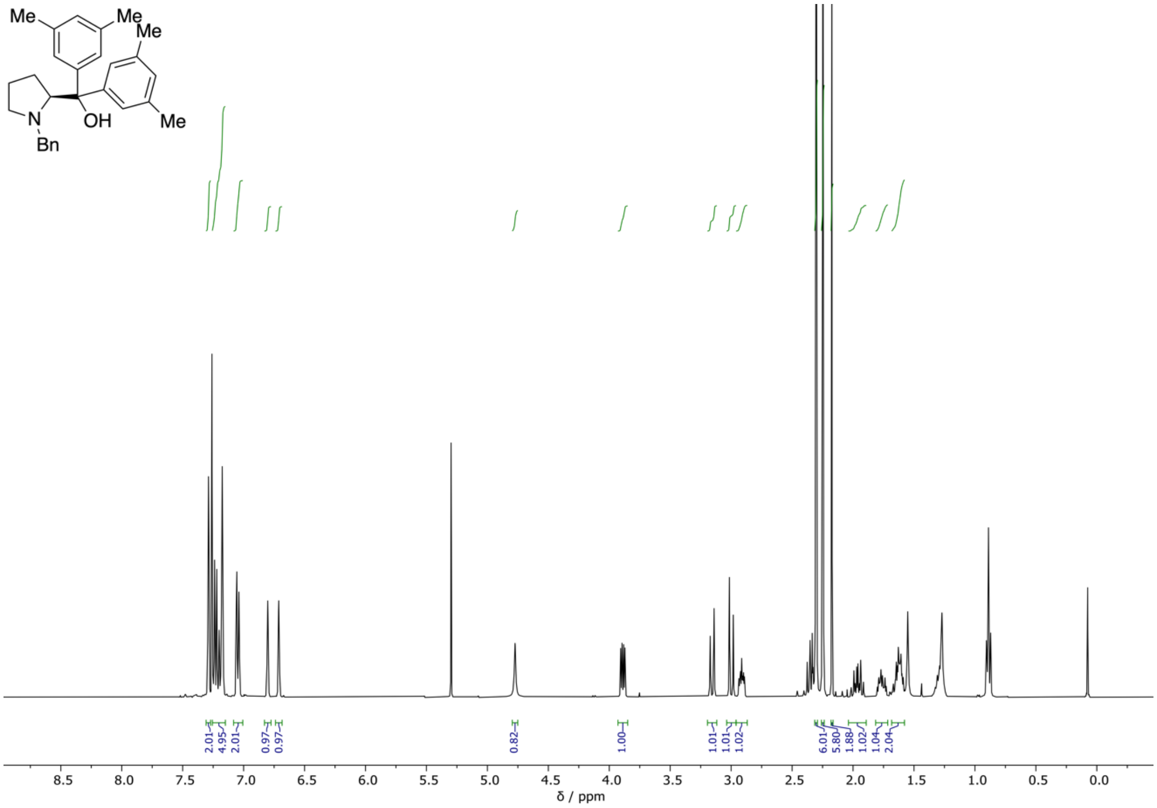
Me
Me
Me
N
Bn
OH
Me
2.01
4.95
2.01
0.97
0.97
0.82
1.00
1.01
1.01
1.02
6.01
5.80
1.88
1.02
1.04
2.04
8.5
8.0
7.5
7.0
6.5
6.0
5.5
5.0
4.5
4.0
3.5
3.0
2.5
2.0
1.5
1.0
0.5
0.0
δ / ppm

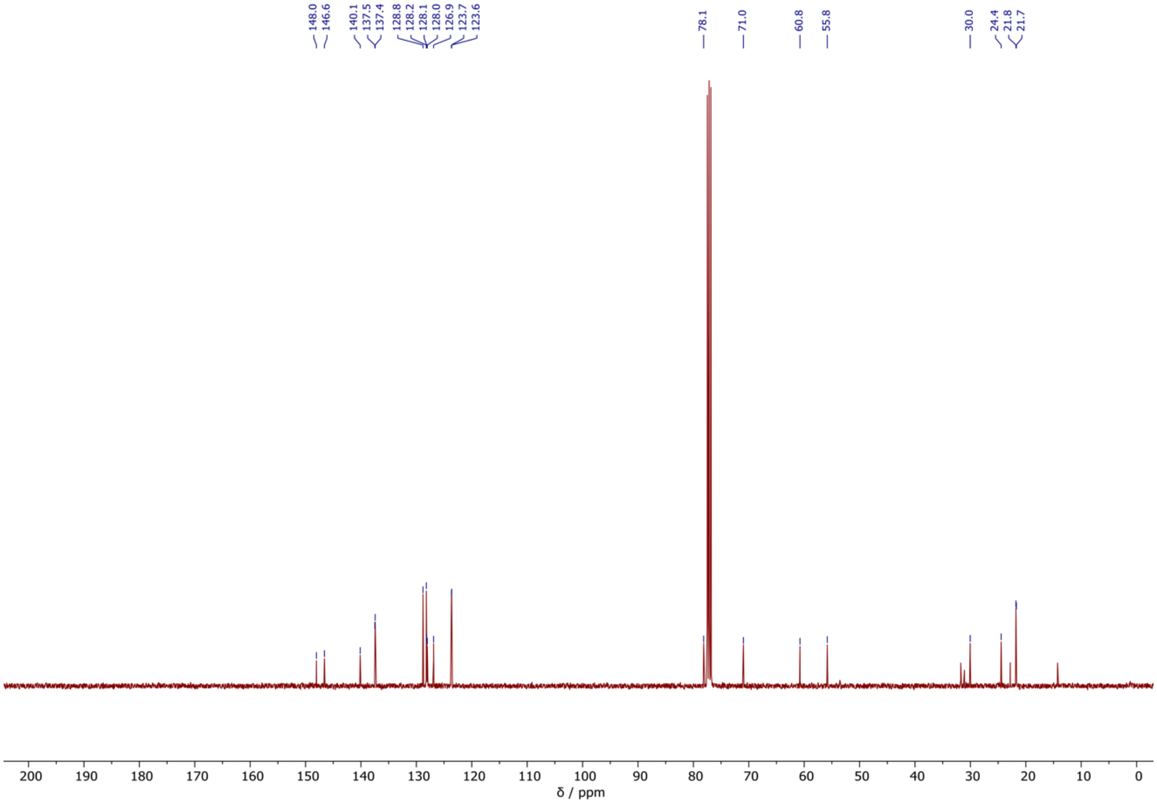
148.0
146.6
140.1
137.5
137.4
128.8
128.2
128.1
128.0
126.9
123.7
123.6
78.1
71.0
60.8
55.8
30.0
24.4
21.8
21.7
200
190
180
170
160
150
140
130
120
110
100
90
80
70
60
50
40
30
20
10
0
δ / ppm

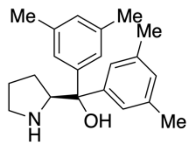
Me
Me
Me
Me
N
H
OH

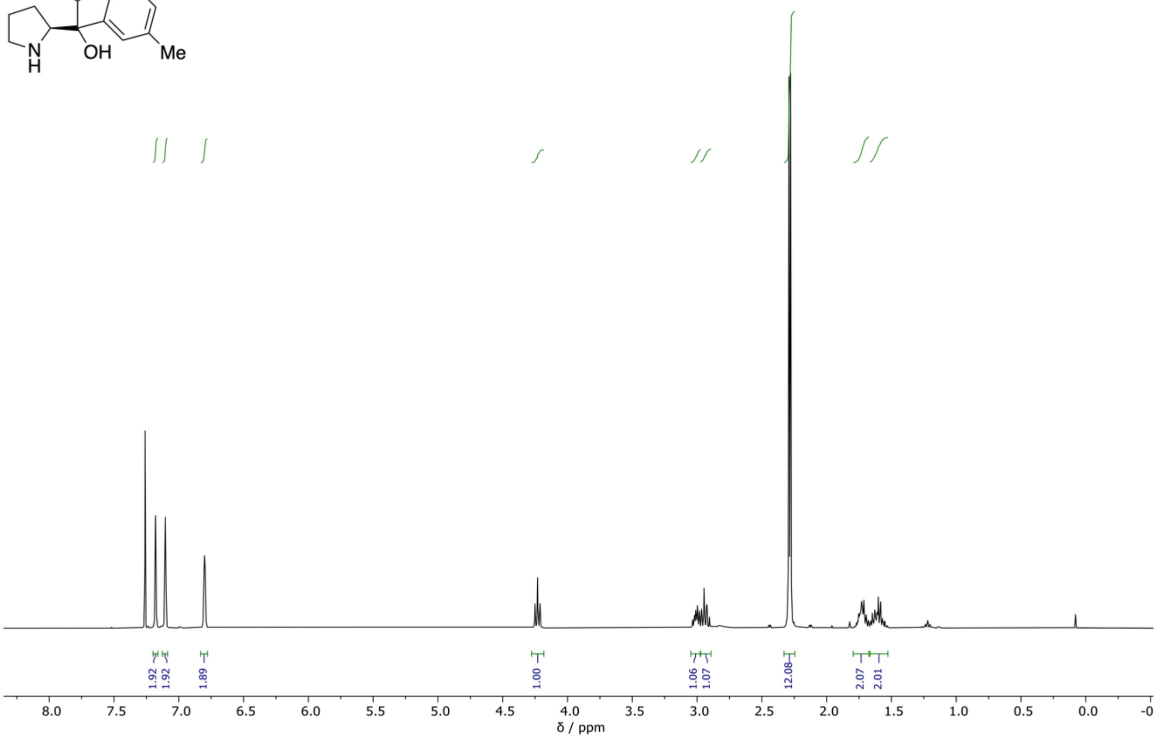
1.92
1.92
1.89
1.00
1.06
1.07
12.08
2.07
2.01
8.0
7.5
7.0
6.5
6.0
5.5
5.0
4.5
4.0
3.5
3.0
2.5
2.0
1.5
1.0
0.5
0.0
δ / ppm

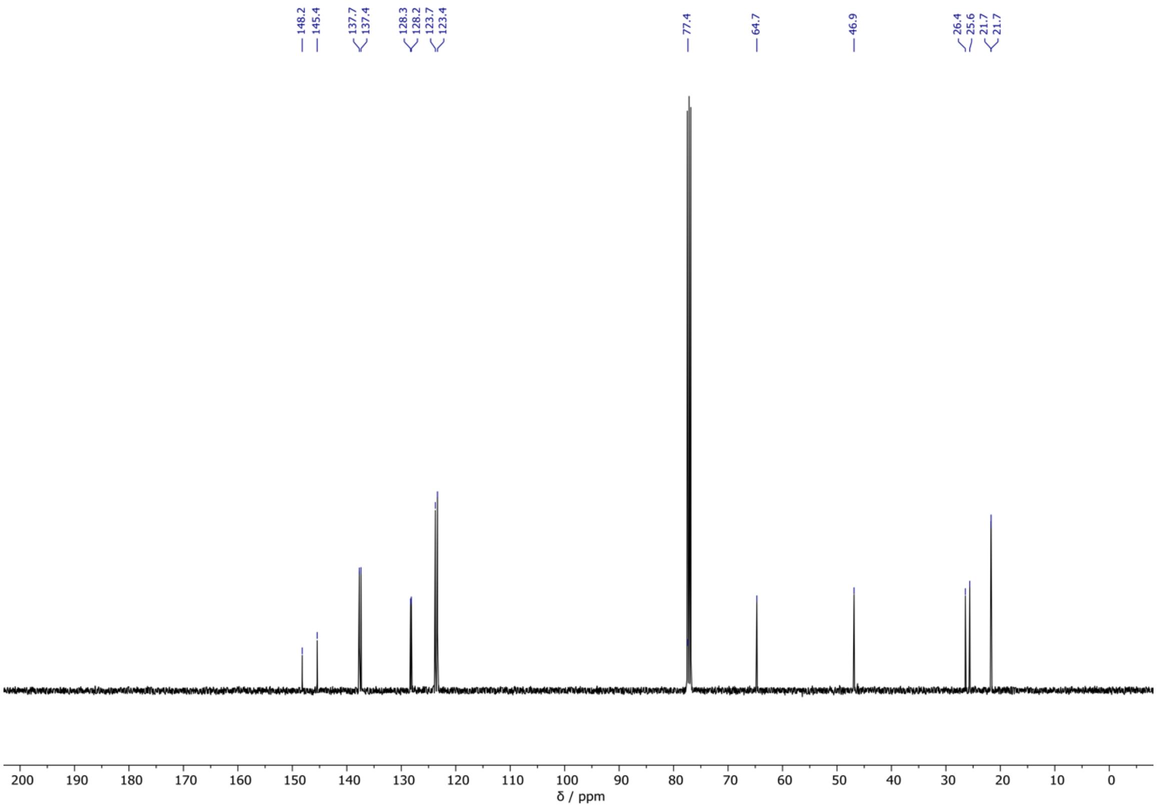
148.2
145.4
137.7
137.4
128.3
128.2
123.7
123.4
77.4
64.7
46.9
26.4
25.6
21.7
21.7
200
190
180
170
160
150
140
130
120
110
100
90
80
70
60
50
40
30
20
10
0
δ / ppm

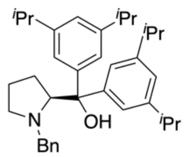

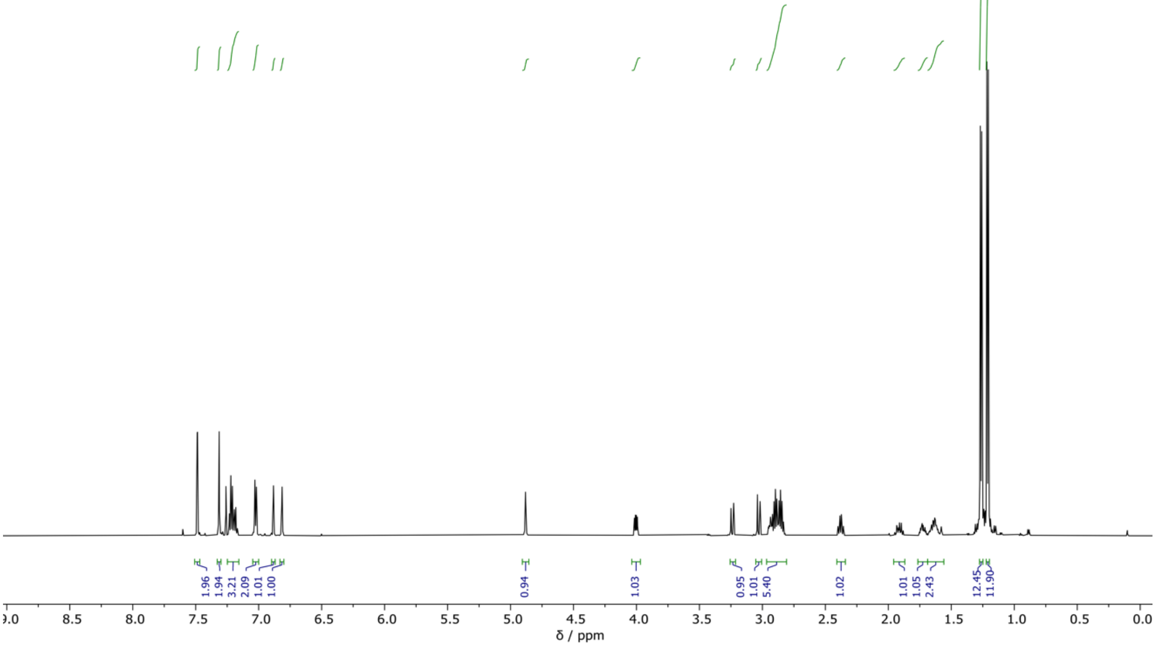

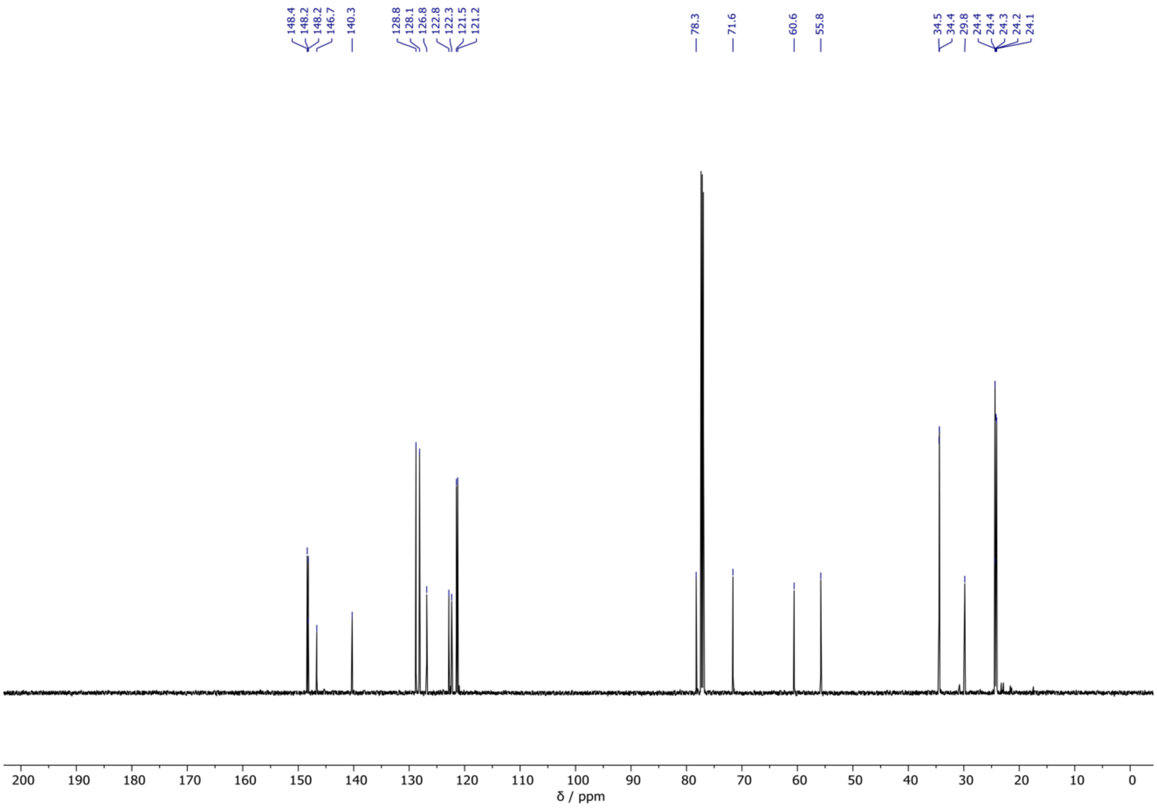

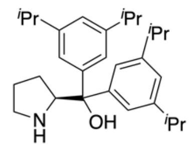

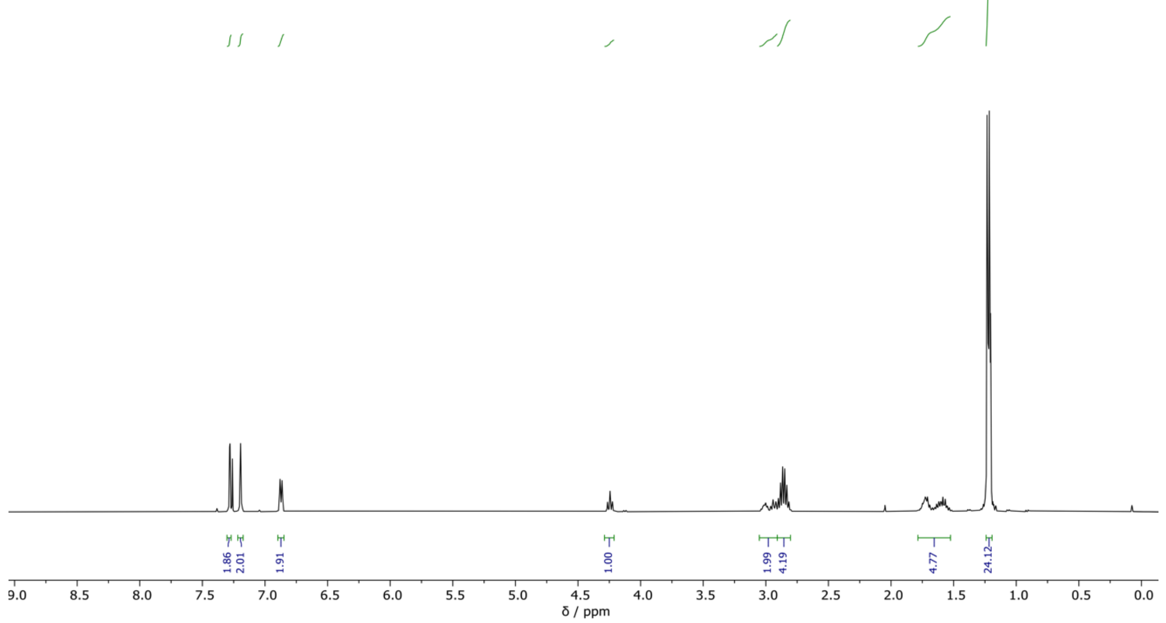

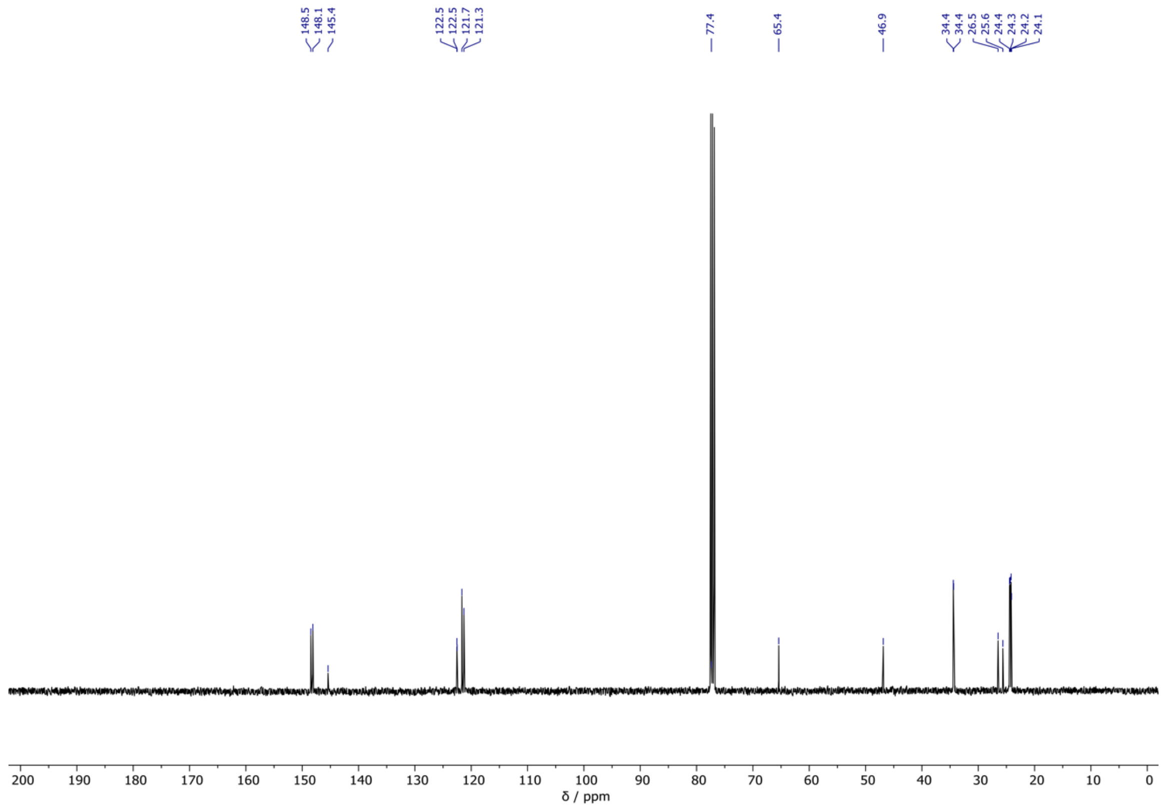

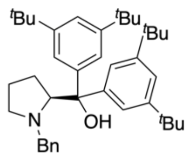
tBu
tBu
tBu
tBu
N
Bn
OH

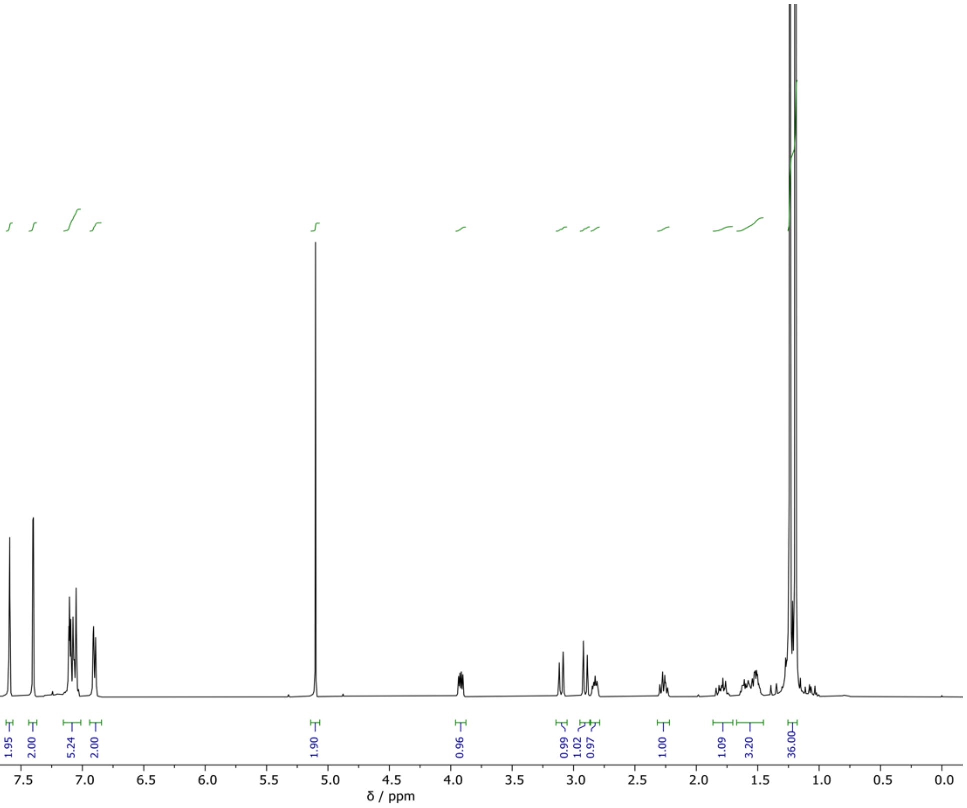
1.95
2.00
5.24
2.00
1.90
0.96
0.99
1.02
0.97
1.00
1.09
3.20
36.00
δ / ppm

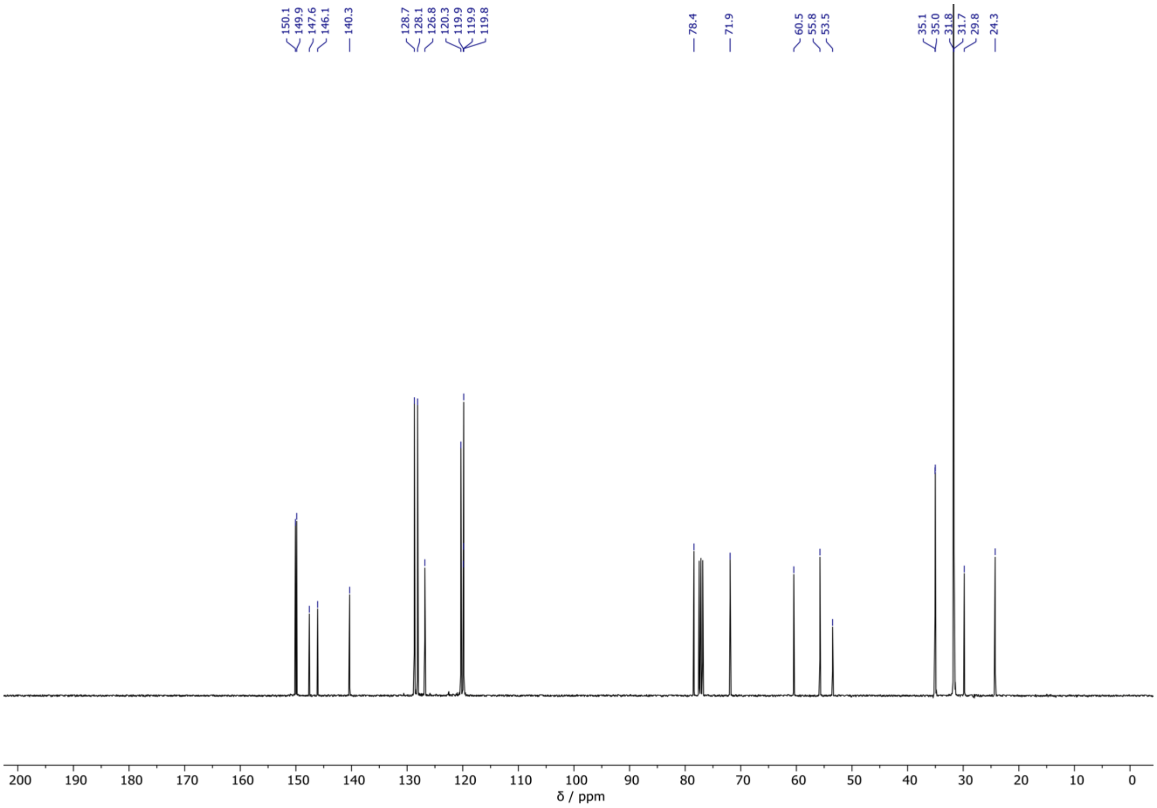
150.1
149.9
147.6
146.1
140.3
128.7
128.1
126.8
120.3
119.9
119.9
119.8
78.4
71.9
60.5
55.8
53.5
35.1
35.0
31.8
31.7
29.8
24.3
δ / ppm

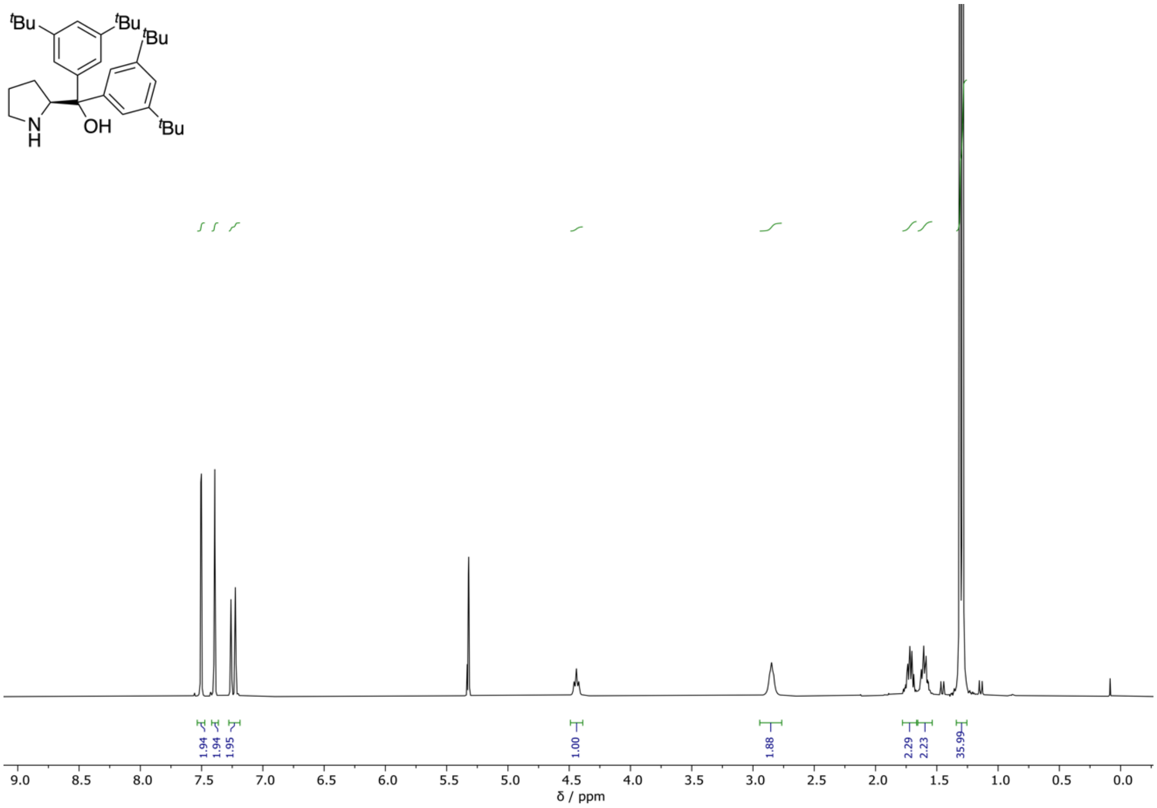

tBu
tBu
tBu
tBu
N
H
OH
1.94
1.94
1.95
1.00
1.88
2.29
2.23
35.99
9.0 8.5 8.0 7.5 7.0 6.5 6.0 5.5 5.0 4.5 4.0 3.5 3.0 2.5 2.0 1.5 1.0 0.5 0.0
δ / ppm

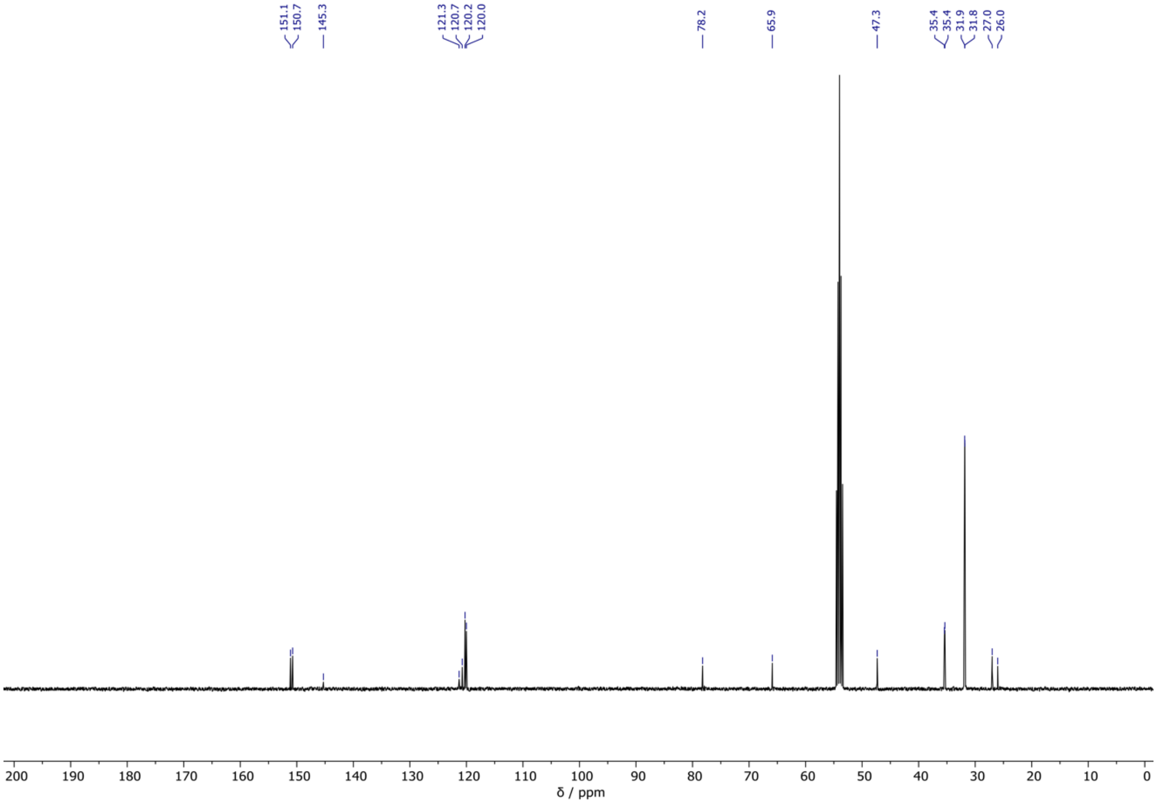

151.1
150.7
145.3
121.3
120.7
120.2
120.0
78.2
65.9
47.3
35.4
35.4
31.9
31.8
27.0
26.0
200 190 180 170 160 150 140 130 120 110 100 90 80 70 60 50 40 30 20 10 0
δ / ppm

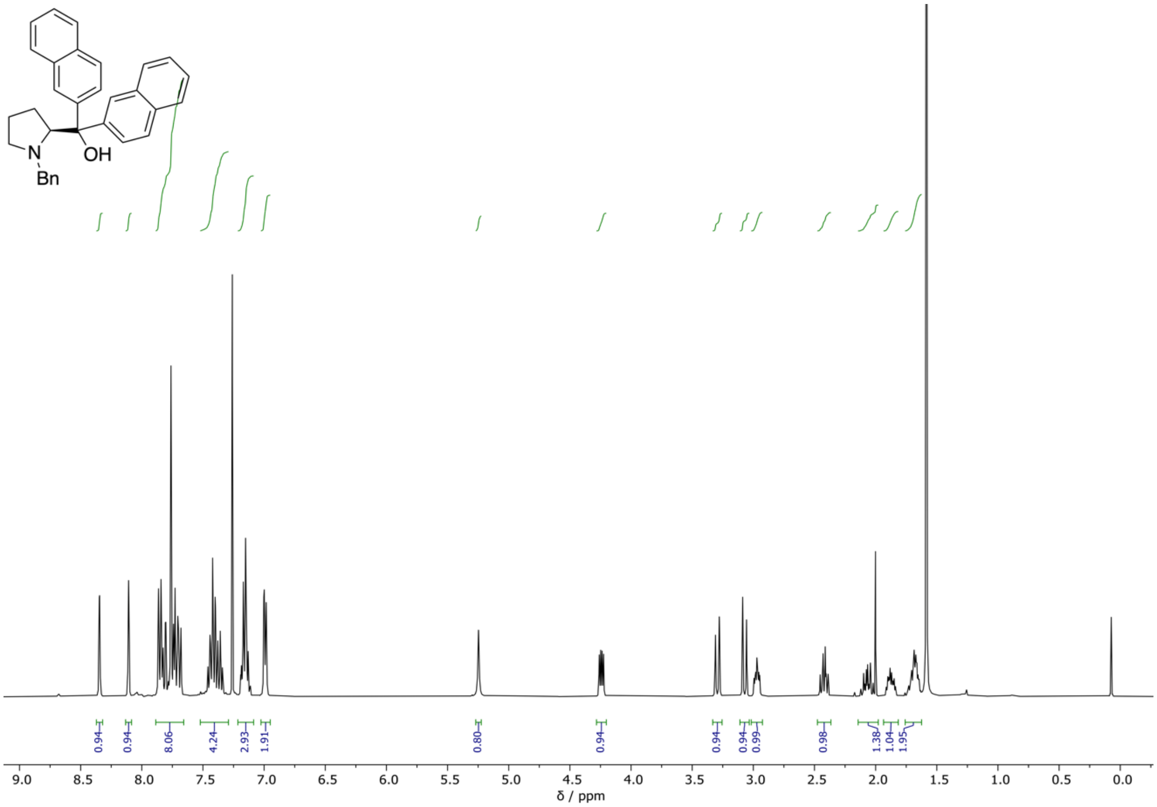
N
Bn
OH
0.94
0.94
8.06
4.24
2.93
1.91
0.80
0.94
0.94
0.94
0.99
0.98
1.38
1.04
1.95
9.0 8.5 8.0 7.5 7.0 6.5 6.0 5.5 5.0 4.5 4.0 3.5 3.0 2.5 2.0 1.5 1.0 0.5 0.0
δ / ppm

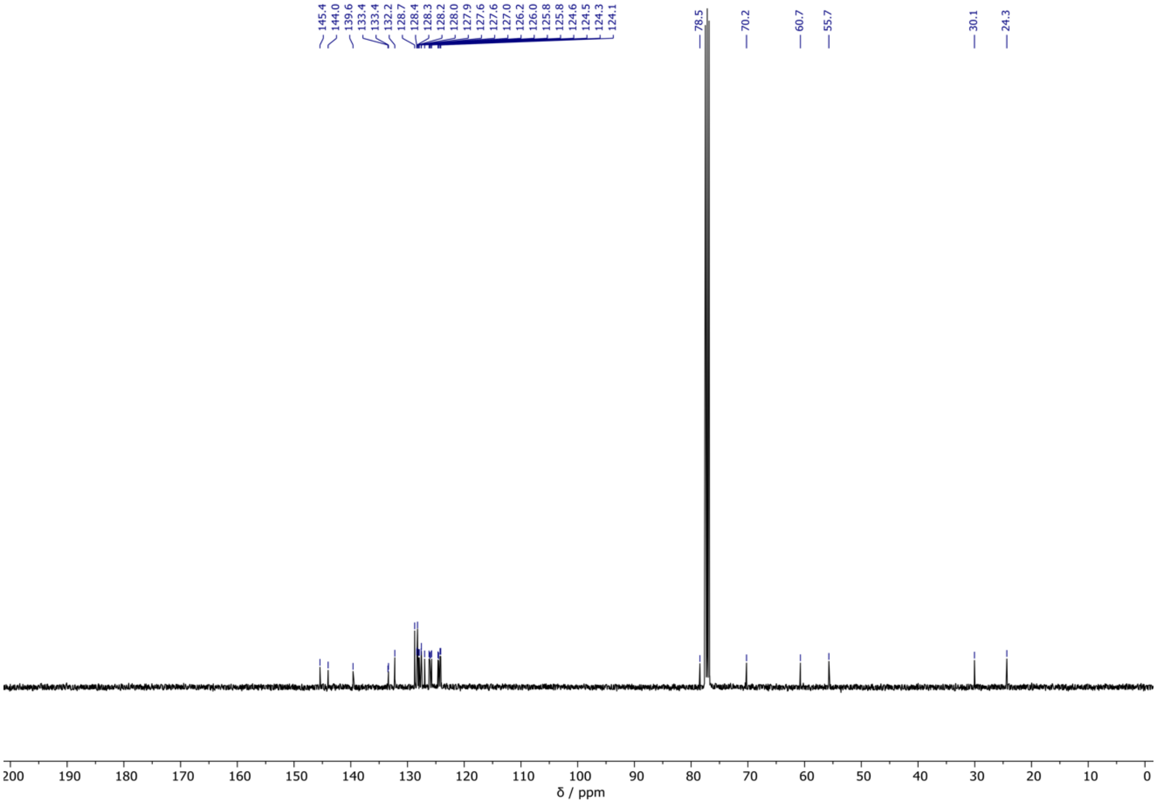
145.4
144.0
139.6
133.4
133.4
132.2
128.7
128.4
128.3
128.2
128.0
127.9
127.6
127.6
127.0
127.0
126.2
126.0
125.8
125.8
124.6
124.5
124.3
124.1
78.5
70.2
60.7
55.7
30.1
24.3
200 190 180 170 160 150 140 130 120 110 100 90 80 70 60 50 40 30 20 10 0
δ / ppm

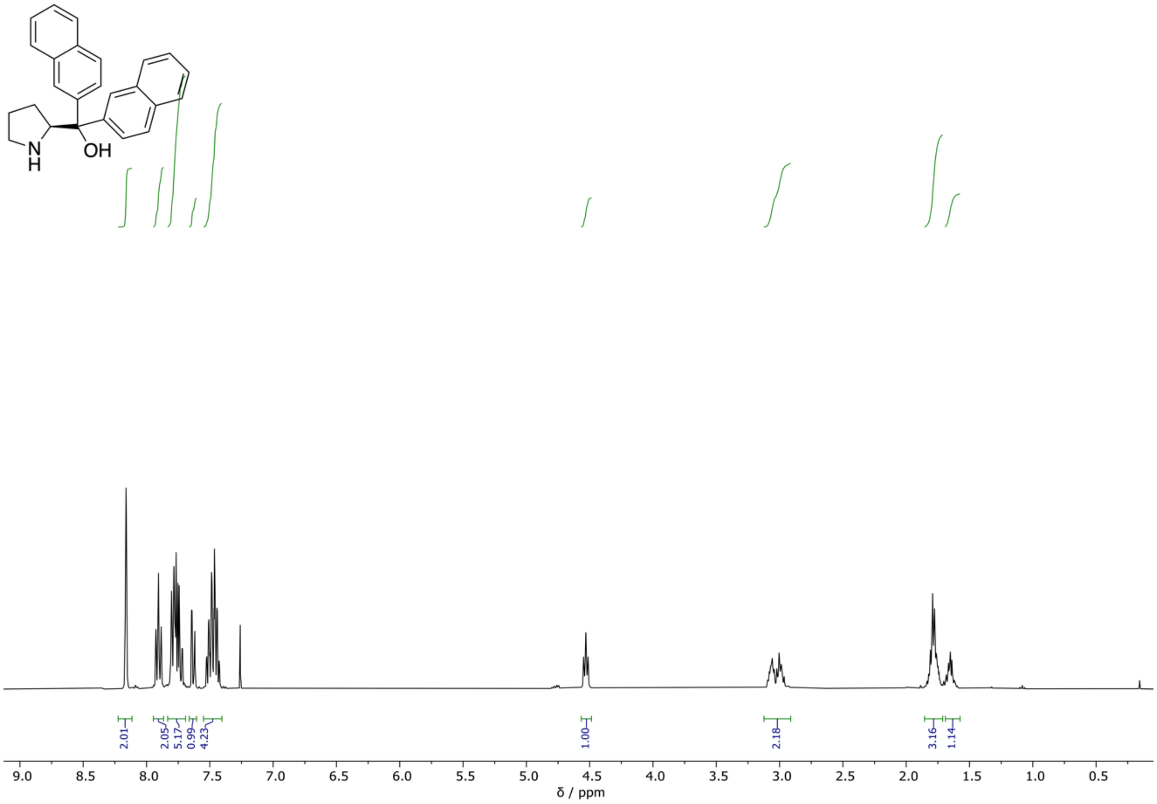
N
H
OH
2.01
2.05
5.17
0.99
4.23
1.00
2.18
3.16
1.14
9.0 8.5 8.0 7.5 7.0 6.5 6.0 5.5 5.0 4.5 4.0 3.5 3.0 2.5 2.0 1.5 1.0 0.5
δ / ppm

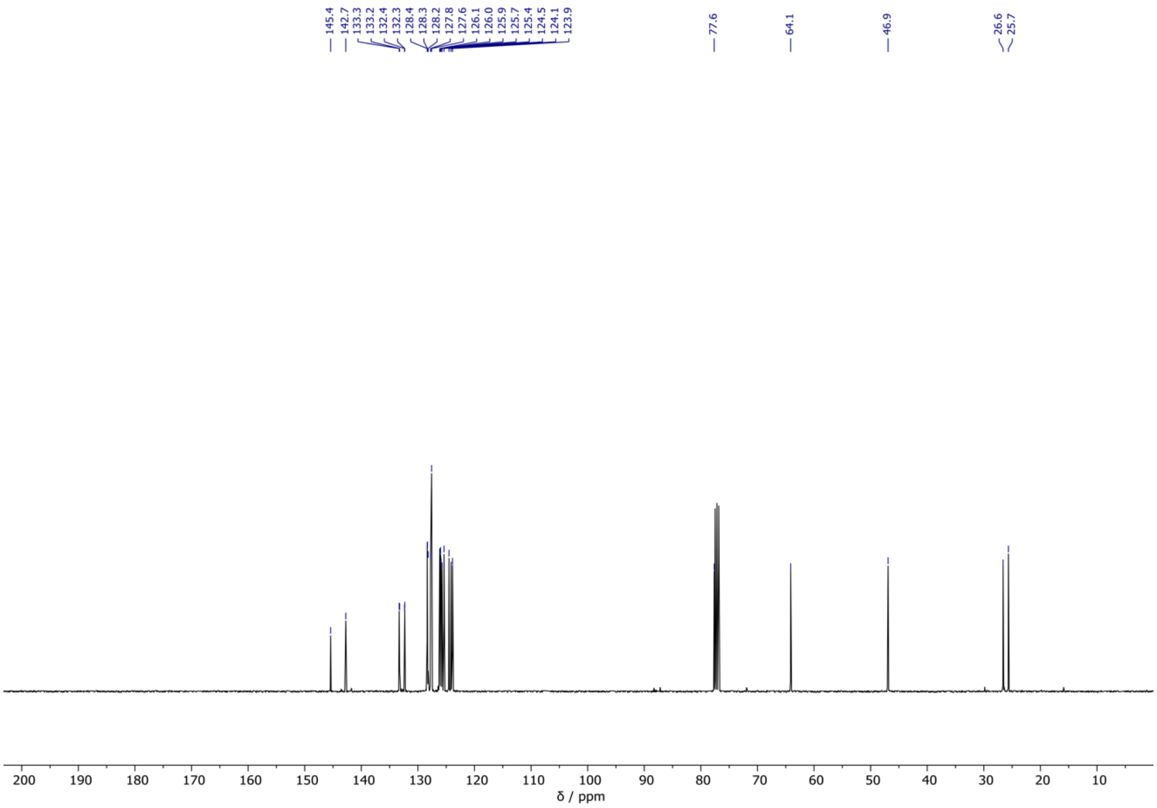
145.4
142.7
133.3
133.2
132.4
132.3
128.4
128.3
128.2
127.8
127.6
126.1
126.0
125.9
125.7
125.4
124.5
124.1
123.9
77.6
64.1
46.9
26.6
25.7
200 190 180 170 160 150 140 130 120 110 100 90 80 70 60 50 40 30 20 10
δ / ppm

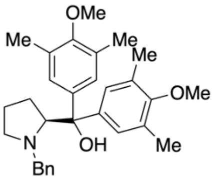

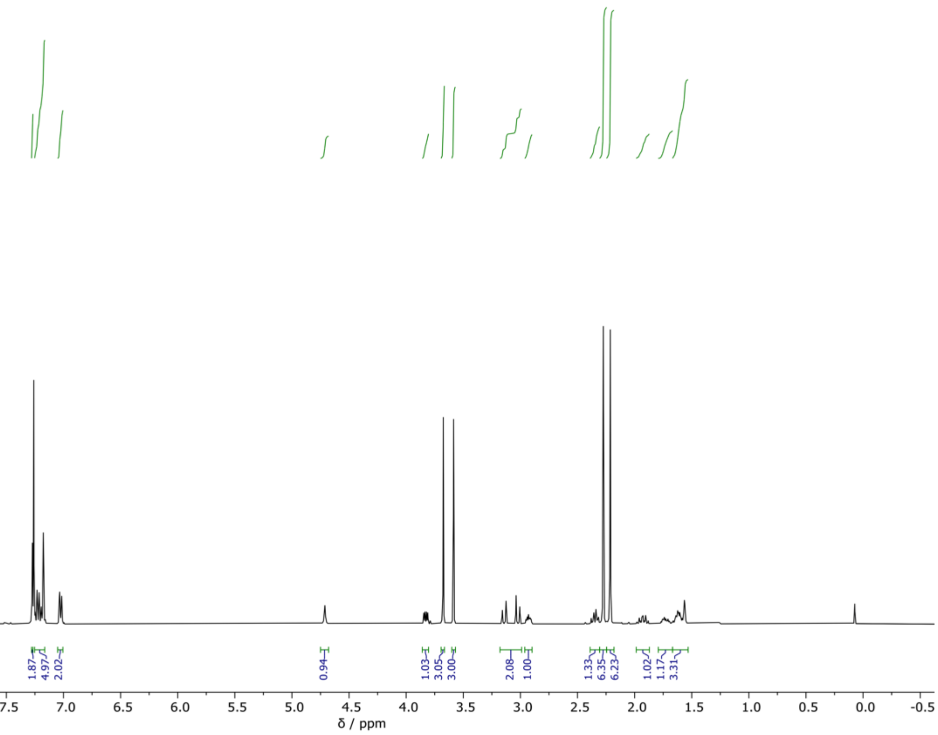
1.87
4.97
2.02
0.94
1.03
3.05
3.00
2.08
1.00
1.33
6.35
6.23
1.02
1.17
3.31
9.0 8.5 8.0 7.5 7.0 6.5 6.0 5.5 5.0 4.5 4.0 3.5 3.0 2.5 2.0 1.5 1.0 0.5 0.0 -0.5
δ / ppm

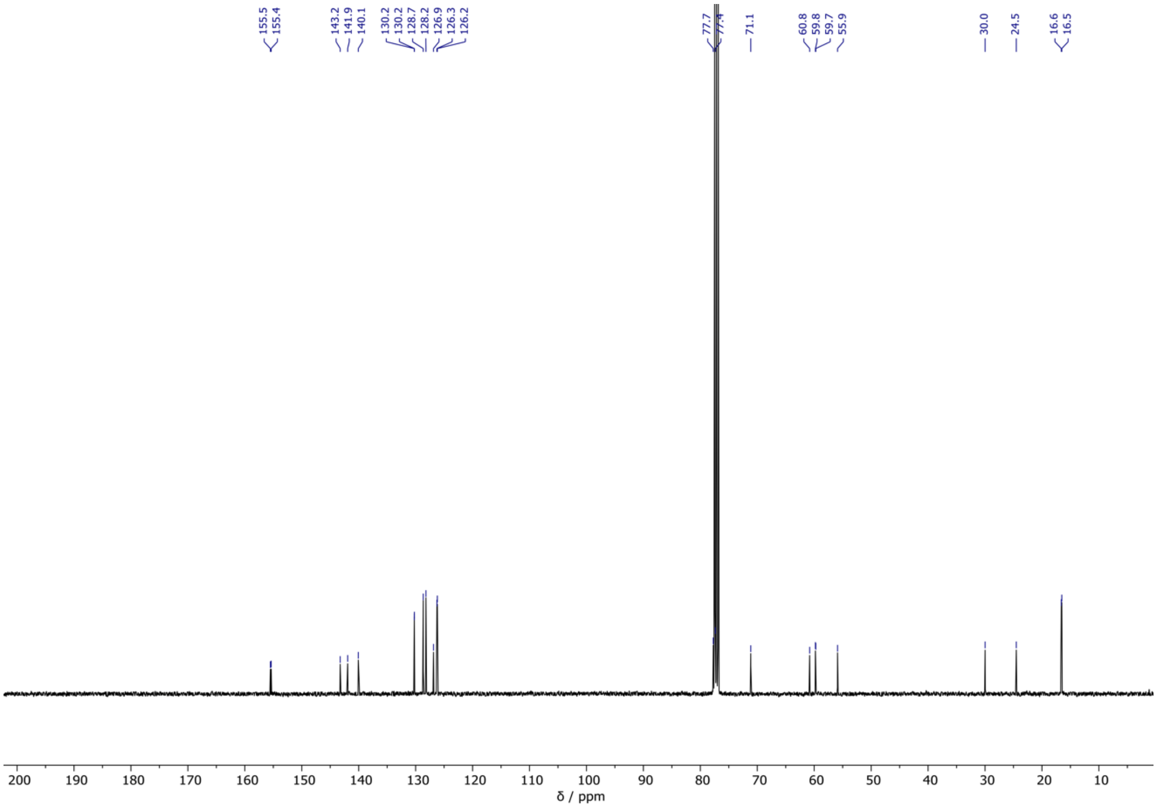
155.5
155.4
143.2
141.9
140.1
130.2
130.2
128.7
128.2
126.9
126.3
126.2
77.7
77.4
71.1
60.8
59.8
59.7
55.9
30.0
24.5
16.6
16.5
200 190 180 170 160 150 140 130 120 110 100 90 80 70 60 50 40 30 20 10
δ / ppm

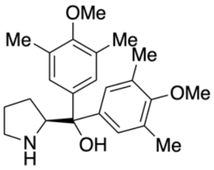
OMe
Me
Me
Me
OMe
Me
N
H
OH

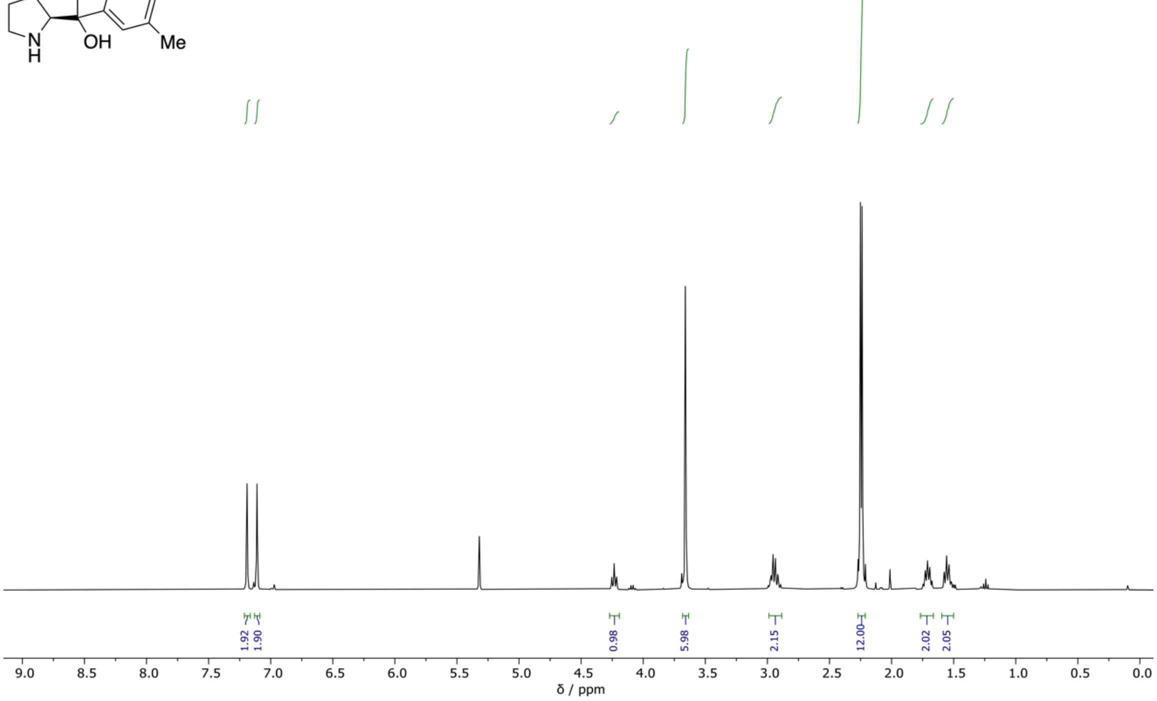
1.92
1.90
0.98
5.98
2.15
12.00
2.02
2.05
9.0 8.5 8.0 7.5 7.0 6.5 6.0 5.5 5.0 4.5 4.0 3.5 3.0 2.5 2.0 1.5 1.0 0.5 0.0
δ / ppm

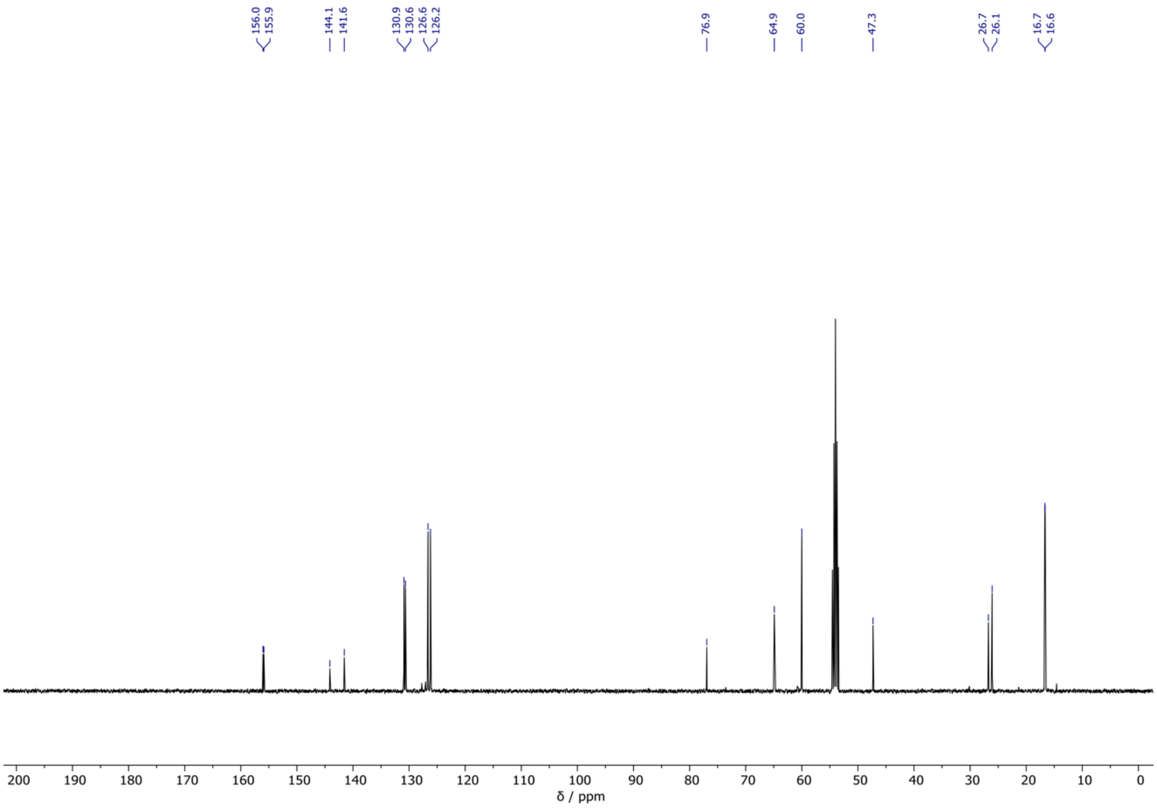
156.0
155.9
144.1
141.6
130.9
130.6
126.6
126.2
76.9
64.9
60.0
47.3
26.7
26.1
16.7
16.6
200 190 180 170 160 150 140 130 120 110 100 90 80 70 60 50 40 30 20 10 0
δ / ppm

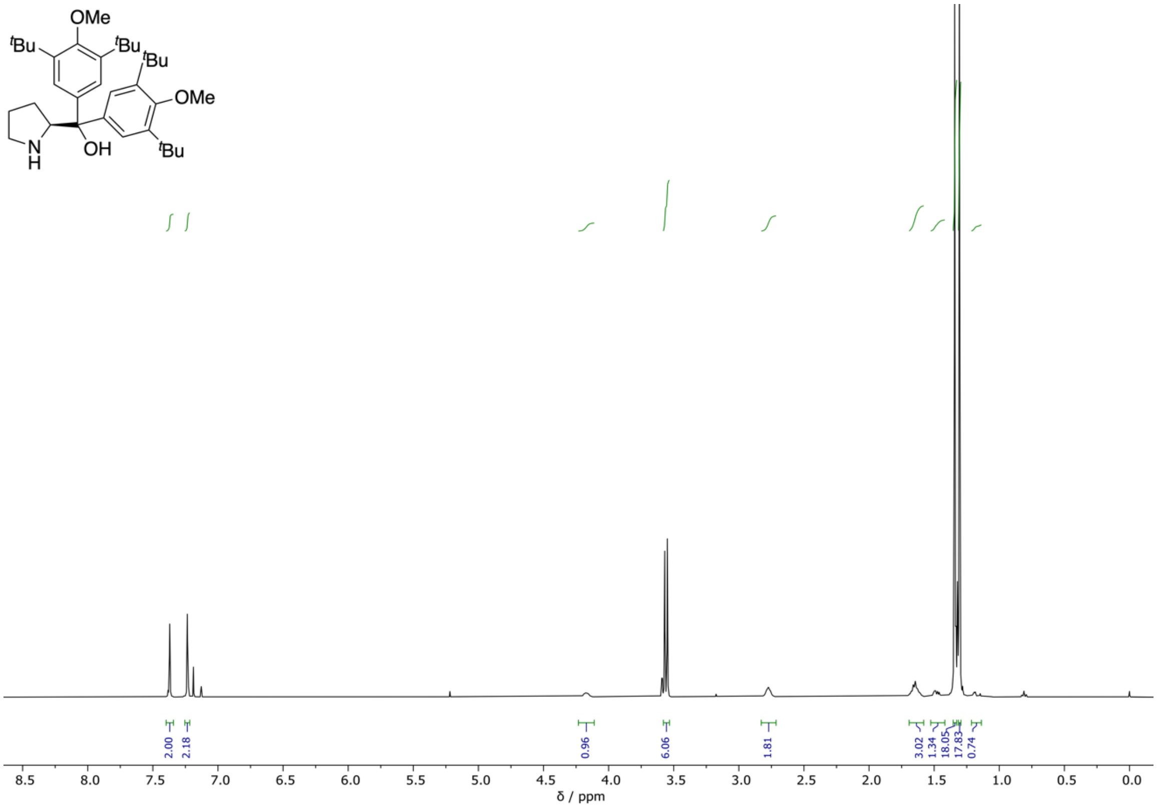
OMe
tBu
tBu
tBu
OMe
tBu
N
H
OH
2.00
2.18
0.96
6.06
1.81
3.02
1.34
18.05
17.83
0.74
8.5 8.0 7.5 7.0 6.5 6.0 5.5 5.0 4.5 4.0 3.5 3.0 2.5 2.0 1.5 1.0 0.5 0.0
δ / ppm

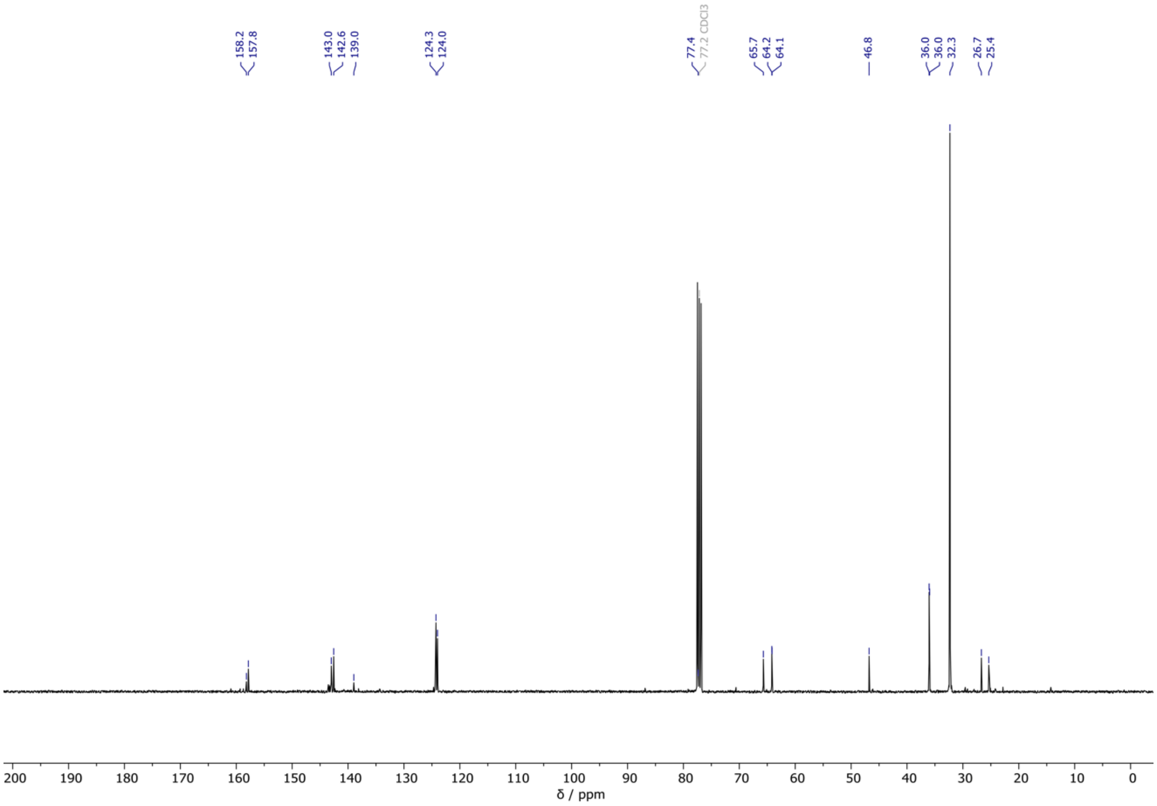
158.2
157.8
143.0
142.6
139.0
124.3
124.0
77.4
77.2 CDCl3
65.7
64.2
64.1
46.8
36.0
36.0
32.3
26.7
25.4
200 190 180 170 160 150 140 130 120 110 100 90 80 70 60 50 40 30 20 10 0
δ / ppm

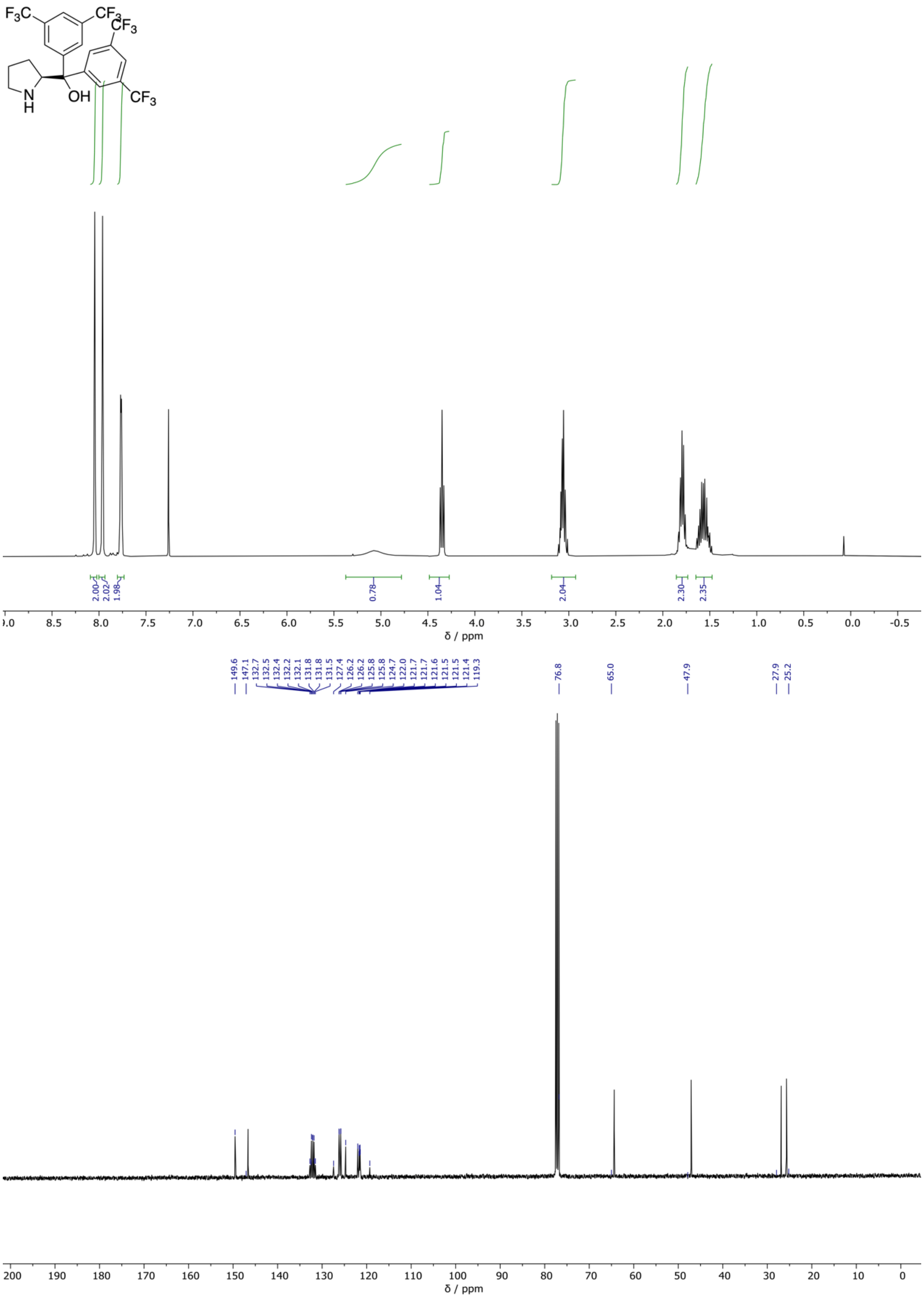

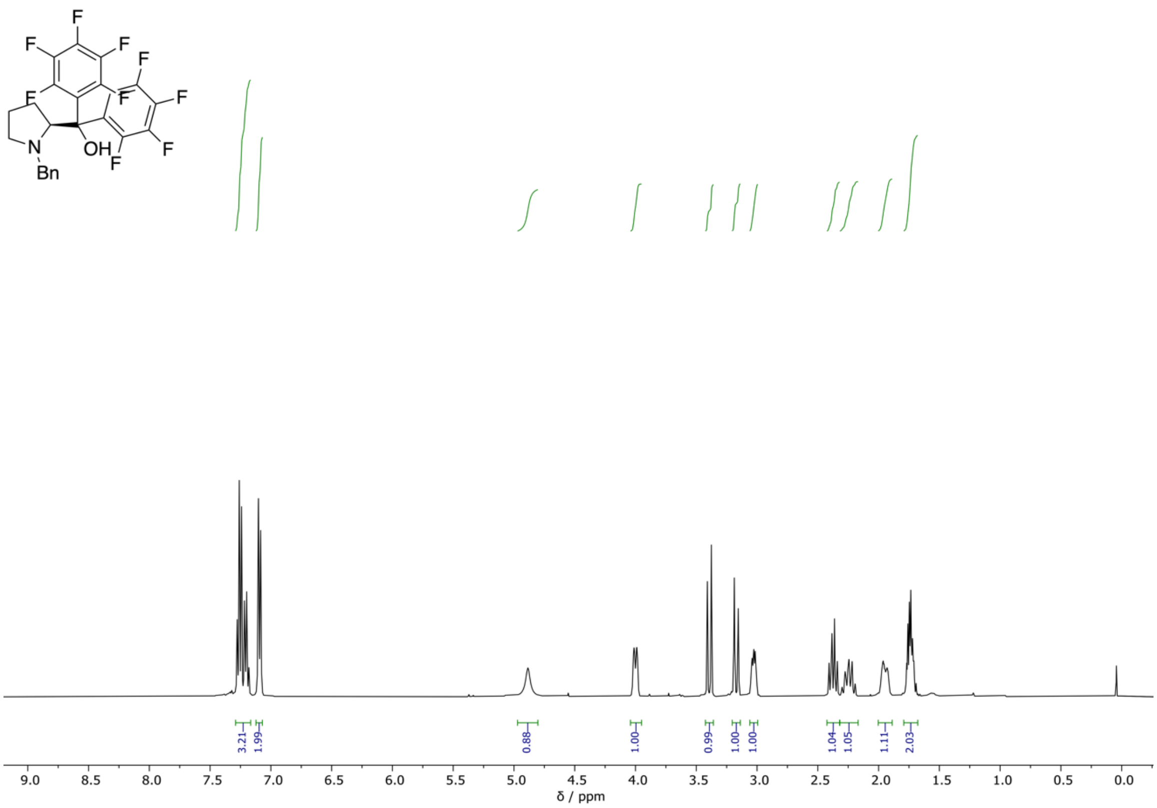
F
F
F
F
F
F
F
F
F
F
N
OH
Bn
3.21
1.99
0.88
1.00
0.99
1.00
1.00
1.04
1.05
1.11
2.03
9.0 8.5 8.0 7.5 7.0 6.5 6.0 5.5 5.0 4.5 4.0 3.5 3.0 2.5 2.0 1.5 1.0 0.5 0.0
δ / ppm

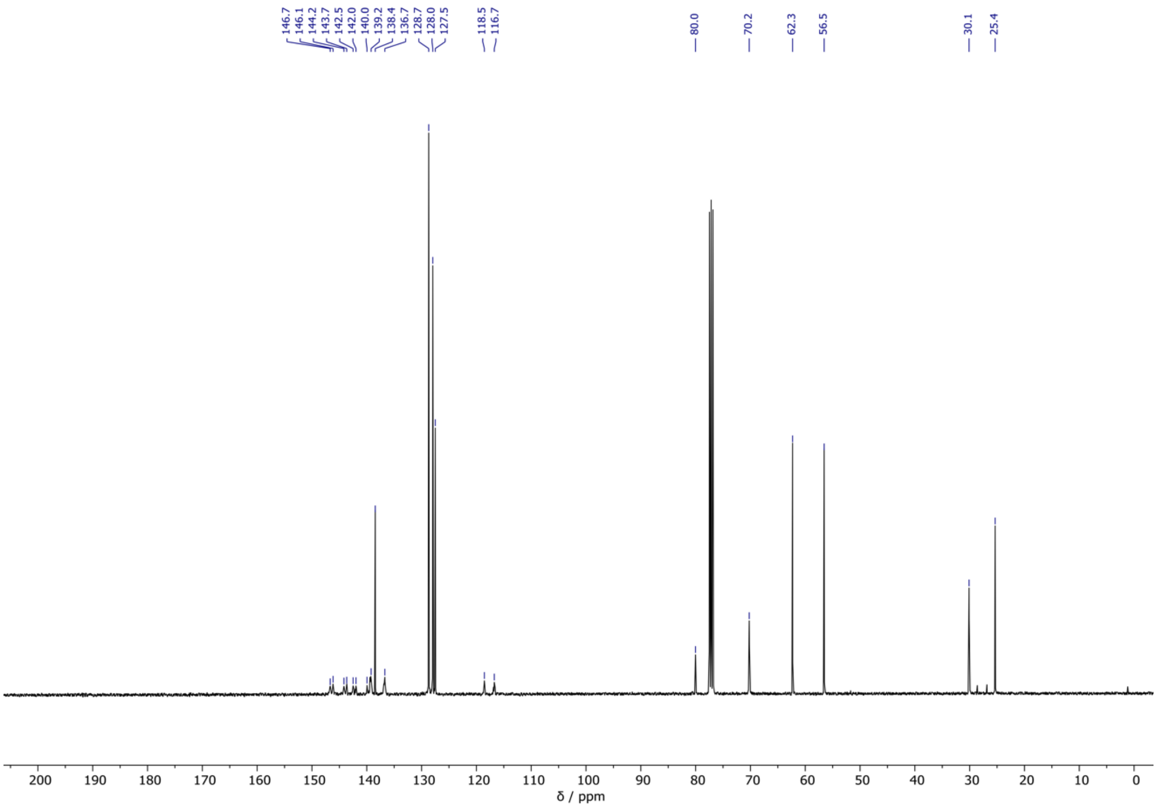
146.7
146.1
144.2
143.7
142.5
142.0
140.0
139.2
138.4
136.7
128.7
128.0
127.5
118.5
116.7
80.0
70.2
62.3
56.5
30.1
25.4
200 190 180 170 160 150 140 130 120 110 100 90 80 70 60 50 40 30 20 10 0
δ / ppm

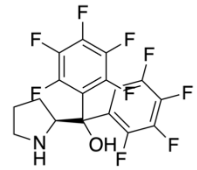
F
F
F
F
F
F
F
F
F
F
N
H
OH

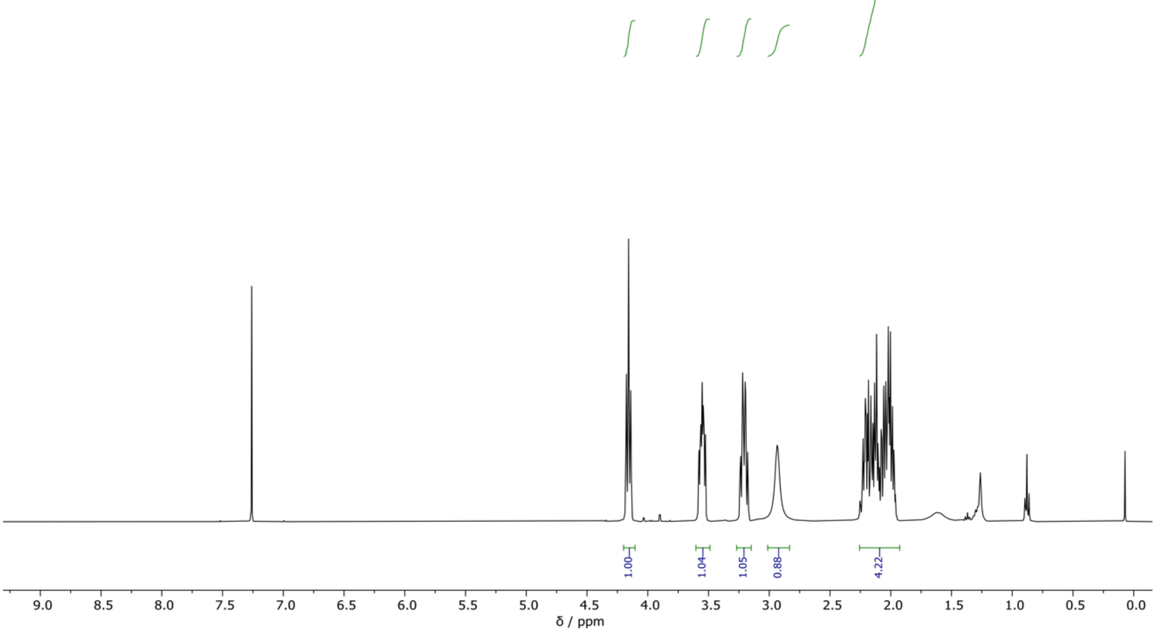
1.00
1.04
1.05
0.88
4.22
9.0
8.5
8.0
7.5
7.0
6.5
6.0
5.5
5.0
4.5
4.0
3.5
3.0
2.5
2.0
1.5
1.0
0.5
0.0
δ / ppm

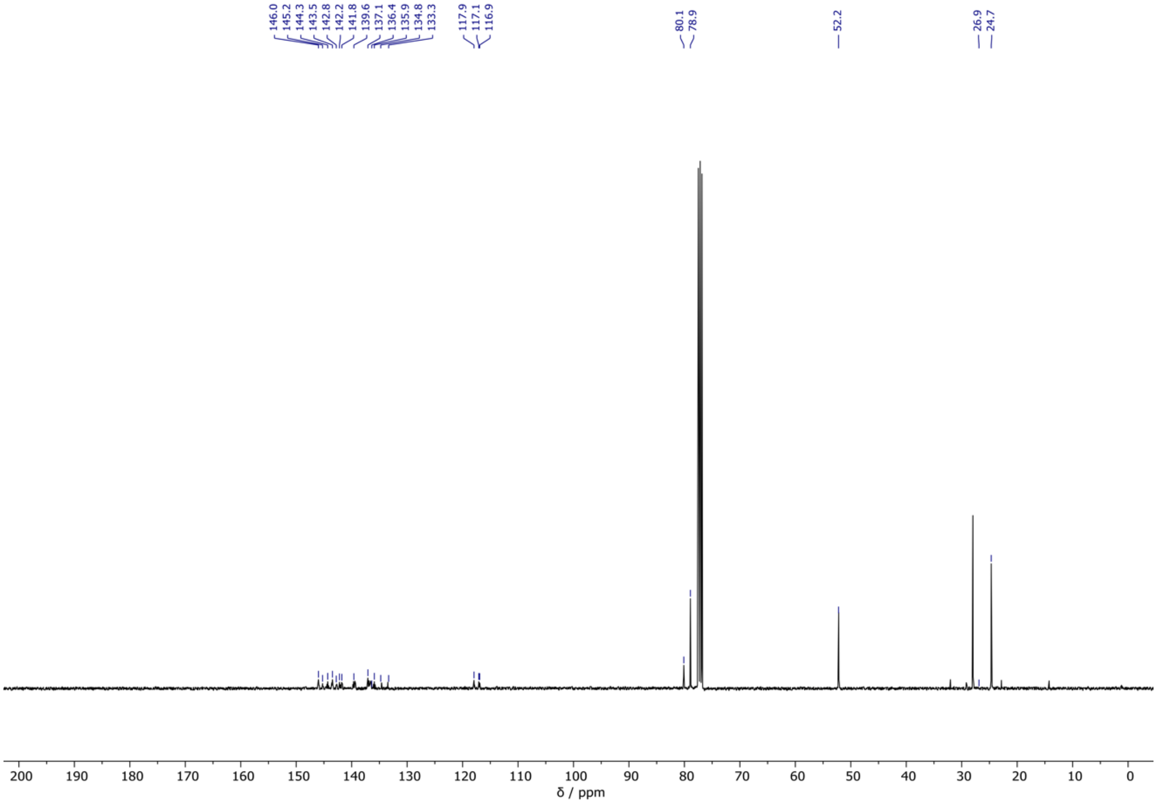
146.0
145.2
144.3
143.5
142.8
142.2
141.8
139.6
137.1
136.4
135.9
134.8
133.3
117.9
117.1
116.9
80.1
78.9
52.2
26.9
24.7
200
190
180
170
160
150
140
130
120
110
100
90
80
70
60
50
40
30
20
10
0
δ / ppm

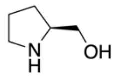
N
H
OH

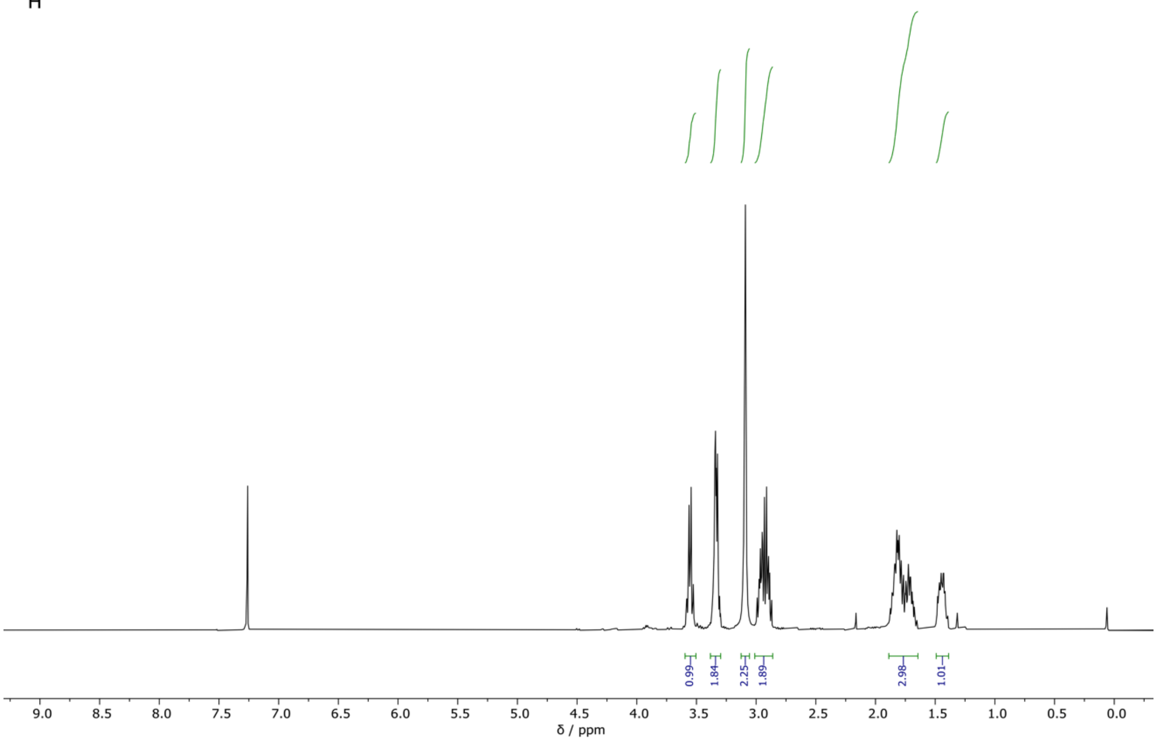
0.99
1.84
2.25
1.89
2.98
1.01
9.0
8.5
8.0
7.5
7.0
6.5
6.0
5.5
5.0
4.5
4.0
3.5
3.0
2.5
2.0
1.5
1.0
0.5
0.0
δ / ppm

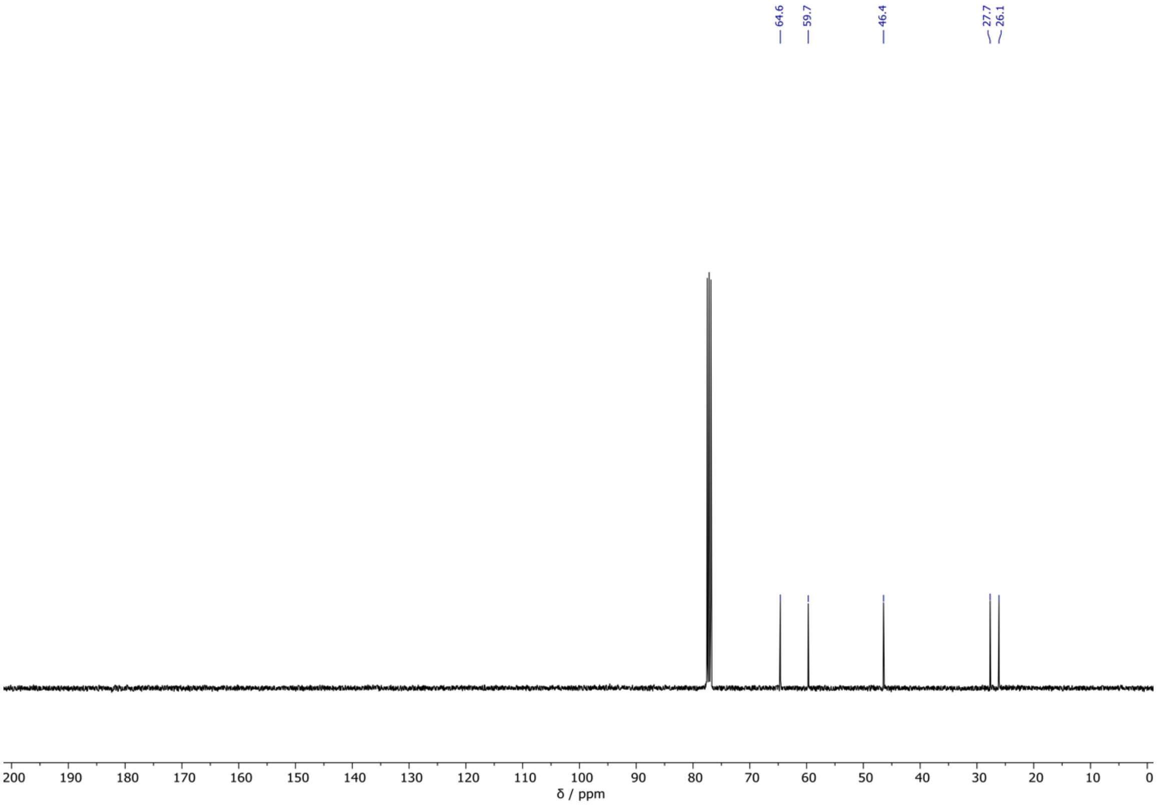
64.6
59.7
46.4
27.7
26.1
200
190
180
170
160
150
140
130
120
110
100
90
80
70
60
50
40
30
20
10
0
δ / ppm

13. Computational Data

Name / Structure	*E* / a.u.	∆*G*(corr)	∆*G*
THF	-232.540987	0.087989	-232.452998
MeCOPh	-385.044840	0.104972	-384.939868
cat	-853.207256	0.296542	-852.910714
BH3	-26.622612	0.008599	-26.614013
**1**	-259.206219	0.118246	-259.087973
**1'**	-1085.758582	0.405473	-1085.353109
**2**	-879.882651	0.328190	-879.554461
**2'**	-1112.434062	0.445663	-1111.988399
**3**	-1264.931566	0.461185	-1264.470381
**TS1R**	-1264.926207	0.462422	-1264.463785
**TS1R'**	-1264.920898	0.463202	-1264.457696
**TS1S**	-1264.920190	0.459673	-1264.460517
**TS1S'**	-1264.917798	0.461617	-1264.456181
**4R**	-1264.968603	0.458608	-1264.509995
**TS2R**	-1264.949252	0.461697	-1264.487555
**5R**	-1264.977859	0.465336	-1264.512523
**TS3R**	-1291.599580	0.492289	-1291.107291
**6R**	-1291.634902	0.497044	-1291.137858
**7**	-411.736562	0.137673	-411.598889
**s-B5-TS1R**	-958.756071	0.398213	-958.357858
**s-B5-TS1R'**	-958.754133	0.397313	-958.356820
**s-B5-TS1S**	-958.752018	0.398844	-958.353174
**s-B5-TS1S'**	-958.752838	0.399630	-958.353208
**TS4R**	-1268.563037	0.529504	-1268.033533
**TS4R'**	-1268.561383	0.530274	-1268.031109
**TS4S**	-1268.561890	0.529804	-1268.032086
**TS4S'**	-1268.556669	0.529898	-1268.026771
**TS5R**	-1191.109392	0.493686	-1190.615706
**TS5S**	-1191.101892	0.492563	-1190.609329

**TS5S'**	-1191.101356	0.492470	-1190.608886
**TS6R**	-1151.779237	0.466232	-1151.313005
**TS6R'**	-1151.774653	0.465795	-1151.308858
**TS6S**	-1151.777105	0.465266	-1151.311839
**TS6S'**	-1151.770626	0.466757	-1151.303869
**TS7R**	-1112.448937	0.439947	-1112.008990
**TS7S**	-1112.446183	0.438100	-1112.008083
**TS8R**	-1562.727856	0.433732	-1562.294124
**TS8R'**	-1562.727856	0.433736	-1562.294120
**TS8S**	-1562.726062	0.432931	-1562.293131
**TS8S'**	-1562.726166	0.432925	-1562.293241
**TS9R**	-1953.025970	0.464726	-1952.561244
**TS9R'**	-1953.016286	0.464376	-1952.551910
**TS9S**	-1953.026983	0.465474	-1952.561509
**TS9S'**	-1953.026994	0.465472	-1952.561522
**B1-TS4R**	-1503.338572	0.675444	-1502.663128
**B1-TS4R'**	-1503.329840	0.675340	-1502.654500
**B1-TS4S**	-1503.333013	0.673937	-1502.659076
**B1-TS4S'**	-1503.328415	0.672926	-1502.655489
**B2-TS4R**	-1464.000475	0.648709	-1463.351766
**B2-TS4R'**	-1463.998198	0.647855	-1463.350343
**B2-TS4S**	-1463.996700	0.647603	-1463.349097
**B2-TS4S'**	-1463.995689	0.647855	-1463.347834
**B3-TS4R**	-1424.662194	0.622430	-1424.039764
**B3-TS4R'**	-1424.661736	0.620756	-1424.040980
**B3-TS4S**	-1424.661177	0.622231	-1424.038946
**B3-TS4S'**	-1424.657641	0.620200	-1424.037441
**B4-TS4R**	-1386.540155	0.612010	-1385.928145
**B4-TS4R'**	-1386.538401	0.613193	-1385.925208
**B4-TS4S**	-1386.537346	0.611649	-1385.925697
**B4-TS4S'**	-1386.530368	0.610903	-1385.919465

**B5-TS4R**	-1347.214290	0.584140	-1346.630150
**B5-TS4S**	-1347.211885	0.583987	-1346.627898
	-1425.883314	0.637030	-1425.246284
**B1-TS5R**	-1425.873619	0.635781	-1425.237838
**B1-TS5R'**	-1425.882278	0.640732	-1425.241546
**B1-TS5S**	-1425.873743	0.640302	-1425.233441
**B1-TS5S'**	-1425.875020	0.639449	-1425.235571
**B2-TS5R**	-1386.542627	0.611556	-1385.931071
**B2-TS5R'**	-1386.542624	0.612113	-1385.930511
**B2-TS5S**	-1386.534284	0.609842	-1385.924442
**B2-TS5S'**	-1386.541759	0.610645	-1385.931114
**B3-TS5R**	-1347.204675	0.584444	-1346.620231
**B3-TS5S**	-1347.198071	0.579805	-1346.618266
**B3-TS5S'**	-1347.203036	0.583267	-1346.619769
**B4-TS5R**	-1309.083097	0.576092	-1308.507005
**B4-TS5S**	-1309.076694	0.574231	-1308.502463
**B4-TS5S'**	-1309.077042	0.574241	-1308.502801
**C1-cat**	-456.813045	0.211172	-456.601873
**C1-B5-TS1R**	-841.970279	0.340019	-841.630260
**C1-B5-TS1R'**	-841.964071	0.340638	-841.623433
**C1-B5-TS1S**	-841.965424	0.339246	-841.626178
**C1-B5-TS1S'**	-841.965241	0.340811	-841.624430
**C1-TS7R**	-689.495562	0.318523	-689.177039
**C1-TS7R'**	-689.479085	0.318854	-689.160231
**C1-TS7S**	-689.486910	0.318631	-689.168279
**C1-TS7S'**	-689.493512	0.319330	-689.174182
**C2-cat**	-614.089075	0.319620	-613.769455
**C2-B5-TS1R**	-999.274382	0.450166	-998.824216
**C2-B5-TS1R'**	-999.268147	0.450553	-998.817594
**C2-B5-TS1S**	-999.268879	0.448706	-998.820173
**C2-B5-TS1S'**	-999.268879	0.450072	-998.818807

**C2-TS7R**	-846.799575	0.428224	-846.371351
**C2-TS7R'**	-846.779938	0.428226	-846.351712
**C2-TS7S**	-846.798728	0.427476	-846.371252
**C2-TS7S'**	-846.785060	0.429679	-846.355381
**C3-cat**	-847.608520	0.447679	-847.160841
**C3-B5-TS1R**	-1232.840964	0.577666	-1232.263298
**C3-B5-TS1R'**	-1232.837176	0.578161	-1232.259015
**C3-B5-TS1S**	-1232.830248	0.577898	-1232.252350
**C3-B5-TS1S'**	-1232.838990	0.578514	-1232.260476
**C3-TS4R**	-1236.480861	0.646731	-1235.834130
**C3-TS4S**	-1236.475113	0.648786	-1235.826327
**C3-TS4S'**	-1236.475293	0.647694	-1235.827599
**C3-TS7R**	-1080.366849	0.555169	-1079.811680
**C3-TS7R'**	-1080.362580	0.556573	-1079.806007
**C3-TS7S**	-1080.358505	0.555561	-1079.802944
**C3-TS7S'**	-1080.367130	0.556355	-1079.810775
**C4-cat**	-692.706300	0.376133	-692.330167
**C4-B5-TS1R**	-1077.909206	0.505415	-1077.403791
**C4-B5-TS1R'**	-1077.905680	0.507321	-1077.398359
**C4-B5-TS1S**	-1077.905571	0.505276	-1077.400295
**C4-B5-TS1S'**	-1077.895731	0.507317	-1077.388414
**C4-TS7R**	-925.436796	0.483199	-924.953597
**C4-TS7R'**	-925.426570	0.483835	-924.942735
**C4-TS7S**	-925.435850	0.484634	-924.951216
**C4-TS7S'**	-925.417950	0.484798	-924.933152
**C5-B5-TS1R**	-1572.326283	0.551610	-1571.774673
**C5-B5-TS1R'**	-1572.323214	0.551331	-1571.771883
**C5-B5-TS1S**	-1572.318369	0.547767	-1571.770602
**C5-B5-TS1S'**	-1572.318013	0.550271	-1571.767742
**C5-TS7R**	-1380.506637	0.502357	-1380.004280
**C5-TS7R'**	-1380.501984	0.503117	-1379.998867

**C5-TS7S**	-1380.503759	0.500700	-1380.003059
**C5-TS7S'**	-1380.499820	0.502297	-1379.997523
**C5-cat**	-997.637074	0.403797	-997.233277
**C6-B5-TS1R**	-1382.917963	0.536119	-1382.381844
**C6-B5-TS1S**	-1382.910383	0.533165	-1382.377218
**C6-B5-TS1S'**	-1382.911083	0.533483	-1382.377600
**C6-TS4R**	-1386.555618	0.604641	-1385.950977
**C6-TS4S**	-1386.548394	0.602210	-1385.946184
**C6-TS7R**	-1230.441175	0.512751	-1229.928424
**C6-TS7R'**	-1230.427553	0.517180	-1229.910373
**C6-TS7S**	-1230.431257	0.512261	-1229.918996
**C6-TS7S'**	-1230.434924	0.510728	-1229.924196
**C5-cat**	-1312.215708	0.621185	-1311.594523
**C7-B5-TS1R**	-1697.566019	0.754582	-1696.811437
**C7-B5-TS1S**	-1697.558748	0.750837	-1696.807911
**C7-B5-TS1S'**	-1697.558748	0.752823	-1696.805925
**C7-TS4S**	-1701.207063	0.823949	-1700.383114
**C7-TS4R**	-1701.198811	0.820535	-1700.378276
**C7-TS4S**	-1545.089132	0.730221	-1544.358911
**C7-TS7R'**	-1545.076591	0.731927	-1544.344664
**C7-TS7S**	-1545.083214	0.729112	-1544.354102
**C7-TS7S'**	-1545.081023	0.730789	-1544.350234
**C5-cat**	-1469.503304	0.731234	-1468.772070
**C8-B5-TS1R**	-1854.882110	0.864687	-1854.017423
**C8-B5-TS1R'**	-1854.876725	0.864528	-1854.012197
**C8-B5-TS1S**	-1854.872421	0.860731	-1854.011690
**C8-B5-TS1S'**	-1854.873921	0.862265	-1854.011656
**C8-TS4S**	-1858.521230	0.933818	-1857.587412
**C8-TS4R**	-1858.515498	0.930256	-1857.585242
**C8-TS4S**	-1702.405384	0.840801	-1701.564583
**C8-TS7R'**	-1702.393557	0.841550	-1701.552007

**C8-TS7S**	-1702.399827	0.839627	-1701.560200
**C8-TS7S'**	-1702.398174	0.841988	-1701.556186
**C5-cat**	-1779.254382	0.876535	-1778.377847
**C9-B5-TS1R**	-2164.699169	1.010476	-2163.688693
**C9-B5-TS1R'**	-2164.697016	1.008983	-2163.688033
**C9-B5-TS1S**	-2164.693240	1.008290	-2163.684950
**C9-B5-TS1S'**	-2164.694697	1.009542	-2163.685155
**C9-TS4S**	-2168.343102	1.080290	-2167.262812
**C9-TS4R**	-2168.337363	1.077292	-2167.260071
**C9-TS7R**	-2012.227016	0.987066	-2011.239950
**C9-TS7R'**	-2012.213018	0.989778	-2011.223240
**C9-TS7S**	-2012.221607	0.986456	-2011.235151
**C9-TS7S'**	-2012.216663	0.986207	-2011.230456
**C5-cat**	-2189.153784	0.299510	-2188.854274
**C10-B5-TS1R**	-2574.208677	0.432028	-2573.776649
**C10-B5-TS1S**	-2574.199462	0.429227	-2573.770235
**C10-B5-TS1S'**	-2574.200564	0.430559	-2573.770005
**C10-TS7R**	-2421.731401	0.409433	-2421.321968
**C10-TS7R'**	-2421.723771	0.409824	-2421.313947
**C10-TS7S**	-2421.729185	0.407522	-2421.321663
**C10-TS7S'**	-2421.724301	0.408003	-2421.316298
**C5-cat**	-1833.122975	0.214724	-1832.908251
**C11-B5-TS1R**	-2218.177960	0.344966	-2217.832994
**C11-B5-TS1R'**	-2218.172707	0.344398	-2217.828309
**C11-B5-TS1S**	-2218.174445	0.341922	-2217.832523
**C11-B5-TS1S'**	-2218.170899	0.344398	-2217.826501
**C11-TS7R**	-2065.704482	0.321866	-2065.382616
**C11-TS7R'**	-2065.695110	0.322873	-2065.372237
**C11-TS7S**	-2065.699322	0.322555	-2065.376767
**C11-TS7S'**	-2065.694349	0.322089	-2065.372260
**C12-TS1R**	-802.644297	0.310982	-802.333315

**C12-TS1R'**	-802.641402	0.311518	-802.329884
**C12-TS1S**	-802.640371	0.310621	-802.329750
**C12-TS1S'**	-802.640836	0.311669	-802.329167
**C12-TS4R**	-806.281840	0.379633	-805.902207
**C12-TS4S**	-806.282279	0.380220	-805.902059
**C12-TS4S'**	-806.279599	0.380128	-805.899471
**C13-B5-TS1R**	-2189.512783	0.760912	-2188.751871
**C13-B5-TS1R'**	-2189.503301	0.761551	-2188.741750
**C13-B5-TS1S**	-2189.499192	0.757537	-2188.741655
**C13-B5-TS1S'**	-2189.497997	0.760376	-2188.737621
**C13-TS7R**	-1997.685170	0.712618	-1996.972552
**C13-TS7R'**	-1997.681556	0.713958	-1996.967598
**C13-TS7S**	-1997.685170	0.711921	-1996.973249
**C13-TS7S'**	-1997.679905	0.712257	-1996.967648
**C8-cat**	-1469.503304	0.731234	-1468.772070
**C8-TS6R**	-1741.734059	0.868271	-1740.865788
**C8-TS6R'**	-1741.726031	0.871901	-1740.854130
**C8-TS6S**	-1741.730937	0.866134	-1740.864803
**C8-TS6S'**	-1741.727184	0.867366	-1740.859818
**C8-TS4R**	-1858.521227	0.933826	-1857.587401
**C8-TS4R'**	-1858.517097	0.933948	-1857.583149
**C8-TS4S**	-1858.515498	0.930256	-1857.585242
**C8-TS4S'**	-1858.510339	0.930914	-1857.579425
**C8-TS7R**	-1702.405384	0.840801	-1701.564583
**C8-TS7R'**	-1702.393557	0.841550	-1701.552007
**C8-TS7S**	-1702.399827	0.839627	-1701.560200
**C8-TS7S'**	-1702.398174	0.841988	-1701.556186
**C8-B5-TS1R**	-1854.882110	0.864687	-1854.017423
**C8-B5-TS1R'**	-1854.876725	0.864528	-1854.012197
**C8-B5-TS1S**	-1854.872421	0.860731	-1854.011690
**C8-B5-TS1S'**	-1854.873921	0.862265	-1854.011656

**C8-TS4R**	-1781.064971	0.894751	-1780.170220
**C8-TS4R'**	-1781.040723	0.898791	-1780.141932
**C8-TS4S**	-1781.053943	0.894555	-1780.159388
**C8-TS4S'**	-1781.057455	0.894718	-1780.162737
**B5-TS1R**	-1456.691357	0.511367	-1456.179990
**B5-TS1R'**	-1456.729715	0.509979	-1456.219736
**B5-TS1S**	-1456.685486	0.509671	-1456.175815
**B5-TS1S'**	-1456.727135	0.511156	-1456.215979
**B5-C12-TS1R**	-994.449639	0.362219	-994.087420
**B5-C12-TS1R'**	-994.449997	0.360247	-994.089750
**B5-C12-TS1S**	-994.450125	0.361114	-994.089011
**B5-C12-TS1S'**	-994.450985	0.363095	-994.087890
**C12h-TS1R**	-763.302180	0.284741	-763.017439
**C12h-TS1S**	-763.298569	0.284651	-763.013918
**C12h-TS1S'**	-763.298215	0.285442	-763.012773
**h-TS7R**	-1073.107460	0.415850	-1072.691610
**h-TS7R'**	-1073.102471	0.416738	-1072.685733
**h-TS7S**	-1073.103762	0.414989	-1072.688773
**h-TS7S'**	-1073.101089	0.416449	-1072.684640

1pri

C	0.523382	0.208367	0.045934
C	0.395128	-0.656089	1.384324
H	-0.191385	-0.063881	2.094885
C	-0.130786	1.585361	0.201847
C	-0.798776	2.143671	-0.892125
C	-0.074269	2.321926	1.388356
C	-1.410662	3.389572	-0.798220
H	-0.828323	1.579097	-1.814050
C	-0.685068	3.571941	1.486102
H	0.447090	1.923427	2.250387
C	-1.361119	4.108793	0.395161
H	-1.922127	3.804217	-1.660173
H	-0.631474	4.123744	2.417907
H	-1.838360	5.079039	0.470657
C	1.976912	0.370550	-0.415237
C	2.814350	1.373260	0.082693
C	2.502750	-0.539151	-1.334840
C	4.146540	1.452558	-0.313252
H	2.426225	2.105957	0.778697
C	3.835109	-0.460948	-1.732747
H	1.846780	-1.293710	-1.744865
C	4.665234	0.532430	-1.221111
H	4.777850	2.239653	0.083920
H	4.223060	-1.177002	-2.448935
H	5.701820	0.595194	-1.531966
O	-0.181106	-0.520553	-0.940827
B	-0.826672	-1.733685	-0.412571
N	-0.325337	-1.870800	0.993076

C	1.687794	-1.136322	2.087649
H	2.551048	-0.510058	1.869647
H	1.529392	-1.120311	3.170966
C	1.851323	-2.584570	1.609948
H	2.329963	-2.602735	0.627225
H	2.444915	-3.194877	2.295022
C	0.391982	-3.041614	1.487656
H	0.261628	-3.891932	0.817143
H	0.018874	-3.347663	2.477585
C	-0.679447	-2.992350	-1.409123
H	-1.270015	-3.860723	-1.095520
H	-0.978535	-2.749648	-2.434855
H	0.365864	-3.311342	-1.463401
H	-3.459688	-1.891659	-1.973405
O	-2.291529	-1.420096	-0.336035
C	-2.854515	-0.705895	0.828332
C	-4.235600	-0.303680	0.349410
C	-3.975952	0.064255	-1.117281
C	-2.983410	-0.998361	-1.571383
H	-2.811394	-1.398430	1.661066
H	-2.226341	0.161599	1.026118
H	-4.632949	0.528866	0.931134
H	-4.931269	-1.143765	0.424911
H	-3.517345	1.053294	-1.175513
H	-4.878466	0.061443	-1.729733
H	-2.220443	-0.630795	-2.251191

2

C	0.179376	-0.202149	0.136388
C	-0.682570	-0.850331	-1.012402
H	-0.104189	-1.655487	-1.453172
C	1.642788	-0.614374	0.059597
C	2.377782	-0.751927	1.238516
C	2.287222	-0.797232	-1.164910
C	3.729355	-1.075106	1.191792
H	1.879355	-0.615598	2.188644
C	3.643631	-1.111770	-1.212110
H	1.738251	-0.700990	-2.094203
C	4.368520	-1.253600	-0.033399
H	4.285118	-1.189156	2.115287
H	4.128145	-1.254171	-2.170981
H	5.421717	-1.506188	-0.068124
C	0.070294	1.324809	0.222741
C	0.799708	2.139536	-0.649287
C	-0.769108	1.933976	1.157446
C	0.667671	3.523229	-0.609977
H	1.488994	1.692613	-1.354046
C	-0.895464	3.321196	1.203459
H	-1.311067	1.325283	1.867241
C	-0.184660	4.121270	0.315542
H	1.241023	4.135313	-1.296438
H	-1.548285	3.773690	1.941012
H	-0.282315	5.199845	0.351514
O	-0.386316	-0.750143	1.349728
B	-1.576575	-1.361201	1.145882
N	-1.864256	-1.489131	-0.316049
C	-1.295712	0.057344	-2.082372
H	-0.675831	0.920756	-2.314864
H	-1.426444	-0.525696	-2.997563
C	-2.667157	0.438111	-1.508739
H	-2.561671	1.234896	-0.769331
H	-3.367182	0.776025	-2.274373
C	-3.121021	-0.863371	-0.852656
H	-3.845312	-0.736231	-0.048866
H	-3.539663	-1.553061	-1.585829
B	-1.858818	-3.140683	-0.528569
H	-2.938911	-3.534271	-0.164231
H	-1.657487	-3.296882	-1.710957
H	-0.947712	-3.549085	0.162236
C	-2.535692	-1.756247	2.303110
H	-3.110446	-2.652610	2.069917
H	-1.986748	-1.910307	3.234232
H	-3.251840	-0.944396	2.484423

2’

C	0.634517	0.211149	-0.031196
C	0.068359	-0.474660	1.264207
H	-0.620704	0.219727	1.737662
C	0.603202	1.735488	0.079612
C	0.457843	2.489548	-1.087975
C	0.744724	2.402445	1.297263
C	0.440777	3.878559	-1.036394
H	0.347359	1.969666	-2.030564
C	0.732674	3.796380	1.350104
H	0.860506	1.842793	2.217667
C	0.577814	4.538809	0.184505
H	0.323369	4.449198	-1.950811
H	0.840792	4.297816	2.305065
H	0.565283	5.621762	0.224635
C	2.059188	-0.223477	-0.409451

C	3.173962	0.259948	0.282984
C	2.267156	-1.104411	-1.471044
C	4.456656	-0.169658	-0.041440
H	3.046081	0.994157	1.068154
C	3.551081	-1.531412	-1.802316
H	1.414263	-1.437204	-2.044773
C	4.650757	-1.075700	-1.081736
H	5.306370	0.213527	0.511962
H	3.689963	-2.217508	-2.630257
H	5.649991	-1.408556	-1.337373
O	-0.262820	-0.191347	-1.062595
B	-1.200519	-1.205837	-0.673241
N	-0.744221	-1.635111	0.777044
C	1.035111	-1.059230	2.291808
H	1.955913	-0.485777	2.375567
H	0.543430	-1.056769	3.263952
C	1.288375	-2.508528	1.825025
H	2.288155	-2.614331	1.401608
H	1.198246	-3.202918	2.659725
C	0.229715	-2.789192	0.743402
H	0.702882	-2.839462	-0.233831
H	-0.329672	-3.706760	0.908093
B	-1.937559	-1.998305	1.827401
H	-2.600813	-2.873133	1.309611
H	-1.420918	-2.392895	2.847943
H	-2.580608	-0.985189	2.024025
C	-1.443372	-2.377560	-1.732635
H	-2.098093	-3.170207	-1.360027
H	-1.876533	-1.982559	-2.657631
H	-0.499604	-2.850874	-2.012186
H	-4.050411	-1.679732	0.224791
O	-2.596084	-0.432134	-0.555847
C	-2.704756	0.983812	-0.171831
C	-4.170899	1.155545	0.188485
C	-4.873919	0.126185	-0.705295
C	-3.924186	-1.058494	-0.658858
H	-2.030629	1.164833	0.656999
H	-2.389572	1.571759	-1.032056
H	-4.509130	2.177652	0.014439
H	-4.325281	0.908082	1.240363
H	-4.969914	0.504205	-1.726773
H	-5.866528	-0.143991	-0.343446
H	-3.921708	-1.666254	-1.558463

3

C	-1.275084	0.222751	0.126109
C	-0.865747	-0.674203	-1.114685
H	0.071163	-0.297472	-1.515289
C	-0.486385	1.533931	0.138345
C	-0.118456	2.088647	1.366283
C	-0.139798	2.208780	-1.033849
C	0.589781	3.284343	1.420157
H	-0.375499	1.555300	2.271211
C	0.564867	3.411040	-0.982104
H	-0.404016	1.797002	-2.000602
C	0.934455	3.952284	0.245399
H	0.876009	3.695757	2.381459
H	0.829364	3.917456	-1.903342
H	1.484490	4.885391	0.288255
C	-2.773979	0.543015	0.213521
C	-3.365722	1.492777	-0.624809
C	-3.578340	-0.115821	1.144044
C	-4.732199	1.746238	-0.563170
H	-2.756085	2.053129	-1.321974
C	-4.945667	0.141447	1.212737
H	-3.116731	-0.819629	1.821503
C	-5.530918	1.066189	0.353471
H	-5.171204	2.483553	-1.225603
H	-5.552349	-0.380676	1.944088
H	-6.594978	1.265794	0.404963
O	-0.919330	-0.549657	1.269346
B	-0.165841	-1.701506	0.931057
N	-0.577669	-2.030631	-0.541871
C	-1.894449	-0.925580	-2.213653
H	-2.512888	-0.054222	-2.419662
H	-1.361242	-1.190445	-3.125998
C	-2.723624	-2.127685	-1.711503
H	-3.703279	-1.804972	-1.356977
H	-2.874152	-2.853827	-2.509966
C	-1.916116	-2.733461	-0.547125
H	-2.421399	-2.553239	0.400729
H	-1.735492	-3.800263	-0.650505
B	0.449866	-2.883854	-1.473531
H	0.696996	-3.903554	-0.866693
H	-0.115117	-3.125428	-2.515582
H	1.439795	-2.206422	-1.669102
O	1.373144	-1.046336	0.714961
C	2.531666	-1.459999	0.537288
C	-0.147125	-2.850878	2.026037
H	0.141032	-3.824910	1.624244

H	0.524380	-2.609930	2.855751
H	-1.146699	-2.961655	2.456475
C	3.542521	-0.458033	0.153362
C	4.875651	-0.817036	-0.095664
C	3.154441	0.885099	0.027207
C	5.801409	0.149892	-0.464462
H	5.191745	-1.848099	-0.010675
C	4.083472	1.847003	-0.339455
C	5.406620	1.481627	-0.586221
H	3.770037	2.879025	-0.433267
H	6.131443	2.233918	-0.875350
C	2.936403	-2.883585	0.751288
H	3.680625	-2.925936	1.552594
H	2.083063	-3.500395	0.998436
H	3.391665	-3.272876	-0.160214
H	2.127795	1.157200	0.220722
H	6.828525	-0.133105	-0.659890

TS1R

C	-1.050678	-0.965683	-0.038126
C	-0.475485	-0.603937	1.393570
H	0.552689	-0.952082	1.446977
C	-0.164250	-2.011010	-0.718314
C	0.052855	-1.926297	-2.094500
C	0.430389	-3.055740	-0.007623
C	0.859445	-2.856329	-2.742496
H	-0.398673	-1.109270	-2.640225
C	1.232399	-3.993234	-0.655648
H	0.289269	-3.138071	1.063675
C	1.453428	-3.894520	-2.026078
H	1.027546	-2.769252	-3.809944
H	1.690763	-4.792867	-0.085110
H	2.081574	-4.618493	-2.532162
C	-2.506550	-1.459673	-0.031821
C	-2.841560	-2.756466	0.368379
C	-3.531878	-0.602190	-0.434568
C	-4.169314	-3.170987	0.407239
H	-2.063018	-3.458544	0.637573
C	-4.861087	-1.017400	-0.403075
H	-3.272814	0.386482	-0.785649
C	-5.187511	-2.299855	0.027187
H	-4.406452	-4.179878	0.725169
H	-5.641606	-0.335243	-0.720833
H	-6.221739	-2.622959	0.053573
O	-1.011949	0.249547	-0.768955
B	-0.272967	1.269911	-0.101787
N	-0.433435	0.901846	1.443038
C	-1.279339	-1.016851	2.622280
H	-1.759143	-1.985429	2.498926
H	-0.605241	-1.077665	3.475789
C	-2.302604	0.121024	2.824799
H	-3.296613	-0.184679	2.497482
H	-2.364806	0.403729	3.875928
C	-1.802662	1.293205	1.958048
H	-2.458714	1.458028	1.106250
H	-1.710874	2.224149	2.509861
B	0.692224	1.411974	2.449678
H	0.621893	2.606802	2.572475
H	0.643438	0.807754	3.490864
H	1.793020	1.122668	1.932846
O	1.243749	0.884525	-0.361150
C	2.296559	1.317329	0.206063
C	3.423467	0.354182	0.272406
C	4.631166	0.670061	0.908783
C	3.272418	-0.901725	-0.330858
C	5.665389	-0.256003	0.944279
H	4.760037	1.627423	1.395394
C	4.311836	-1.821809	-0.298650
C	5.508687	-1.502712	0.339345
H	4.179691	-2.787023	-0.771359
H	6.318328	-2.222824	0.367751
C	2.571554	2.789133	0.313748
H	3.068789	3.080838	-0.619155
H	1.652987	3.355792	0.406895
H	3.231105	3.022119	1.145612
H	2.343394	-1.139769	-0.826388
H	6.592922	-0.008020	1.446156
C	-0.645342	2.727785	-0.622784
C	-0.860847	2.883929	-2.001065
C	-0.779178	3.868503	0.180362
C	-1.182993	4.119775	-2.554621
H	-0.780993	2.015433	-2.645116
C	-1.105933	5.109382	-0.364140
H	-0.614207	3.795632	1.248370
C	-1.306380	5.240181	-1.735435
H	-1.341816	4.209743	-3.623847
H	-1.203999	5.973584	0.283913
H	-1.559368	6.204544	-2.161684

TS1Rpri

C	-0.213874	1.049117	0.096562
C	-0.209145	0.347309	1.488577
H	-1.103526	-0.268243	1.549164
C	-1.615426	1.367244	-0.412112
C	-1.773037	1.647526	-1.773238
C	-2.718426	1.481290	0.430437
C	-3.008128	2.029758	-2.279094
H	-0.913484	1.565949	-2.426359
C	-3.959881	1.867357	-0.075458
H	-2.626672	1.268312	1.487610
C	-4.109062	2.144971	-1.428858
H	-3.113831	2.245831	-3.336285
H	-4.808571	1.948199	0.593854
H	-5.072349	2.449819	-1.821213
C	0.600742	2.353344	0.042636
C	0.067492	3.543364	0.550985
C	1.876539	2.384297	-0.519560
C	0.811880	4.717712	0.546802
H	-0.944160	3.559373	0.936105
C	2.621565	3.561895	-0.531212
H	2.291134	1.486832	-0.952358
C	2.098333	4.730904	0.011204
H	0.381466	5.626476	0.951720
H	3.613040	3.559836	-0.969529
H	2.678242	5.646462	0.004217
O	0.390997	0.072916	-0.762662
B	1.144130	-0.891416	-0.072197
N	0.963171	-0.598372	1.456197
C	-0.000863	1.192934	2.739844
H	-0.484208	2.165865	2.670781
H	-0.424829	0.661346	3.590611
C	1.531002	1.310462	2.888499
H	1.879175	2.285112	2.545742
H	1.828962	1.191269	3.929911
C	2.133973	0.201807	2.005454
H	2.709991	0.621812	1.184942
H	2.773735	-0.483379	2.554182
B	0.702397	-1.828770	2.509256
H	1.555091	-2.663215	2.332063
H	0.748439	-1.358205	3.621078
H	-0.411689	-2.267078	2.290169
O	0.364715	-2.518627	-0.259465
C	-0.656565	-3.076013	0.166936
C	-2.000948	-2.492036	-0.021605
C	-3.049842	-2.738121	0.874776
C	-2.241058	-1.745975	-1.182035
C	-4.317647	-2.237694	0.614106
H	-2.860922	-3.282798	1.790615
C	-3.522349	-1.288521	-1.459608
C	-4.557757	-1.526915	-0.562117
H	-3.705114	-0.717926	-2.359376
H	-5.550511	-1.146834	-0.771577
C	-0.534368	-4.479805	0.681734
H	-0.813167	-5.159821	-0.131654
H	0.493664	-4.665699	0.981733
H	-1.212590	-4.667530	1.512760
H	-1.419609	-1.540282	-1.854103
H	-5.120172	-2.405057	1.322474
C	2.552769	-1.276358	-0.678410
C	2.901858	-0.815075	-1.954717
C	3.479109	-2.082481	-0.001831
C	4.129649	-1.131945	-2.532580
H	2.194314	-0.201491	-2.501205
C	4.710057	-2.397943	-0.569003
H	3.227243	-2.472687	0.976796
C	5.040665	-1.921798	-1.836835
H	4.374523	-0.763445	-3.522861
H	5.412717	-3.019109	-0.024169
H	5.999601	-2.168132	-2.279168

TS1S

C	1.846244	0.135618	-0.009082
C	1.253543	0.294637	1.452269
H	1.011631	1.341339	1.609853
C	2.196098	1.504541	-0.597437
C	2.000258	1.728351	-1.960753
C	2.733470	2.532069	0.180902
C	2.326483	2.953689	-2.531626
H	1.573892	0.935312	-2.559594
C	3.065578	3.759897	-0.389894
H	2.895278	2.387109	1.242942
C	2.860958	3.975842	-1.748983
H	2.161776	3.112789	-3.591425
H	3.480908	4.545868	0.230689
H	3.114486	4.930818	-2.194475
C	3.066261	-0.792821	-0.094289
C	4.341229	-0.365313	0.287507
C	2.912600	-2.102138	-0.553367
C	5.426768	-1.234839	0.247245
H	4.496029	0.657541	0.606029

C	3.999531	-2.971902	-0.601085
H	1.936743	-2.425182	-0.886809
C	5.259767	-2.545687	-0.193035
H	6.406274	-0.883473	0.551040
H	3.858585	-3.984207	-0.962883
H	6.105703	-3.222329	-0.229012
O	0.801046	-0.458208	-0.767922
B	-0.439269	-0.437717	-0.087363
N	-0.052179	-0.456981	1.445946
C	2.042484	-0.283694	2.623360
H	3.117257	-0.188569	2.483420
H	1.767404	0.255587	3.528908
C	1.592716	-1.757124	2.720191
H	2.362390	-2.426846	2.335786
H	1.392195	-2.032762	3.755676
C	0.322003	-1.867463	1.856448
H	0.510174	-2.453608	0.958958
H	-0.522257	-2.304079	2.382264
B	-1.068336	0.157692	2.529625
H	-2.063489	-0.515956	2.577218
H	-0.510186	0.244528	3.596559
H	-1.353699	1.298314	2.162427
O	-0.919288	1.132818	-0.262621
C	-1.829534	1.788348	0.303461
C	-3.238340	1.337563	0.309102
C	-4.097103	1.611158	1.379736
C	-3.732707	0.697255	-0.831970
C	-5.427442	1.224477	1.317069
H	-3.703816	2.070496	2.277367
C	-5.072551	0.332855	-0.899461
H	-3.068196	0.489149	-1.657950
C	-5.918022	0.588157	0.175136
H	-6.082584	1.409671	2.159716
H	-5.444566	-0.169124	-1.783715
H	-6.958452	0.288024	0.129486
C	-1.535076	3.229324	0.602311
H	-2.125895	3.597076	1.439618
H	-0.470385	3.368018	0.780652
H	-1.821916	3.805677	-0.285432
C	-1.498415	-1.465134	-0.663542
C	-2.524493	-2.054955	0.085191
C	-1.425333	-1.784990	-2.028581
C	-3.448559	-2.914621	-0.503364
H	-2.620592	-1.818576	1.136637
C	-2.352802	-2.633045	-2.627521
H	-0.626885	-1.356172	-2.623567
C	-3.372108	-3.199980	-1.863847
H	-4.236247	-3.352816	0.099623
H	-2.278288	-2.858164	-3.686030
H	-4.095095	-3.863796	-2.325110

TS1Spri

C	-1.624449	-0.546231	-0.009268
C	-0.869103	-0.655327	1.357872
H	-0.343734	-1.606135	1.367788
C	-1.959585	-1.928090	-0.571348
C	-2.006071	-2.094604	-1.957660
C	-2.240334	-3.023123	0.248143
C	-2.316864	-3.331612	-2.510925
H	-1.776211	-1.247943	-2.590399
C	-2.555819	-4.263186	-0.305646
H	-2.207697	-2.922648	1.326446
C	-2.593518	-4.421750	-1.686995
H	-2.342610	-3.446449	-3.588637
H	-2.767355	-5.103725	0.345409
H	-2.835087	-5.385829	-2.119193
C	-2.911603	0.291642	0.070254
C	-4.104073	-0.260178	0.550682
C	-2.910240	1.631821	-0.316640
C	-5.249691	0.518386	0.681981
H	-4.148154	-1.310207	0.809713
C	-4.057142	2.412203	-0.190774
H	-2.010123	2.066018	-0.722336
C	-5.229946	1.862660	0.317652
H	-6.161601	0.070027	1.059640
H	-4.029204	3.452140	-0.495927
H	-6.122643	2.469319	0.416366
O	-0.687661	0.077461	-0.877151
B	0.434122	0.694199	-0.232267
N	0.179935	0.424080	1.336888
C	-1.644256	-0.450820	2.656275
H	-2.666956	-0.817359	2.590992
H	-1.137824	-0.996662	3.451962
C	-1.587568	1.065717	2.924118
H	-2.530267	1.541218	2.652614
H	-1.400784	1.268112	3.979035
C	-0.455088	1.612731	2.036546
H	-0.842180	2.297499	1.287779
H	0.320731	2.128037	2.597243
B	1.451236	-0.053266	2.173608

H	2.303395	0.795262	2.162729
H	1.116918	-0.436848	3.266482
H	1.912150	-1.050065	1.562118
O	1.801445	-0.009940	-0.563483
C	2.294272	-1.115974	-0.172383
C	3.766074	-1.136193	-0.001239
C	4.466450	-2.327510	0.221514
C	4.468390	0.073890	-0.072557
C	5.847693	-2.308562	0.373049
H	3.939243	-3.270332	0.283250
C	5.847750	0.088060	0.077341
C	6.540016	-1.101508	0.302024
H	6.385674	1.026809	0.021034
H	7.617144	-1.087986	0.421620
C	1.554974	-2.405299	-0.409234
H	0.490233	-2.233968	-0.513468
H	1.918434	-2.806057	-1.362225
H	1.744056	-3.144026	0.367126
H	3.915502	0.986269	-0.248065
H	6.383719	-3.233776	0.547211
C	0.733809	2.198713	-0.690425
C	0.134931	2.716554	-1.845098
C	1.649993	3.019643	-0.015807
C	0.419280	4.003212	-2.301537
H	-0.559527	2.093558	-2.397562
C	1.935544	4.308100	-0.458792
H	2.146787	2.644249	0.872191
C	1.317709	4.806214	-1.605123
H	-0.058466	4.376751	-3.200957
H	2.641556	4.924777	0.087281
H	1.539056	5.809083	-1.953104

4R

C	-1.229692	0.086027	0.150719
C	-0.712831	-1.143413	-0.682143
H	0.366818	-1.026141	-0.739120
C	-0.697134	1.424076	-0.378835
C	-0.975342	2.566886	0.378695
C	0.063426	1.566606	-1.537490
C	-0.492882	3.812791	-0.000634
H	-1.568158	2.466078	1.279595
C	0.545058	2.816669	-1.925714
H	0.308869	0.706550	-2.143128
C	0.273482	3.943582	-1.158188
H	-0.715079	4.683550	0.605609
H	1.140440	2.903282	-2.827614
H	0.651604	4.914029	-1.457827
C	-2.756263	0.103607	0.209535
C	-3.511296	0.810084	-0.730148
C	-3.422185	-0.645670	1.182100
C	-4.901717	0.742727	-0.719629
H	-3.011559	1.421941	-1.470310
C	-4.812260	-0.713288	1.193482
H	-2.839218	-1.170007	1.925546
C	-5.557775	-0.025981	0.238408
H	-5.471566	1.296363	-1.457050
H	-5.314106	-1.301612	1.953399
H	-6.640364	-0.078107	0.248848
O	-0.795086	-0.072922	1.513730
B	0.500807	0.015912	1.951345
N	-0.995158	-2.434013	-0.039501
C	-1.347862	-1.293305	-2.077963
H	-1.655802	-0.345162	-2.515577
H	-0.604944	-1.739640	-2.744560
C	-2.536902	-2.252033	-1.884800
H	-3.452563	-1.692111	-1.701577
H	-2.699588	-2.873196	-2.767057
C	-2.169933	-3.095573	-0.652085
H	-2.998463	-3.141367	0.056260
H	-1.899009	-4.117723	-0.921416
B	-0.198639	-3.036848	0.923468
H	-0.521409	-4.105666	1.346998
H	0.800352	-2.503825	1.288988
H	3.068935	0.040225	2.338861
O	1.476565	0.259950	1.031531
C	2.852618	0.475067	1.358944
C	0.751071	-0.152920	3.495762
H	1.399399	-1.011748	3.699445
H	1.245747	0.723548	3.928399
H	-0.187542	-0.308314	4.029544
C	3.705199	-0.226337	0.325590
C	4.789349	-1.011381	0.713438
C	3.429442	-0.075201	-1.036086
C	5.591932	-1.635084	-0.240459
H	5.004331	-1.142799	1.768900
C	4.225094	-0.700557	-1.989566
C	5.310497	-1.482045	-1.594486
H	3.999984	-0.580105	-3.043403
H	5.929181	-1.970883	-2.338103
C	3.110659	1.980454	1.400501

H	2.843883	2.429833	0.441834
H	2.502258	2.447370	2.178722
H	4.164370	2.183634	1.604985
H	2.584570	0.530146	-1.337865
H	6.429552	-2.246327	0.075333

TS2R

C	-1.508850	0.021506	-0.093046
C	-1.425998	-0.897905	-1.384281
H	-1.236612	-0.258734	-2.244784
C	-0.619574	1.276470	-0.131706
C	-0.300965	1.876993	1.091533
C	-0.199971	1.901865	-1.303392
C	0.426106	3.058104	1.141624
H	-0.625938	1.399957	2.006970
C	0.529235	3.091660	-1.257855
H	-0.418198	1.470088	-2.269963
C	0.846167	3.674906	-0.037625
H	0.665575	3.501722	2.101375
H	0.853175	3.553415	-2.183416
H	1.416087	4.595837	-0.001134
C	-2.942029	0.493031	0.147859
C	-3.523359	1.432114	-0.708993
C	-3.702338	-0.028551	1.193775
C	-4.845315	1.825948	-0.535026
H	-2.937759	1.866542	-1.509934
C	-5.024306	0.374670	1.374137
H	-3.249572	-0.750576	1.858165
C	-5.602992	1.297988	0.508991
H	-5.281915	2.553594	-1.209602
H	-5.601864	-0.036565	2.194456
H	-6.631379	1.609965	0.649330
O	-1.138493	-0.820654	0.993055
B	0.056653	-1.515996	0.955782
N	-0.328022	-1.865097	-1.212381
C	-2.669006	-1.776279	-1.653494
H	-3.588637	-1.353916	-1.258007
H	-2.791906	-1.877951	-2.735902
C	-2.313082	-3.137637	-1.047478
H	-2.473180	-3.120107	0.032587
H	-2.895222	-3.958161	-1.470286
C	-0.824910	-3.247323	-1.363461
H	-0.281344	-3.923544	-0.703369
H	-0.679540	-3.596768	-2.393432
B	1.062232	-1.502039	-1.384756
H	1.854371	-2.393390	-1.355003
H	1.341978	-0.464191	-1.885647
H	2.721381	-2.062103	1.337664
O	1.243362	-0.837526	0.559013
C	2.500539	-0.994230	1.238875
C	0.124861	-2.863913	1.769471
H	0.928390	-3.523815	1.431815
H	0.304148	-2.664402	2.831833
H	-0.819016	-3.408419	1.709677
C	3.577844	-0.347069	0.399617
C	4.821478	-0.957465	0.249893
C	3.351079	0.897190	-0.191726
C	5.833034	-0.332156	-0.476805
H	4.999188	-1.930726	0.696069
C	4.356936	1.519063	-0.921825
C	5.602332	0.908298	-1.064746
H	4.165219	2.481173	-1.383364
H	6.385373	1.393302	-1.636326
C	2.422518	-0.348009	2.622531
H	2.195867	0.714399	2.518319
H	1.649699	-0.815139	3.236475
H	3.381511	-0.450443	3.135421
H	2.381021	1.362863	-0.095193
H	6.795254	-0.818217	-0.590888

5R

C	-1.497818	0.069423	-0.035761
C	-1.352604	-0.888104	-1.277133
H	-0.960629	-0.344174	-2.130348
C	-0.601041	1.317828	-0.101222
C	-0.256880	1.923051	1.111878
C	-0.187097	1.924666	-1.285421
C	0.487138	3.095220	1.139988
H	-0.573911	1.451188	2.032148
C	0.561272	3.102973	-1.261503
H	-0.429207	1.490103	-2.245999
C	0.901512	3.693402	-0.050567
H	0.746502	3.544608	2.091879
H	0.881159	3.550654	-2.195341
H	1.485844	4.605797	-0.030648
C	-2.939775	0.537185	0.158008
C	-3.505009	1.475772	-0.709474
C	-3.723067	0.001935	1.179482
C	-4.835020	1.856822	-0.569831
H	-2.901691	1.918354	-1.492692

C	-5.053267	0.391901	1.325682
H	-3.276724	-0.716837	1.852101
C	-5.615934	1.315367	0.449900
H	-5.260017	2.584271	-1.252033
H	-5.649649	-0.028613	2.127621
H	-6.650709	1.617136	0.563361
O	-1.120993	-0.730488	1.079711
B	-0.144311	-1.691920	0.720191
N	-0.335546	-1.918305	-0.860167
C	-2.603857	-1.699211	-1.696561
H	-3.525977	-1.283145	-1.299634
H	-2.682379	-1.687074	-2.786723
C	-2.336813	-3.129491	-1.209204
H	-2.638183	-3.238189	-0.164104
H	-2.862404	-3.884683	-1.795617
C	-0.822742	-3.237010	-1.329665
H	-0.370726	-4.028344	-0.735365
H	-0.519380	-3.375186	-2.371976
B	1.195471	-1.574412	-1.078439
H	1.849531	-2.581739	-1.161248
H	1.400014	-0.746074	-1.918534
H	2.734076	-2.103987	1.296316
O	1.262147	-1.001764	0.368538
C	2.471627	-1.050542	1.150818
C	-0.015257	-2.955762	1.668580
H	0.816200	-3.612634	1.395921
H	0.126556	-2.658023	2.710834
H	-0.930616	-3.554872	1.641768
C	3.577615	-0.366269	0.381788
C	4.830297	-0.963701	0.258596
C	3.361587	0.894072	-0.180503
C	5.863385	-0.307628	-0.408203
H	4.997520	-1.949697	0.679459
C	4.388446	1.545453	-0.852744
C	5.643853	0.948697	-0.965539
H	4.206169	2.519946	-1.291340
H	6.443971	1.457707	-1.490705
C	2.222539	-0.376370	2.495280
H	1.966888	0.672587	2.344103
H	1.411953	-0.861029	3.039988
H	3.131175	-0.429883	3.098619
H	2.386133	1.353528	-0.105623
H	6.833760	-0.781962	-0.499785

TS3R

C	-1.244546	0.180246	-0.111181
C	-1.467567	-0.849798	-1.291071
H	-0.750634	-0.601773	-2.067980
C	-0.725183	1.525168	-0.637807
C	-0.151197	2.404201	0.288319
C	-0.822274	1.927122	-1.968741
C	0.350551	3.634037	-0.113811
H	-0.093059	2.102703	1.326594
C	-0.328824	3.169037	-2.373307
H	-1.280296	1.285101	-2.709493
C	0.266646	4.021040	-1.451834
H	0.806463	4.293692	0.615207
H	-0.408376	3.461909	-3.413709
H	0.658482	4.980354	-1.768320
C	-2.502884	0.463242	0.729150
C	-3.478740	1.356477	0.275966
C	-2.686371	-0.148704	1.969050
C	-4.637314	1.580610	1.012220
H	-3.327108	1.897296	-0.649476
C	-3.842113	0.079417	2.711889
H	-1.911793	-0.794074	2.355766
C	-4.829247	0.934281	2.231092
H	-5.383761	2.272175	0.639061
H	-3.965498	-0.408939	3.671718
H	-5.729485	1.111033	2.807843
O	-0.271537	-0.361790	0.781993
B	0.487389	-1.502720	0.666305
N	-1.206555	-2.240213	-0.893763
C	-2.904875	-0.935133	-1.832078
H	-3.433220	0.015086	-1.848786
H	-2.849454	-1.305180	-2.858353
C	-3.593348	-1.981194	-0.919241
H	-4.237467	-1.498092	-0.184948
H	-4.208948	-2.663050	-1.507448
C	-2.430389	-2.731137	-0.220727
H	-2.400962	-2.492724	0.841717
H	-2.485632	-3.812492	-0.324677
B	-0.441023	-3.100911	-1.726781
H	0.231094	-2.623988	-2.580777
H	-0.613818	-4.272626	-1.637178
H	1.460100	0.544868	-0.383198
O	1.609796	-1.486643	-0.218781
C	2.200760	-0.191757	-0.665408
C	0.426035	-2.610319	1.769099
H	0.582994	-3.610466	1.367807

H	1.241422	-2.438697	2.482440
H	-0.514850	-2.586632	2.321309
C	3.448672	0.080372	0.137247
C	3.352909	0.923166	1.247610
C	4.686047	-0.484408	-0.179841
C	4.470748	1.202504	2.028291
H	2.394145	1.363840	1.496010
C	5.803902	-0.208661	0.600959
C	5.700326	0.635236	1.704889
H	6.757675	-0.657854	0.349812
H	6.573902	0.848032	2.310268
C	2.357767	-0.161608	-2.172438
H	3.108561	-0.861592	-2.530212
H	1.410305	-0.399712	-2.656647
H	2.647797	0.851531	-2.460393
H	4.771854	-1.156139	-1.022746
H	4.382192	1.860396	2.885163
H	1.483845	-3.673440	-0.742466
H	3.022772	-3.169897	0.463962
H	3.062081	-2.710688	-1.504289
B	2.397945	-2.908693	-0.528066

6R

C	-1.359858	0.168977	-0.021271
C	-1.318903	-0.656407	-1.360701
H	-0.739601	-0.089605	-2.081631
C	-1.134305	1.659272	-0.284703
C	-0.572942	2.438097	0.730602
C	-1.496761	2.277851	-1.481586
C	-0.348941	3.796428	0.541106
H	-0.304671	1.961645	1.664721
C	-1.282510	3.643143	-1.670324
H	-1.943376	1.702917	-2.283902
C	-0.700733	4.405022	-0.663292
H	0.099244	4.383091	1.334831
H	-1.564652	4.105345	-2.609238
H	-0.525086	5.463906	-0.812051
C	-2.654554	0.012008	0.790416
C	-3.839559	0.634925	0.386333
C	-2.664400	-0.742059	1.964141
C	-5.016749	0.462281	1.107024
H	-3.841936	1.278789	-0.484286
C	-3.840583	-0.910241	2.691698
H	-1.737746	-1.180655	2.306855
C	-5.024216	-0.319165	2.260500
H	-5.925953	0.949520	0.774018
H	-3.827525	-1.500211	3.601144
H	-5.939808	-0.450815	2.825157
O	-0.276391	-0.344836	0.750070
B	0.468244	-1.373884	0.090822
N	-0.525131	-1.894300	-1.044617
C	-2.617728	-1.190562	-1.959714
H	-3.451696	-0.502255	-1.844642
H	-2.459560	-1.350635	-3.027824
C	-2.864097	-2.541429	-1.246894
H	-3.687622	-2.470074	-0.535892
H	-3.109791	-3.319754	-1.970154
C	-1.552540	-2.856446	-0.503858
H	-1.667628	-2.674080	0.561289
H	-1.194514	-3.871483	-0.647529
B	0.206509	-2.458312	-2.328336
H	0.066355	-1.768688	-3.289123
H	0.005922	-3.626881	-2.496103
H	1.529739	0.932296	0.361370
O	1.582396	-0.694184	-0.828809
C	2.228400	0.552927	-0.376738
C	1.117181	-2.467397	1.042922
H	1.638761	-3.253508	0.491357
H	1.853643	-2.004670	1.705126
H	0.367477	-2.947495	1.678764
C	3.542219	0.272932	0.314301
C	3.577066	0.249438	1.710075
C	4.722924	0.036719	-0.393751
C	4.764315	-0.007686	2.389916
H	2.662956	0.431043	2.264800
C	5.910464	-0.224879	0.281940
C	5.934499	-0.247702	1.674916
H	6.818296	-0.413137	-0.279323
H	6.860992	-0.450876	2.199405
C	2.295458	1.536836	-1.531787
H	2.945729	1.192948	-2.335178
H	1.296144	1.704760	-1.934176
H	2.677482	2.489111	-1.158076
H	4.715543	0.045051	-1.476280
H	4.775093	-0.021715	3.473604
H	1.468510	-2.595702	-2.021142
H	3.143563	-2.274328	-1.305776
H	2.569990	-1.050245	-2.828549
B	2.292187	-1.597535	-1.807878

s-B5-TS1R

C	1.190815	-0.015146	1.079250
H	0.222423	0.430901	1.303113
O	1.657431	0.122361	-1.256418
B	0.791697	-0.988210	-1.092689
N	0.936599	-1.362338	0.457819
C	1.983413	-0.309586	2.353728
H	2.633228	0.519569	2.632213
H	1.290497	-0.474915	3.177668
C	2.765112	-1.611035	2.046141
H	3.840719	-1.435516	1.989209
H	2.590642	-2.353081	2.825227
C	2.230951	-2.101460	0.687389
H	2.919750	-1.846646	-0.117041
H	2.036485	-3.170434	0.654421
B	-0.250981	-2.157302	1.151140
H	-0.307377	-3.270677	0.691991
H	-0.153033	-2.124143	2.352938
H	-1.308436	-1.550786	0.878240
O	-0.698903	-0.355386	-1.091921
C	-1.791714	-0.900225	-0.750975
C	0.983416	-2.156059	-2.160597
H	0.657702	-3.137864	-1.806889
H	0.446321	-1.939160	-3.088921
H	2.041764	-2.241751	-2.422535
C	-2.830887	0.012071	-0.204223
C	-4.133258	-0.425341	0.066216
C	-2.498867	1.351773	0.035587
C	-5.081091	0.458887	0.567902
H	-4.412641	-1.455703	-0.105352
C	-3.446772	2.232777	0.536407
C	-4.740728	1.788577	0.805305
H	-3.178443	3.266904	0.716911
H	-5.480774	2.475743	1.198480
C	-2.206109	-2.236599	-1.301478
H	-2.737358	-2.044825	-2.241380
H	-1.342451	-2.856414	-1.504045
H	-2.870965	-2.766378	-0.624254
H	-1.495201	1.686104	-0.182129
H	-6.085191	0.109244	0.775737
C	1.894506	0.823878	-0.031097
C	1.347390	2.266114	-0.137841
C	3.415556	1.047374	0.071103
C	2.484913	3.124814	-0.748936
H	1.068305	2.633863	0.854487
H	0.451987	2.256007	-0.758241
C	3.663705	2.149196	-0.964533
H	3.979210	0.134220	-0.121038
H	3.678852	1.413620	1.068435
H	2.181409	3.606340	-1.679877
H	2.772922	3.920744	-0.056660
H	3.611538	1.713050	-1.963694
H	4.638034	2.630040	-0.853363

s-B5-TS1Rpri

C	1.179954	-0.072038	1.093718
H	0.196638	-0.175637	1.551247
O	0.558360	0.506101	-1.088012
B	0.834816	-0.873493	-1.171657
N	1.402561	-1.295577	0.245821
C	2.290760	-0.072992	2.141240
H	2.578213	0.941966	2.421060
H	1.938220	-0.575618	3.040564
C	3.455025	-0.869966	1.506906
H	4.314407	-0.230042	1.298294
H	3.785960	-1.659648	2.180120
C	2.903889	-1.458605	0.195988
H	3.302928	-0.920504	-0.661843
H	3.127294	-2.515046	0.068158
B	0.786763	-2.608831	0.994228
H	0.834309	-3.537216	0.221347
H	1.451222	-2.797482	1.988560
H	-0.361548	-2.335876	1.308748
O	-0.694222	-1.768912	-1.274824
C	-1.702648	-1.802581	-0.559189
C	1.499999	-1.451455	-2.487869
H	1.654711	-2.532242	-2.438617
H	0.853504	-1.246616	-3.346524
H	2.463250	-0.976525	-2.698127
C	-2.355985	-0.566957	-0.071275
C	-2.974111	-0.500586	1.183046
C	-2.426148	0.529275	-0.939855
C	-3.639824	0.656453	1.568345
H	-2.896310	-1.335755	1.867444
C	-3.132955	1.666090	-0.567338
C	-3.734111	1.733914	0.687488
H	-3.197374	2.506141	-1.248172
H	-4.270697	2.627860	0.983104
C	-2.393064	-3.116863	-0.359519
H	-3.229491	-3.168598	-1.066660

H	-1.693205	-3.926601	-0.549269
H	-2.810083	-3.200012	0.643496
H	-1.925166	0.478710	-1.895771
H	-4.092685	0.716375	2.550607
C	1.110824	1.097775	0.103611
C	0.231234	2.254445	0.580675
C	2.407559	1.833079	-0.286493
C	0.505714	3.398369	-0.414654
H	0.559219	2.533489	1.588065
H	-0.815316	1.965879	0.642352
C	1.900091	3.083168	-1.032010
H	3.069353	1.220228	-0.898286
H	2.952299	2.121261	0.617034
H	-0.258872	3.412453	-1.193571
H	0.483729	4.373585	0.076188
H	1.793996	2.855151	-2.093312
H	2.595351	3.920318	-0.944021

s-B5-TS1S

C	1.704608	0.721462	-0.782963
H	1.260331	0.298648	-1.682515
O	1.463189	-0.812170	1.030489
B	0.246522	-0.126329	0.925284
N	0.581273	1.183492	0.102002
C	2.515734	1.979251	-1.093036
H	3.565427	1.750561	-1.280297
H	2.117889	2.450720	-1.990715
C	2.302086	2.902441	0.132286
H	3.223765	3.058108	0.695794
H	1.938423	3.878669	-0.186465
C	1.258609	2.184926	1.006933
H	1.743529	1.643431	1.819423
H	0.504708	2.846689	1.425518
B	-0.603071	1.918699	-0.678768
H	-1.434726	2.262957	0.122015
H	-0.163209	2.826928	-1.343426
H	-1.092012	1.100489	-1.463763
O	-0.570619	-1.016508	-0.318192
C	-1.573438	-0.706531	-1.003557
C	-0.643786	0.006402	2.222901
H	-1.575004	0.548672	2.054113
H	-0.884626	-0.976019	2.637894
H	-0.085358	0.542992	2.997029
C	-2.893337	-0.419650	-0.384738
C	-3.803129	0.479028	-0.950790
C	-3.264204	-1.161499	0.740365
C	-5.058683	0.647005	-0.384773
H	-3.502002	1.082512	-1.797464
C	-4.530642	-1.004762	1.294070
H	-2.560536	-1.864105	1.165215
C	-5.426700	-0.097742	0.736565
H	-5.749615	1.364584	-0.810672
H	-4.813042	-1.586757	2.163159
H	-6.409274	0.033216	1.174610
C	-1.557572	-1.080510	-2.462239
H	-2.235392	-0.463283	-3.049245
H	-0.545009	-1.020623	-2.856678
H	-1.904629	-2.118243	-2.532721
C	2.402279	-0.428210	0.011579
C	2.704182	-1.639207	-0.887803
C	3.756816	-0.132363	0.725562
C	3.772192	-2.419337	-0.120806
H	3.120423	-1.283894	-1.839754
H	1.788323	-2.192582	-1.096105
C	4.705547	-1.300653	0.369387
H	3.551764	-0.106686	1.795973
H	4.187971	0.829087	0.450269
H	3.309237	-2.926110	0.730578
H	4.283436	-3.170620	-0.726908
H	5.332656	-1.600526	1.211381
H	5.376474	-1.005986	-0.444864

s-B5-TS1Spri

C	1.674098	0.376421	-1.031348
H	1.103074	-0.233984	-1.731197
O	1.317652	-0.922505	0.864933
B	0.436792	0.197407	1.101203
N	0.678515	1.149889	-0.206256
C	2.541653	1.402218	-1.773985
H	3.599152	1.143162	-1.697181
H	2.289370	1.419120	-2.833667
C	2.227811	2.769605	-1.117533
H	3.131640	3.315776	-0.840166
H	1.659099	3.393450	-1.805974
C	1.387691	2.436814	0.119000
H	2.024450	2.288588	0.988501
H	0.648234	3.196736	0.362424
B	-0.619093	1.487795	-1.073191
H	-1.365422	2.188095	-0.436835
H	-0.298131	1.920315	-2.153779

H	-1.203650	0.403553	-1.280328
O	-1.064167	-0.238866	1.021456
C	-1.686327	-0.759469	0.043594
C	0.530993	0.905957	2.530646
H	-0.111703	1.788387	2.614603
H	0.205191	0.192737	3.294900
H	1.548415	1.205985	2.795557
C	-3.139398	-0.487137	-0.016086
C	-3.989318	-1.214342	-0.857869
C	-3.673633	0.519355	0.799043
C	-5.351766	-0.941037	-0.881754
H	-3.593429	-1.993165	-1.495633
C	-5.033632	0.790643	0.771394
C	-5.875324	0.061656	-0.068693
H	-5.440426	1.573163	1.400583
H	-6.937484	0.276252	-0.090735
C	-1.114668	-1.938052	-0.696398
H	-0.037809	-1.955383	-0.579600
H	-1.519739	-2.839316	-0.221326
H	-1.400205	-1.940855	-1.746202
H	-3.003902	1.078463	1.436711
H	-6.004245	-1.508175	-1.534637
C	2.365294	-0.581353	-0.041833
C	2.893806	-1.852879	-0.733251
C	3.602637	-0.029852	0.745188
C	4.059194	-2.328081	0.136862
H	3.268909	-1.588491	-1.729483
H	2.096858	-2.587076	-0.858517
C	4.763519	-1.008068	0.479732
H	3.334603	-0.043201	1.801769
H	3.865331	0.995889	0.485257
H	3.671819	-2.795907	1.047050
H	4.709030	-3.048781	-0.364453
H	5.446807	-1.087294	1.327902
H	5.352413	-0.676703	-0.383169

TS4R

C	1.415636	0.238102	-0.169986
C	0.959132	-0.730516	1.005050
H	0.005959	-0.379239	1.387779
C	0.512502	1.475593	-0.231942
C	0.211622	2.027212	-1.478520
C	-0.008830	2.083621	0.911534
C	-0.604749	3.148190	-1.580832
H	0.608073	1.548202	-2.363337
C	-0.825274	3.209920	0.812414
H	0.210328	1.681507	1.893963
C	-1.130147	3.744478	-0.435179
H	-0.834405	3.557321	-2.558369
H	-1.223270	3.665304	1.712294
H	-1.769796	4.615692	-0.515110
C	2.883018	0.672401	-0.076554
C	3.291923	1.680420	0.801609
C	3.848328	0.044718	-0.864850
C	4.634352	2.029139	0.912006
H	2.558519	2.209317	1.396911
C	5.192177	0.396583	-0.760462
H	3.530860	-0.708438	-1.571901
C	5.592958	1.384776	0.133572
H	4.929605	2.812243	1.601068
H	5.925694	-0.101465	-1.384570
H	6.638348	1.658824	0.215716
O	1.255097	-0.522625	-1.362217
B	0.406141	-1.644904	-1.152366
N	0.703383	-2.057659	0.349455
C	1.936703	-1.028045	2.140469
H	2.557996	-0.170154	2.389981
H	1.363083	-1.301521	3.025781
C	2.769222	-2.233643	1.652575
H	3.777242	-1.924862	1.373893
H	2.851004	-2.988537	2.434649
C	2.028178	-2.781614	0.418917
H	2.590618	-2.568571	-0.488339
H	1.831275	-3.849766	0.465785
B	-0.406695	-2.908534	1.133424
H	-0.569868	-3.951897	0.547098
H	-0.062087	-3.061540	2.281088
H	-1.448248	-2.244582	1.136766
O	-1.058183	-0.974706	-0.972299
C	-2.158267	-1.421868	-0.574002
C	0.449737	-2.761622	-2.280758
H	0.067462	-3.728145	-1.942871
H	-0.127059	-2.456490	-3.159211
H	1.477774	-2.920245	-2.618927
C	-2.734866	-2.706598	-1.076249
H	-3.453509	-2.440662	-1.862640
H	-1.970028	-3.351325	-1.492920
H	-3.275487	-3.229409	-0.290898
C	-3.027757	-0.418443	0.140021
C	-3.560910	0.605036	-0.896723

C	-4.170307	-1.014471	0.968527
H	-2.351511	0.134886	0.798501
C	-4.372206	1.699681	-0.195648
H	-4.194305	0.087618	-1.627900
H	-2.716319	1.041034	-1.432609
C	-4.961611	0.095298	1.669616
H	-4.849033	-1.575043	0.315265
H	-3.767106	-1.720674	1.699360
C	-5.500793	1.116810	0.661867
H	-4.775523	2.388465	-0.943998
H	-3.694570	2.284458	0.435629
H	-5.781782	-0.341593	2.246257
H	-4.307824	0.603648	2.388687
H	-6.032200	1.919107	1.182173
H	-6.234742	0.625019	0.010768

TS4Rpri

C	0.987179	0.293404	-0.084834
C	0.686892	-0.632966	1.139046
H	-0.327920	-0.423996	1.466342
C	0.230326	1.618466	-0.011845
C	-0.013887	2.305333	-1.204845
C	-0.166606	2.199472	1.192450
C	-0.644460	3.543330	-1.192196
H	0.289740	1.848544	-2.137583
C	-0.802126	3.441927	1.206486
H	0.011850	1.690852	2.132036
C	-1.042423	4.117590	0.015498
H	-0.830212	4.061744	-2.125962
H	-1.108149	3.877141	2.150906
H	-1.534965	5.082827	0.025255
C	2.479452	0.617822	-0.276251
C	3.130777	1.540588	0.549641
C	3.208152	0.014277	-1.300518
C	4.487511	1.803868	0.394502
H	2.571923	2.078573	1.304646
C	4.566319	0.280720	-1.462407
H	2.698389	-0.650957	-1.980489
C	5.215486	1.165919	-0.607891
H	4.973296	2.518790	1.048736
H	5.113746	-0.201331	-2.264530
H	6.272058	1.372940	-0.732038
O	0.502060	-0.445280	-1.200183
B	0.355713	-1.855730	-0.964037
N	0.690469	-2.031976	0.590827
C	1.642051	-0.657383	2.330687
H	2.106591	0.308951	2.513769
H	1.075408	-0.932433	3.219742
C	2.679712	-1.749393	2.000110
H	3.635125	-1.305173	1.718329
H	2.848670	-2.395034	2.861782
C	2.100860	-2.536093	0.810863
H	2.691918	-2.353655	-0.081248
H	2.052147	-3.609103	0.980000
B	-0.317377	-2.961647	1.440200
H	-0.342857	-4.061352	0.945340
H	0.021972	-2.952704	2.599456
H	-1.435722	-2.438907	1.355429
O	-1.179484	-2.327768	-1.113539
C	-2.209944	-2.075225	-0.443150
C	1.056799	-2.847666	-1.999089
H	0.891916	-3.893682	-1.725349
H	0.614790	-2.698888	-2.989756
H	2.133285	-2.698107	-2.104129
C	-3.225099	-3.177045	-0.366018
H	-3.970309	-3.018039	-1.153556
H	-2.728735	-4.130373	-0.528705
H	-3.742494	-3.179175	0.591139
C	-2.604800	-0.663712	-0.103705
C	-3.315690	-0.080105	-1.357906
C	-3.487101	-0.507715	1.140987
H	-1.695672	-0.078763	0.015491
C	-3.700918	1.383893	-1.119784
H	-4.215995	-0.665604	-1.577721
H	-2.647026	-0.163901	-2.217424
C	-3.854400	0.962410	1.367424
H	-4.408863	-1.086770	1.016608
H	-2.966842	-0.916964	2.010838
C	-4.553926	1.556557	0.140544
H	-4.235570	1.761665	-1.996886
H	-2.789690	1.978993	-1.023662
H	-4.494383	1.049252	2.250415
H	-2.944836	1.535154	1.575830
H	-4.767219	2.616953	0.303843
H	-5.521760	1.057985	-0.000504

TS4S

C	1.646222	0.084094	-0.120363

C	0.991028	-0.573579	1.169704
H	0.500871	0.208841	1.740594
C	1.615750	1.613183	-0.021185
C	1.416248	2.360819	-1.182825
C	1.802522	2.289016	1.186202
C	1.390922	3.750110	-1.136772
H	1.259186	1.834561	-2.114408
C	1.779990	3.682585	1.235030
H	1.961428	1.736588	2.105240
C	1.570722	4.418759	0.073247
H	1.227481	4.314045	-2.048379
H	1.924316	4.189081	2.182731
H	1.549022	5.501839	0.108947
C	3.081333	-0.383331	-0.395236
C	4.164077	0.113125	0.336724
C	3.328510	-1.333537	-1.386812
C	5.454683	-0.352483	0.106230
H	4.004443	0.879898	1.083937
C	4.620883	-1.796548	-1.623902
H	2.498896	-1.689947	-1.980351
C	5.689024	-1.314486	-0.873674
H	6.279425	0.044550	0.687078
H	4.791369	-2.532606	-2.401627
H	6.694768	-1.674462	-1.057092
O	0.831718	-0.338270	-1.207607
B	-0.428761	-0.825980	-0.761357
N	-0.089586	-1.479040	0.648758
C	1.853884	-1.459048	2.066240
H	2.880435	-1.105564	2.137555
H	1.424252	-1.458805	3.067725
C	1.761899	-2.870624	1.447566
H	2.685785	-3.131633	0.930491
H	1.582822	-3.620196	2.218642
C	0.597668	-2.809521	0.440934
H	0.975294	-2.849795	-0.578926
H	-0.137245	-3.599560	0.573830
B	-1.280716	-1.652555	1.701434
H	-2.095638	-2.417314	1.242588
H	-0.841466	-2.006929	2.768338
H	-1.796697	-0.535476	1.859644
O	-1.199445	0.501932	-0.275031
C	-2.261613	0.655003	0.379620
C	-1.230633	-1.690757	-1.827991
H	-2.016330	-2.308141	-1.386494
H	-1.689630	-1.053725	-2.589757
H	-0.548103	-2.364151	-2.354174
C	-2.335440	1.899187	1.216036
H	-2.817650	1.702086	2.172205
H	-1.335265	2.302476	1.362646
H	-2.943275	2.639261	0.683772
C	-3.520693	-0.089471	0.025586
C	-4.570729	-0.162363	1.139241
C	-4.112805	0.580624	-1.243414
H	-3.231742	-1.103254	-0.244086
C	-5.807122	-0.931819	0.659705
H	-4.872991	0.848672	1.435651
H	-4.133463	-0.646095	2.015473
C	-5.352353	-0.187368	-1.715902
H	-4.390564	1.617001	-1.017736
H	-3.356742	0.610862	-2.030347
C	-6.402474	-0.305058	-0.606005
H	-6.555318	-0.963823	1.456723
H	-5.522989	-1.970639	0.454067
H	-5.777327	0.309141	-2.593329
H	-5.046829	-1.190079	-2.036981
H	-7.255600	-0.893550	-0.955876
H	-6.787635	0.694416	-0.366806

TS4Spri

C	1.577077	0.225038	-0.094081
C	0.880762	-0.385317	1.168106
H	0.168287	0.349857	1.532059
C	1.636204	1.750914	-0.025622
C	1.612759	2.474013	-1.221126
C	1.740147	2.445943	1.180452
C	1.680626	3.862381	-1.208891
H	1.522444	1.931814	-2.152905
C	1.812253	3.838524	1.193325
H	1.757572	1.910877	2.122386
C	1.780740	4.551155	-0.000654
H	1.655598	4.409696	-2.144437
H	1.888820	4.363259	2.138831
H	1.833108	5.633655	0.008404
C	2.998234	-0.307348	-0.344956
C	4.083332	0.122584	0.426183
C	3.232645	-1.228662	-1.365366
C	5.355508	-0.401245	0.221862
H	3.943080	0.888936	1.177581
C	4.507318	-1.749844	-1.577097

H	2.411003	-1.517493	-2.003172
C	5.572006	-1.349326	-0.776073
H	6.181361	-0.058150	0.834590
H	4.666388	-2.465706	-2.375691
H	6.563672	-1.755450	-0.938259
O	0.726846	-0.148209	-1.170524
B	-0.179884	-1.224799	-0.883827
N	0.083597	-1.555873	0.662751
C	1.727185	-0.934485	2.314040
H	2.647996	-0.374170	2.460808
H	1.143974	-0.872699	3.232398
C	1.996359	-2.407375	1.948046
H	3.013089	-2.532239	1.573825
H	1.874941	-3.052776	2.818100
C	0.988548	-2.757801	0.837867
H	1.510375	-2.953653	-0.093755
H	0.365397	-3.617954	1.071346
B	-1.222845	-1.763501	1.573645
H	-1.883095	-2.666701	1.124332
H	-0.893887	-1.910887	2.725697
H	-1.876777	-0.708119	1.493077
O	-1.711000	-0.746249	-0.942271
C	-2.363009	0.028660	-0.194661
C	-0.209558	-2.439675	-1.917791
H	-0.914329	-3.216137	-1.606817
H	-0.545445	-2.068031	-2.891416
H	0.761222	-2.914768	-2.076422
C	-1.875384	1.408967	0.131679
H	-0.824972	1.533912	-0.102872
H	-2.445751	2.095934	-0.505560
H	-2.080258	1.679899	1.165766
C	-3.828892	-0.296544	-0.054734
C	-4.528377	0.344234	1.149523
C	-4.545019	0.089644	-1.374113
H	-3.874841	-1.384827	0.038625
C	-6.004179	-0.068151	1.198640
H	-4.467651	1.436496	1.077887
H	-4.016447	0.049188	2.068984
C	-6.023796	-0.309657	-1.320271
H	-4.465924	1.172592	-1.529816
H	-4.043135	-0.397979	-2.212458
C	-6.730966	0.293787	-0.101660
H	-6.493733	0.407646	2.053193
H	-6.068523	-1.150434	1.363092
H	-6.521086	0.001958	-2.243613
H	-6.095379	-1.402941	-1.277025
H	-7.770421	-0.044756	-0.062522
H	-6.760123	1.385919	-0.206001

TS5R

C	1.056183	0.195462	-0.157147
C	0.440991	-0.605462	1.066168
H	-0.367370	-0.012931	1.482037
C	0.601959	1.655820	-0.107630
C	0.236430	2.299037	-1.290251
C	0.540679	2.369576	1.091774
C	-0.189597	3.623845	-1.273001
H	0.270543	1.738327	-2.214115
C	0.121587	3.697833	1.110605
H	0.797616	1.887483	2.028017
C	-0.248538	4.329708	-0.072947
H	-0.477734	4.106494	-2.200210
H	0.074175	4.233034	2.052118
H	-0.582320	5.360779	-0.060185
C	2.590244	0.131994	-0.248069
C	3.412044	0.886517	0.594924
C	3.196452	-0.698478	-1.192277
C	4.797767	0.781556	0.524707
H	2.974446	1.577097	1.303508
C	4.583420	-0.800958	-1.269116
H	2.567329	-1.248318	-1.877152
C	5.391446	-0.068904	-0.404711
H	5.413770	1.374028	1.191588
H	5.031247	-1.451423	-2.012058
H	6.470809	-0.148347	-0.462594
O	0.527017	-0.434313	-1.314348
B	-0.494441	-1.379874	-1.021387
N	-0.175102	-1.838686	0.467343
C	1.381276	-1.118596	2.153921
H	2.204097	-0.434527	2.350067
H	0.812725	-1.239099	3.075493
C	1.871529	-2.491280	1.643608
H	2.906490	-2.436244	1.304532
H	1.813575	-3.240265	2.433521
C	0.950390	-2.849576	0.462319
H	1.490135	-2.767377	-0.478792
H	0.518085	-3.844304	0.531889
B	-1.381799	-2.406140	1.359121
H	-1.755387	-3.446623	0.875529

H	-1.026971	-2.518268	2.506852
H	-2.301520	-1.573544	1.330995
O	-1.813560	-0.476833	-0.785513
C	-2.951546	-0.799600	-0.363114
C	-0.686877	-2.501051	-2.138675
H	-1.105928	-3.435625	-1.757184
H	-1.330296	-2.151458	-2.951726
H	0.280556	-2.743192	-2.587543
C	-3.644691	-2.055790	-0.799338
H	-4.311087	-1.781060	-1.626371
H	-2.942866	-2.802441	-1.145303
H	-4.250686	-2.470050	0.001251
C	-3.782058	0.330249	0.243671
C	-4.944452	-0.197091	1.097006
H	-5.471336	0.648515	1.544601
H	-5.669970	-0.762030	0.508523
H	-4.577668	-0.834248	1.904782
C	-4.336434	1.122663	-0.970341
H	-3.519055	1.519583	-1.574379
H	-4.978568	0.505678	-1.603489
H	-4.933363	1.959792	-0.601437
C	-2.898313	1.265455	1.079219
H	-2.493902	0.737405	1.944899
H	-2.071725	1.661101	0.493754
H	-3.497945	2.104529	1.439574

TS5S

C	1.089032	0.200588	-0.078573
C	0.365448	-0.374294	1.189710
H	-0.290311	0.405451	1.565028
C	1.236824	1.720155	0.007347
C	1.215226	2.464904	-1.174478
C	1.419669	2.386039	1.220643
C	1.363149	3.846948	-1.141332
H	1.063055	1.946129	-2.111540
C	1.572347	3.771413	1.254410
H	1.435801	1.832763	2.152112
C	1.543041	4.506502	0.073825
H	1.338627	4.411998	-2.066260
H	1.709418	4.273395	2.205394
H	1.658364	5.583841	0.098962
C	2.477105	-0.402805	-0.359716
C	3.596779	-0.046530	0.399988
C	2.651289	-1.299397	-1.414286
C	4.840990	-0.616425	0.149884
H	3.508949	0.703133	1.175804
C	3.897417	-1.867163	-1.671087
H	1.805934	-1.530822	-2.044601
C	4.995559	-1.538596	-0.882683
H	5.693403	-0.328207	0.754415
H	4.008088	-2.562177	-2.495801
H	5.965277	-1.980133	-1.080908
O	0.207824	-0.112069	-1.148779
B	-0.759459	-1.136957	-0.865757
N	-0.514081	-1.483683	0.682699
C	1.189093	-0.978236	2.332884
H	2.200897	-0.582675	2.359978
H	0.710255	-0.736448	3.281158
C	1.177179	-2.507498	2.100478
H	2.184186	-2.900341	1.953220
H	0.735895	-3.017468	2.956322
C	0.335880	-2.722332	0.837351
H	0.984373	-2.816872	-0.029382
H	-0.318030	-3.589888	0.882044
B	-1.815571	-1.631454	1.607836
H	-2.435637	-2.599023	1.253216
H	-1.496345	-1.643369	2.771250
H	-2.510424	-0.611373	1.426746
O	-2.253314	-0.570363	-0.953409
C	-2.871644	0.230153	-0.203411
C	-0.842351	-2.360492	-1.887513
H	-1.605025	-3.084913	-1.586862
H	-1.126246	-1.982212	-2.875012
H	0.098402	-2.901500	-2.009902
C	-2.265468	1.541294	0.217681
H	-2.439331	1.749449	1.271163
H	-1.208885	1.581952	-0.013495
H	-2.761807	2.328099	-0.359767
C	-4.393400	0.087976	-0.178371
C	-4.898702	0.835713	-1.441798
H	-5.983725	0.726468	-1.503085
H	-4.671509	1.903454	-1.409271
H	-4.457755	0.410396	-2.345144
C	-5.003770	0.725477	1.078328
H	-6.086877	0.584682	1.063168
H	-4.611492	0.253527	1.981444
H	-4.812216	1.799057	1.133752
C	-4.807861	-1.385602	-0.280554
H	-4.478445	-1.941025	0.596414

H	-5.896313	-1.449697	-0.353704
H	-4.369892	-1.855102	-1.161002

TS5Spri

C	1.086685	0.205661	-0.081628
C	0.361546	-0.370649	1.183160
H	-0.299174	0.405369	1.557585
C	1.232648	1.725350	0.001073
C	1.233800	2.463749	-1.185008
C	1.391104	2.397744	1.213973
C	1.378257	3.846135	-1.156296
H	1.101742	1.939589	-2.122135
C	1.540721	3.783721	1.243418
H	1.390636	1.849326	2.148398
C	1.532484	4.512503	0.058779
H	1.371002	4.406323	-2.084483
H	1.658549	4.290876	2.194249
H	1.644500	5.590277	0.080454
C	2.475901	-0.398895	-0.353744
C	3.591075	-0.039084	0.410837
C	2.654881	-1.303369	-1.400625
C	4.835185	-0.614843	0.174417
H	3.499786	0.716993	1.180148
C	3.901179	-1.876764	-1.643951
H	1.813510	-1.535817	-2.035933
C	4.994299	-1.545917	-0.849519
H	5.684281	-0.324147	0.782470
H	4.016007	-2.577635	-2.463134
H	5.964092	-1.991888	-1.037184
O	0.209584	-0.109586	-1.154676
B	-0.758994	-1.133833	-0.873625
N	-0.511519	-1.484310	0.675110
C	1.183300	-0.971117	2.326721
H	2.178617	-0.538902	2.388621
H	0.673071	-0.771121	3.268190
C	1.230900	-2.492166	2.056197
H	2.247543	-2.828296	1.847855
H	0.862198	-3.043900	2.920679
C	0.339833	-2.724033	0.827931
H	0.953974	-2.845226	-0.059716
H	-0.318142	-3.585210	0.915881
B	-1.810812	-1.636881	1.599658
H	-2.431741	-2.602791	1.243374
H	-1.491924	-1.652393	2.763031
H	-2.506797	-0.615305	1.420716
O	-2.253700	-0.567270	-0.955798
C	-2.872316	0.225854	-0.196873
C	-0.845689	-2.352178	-1.901022
H	-1.604745	-3.079674	-1.598773
H	-1.137998	-1.968303	-2.883875
H	0.095051	-2.890770	-2.034951
C	-2.270086	1.540318	0.220732
H	-1.214259	1.584328	-0.013632
H	-2.770454	2.324041	-0.357524
H	-2.441734	1.750778	1.274151
C	-4.393827	0.077054	-0.165870
C	-4.802181	-1.398565	-0.262342
H	-4.364453	-1.868391	-1.142802
H	-4.467445	-1.949906	0.614994
H	-5.890526	-1.467780	-0.332513
C	-4.908308	0.818206	-1.429199
H	-5.992667	0.700329	-1.487226
H	-4.689669	1.887805	-1.399480
H	-4.466507	0.394468	-2.332818
C	-5.001693	0.715725	1.091363
H	-4.814513	1.790360	1.141911
H	-6.084145	0.569782	1.081784
H	-4.603266	0.248768	1.994399

TS6R

C	0.863147	0.194119	-0.153501
C	0.318321	-0.610000	1.101471
H	-0.475929	-0.025863	1.554949
C	0.377411	1.644373	-0.096999
C	-0.039323	2.272373	-1.270811
C	0.342060	2.365204	1.099014
C	-0.489771	3.588888	-1.247959
H	-0.024615	1.706438	-2.192038
C	-0.101710	3.685407	1.123468
H	0.641440	1.895647	2.029030
C	-0.522680	4.301980	-0.051076
H	-0.817275	4.059597	-2.168223
H	-0.127811	4.226235	2.062605
H	-0.875314	5.326697	-0.033840
C	2.392990	0.165282	-0.306362
C	3.229657	0.948957	0.494370
C	2.980197	-0.662154	-1.265042
C	4.613498	0.876346	0.368604

H	2.804161	1.636587	1.213222
C	4.365034	-0.732266	-1.397476
H	2.337283	-1.235177	-1.917339
C	5.189403	0.029415	-0.575166
H	5.241531	1.491101	1.003314
H	4.798054	-1.380771	-2.150840
H	6.267131	-0.024745	-0.676451
O	0.302633	-0.458587	-1.283344
B	-0.704391	-1.401604	-0.937650
N	-0.311757	-1.853095	0.537542
C	1.317621	-1.107156	2.143356
H	2.142652	-0.413909	2.291827
H	0.798522	-1.225787	3.093818
C	1.794364	-2.479380	1.618496
H	2.813356	-2.420058	1.234682
H	1.775017	-3.224758	2.413706
C	0.825320	-2.849132	0.480038
H	1.320137	-2.757332	-0.484634
H	0.410365	-3.849799	0.567043
B	-1.465931	-2.429909	1.490455
H	-1.830337	-3.492579	1.048523
H	-1.065636	-2.500371	2.625908
H	-2.415979	-1.627635	1.475119
O	-2.005528	-0.499814	-0.635633
C	-3.121457	-0.842348	-0.173189
C	-0.959206	-2.526545	-2.037387
H	-1.383144	-3.449557	-1.635033
H	-1.626718	-2.169065	-2.827346
H	-0.014884	-2.790273	-2.521796
C	-3.831146	-2.093809	-0.599570
H	-4.247475	-1.904682	-1.595921
H	-3.159148	-2.940543	-0.661922
H	-4.647212	-2.323061	0.083523
C	-3.944921	0.240936	0.488962
H	-4.627620	-0.255536	1.183975
C	-3.095040	1.265575	1.235613
H	-2.522511	0.786024	2.030942
H	-2.397816	1.763910	0.563050
H	-3.740077	2.022360	1.687710
C	-4.787921	0.921545	-0.616059
H	-5.409177	1.698717	-0.166889
H	-4.132604	1.391070	-1.353241
H	-5.446408	0.217891	-1.129209

TS6Rpri

C	0.675872	0.248545	-0.089813
C	0.161351	-0.571115	1.143554
H	-0.725556	-0.070774	1.520644
C	0.277687	1.721709	0.013960
C	0.045161	2.437537	-1.163259
C	0.147833	2.379302	1.238553
C	-0.323861	3.776938	-1.115172
H	0.136629	1.922052	-2.109816
C	-0.219594	3.723606	1.288008
H	0.318984	1.847309	2.166696
C	-0.459667	4.426101	0.111521
H	-0.507665	4.316592	-2.037359
H	-0.321165	4.217170	2.247762
H	-0.749080	5.469864	0.148405
C	2.194080	0.156925	-0.311229
C	3.090022	0.878786	0.484673
C	2.708939	-0.649505	-1.326150
C	4.463857	0.753817	0.305550
H	2.716444	1.563008	1.235804
C	4.084335	-0.771718	-1.511786
H	2.022019	-1.163572	-1.981288
C	4.968553	-0.080663	-0.689451
H	5.140353	1.319205	0.936295
H	4.462251	-1.404470	-2.306930
H	6.038698	-0.175841	-0.832081
O	-0.006036	-0.324857	-1.198445
B	-0.588230	-1.614190	-0.955099
N	-0.287153	-1.895117	0.592805
C	1.110880	-0.899225	2.294742
H	1.872308	-0.135865	2.438391
H	0.527601	-0.969326	3.212303
C	1.724885	-2.270422	1.946854
H	2.768888	-2.165826	1.648454
H	1.685886	-2.939305	2.806549
C	0.899621	-2.817429	0.769769
H	1.495765	-2.811309	-0.137905
H	0.524690	-3.825721	0.929677
B	-1.517431	-2.455536	1.467099
H	-1.906433	-3.490327	0.985989
H	-1.178217	-2.541347	2.622987
H	-2.416425	-1.601305	1.388355
O	-2.196109	-1.578044	-1.061957
C	-3.057227	-0.994881	-0.357289
C	-0.255693	-2.767729	-2.005859

H	-0.716153	-3.717605	-1.719396
H	-0.667411	-2.489993	-2.981593
H	0.812522	-2.945005	-2.149744
C	-4.387627	-1.677274	-0.247119
H	-5.020814	-1.321948	-1.068451
H	-4.249056	-2.751125	-0.345786
H	-4.886489	-1.442762	0.690976
C	-2.935524	0.474461	-0.029708
H	-1.881346	0.700371	0.078428
C	-3.683844	0.926540	1.222842
H	-3.396002	0.330362	2.091016
H	-3.438678	1.970761	1.428081
H	-4.767693	0.857065	1.099275
C	-3.400091	1.250140	-1.284875
H	-3.225030	2.314433	-1.119041
H	-2.828678	0.944114	-2.161816
H	-4.465299	1.102089	-1.481193

TS6S

C	-1.003382	0.131669	0.121362
C	-0.329089	-0.604950	-1.114027
H	0.277653	0.116954	-1.651832
C	-0.820301	1.648422	0.005695
C	-0.622825	2.396544	1.167226
C	-0.865622	2.314790	-1.220647
C	-0.462018	3.776177	1.103287
H	-0.576057	1.876660	2.114353
C	-0.707710	3.698696	-1.287456
H	-1.018897	1.760948	-2.139793
C	-0.501902	4.434645	-0.124872
H	-0.302543	4.340440	2.015357
H	-0.743633	4.197444	-2.249448
H	-0.374933	5.509926	-0.174165
C	-2.491268	-0.196002	0.302860
C	-2.890497	-1.111620	1.277327
C	-3.473303	0.394982	-0.497879
C	-4.233042	-1.450873	1.429534
H	-2.138738	-1.538592	1.925472
C	-4.814066	0.053052	-0.351955
H	-3.195459	1.139388	-1.233010
C	-5.200451	-0.876786	0.610559
H	-4.521691	-2.162217	2.195239
H	-5.558531	0.521798	-0.985404
H	-6.245178	-1.140360	0.727942
O	-0.306341	-0.347150	1.265609
B	0.919264	-0.976335	0.913714
N	0.615526	-1.607157	-0.512615
C	-1.212650	-1.409153	-2.065541
H	-2.193599	-0.958664	-2.201097
H	-0.722575	-1.454859	-3.037732
C	-1.296781	-2.820455	-1.443625
H	-2.278812	-2.996166	-1.003245
H	-1.123348	-3.586630	-2.199380
C	-0.215602	-2.858957	-0.347347
H	-0.674270	-2.844449	0.639410
H	0.444522	-3.720174	-0.414074
B	1.849319	-1.914073	-1.482307
H	2.553020	-2.746761	-0.962491
H	1.445595	-2.245118	-2.570387
H	2.478201	-0.854875	-1.621101
O	1.867169	0.261892	0.478933
C	2.975427	0.305246	-0.108061
C	1.551543	-1.898846	2.042197
H	0.773227	-2.509797	2.508458
H	2.318134	-2.582604	1.670603
H	1.994671	-1.296074	2.840365
C	3.223429	1.530563	-0.939521
H	3.879166	2.205538	-0.378222
H	3.728045	1.280306	-1.871434
H	2.279735	2.036002	-1.135521
C	4.137392	-0.563898	0.315751
H	3.730358	-1.540361	0.565104
C	4.713875	0.060958	1.607914
H	5.151820	1.044599	1.417960
H	3.945864	0.163823	2.375220
H	5.502867	-0.586951	1.995078
C	5.213974	-0.734550	-0.755000
H	4.788660	-1.151713	-1.668738
H	5.707209	0.211753	-0.992428
H	5.980504	-1.422928	-0.392812

TS6Spri

C	0.910964	0.187585	-0.057491
C	0.241865	-0.405170	1.235995
H	-0.351210	0.386885	1.683087
C	1.099274	1.700508	0.061954
C	1.018171	2.481253	-1.093642
C	1.373667	2.325213	1.279799

C	1.197166	3.858344	-1.029661
H	0.795201	1.993676	-2.033217
C	1.557889	3.705883	1.344355
H	1.437662	1.742613	2.191337
C	1.468384	4.477027	0.190240
H	1.125785	4.451877	-1.934161
H	1.766060	4.175739	2.298806
H	1.607411	5.550743	0.239275
C	2.266596	-0.439099	-0.431334
C	3.438055	-0.114650	0.262439
C	2.357706	-1.326999	-1.503388
C	4.653243	-0.704050	-0.071082
H	3.412175	0.623346	1.054075
C	3.574426	-1.915682	-1.842400
H	1.472688	-1.533388	-2.085674
C	4.725532	-1.616850	-1.120864
H	5.546784	-0.439560	0.482821
H	3.620042	-2.603030	-2.679627
H	5.672327	-2.074122	-1.383730
O	-0.032230	-0.070884	-1.086982
B	-1.007063	-1.082927	-0.788515
N	-0.726567	-1.450325	0.751319
C	1.111230	-1.083725	2.307741
H	2.162431	-0.837293	2.185121
H	0.802512	-0.744986	3.296530
C	0.868721	-2.604159	2.154031
H	1.803186	-3.161218	2.070991
H	0.318145	-2.988477	3.011816
C	0.042500	-2.741911	0.876100
H	0.700073	-2.847278	0.015666
H	-0.660587	-3.571505	0.886110
B	-2.001674	-1.502643	1.727774
H	-2.696308	-2.432816	1.411639
H	-1.638510	-1.518566	2.877693
H	-2.632069	-0.438878	1.561274
O	-2.487628	-0.487871	-0.832251
C	-3.048025	0.353951	-0.083760
C	-1.143606	-2.296876	-1.817009
H	-1.931429	-2.992653	-1.513568
H	-1.422537	-1.901478	-2.799310
H	-0.226515	-2.873941	-1.952160
C	-2.405126	1.658401	0.285533
H	-1.329568	1.593031	0.373520
H	-2.607617	2.349317	-0.540803
H	-2.844522	2.070811	1.192926

C	-4.557858	0.266224	0.031514
H	-4.818133	0.623242	1.032303
C	-5.082382	-1.154459	-0.164768
H	-4.842816	-1.517841	-1.165642
H	-4.635688	-1.836569	0.556996
H	-6.167877	-1.166743	-0.041531
C	-5.183714	1.234341	-1.000273
H	-6.271226	1.151675	-0.953841
H	-4.917790	2.275760	-0.811631
H	-4.867619	0.971175	-2.012934

TS7R

C	0.684670	0.162412	-0.161160
C	0.022457	-0.567224	1.082060
H	-0.728416	0.096072	1.500103
C	0.350665	1.655461	-0.135473
C	0.064018	2.311120	-1.332984
C	0.334767	2.389456	1.052616
C	-0.240896	3.668738	-1.341252
H	0.063603	1.736416	-2.248942
C	0.035102	3.749866	1.046458
H	0.538644	1.901871	1.999114
C	-0.256826	4.394751	-0.151739
H	-0.496592	5.451702	-0.158703
C	2.207673	-0.022947	-0.263853
C	3.092633	0.682819	0.557118
C	2.738909	-0.916523	-1.195304
C	4.465382	0.469671	0.478586
H	2.715069	1.419271	1.254185
C	4.113151	-1.127320	-1.280553
H	2.062932	-1.429414	-1.864044
C	4.983039	-0.442692	-0.437617
H	6.052485	-0.606399	-0.502159
O	0.095847	-0.443864	-1.302966
B	-1.005906	-1.282914	-0.981309
N	-0.703721	-1.752072	0.509562
C	0.932473	-1.143625	2.164065
H	1.813040	-0.529159	2.338371
H	0.370193	-1.203105	3.095354
C	1.297025	-2.559932	1.667076
H	2.328327	-2.597891	1.314861
H	1.185667	-3.290989	2.468000
C	0.333387	-2.853342	0.501744
H	0.864869	-2.825270	-0.447165

H	-0.178609	-3.808142	0.587988
B	-1.938634	-2.203031	1.423740
H	-2.399670	-3.222352	0.971973
H	-1.589154	-2.306453	2.573214
H	-2.794572	-1.300702	1.376693
O	-2.225464	-0.257492	-0.724637
C	-3.374356	-0.486510	-0.272121
C	-4.211045	-1.653173	-0.708090
H	-3.622274	-2.548731	-0.860472
H	-5.000402	-1.850649	0.015116
H	-4.674349	-1.367900	-1.661057
C	-4.098037	0.663656	0.379151
H	-4.761264	0.257921	1.146543
H	-4.760454	1.061195	-0.404737
C	-3.199973	1.764321	0.928680
H	-2.592005	1.385544	1.751784
H	-2.525391	2.148390	0.164941
H	-3.804874	2.590336	1.307654
H	4.502739	-1.825087	-2.013325
H	5.131466	1.026059	1.128174
H	-0.468777	4.161480	-2.279836
H	0.021383	4.301400	1.979635
C	-1.334769	-2.392294	-2.076674
H	-0.406593	-2.750340	-2.530894
H	-1.854296	-3.266189	-1.676159
H	-1.943727	-1.983937	-2.888871

TS7S

C	0.780489	0.143628	-0.115375
C	0.192586	-0.608256	1.154459
H	-0.431302	0.088803	1.705358
C	0.522884	1.650488	-0.013952
C	0.247017	2.369483	-1.177939
C	0.577915	2.337380	1.200580
C	0.019367	3.740246	-1.127451
H	0.193758	1.833347	-2.115633
C	0.353125	3.712618	1.253700
H	0.792108	1.806421	2.121044
C	0.069623	4.419077	0.089172
H	-0.109144	5.487400	0.128014
C	2.275626	-0.108253	-0.350271
C	3.254400	0.545471	0.404423
C	2.685861	-1.015224	-1.328213
C	4.605006	0.273388	0.210488
H	2.965237	1.284335	1.140789
C	4.037908	-1.284638	-1.528502
H	1.934077	-1.490875	-1.941501
C	5.003677	-0.648138	-0.755019
H	6.055798	-0.857310	-0.909889
O	0.066283	-0.387999	-1.226038
B	-1.107352	-1.076842	-0.818404
N	-0.719641	-1.667613	0.602779
C	1.152716	-1.350346	2.082173
H	2.113940	-0.848624	2.171097
H	0.704362	-1.403655	3.073825
C	1.284462	-2.766578	1.479597
H	2.259037	-2.903831	1.009988
H	1.171601	-3.526725	2.252639
C	0.171766	-2.875008	0.420585
H	0.596873	-2.846314	-0.580869
H	-0.438619	-3.769373	0.516983
B	-1.897325	-2.031190	1.626598
H	-2.564250	-2.914974	1.144029
H	-1.427883	-2.324641	2.698998
H	-2.585601	-1.012361	1.780390
O	-2.105501	0.121514	-0.361415
C	-3.200701	0.105905	0.244841
C	-3.518296	1.319239	1.068362
H	-4.139199	1.989167	0.462325
H	-4.087047	1.049665	1.957881
H	-2.601638	1.842754	1.333710
C	-4.318336	-0.834524	-0.112126
H	-3.917418	-1.824300	-0.304609
H	-5.003764	-0.908691	0.733101
C	-5.053917	-0.303782	-1.359731
H	-4.372525	-0.227514	-2.208315
H	-5.856283	-0.993290	-1.627738
H	-5.499902	0.678271	-1.185855
H	0.398263	4.227680	2.206663
H	-0.200059	4.281555	-2.040989
H	5.346897	0.790061	0.808683
H	4.334842	-1.990487	-2.296115
C	-1.742463	-2.042337	-1.907634
H	-0.956847	-2.624639	-2.397621
H	-2.460974	-2.754280	-1.495568
H	-2.244012	-1.471281	-2.694604

TS8R

C	1.593860	0.117225	-0.233952

C	0.782956	-0.419212	1.009515
H	-0.096304	0.207813	1.132129
C	1.223858	1.562878	-0.544800
C	1.114297	1.973904	-1.873649
C	1.017064	2.496921	0.471692
C	0.800333	3.294599	-2.178449
H	1.255819	1.244642	-2.659127
C	0.718076	3.822437	0.167359
H	1.068483	2.196316	1.511445
C	0.606834	4.225990	-1.159582
H	0.707392	3.597593	-3.214989
H	0.556258	4.533181	0.969329
H	0.367345	5.255530	-1.399052
C	3.120450	-0.008482	-0.108317
C	3.856796	0.861515	0.701805
C	3.806650	-0.999999	-0.811879
C	5.232187	0.711098	0.845420
H	3.362101	1.677578	1.211374
C	5.185001	-1.147887	-0.675162
H	3.256651	-1.644294	-1.482584
C	5.902952	-0.301041	0.163091
H	5.781396	1.394340	1.482855
H	5.696807	-1.924171	-1.232382
H	6.974981	-0.416298	0.271498
O	1.174688	-0.718764	-1.318837
B	0.290859	-1.706449	-0.948904
N	0.305558	-1.785734	0.598694
C	1.518689	-0.641307	2.326962
H	2.270166	0.121194	2.519478
H	0.791386	-0.612046	3.137349
C	2.137114	-2.051035	2.208709
H	3.208190	-1.991614	2.014661
H	1.989863	-2.617204	3.128117
C	1.428830	-2.727268	1.019788
H	2.116022	-2.857075	0.185552
H	0.987406	-3.690633	1.259632
B	-1.010782	-2.251404	1.378562
H	-1.339216	-3.334878	0.985092
H	-0.836709	-2.171071	2.567236
H	-1.893957	-1.414252	1.103300
O	-1.413031	-0.655006	-1.102585
C	-2.406792	-0.648345	-0.362088
C	0.037386	-2.942644	-1.868167
H	-0.738958	-3.600254	-1.481333
H	-0.253574	-2.618020	-2.869406
H	0.958611	-3.528067	-1.971861
C	-2.858566	0.620656	0.284452
C	-3.800938	0.664120	1.320420
C	-2.305570	1.810778	-0.200067
C	-4.180760	1.885950	1.859321
H	-4.216499	-0.251177	1.715955
C	-2.700506	3.031149	0.335680
C	-3.635932	3.071381	1.365705
H	-2.270045	3.945445	-0.051683
H	-3.940738	4.022992	1.785853
C	-3.498411	-1.730134	-0.623152
H	-1.577311	1.765180	-0.997497
H	-4.902159	1.914298	2.667185
F	-2.997166	-2.803729	-1.234053
F	-4.154705	-2.145137	0.474729
F	-4.413808	-1.176131	-1.451767

TS8Rpri

C	1.594012	0.117239	-0.234125
C	0.783250	-0.418858	1.009623
H	-0.095980	0.208204	1.132261
C	1.223460	1.562590	-0.545700
C	1.113125	1.972814	-1.874727
C	1.016814	2.497145	0.470356
C	0.798538	3.293223	-2.180130
H	1.254500	1.243151	-2.659855
C	0.717231	3.822385	0.165410
H	1.068801	2.197145	1.510256
C	0.605198	4.225133	-1.161710
H	0.704967	3.595581	-3.216798
H	0.555557	4.533531	0.967053
H	0.365213	5.254445	-1.401659
C	3.120623	-0.007990	-0.108362
C	3.856784	0.862871	0.700993
C	3.807005	-1.000137	-0.810868
C	5.232184	0.712762	0.844868
H	3.361932	1.679316	1.209792
C	5.185359	-1.147720	-0.673892
H	3.257153	-1.645162	-1.480991
C	5.903135	-0.299955	0.163585
H	5.781252	1.396688	1.481692
H	5.697308	-1.924494	-1.230297
H	6.975169	-0.414969	0.272194
O	1.175178	-0.719442	-1.318605

B	0.291215	-1.706802	-0.948320
N	0.305778	-1.785498	0.599254
C	1.519199	-0.640587	2.326991
H	2.270443	0.122192	2.519366
H	0.791959	-0.611527	3.137445
C	2.138107	-2.050058	2.208698
H	3.208922	-1.990131	2.013396
H	1.992244	-2.615930	3.128514
C	1.428843	-2.727152	1.020839
H	2.115533	-2.858343	0.186402
H	0.986975	-3.689981	1.261994
B	-1.010735	-2.250825	1.378990
H	-1.338795	-3.334691	0.986268
H	-0.837177	-2.169448	2.567666
H	-1.893985	-1.414156	1.102655
O	-1.412643	-0.653860	-1.102765
C	-2.406420	-0.648061	-0.362251
C	0.037115	-2.943182	-1.867107
H	-0.740258	-3.599713	-1.480540
H	-0.252623	-2.618799	-2.868776
H	0.957785	-3.529683	-1.969673
C	-2.858703	0.620421	0.285061
C	-3.801478	0.662937	1.320699
C	-2.305600	1.810995	-0.198200
C	-4.181576	1.884267	1.860537
H	-4.217139	-0.252723	1.715296
C	-2.700746	3.030874	0.338531
C	-3.636569	3.070160	1.368230
H	-2.270168	3.945531	-0.047856
H	-3.941555	4.021381	1.789131
C	-3.497675	-1.730055	-0.624152
H	-1.577094	1.766137	-0.995444
H	-4.903304	1.911858	2.668133
F	-2.996062	-2.802777	-1.236336
F	-4.153529	-2.146550	0.473430
F	-4.413525	-1.175643	-1.451979

TS8S

C	1.760347	-0.012316	-0.224967
C	0.995369	-0.397627	1.103275
H	0.393307	0.452495	1.400436
C	1.782602	1.499118	-0.431961
C	1.725649	2.010752	-1.728789
C	1.905802	2.384662	0.639029
C	1.784011	3.382402	-1.948384
H	1.617199	1.325014	-2.557874
C	1.969676	3.758857	0.419863
H	1.938015	2.014528	1.657008
C	1.908328	4.262561	-0.875224
H	1.728588	3.766082	-2.960741
H	2.056147	4.433181	1.263918
H	1.950430	5.331717	-1.047293
C	3.187193	-0.567382	-0.320744
C	4.233525	0.013161	0.402745
C	3.470540	-1.665149	-1.134714
C	5.517315	-0.519512	0.351404
H	4.052164	0.900107	0.995885
C	4.757571	-2.195367	-1.193760
H	2.680812	-2.090403	-1.737508
C	5.784408	-1.632522	-0.442679
H	6.312526	-0.055917	0.923710
H	4.956175	-3.046774	-1.834762
H	6.785520	-2.045063	-0.486886
O	0.984612	-0.612535	-1.275521
B	-0.134480	-1.259426	-0.818639
N	0.045759	-1.495101	0.699839
C	1.788996	-0.976750	2.269534
H	2.765491	-0.510763	2.381574
H	1.225893	-0.806875	3.186699
C	1.893911	-2.488146	1.975344
H	2.888520	-2.745928	1.610999
H	1.699450	-3.073895	2.873593
C	0.847669	-2.780097	0.883469
H	1.335007	-3.030292	-0.057715
H	0.154061	-3.576429	1.138898
B	-1.244629	-1.608521	1.640227
H	-1.943629	-2.498303	1.236011
H	-0.906068	-1.712650	2.791194
H	-1.868781	-0.535751	1.543962
O	-1.313428	0.377101	-0.609002
C	-2.323684	0.412442	0.102875
C	-0.957166	-2.226367	-1.728547
H	-1.929789	-2.475505	-1.304943
H	-1.104923	-1.797488	-2.721820
H	-0.409100	-3.166623	-1.861740
C	-3.588108	-0.280677	-0.269730
C	-4.526425	-0.739975	0.659642
C	-3.831006	-0.437779	-1.638296
C	-5.691416	-1.353317	0.218648

H	-4.325387	-0.648903	1.717222
C	-5.004476	-1.041407	-2.074477
H	-3.096217	-0.074620	-2.344316
C	-5.934686	-1.502245	-1.146566
H	-6.408345	-1.724421	0.941085
H	-5.190493	-1.153881	-3.135855
H	-6.846786	-1.980158	-1.484598
C	-2.449481	1.659417	1.022225
F	-2.962932	2.663088	0.282290
F	-3.261922	1.480006	2.078551
F	-1.257680	2.058685	1.484485

TS8Spri

C	1.760456	-0.012207	-0.225058
C	0.995452	-0.397634	1.103154
H	0.393230	0.452363	1.400347
C	1.782602	1.499230	-0.431983
C	1.725179	2.010963	-1.728746
C	1.906095	2.384697	0.639041
C	1.783373	3.382638	-1.948246
H	1.616483	1.325283	-2.557845
C	1.969793	3.758912	0.419971
H	1.938652	2.014473	1.656979
C	1.907979	4.262717	-0.875057
H	1.727579	3.766398	-2.960553
H	2.056481	4.433173	1.264054
H	1.949935	5.331891	-1.047050
C	3.187355	-0.567204	-0.320724
C	4.233773	0.013795	0.402273
C	3.470618	-1.665510	-1.134003
C	5.517562	-0.518909	0.351120
H	4.052472	0.901082	0.994918
C	4.757640	-2.195763	-1.192865
H	2.680845	-2.091119	-1.736484
C	5.784568	-1.632427	-0.442274
H	6.312838	-0.054940	0.923033
H	4.956168	-3.047583	-1.833343
H	6.785679	-2.044987	-0.486342
O	0.984876	-0.612555	-1.275644
B	-0.134136	-1.259662	-0.818822
N	0.046022	-1.495262	0.699699
C	1.789264	-0.976566	2.269386
H	2.765825	-0.510605	2.381001
H	1.226468	-0.806464	3.186701
C	1.894225	-2.487992	1.975396
H	2.888789	-2.745673	1.610863
H	1.700066	-3.073667	2.873758
C	0.847763	-2.780280	0.883831
H	1.334783	-3.031168	-0.057330
H	0.154049	-3.576313	1.139915
B	-1.244599	-1.608570	1.639727
H	-1.943871	-2.497933	1.235120
H	-0.906405	-1.712896	2.790783
H	-1.868473	-0.535516	1.543515
O	-1.313750	0.376356	-0.609551
C	-2.323691	0.411998	0.102854
C	-0.956277	-2.226918	-1.728901
H	-1.928809	-2.476624	-1.305421
H	-1.104150	-1.797971	-2.722126
H	-0.407699	-3.166862	-1.862203
C	-3.588404	-0.280870	-0.269409
C	-4.527271	-0.738591	0.660194
C	-3.830998	-0.439393	-1.637858
C	-5.692488	-1.351768	0.219557
H	-4.326541	-0.646436	1.717741
C	-5.004664	-1.042882	-2.073702
C	-5.935424	-1.502136	-1.145556
H	-5.190429	-1.156446	-3.135009
H	-6.847693	-1.979915	-1.483320
C	-2.449351	1.659471	1.021561
H	-3.095825	-0.077376	-2.344055
H	-6.409855	-1.721615	0.942204
F	-1.257300	2.059820	1.482220
F	-3.260451	1.480155	2.078981
F	-2.964344	2.662338	0.281638

TS9R

C	-2.507440	-0.294931	0.166837
C	-1.779977	-0.330823	-1.243041
H	-1.353356	0.650978	-1.431995
C	-2.740850	1.154096	0.597257
C	-2.538928	1.514270	1.930052
C	-3.146593	2.135879	-0.310988
C	-2.727168	2.829487	2.343367
H	-2.206080	0.756045	2.625153
C	-3.344466	3.451670	0.103015
H	-3.290255	1.888008	-1.356198
C	-3.131339	3.804122	1.432756

H	-2.553304	3.095452	3.379746
H	-3.656400	4.199803	-0.616925
H	-3.276972	4.828147	1.756553
C	-3.835411	-1.068394	0.216820
C	-5.004550	-0.563356	-0.360484
C	-3.894249	-2.308876	0.854279
C	-6.186428	-1.297370	-0.340826
H	-5.005520	0.417927	-0.816203
C	-5.077656	-3.042959	0.880945
H	-3.006459	-2.682195	1.343951
C	-6.227234	-2.545928	0.274814
H	-7.079048	-0.886972	-0.798976
H	-5.099633	-4.003501	1.383256
H	-7.148312	-3.116749	0.293657
O	-1.602522	-0.923824	1.066160
B	-0.335033	-1.184048	0.485406
N	-0.636310	-1.285378	-1.066236
C	-2.547915	-0.853178	-2.454406
H	-3.598907	-0.573366	-2.435802
H	-2.100499	-0.433253	-3.354757
C	-2.350006	-2.384919	-2.421385
H	-3.263005	-2.891197	-2.106332
H	-2.076926	-2.760019	-3.407658
C	-1.230237	-2.638912	-1.395197
H	-1.639570	-3.071751	-0.485315
H	-0.434978	-3.282087	-1.762674
B	0.572613	-0.997651	-2.060512
H	1.416830	-1.840389	-1.885587
H	0.181069	-0.925336	-3.199163
H	1.022933	0.121342	-1.768279
O	0.414200	0.282285	0.556826
C	1.461457	0.713573	0.006051
C	0.517142	-2.341592	1.152600
H	1.424623	-2.578948	0.593263
H	0.797746	-2.085811	2.176128
H	-0.075043	-3.259471	1.206839
C	1.518346	2.148454	-0.326283
C	2.705771	2.776219	-0.726263
C	0.346574	2.908565	-0.190059
C	2.713956	4.136172	-1.005793
H	3.621804	2.210963	-0.818283
C	0.361857	4.266599	-0.466853
C	1.543355	4.882421	-0.880163
H	-0.548943	4.841139	-0.353951
H	1.554284	5.944096	-1.097931
H	-0.559510	2.428053	0.145176
H	3.633807	4.615166	-1.318109
C	2.740226	-0.067545	0.118463
C	3.111148	-0.522427	1.386200
C	3.609531	-0.340781	-0.938955
C	4.254416	-1.280647	1.588077
C	4.750747	-1.109558	-0.757073
C	5.072630	-1.583462	0.508223
F	3.381450	0.128150	-2.165152
F	5.552389	-1.379056	-1.789456
F	6.174096	-2.309280	0.687946
F	4.574031	-1.710459	2.810426
F	2.370501	-0.223004	2.457769

TS9Rpri

C	2.438336	-0.155075	-0.142599
C	1.623388	-0.457278	1.157304
H	0.991806	0.406349	1.347840
C	2.771596	1.325665	-0.293157
C	3.011852	1.826427	-1.575822
C	2.933140	2.172015	0.801930
C	3.402583	3.147017	-1.757406
H	2.884673	1.166327	-2.424012
C	3.328989	3.496935	0.621865
H	2.742963	1.813074	1.805527
C	3.566866	3.988742	-0.656451
H	3.585757	3.521314	-2.758371
H	3.446165	4.143032	1.484342
H	3.877954	5.017453	-0.797049
C	3.767028	-0.927254	-0.275007
C	4.885533	-0.586075	0.493211
C	3.894731	-1.956591	-1.208112
C	6.074434	-1.298913	0.380264
H	4.842751	0.261246	1.164921
C	5.086923	-2.667352	-1.329336
H	3.056741	-2.185712	-1.849512
C	6.177947	-2.352014	-0.525951
H	6.925756	-1.020763	0.990969
H	5.161322	-3.465062	-2.059737
H	7.105188	-2.905485	-0.617732
O	1.556854	-0.553951	-1.189128
B	0.534157	-1.455080	-0.778681
N	0.718719	-1.605005	0.792159
C	2.361660	-0.910920	2.415483

H	3.345866	-0.458273	2.505431
H	1.777661	-0.617839	3.286681
C	2.436793	-2.451065	2.312855
H	3.458435	-2.784697	2.127567
H	2.090348	-2.913530	3.236720
C	1.538749	-2.837759	1.126151
H	2.145846	-3.109572	0.268201
H	0.852900	-3.652637	1.340981
B	-0.592567	-1.653231	1.728470
H	-1.201133	-2.658137	1.482525
H	-0.263092	-1.553538	2.885295
H	-1.289919	-0.672759	1.460370
O	-0.972069	-0.723899	-1.000321
C	-1.618753	0.090629	-0.302617
C	0.299699	-2.766700	-1.643904
H	-0.536960	-3.357139	-1.264377
H	0.061752	-2.484856	-2.674162
H	1.178758	-3.415011	-1.681568
C	-1.051058	1.400608	0.097061
C	-1.287871	1.980979	1.348063
C	-0.339953	2.103973	-0.878095
C	-0.817989	3.256501	1.614905
H	-1.810144	1.413512	2.107971
C	0.094297	3.398952	-0.616440
C	-0.139627	3.973212	0.625941
H	0.648308	3.939949	-1.371016
H	0.222829	4.972550	0.834600
H	-0.137213	1.633048	-1.829868
H	-0.980264	3.697094	2.591236
C	-3.093290	-0.129723	-0.223529
C	-4.023355	0.919711	-0.148829
C	-3.621527	-1.432788	-0.298490
C	-5.390756	0.688634	-0.088415
C	-4.985532	-1.673014	-0.225611
C	-5.874367	-0.610744	-0.121461
F	-3.648095	2.201323	-0.170943
F	-6.242909	1.714340	-0.021974
F	-7.182681	-0.838122	-0.065471
F	-5.452104	-2.921645	-0.268907
F	-2.841446	-2.497114	-0.414379

TS9S

C	2.297457	-0.558062	0.175798
C	1.945166	0.962637	0.304065
H	0.999724	1.118437	-0.209693
C	1.708155	-1.190300	-1.072244
C	1.269765	-2.514396	-1.031796
C	1.665274	-0.496090	-2.282451
C	0.817962	-3.138067	-2.188927
H	1.272700	-3.041591	-0.088090
C	1.223387	-1.126018	-3.443308
H	1.965903	0.543456	-2.331697
C	0.805956	-2.451261	-3.400720
H	0.469286	-4.163128	-2.142521
H	1.184879	-0.570116	-4.371943
H	0.452479	-2.939697	-4.301117
C	3.798492	-0.860357	0.267884
C	4.626175	-0.689772	-0.846505
C	4.379146	-1.270328	1.470273
C	6.000730	-0.875518	-0.748298
H	4.194407	-0.424772	-1.802310
C	5.755300	-1.465263	1.567564
H	3.756489	-1.455567	2.333973
C	6.573950	-1.257277	0.462698
H	6.623543	-0.732328	-1.623653
H	6.183857	-1.785561	2.510222
H	7.644544	-1.406547	0.538137
O	1.660950	-1.132218	1.342732
B	1.425207	-0.208593	2.301955
N	1.687747	1.176504	1.773750
C	2.981663	2.016650	-0.070687
H	3.574840	1.739524	-0.940798
H	2.454050	2.944969	-0.298759
C	3.837411	2.182230	1.194134
H	4.644932	1.451295	1.204892
H	4.283531	3.175605	1.258560
C	2.862130	1.935854	2.353856
H	3.314366	1.362488	3.163804
H	2.469902	2.862677	2.766099
B	0.273551	1.996223	2.005168
H	0.243849	2.315528	3.167525
H	0.311891	2.939313	1.246349
H	-0.592963	1.202135	1.725319
O	-1.860124	-1.695887	0.702743
C	-2.774581	-0.968237	0.379762
C	0.997519	-0.560758	3.751584
H	-0.048092	-0.275325	3.896141
H	1.096300	-1.628314	3.955350
H	1.570449	0.001759	4.494512

C	-4.196076	-1.300072	0.646818
C	-5.246885	-0.547337	0.113310
C	-4.478356	-2.418995	1.441064
C	-6.564804	-0.908001	0.371500
H	-5.034354	0.311210	-0.510966
C	-5.793446	-2.772848	1.702776
H	-3.649002	-2.988988	1.839996
C	-6.838537	-2.017405	1.167754
H	-7.376705	-0.324766	-0.045937
H	-6.009713	-3.635003	2.322367
H	-7.866076	-2.295137	1.372240
C	-2.454134	0.333579	-0.324632
C	-1.772216	0.320147	-1.536520
C	-2.685721	1.559552	0.284797
C	-1.306312	1.493878	-2.110513
C	-2.226051	2.745244	-0.268765
C	-1.521407	2.705612	-1.462937
F	-3.315390	1.609414	1.464585
F	-2.407566	3.906227	0.360260
F	-1.040086	3.832446	-1.991981
F	-0.653910	1.480024	-3.280993
F	-1.579972	-0.835280	-2.179390

TS9Spri

C	1.805351	-0.303623	-0.035706
C	1.094093	-0.939988	1.196996
H	0.281518	-0.273783	1.472380
C	1.798475	1.221708	0.009783
C	1.997641	1.919336	-1.185563
C	1.687314	1.941661	1.198844
C	2.086779	3.305357	-1.189144
H	2.072127	1.359677	-2.108965
C	1.775173	3.334591	1.196733
H	1.532452	1.428956	2.140071
C	1.978562	4.019383	0.004344
H	2.231151	3.832197	-2.124692
H	1.674044	3.879613	2.127943
H	2.039789	5.100801	0.000415
C	3.268190	-0.745943	-0.224624
C	4.281032	-0.266999	0.614144
C	3.621269	-1.600058	-1.268515
C	5.596956	-0.687671	0.456111
H	4.049770	0.463587	1.378512
C	4.940454	-2.017892	-1.433975
H	2.857887	-1.918196	-1.961691
C	5.931676	-1.576365	-0.563773
H	6.364440	-0.308472	1.121121
H	5.191318	-2.684021	-2.251728
H	6.957613	-1.901959	-0.690189
O	1.015036	-0.740259	-1.137267
B	0.261204	-1.937372	-0.885453
N	0.467430	-2.203639	0.679928
C	1.899199	-1.358298	2.426081
H	2.761299	-0.719032	2.598648
H	1.252587	-1.290716	3.300243
C	2.303994	-2.826834	2.173279
H	3.369993	-2.907787	1.957742
H	2.088410	-3.440640	3.047637
C	1.489840	-3.285369	0.950550
H	2.137757	-3.390722	0.085642
H	0.960720	-4.223200	1.104446
B	-0.857208	-2.534043	1.510468
H	-1.390737	-3.510933	1.047527
H	-0.606292	-2.593563	2.687723
H	-1.618995	-1.563134	1.332494
O	-1.322229	-1.673386	-1.054898
C	-2.091049	-0.934740	-0.382416
C	0.448441	-3.167852	-1.882537
H	-0.185869	-4.013012	-1.598974
H	0.147568	-2.858944	-2.888700
H	1.473790	-3.535695	-1.951734
C	-3.502239	-1.343488	-0.262437
C	-4.511345	-0.437706	0.085306
C	-3.832760	-2.678342	-0.539158
C	-5.831828	-0.862435	0.163046
H	-4.272960	0.598950	0.279808
C	-5.151253	-3.096666	-0.458362
C	-6.152899	-2.190856	-0.105066
H	-5.402405	-4.129754	-0.665616
H	-7.183085	-2.521209	-0.041097
H	-3.040867	-3.365196	-0.800362
H	-6.609453	-0.157250	0.429731
C	-1.735124	0.506522	-0.166294
C	-1.888200	1.194035	1.038994
C	-1.354810	1.251955	-1.286660
C	-1.639519	2.554071	1.135980
C	-1.148748	2.621860	-1.212634
C	-1.289435	3.274191	0.002045
F	-0.818235	3.311577	-2.305477

F	-1.087651	4.586578	0.086235
F	-1.734715	3.177857	2.313469
F	-2.261308	0.556592	2.153442
F	-1.233576	0.677828	-2.481871

B1-TS4Rpri

C	1.038336	-1.145895	-0.008973
C	0.648725	-0.713603	1.438094
H	1.216104	0.182818	1.673760
C	2.479127	-0.779297	-0.360927
C	2.812693	-0.640660	-1.711458
C	3.482317	-0.638365	0.597518
C	4.119457	-0.362911	-2.092984
H	2.031484	-0.745768	-2.452821
C	4.795186	-0.357717	0.216107
H	3.252592	-0.743391	1.650771
C	5.118018	-0.218885	-1.129028
H	4.361321	-0.254790	-3.144185
H	5.561953	-0.247428	0.974371
H	6.137095	-0.001121	-1.426331
C	0.859237	-2.649144	-0.285961
C	1.769676	-3.587841	0.212865
C	-0.204875	-3.105136	-1.062480
C	1.581666	-4.948568	-0.003166
H	2.646456	-3.256371	0.754342
C	-0.392150	-4.467812	-1.286453
H	-0.873903	-2.384064	-1.505580
C	0.491083	-5.396663	-0.746038
H	2.295540	-5.658956	0.397938
H	-1.228262	-4.799759	-1.891589
H	0.345145	-6.456799	-0.917605
O	0.163724	-0.386977	-0.832691
B	-1.022363	0.100581	-0.174198
N	-0.799956	-0.302523	1.366023
C	0.732134	-1.720182	2.581775
H	1.556364	-2.420466	2.465434
H	0.883869	-1.172051	3.510838
C	-0.635582	-2.427740	2.595429
H	-0.565030	-3.414884	2.137234
H	-0.997748	-2.555487	3.615634
C	-1.581234	-1.536073	1.769831
H	-1.915384	-2.060415	0.879765
H	-2.456561	-1.213500	2.325911
B	-1.057153	0.852763	2.473162
H	-2.188242	1.253550	2.383271
H	-0.790425	0.407134	3.563252
H	-0.269800	1.768126	2.217621
O	-1.075480	1.705786	-0.155905
C	-0.341930	2.586573	0.350841
C	-1.032372	3.842788	0.788996
H	-0.902733	4.603801	0.011394
H	-2.091577	3.641171	0.926720
H	-0.601080	4.230034	1.710183
C	1.150736	2.565578	0.182858
C	1.452044	3.162044	-1.222577
C	1.943355	3.304236	1.268523
H	1.470287	1.527130	0.137310
C	2.958308	3.133972	-1.502903
H	1.089254	4.195336	-1.268942
H	0.912541	2.585365	-1.977032
C	3.446002	3.250955	0.974106
H	1.627986	4.352599	1.309867
H	1.721903	2.863900	2.244171
C	3.761890	3.838271	-0.405492
H	3.150561	3.599051	-2.474832
H	3.284961	2.094402	-1.578657
H	3.992530	3.790923	1.752835
H	3.785287	2.210558	1.008727
H	4.832076	3.754764	-0.615898
H	3.522712	4.909689	-0.405775
C	-2.365684	-0.259316	-0.983860
H	-2.589053	-1.329958	-0.915442
H	-2.063808	-0.111221	-2.031076
C	-3.675195	0.516495	-0.765542
C	-4.623569	0.331499	-1.963311
C	-4.398628	0.127444	0.532202
H	-3.433049	1.584055	-0.700576
C	-5.943763	1.091240	-1.793303
H	-4.835560	-0.740121	-2.083035
H	-4.119823	0.651950	-2.881748
C	-5.719247	0.885365	0.712868
H	-4.602809	-0.952427	0.511392
H	-3.750564	0.316121	1.388246
C	-6.648724	0.698385	-0.490526
H	-6.599422	0.911305	-2.651630
H	-5.737237	2.169341	-1.777135
H	-6.216260	0.560705	1.632748
H	-5.502556	1.954339	0.836945
H	-7.566005	1.282017	-0.361091

H	-6.952027	-0.354970	-0.550105

B1-TS4S

C	-1.919462	0.430572	0.024019
C	-1.410415	1.515479	-1.013606
H	-1.015216	2.360714	-0.458916
C	-1.980124	1.017323	1.437088
C	-1.685501	0.191145	2.523170
C	-2.343434	2.342813	1.681887
C	-1.736478	0.683287	3.822468
H	-1.398999	-0.833825	2.331010
C	-2.399329	2.837920	2.984534
H	-2.581665	3.006301	0.858354
C	-2.091621	2.010529	4.059670
H	-1.496205	0.029584	4.653429
H	-2.681066	3.870928	3.154744
H	-2.129605	2.394272	5.072604
C	-3.277390	-0.187524	-0.333974
C	-4.475215	0.493309	-0.097011
C	-3.336701	-1.454384	-0.916165
C	-5.696420	-0.061155	-0.467427
H	-4.460529	1.458024	0.394119
C	-4.558608	-2.014549	-1.281724
H	-2.416698	-2.001361	-1.063482
C	-5.743606	-1.317347	-1.067556
H	-6.612877	0.485784	-0.277317
H	-4.582049	-3.001487	-1.730095
H	-6.694751	-1.751957	-1.352327
O	-0.944501	-0.602714	-0.015634
B	0.260328	-0.186894	-0.648337
N	-0.248304	0.874225	-1.721471
C	-2.353307	1.970394	-2.125263
H	-3.389682	2.016744	-1.797263
H	-2.053806	2.967211	-2.447658
C	-2.145018	0.953870	-3.269009
H	-2.993209	0.273027	-3.346022
H	-2.033495	1.464703	-4.225456
C	-0.872987	0.166687	-2.900884
H	-1.128139	-0.848686	-2.603788
H	-0.139972	0.123279	-3.702655
B	0.787043	1.967582	-2.250782
H	1.699504	1.402342	-2.803980
H	0.222451	2.767985	-2.955719
H	1.204080	2.578633	-1.255101
O	0.933956	0.812272	0.435978
C	1.845440	1.675325	0.347207
C	1.699827	2.861767	1.256370
H	2.022830	3.779086	0.766759
H	0.666908	2.942725	1.590009
H	2.342188	2.706906	2.130616
C	3.184266	1.362861	-0.266225
C	3.985378	2.576244	-0.750959
C	4.005008	0.557311	0.775440
H	3.002671	0.712763	-1.120361
C	5.309710	2.119610	-1.372839
H	4.197946	3.246301	0.090212
H	3.393339	3.135115	-1.478476
C	5.332101	0.102340	0.158939
H	4.203722	1.185544	1.651642
H	3.425822	-0.301311	1.117044
C	6.137616	1.286265	-0.387501
H	5.881884	2.989567	-1.707459
H	5.094978	1.522789	-2.267178
H	5.914450	-0.444399	0.906595
H	5.120845	-0.602813	-0.653787
H	7.053495	0.929554	-0.867595
H	6.449879	1.923740	0.449608
C	1.200524	-1.395489	-1.106183
H	2.259837	-1.121087	-1.109830
H	0.974454	-1.648536	-2.148689
C	1.024151	-2.667657	-0.258174
C	1.767057	-3.869693	-0.859825
C	1.458166	-2.465579	1.201438
H	-0.045666	-2.910293	-0.241832
C	1.572195	-5.144837	-0.030402
H	2.838607	-3.632428	-0.917506
H	1.430664	-4.033651	-1.889485
C	1.247736	-3.729477	2.042098
H	2.525673	-2.206374	1.215490
H	0.919576	-1.621754	1.638510
C	1.978899	-4.931210	1.432483
H	2.140923	-5.972974	-0.465664
H	0.514965	-5.436943	-0.068288
H	1.584696	-3.562326	3.070474
H	0.173731	-3.948483	2.096007
H	1.783176	-5.835580	2.017530
H	3.061213	-4.753741	1.481818

B1-TS4Spri

C	1.962685	-0.463627	0.020336
C	1.544618	-1.280338	-1.254032
H	1.171885	-2.241520	-0.913708
C	2.376198	-1.391913	1.162673
C	2.138948	-0.986885	2.478534
C	3.005622	-2.617483	0.937716
C	2.512786	-1.796500	3.545056
H	1.645413	-0.039575	2.649331
C	3.385168	-3.428046	2.006821
H	3.198064	-2.956664	-0.073308
C	3.137782	-3.021323	3.313708
H	2.317050	-1.471299	4.560614
H	3.869313	-4.378656	1.814365
H	3.428693	-3.651953	4.145735
C	3.091905	0.556742	-0.208465
C	4.428140	0.156777	-0.320608
C	2.802147	1.919626	-0.274977
C	5.436412	1.088325	-0.547059
H	4.693026	-0.886321	-0.203732
C	3.810213	2.855266	-0.496572
H	1.783859	2.244227	-0.129815
C	5.130731	2.443906	-0.645042
H	6.464162	0.754507	-0.633363
H	3.559307	3.909000	-0.543726
H	5.916282	3.170566	-0.816748
O	0.774679	0.217965	0.398538
B	-0.231002	0.318641	-0.624400
N	0.372949	-0.542114	-1.841689
C	2.547232	-1.476287	-2.400216
H	3.572559	-1.331672	-2.069889
H	2.460378	-2.492869	-2.782686
C	2.150707	-0.454487	-3.491819
H	2.978900	0.207732	-3.748178
H	1.836124	-0.971307	-4.397787
C	0.994165	0.347628	-2.891472
H	1.374019	1.246461	-2.412442
H	0.228763	0.629047	-3.610317
B	-0.623346	-1.573854	-2.559490
H	-1.477879	-0.951862	-3.133660
H	0.002066	-2.329675	-3.261165
H	-1.151816	-2.263663	-1.659349
O	-1.567413	-0.425173	-0.186521
C	-1.816608	-1.649789	-0.028748
C	-0.931328	-2.554537	0.778352
H	0.092476	-2.209111	0.825060
H	-1.318750	-2.538042	1.803489
H	-0.982619	-3.580082	0.415401
C	-3.258057	-2.067029	-0.203396
C	-3.996347	-1.784600	1.135934
C	-3.967372	-1.348683	-1.358247
H	-3.274225	-3.148707	-0.375657
C	-5.475721	-2.173730	1.044669
H	-3.906699	-0.715694	1.357672
H	-3.521939	-2.326469	1.958105
C	-5.444740	-1.743837	-1.439561
H	-3.878498	-0.269433	-1.202694
H	-3.452315	-1.572659	-2.292116
C	-6.175759	-1.470722	-0.121741
H	-5.971280	-1.935492	1.990701
H	-5.553820	-3.260150	0.913243
H	-5.923243	-1.201564	-2.260065
H	-5.524854	-2.810924	-1.683579
H	-7.218771	-1.794502	-0.186283
H	-6.190770	-0.389883	0.065462
C	-0.807265	1.769010	-0.999873
H	-1.469278	1.646049	-1.866799
H	-0.014089	2.449973	-1.329402
C	-1.602704	2.466530	0.117240
C	-0.709080	2.924329	1.281511
C	-2.411313	3.657650	-0.421191
H	-2.323821	1.743791	0.521683
C	-1.511554	3.619243	2.387589
H	0.041560	3.626191	0.890815
H	-0.156659	2.072982	1.683493
C	-3.220992	4.356619	0.676871
H	-1.718546	4.378449	-0.877214
H	-3.075888	3.316933	-1.222534
C	-2.324127	4.797558	1.839001
H	-0.841639	3.958405	3.184573
H	-2.196618	2.892619	2.844150
H	-3.759698	5.216049	0.264494
H	-3.982750	3.663004	1.056515
H	-2.925159	5.251448	2.633715
H	-1.634276	5.574499	1.484765

B2-TS4R

C	-0.191505	1.256794	0.065685
C	-0.175600	0.773289	1.550219

H	-1.089215	0.209403	1.717377
C	-1.606808	1.512416	-0.450214
C	-1.825080	1.462904	-1.830335
C	-2.669123	1.852804	0.386857
C	-3.078912	1.743372	-2.359007
H	-1.000527	1.194730	-2.477702
C	-3.929583	2.133446	-0.142534
H	-2.528099	1.900622	1.459659
C	-4.138581	2.079831	-1.515873
H	-3.232906	1.697858	-3.431107
H	-4.745118	2.392342	0.522957
H	-5.116613	2.298161	-1.928340
C	0.635263	2.528379	-0.192044
C	0.167838	3.784977	0.209228
C	1.859388	2.457355	-0.855340
C	0.935488	4.927671	0.013559
H	-0.813142	3.878726	0.657225
C	2.628157	3.601655	-1.059016
H	2.198096	1.502340	-1.225863
C	2.177403	4.839565	-0.612660
H	0.557134	5.890061	0.338777
H	3.578584	3.521746	-1.574490
H	2.775621	5.729831	-0.767968
O	0.359445	0.159068	-0.651670
B	1.148898	-0.742415	0.145175
N	0.962541	-0.208011	1.643477
C	0.054979	1.773857	2.679549
H	-0.372967	2.751300	2.468580
H	-0.416966	1.386859	3.581855
C	1.583996	1.835061	2.860453
H	1.989560	2.747175	2.421106
H	1.850632	1.822500	3.917283
C	2.146075	0.605678	2.123074
H	2.740157	0.918551	1.269640
H	2.757220	-0.031325	2.757058
B	0.616520	-1.312709	2.775115
H	1.494605	-2.135942	2.803838
H	0.446697	-0.746642	3.828590
H	-0.447837	-1.839813	2.436581
O	0.516981	-2.224086	0.158861
C	-0.564385	-2.684751	0.593656
C	-0.522449	-4.093945	1.103889
H	-0.894413	-4.761797	0.319007
H	0.503026	-4.357623	1.349463
H	-1.160962	-4.216131	1.976704
C	2.622652	-1.041617	-0.421771
C	3.447864	-2.069496	0.373798
C	2.531446	-1.473871	-1.900754
H	3.193173	-0.102735	-0.411629
C	4.831446	-2.302789	-0.245817
H	2.900503	-3.017673	0.401659
H	3.556199	-1.756247	1.414988
C	3.910518	-1.714174	-2.526127
H	1.945946	-2.400241	-1.962498
H	1.980296	-0.722257	-2.473509
C	4.724296	-2.728007	-1.714371
H	5.380948	-3.057406	0.327162
H	5.416124	-1.375381	-0.182861
H	3.804469	-2.054718	-3.561946
H	4.458442	-0.763318	-2.564222
H	5.720482	-2.856387	-2.150623
H	4.229941	-3.706910	-1.766657
C	-1.883476	-2.042202	0.267226
C	-2.279470	-2.536430	-1.154260
C	-3.010188	-2.309810	1.272921
H	-1.722491	-0.970774	0.172146
C	-3.600642	-1.897028	-1.595132
H	-2.384187	-3.627451	-1.148976
H	-1.480239	-2.283021	-1.854193
C	-4.316217	-1.652352	0.814804
H	-3.173069	-3.388811	1.369928
H	-2.713823	-1.941124	2.258368
C	-4.722298	-2.137679	-0.580440
H	-3.880822	-2.293406	-2.576031
H	-3.450697	-0.821884	-1.718316
H	-5.108334	-1.865326	1.538588
H	-4.187009	-0.565494	0.793225
H	-5.634958	-1.630312	-0.906218
H	-4.954714	-3.209634	-0.537280

B2-TS4Rpri

C	-0.907332	-1.229300	-0.088743
C	-0.638761	-0.902099	1.436200
H	0.412349	-1.080688	1.636484
C	0.250666	-2.056719	-0.652974
C	0.747441	-1.761458	-1.922380
C	0.842062	-3.092450	0.075162
C	1.821528	-2.475395	-2.445762
H	0.297301	-0.949927	-2.477101
C	1.912501	-3.813021	-0.448997

H	0.491336	-3.326453	1.073598
C	2.410314	-3.503264	-1.711596
H	2.202671	-2.225958	-3.429720
H	2.363449	-4.607146	0.135056
H	3.249645	-4.055345	-2.118104
C	-2.241878	-1.946431	-0.358263
C	-2.414998	-3.310886	-0.106311
C	-3.323540	-1.225984	-0.868195
C	-3.644827	-3.926953	-0.317390
H	-1.583171	-3.908863	0.241490
C	-4.553934	-1.841089	-1.086231
H	-3.182023	-0.182375	-1.108933
C	-4.724019	-3.192787	-0.802999
H	-3.755567	-4.985451	-0.111135
H	-5.378748	-1.260945	-1.484667
H	-5.681028	-3.672825	-0.971519
O	-0.958494	0.031007	-0.728288
B	-0.584555	1.127066	0.111853
N	-0.869338	0.580593	1.584997
C	-1.528706	-1.554683	2.489319
H	-1.815947	-2.568454	2.218272
H	-0.983617	-1.596185	3.431566
C	-2.748695	-0.618937	2.614239
H	-3.618792	-1.044621	2.113640
H	-3.007528	-0.455652	3.660568
C	-2.339524	0.697587	1.926663
H	-2.903776	0.842397	1.007534
H	-2.474178	1.569299	2.560605
B	-0.000689	1.168031	2.798775
H	-0.286894	2.326707	2.961340
H	-0.161321	0.491454	3.783758
H	1.203378	1.075486	2.481859
O	1.018220	1.090895	0.074581
C	1.902491	1.633799	0.788621
C	1.875118	3.084990	1.156923
H	2.227041	3.645687	0.282664
H	0.876271	3.416349	1.405894
H	2.545249	3.282563	1.991699
C	3.235504	0.927901	0.846248
C	3.121037	-0.586776	1.047766
C	3.965399	1.237727	-0.491220
H	3.816504	1.367211	1.663131
C	4.487284	-1.274066	0.982833
H	2.475686	-0.993856	0.269853
H	2.638549	-0.783712	2.008405
C	5.330882	0.545429	-0.548687
H	3.333766	0.879957	-1.310391
H	4.085130	2.317463	-0.618206
C	5.203267	-0.966015	-0.335160
H	4.351303	-2.353146	1.095753
H	5.110477	-0.942960	1.823504
H	5.803004	0.758193	-1.512669
H	5.985377	0.969932	0.222756
H	6.192047	-1.434179	-0.351319
H	4.629175	-1.401510	-1.161068
C	-1.256370	2.478971	-0.451929
C	-1.567000	3.688487	0.448853
C	-0.528663	2.949873	-1.730541
H	-2.238839	2.118927	-0.794304
C	-2.386111	4.746541	-0.303669
H	-0.646578	4.168653	0.792823
H	-2.100607	3.379230	1.349561
C	-1.318724	4.027094	-2.482751
H	0.455813	3.353682	-1.460005
H	-0.342515	2.094161	-2.385502
C	-1.663615	5.211828	-1.573370
H	-2.591062	5.601188	0.349589
H	-3.359467	4.319640	-0.578181
H	-0.756069	4.371645	-3.357134
H	-2.248043	3.583961	-2.862701
H	-2.272143	5.943789	-2.114184
H	-0.736617	5.726319	-1.286989

B2-TS4S

C	1.961703	0.269106	0.007074
C	1.351935	0.441034	1.459583
H	1.029888	1.470921	1.576502
C	2.156866	1.634445	-0.659642
C	1.939507	1.753561	-2.032916
C	2.564743	2.762799	0.054542
C	2.112090	2.974671	-2.674882
H	1.612379	0.881239	-2.582065
C	2.741286	3.988278	-0.587153
H	2.743263	2.700623	1.122008
C	2.511773	4.099484	-1.954839
H	1.931208	3.050422	-3.741307
H	3.055386	4.853794	-0.014712
H	2.644029	5.051658	-2.455492
C	3.279566	-0.515524	-0.028485
C	4.493991	0.077238	0.329736

C	3.282097	-1.856986	-0.413316
C	5.673171	-0.661167	0.338003
H	4.526317	1.126978	0.591915
C	4.462959	-2.596086	-0.413425
H	2.352680	-2.307709	-0.729336
C	5.662605	-2.004745	-0.029387
H	6.603206	-0.182272	0.622475
H	4.442968	-3.636050	-0.719617
H	6.581708	-2.579182	-0.027971
O	0.991726	-0.478074	-0.711643
B	-0.276619	-0.484280	-0.070207
N	0.106862	-0.408723	1.481333
C	2.176992	-0.024162	2.656577
H	3.242010	0.148912	2.516907
H	1.854517	0.529318	3.537756
C	1.843687	-1.522106	2.813588
H	2.661165	-2.143775	2.447519
H	1.671340	-1.774384	3.860126
C	0.580314	-1.761187	1.965103
H	0.811540	-2.378795	1.098618
H	-0.228419	-2.226550	2.521196
B	-0.951260	0.196081	2.520935
H	-1.916827	-0.519906	2.582612
H	-0.422129	0.364066	3.592147
H	-1.279958	1.312494	2.093100
O	-0.796124	1.041929	-0.279883
C	-1.739695	1.682377	0.248934
C	-1.540724	3.167104	0.351914
H	-1.960346	3.560588	1.276271
H	-0.480513	3.401579	0.277772
H	-2.066556	3.643698	-0.482879
C	-3.124571	1.101644	0.357018
C	-4.012084	1.709318	1.448880
C	-3.806894	1.244492	-1.030462
H	-3.013317	0.038256	0.557721
C	-5.377659	1.014047	1.467974
H	-4.157142	2.779444	1.260743
H	-3.515963	1.607253	2.415849
C	-5.175298	0.554430	-1.014892
H	-3.936617	2.306676	-1.267838
H	-3.169152	0.814606	-1.805918
C	-6.071266	1.098857	0.103232
H	-6.009188	1.457337	2.243035
H	-5.236681	-0.038258	1.741775
H	-5.659605	0.680804	-1.987821
H	-5.027825	-0.522387	-0.873303
H	-7.019288	0.553500	0.124055
H	-6.316791	2.146810	-0.111152
C	-1.304085	-1.572037	-0.684846
C	-0.613014	-2.499092	-1.710502
C	-2.131845	-2.447073	0.280384
H	-2.031881	-0.989449	-1.266649
C	-1.622150	-3.367017	-2.470936
H	0.096476	-3.151067	-1.183553
H	-0.018381	-1.909478	-2.410339
C	-3.167498	-3.292833	-0.469978
H	-1.460730	-3.124211	0.819518
H	-2.623331	-1.846693	1.048053
C	-2.501371	-4.187933	-1.520972
H	-1.101151	-4.029707	-3.170262
H	-2.262588	-2.714377	-3.079099
H	-3.743820	-3.900495	0.235663
H	-3.883712	-2.625289	-0.967466
H	-3.256287	-4.746754	-2.083768
H	-1.878111	-4.932606	-1.009022

B2-TS4Spri

C	-1.877164	-0.472709	0.028982
C	-1.321795	-0.571951	1.490579
H	-0.898083	-1.564590	1.615312
C	-2.286866	-1.843032	-0.512789
C	-2.178785	-2.077582	-1.885927
C	-2.788223	-2.855909	0.306442
C	-2.554219	-3.302449	-2.425133
H	-1.781848	-1.292999	-2.516082
C	-3.168861	-4.083890	-0.234062
H	-2.878155	-2.702015	1.375031
C	-3.051388	-4.311613	-1.600910
H	-2.459996	-3.471450	-3.491900
H	-3.552297	-4.861175	0.417009
H	-3.343811	-5.266454	-2.022084
C	-3.070613	0.485261	-0.124447
C	-4.353636	0.120033	0.297449
C	-2.894748	1.744358	-0.697359
C	-5.415699	1.013335	0.207038
H	-4.533872	-0.875438	0.682022
C	-3.958029	2.639447	-0.794929
H	-1.922246	2.012837	-1.078353
C	-5.220274	2.283968	-0.330693
H	-6.400205	0.711790	0.546166

H	-3.796639	3.614335	-1.241080
H	-6.047926	2.979889	-0.403570
O	-0.775118	0.000646	-0.728221
B	0.274127	0.627858	0.032039
N	-0.174679	0.404594	1.569185
C	-2.232503	-0.230042	2.665081
H	-3.261021	-0.549850	2.509104
H	-1.850866	-0.733732	3.553412
C	-2.112825	1.292441	2.817632
H	-2.907166	1.792628	2.263474
H	-2.189028	1.597719	3.861940
C	-0.742456	1.658099	2.221907
H	-0.840473	2.444046	1.480389
H	-0.032678	1.985597	2.977115
B	0.949766	-0.190009	2.556937
H	1.814759	0.629221	2.706914
H	0.429910	-0.552617	3.584019
H	1.435739	-1.205359	2.007741
O	1.645721	-0.158176	-0.130692
C	1.965726	-1.322903	0.232106
C	1.099120	-2.517160	-0.043753
H	0.041669	-2.299918	0.001783
H	1.307329	-2.821030	-1.075861
H	1.352942	-3.345167	0.616672
C	0.680719	2.103781	-0.463130
C	1.841486	2.766601	0.300661
C	0.997483	2.075201	-1.973368
H	-0.190106	2.764041	-0.349490
C	2.169389	4.162481	-0.243590
H	2.728575	2.132581	0.214217
H	1.620454	2.827229	1.369471
C	1.330623	3.465961	-2.525904
H	1.852742	1.408550	-2.141959
H	0.155687	1.640582	-2.520226
C	2.476383	4.119146	-1.744814
H	3.014715	4.594129	0.303155
H	1.313123	4.828332	-0.071865
H	1.584257	3.402549	-3.589710
H	0.439410	4.103530	-2.454244
H	2.674043	5.126636	-2.125549
H	3.393940	3.537475	-1.903982
C	3.435315	-1.587685	0.468414
C	4.074823	-2.046159	-0.871956
C	4.195765	-0.383208	1.032134
H	3.503212	-2.425143	1.171514
C	5.567609	-2.345821	-0.693082
H	3.942126	-1.247022	-1.609859
H	3.565682	-2.932139	-1.258783
C	5.682792	-0.698834	1.214811
H	4.080943	0.455707	0.340534
H	3.740824	-0.076528	1.973603
C	6.323558	-1.153079	-0.100212
H	5.997342	-2.631513	-1.657956
H	5.680649	-3.212085	-0.029489
H	6.199397	0.182270	1.605817
H	5.799817	-1.488591	1.967908
H	7.375292	-1.412539	0.052886
H	6.304060	-0.322010	-0.816090

B3-TS4R

C	-0.508496	-1.124358	0.037958
C	-0.430661	-0.598250	1.507034
H	0.622756	-0.460817	1.746904
C	0.703154	-1.974561	-0.343247
C	1.025202	-2.090468	-1.701345
C	1.459833	-2.685049	0.592514
C	2.083317	-2.895789	-2.113068
H	0.438566	-1.533337	-2.422713
C	2.525487	-3.490956	0.180886
H	1.227444	-2.617636	1.650110
C	2.840996	-3.598466	-1.171667
H	2.322323	-2.972798	-3.169508
H	3.105585	-4.032909	0.921651
H	3.667912	-4.224486	-1.492077
C	-1.770804	-1.948239	-0.272884
C	-1.910447	-3.260909	0.199332
C	-2.792728	-1.412833	-1.060011
C	-3.070746	-3.990138	-0.049893
H	-1.097441	-3.728088	0.744248
C	-3.953673	-2.143728	-1.317276
H	-2.661811	-0.427323	-1.483690
C	-4.104994	-3.429398	-0.801472
H	-3.161059	-5.002440	0.332541
H	-4.737230	-1.705563	-1.928425
H	-5.009027	-3.997503	-0.998035
O	-0.489893	0.062153	-0.747345
B	-0.891070	1.249383	-0.037071
N	-1.052244	0.771206	1.479555
C	-1.146669	-1.346780	2.630435
H	-1.152441	-2.426303	2.481774

H	-0.625839	-1.137462	3.565986
C	-2.569970	-0.754395	2.667314
H	-3.284953	-1.436570	2.201729
H	-2.892480	-0.578894	3.695599
C	-2.501404	0.558640	1.862981
H	-3.108017	0.480468	0.963017
H	-2.828408	1.431030	2.426656
B	-0.358025	1.694607	2.619198
H	-0.807365	2.812516	2.541246
H	-0.542498	1.175002	3.696704
H	0.854364	1.700438	2.378728
O	0.289123	2.339849	-0.003989
C	1.447164	2.338133	0.480750
C	1.970951	3.665362	0.944517
H	2.728700	4.024486	0.238343
H	1.150474	4.378553	1.001949
H	2.447514	3.567129	1.920562
C	2.388525	1.183364	0.286511
C	3.029145	1.362770	-1.121853
C	3.469364	1.038812	1.366644
H	1.798445	0.270023	0.229311
C	3.994170	0.210087	-1.424790
H	3.571839	2.316317	-1.161701
H	2.233255	1.402752	-1.871537
C	4.406565	-0.130853	1.044956
H	4.060508	1.961642	1.425724
H	2.991194	0.900780	2.341809
C	5.056115	0.043189	-0.332707
H	4.468451	0.386666	-2.397167
H	3.420844	-0.717237	-1.514792
H	5.173610	-0.213185	1.822946
H	3.835579	-1.067227	1.058307
H	5.695034	-0.816761	-0.562225
H	5.709623	0.927017	-0.316077
C	-2.057663	2.103014	-0.732954
C	-2.486175	3.413097	-0.015663
C	-1.632363	2.548810	-2.176081
H	-2.949415	1.475623	-0.836286
C	-2.795552	4.405827	-1.146539
H	-1.664461	3.800029	0.594133
H	-3.334458	3.259695	0.658924
C	-1.708690	4.088474	-2.181442
H	-2.323569	2.135536	-2.919465
H	-0.635525	2.189511	-2.449903
H	-3.785825	4.197676	-1.573627
H	-2.792629	5.451153	-0.819264
H	-1.912505	4.505440	-3.173357
H	-0.753442	4.506189	-1.839548

B3-TS4Rpri

C	-1.320403	-0.722213	-0.108065
C	-0.984904	-0.345107	1.393749
H	-0.061211	-0.845102	1.666566
C	-0.462514	-1.912142	-0.547626
C	0.123914	-1.903219	-1.813195
C	-0.234432	-3.007223	0.289954
C	0.929454	-2.959740	-2.227929
H	-0.041739	-1.046380	-2.451390
C	0.565585	-4.069358	-0.125119
H	-0.658327	-3.028315	1.287303
C	1.154438	-4.047632	-1.386309
H	1.385781	-2.932362	-3.211135
H	0.736870	-4.906466	0.541954
H	1.784452	-4.868407	-1.708855
C	-2.806978	-1.021044	-0.366344
C	-3.391451	-2.240927	-0.012460
C	-3.612338	-0.052073	-0.967613
C	-4.748207	-2.471052	-0.219317
H	-2.785812	-3.029366	0.414139
C	-4.969353	-0.281716	-1.181412
H	-3.156568	0.874793	-1.284315
C	-5.546508	-1.489170	-0.800683
H	-5.178844	-3.424249	0.065609
H	-5.573681	0.484976	-1.653260
H	-6.602287	-1.669471	-0.966596
O	-0.975460	0.426005	-0.860434
B	-0.248514	1.403907	-0.114631
N	-0.698585	1.134448	1.393538
C	-2.062700	-0.547036	2.457099
H	-2.693135	-1.408066	2.245764
H	-1.578184	-0.708562	3.419153
C	-2.865501	0.772363	2.477720
H	-3.850953	0.640399	2.029559
H	-3.004573	1.122408	3.500688
C	-2.042686	1.776869	1.650494
H	-2.526366	1.968615	0.694450
H	-1.878657	2.725284	2.154641
B	0.309749	1.506428	2.588044
H	0.414826	2.703392	2.664128
H	-0.071307	1.001806	3.615092

H	1.413659	1.004356	2.318483
O	1.246578	0.826671	-0.092337
C	2.262088	1.152257	0.573861
C	2.676116	2.572401	0.799779
H	3.363470	2.835077	-0.013969
H	1.835962	3.252565	0.784348
H	3.212413	2.668059	1.741963
C	3.313478	0.085094	0.744659
C	2.746879	-1.268459	1.192907
C	4.032028	-0.070672	-0.624956
H	4.043342	0.441213	1.478837
C	3.837853	-2.339967	1.259756
H	1.981115	-1.576582	0.480859
H	2.262150	-1.150667	2.164761
C	5.124576	-1.142090	-0.553386
H	3.279689	-0.355418	-1.367004
H	4.460288	0.884730	-0.942404
C	4.560277	-2.486405	-0.082928
H	3.388589	-3.293106	1.552521
H	4.564391	-2.080043	2.040228
H	5.592925	-1.246586	-1.536806
H	5.911546	-0.812393	0.136318
H	5.362509	-3.226445	-0.006404
H	3.849234	-2.859662	-0.828410
C	-0.410200	2.853574	-0.788648
C	-0.334649	4.179327	0.035890
C	0.516861	3.090166	-2.007134
H	-1.423552	2.816950	-1.206044
C	0.473264	5.183322	-0.824158
H	0.105923	4.050913	1.024824
H	-1.344399	4.558400	0.212523
C	0.443193	4.603148	-2.246844
H	0.212844	2.496532	-2.872051
H	1.549498	2.808073	-1.776896
H	0.076269	6.199473	-0.768057
H	1.510931	5.230169	-0.476058
H	-0.505726	4.853107	-2.734678
H	1.248534	4.984489	-2.880502

B3-TS4S

C	1.830766	0.289381	0.013709
C	1.191393	0.379657	1.463415
H	0.742369	1.363825	1.576398
C	1.883382	1.677821	-0.632788
C	1.666617	1.793351	-2.009218
C	2.159935	2.835428	0.103115
C	1.712951	3.037619	-2.633295
H	1.439171	0.897325	-2.573734
C	2.209832	4.084150	-0.520994
H	2.333774	2.775332	1.173400
C	1.983028	4.189926	-1.892080
H	1.533898	3.109522	-3.702157
H	2.422917	4.971203	0.068235
H	2.016361	5.159872	-2.378831
C	3.225814	-0.349933	-0.015109
C	4.367088	0.368495	0.363693
C	3.378384	-1.681877	-0.411562
C	5.621371	-0.238812	0.379895
H	4.280480	1.414772	0.635400
C	4.633860	-2.290420	-0.400862
H	2.506134	-2.227437	-0.747033
C	5.760187	-1.574833	0.002137
H	6.491902	0.336481	0.680670
H	4.729788	-3.325862	-0.714442
H	6.737257	-2.048149	0.011627
O	0.948746	-0.543350	-0.728357
B	-0.308486	-0.698451	-0.080836
N	0.063721	-0.616418	1.464315
C	2.051576	0.020268	2.676654
H	3.099243	0.282373	2.531080
H	1.680841	0.571186	3.542621
C	1.849477	-1.498719	2.882890
H	2.754920	-2.057318	2.634434
H	1.591457	-1.715978	3.921107
C	0.710955	-1.898529	1.926645
H	1.115508	-2.417845	1.060274
H	-0.052586	-2.523776	2.387248
B	-1.072987	-0.198841	2.508906
H	-1.963971	-1.017098	2.456162
H	-0.592896	-0.085191	3.612560
H	-1.483998	0.918941	2.159578
O	-1.023329	0.747377	-0.273619
C	-2.004759	1.311481	0.279903
C	-1.910162	2.807931	0.391700
H	-2.274009	3.151218	1.360913
H	-0.879638	3.125513	0.232475
H	-2.548427	3.259144	-0.378072
C	-3.340882	0.633725	0.445906
C	-4.229652	1.214434	1.553486
C	-4.084345	0.683731	-0.916590

H	-3.143678	-0.412666	0.678916
C	-5.541606	0.426352	1.649250
H	-4.458085	2.266452	1.337315
H	-3.691281	1.180356	2.504604
C	-5.396827	-0.104150	-0.823746
H	-4.300278	1.727970	-1.179075
H	-3.448879	0.274425	-1.705269
C	-6.289805	0.414150	0.309865
H	-6.174733	0.851927	2.435365
H	-5.314959	-0.604414	1.952037
H	-5.924181	-0.051559	-1.782825
H	-5.160798	-1.162644	-0.650862
H	-7.197266	-0.195208	0.384801
H	-6.616312	1.435865	0.070111
C	-1.210760	-1.863451	-0.698680
C	-1.373889	-1.710682	-2.228024
C	-0.697226	-3.317754	-0.609365
H	-2.213444	-1.854529	-0.249394
C	-2.023510	-3.029987	-2.679734
H	-0.377621	-1.602293	-2.672747
H	-1.947614	-0.827724	-2.524458
C	-1.487499	-4.101659	-1.689171
H	-0.815458	-3.754433	0.385782
H	0.372605	-3.340990	-0.849344
H	-3.113983	-2.952245	-2.596785
H	-1.801914	-3.272850	-3.723697
H	-2.315614	-4.656607	-1.236423
H	-0.853192	-4.840270	-2.188986

B3-TS4Spri

C	1.833591	0.378033	0.084633
C	1.331875	0.267729	1.570206
H	0.960831	1.246402	1.864326
C	2.188956	1.822343	-0.280234
C	2.031762	2.232614	-1.606911
C	2.695105	2.731122	0.649819
C	2.359377	3.527660	-1.989930
H	1.632818	1.527172	-2.323696
C	3.028741	4.029905	0.265632
H	2.825922	2.440457	1.685332
C	2.859477	4.433096	-1.054263
H	2.226119	3.832960	-3.021704
H	3.416113	4.724430	1.002282
H	3.114903	5.442964	-1.353454
C	3.051730	-0.496746	-0.262702
C	4.334704	-0.173347	0.192268
C	2.900725	-1.620175	-1.075082
C	5.422597	-0.988408	-0.101180
H	4.494927	0.732695	0.762390
C	3.990578	-2.433909	-1.378119
H	1.926231	-1.844202	-1.478747
C	5.253778	-2.130800	-0.881003
H	6.406327	-0.722951	0.268958
H	3.848351	-3.304072	-2.009096
H	6.101823	-2.764649	-1.112750
O	0.719112	-0.020153	-0.692282
B	-0.268758	-0.797630	0.005569
N	0.136477	-0.664122	1.563755
C	2.277024	-0.297963	2.626049
H	3.308928	0.019319	2.492762
H	1.938348	0.037492	3.608576
C	2.095491	-1.811720	2.492591
H	2.690491	-2.192466	1.661989
H	2.390586	-2.350286	3.394738
C	0.600872	-1.965676	2.211256
H	0.375125	-2.805778	1.564981
H	0.039261	-2.087191	3.134889
B	-1.000533	-0.063485	2.549892
H	-1.865859	-0.887391	2.686793
H	-0.467617	0.259524	3.585488
H	-1.483285	0.960292	2.036274
O	-1.689379	-0.106415	-0.123737
C	-2.054076	1.048837	0.217527
C	-1.214779	2.271361	-0.008746
H	-0.173480	2.128011	0.241416
H	-1.247935	2.489846	-1.081933
H	-1.621862	3.121588	0.536502
C	-3.532963	1.256194	0.425824
C	-4.147010	1.707882	-0.929406
C	-4.261062	0.017791	0.958163
H	-3.648418	2.083407	1.134419
C	-5.654931	1.946733	-0.793987
H	-3.960624	0.923183	-1.671198
H	-3.657370	2.616375	-1.289044
C	-5.765262	0.271380	1.090896
H	-4.087007	-0.813861	0.270523
H	-3.826273	-0.270697	1.915038
C	-6.379135	0.715741	-0.240526
H	-6.065832	2.228577	-1.768134
H	-5.822495	2.798168	-0.122862

H	-6.258834	-0.634619	1.453639
H	-5.941249	1.046245	1.847934
H	-7.445322	0.929345	-0.120228
H	-6.301170	-0.104189	-0.965227
C	-0.555639	-2.254834	-0.593480
C	-1.762642	-3.028128	0.035217
C	-0.874270	-2.181518	-2.114747
H	0.342470	-2.870234	-0.481228
C	-2.756266	-3.278845	-1.116992
H	-2.227928	-2.479109	0.854655
H	-1.427445	-3.985868	0.445600
C	-1.872701	-3.317728	-2.369594
H	0.023641	-2.252738	-2.733080
H	-1.348272	-1.224623	-2.357669
H	-3.352722	-4.183810	-0.975254
H	-3.453346	-2.437512	-1.196230
H	-1.344740	-4.277367	-2.422236
H	-2.434758	-3.199585	-3.300251

B4-TS4R

C	-1.261503	-0.626333	-0.035129
C	-0.830869	-0.003704	1.353440
H	0.179801	-0.331846	1.572166
C	-0.269331	-1.707840	-0.471463
C	0.034664	-1.843575	-1.826705
C	0.320729	-2.587060	0.439186
C	0.914884	-2.829191	-2.260568
H	-0.414943	-1.154463	-2.528483
C	1.198036	-3.580269	0.006328
H	0.104485	-2.502837	1.498119
C	1.500045	-3.703728	-1.346236
H	1.145261	-2.915973	-3.316473
H	1.646554	-4.252949	0.728663
H	2.185575	-4.471696	-1.685039
C	-2.685434	-1.199853	-0.046873
C	-2.974984	-2.455028	0.497024
C	-3.731763	-0.455558	-0.593555
C	-4.279684	-2.937855	0.522934
H	-2.178013	-3.071202	0.892846
C	-5.037784	-0.939844	-0.574386
H	-3.510120	0.498495	-1.047184
C	-5.319794	-2.179616	-0.009264
H	-4.481370	-3.912926	0.951616
H	-5.834531	-0.345490	-1.007412
H	-6.335609	-2.557151	0.006819
O	-1.217007	0.457788	-0.950817
B	-0.530663	1.589193	-0.438967
N	-0.764450	1.486396	1.133528
C	-1.742973	-0.194726	2.561928
H	-2.246942	-1.159055	2.550757
H	-1.137718	-0.136673	3.465832
C	-2.736884	0.983826	2.507457
H	-3.716386	0.650933	2.162874
H	-2.860031	1.433475	3.492686
C	-2.142010	1.989167	1.503003
H	-2.754014	2.034570	0.605939
H	-2.040177	2.991959	1.908918
B	0.309740	2.131004	2.145580
H	0.399247	3.310866	1.930969
H	-0.025109	1.885251	3.279334
H	1.385793	1.561422	1.942597
O	1.048118	1.091259	-0.483086
C	2.126199	1.385201	0.079580
C	2.683810	2.773091	0.098686
H	3.416164	2.832152	-0.716843
H	1.911746	3.514798	-0.060500
H	3.203231	2.976076	1.031960
C	3.027641	0.214800	0.389202
C	3.648663	-0.285498	-0.942237
C	4.113385	0.479827	1.437632
H	2.368531	-0.583464	0.741670
C	4.512122	-1.527818	-0.699175
H	4.264512	0.510896	-1.378135
H	2.846628	-0.511000	-1.646935
C	4.953427	-0.779914	1.675736
H	4.774753	1.283315	1.093743
H	3.651501	0.818147	2.369048
C	5.580889	-1.283539	0.370972
H	4.976542	-1.836431	-1.640708
H	3.860278	-2.349730	-0.386673
H	5.729465	-0.572956	2.418128
H	4.314678	-1.564849	2.098799
H	6.149092	-2.200686	0.552246
H	6.297914	-0.537135	0.005864
C	-0.787055	2.921520	-1.337896
C	-2.198869	2.858491	-1.968542
H	-2.991634	2.898366	-1.216841
H	-2.346845	3.716589	-2.633866
H	-2.332407	1.946463	-2.551282
C	0.209818	2.919560	-2.520070

H	-0.047747	3.712603	-3.230882
H	1.238635	3.090134	-2.199458
H	0.183933	1.966898	-3.055865
C	-0.685957	4.270265	-0.601592
H	0.271008	4.424888	-0.104103
H	-0.824966	5.092203	-1.313564
H	-1.456382	4.371108	0.165607

B2-TS5R

C	1.465507	0.000436	-0.077181
C	0.967456	0.230600	1.406078
H	0.692415	1.274865	1.507865
C	1.821747	1.339188	-0.725523
C	1.453802	1.575754	-2.050088
C	2.503851	2.339357	-0.028314
C	1.752707	2.789792	-2.661472
H	0.913010	0.804665	-2.581101
C	2.809811	3.552605	-0.640202
H	2.780028	2.190630	1.009228
C	2.432169	3.783594	-1.959790
H	1.452240	2.961074	-3.689106
H	3.332748	4.319864	-0.080754
H	2.662318	4.729671	-2.435846
C	2.654015	-0.969313	-0.205123
C	3.960543	-0.586095	0.112887
C	2.437912	-2.275023	-0.649620
C	5.011827	-1.494361	0.033517
H	4.170476	0.433038	0.409079
C	3.489006	-3.184604	-0.736274
H	1.438712	-2.562843	-0.942540
C	4.780054	-2.802216	-0.385175
H	6.015584	-1.174808	0.289573
H	3.296379	-4.192987	-1.085095
H	5.599015	-3.509098	-0.450703
O	0.360801	-0.580742	-0.744558
B	-0.831082	-0.637488	0.039814
N	-0.299110	-0.575930	1.540671
C	1.858812	-0.232693	2.553379
H	2.916279	-0.084189	2.344944
H	1.604361	0.338288	3.445505
C	1.507529	-1.721425	2.748604
H	2.279181	-2.362310	2.321443
H	1.414585	-1.963970	3.807367
C	0.174938	-1.937450	2.005712
H	0.318394	-2.576677	1.136792
H	-0.597336	-2.369641	2.635205
B	-1.264275	0.046860	2.662187
H	-2.222444	-0.670812	2.790714
H	-0.649694	0.209732	3.687094
H	-1.625387	1.170196	2.266860
O	-1.444415	0.855468	-0.123517
C	-2.349575	1.468473	0.497488
C	-3.666078	0.833152	0.821445
H	-4.359141	1.097448	0.013570
H	-3.577962	-0.242061	0.872863
H	-4.071530	1.208388	1.756604
C	-1.772802	-1.829072	-0.506360
C	-2.724645	-2.624571	0.408387
C	-2.505570	-1.397212	-1.796094
H	-1.023925	-2.565217	-0.836500
C	-3.334638	-3.823393	-0.332015
H	-3.548470	-2.003696	0.772615
H	-2.202624	-2.972794	1.301092
C	-3.139320	-2.584950	-2.529296
H	-3.293066	-0.675189	-1.547154
H	-1.807872	-0.875951	-2.457745
C	-4.065157	-3.384807	-1.606112
H	-4.018373	-4.366369	0.328904
H	-2.533756	-4.524500	-0.600269
H	-3.689203	-2.238807	-3.411121
H	-2.341613	-3.243493	-2.895640
H	-4.471171	-4.254605	-2.132514
H	-4.923667	-2.758995	-1.328017
C	-2.258896	2.994821	0.480193
C	-0.809293	3.462305	0.654917
H	-0.156211	3.021306	-0.093798
H	-0.763700	4.548997	0.552886
H	-0.437577	3.198022	1.646835
C	-2.746505	3.409039	-0.933913
H	-2.699472	4.497124	-1.018305
H	-2.106104	2.972840	-1.701943
H	-3.778847	3.101987	-1.117463
C	-3.150134	3.641593	1.550101
H	-4.211006	3.446305	1.382593
H	-2.882883	3.285972	2.547833
H	-3.007647	4.724267	1.527126

B2-TS5Rpri

C	1.466265	0.004962	-0.078655

C	0.967303	0.235720	1.404160
H	0.687366	1.278898	1.503687
C	1.814397	1.344145	-0.730910
C	1.445618	1.573639	-2.056378
C	2.488456	2.351660	-0.036512
C	1.735068	2.788096	-2.671475
H	0.911820	0.796452	-2.585586
C	2.785549	3.565191	-0.652328
H	2.766285	2.208188	1.001379
C	2.406265	3.789426	-1.972584
H	1.433559	2.953685	-3.699744
H	3.302775	4.338059	-0.095240
H	2.629119	4.735931	-2.451252
C	2.660013	-0.958511	-0.203761
C	3.964930	-0.563817	0.106707
C	2.451468	-2.269584	-0.635889
C	5.022199	-1.465484	0.031856
H	4.168425	0.459119	0.394191
C	3.508471	-3.172637	-0.718022
H	1.453105	-2.568471	-0.920963
C	4.798064	-2.778441	-0.374825
H	6.024549	-1.136809	0.281841
H	3.321484	-4.185473	-1.056863
H	5.621605	-3.480284	-0.437057
O	0.364163	-0.584056	-0.744049
B	-0.827603	-0.641065	0.040525
N	-0.296071	-0.575884	1.541027
C	1.860480	-0.222614	2.552316
H	2.917366	-0.070937	2.343189
H	1.603818	0.348713	3.443567
C	1.514628	-1.712704	2.749672
H	2.289448	-2.351295	2.325021
H	1.421164	-1.953415	3.808799
C	0.183630	-1.934844	2.005912
H	0.329367	-2.572738	1.136475
H	-0.587597	-2.370918	2.634287
B	-1.264276	0.041268	2.662270
H	-2.220752	-0.679771	2.785021
H	-0.651965	0.201788	3.688936
H	-1.627057	1.164479	2.269548
O	-1.443737	0.852148	-0.121874
C	-2.353041	1.458649	0.499277
C	-3.666546	0.814943	0.818567
H	-4.361494	1.085333	0.014529
H	-3.573754	-0.260353	0.857781
H	-4.072561	1.178350	1.758192
C	-1.766923	-1.834069	-0.506508
C	-2.714709	-2.638061	0.404912
C	-2.502595	-1.401780	-1.794640
H	-1.014822	-2.565524	-0.839797
C	-3.315057	-3.839240	-0.339767
H	-3.543877	-2.024128	0.769205
H	-2.192679	-2.985937	1.297904
C	-3.123642	-2.592792	-2.533220
H	-3.297090	-0.688447	-1.542780
H	-1.809416	-0.870942	-2.453361
C	-4.044896	-3.403751	-1.615421
H	-3.997184	-4.387627	0.318349
H	-2.509155	-4.535152	-0.606658
H	-3.674002	-2.249438	-3.415807
H	-2.318341	-3.242649	-2.898573
H	-4.440531	-4.276089	-2.145505
H	-4.910566	-2.786944	-1.339334
C	-2.272547	2.985451	0.487944
C	-0.824412	3.461544	0.648602
H	-0.440780	3.195485	1.635379
H	-0.176363	3.027367	-0.108546
H	-0.786626	4.548980	0.550899
C	-2.780120	3.405069	-0.917696
H	-3.812404	3.091934	-1.090978
H	-2.741584	4.494090	-0.994551
H	-2.146137	2.979703	-1.697140
C	-3.156645	3.619969	1.571431
H	-3.021669	4.703754	1.553420
H	-4.217810	3.418036	1.413889
H	-2.876576	3.259629	2.563886

B2-TS5S

C	1.498495	0.261181	-0.027950
C	0.843628	0.483671	1.383225
H	0.555426	1.526258	1.462350
C	2.004714	1.576832	-0.621987
C	1.927913	1.766984	-2.003089
C	2.559263	2.586869	0.167886
C	2.381826	2.948606	-2.579676
H	1.488490	0.986690	-2.609560
C	3.016637	3.769835	-0.408963
H	2.621991	2.467527	1.243284
C	2.927202	3.955600	-1.785237

H	2.308682	3.084776	-3.652716
H	3.437417	4.546485	0.219410
H	3.278543	4.876830	-2.235182
C	2.664492	-0.744891	-0.018315
C	3.935952	-0.381404	0.439702
C	2.472907	-2.051691	-0.462169
C	4.968773	-1.311583	0.495139
H	4.134261	0.638784	0.740493
C	3.506531	-2.984596	-0.414379
H	1.512237	-2.330284	-0.859465
C	4.757298	-2.622663	0.074507
H	5.943823	-1.007039	0.858048
H	3.329866	-3.994826	-0.766179
H	5.562888	-3.346605	0.113894
O	0.441288	-0.239212	-0.837535
B	-0.794924	-0.481111	-0.171364
N	-0.426885	-0.328416	1.382473
C	1.608610	0.043924	2.628427
H	2.682556	0.184311	2.527261
H	1.265735	0.639192	3.473615
C	1.232799	-1.436831	2.826045
H	2.039487	-2.088852	2.490281
H	1.040292	-1.653010	3.877127
C	-0.022496	-1.669887	1.966207
H	0.193329	-2.357738	1.153286
H	-0.864320	-2.056965	2.532658
B	-1.501184	0.354419	2.362176
H	-2.466418	-0.350896	2.456868
H	-0.983523	0.618882	3.418959
H	-1.836472	1.437157	1.836948
O	-1.904423	0.717847	-0.419150
C	-2.115554	1.851848	0.084321
C	-1.067047	2.933263	0.040334
H	-0.981941	3.459770	0.988811
H	-0.105886	2.538024	-0.261512
H	-1.375504	3.662464	-0.714916
C	-1.654971	-1.721884	-0.754690
C	-0.869749	-2.897186	-1.366157
C	-2.761876	-2.266695	0.167656
H	-2.175682	-1.257456	-1.605982
C	-1.793756	-3.875900	-2.104264
H	-0.337701	-3.452866	-0.582489
H	-0.113580	-2.515583	-2.056551
C	-3.696162	-3.235716	-0.566265
H	-2.309174	-2.801395	1.010594
H	-3.333892	-1.448669	0.602729
C	-2.913912	-4.393671	-1.195852
H	-1.214107	-4.713495	-2.507100
H	-2.239389	-3.360052	-2.964563
H	-4.457268	-3.622609	0.119946
H	-4.232027	-2.691081	-1.354826
H	-3.585310	-5.052943	-1.755824
H	-2.473321	-5.002741	-0.395431
C	-3.749020	3.406754	1.251546
H	-4.804314	3.681234	1.317016
H	-3.421952	3.070944	2.237714
H	-3.190469	4.306888	0.987077
C	-3.579397	2.289400	0.211295
C	-4.484843	1.102708	0.557742
H	-4.408131	0.320166	-0.196150
H	-4.213047	0.677186	1.522900
H	-5.523092	1.440947	0.600415
C	-3.979880	2.817730	-1.192253
H	-5.040842	3.077465	-1.179909
H	-3.421134	3.712233	-1.475024
H	-3.822969	2.050547	-1.952898

B2-TS5Spri

C	1.531183	0.220608	0.001780
C	0.971238	0.523606	1.430830
H	0.723519	1.580836	1.465670
C	2.201245	1.450080	-0.614178
C	2.196576	1.586121	-2.004927
C	2.837853	2.424611	0.155870
C	2.804608	2.679904	-2.610138
H	1.694258	0.833743	-2.598413
C	3.452288	3.519621	-0.450521
H	2.852475	2.346933	1.236258
C	3.435804	3.652039	-1.834875
H	2.786792	2.775134	-3.689919
H	3.938545	4.269730	0.162723
H	3.909502	4.504842	-2.307034
C	2.530675	-0.947029	-0.052040
C	3.848087	-0.794437	0.394103
C	2.143368	-2.183761	-0.565774
C	4.732372	-1.867636	0.386272
H	4.196561	0.173215	0.731377
C	3.029200	-3.259250	-0.581490
H	1.148541	-2.291884	-0.968585

C	4.323055	-3.110686	-0.092752
H	5.746705	-1.729150	0.742754
H	2.705409	-4.212568	-0.983727
H	5.012626	-3.946760	-0.102291
O	0.375032	-0.097054	-0.759492
B	-0.788722	-0.455714	0.007584
N	-0.328582	-0.234169	1.538351
C	1.783416	0.115898	2.655655
H	2.854851	0.237198	2.510002
H	1.480990	0.742809	3.494148
C	1.394709	-1.348372	2.910583
H	2.118384	-2.019337	2.447622
H	1.363622	-1.572061	3.977464
C	0.014250	-1.532254	2.254973
H	0.032364	-2.349371	1.540733
H	-0.772072	-1.727316	2.979456
B	-1.342772	0.586997	2.479588
H	-2.339432	-0.062272	2.640896
H	-0.782215	0.895717	3.502565
H	-1.634795	1.641417	1.879301
O	-1.985508	0.579967	-0.228329
C	-2.078537	1.799226	0.076988
C	-0.968474	2.770860	-0.218232
H	-0.058393	2.260466	-0.501958
H	-1.287382	3.379225	-1.070693
H	-0.787512	3.446169	0.615165
C	-1.466815	-1.849286	-0.418961
C	-2.725523	-2.254250	0.367986
C	-1.775262	-1.836906	-1.931338
H	-0.728742	-2.649405	-0.267874
C	-3.300831	-3.592470	-0.112084
H	-3.482948	-1.474380	0.248326
H	-2.515953	-2.304657	1.439585
C	-2.353554	-3.170157	-2.419298
H	-2.497075	-1.036660	-2.138721
H	-0.869252	-1.587750	-2.491453
C	-3.596300	-3.569528	-1.616121
H	-4.209550	-3.837898	0.448044
H	-2.577970	-4.391903	0.098953
H	-2.593787	-3.113021	-3.486574
H	-1.590958	-3.952941	-2.312178
H	-3.969672	-4.543938	-1.948177
H	-4.397002	-2.844068	-1.811787
C	-4.429205	1.311121	0.872663
H	-5.435331	1.731251	0.946218
H	-4.475824	0.410864	0.261538
H	-4.089722	1.027118	1.867445
C	-3.493057	2.353377	0.249313
C	-3.993357	2.668387	-1.186503
H	-5.026833	3.017107	-1.127564
H	-3.403666	3.450501	-1.669036
H	-3.967871	1.773579	-1.811335
C	-3.497494	3.638357	1.091172
H	-2.920074	4.440416	0.627230
H	-4.524825	3.992721	1.200361
H	-3.094030	3.449048	2.088015

B3-TS5R

C	1.392399	0.174385	-0.093459
C	0.804574	0.104487	1.373830
H	0.309373	1.046976	1.582148
C	1.453364	1.627800	-0.565985
C	1.084326	1.936724	-1.875140
C	1.864892	2.661992	0.278237
C	1.116146	3.252465	-2.327665
H	0.751010	1.134980	-2.519485
C	1.904350	3.978605	-0.174601
H	2.133692	2.452840	1.307267
C	1.526632	4.279466	-1.479941
H	0.817540	3.476743	-3.345602
H	2.218983	4.768884	0.497447
H	1.548834	5.304172	-1.832313
C	2.774496	-0.483387	-0.254998
C	3.948831	0.144299	0.171582
C	2.875777	-1.746780	-0.840425
C	5.181840	-0.490557	0.055770
H	3.911809	1.144352	0.582800
C	4.108961	-2.382172	-0.963351
H	1.977493	-2.217517	-1.212810
C	5.267741	-1.761591	-0.506928
H	6.077967	0.014954	0.397296
H	4.161993	-3.362934	-1.422614
H	6.228281	-2.254858	-0.600491
O	0.474045	-0.554254	-0.888828
B	-0.708534	-0.943990	-0.194924
N	-0.264455	-0.956615	1.335957
C	1.735830	-0.305258	2.510503
H	2.745676	0.077177	2.377592
H	1.341956	0.091297	3.445374

C	1.693821	-1.847335	2.519247
H	2.600519	-2.263026	2.078897
H	1.606759	-2.227925	3.537063
C	0.470106	-2.238331	1.668731
H	0.782857	-2.715503	0.741985
H	-0.219056	-2.899442	2.185880
B	-1.379311	-0.691438	2.465269
H	-2.164722	-1.604364	2.454630
H	-0.842449	-0.546145	3.535861
H	-1.955439	0.370881	2.189068
O	-1.612993	0.408000	-0.221976
C	-2.671348	0.718701	0.375701
C	-3.814098	-0.236596	0.528233
H	-4.534196	-0.007704	-0.266506
H	-3.489271	-1.261229	0.422126
H	-4.312089	-0.109949	1.485296
C	-2.937031	2.213592	0.543225
C	-1.641759	2.968362	0.865682
H	-1.252601	2.662493	1.838639
H	-0.878705	2.790612	0.111987
H	-1.847130	4.040683	0.901691
C	-3.457681	2.686716	-0.840577
H	-4.382348	2.179351	-1.125530
H	-3.663587	3.758401	-0.791940
H	-2.706057	2.512947	-1.612225
C	-3.992687	2.495408	1.622173
H	-4.106510	3.575522	1.737387
H	-4.971756	2.087718	1.363368
H	-3.685596	2.081919	2.585432
C	-1.381857	-2.226745	-0.898540
C	-2.104571	-3.337597	-0.094810
C	-2.343508	-1.895332	-2.088944
H	-0.517251	-2.726654	-1.351337
C	-2.943842	-4.073121	-1.149280
H	-2.749510	-2.940244	0.692336
H	-1.397267	-4.001909	0.405305
C	-3.483931	-2.941228	-2.036559
H	-1.800641	-1.955543	-3.034673
H	-2.739009	-0.879683	-2.033498
H	-2.294803	-4.729956	-1.739410
H	-3.734009	-4.693649	-0.718523
H	-3.785492	-3.286159	-3.028371
H	-4.373904	-2.506872	-1.568816

B3-TS5S

C	1.371830	0.361974	-0.029770
C	0.761039	0.442786	1.431507
H	0.237463	1.387171	1.526401
C	1.340568	1.742769	-0.687401
C	0.963310	1.858170	-2.025074
C	1.679675	2.900452	0.018800
C	0.920291	3.104875	-2.642046
H	0.684932	0.963169	-2.563575
C	1.642507	4.148414	-0.598643
H	1.957259	2.840316	1.065186
C	1.259903	4.254967	-1.932969
H	0.616671	3.178356	-3.680219
H	1.904964	5.035745	-0.033969
H	1.224130	5.225251	-2.414468
C	2.796704	-0.211239	-0.097745
C	3.915629	0.549108	0.257453
C	2.999161	-1.524325	-0.524457
C	5.193879	0.000646	0.221967
H	3.798966	1.584128	0.549491
C	4.277947	-2.074949	-0.564970
H	2.146557	-2.102372	-0.847007
C	5.381311	-1.318213	-0.184334
H	6.045161	0.609747	0.503730
H	4.408762	-3.096929	-0.902375
H	6.377054	-1.744922	-0.215782
O	0.509189	-0.519463	-0.738652
B	-0.650875	-0.876694	-0.025555
N	-0.275377	-0.646962	1.490435
C	1.677714	0.211442	2.636388
H	2.707623	0.487024	2.422079
H	1.330476	0.824899	3.467074
C	1.538374	-1.288848	2.984137
H	2.484804	-1.819473	2.871427
H	1.201057	-1.411276	4.013023
C	0.503368	-1.841670	1.997243
H	1.003667	-2.312357	1.155529
H	-0.193541	-2.550521	2.436122
B	-1.439639	-0.312734	2.537159
H	-2.143250	-1.282786	2.652091
H	-0.962120	0.066591	3.578044
H	-2.095355	0.628934	2.068275
O	-1.805674	0.352036	-0.297697
C	-2.490295	1.180060	0.347304
C	-1.953810	2.578357	0.552125

H	-2.099243	2.921610	1.574744
H	-0.904670	2.624665	0.279100
H	-2.504217	3.256930	-0.106511
C	-4.613612	1.762054	1.623057
H	-5.692997	1.594938	1.649518
H	-4.192852	1.423669	2.572024
H	-4.449217	2.837604	1.532616
C	-4.007619	0.978475	0.449605
C	-4.376120	-0.503898	0.562882
H	-4.005049	-1.067430	-0.291233
H	-3.960814	-0.939245	1.470585
H	-5.464215	-0.602117	0.591584
C	-4.574951	1.523995	-0.889390
H	-5.653941	1.354757	-0.907975
H	-4.402293	2.595804	-1.006589
H	-4.130410	1.003200	-1.739631
C	-1.399364	-2.192539	-0.513087
C	-1.897429	-2.082762	-1.970228
C	-0.591492	-3.510430	-0.568994
H	-2.270052	-2.365205	0.130047
C	-2.325890	-3.512259	-2.336228
H	-1.065748	-1.759302	-2.606125
H	-2.698537	-1.352321	-2.094275
C	-1.358426	-4.436732	-1.550520
H	-0.465443	-3.975326	0.409942
H	0.408554	-3.309037	-0.963766
H	-3.355565	-3.679662	-2.005201
H	-2.302443	-3.698050	-3.412822
H	-1.911999	-5.207733	-1.009085
H	-0.667283	-4.957245	-2.217945

B3-TS5Spri

C	1.458449	0.248913	-0.007547
C	0.831853	0.311044	1.426265
H	0.432087	1.312334	1.555631
C	1.970258	1.618734	-0.455177
C	1.967984	1.919823	-1.819656
C	2.464741	2.565620	0.443175
C	2.439633	3.146247	-2.273360
H	1.577176	1.186430	-2.512499
C	2.942593	3.794178	-0.011535
H	2.475091	2.359801	1.506801
C	2.929761	4.089260	-1.370616
H	2.426219	3.367633	-3.334575
H	3.319846	4.519877	0.699959
H	3.297851	5.045068	-1.724943
C	2.606102	-0.764089	-0.166914
C	3.878955	-0.511827	0.357367
C	2.407653	-1.946203	-0.879722
C	4.902445	-1.444980	0.228915
H	4.086858	0.431823	0.845263
C	3.432700	-2.880049	-1.016195
H	1.448953	-2.117802	-1.344154
C	4.680813	-2.640965	-0.450540
H	5.878847	-1.230069	0.647970
H	3.253531	-3.792099	-1.574359
H	5.478937	-3.366775	-0.554427
O	0.365948	-0.120492	-0.838096
B	-0.743567	-0.734798	-0.153939
N	-0.344721	-0.628475	1.400386
C	1.666742	-0.094786	2.644262
H	2.731608	0.034598	2.471892
H	1.385311	0.532915	3.488885
C	1.306042	-1.570321	2.930937
H	2.168120	-2.225619	2.798964
H	0.950686	-1.682115	3.955090
C	0.208544	-1.933953	1.921902
H	0.632504	-2.496298	1.094107
H	-0.608991	-2.507986	2.350131
B	-1.468418	-0.097195	2.419011
H	-2.343029	-0.915538	2.509306
H	-0.958779	0.196963	3.472098
H	-1.936445	0.951237	1.930997
O	-2.076291	0.132528	-0.310853
C	-2.363311	1.273907	0.138194
C	-1.410154	2.430012	0.004821
H	-0.429479	2.097967	-0.309096
H	-1.804790	3.087894	-0.776311
H	-1.346672	3.012515	0.921358
C	-4.612430	0.332012	0.801818
H	-5.675204	0.573052	0.882694
H	-4.498222	-0.491927	0.098764
H	-4.250284	0.001015	1.774269
C	-3.849217	1.575300	0.328044
C	-4.374146	1.971809	-1.078424
H	-5.450255	2.147123	-1.013272
H	-3.908056	2.886151	-1.451677
H	-4.199247	1.169983	-1.798045
C	-4.068972	2.735960	1.309690

H	-3.620726	3.667433	0.958294
H	-5.140844	2.909611	1.427305
H	-3.652676	2.496394	2.290337
C	-1.208665	-2.147064	-0.750506
C	-2.403783	-2.840336	-0.033747
C	-1.662755	-1.981098	-2.241080
H	-0.360407	-2.835165	-0.738249
C	-3.317325	-3.364516	-1.154969
H	-2.949989	-2.123539	0.580386
H	-2.074591	-3.639662	0.634710
C	-3.164500	-2.311951	-2.259311
H	-1.122811	-2.686722	-2.880223
H	-1.453969	-0.982964	-2.631812
H	-2.952582	-4.332236	-1.518949
H	-4.352413	-3.506424	-0.831779
H	-3.514224	-2.655434	-3.236690
H	-3.743270	-1.420528	-1.997169

B4-TS5R

C	1.131781	0.397082	-0.039903
C	0.500139	0.007218	1.353760
H	-0.273640	0.731269	1.583313
C	0.781488	1.842435	-0.397346
C	0.414618	2.154032	-1.706296
C	0.822214	2.866930	0.551652
C	0.085639	3.460155	-2.057055
H	0.369558	1.355601	-2.433722
C	0.500964	4.175836	0.201325
H	1.077147	2.646600	1.581828
C	0.128084	4.477026	-1.105505
H	-0.206450	3.684189	-3.076909
H	0.530273	4.956109	0.953223
H	-0.130265	5.493410	-1.379077
C	2.658653	0.213285	-0.118150
C	3.542985	1.118086	0.479180
C	3.196837	-0.878798	-0.799211
C	4.918137	0.911436	0.435281
H	3.165545	2.005385	0.968866
C	4.573567	-1.085398	-0.850885
H	2.527650	-1.556194	-1.303478
C	5.442008	-0.197167	-0.225213
H	5.581202	1.625843	0.909823
H	4.964549	-1.941938	-1.388638
H	6.513371	-0.356517	-0.263160
O	0.526640	-0.488020	-0.968569
B	-0.517857	-1.288756	-0.429085
N	-0.187225	-1.320026	1.138372
C	1.430191	-0.211261	2.543446
H	2.286777	0.458841	2.528828
H	0.872928	-0.028282	3.460981
C	1.850920	-1.692495	2.456006
H	2.875548	-1.786528	2.094909
H	1.791636	-2.171081	3.433614
C	0.881000	-2.345170	1.453497
H	1.402707	-2.602329	0.535298
H	0.395615	-3.235402	1.842937
B	-1.387799	-1.491192	2.190571
H	-1.884076	-2.578420	2.057029
H	-0.984571	-1.288581	3.308370
H	-2.226896	-0.604506	1.943956
O	-1.809909	-0.271624	-0.402050
C	-2.916940	-0.292729	0.196598
C	-3.801715	-1.504181	0.236988
H	-4.535602	-1.398557	-0.570721
H	-3.241163	-2.413936	0.085028
H	-4.340032	-1.566038	1.178260
C	-3.575136	1.068327	0.446258
C	-2.545705	2.108960	0.896026
H	-3.027290	3.086210	0.976668
H	-2.137893	1.850309	1.875226
H	-1.729152	2.189650	0.184644
C	-4.714012	0.987286	1.472953
H	-4.354560	0.587675	2.424040
H	-5.102614	1.991890	1.654128
H	-5.546260	0.371047	1.127941
C	-4.138365	1.498983	-0.934311
H	-3.332758	1.582906	-1.665064
H	-4.885562	0.796441	-1.310773
H	-4.617392	2.475243	-0.830467
C	-0.784639	-2.623616	-1.342791
C	-1.235130	-3.897204	-0.595896
H	-1.519253	-4.669786	-1.319553
H	-0.423852	-4.308249	0.008334
H	-2.076361	-3.751919	0.079751
C	0.508558	-3.013636	-2.097973
H	0.307817	-3.867421	-2.754986
H	0.877720	-2.190650	-2.710418
H	1.307835	-3.317442	-1.417695
C	-1.814834	-2.283455	-2.446664

H	-1.524456	-1.378762	-2.987577
H	-1.869484	-3.103720	-3.170818
H	-2.821118	-2.127075	-2.059640

B4-TS5S

C	-1.256405	0.339271	0.019483
C	-0.545218	0.070643	-1.355166
H	0.066837	0.933390	-1.590871
C	-1.321395	1.835500	0.332605
C	-1.213879	2.251265	1.660970
C	-1.505754	2.798412	-0.662518
C	-1.272901	3.602698	1.985021
H	-1.062395	1.503280	2.427275
C	-1.567334	4.152142	-0.338881
H	-1.583139	2.502087	-1.702269
C	-1.448257	4.559097	0.986661
H	-1.179883	3.910786	3.020259
H	-1.703029	4.886822	-1.124335
H	-1.491254	5.611987	1.239866
C	-2.686959	-0.225735	0.099598
C	-3.761484	0.429515	-0.513276
C	-2.945244	-1.408624	0.787902
C	-5.043451	-0.108815	-0.475928
H	-3.607584	1.378268	-1.009809
C	-4.228972	-1.948441	0.833404
H	-2.136245	-1.897110	1.301399
C	-5.283697	-1.306944	0.193223
H	-5.858111	0.416199	-0.961599
H	-4.400558	-2.870199	1.377915
H	-6.283279	-1.724421	0.226734
O	-0.425754	-0.302827	0.981007
B	0.691879	-1.024161	0.464119
N	0.394462	-1.086571	-1.118940
C	-1.390720	-0.326355	-2.563951
H	-2.373665	0.139002	-2.551974
H	-0.875401	-0.006424	-3.468456
C	-1.476350	-1.864977	-2.511290
H	-2.466461	-2.189671	-2.189574
H	-1.280431	-2.297756	-3.492304
C	-0.422987	-2.308234	-1.480489
H	-0.912703	-2.678760	-0.586708
H	0.252806	-3.072652	-1.853301
B	1.628732	-0.982454	-2.138476
H	2.336550	-1.941831	-2.014256
H	1.222974	-0.814182	-3.261223
H	2.270143	0.046645	-1.830216
O	2.099119	-0.178839	0.511516
C	2.646903	0.711716	-0.189824
C	1.983236	2.048730	-0.407188
H	1.998853	2.347391	-1.453737
H	0.969138	2.047965	-0.029612
H	2.546259	2.798972	0.154681
C	1.108601	-2.327329	1.364696
C	4.665542	1.448128	-1.541036
H	5.755017	1.389093	-1.594594
H	4.255693	1.023739	-2.459938
H	4.396002	2.505060	-1.495300
C	4.175443	0.665057	-0.313425
C	4.696218	-0.774547	-0.369858
H	4.414806	-1.329298	0.523881
H	4.301064	-1.298847	-1.238820
H	5.787069	-0.758813	-0.433643
C	4.719875	1.326823	0.980989
H	5.809759	1.253929	0.979144
H	4.456786	2.383900	1.051504
H	4.343482	0.812281	1.867100
C	1.830324	-1.811097	2.635116
H	2.008191	-2.642136	3.326815
H	2.793205	-1.357664	2.401412
H	1.224371	-1.065060	3.157744
C	-0.089224	-3.145198	1.889802
H	-0.738773	-2.535141	2.520403
H	-0.692993	-3.592045	1.097198
H	0.277901	-3.973457	2.506472
C	2.054055	-3.297060	0.632633
H	2.902872	-2.781901	0.188451
H	2.440361	-4.048057	1.332294
H	1.544770	-3.836828	-0.169461

B4-TS5Spri

C	1.256442	0.339294	-0.019516
C	0.545296	0.070603	1.355155
H	-0.066787	0.933316	1.590916
C	1.321200	1.835526	-0.332661
C	1.213337	2.251290	-1.660997
C	1.505642	2.798455	0.662433
C	1.272109	3.602736	-1.985047
H	1.061780	1.503291	-2.427273

C	1.566970	4.152196	0.338797
H	1.583268	2.502131	1.702166
C	1.447553	4.559148	-0.986716
H	1.178824	3.910821	-3.020262
H	1.702731	4.886887	1.124230
H	1.490353	5.612046	-1.239920
C	2.687057	-0.225552	-0.099636
C	3.761525	0.429814	0.513209
C	2.945437	-1.408459	-0.787883
C	5.043536	-0.108417	0.475893
H	3.607547	1.378572	1.009706
C	4.229208	-1.948172	-0.833352
H	2.136474	-1.897051	-1.301340
C	5.283879	-1.306558	-0.193197
H	5.858151	0.416684	0.961545
H	4.400872	-2.869945	-1.377813
H	6.283494	-1.723959	-0.226680
O	0.425897	-0.302958	-0.981018
B	-0.691763	-1.024197	-0.464108
N	-0.394329	-1.086657	1.118928
C	1.390859	-0.326421	2.563876
H	2.373681	0.139208	2.552056
H	0.875401	-0.006838	3.468424
C	1.476896	-1.864992	2.510836
H	2.466957	-2.189289	2.188575
H	1.281578	-2.298074	3.491837
C	0.423186	-2.308319	1.480403
H	0.912595	-2.679015	0.586526
H	-0.252546	-3.072633	1.853535
B	-1.628586	-0.982691	2.138516
H	-2.336351	-1.942098	2.014212
H	-1.222788	-0.814569	3.261273
H	-2.270040	0.046411	1.830412
O	-2.098933	-0.178667	-0.511463
C	-2.646886	0.711691	0.189977
C	-1.983422	2.048755	0.407661
H	-0.969276	2.048203	0.030212
H	-2.546480	2.799026	-0.154137
H	-1.999214	2.347215	1.454266
C	-1.108573	-2.327297	-1.364730
C	-4.695987	-0.774907	0.369569
H	-5.786833	-0.759348	0.433486
H	-4.414601	-1.329339	-0.524375
H	-4.300660	-1.299422	1.238324
C	-4.175422	0.664787	0.313534
C	-4.719916	1.326830	-0.980714
H	-5.809778	1.253596	-0.979013
H	-4.457150	2.384015	-1.050823
H	-4.343274	0.812718	-1.866969
C	-4.665673	1.447462	1.541337
H	-4.396244	2.504435	1.495900
H	-5.755145	1.388294	1.594816
H	-4.255827	1.022865	2.460142
C	0.089280	-3.145002	-1.890040
H	0.738773	-2.534788	-2.520546
H	0.693108	-3.591955	-1.097538
H	-0.277813	-3.973177	-2.506841
C	-2.053846	-3.297179	-0.632637
H	-2.440110	-4.048190	-1.332307
H	-1.544432	-3.836923	0.169390
H	-2.902697	-2.782155	-0.188364
C	-1.830488	-1.811031	-2.635024
H	-2.008396	-2.642039	-3.326749
H	-2.793363	-1.357665	-2.401172
H	-1.224647	-1.064928	-3.157687

C1-B5-TS1R

C	2.175038	1.340943	-0.385565
C	1.783504	0.371058	0.788381
H	0.856084	0.724357	1.243347
O	1.801213	0.660653	-1.588934
B	1.049653	-0.506691	-1.329428
N	1.482340	-0.954973	0.137631
C	2.850700	0.063846	1.849203
H	3.543212	0.897003	1.980565
H	2.366255	-0.113444	2.810443
C	3.555290	-1.228631	1.361013
H	4.622218	-1.075552	1.177281
H	3.457563	-2.017930	2.108941
C	2.830377	-1.622185	0.063612
H	3.355265	-1.242179	-0.815554
H	2.685707	-2.695115	-0.052120
B	0.488863	-1.859903	1.005627
H	0.508954	-2.987958	0.569609
H	0.769152	-1.783682	2.179788
H	-0.658221	-1.393564	0.881688
O	-0.454276	-0.067969	-1.131257
C	-1.406676	-0.841555	-0.801544
C	-2.599383	-0.195726	-0.208613

C	-3.743014	-0.934285	0.132218
C	-2.590904	1.192258	0.004105
C	-4.852976	-0.296534	0.677857
H	-3.766658	-2.006995	-0.015934
C	-3.702340	1.826582	0.545682
C	-4.835372	1.083549	0.885671
H	-3.687638	2.900189	0.703333
H	-5.702228	1.578921	1.311865
C	-1.506960	-2.235070	-1.356170
H	-2.131114	-2.176945	-2.257518
H	-0.527011	-2.616030	-1.623866
H	-1.973687	-2.918929	-0.650108
H	-1.709116	1.756253	-0.271065
H	-5.730948	-0.876411	0.942999
C	1.383807	2.647959	-0.269571
H	0.311665	2.441352	-0.271394
H	1.634286	3.179499	0.655489
H	1.608803	3.301192	-1.117050
C	3.669500	1.663476	-0.499467
H	3.831954	2.201815	-1.436737
H	4.014339	2.297836	0.322288
H	4.282791	0.760530	-0.522612
H	1.130370	-1.372009	-2.157452

C1-B5-TS1Rpri

C	-1.194961	-1.455136	-0.419567
C	-1.426992	-0.437439	0.723458
H	-0.504017	-0.385963	1.304409
O	-0.526739	-0.682906	-1.421724
B	-0.877682	0.698980	-1.361770
N	-1.595889	0.899500	0.052951
C	-2.647022	-0.610683	1.627596
H	-2.921107	-1.662740	1.743239
H	-2.414196	-0.220826	2.619645
C	-3.768546	0.233216	0.975956
H	-4.534384	-0.401919	0.523022
H	-4.263352	0.857214	1.722818
C	-3.083917	1.094245	-0.105981
H	-3.382169	0.787427	-1.109428
H	-3.297321	2.158168	-0.007311
B	-1.039284	2.072392	1.008718
H	-1.150662	3.130100	0.431365
H	-1.640907	2.026926	2.060215
H	0.152726	1.823463	1.224093
O	0.399122	1.648409	-1.346555
C	1.341528	1.706730	-0.511129
C	2.085762	0.509126	-0.066756
C	2.652768	0.431677	1.214950
C	2.327758	-0.512031	-0.997858
C	3.431785	-0.664608	1.566625
H	2.437107	1.205585	1.943367
C	3.141030	-1.588103	-0.651476
C	3.686916	-1.670341	0.629215
H	3.333079	-2.370764	-1.377937
H	4.307288	-2.518638	0.901372
C	1.924235	3.063342	-0.253993
H	2.412446	3.115237	0.719008
H	2.690374	3.243158	-1.019829
H	1.143209	3.817367	-0.335542
H	1.874168	-0.454439	-1.977735
H	3.843657	-0.735439	2.567992
C	-2.471066	-2.053078	-1.031746
H	-2.188856	-2.688957	-1.875069
H	-3.021192	-2.665400	-0.311026
H	-3.139439	-1.279911	-1.413773
C	-0.278277	-2.585269	0.047114
H	-0.783278	-3.202904	0.797414
H	-0.009295	-3.225365	-0.797944
H	0.639613	-2.188633	0.484164
H	-1.500480	1.158198	-2.280726

C1-B5-TS1S

C	-2.681775	-0.891458	-0.510162
C	-2.300162	0.054316	0.684044
H	-1.989176	-0.558784	1.531018
O	-1.557532	-0.847046	-1.406901
B	-0.487219	-0.126859	-0.864341
N	-1.088148	0.814990	0.226231
C	-3.304741	1.129988	1.114191
H	-4.338678	0.807693	0.974483
H	-3.165073	1.338519	2.176139
C	-2.946536	2.385549	0.278881
H	-3.740468	2.648274	-0.425643
H	-2.785868	3.244181	0.932558
C	-1.653988	2.023403	-0.476437
H	-1.867482	1.764167	-1.516699
H	-0.901081	2.810243	-0.461026
B	-0.146613	1.330736	1.446101

H	0.845829	1.803909	0.933934
H	-0.769415	2.165364	2.069464
H	0.103406	0.377787	2.163548
O	0.355196	-1.247260	0.078440
C	1.450840	-1.154289	0.669667
C	2.599766	-0.409856	0.127112
C	3.522789	0.210071	0.983976
C	2.813358	-0.384812	-1.259854
C	4.624596	0.873093	0.456802
H	3.349180	0.216536	2.053529
C	3.932820	0.256607	-1.779662
H	2.110577	-0.882629	-1.915503
C	4.833587	0.892999	-0.924330
H	5.319295	1.377162	1.120412
H	4.099599	0.264195	-2.851717
H	5.699479	1.404598	-1.332951
C	1.627862	-1.951626	1.927843
H	1.815988	-1.268526	2.760500
H	0.719468	-2.516031	2.134176
H	2.489899	-2.619731	1.838671
C	-2.858719	-2.326782	-0.007229
H	-3.685906	-2.391053	0.708570
H	-3.072036	-2.998104	-0.843880
H	-1.943421	-2.666843	0.483217
C	-3.912442	-0.465983	-1.315587
H	-3.992095	-1.110875	-2.194638
H	-4.832794	-0.567697	-0.733210
H	-3.833188	0.565999	-1.663882
H	0.285319	0.374468	-1.621322

C1-B5-TS1Spri

C	2.771188	-1.038213	0.174792
C	2.337521	0.124166	-0.761910
H	1.856185	-0.323714	-1.632844
O	1.577176	-1.340845	0.895157
B	0.854119	-0.132692	1.190784
N	1.255237	0.873920	-0.013920
C	3.389992	1.168761	-1.188666
H	4.368498	0.927414	-0.767877
H	3.502691	1.190436	-2.274538
C	2.876892	2.522957	-0.647636
H	3.683774	3.165759	-0.284694
H	2.337929	3.061827	-1.429196
C	1.914006	2.132123	0.472027
H	2.450395	1.923123	1.400886
H	1.147418	2.878373	0.680398
B	0.059511	1.257892	-1.013143
H	-0.688859	2.031949	-0.464318
H	0.509855	1.641305	-2.068765
H	-0.572942	0.208394	-1.244933
O	-0.662541	-0.333069	1.130531
C	-1.349622	-0.817098	0.169316
C	-2.736775	-0.327281	0.063833
C	-3.683682	-0.971859	-0.746909
C	-3.117330	0.805163	0.802845
C	-4.988959	-0.494264	-0.814007
H	-3.407205	-1.846727	-1.323116
C	-4.420898	1.279791	0.731412
C	-5.358836	0.631653	-0.076513
H	-4.707957	2.157538	1.301190
H	-6.376750	1.005051	-0.132709
C	-0.938946	-2.088023	-0.521427
H	0.134603	-2.223047	-0.430848
H	-1.434585	-2.915199	0.004027
H	-1.255660	-2.101070	-1.563696
H	-2.373729	1.298361	1.415268
H	-5.716697	-0.998566	-1.441314
C	3.175147	-2.268495	-0.636425
H	2.369859	-2.565239	-1.314736
H	4.069932	-2.064745	-1.233756
H	3.390158	-3.107282	0.031346
C	3.883919	-0.693266	1.177024
H	4.855009	-0.565504	0.690362
H	3.658332	0.212843	1.743001
H	3.965513	-1.517205	1.890484
H	1.040641	0.377103	2.267578

C1-TS7R

C	1.418883	1.275882	-0.323119
C	1.129828	0.163737	0.751088
H	0.384433	0.538787	1.454590
O	0.723517	0.858351	-1.506972
B	-0.127214	-0.233562	-1.255632
N	0.495507	-0.977182	-0.003422
C	2.328420	-0.454758	1.485190
H	3.154969	0.251712	1.578187
H	2.026237	-0.740738	2.493491
C	2.707891	-1.715408	0.666024
H	3.723457	-1.658580	0.264832
H	2.647816	-2.606284	1.293662

C	1.677421	-1.786221	-0.472594
H	2.067737	-1.333313	-1.386909
H	1.340897	-2.796745	-0.698879
B	-0.434006	-1.904509	0.935382
H	-0.708373	-2.906202	0.309510
H	0.155547	-2.145331	1.963978
H	-1.463250	-1.281307	1.209959
O	-1.488459	0.371128	-0.644308
C	-2.511754	-0.289857	-0.350655
C	-2.912150	-1.533555	-1.084739
H	-2.066400	-2.199014	-1.233431
H	-3.707245	-2.057913	-0.554202
H	-3.286808	-1.219049	-2.067705
C	0.852283	2.618376	0.150206
C	2.891618	1.447165	-0.708613
C	-2.990204	1.549776	1.346797
H	-2.666242	2.341356	0.667764
H	-3.771652	1.949323	1.997987
H	-2.136365	1.264444	1.966776
H	-0.392244	-0.932058	-2.193157
C	-3.510059	0.341353	0.578112
H	-3.865025	-0.444740	1.252511
H	-4.378942	0.605588	-0.045355
H	1.005074	3.383506	-0.616070
H	-0.219284	2.529156	0.340162
H	1.345486	2.947800	1.071738
H	2.945150	2.120110	-1.568248
H	3.477147	1.885683	0.104687
H	3.351916	0.499659	-0.996617

C1-TS7Rpri

C	-1.427081	1.263379	0.253805
C	-1.319510	0.040799	-0.700036
H	-0.755543	0.359881	-1.577163
O	-0.180723	1.241906	0.953454
B	0.219005	-0.106406	1.199328
N	-0.454233	-0.970491	0.023542
C	-2.612015	-0.692589	-1.112125
H	-3.491806	-0.176199	-0.722254
H	-2.711658	-0.722894	-2.198841
C	-2.495342	-2.118426	-0.522991
H	-3.439572	-2.481036	-0.107227
H	-2.173312	-2.819143	-1.295021
C	-1.416916	-1.996530	0.552846
H	-1.837656	-1.643487	1.496900
H	-0.876380	-2.922817	0.746006
B	0.545571	-1.689480	-1.023222
H	1.048748	-2.647572	-0.475326
H	-0.071121	-1.976681	-2.023862
H	1.421754	-0.875642	-1.329869
O	1.764987	-0.308265	1.094503
C	2.682829	-0.262716	0.238977
C	3.683256	-1.383158	0.316399
H	4.594266	-1.176570	-0.246272
H	3.917376	-1.574806	1.366791
H	3.197596	-2.278129	-0.081907
C	3.062585	0.921646	-0.605319
H	3.572955	0.526754	-1.489280
H	3.862403	1.388374	-0.001889
C	2.019681	1.957566	-0.988724
H	1.477212	2.325867	-0.119831
H	2.509373	2.791357	-1.500141
H	1.293159	1.514776	-1.673378
C	-1.537369	2.566754	-0.536328
C	-2.570175	1.198555	1.280104
H	-0.018483	-0.563610	2.288016
H	-0.714097	2.667242	-1.246406
H	-2.479637	2.599541	-1.093070
H	-1.512166	3.421962	0.144830
H	-3.551980	1.318041	0.813178
H	-2.431643	2.013768	1.994888
H	-2.566813	0.263095	1.842505

C1-TS7S

C	1.725150	1.129067	-0.168833
C	1.478523	-0.086532	0.769704
H	1.086450	0.305415	1.709990
O	0.444335	1.348664	-0.757741
B	-0.170452	0.083134	-1.037435
N	0.367809	-0.899563	0.135155
C	2.620956	-1.068815	1.018496
H	3.596515	-0.575930	1.055341
H	2.460136	-1.562752	1.981176
C	2.498420	-2.092669	-0.120719
H	3.019824	-1.744155	-1.014179
H	2.923221	-3.064139	0.145798
C	0.986246	-2.189384	-0.365195
H	0.727173	-2.328488	-1.415148
H	0.553548	-3.009815	0.207520
B	-0.729486	-1.293635	1.246432

H	-1.471658	-2.129685	0.788510
H	-0.149282	-1.656762	2.246731
H	-1.376534	-0.270807	1.526167
O	-1.699680	0.148614	-0.885248
C	-2.358905	0.630696	0.081131
C	-2.023027	1.939579	0.733196
H	-0.951773	2.111322	0.749748
H	-2.477850	2.720112	0.106850
H	-2.466871	1.998104	1.727889
C	-3.745380	0.085424	0.298355
H	-3.844334	-0.114820	1.370422
H	-4.435436	0.917954	0.092640
C	-4.094815	-1.141762	-0.535748
H	-3.410971	-1.960725	-0.307646
H	-5.116706	-1.462954	-0.317410
H	-4.019912	-0.925129	-1.603929
C	2.754465	0.910775	-1.287918
C	2.107186	2.363128	0.647094
H	-0.001023	-0.394901	-2.131055
H	1.361456	2.560317	1.422840
H	2.170927	3.239997	-0.002718
H	3.078267	2.222751	1.133247
H	3.751494	0.691516	-0.894422
H	2.452282	0.103699	-1.956983
H	2.815937	1.824037	-1.885907

C1-TS7Spri

C	1.618991	1.250345	-0.172914
C	1.393675	-0.005734	0.748166
H	0.877358	0.310589	1.656256
O	0.646858	1.137915	-1.223506
B	-0.296875	0.131598	-0.947623
N	0.448583	-0.902762	-0.010621
C	2.609394	-0.885344	1.074868
H	3.536000	-0.308957	1.084255
H	2.483789	-1.320584	2.067109
C	2.610904	-2.003122	0.000270
H	3.517081	-1.988626	-0.611503
H	2.545885	-2.983784	0.474899
C	1.367856	-1.737878	-0.864437
H	1.624791	-1.167123	-1.759835
H	0.839819	-2.641632	-1.164618
B	-0.398227	-1.877917	0.957345
H	-0.952816	-2.699547	0.258929
H	0.349323	-2.382177	1.764199
H	-1.231808	-1.200951	1.563732
O	-1.376313	0.782984	0.056170
C	-2.409401	0.216845	0.484813
C	-3.065855	0.810463	1.692868
H	-4.030224	1.242903	1.399452
H	-3.269003	0.023058	2.422333
H	-2.435065	1.581027	2.134871
C	-3.163465	-0.812547	-0.308243
H	-2.467670	-1.528565	-0.739483
H	-3.833876	-1.355121	0.362605
C	-3.962833	-0.101168	-1.420609
H	-3.287504	0.406874	-2.112710
H	-4.539258	-0.840787	-1.981495
H	-4.664375	0.635457	-1.016543
C	2.992757	1.331299	-0.845008
C	1.357996	2.533806	0.622678
H	-0.865739	-0.341627	-1.891195
H	1.463120	3.407806	-0.026230
H	2.067634	2.630614	1.452135
H	0.343651	2.525633	1.028056
H	2.970091	2.143299	-1.576355
H	3.787040	1.544128	-0.123595
H	3.242718	0.410023	-1.375550

C2-B5-TS1R

C	1.779825	0.694100	-0.248415
C	1.461977	-0.387384	0.861527
H	0.680241	0.016358	1.501869
O	1.335761	0.096521	-1.478658
B	0.452178	-0.986421	-1.280724
N	0.833793	-1.570847	0.151751
C	2.586421	-0.986810	1.738209
H	3.510978	-0.422575	1.651213
H	2.288768	-0.956504	2.788375
C	2.768594	-2.450744	1.268351
H	3.818253	-2.713747	1.109968
H	2.355275	-3.139749	2.007282
C	1.972286	-2.536413	-0.031837
H	2.573593	-2.211987	-0.882054
H	1.567465	-3.525132	-0.242232
B	-0.294063	-2.259743	1.069287
H	-0.485992	-3.388160	0.678133
H	0.028595	-2.198380	2.233413
H	-1.341497	-1.602239	0.952701
O	-0.993484	-0.384851	-1.104653

C	-2.036266	-1.027166	-0.775970
C	-2.291841	-2.416468	-1.285832
H	-2.886732	-2.309869	-2.202641
H	-1.361419	-2.923880	-1.518984
H	-2.856194	-3.015342	-0.574102
C	0.848243	1.925398	0.029145
C	3.256583	1.104389	-0.536289
C	4.146095	-0.052769	-1.017250
H	4.414244	-0.752686	-0.221879
H	3.651799	-0.602584	-1.819030
H	5.082612	0.353030	-1.414133
C	3.967130	1.937850	0.546293
H	5.043505	1.957206	0.347549
H	3.624870	2.973899	0.550816
H	3.830464	1.546891	1.557315
C	0.860859	2.945923	-1.112364
H	0.040480	3.662430	-0.991239
H	1.790905	3.524486	-1.132717
H	0.744399	2.443175	-2.074733
C	1.009409	2.625095	1.390353
H	1.174195	1.923707	2.213797
H	1.831957	3.341984	1.398608
H	0.095640	3.183138	1.621041
H	0.438018	-1.810935	-2.155244
H	-0.147364	1.473610	0.038049
H	3.154646	1.754958	-1.411999
C	-3.152267	-0.221836	-0.233819
C	-4.309032	-0.823296	0.285152
C	-3.054310	1.179514	-0.252582
C	-5.343229	-0.037712	0.783917
H	-4.393096	-1.902650	0.323949
C	-4.094263	1.960721	0.238175
H	-2.166278	1.637343	-0.669963
C	-5.238914	1.354384	0.760329
H	-6.229217	-0.510765	1.194507
H	-4.013974	3.042633	0.213049
H	-6.048275	1.965082	1.148177

C2-B5-TS1Rpri

C	-1.181352	-0.816908	-0.148929
C	-1.541373	0.353223	0.829223
H	-0.888146	0.256629	1.693617
O	-0.314171	-0.205153	-1.122150
B	-0.414101	1.207926	-1.181647
N	-1.113571	1.653863	0.167962
C	-2.993061	0.565779	1.311974
H	-3.696930	-0.026433	0.733462
H	-3.103815	0.264952	2.356960
C	-3.281336	2.065895	1.105716
H	-4.334036	2.260989	0.880786
H	-3.007961	2.637772	1.993477
C	-2.371324	2.440956	-0.060746
H	-2.815146	2.146465	-1.014389
H	-2.119797	3.500616	-0.106866
B	-0.176435	2.466027	1.227791
H	0.094690	3.546158	0.745103
H	-0.786475	2.558421	2.269802
H	0.826095	1.785681	1.406266
O	1.022936	1.923114	-1.231495
C	2.084040	1.797764	-0.573417
C	2.837216	3.064025	-0.293706
H	2.326201	3.568773	0.532258
H	3.883795	2.889779	-0.047836
H	2.751957	3.714464	-1.167253
C	-0.290142	-1.847935	0.617384
C	-2.319726	-1.493989	-0.979400
C	-0.847936	-2.374561	1.950194
H	-0.056559	-2.912442	2.482886
H	-1.190877	-1.572768	2.609567
H	-1.677057	-3.070989	1.815746
C	0.169233	-3.013423	-0.266106
H	1.001501	-3.540532	0.210969
H	-0.629255	-3.743876	-0.429398
H	0.509821	-2.654972	-1.238966
C	-3.256280	-2.457011	-0.226555
H	-2.744077	-3.369427	0.082894
H	-3.707062	-2.016756	0.665542
H	-4.074416	-2.757947	-0.889312
C	-3.130547	-0.522803	-1.853526
H	-3.837935	0.083358	-1.281605
H	-2.476297	0.147404	-2.411891
H	-3.718714	-1.096136	-2.577914
H	-0.914164	1.697442	-2.161500
H	0.601245	-1.258714	0.863104
H	-1.756382	-2.106864	-1.691503
C	2.677480	0.499312	-0.225336
C	3.448527	0.362095	0.941688
C	2.561225	-0.577076	-1.116588
C	4.064240	-0.849238	1.228932
H	3.520025	1.190129	1.638121

C	3.224163	-1.771253	-0.846999
H	1.958776	-0.465945	-2.007656
C	3.963141	-1.913375	0.326990
H	4.630301	-0.964379	2.147333
H	3.147843	-2.597430	-1.545276
H	4.463280	-2.852763	0.541416

C2-B5-TS1S

C	-2.112569	0.320817	0.317722
C	-1.568905	-0.521346	-0.906259
H	-1.374510	0.171966	-1.722022
O	-1.061744	0.243506	1.304396
B	0.147392	-0.228659	0.781420
N	-0.211681	-1.050282	-0.500389
C	-2.345764	-1.744220	-1.443638
H	-3.366941	-1.774216	-1.072243
H	-2.401918	-1.687111	-2.532583
C	-1.543785	-2.997275	-1.013458
H	-2.170453	-3.741632	-0.513941
H	-1.087638	-3.470813	-1.883294
C	-0.462836	-2.470458	-0.068537
H	-0.814424	-2.474517	0.964699
H	0.477701	-3.017586	-0.121209
B	0.821185	-1.083907	-1.758282
H	1.857688	-1.557368	-1.346935
H	0.325988	-1.765671	-2.630033
H	0.958218	0.055342	-2.171055
O	0.924018	1.128685	0.186943
C	2.033063	1.275193	-0.369938
C	2.158844	2.387835	-1.368473
H	1.175179	2.800773	-1.586426
H	2.817085	3.165234	-0.964223
H	2.612270	2.020038	-2.291042
C	-3.385495	-0.150962	1.083913
C	-2.192037	1.820060	-0.134109
C	-2.496843	2.772110	1.026942
H	-1.854864	2.549761	1.881991
H	-2.320520	3.809294	0.722028
H	-3.541528	2.704170	1.349373
C	-4.729966	0.006029	0.350465
H	-5.054110	1.047407	0.316038
H	-4.710684	-0.362476	-0.677862
H	-5.505741	-0.553820	0.882714
C	-3.257958	-1.547118	1.712299
H	-3.307397	-2.356487	0.980305
H	-2.322892	-1.630861	2.267605
H	-4.082057	-1.702351	2.416597
C	-3.094159	2.128117	-1.342229
H	-3.010777	1.381904	-2.137898
H	-4.148164	2.203490	-1.070385
H	-2.805493	3.092769	-1.773096
H	0.894824	-0.765947	1.541907
H	-1.166256	2.032331	-0.451528
H	-3.416512	0.534665	1.937837
C	3.241418	0.535343	0.032402
C	4.252687	0.242632	-0.896287
C	3.418028	0.191581	1.381752
C	5.408413	-0.409943	-0.484866
H	4.109238	0.475716	-1.944546
C	4.588378	-0.438497	1.792216
H	2.645102	0.436849	2.098406
C	5.580173	-0.746808	0.860093
H	6.174138	-0.660363	-1.211520
H	4.724637	-0.692045	2.838337
H	6.487381	-1.249718	1.180274

C2-B5-TS1Spri

C	2.125330	0.499966	-0.121129
C	1.570774	-0.492744	0.967121
H	1.160506	0.139108	1.755672
O	1.007424	0.665837	-0.999212
B	0.145618	-0.479740	-1.049465
N	0.382074	-1.219777	0.364283
C	2.414107	-1.600215	1.601067
H	3.454977	-1.323293	1.756186
H	1.980098	-1.825484	2.580565
C	2.220318	-2.806583	0.677410
H	2.891814	-2.750580	-0.181352
H	2.411616	-3.757429	1.182101
C	0.757991	-2.683949	0.237138
H	0.583989	-3.010606	-0.788133
H	0.105540	-3.256262	0.897317
B	-0.870396	-1.136454	1.361771
H	-1.732677	-1.896696	0.995997
H	-0.487510	-1.307170	2.498078
H	-1.336832	0.025734	1.284015
O	-1.339110	-0.094839	-1.118818
C	-1.986786	0.677623	-0.330856
C	-1.476341	2.056684	-0.013012
H	-0.396333	2.075226	-0.111208

H	-1.905183	2.738510	-0.759035
H	-1.786293	2.389270	0.977106
C	3.377274	0.133963	-1.001037
C	2.305012	1.896628	0.568542
C	4.636252	-0.402659	-0.293337
H	5.498985	-0.262825	-0.953247
H	4.862332	0.101372	0.645825
H	4.568192	-1.474060	-0.092180
C	3.373147	1.967227	1.667541
H	4.383443	1.993195	1.250914
H	3.243368	2.886951	2.247434
H	3.313606	1.128131	2.367414
C	2.486446	3.047058	-0.429351
H	3.500199	3.077256	-0.841946
H	1.781063	2.958151	-1.257943
H	2.317182	4.005898	0.072232
C	3.064225	-0.755458	-2.213911
H	3.972054	-0.873621	-2.815642
H	2.728332	-1.756067	-1.931369
H	2.290081	-0.311224	-2.837827
H	0.274672	-1.234513	-1.977963
H	1.339474	2.060623	1.062976
H	3.664662	1.097907	-1.433656
C	-3.419710	0.362830	-0.158576
C	-4.299645	1.260504	0.464670
C	-3.911659	-0.859957	-0.643264
C	-5.647626	0.941594	0.598597
H	-3.937451	2.207738	0.845996
C	-5.258289	-1.174401	-0.508573
H	-3.220235	-1.545833	-1.115183
C	-6.128628	-0.275375	0.112914
H	-6.322189	1.640904	1.081772
H	-5.631617	-2.121737	-0.883785
H	-7.180160	-0.523561	0.219863

C2-TS7R

C	1.270135	-0.504499	0.187510
C	1.055273	0.744877	-0.729155
H	0.971831	0.437363	-1.771328
O	0.512562	-0.189455	1.362179
B	-0.615832	0.596949	1.077880
N	-0.316860	1.319920	-0.325461
C	2.021711	1.936300	-0.641826
H	2.453693	2.017591	0.358360
H	2.838493	1.843016	-1.358228
C	1.128561	3.148608	-0.914610
H	1.579527	4.095614	-0.605235
H	0.876236	3.213404	-1.977337
C	-0.105916	2.799004	-0.100103
H	0.081854	2.978575	0.961964
H	-1.009646	3.333349	-0.395550
B	-1.378109	1.127594	-1.527634
H	-1.224088	2.014825	-2.336026
H	-1.268130	0.027340	-2.007931
H	-2.518171	1.237937	-1.044105
O	-1.857016	-0.332535	0.759382
C	-3.002232	0.117250	0.487092
C	-3.565239	1.357560	1.129923
H	-3.885290	1.075453	2.142351
H	-2.828964	2.150328	1.220570
H	-4.435273	1.712438	0.576777
C	0.616629	-1.758794	-0.490791
C	2.734412	-0.824695	0.639171
C	3.127943	-0.180358	1.976003
H	4.127468	-0.524543	2.264571
H	3.162424	0.911301	1.915435
H	2.422534	-0.444781	2.763092
C	3.833004	-0.567496	-0.411493
H	3.510008	-0.773846	-1.433260
H	4.191326	0.463529	-0.375646
H	4.694877	-1.210087	-0.204548
C	0.339904	-2.884541	0.513292
H	-0.197660	-2.506606	1.383436
H	-0.266154	-3.663201	0.037610
H	1.260998	-3.363883	0.863109
C	1.377816	-2.306899	-1.704189
H	0.781057	-3.087256	-2.187790
H	1.575918	-1.539892	-2.459312
H	2.333048	-2.759450	-1.420091
C	-3.344962	-2.067654	-0.788757
H	-2.788879	-2.637022	-0.041144
H	-4.121777	-2.710766	-1.210506
H	-2.652192	-1.786952	-1.584229
H	-0.941742	1.344105	1.961169
C	-3.973898	-0.821575	-0.175091
H	-4.536686	-0.245792	-0.916450
H	-4.704504	-1.089170	0.605129
H	-0.353536	-1.417551	-0.856157
H	2.735905	-1.904129	0.823955

C2-TS7Rpri

C	-1.181219	-0.472445	-0.169421
C	-0.723070	0.664957	0.807233
H	-0.355440	0.176043	1.707548
O	-0.036846	-0.664075	-1.022843
B	0.796878	0.482433	-1.115399
N	0.502913	1.335607	0.197576
C	-1.688056	1.803677	1.203074
H	-2.569615	1.812984	0.566399
H	-2.035393	1.682896	2.232220
C	-0.888451	3.106225	1.007831
H	-1.526180	3.953740	0.740290
H	-0.338713	3.362655	1.915532
C	0.085563	2.747113	-0.109479
H	-0.407404	2.774055	-1.082755
H	0.972300	3.378296	-0.150125
B	1.691886	1.384458	1.302882
H	2.522769	2.179589	0.930740
H	1.222454	1.668261	2.381335
H	2.175137	0.249028	1.387722
O	2.336664	0.112368	-1.102447
C	3.263124	-0.254088	-0.344217
C	4.474459	0.629445	-0.237542
H	5.307589	0.147309	-0.764927
H	4.270591	1.605189	-0.672482
H	4.763127	0.742887	0.808911
C	3.430244	-1.669313	0.142604
H	4.104250	-1.655327	1.004375
H	4.006365	-2.137271	-0.674024
C	2.173371	-2.477163	0.421684
H	2.427950	-3.530408	0.567419
H	1.695516	-2.108897	1.331375
H	1.456757	-2.385027	-0.394417
C	-1.320140	-1.790362	0.657847
C	-2.395455	-0.234114	-1.127674
C	-2.260012	-1.742988	1.874728
H	-2.054140	-2.604273	2.518977
H	-2.113815	-0.845150	2.482228
H	-3.314938	-1.790822	1.603220
C	-1.602855	-3.019292	-0.212652
H	-1.445944	-3.935309	0.366709
H	-2.635317	-3.039275	-0.575858
H	-0.935948	-3.042131	-1.077594
C	-3.802933	-0.307893	-0.506009
H	-4.069961	-1.323739	-0.211737
H	-3.923950	0.331179	0.371825
H	-4.541719	0.015006	-1.246795
C	-2.284040	1.008752	-2.025715
H	-2.465831	1.942663	-1.486622
H	-1.308317	1.070905	-2.506726
H	-3.041129	0.947985	-2.814621
H	0.742363	1.136845	-2.123514
H	-0.315090	-1.919572	1.065955
H	-2.314252	-1.085431	-1.812462

C2-TS7S

C	1.288684	-0.356951	0.123595
C	0.668781	0.743342	-0.835238
H	0.239740	0.244398	-1.704268
O	0.338017	-0.463109	1.200107
B	-0.864402	0.217012	0.939594
N	-0.490248	1.352947	-0.082035
C	1.525226	1.947332	-1.266437
H	2.588174	1.714920	-1.286850
H	1.236234	2.242289	-2.276137
C	1.189206	3.083946	-0.266658
H	2.068600	3.420185	0.289757
H	0.779754	3.945750	-0.796190
C	0.140685	2.486337	0.682372
H	0.609510	2.082610	1.583244
H	-0.639718	3.186728	0.976149
B	-1.622529	1.958905	-1.068808
H	-2.388254	2.605201	-0.386539
H	-1.084135	2.632935	-1.917030
H	-2.207614	1.027392	-1.618011
O	-1.771751	-0.791410	0.063898
C	-2.943881	-0.598287	-0.329116
C	-3.426490	-1.500806	-1.429243
H	-4.482212	-1.349017	-1.654422
H	-2.831984	-1.306267	-2.324752
H	-3.252884	-2.540977	-1.136496
C	-3.926199	0.271681	0.392698
H	-3.413692	1.088218	0.893169
H	-4.618598	0.698098	-0.336659
C	-4.685932	-0.605022	1.413500
H	-5.208460	-1.438295	0.933628
H	-3.998177	-1.011283	2.159645
H	-5.430103	0.006048	1.929736
C	2.644801	0.068736	0.784791
C	1.260585	-1.726090	-0.629813

C	1.629625	-2.922610	0.253466
H	1.098800	-2.874765	1.206126
H	1.355088	-3.856862	-0.248909
H	2.704130	-2.968901	0.454525
C	2.846360	-0.510368	2.195263
H	3.000839	-1.591588	2.176528
H	3.736627	-0.058209	2.646148
H	1.986758	-0.304655	2.831813
C	3.913276	-0.188469	-0.043753
H	3.835917	0.145447	-1.080255
H	4.756625	0.340660	0.412023
H	4.170282	-1.251133	-0.056895
C	2.015609	-1.786743	-1.969212
H	1.865359	-0.892635	-2.582102
H	3.090036	-1.922435	-1.837711
H	1.652975	-2.641557	-2.550071
H	-1.497014	0.564053	1.898312
H	0.197516	-1.839658	-0.869841
H	2.574138	1.153080	0.923310

C2-TS7Spri

C	1.340306	0.535861	-0.150675
C	0.777894	-0.430927	0.944630
H	0.305050	0.203443	1.687980
O	0.178931	0.755253	-0.962103
B	-0.676126	-0.395051	-1.025083
N	-0.362538	-1.211207	0.324604
C	1.636277	-1.482231	1.645685
H	2.667647	-1.164396	1.796230
H	1.198748	-1.667227	2.629888
C	1.503452	-2.747859	0.783744
H	2.299313	-2.797576	0.037778
H	1.571271	-3.656754	1.386734
C	0.130482	-2.625204	0.097139
H	0.181144	-2.818625	-0.974425
H	-0.600913	-3.306860	0.529007
B	-1.585109	-1.293178	1.374797
H	-2.410986	-2.068502	0.957895
H	-1.148055	-1.589969	2.464433
H	-2.088207	-0.158851	1.461577
O	-2.180495	-0.013037	-0.985269
C	-2.786205	0.703946	-0.140249
C	-2.237588	2.008850	0.353295
H	-1.156081	1.979791	0.407648
H	-2.514640	2.765364	-0.394074
H	-2.687421	2.287329	1.307045
C	-4.260356	0.456679	0.040813
H	-4.461117	0.474387	1.117114
H	-4.766319	1.346655	-0.364663
C	-4.784608	-0.818496	-0.609437
H	-4.285753	-1.691455	-0.185774
H	-5.860979	-0.908860	-0.440906
H	-4.600376	-0.813484	-1.686130
C	2.525980	0.088828	-1.086370
C	1.795598	1.924320	0.452803
C	3.823264	-0.348274	-0.381029
H	4.661365	-0.260280	-1.079880
H	4.068797	0.259941	0.493908
H	3.787202	-1.391639	-0.061629
C	1.472434	3.077856	-0.510980
H	0.399156	3.275998	-0.546898
H	1.977781	3.990729	-0.179400
H	1.791889	2.865242	-1.533514
C	1.307076	2.280513	1.865192
H	0.217111	2.342543	1.937907
H	1.652404	1.567159	2.618960
H	1.702390	3.263130	2.142831
C	2.185501	-0.904686	-2.203567
H	3.059274	-1.022006	-2.853792
H	1.930681	-1.898103	-1.828566
H	1.354176	-0.548437	-2.809845
H	-0.620695	-1.089702	-2.003499
H	2.886265	1.876970	0.529342
H	2.769294	1.020858	-1.611067

C3-B5-TS1R

C	-1.291687	0.046161	-0.140796
C	-0.560939	0.860899	1.007044
H	0.294296	0.290678	1.363392
O	-0.805315	0.653887	-1.346719
B	0.210420	1.611448	-1.133062
N	0.000398	2.102772	0.351429
C	-1.382937	1.411477	2.186010
H	-2.231776	0.782301	2.439223
H	-0.740655	1.460606	3.066179
C	-1.811383	2.841914	1.766268
H	-2.897684	2.941801	1.689680
H	-1.461231	3.572816	2.497575
C	-1.142856	3.080314	0.402924
H	-1.825112	2.865333	-0.422084

H	-0.748736	4.087039	0.276541
B	1.243765	2.746412	1.145638
H	1.437034	3.854593	0.699754
H	1.027878	2.738557	2.334978
H	2.246278	2.048774	0.951906
O	1.592087	0.835192	-1.040120
C	2.727161	1.354987	-0.826418
C	3.773358	0.455192	-0.295058
C	3.395509	-0.692857	0.419880
C	5.134870	0.713926	-0.508892
C	4.358497	-1.570537	0.899125
H	2.345268	-0.870591	0.607374
C	6.098732	-0.173258	-0.035319
C	5.713359	-1.314960	0.667667
H	7.149909	0.027715	-0.214212
H	6.466770	-2.001632	1.040789
C	3.103881	2.689698	-1.401134
H	3.551890	2.507594	-2.387064
H	2.227912	3.317723	-1.525098
H	3.830567	3.198858	-0.769897
H	5.445246	1.595951	-1.056734
H	4.056029	-2.450626	1.457380
C	-0.946947	-1.490387	-0.147250
C	0.125308	-1.911903	-1.165796
C	-0.625581	-2.085182	1.236954
H	-1.856927	-1.999416	-0.485645
C	0.248619	-3.439852	-1.228664
H	1.093025	-1.478150	-0.907026
H	-0.131743	-1.506251	-2.146144
C	-0.515392	-3.613979	1.173938
H	0.332005	-1.691536	1.602411
H	-1.379028	-1.791811	1.975196
C	0.539184	-4.047175	0.148847
H	1.032188	-3.722570	-1.941548
H	-0.690181	-3.863488	-1.613386
H	-0.275754	-4.015875	2.165455
H	-1.489954	-4.035850	0.891298
H	0.586690	-5.140715	0.086468
H	1.529519	-3.709957	0.489274
C	-2.854114	0.216328	-0.155264
C	-3.431896	0.027057	-1.572908
C	-3.656840	-0.676810	0.817221
H	-3.069050	1.253421	0.123910
C	-4.926492	0.368692	-1.619719
H	-3.287082	-1.015112	-1.890588
H	-2.876335	0.647135	-2.276690
C	-5.147085	-0.308365	0.801024
H	-3.561433	-1.727940	0.519788
H	-3.279225	-0.618762	1.840575
C	-5.735422	-0.448166	-0.606704
H	-5.314417	0.211535	-2.633011
H	-5.053977	1.438586	-1.400613
H	-5.695267	-0.940345	1.509525
H	-5.268618	0.728034	1.146395
H	-6.788102	-0.141925	-0.615724
H	-5.713296	-1.507752	-0.898020
H	0.281757	2.480624	-1.957651

C3-B5-TS1Rpri

C	0.959043	-0.043838	0.239749
C	0.914223	-1.037923	1.463406
H	0.448239	-0.526276	2.299972
O	-0.138498	-0.478008	-0.571357
B	-0.285763	-1.887801	-0.460671
N	-0.038372	-2.167703	1.084023
C	2.203797	-1.740447	1.929608
H	3.109285	-1.184111	1.697485
H	2.164629	-1.859889	3.017507
C	2.160529	-3.124606	1.266598
H	2.493453	-3.070982	0.225713
H	2.780576	-3.863922	1.781250
C	0.676811	-3.461138	1.333811
H	0.346129	-4.200827	0.603152
H	0.405602	-3.813225	2.332095
B	-1.380742	-2.129814	1.992499
H	-1.945234	-3.195745	1.865177
H	-1.031416	-1.927616	3.138051
H	-2.087346	-1.206183	1.596709
O	-1.741354	-2.357096	-0.752046
C	-2.834574	-1.773172	-0.517303
C	-3.079177	-0.371404	-0.879010
C	-4.009492	0.409154	-0.174112
C	-2.433787	0.163413	-2.005576
C	-4.263836	1.716336	-0.571328
H	-4.484854	0.011518	0.714959
C	-2.715386	1.461706	-2.416040
C	-3.621456	2.241910	-1.695671
H	-2.216426	1.869928	-3.288525
H	-3.825229	3.261673	-2.006828
C	-3.985073	-2.635861	-0.104813

H	-4.685770	-2.719013	-0.944095
H	-3.622166	-3.616205	0.194520
H	-4.523013	-2.176732	0.726567
H	-1.721889	-0.443950	-2.548188
H	-4.957087	2.328220	-0.003970
C	2.247644	-0.143328	-0.658456
C	1.951139	0.278529	-2.114093
C	3.500834	0.623444	-0.187871
H	2.518290	-1.205422	-0.701721
C	3.145340	0.014938	-3.039236
H	1.701117	1.348595	-2.137909
H	1.072947	-0.255493	-2.476135
C	4.708204	0.334449	-1.091011
H	3.296494	1.701031	-0.212818
H	3.764850	0.384314	0.844826
C	4.418463	0.709683	-2.547008
H	2.905095	0.336261	-4.059891
H	3.323150	-1.068721	-3.088335
H	5.586631	0.877126	-0.722453
H	4.952900	-0.735551	-1.032774
H	5.270964	0.455286	-3.187811
H	4.286296	1.798712	-2.616285
C	0.677406	1.425078	0.691039
C	-0.819473	1.701081	0.907188
C	1.449795	1.881132	1.948822
H	0.999432	2.071661	-0.137723
C	-1.088477	3.176458	1.223891
H	-1.196534	1.070329	1.722137
H	-1.367060	1.412466	0.015700
C	1.210602	3.369523	2.238657
H	1.104581	1.307070	2.817372
H	2.520414	1.694169	1.861488
C	-0.280697	3.665331	2.430266
H	-2.161556	3.327625	1.392535
H	-0.824708	3.783863	0.345722
H	1.777888	3.671509	3.126970
H	1.596972	3.968453	1.401666
H	-0.442292	4.737023	2.597413
H	-0.633294	3.148181	3.333589
H	0.385468	-2.595009	-1.176243

C3-B5-TS1S

C	1.477649	0.022724	0.214465
C	0.689250	-0.509279	1.485754
H	0.343061	0.340437	2.056373
O	0.642104	-0.341800	-0.895047
B	-0.656004	-0.710354	-0.515904
N	-0.572831	-1.170638	0.979778
C	1.334401	-1.520267	2.459269
H	2.409722	-1.608554	2.304385
H	1.188884	-1.175096	3.484852
C	0.593438	-2.865156	2.242295
H	1.280116	-3.704448	2.097884
H	-0.032527	-3.091237	3.106217
C	-0.281994	-2.647151	1.003992
H	0.244851	-2.924238	0.089287
H	-1.229217	-3.183906	1.036362
B	-1.821862	-0.903022	1.988965
H	-2.753684	-1.559781	1.583057
H	-1.477064	-1.233285	3.103369
H	-2.072116	0.293756	1.994253
O	-1.502996	0.715061	-0.455198
C	-2.692231	0.959037	-0.151773
C	-3.815851	0.073086	-0.504235
C	-4.957969	-0.002180	0.308093
C	-3.776750	-0.635384	-1.714654
C	-6.031949	-0.797001	-0.072700
H	-4.978063	0.512724	1.261192
C	-4.866003	-1.409171	-2.103168
H	-2.901967	-0.560361	-2.347347
C	-5.989944	-1.497260	-1.281009
H	-6.900109	-0.876070	0.573168
H	-4.835448	-1.946118	-3.045519
H	-6.833389	-2.111837	-1.579941
C	-2.996473	2.319799	0.402363
H	-3.634959	2.240798	1.284032
H	-2.073000	2.834950	0.660414
H	-3.548229	2.893715	-0.351181
C	2.873180	-0.635169	-0.044987
C	2.810666	-2.150497	-0.318515
C	3.646676	0.004013	-1.222633
H	3.470264	-0.466176	0.864368
C	4.200496	-2.778818	-0.487055
H	2.221148	-2.301557	-1.229697
H	2.294866	-2.679605	0.478196
C	5.046012	-0.600645	-1.397813
H	3.063943	-0.149321	-2.139129
H	3.755751	1.082319	-1.094061
C	4.990549	-2.113867	-1.615374
H	4.098360	-3.854981	-0.670912

H	4.758583	-2.675626	0.454753
H	5.556228	-0.110133	-2.235296
H	5.643939	-0.386571	-0.500264
H	6.001074	-2.533888	-1.682865
H	4.495530	-2.323151	-2.573787
C	1.676519	1.600174	0.274358
C	1.310809	2.270049	-1.066306
C	0.999128	2.375162	1.423018
H	2.749604	1.761804	0.439479
C	1.706933	3.749971	-1.095776
H	0.230984	2.180540	-1.212814
H	1.769114	1.738434	-1.900610
C	1.369547	3.866039	1.394089
H	-0.090097	2.267210	1.363409
H	1.302138	1.971793	2.395368
C	1.042973	4.520098	0.049011
H	1.434767	4.190684	-2.062316
H	2.799195	3.842688	-1.007280
H	0.858823	4.388153	2.211978
H	2.447141	3.967075	1.585842
H	1.356619	5.570709	0.048523
H	-0.046431	4.517544	-0.101814
H	-1.236480	-1.453992	-1.247973

C3-B5-TS1Spri

C	1.361932	0.073109	-0.019987
C	0.628351	-0.055833	1.353086
H	0.224638	0.933890	1.573157
O	0.234237	0.072381	-0.910796
B	-0.672380	-0.970231	-0.509943
N	-0.566976	-0.957328	1.105597
C	1.297373	-0.648709	2.594287
H	2.342687	-0.363737	2.703674
H	0.760567	-0.275005	3.470779
C	1.086236	-2.164035	2.473383
H	1.900793	-2.628726	1.913280
H	1.048915	-2.650449	3.451604
C	-0.248460	-2.305716	1.720759
H	-0.207551	-3.057673	0.931635
H	-1.062377	-2.564765	2.396934
B	-1.865778	-0.374463	1.849691
H	-2.738123	-1.210031	1.841419
H	-1.553443	0.012381	2.954460
H	-2.271938	0.608315	1.198158
O	-2.124865	-0.681796	-0.875964
C	-2.816519	0.365342	-0.637480
C	-4.263055	0.149941	-0.458370
C	-5.171110	1.220203	-0.463183
C	-4.742195	-1.160433	-0.292145
C	-6.533457	0.983565	-0.308113
H	-4.819368	2.236934	-0.590401
C	-6.102819	-1.392197	-0.136414
C	-7.000674	-0.321489	-0.143752
H	-6.466407	-2.406047	-0.004630
H	-8.063667	-0.504084	-0.019453
C	-2.279434	1.730446	-0.949212
H	-2.551010	1.957167	-1.989090
H	-2.709315	2.494930	-0.303415
H	-1.198734	1.718639	-0.869952
H	-4.029932	-1.975046	-0.280361
H	-7.230020	1.815517	-0.314129
C	2.271436	-1.136725	-0.426916
C	2.453108	-1.210477	-1.957240
C	3.656317	-1.224639	0.241609
H	1.723530	-2.044980	-0.151885
C	3.181297	-2.493542	-2.374429
H	3.031919	-0.342604	-2.302517
H	1.478188	-1.150838	-2.441662
C	4.386169	-2.515481	-0.158557
H	4.268057	-0.368008	-0.067784
H	3.582405	-1.179745	1.330181
C	4.539890	-2.627314	-1.678635
H	3.306501	-2.515938	-3.463385
H	2.555599	-3.359021	-2.114818
H	5.366665	-2.555443	0.330337
H	3.816585	-3.379702	0.212176
H	5.020657	-3.575536	-1.946440
H	5.205638	-1.826478	-2.030425
C	2.126447	1.427421	-0.162077
C	1.236961	2.586723	-0.641243
C	2.883679	1.866272	1.109227
H	2.880952	1.281955	-0.946032
C	2.049630	3.865028	-0.883850
H	0.462635	2.791304	0.111080
H	0.724596	2.290794	-1.558148
C	3.713632	3.132792	0.860648
H	2.163373	2.072995	1.911001
H	3.537620	1.072470	1.474470
C	2.837819	4.285604	0.360626
H	1.384394	4.674142	-1.207845

H	2.751752	3.689006	-1.710987
H	4.238742	3.420060	1.778986
H	4.488199	2.914396	0.112008
H	3.449389	5.170333	0.148692
H	2.133859	4.571836	1.154743
H	-0.477657	-2.072268	-0.961389

C3-TS4R

C	1.339123	-0.008157	-0.122331
C	0.761547	-0.904008	1.046385
H	-0.143031	-0.437255	1.433015
O	0.834527	-0.643279	-1.309169
B	-0.012352	-1.743166	-1.053984
N	0.326172	-2.203200	0.409213
C	1.691349	-1.348090	2.185823
H	2.451050	-0.607324	2.421872
H	1.093022	-1.499996	3.084975
C	2.297783	-2.698504	1.723401
H	3.378168	-2.631956	1.568026
H	2.117874	-3.472190	2.471837
C	1.577581	-3.039889	0.406699
H	2.181382	-2.771462	-0.463024
H	1.294223	-4.087536	0.322223
B	-0.793683	-3.005313	1.257958
H	-0.922367	-4.092274	0.739206
H	-0.444096	-3.082046	2.413181
H	-1.853715	-2.386212	1.212219
O	-1.493280	-1.152660	-0.903302
C	-2.563490	-1.774533	-0.701002
C	-2.814092	-3.163992	-1.193655
H	-3.481521	-3.073077	-2.062472
H	-1.898947	-3.671162	-1.482837
H	-3.336152	-3.741913	-0.430454
C	0.830558	1.477242	-0.059868
C	-0.462246	1.739214	-0.847565
C	0.681861	2.045027	1.366770
H	1.599454	2.081939	-0.557287
C	-0.809786	3.232125	-0.877721
H	-1.285786	1.186179	-0.390942
H	-0.356279	1.345044	-1.859985
C	0.357806	3.544573	1.338886
H	-0.136683	1.529995	1.885394
H	1.581957	1.869705	1.961200
C	-0.917723	3.819246	0.534306
H	-1.747331	3.386356	-1.426506
H	-0.032449	3.775124	-1.433671
H	0.255161	3.925562	2.361694
H	1.197657	4.088332	0.884090
H	-1.121454	4.895569	0.491141
H	-1.771974	3.358341	1.051862
C	2.906789	-0.018441	-0.258591
C	3.345793	0.247789	-1.714325
C	3.696626	0.934735	0.664919
H	3.244326	-1.032651	-0.021183
C	4.857875	0.063880	-1.892817
H	3.070773	1.273687	-1.997110
H	2.800191	-0.416135	-2.384594
C	5.210597	0.730050	0.511558
H	3.460486	1.975173	0.410854
H	3.426426	0.807969	1.715307
C	5.660604	0.950416	-0.935684
H	5.138547	0.271502	-2.932133
H	5.113905	-0.989022	-1.706401
H	5.746996	1.406438	1.187347
H	5.469424	-0.292262	0.821798
H	6.735077	0.758085	-1.038254
H	5.502429	2.004954	-1.202226
H	-0.016876	-2.609275	-1.884822
C	-3.740387	-0.953990	-0.233934
C	-3.463108	-0.165831	1.062568
C	-4.124564	0.008669	-1.392713
H	-4.581200	-1.636158	-0.059414
C	-4.637021	0.748775	1.426265
H	-2.559615	0.435003	0.921683
H	-3.250409	-0.869109	1.871137
C	-5.298878	0.911573	-1.002276
H	-3.248957	0.621727	-1.631309
H	-4.368148	-0.567232	-2.293250
C	-4.994728	1.698142	0.277328
H	-4.387623	1.316487	2.329397
H	-5.514404	0.135817	1.675432
H	-5.521788	1.593186	-1.830690
H	-6.197347	0.298331	-0.848436
H	-5.851133	2.324054	0.551016
H	-4.151474	2.377876	0.091956

C3-TS4S

C	1.628295	0.007321	0.207958
C	0.864611	-0.571345	1.475471
H	0.536447	0.256408	2.087486

O	0.782027	-0.330689	-0.897863
B	-0.533972	-0.648037	-0.505439
N	-0.412979	-1.214531	0.972733
C	1.524241	-1.624603	2.394009
H	2.596087	-1.708493	2.216192
H	1.397919	-1.324341	3.436107
C	0.774561	-2.956360	2.130016
H	1.453290	-3.796882	1.958871
H	0.142749	-3.205735	2.984257
C	-0.095178	-2.682027	0.900536
H	0.446737	-2.879667	-0.025245
H	-1.027642	-3.244320	0.887750
B	-1.613505	-1.007760	2.039446
H	-2.478846	-1.823707	1.807146
H	-1.169586	-1.102178	3.160538
H	-2.071436	0.131966	1.908875
O	-1.304011	0.730173	-0.325891
C	-2.501284	0.867827	0.039870
C	-2.905401	2.237613	0.495609
H	-3.437374	2.177819	1.446675
H	-2.028261	2.875458	0.595722
H	-3.594686	2.671344	-0.238295
C	3.028972	-0.627534	-0.089162
C	2.978941	-2.132035	-0.420435
C	3.784039	0.064283	-1.248871
H	3.633612	-0.486782	0.820192
C	4.371635	-2.740276	-0.633922
H	2.377748	-2.254180	-1.328405
H	2.481013	-2.697952	0.362351
C	5.185648	-0.521131	-1.467291
H	3.190972	-0.056497	-2.163503
H	3.886646	1.137020	-1.077217
C	5.138230	-2.024132	-1.746562
H	4.275251	-3.809169	-0.858752
H	4.943661	-2.669797	0.302580
H	5.680041	0.007923	-2.290640
H	5.794681	-0.339370	-0.570039
H	6.150477	-2.433096	-1.847467
H	4.628811	-2.197314	-2.704587
C	1.814859	1.585454	0.319831
C	1.445468	2.297008	-0.998977
C	1.130919	2.320475	1.491392
H	2.886901	1.748921	0.490609
C	1.835712	3.778761	-0.980842
H	0.366137	2.209455	-1.147983
H	1.904830	1.794614	-1.850367
C	1.492601	3.813705	1.510249
H	0.042378	2.208459	1.425412
H	1.436242	1.890878	2.451723
C	1.165873	4.509160	0.186297
H	1.563323	4.248534	-1.933543
H	2.927336	3.873796	-0.887315
H	0.976932	4.306546	2.343079
H	2.569116	3.914802	1.708027
H	1.474643	5.560661	0.220176
H	0.076734	4.506567	0.032979
H	-1.144586	-1.350298	-1.264539
C	-3.553785	-0.153302	-0.289557
C	-3.932351	0.038570	-1.785048
C	-4.952287	-1.026129	-2.207748
C	-6.191669	-1.019007	-1.304784
C	-5.806421	-1.174593	0.171796
C	-4.799126	-0.102720	0.603868
H	-3.098475	-1.139009	-0.192586
H	-3.031535	-0.025393	-2.402253
H	-4.362459	1.038351	-1.932737
H	-5.237387	-0.863203	-3.253132
H	-4.472231	-2.012355	-2.160970
H	-6.881318	-1.815539	-1.605233
H	-6.730050	-0.070199	-1.439101
H	-6.697772	-1.123926	0.806413
H	-5.361937	-2.165626	0.332654
H	-4.498863	-0.253732	1.644479
H	-5.274785	0.884173	0.532259

C3-TS4Spri

C	1.506206	0.066681	0.001620
C	0.909208	-0.087841	1.436558
H	0.556605	0.903667	1.726534
O	0.293657	0.132498	-0.768603
B	-0.595515	-0.898125	-0.310316
N	-0.329627	-0.954600	1.284318
C	1.685271	-0.735588	2.584054
H	2.743037	-0.476882	2.592358
H	1.250864	-0.378448	3.521614
C	1.426441	-2.241828	2.440801
H	2.180268	-2.709787	1.803557
H	1.458730	-2.753063	3.406367
C	0.030622	-2.335787	1.798739
H	-0.000707	-3.044615	0.970360

H	-0.726322	-2.627926	2.524760
B	-1.524172	-0.383672	2.210307
H	-2.376457	-1.233065	2.310394
H	-1.064325	-0.043784	3.278365
H	-2.007683	0.618863	1.663356
O	-2.073889	-0.543052	-0.515973
C	-2.686618	0.518133	-0.199811
C	-2.095818	1.877736	-0.410030
H	-2.201329	2.112127	-1.477672
H	-2.635264	2.629791	0.166873
H	-1.037562	1.878623	-0.182966
C	2.327107	-1.157146	-0.533389
C	2.345085	-1.189049	-2.075924
C	3.771053	-1.307472	-0.018217
H	1.782408	-2.057143	-0.227229
C	2.982678	-2.480355	-2.602405
H	2.913220	-0.328924	-2.456397
H	1.327527	-1.085221	-2.453408
C	4.411092	-2.607263	-0.527938
H	4.374922	-0.461007	-0.368180
H	3.813999	-1.293111	1.072879
C	4.401456	-2.676353	-2.058179
H	2.992007	-2.473333	-3.698695
H	2.359127	-3.333955	-2.301566
H	5.435192	-2.692459	-0.146025
H	3.854173	-3.464503	-0.123359
H	4.819911	-3.629666	-2.401689
H	5.052786	-1.885186	-2.455842
C	2.300905	1.398377	-0.179176
C	1.413533	2.606329	-0.521892
C	3.207278	1.767976	1.014085
H	2.959727	1.253446	-1.045231
C	2.250182	3.854866	-0.828612
H	0.749754	2.825611	0.326274
H	0.777951	2.358244	-1.373297
C	4.059970	3.006719	0.707882
H	2.585087	1.978860	1.893352
H	3.862301	0.938985	1.287117
C	3.188774	4.208549	0.329482
H	1.590148	4.699744	-1.058112
H	2.846939	3.670753	-1.733046
H	4.690661	3.247343	1.571528
H	4.741522	2.776717	-0.123186
H	3.814064	5.070563	0.069571
H	2.589389	4.505672	1.201563
H	-0.495332	-1.984924	-0.822451
C	-4.174680	0.407496	0.011407
C	-4.916253	1.011068	-1.214445
C	-4.652096	-1.024230	0.281484
H	-4.406424	1.042533	0.876719
C	-6.436126	0.946718	-1.016084
H	-4.635341	0.440218	-2.109265
H	-4.606999	2.047277	-1.382100
C	-6.170830	-1.070786	0.481055
H	-4.370238	-1.655131	-0.569459
C	-6.914384	-0.479367	-0.722031
H	-6.936016	1.345575	-1.905988
H	-6.712767	1.602138	-0.178891
H	-6.488466	-2.104542	0.655147
H	-6.437073	-0.506531	1.385725
H	-7.996182	-0.485332	-0.547866
H	-6.733132	-1.109126	-1.603931
H	-4.127353	-1.418972	1.153657

C3-TS7R

C	0.594773	-0.099106	-0.042208
C	-0.106065	-0.975963	1.078628
H	-0.770190	-0.334364	1.657430
O	-0.087492	-0.467091	-1.257256
B	-1.246405	-1.228453	-1.024172
N	-0.994424	-1.957668	0.350269
C	0.754470	-1.850262	2.008092
H	1.754140	-1.445020	2.151515
H	0.278473	-1.897747	2.988458
C	0.776510	-3.259138	1.361061
H	1.789679	-3.581953	1.105577
H	0.353283	-3.997221	2.044463
C	-0.088378	-3.134129	0.098843
H	0.525662	-2.919420	-0.779259
H	-0.700793	-4.010850	-0.106269
B	-2.246049	-2.445674	1.251742
H	-2.754742	-3.391909	0.685563
H	-1.850737	-2.730181	2.358303
H	-3.055917	-1.521503	1.343179
O	-2.412892	-0.183025	-0.664660
C	-3.618870	-0.456635	-0.467321
C	-4.298843	-1.621167	-1.119350
H	-3.755807	-2.543566	-0.921623
H	-5.327755	-1.716857	-0.771774
H	-4.294699	-1.445211	-2.201818

C	0.242520	1.396308	0.224151
C	0.625763	1.988094	1.595037
C	0.664493	2.358880	-0.896357
H	-0.854802	1.387261	0.205546
C	-0.065897	3.345242	1.793709
H	1.705833	2.139921	1.665330
H	0.352281	1.309556	2.410956
C	-0.006556	3.727031	-0.712116
H	1.753246	2.495242	-0.889117
H	0.397539	1.926360	-1.863933
C	0.298149	4.322760	0.668428
H	0.205405	3.769026	2.767692
H	-1.155216	3.196958	1.807952
H	0.313204	4.416755	-1.502099
H	-1.093959	3.607703	-0.821821
H	-0.231231	5.273640	0.801767
H	1.371631	4.550829	0.730094
C	2.102106	-0.451522	-0.269542
C	2.575694	-0.278597	-1.728103
C	3.120284	0.234352	0.660919
H	2.184517	-1.528189	-0.073411
C	3.971386	-0.885912	-1.921546
H	2.606888	0.785040	-1.989462
H	1.857922	-0.746716	-2.402154
C	4.516275	-0.381522	0.494107
H	3.178518	1.299997	0.410943
H	2.814833	0.180747	1.709511
C	4.997579	-0.279414	-0.957559
H	4.298269	-0.750000	-2.959197
H	3.916721	-1.971020	-1.752177
H	5.226835	0.111161	1.168098
H	4.481595	-1.438707	0.793566
H	5.972589	-0.767096	-1.073823
H	5.144114	0.780308	-1.209829
H	-1.616708	-1.929796	-1.924300
C	-4.443946	0.587544	0.225679
H	-3.777657	1.209289	0.826955
H	-5.155919	0.088954	0.889342
C	-5.195686	1.447684	-0.811100
H	-5.877602	0.844947	-1.417720
H	-5.786061	2.210686	-0.298224
H	-4.491652	1.951010	-1.479352

C3-TS7Rpri

C	0.525872	0.074436	-0.011463
C	-0.171164	-0.308251	1.333904
H	-0.836416	0.521374	1.575892
O	-0.527089	-0.235491	-0.944244
B	-1.088729	-1.504462	-0.580187
N	-1.069632	-1.491896	1.023125
C	0.612708	-0.742826	2.574027
H	1.539895	-0.190571	2.719708
H	-0.020407	-0.560095	3.446789
C	0.817802	-2.253336	2.400537
H	1.718783	-2.460892	1.818404
H	0.927531	-2.766519	3.359569
C	-0.442621	-2.718052	1.652129
H	-0.225389	-3.450994	0.874036
H	-1.172667	-3.149620	2.336243
B	-2.515360	-1.261598	1.722062
H	-3.168262	-2.274094	1.597511
H	-2.321851	-0.962877	2.881574
H	-3.062079	-0.318953	1.147946
O	-2.572261	-1.657775	-1.004049
C	-3.532994	-0.849752	-0.924167
C	-4.895763	-1.447427	-0.785555
H	-4.827391	-2.533514	-0.758257
H	-5.339246	-1.089279	0.147129
H	-5.539975	-1.116504	-1.607601
C	1.762702	-0.796872	-0.419876
C	1.999971	-0.753706	-1.944047
C	3.093636	-0.501301	0.296813
H	1.498365	-1.835818	-0.192049
C	3.085547	-1.748257	-2.372332
H	2.306381	0.258903	-2.241051
H	1.064621	-0.963953	-2.463460
C	4.182342	-1.503359	-0.115322
H	3.433005	0.509241	0.037060
H	2.981317	-0.525248	1.382575
C	4.403758	-1.503156	-1.630946
H	3.240330	-1.689616	-3.456131
H	2.737043	-2.769135	-2.161998
H	5.117073	-1.273534	0.409422
H	3.882797	-2.510957	0.206531
H	5.150794	-2.255631	-1.909541
H	4.811398	-0.527950	-1.932805
C	0.850300	1.599181	-0.092767
C	-0.339568	2.452678	-0.563468
C	1.413901	2.193819	1.215500
H	1.629472	1.717431	-0.856985

C	0.044828	3.928794	-0.727949
H	-1.160752	2.372125	0.162618
H	-0.709036	2.058813	-1.511768
C	1.821199	3.662329	1.037988
H	0.650657	2.137450	2.001730
H	2.271467	1.620637	1.572227
C	0.641946	4.512639	0.556241
H	-0.831070	4.508996	-1.041863
H	0.782498	4.016024	-1.538087
H	2.217547	4.055160	1.981423
H	2.637620	3.724772	0.304737
H	0.954511	5.551252	0.397581
H	-0.129944	4.531467	1.338452
H	-0.576888	-2.492901	-1.042426
C	-3.386686	0.610649	-1.220928
H	-2.361278	0.895730	-1.005270
H	-3.493689	0.661800	-2.318031
C	-4.405784	1.535128	-0.556115
H	-4.325523	1.465011	0.531765
H	-4.204983	2.569082	-0.848026
H	-5.435189	1.302933	-0.843712

C3-TS7S

C	0.639579	-0.012695	-0.085971
C	-0.234380	-0.392116	1.167055
H	-0.775434	0.496830	1.490534
O	-0.185375	-0.395946	-1.196463
B	-1.413676	-0.991425	-0.846203
N	-1.280896	-1.367129	0.680800
C	0.420551	-1.107471	2.357548
H	1.459318	-0.818886	2.497220
H	-0.116970	-0.839404	3.268001
C	0.255346	-2.622960	2.074773
H	1.217509	-3.117517	1.915934
H	-0.234530	-3.116005	2.916468
C	-0.616425	-2.712877	0.809728
H	-0.012893	-2.895010	-0.081981
H	-1.388919	-3.478378	0.864106
B	-2.597620	-1.372894	1.600208
H	-3.297390	-2.295709	1.252664
H	-2.290001	-1.437428	2.767238
H	-3.186408	-0.298015	1.438910
O	-2.501239	0.197633	-0.839920
C	-3.665691	0.286809	-0.376347
C	-4.073295	1.643059	0.140807
H	-3.240389	2.122845	0.652556
H	-4.349767	2.260102	-0.723890
H	-4.937127	1.574516	0.803615
C	-4.798006	-0.673211	-0.654506
H	-5.253404	-0.921650	0.308181
H	-5.542138	-0.051247	-1.177578
C	-4.508544	-1.926842	-1.470448
H	-3.870426	-2.615201	-0.918070
H	-5.451873	-2.433080	-1.695180
H	-4.014727	-1.684883	-2.413895
H	-1.788944	-1.874465	-1.558528
C	0.919344	1.526113	-0.149086
C	-0.224223	2.337817	-0.778532
C	1.295400	2.169195	1.203324
H	1.782660	1.652953	-0.816097
C	0.172655	3.807134	-0.966717
H	-1.109300	2.280896	-0.133461
H	-0.510682	1.888862	-1.730261
C	1.718468	3.633577	1.022736
H	0.427051	2.142576	1.874241
H	2.090175	1.617874	1.707677
C	0.611157	4.453801	0.352173
H	-0.661018	4.367555	-1.406791
H	1.000270	3.864747	-1.687784
H	1.983249	4.069516	1.993141
H	2.625718	3.672218	0.403616
H	0.943809	5.484984	0.185050
H	-0.252220	4.508308	1.030722
C	1.979494	-0.825135	-0.256877
C	2.384674	-0.927082	-1.744057
C	3.211126	-0.346059	0.541025
H	1.773867	-1.849757	0.072342
C	3.578170	-1.868295	-1.946802
H	2.646001	0.072441	-2.119038
H	1.531318	-1.265598	-2.330602
C	4.400158	-1.301705	0.363433
H	3.509620	0.649919	0.191496
H	2.997988	-0.247857	1.607387
C	4.789339	-1.446489	-1.110106
H	3.842846	-1.909391	-3.009938
H	3.281817	-2.887837	-1.660928
H	5.252773	-0.944734	0.952952
H	4.131785	-2.288224	0.767501
H	5.609269	-2.165467	-1.223146
H	5.163809	-0.480799	-1.477860

C4-B5-TS1R

C	-1.865624	-0.511527	-0.090248
C	-1.426995	0.613006	0.943811
H	-0.834132	0.115387	1.696653
O	-1.000855	-0.303605	-1.206241
B	-0.113466	0.798965	-1.130867
N	-0.442453	1.548581	0.253935
C	-2.426764	1.535111	1.681200
H	-3.460898	1.278220	1.471901
H	-2.288248	1.430282	2.759809
C	-2.095025	2.979533	1.231993
H	-2.989273	3.572354	1.018632
H	-1.527599	3.492664	2.011440
C	-1.222704	2.808791	-0.009069
H	-1.821219	2.686161	-0.908646
H	-0.527260	3.630977	-0.168181
B	0.762109	1.932687	1.287464
H	1.052036	3.097945	1.154959
H	0.443589	1.645284	2.417097
H	1.770229	1.261356	1.018527
O	1.325481	0.207285	-1.034357
C	2.361696	0.877187	-0.731864
C	2.522309	2.307060	-1.170617
H	3.064457	2.286327	-2.124917
H	1.558796	2.780866	-1.326487
H	3.095415	2.891031	-0.454008
C	-1.474217	-1.907415	0.604775
C	-3.343377	-0.434553	-0.711021
C	-3.654687	0.991953	-1.218959
H	-3.714991	1.743802	-0.435456
H	-2.911513	1.302091	-1.956468
H	-4.627908	0.983074	-1.719166
C	-3.454620	-1.300176	-1.995291
H	-4.355203	-1.002685	-2.542191
H	-2.592160	-1.136227	-2.641371
H	-3.546961	-2.364328	-1.796103
C	-4.477470	-0.871172	0.237444
H	-5.441012	-0.651395	-0.235202
H	-4.456399	-1.945191	0.430694
H	-4.464757	-0.359715	1.201791
C	-2.066934	-2.059482	2.025037
H	-1.742038	-3.018961	2.441068
H	-1.724834	-1.284921	2.716286
H	-3.156027	-2.056055	2.034598
C	0.064708	-1.996976	0.723088
H	0.505851	-2.050577	-0.270838
H	0.525997	-1.155204	1.244701
H	0.328721	-2.904827	1.275064
C	-1.871619	-3.159313	-0.200839
H	-2.944927	-3.354132	-0.186401
H	-1.537475	-3.087373	-1.237482
H	-1.385523	-4.033428	0.245842
H	-0.134150	1.556507	-2.071362
C	3.554215	0.101121	-0.326458
C	4.755166	0.735491	0.028573
C	3.487584	-1.301504	-0.309291
C	5.864874	-0.018784	0.396137
H	4.822849	1.816469	0.034413
C	4.600326	-2.051401	0.053314
H	2.560840	-1.785054	-0.586638
C	5.790424	-1.412715	0.408445
H	6.786824	0.480451	0.675724
H	4.540613	-3.134916	0.059808
H	6.657646	-1.999428	0.695197

C4-B5-TS1Rpri

C	-1.241690	-0.648489	0.007378
C	-1.546707	0.647733	0.855847
H	-0.974092	0.544686	1.767338
O	-0.141656	-0.222952	-0.811922
B	-0.200503	1.171417	-1.139322
N	-0.898469	1.831439	0.144668
C	-2.965100	1.097985	1.268687
H	-3.746895	0.569550	0.729422
H	-3.129105	0.900259	2.330895
C	-3.008797	2.610742	0.964409
H	-4.009672	2.958931	0.692787
H	-2.675884	3.179443	1.835852
C	-2.009305	2.786841	-0.177462
H	-2.450765	2.518479	-1.137872
H	-1.602228	3.794520	-0.252930
B	0.142626	2.548913	1.148070
H	0.403721	3.651343	0.726922
H	-0.302032	2.548920	2.273732
H	1.186378	1.861453	1.152352
O	1.185661	1.833813	-1.294030
C	2.210838	1.745276	-0.548200
C	3.005555	3.003906	-0.366188
H	3.698589	3.076102	-1.214403

H	2.339157	3.863408	-0.361967
H	3.601845	2.980741	0.545591
C	-0.683639	-1.736773	1.042955
C	-2.391254	-1.170458	-0.991820
C	-0.380688	-3.115142	0.422948
H	0.152698	-3.721300	1.163261
H	-1.272718	-3.674011	0.137972
H	0.268313	-3.014822	-0.449507
C	-1.632854	-1.939220	2.248398
H	-2.637577	-2.251665	1.971542
H	-1.216401	-2.718672	2.894944
H	-1.719642	-1.037153	2.860000
C	0.674418	-1.277414	1.622751
H	0.944253	-1.941833	2.450507
H	1.452537	-1.348548	0.870789
H	0.676860	-0.257811	2.009946
C	-3.514466	-1.991567	-0.322855
H	-3.161779	-2.939044	0.085027
H	-4.022898	-1.448681	0.477509
H	-4.269401	-2.231127	-1.079487
C	-3.092890	-0.022270	-1.762903
H	-3.657096	0.661237	-1.131459
H	-2.386501	0.557042	-2.353567
H	-3.812056	-0.469192	-2.456499
C	-1.773954	-2.040630	-2.116229
H	-1.436783	-3.016066	-1.775781
H	-2.533177	-2.214412	-2.885791
H	-0.929973	-1.526651	-2.576700
H	-0.727216	1.502009	-2.169326
C	2.859879	0.450018	-0.281318
C	3.719372	0.270659	0.813501
C	2.691390	-0.589218	-1.209868
C	4.379644	-0.939453	0.990467
H	3.826187	1.058650	1.550528
C	3.374853	-1.789398	-1.041477
H	2.025238	-0.446504	-2.049648
C	4.212907	-1.968854	0.059838
H	5.020577	-1.084281	1.853823
H	3.244704	-2.588150	-1.764186
H	4.734395	-2.911113	0.196422

C4-B5-TS1S

C	2.032800	0.124174	-0.167027
C	1.232533	-0.495520	1.058534
H	1.122849	0.298359	1.783134
O	1.025419	0.335331	-1.156643
B	-0.278276	-0.107085	-0.836546
N	-0.191593	-0.781950	0.602207
C	1.686251	-1.758786	1.827850
H	2.648049	-2.129118	1.485763
H	1.800206	-1.515723	2.886651
C	0.566381	-2.812455	1.633600
H	0.960807	-3.809197	1.414603
H	-0.042471	-2.881705	2.536824
C	-0.277941	-2.279752	0.477925
H	0.124431	-2.587426	-0.484663
H	-1.325032	-2.573054	0.525023
B	-1.221801	-0.347327	1.791420
H	-2.224771	-1.009076	1.698248
H	-0.664474	-0.482398	2.857807
H	-1.500773	0.847741	1.667840
O	-1.143979	1.228938	-0.719524
C	-2.238270	1.454829	-0.134229
C	-2.435524	2.856501	0.370879
H	-1.474122	3.295251	0.632943
H	-2.886893	3.440902	-0.441151
H	-3.116186	2.894387	1.220709
C	3.116891	-0.803599	-0.903076
C	2.558528	1.556223	0.335721
C	3.378955	1.464985	1.643177
H	4.297778	0.892194	1.527418
H	3.663733	2.476175	1.952680
H	2.815327	1.027756	2.471513
C	3.463518	-0.249980	-2.311874
H	4.133822	0.604929	-2.290859
H	3.970872	-1.035295	-2.881605
H	2.555220	0.028437	-2.846330
C	4.444943	-0.992596	-0.143623
H	5.037729	-0.076176	-0.127206
H	4.317708	-1.325057	0.888620
H	5.044944	-1.750597	-0.658879
C	2.524577	-2.199021	-1.209406
H	2.300379	-2.795777	-0.328505
H	1.620715	-2.102018	-1.814264
H	3.251559	-2.768335	-1.796585
C	1.346793	2.480532	0.605512
H	0.811748	2.685956	-0.320766
H	0.629302	2.067586	1.318459
H	1.710347	3.426117	1.020820
C	3.409224	2.320066	-0.696383

H	4.405339	1.894243	-0.825685
H	2.910471	2.361519	-1.666466
H	3.547255	3.349090	-0.347325
H	-0.807058	-0.812581	-1.654723
C	-3.410254	0.564456	-0.251279
C	-4.433802	0.577720	0.709526
C	-3.544406	-0.241860	-1.390898
C	-5.560606	-0.216064	0.541774
H	-4.317446	1.164499	1.612930
C	-4.683843	-1.023356	-1.563160
H	-2.760785	-0.240175	-2.136673
C	-5.689644	-1.016200	-0.597199
H	-6.336010	-0.219600	1.300721
H	-4.784236	-1.638432	-2.451483
H	-6.572935	-1.633627	-0.728071

C4-B5-TS1Spri

C	-1.974451	-0.308013	-0.059696
C	-1.346228	0.662778	1.028739
H	-0.969573	0.017012	1.804074
O	-0.864547	-0.518873	-0.941133
B	0.050739	0.591809	-1.012388
N	-0.098791	1.295872	0.433301
C	-2.058328	1.817299	1.743584
H	-3.123390	1.656347	1.893046
H	-1.601564	1.901636	2.735228
C	-1.719041	3.078747	0.942427
H	-2.440135	3.241818	0.139788
H	-1.724749	3.975671	1.567723
C	-0.315534	2.796198	0.383174
H	-0.184643	3.139677	-0.643685
H	0.450678	3.266087	0.999683
B	1.152516	1.010462	1.391541
H	2.072840	1.728950	1.095080
H	0.807134	1.068574	2.550290
H	1.535568	-0.176499	1.168596
O	1.513403	0.176329	-1.169838
C	2.175168	-0.668073	-0.468190
C	1.691741	-2.088184	-0.360797
H	0.617624	-2.115331	-0.509369
H	2.176044	-2.647290	-1.171747
H	1.965955	-2.554534	0.585022
C	-3.190122	0.265908	-0.944367
C	-2.261643	-1.742117	0.639443
C	-4.365252	0.896065	-0.154111
H	-5.236669	0.949759	-0.815569
H	-4.667661	0.335399	0.727658
H	-4.149904	1.920124	0.151997
C	-2.212451	-2.904081	-0.387266
H	-1.380003	-2.784018	-1.081086
H	-2.082470	-3.851152	0.144855
H	-3.123372	-2.998555	-0.972198
C	-3.604886	-1.805167	1.389646
H	-4.470600	-1.744935	0.729952
H	-3.669663	-2.760932	1.921211
H	-3.685892	-1.013500	2.141875
C	-1.190469	-2.092650	1.711242
H	-0.175316	-1.866018	1.392129
H	-1.363096	-1.581988	2.662786
H	-1.242564	-3.163782	1.925296
C	-2.705763	1.354810	-1.928820
H	-3.583792	1.794850	-2.413919
H	-2.158838	2.165056	-1.454712
H	-2.065366	0.933359	-2.702460
C	-3.789474	-0.814018	-1.874375
H	-4.409244	-1.542289	-1.349945
H	-4.435758	-0.318047	-2.605976
H	-3.007331	-1.338207	-2.425449
H	-0.076801	1.377951	-1.909797
C	3.609372	-0.367970	-0.272864
C	4.478866	-1.298705	0.314969
C	4.111960	0.872347	-0.696671
C	5.827141	-0.994139	0.475544
H	4.106593	-2.258143	0.654007
C	5.460177	1.170819	-0.540282
H	3.428568	1.582232	-1.144234
C	6.319890	0.239785	0.047544
H	6.493001	-1.717809	0.934349
H	5.842634	2.130711	-0.871975
H	7.372137	0.475891	0.173130

C4-TS7R

C	1.418883	1.275882	-0.323119
C	1.129828	0.163737	0.751088
H	0.384433	0.538787	1.454590
O	0.723517	0.858351	-1.506972
B	-0.127214	-0.233562	-1.255632
N	0.495507	-0.977182	-0.003422
C	2.328420	-0.454758	1.485190
H	3.154969	0.251712	1.578187

H	2.026237	-0.740738	2.493491
C	2.707891	-1.715408	0.666024
H	3.723457	-1.658580	0.264832
H	2.647816	-2.606284	1.293662
C	1.677421	-1.786221	-0.472594
H	2.067737	-1.333313	-1.386909
H	1.340897	-2.796745	-0.698879
B	-0.434006	-1.904509	0.935382
H	-0.708373	-2.906202	0.309510
H	0.155547	-2.145331	1.963978
H	-1.463250	-1.281307	1.209959
O	-1.488459	0.371128	-0.644308
C	-2.511754	-0.289857	-0.350655
C	-2.912150	-1.533555	-1.084739
H	-2.066400	-2.199014	-1.233431
H	-3.707245	-2.057913	-0.554202
H	-3.286808	-1.219049	-2.067705
C	0.852283	2.618376	0.150206
C	2.891618	1.447165	-0.708613
C	-2.990204	1.549776	1.346797
H	-2.666242	2.341356	0.667764
H	-3.771652	1.949323	1.997987
H	-2.136365	1.264444	1.966776
H	-0.392244	-0.932058	-2.193157
C	-3.510059	0.341353	0.578112
H	-3.865025	-0.444740	1.252511
H	-4.378942	0.605588	-0.045355
H	1.005074	3.383506	-0.616070
H	-0.219284	2.529156	0.340162
H	1.345486	2.947800	1.071738
H	2.945150	2.120110	-1.568248
H	3.477147	1.885683	0.104687
H	3.351916	0.499659	-0.996617

C4-TS7Rpri

C	1.053263	-0.412506	0.013082
C	0.779312	0.923813	-0.782019
H	0.423298	0.621157	-1.758187
O	-0.208624	-0.630640	0.672855
B	-0.846244	0.608932	1.034730
N	-0.425254	1.606802	-0.145508
C	1.843990	2.017380	-1.010411
H	2.716162	1.887381	-0.375823
H	2.199989	1.988848	-2.042970
C	1.125753	3.347021	-0.696234
H	1.802842	4.108955	-0.299059
H	0.658713	3.745625	-1.599876
C	0.046091	2.955723	0.311800
H	0.450797	2.874876	1.321130
H	-0.801208	3.639972	0.333588
B	-1.578574	1.808286	-1.265955
H	-2.376876	2.623013	-0.865135
H	-1.068663	2.104283	-2.322318
H	-2.162350	0.715273	-1.410032
O	-2.390319	0.573844	1.026190
C	-3.175138	0.037497	0.193615
C	-4.482158	0.733514	-0.031989
H	-5.139490	0.503150	0.815760
H	-4.322335	1.809741	-0.070403
H	-4.963737	0.383117	-0.945915
C	-3.031291	-1.400246	-0.202121
H	-1.995247	-1.687689	-0.055022
H	-3.306148	-1.523271	-1.252600
C	-3.950618	-2.263020	0.691164
H	-3.796729	-3.317631	0.449470
H	-3.710638	-2.120605	1.748623
H	-5.009103	-2.033053	0.540846
C	1.237797	-1.558894	-1.090328
C	2.189794	-0.378231	1.159386
C	1.610448	-2.945629	-0.530012
H	1.528777	-3.680404	-1.338479
H	2.629682	-3.007134	-0.148367
H	0.919626	-3.246806	0.260212
C	2.287899	-1.165302	-2.159503
H	3.257167	-0.896309	-1.745705
H	2.443798	-2.017611	-2.828715
H	1.948229	-0.334085	-2.782659
C	-0.087978	-1.804521	-1.853067
H	0.136423	-2.325149	-2.789814
H	-0.749377	-2.452403	-1.279373
H	-0.639907	-0.897712	-2.102450
C	3.641443	-0.502220	0.646365
H	3.846492	-1.458223	0.165497
H	3.917135	0.292453	-0.050855
H	4.319011	-0.425651	1.503460
C	2.154709	0.910172	2.022601
H	2.352578	1.825772	1.468908
H	1.207719	1.020398	2.545404
H	2.940259	0.830363	2.780599
C	1.951785	-1.515434	2.185643

H	2.204168	-2.502845	1.806796
H	2.584852	-1.337892	3.061005
H	0.911109	-1.525972	2.510132
H	-0.635210	1.062042	2.126816

C4-TS7S

C	-1.339541	-0.257773	-0.097181
C	-0.518671	0.666557	0.903026
H	-0.219897	0.034550	1.726573
O	-0.422134	-0.497890	-1.166371
B	0.845892	0.119387	-1.063243
N	0.794038	1.052197	0.233893
C	-1.075688	1.977359	1.505926
H	-2.120341	2.135923	1.255331
H	-1.015803	1.927226	2.595331
C	-0.176297	3.119465	0.970335
H	-0.751257	3.985676	0.630183
H	0.507186	3.455229	1.752762
C	0.616175	2.490163	-0.173654
H	0.069740	2.535985	-1.112888
H	1.596408	2.939586	-0.322783
B	1.996170	1.012766	1.345513
H	2.806822	1.867061	1.070856
H	1.521684	1.166021	2.446502
H	2.548219	-0.090347	1.321767
O	1.862596	-1.051797	-0.773162
C	3.069508	-0.933179	-0.445962
C	3.718700	-2.144294	0.153800
H	2.958060	-2.869634	0.439285
H	4.394049	-2.589776	-0.587548
H	4.320483	-1.866800	1.020488
C	3.929198	0.197108	-0.943765
H	4.185909	-0.112002	-1.970686
H	3.323266	1.095491	-1.038331
C	5.200067	0.469796	-0.141984
H	5.871985	-0.392876	-0.124608
H	5.745272	1.304339	-0.589804
H	4.944548	0.743683	0.883760
C	-2.628435	0.367082	-0.823617
C	-1.586000	-1.634870	0.695015
C	-2.267474	-1.418796	2.066804
H	-3.258798	-0.976002	1.986533
H	-2.385216	-2.390593	2.557682
H	-1.676785	-0.794775	2.742736
C	-3.032288	-0.471634	-2.066978
H	-3.563427	-1.387200	-1.821403
H	-3.706673	0.126731	-2.688054
H	-2.153503	-0.723019	-2.660768
C	-3.882844	0.507573	0.061567
H	-4.318357	-0.461964	0.309421
H	-3.703699	1.041373	0.997131
H	-4.645818	1.066625	-0.491057
C	-2.302767	1.753393	-1.426218
H	-2.078171	2.522307	-0.690750
H	-1.470175	1.674672	-2.128019
H	-3.172497	2.104635	-1.989553
C	-0.231342	-2.335743	0.958257
H	0.222273	-2.656115	0.021508
H	0.496312	-1.712270	1.482855
H	-0.409128	-3.218488	1.581095
C	-2.416016	-2.675475	-0.079928
H	-3.474084	-2.417109	-0.141062
H	-2.026147	-2.821091	-1.089002
H	-2.354388	-3.635714	0.443345
H	1.241975	0.699628	-2.043825

C4-TS7Spri

C	-1.188262	-0.272861	-0.062651
C	-0.531930	0.707743	0.998337
H	-0.220594	0.076765	1.813238
O	-0.057904	-0.614112	-0.878100
B	0.924936	0.431988	-0.974472
N	0.775197	1.229111	0.418703
C	-1.189815	1.938882	1.630689
H	-2.268306	1.854282	1.744856
H	-0.764889	2.042745	2.634398
C	-0.741657	3.135292	0.783228
H	-1.428869	3.309609	-0.046597
H	-0.702111	4.058559	1.367492
C	0.653603	2.733769	0.276996
H	0.826812	3.009621	-0.764022
H	1.435097	3.191002	0.883261
B	1.963128	0.919083	1.454714
H	2.931392	1.580265	1.179169
H	1.570774	1.075060	2.589035
H	2.261264	-0.296487	1.327103
O	2.372366	-0.085864	-1.037960
C	2.941476	-0.925022	-0.272578
C	2.367566	-2.293245	-0.048724
H	1.285279	-2.272829	-0.102431

H	2.741840	-2.915775	-0.873288
H	2.721278	-2.726710	0.888134
C	4.427923	-0.762837	-0.056196
H	4.610751	-0.831291	1.021035
H	4.898720	-1.656711	-0.490909
C	5.032044	0.507146	-0.646556
H	4.585500	1.392136	-0.192038
H	6.110256	0.524771	-0.465814
H	4.860105	0.557065	-1.724363
C	-2.317893	0.335249	-1.032720
C	-1.616907	-1.642829	0.696818
C	-3.480166	1.080938	-0.328786
H	-4.313891	1.167262	-1.033932
H	-3.861091	0.582365	0.559223
H	-3.202630	2.098750	-0.054213
C	-1.565885	-2.864141	-0.257974
H	-0.668073	-2.844567	-0.875849
H	-1.558822	-3.784673	0.333292
H	-2.424226	-2.925653	-0.921624
C	-3.012563	-1.587210	1.347298
H	-3.825678	-1.513003	0.625212
H	-3.171970	-2.508295	1.918637
H	-3.097218	-0.754298	2.052831
C	-0.657268	-1.992476	1.870517
H	0.395732	-1.865329	1.625439
H	-0.861956	-1.402960	2.768792
H	-0.809987	-3.039008	2.148439
C	-1.719744	1.339967	-2.044830
H	-2.546021	1.809410	-2.589221
H	-1.141201	2.137894	-1.587130
H	-1.077950	0.839304	-2.768154
C	-2.941679	-0.751007	-1.938194
H	-3.628094	-1.414531	-1.411468
H	-3.521956	-0.255217	-2.723270
H	-2.168292	-1.347457	-2.424629
H	0.893562	1.164448	-1.922325

C5-B5-TS1R

C	-0.757696	-0.430465	0.259015
C	-0.312185	-1.453262	-0.872878
H	0.596031	-1.071403	-1.337880
O	-0.367658	-1.034437	1.482543
B	0.499937	-2.155912	1.286859
N	0.061141	-2.711046	-0.144821
C	-1.343777	-1.887371	-1.912695
H	-2.019174	-1.079137	-2.193805
H	-0.815837	-2.218423	-2.808521
C	-2.088375	-3.073783	-1.258957
H	-3.086669	-2.777314	-0.929556
H	-2.197569	-3.899789	-1.964776
C	-1.234179	-3.481676	-0.040838
H	-1.734312	-3.202931	0.886553
H	-1.000158	-4.544537	-0.005105
B	1.125254	-3.578593	-0.954099
H	1.322083	-4.612417	-0.359941
H	0.771371	-3.728996	-2.099554
H	2.182359	-2.910124	-0.992041
O	1.907997	-1.516902	0.956880
C	2.956891	-2.046769	0.467467
C	0.527738	-3.186574	2.506273
H	0.843785	-4.194367	2.218606
H	1.195042	-2.842180	3.303951
H	-0.470198	-3.278836	2.947891
C	3.827692	-1.136303	-0.313395
C	4.970365	-1.596991	-0.986785
C	3.507407	0.230601	-0.355531
C	5.773284	-0.705486	-1.690339
H	5.221450	-2.651087	-0.983490
C	4.321088	1.120724	-1.048280
C	5.452970	0.654404	-1.718870
H	4.067421	2.175461	-1.057459
H	6.086432	1.347513	-2.264150
H	2.631590	0.581128	0.171695
H	6.648803	-1.070155	-2.217785
C	-2.272969	-0.175902	0.320236
C	-3.046542	-0.813539	1.325582
C	-2.913378	0.650969	-0.580329
C	-4.410414	-0.649120	1.383968
H	-2.529666	-1.416635	2.060181
H	-2.347084	1.189740	-1.332534
C	-4.318183	0.839747	-0.552403
C	-5.091415	0.168888	0.447091
H	-4.985548	-1.143699	2.162251
C	-4.984404	1.675316	-1.487899
C	-6.498218	0.355583	0.469835
C	-6.349795	1.835567	-1.441235
H	-4.394114	2.186227	-2.243786
C	-7.114870	1.168591	-0.452981
H	-7.080317	-0.155920	1.231572
H	-6.847671	2.476046	-2.162807

H	-8.191785	1.303750	-0.426790
C	-0.017353	0.896155	0.070018
C	0.197852	1.443161	-1.223345
C	0.470324	1.568876	1.168411
C	0.896561	2.615445	-1.391822
H	-0.170804	0.918484	-2.098933
H	0.324373	1.138195	2.152382
C	1.206159	2.773375	1.031144
C	1.430757	3.310612	-0.277621
H	1.065836	3.012239	-2.388963
C	1.754510	3.452960	2.151433
C	2.194587	4.499982	-0.414040
C	2.491616	4.602742	1.987218
H	1.583360	3.042781	3.142583
C	2.715664	5.131243	0.692095
H	2.359234	4.903721	-1.409530
H	2.906997	5.110833	2.852016
H	3.299103	6.039492	0.576783
C	3.530251	-3.312956	1.041238
H	2.747076	-3.962751	1.415507
H	4.127669	-3.857846	0.312888
H	4.182447	-3.014962	1.872447

C5-B5-TS1Rpri

C	0.430654	-0.474852	-0.221671
C	0.013443	-1.215828	1.085919
H	-1.026370	-0.957418	1.283011
C	-0.244828	0.882878	-0.374153
C	-0.219069	1.483881	-1.663512
C	-0.814034	1.567530	0.673833
C	-0.774413	2.719314	-1.877119
H	0.245041	0.936751	-2.476397
H	-0.841905	1.139315	1.670066
C	-1.401107	3.431389	-0.817119
C	1.944606	-0.242865	-0.382642
C	2.593985	0.732370	0.347830
C	2.692036	-1.005940	-1.315868
H	2.031396	1.383302	1.009409
H	2.166738	-1.720972	-1.934193
C	4.744800	0.128976	-0.669962
O	-0.032446	-1.347857	-1.256622
B	-0.251022	-2.688163	-0.813572
N	0.036273	-2.673483	0.736074
C	0.884519	-1.096185	2.334816
H	1.333449	-0.108665	2.441871
H	0.257394	-1.279993	3.208605
C	1.943282	-2.214726	2.192656
H	2.922360	-1.799938	1.942353
H	2.039781	-2.774046	3.124606
C	1.448262	-3.118028	1.045289
H	2.078549	-2.991048	0.166558
H	1.418331	-4.175902	1.301683
B	-0.959084	-3.516578	1.721798
H	-1.097163	-4.621104	1.240764
H	-0.443119	-3.553963	2.817035
H	-2.013104	-2.902016	1.778215
O	-1.891083	-3.098541	-0.941611
C	-2.977801	-2.787342	-0.412100
C	0.352899	-3.867635	-1.696084
H	0.094554	-4.848041	-1.284517
H	-0.053955	-3.818520	-2.712321
H	1.442652	-3.820155	-1.784716
C	-3.412126	-1.394264	-0.234811
C	-4.270219	-1.033024	0.818767
C	-3.049137	-0.441546	-1.198551
C	-4.729873	0.273953	0.920383
H	-4.533940	-1.767152	1.572246
C	-3.567977	0.847357	-1.124394
C	-4.390458	1.211923	-0.060571
H	-3.292799	1.579095	-1.873099
H	-4.752810	2.232024	0.014539
C	-3.917667	-3.914479	-0.089255
H	-3.612300	-4.325166	0.878011
H	-4.959300	-3.596685	-0.048293
H	-3.781940	-4.701622	-0.833369
H	-2.370348	-0.724151	-1.993061
H	-5.359324	0.562694	1.755820
C	3.992163	0.935164	0.242464
C	4.049460	-0.828960	-1.450300
H	4.607003	-1.422964	-2.169446
C	6.146198	0.330549	-0.771432
C	6.776451	1.285257	-0.007344
C	6.031417	2.086771	0.892621
C	4.671897	1.916438	1.012367
H	6.712998	-0.284156	-1.465321
H	7.849083	1.429947	-0.092018
H	6.540398	2.838145	1.488491
H	4.096811	2.528980	1.701365
C	-1.415274	2.840742	0.484948

C	-2.032502	4.686353	-1.009342
C	-2.658042	5.328283	0.036507
C	-2.672756	4.743499	1.325192
C	-2.061594	3.528894	1.543203
H	-2.019206	5.131525	-2.000496
H	-3.141647	6.286884	-0.123931
H	-3.168427	5.258110	2.142606
H	-2.074925	3.073543	2.529606
H	-0.752295	3.167550	-2.866852

C5-B5-TS1S

C	0.723567	-0.312371	-0.069809
C	0.076253	-1.123640	1.130446
H	-0.508752	-0.430785	1.730535
O	-0.056210	-0.664239	-1.214827
B	-1.209980	-1.381263	-0.872378
N	-0.894464	-2.069147	0.490488
C	0.971674	-2.000010	2.003823
H	1.958638	-1.563359	2.160574
H	0.490084	-2.119091	2.975929
C	1.037791	-3.358948	1.271920
H	2.004550	-3.493725	0.781508
H	0.895268	-4.184095	1.972046
C	-0.083719	-3.319980	0.214694
H	0.343927	-3.258791	-0.787106
H	-0.759015	-4.172938	0.255235
B	-2.110731	-2.472298	1.490513
H	-2.922033	-3.086956	0.835765
H	-1.641327	-3.134858	2.391057
H	-2.574158	-1.436114	1.946258
O	-2.248837	-0.025283	-0.283541
C	-3.259164	0.109950	0.431022
C	-1.976267	-2.162031	-2.007283
H	-2.899005	-2.619657	-1.644704
H	-2.215945	-1.503064	-2.847951
H	-1.346049	-2.965124	-2.406970
C	-4.543099	-0.548712	0.109352
C	-5.461444	-0.905478	1.107262
C	-4.867619	-0.758724	-1.238032
C	-6.675227	-1.487355	0.760049
H	-5.200675	-0.778460	2.151442
C	-6.093238	-1.321133	-1.582789
H	-4.161398	-0.459715	-2.002158
C	-6.995100	-1.691577	-0.584777
H	-7.371124	-1.787795	1.536519
H	-6.342833	-1.472231	-2.628025
H	-7.946775	-2.139953	-0.852755
C	-3.223256	1.164165	1.503837
H	-3.710689	0.822860	2.418292
H	-2.192054	1.454103	1.704729
H	-3.778351	2.038750	1.142761
C	2.183804	-0.679416	-0.363635
C	2.484258	-1.559864	-1.436130
C	3.222430	-0.192106	0.403240
C	3.777123	-1.952771	-1.690776
H	1.672016	-1.898462	-2.065661
H	3.030150	0.514790	1.203301
C	4.566195	-0.579621	0.171463
C	4.855077	-1.488097	-0.895431
H	3.988718	-2.624972	-2.518034
C	5.637667	-0.093878	0.967146
C	6.200695	-1.878301	-1.122421
C	6.931725	-0.491440	0.723393
H	5.415540	0.598655	1.774473
C	7.216430	-1.393226	-0.331400
H	6.414322	-2.568023	-1.934405
H	7.742198	-0.114120	1.339412
H	8.242172	-1.698856	-0.513342
C	0.592769	1.189992	0.187538
C	0.794175	1.733309	1.484888
C	0.272010	2.038470	-0.847943
C	0.668447	3.084054	1.716599
H	1.040222	1.075911	2.313041
H	0.098158	1.622353	-1.833494
C	0.128652	3.433389	-0.644318
C	0.330186	3.974219	0.666103
H	0.821804	3.483720	2.715302
C	-0.218188	4.316252	-1.701769
C	0.179028	5.371303	0.866981
C	-0.356802	5.666069	-1.476113
H	-0.370684	3.903080	-2.694854
C	-0.156329	6.199521	-0.179013
H	0.333983	5.775723	1.863638
H	-0.620510	6.330278	-2.293485
H	-0.268209	7.266853	-0.014719

C5-B5-TS1Spri

C	0.650985	0.068096	0.309575

C	-0.142658	-0.615483	1.474687
H	-0.937874	0.067685	1.770928
O	-0.040372	-0.329607	-0.863083
B	-0.902928	-1.475283	-0.709972
N	-0.798469	-1.821082	0.861015
C	0.600954	-1.141001	2.702752
H	1.452536	-0.520469	2.981518
H	-0.095076	-1.155538	3.543507
C	1.024538	-2.575196	2.333307
H	2.075974	-2.601400	2.038962
H	0.896203	-3.253543	3.179597
C	0.138961	-2.980218	1.138890
H	0.751441	-3.167103	0.260386
H	-0.468652	-3.864350	1.329400
B	-2.192262	-2.111717	1.582190
H	-2.728103	-3.062620	1.068840
H	-2.042064	-2.179376	2.779338
H	-2.912979	-1.103566	1.354765
O	-2.413270	-1.120701	-0.970345
C	-3.228437	-0.430764	-0.271604
C	-0.686941	-2.678514	-1.746942
H	-1.334829	-3.532233	-1.517994
H	-0.953283	-2.332642	-2.752314
H	0.339641	-3.051982	-1.801779
C	-4.648593	-0.851360	-0.334650
C	-5.680845	-0.031863	0.143894
C	-4.960868	-2.099252	-0.895215
C	-7.005084	-0.452811	0.059267
H	-5.456113	0.933038	0.583177
C	-6.284111	-2.516121	-0.977738
C	-7.308669	-1.694512	-0.501031
H	-6.519464	-3.482920	-1.410971
H	-8.341783	-2.022090	-0.564902
C	-2.892030	0.986761	0.115616
H	-1.820512	1.153682	0.091508
H	-3.342965	1.640199	-0.641620
H	-3.299830	1.260291	1.088670
H	-4.151970	-2.722939	-1.253914
H	-7.799272	0.186825	0.430507
C	0.597293	1.593900	0.418481
C	0.589085	2.257250	1.674154
C	0.577746	2.346533	-0.736634
C	0.561238	3.630973	1.748027
H	0.593640	1.678194	2.591613
H	0.568320	1.837344	-1.693950
C	0.544083	3.762207	-0.696389
C	0.537538	4.424428	0.573406
H	0.550086	4.125551	2.715429
C	0.514205	4.548876	-1.878927
C	0.502062	5.843110	0.610764
C	0.481408	5.922134	-1.809884
H	0.519985	4.043140	-2.840312
C	0.475061	6.576081	-0.552944
H	0.497274	6.340483	1.576852
H	0.460725	6.512500	-2.720780
H	0.449240	7.660696	-0.513057
C	2.122430	-0.369202	0.222060
C	3.089310	0.118023	1.145480
C	2.531263	-1.237619	-0.764783
C	4.392291	-0.315295	1.103841
H	2.802151	0.861738	1.879763
H	1.816087	-1.564312	-1.507698
C	3.869282	-1.704000	-0.839420
C	4.822889	-1.247622	0.123932
H	5.116472	0.065483	1.818849
C	4.289538	-2.619979	-1.839242
C	6.156468	-1.726353	0.054262
C	5.589854	-3.068837	-1.880349
H	3.562423	-2.962791	-2.570132
C	6.532317	-2.618227	-0.924435
H	6.877303	-1.375614	0.787845
H	5.899073	-3.771667	-2.647920
H	7.555204	-2.979523	-0.968151

C5-TS7R

C	0.218962	-0.533073	-0.201171
C	-0.062312	-1.621035	0.925535
H	-0.891253	-1.272481	1.539426
O	-0.279224	-1.107042	-1.406875
B	-1.115692	-2.220130	-1.139607
N	-0.542872	-2.841545	0.192685
C	1.108277	-2.105620	1.778262
H	1.811819	-1.306393	2.013297
H	0.712354	-2.501045	2.715037
C	1.756709	-3.237260	0.950869
H	2.677426	-2.893870	0.474351
H	2.004074	-4.090840	1.585437
C	0.717205	-3.616475	-0.124393
H	1.066000	-3.329147	-1.117476

H	0.467259	-4.675853	-0.136172
B	-1.513146	-3.757859	1.098368
H	-1.739134	-4.774864	0.481205
H	-0.981084	-3.966004	2.164506
H	-2.560725	-3.129803	1.295594
O	-2.503277	-1.616102	-0.672919
C	-3.538551	-2.244973	-0.346562
C	-3.928925	-3.547738	-0.973469
H	-3.063391	-4.142380	-1.248574
H	-4.575129	-4.118262	-0.306212
H	-4.498662	-3.300719	-1.880212
H	-1.269395	-2.985156	-2.048644
C	-4.561151	-1.505868	0.469797
H	-4.951336	-2.206527	1.215443
H	-5.403294	-1.308234	-0.212324
C	-4.055593	-0.216409	1.108307
H	-3.241226	-0.426328	1.805138
H	-3.675996	0.479769	0.359041
H	-4.863213	0.270565	1.660460
C	1.706557	-0.215649	-0.413322
C	2.397375	-0.808637	-1.502874
C	2.403465	0.619681	0.435713
C	3.740615	-0.587511	-1.696549
H	1.833792	-1.424211	-2.191343
H	1.896821	1.120160	1.253859
C	3.789633	0.865603	0.268462
C	4.481417	0.243641	-0.818502
H	4.252191	-1.047522	-2.537832
C	4.515466	1.711926	1.148257
C	5.870010	0.488222	-0.981121
C	5.861496	1.928679	0.965613
H	3.986554	2.185670	1.970813
C	6.546147	1.310246	-0.109385
H	6.390156	0.013679	-1.808748
H	6.405662	2.576847	1.645694
H	7.608551	1.489798	-0.242648
C	-0.552626	0.751279	0.116543
C	-0.637677	1.253154	1.442942
C	-1.181284	1.438748	-0.897193
C	-1.340639	2.401424	1.724501
H	-0.155090	0.717336	2.254476
H	-1.129996	1.046558	-1.906421
C	-1.920784	2.621079	-0.642653
C	-2.006840	3.117458	0.697879
H	-1.402271	2.768101	2.745430
C	-2.592093	3.326665	-1.676969
C	-2.757546	4.295361	0.950518
C	-3.311481	4.466161	-1.400688
H	-2.524215	2.948189	-2.693048
C	-3.395655	4.955678	-0.073902
H	-2.819116	4.666969	1.969800
H	-3.818452	4.997051	-2.200663
H	-3.966442	5.856315	0.130394

C5-TS7Rpri

C	-0.052693	-0.521567	-0.148106
C	-0.390121	-1.213772	1.215155
H	-1.356321	-0.834220	1.545982
O	-0.669245	-1.361153	-1.121401
B	-0.860614	-2.706203	-0.664863
N	-0.584601	-2.672471	0.900834
C	0.630331	-1.170892	2.349619
H	1.177507	-0.227427	2.387914
H	0.097203	-1.297757	3.294187
C	1.554001	-2.373519	2.090202
H	2.431112	-2.063199	1.519078
H	1.904511	-2.817320	3.024905
C	0.712944	-3.369781	1.268863
H	1.230172	-3.671088	0.358244
H	0.454277	-4.266815	1.829978
B	-1.769611	-3.271873	1.840573
H	-1.914634	-4.440780	1.566831
H	-1.459843	-3.073212	2.994738
H	-2.799432	-2.637989	1.581414
O	-2.342008	-3.178147	-0.862636
C	-3.404776	-2.637036	-0.467115
C	-4.597804	-3.528621	-0.331057
H	-5.329182	-3.270609	-1.106911
H	-4.304249	-4.573013	-0.422964
H	-5.071228	-3.353916	0.637778
H	-0.247899	-3.564347	-1.241617
C	-3.616893	-1.150853	-0.420402
H	-4.498643	-0.939770	0.189588
H	-2.761131	-0.648745	0.019055
C	-3.805683	-0.620092	-1.857894
H	-4.660960	-1.083921	-2.359023
H	-3.970741	0.458730	-1.819506
H	-2.901832	-0.806242	-2.439305
C	1.453484	-0.418946	-0.429078

C	2.087529	-1.388376	-1.249240
C	2.215915	0.589988	0.123522
C	3.446920	-1.356842	-1.455334
H	1.480151	-2.151090	-1.718541
H	1.743140	1.370231	0.711903
C	3.619236	0.649434	-0.065288
C	4.255702	-0.351739	-0.866107
H	3.919549	-2.106835	-2.083810
C	4.415783	1.671705	0.515932
C	5.662088	-0.294345	-1.049681
C	5.777071	1.699178	0.320998
H	3.928649	2.431996	1.120331
C	6.406871	0.706010	-0.469006
H	6.140423	-1.056444	-1.658779
H	6.375835	2.484332	0.772334
H	7.482135	0.739423	-0.615085
C	-0.677821	0.865164	-0.263890
C	-0.896574	1.380091	-1.571324
C	-1.002498	1.642168	0.826186
C	-1.442228	2.625312	-1.755910
H	-0.630876	0.758242	-2.417880
H	-0.836527	1.280543	1.836197
C	-1.564758	2.938512	0.671027
C	-1.795109	3.442248	-0.647603
H	-1.609587	3.006304	-2.759739
C	-1.910727	3.746768	1.784436
C	-2.362103	4.733141	-0.800855
C	-2.459168	4.996504	1.603973
H	-1.735269	3.360746	2.784924
C	-2.687161	5.494629	0.299103
H	-2.534406	5.111871	-1.804583
H	-2.719383	5.605699	2.464041
H	-3.119697	6.481863	0.169490

C5-TS7S

C	-0.245081	-0.242465	-0.234249
C	0.014320	-1.360301	-1.334942
H	0.944066	-1.128103	-1.852704
O	0.035486	-0.874007	1.013169
B	0.753393	-2.079305	0.817371
N	0.248263	-2.629577	-0.564399
C	-1.111711	-1.696200	-2.312305
H	-1.679173	-0.816926	-2.618160
H	-0.670444	-2.144778	-3.203741
C	-1.984819	-2.731967	-1.569760
H	-2.905813	-2.274724	-1.201751
H	-2.258051	-3.555629	-2.232482
C	-1.130513	-3.223920	-0.383344
H	-1.545151	-2.871311	0.562335
H	-1.026226	-4.306602	-0.338898
B	1.172236	-3.684252	-1.371884
H	1.227475	-4.693806	-0.702700
H	0.673723	-3.881860	-2.457144
H	2.288922	-3.188553	-1.519692
O	2.248301	-1.626786	0.493833
C	3.257848	-2.344895	0.322226
C	4.423504	-1.675727	-0.344994
H	5.286715	-2.335412	-0.431544
H	4.111933	-1.340435	-1.336748
H	4.690550	-0.782313	0.228979
H	0.740845	-2.850667	1.733792
C	3.427885	-3.693095	0.948730
H	2.460476	-4.168453	1.087638
H	4.023061	-4.317831	0.278795
C	4.151403	-3.510238	2.302139
H	3.550116	-2.902028	2.982983
H	4.305666	-4.488776	2.762840
H	5.129749	-3.034365	2.185102
C	0.712661	0.937183	-0.436379
C	1.045967	1.411792	-1.733101
C	1.253672	1.567129	0.662448
C	1.897447	2.479172	-1.901857
H	0.632831	0.922752	-2.609909
H	1.009689	1.196286	1.651481
C	2.139633	2.664464	0.526003
C	2.472426	3.135844	-0.784588
H	2.145257	2.827847	-2.900713
C	2.714873	3.312518	1.651836
C	3.363988	4.232539	-0.917390
C	3.575182	4.373772	1.490948
H	2.458611	2.954084	2.644935
C	3.903621	4.838676	0.193506
H	3.612249	4.586263	-1.914468
H	4.006000	4.861935	2.359848
H	4.583170	5.677824	0.080401
C	-1.690606	0.267823	-0.188773
C	-2.180859	1.172925	-1.170000
C	-2.542250	-0.150898	0.809556
C	-3.487139	1.598236	-1.154271

H	-1.511402	1.546979	-1.935767
H	-2.164090	-0.810789	1.580815
C	-3.895871	0.271959	0.858781
C	-4.387707	1.159880	-0.149133
H	-3.844694	2.288268	-1.913618
C	-4.782641	-0.165528	1.877490
C	-5.743967	1.573385	-0.101924
C	-6.094076	0.251197	1.896454
H	-4.405333	-0.838574	2.642412
C	-6.579827	1.128963	0.897140
H	-6.111762	2.248675	-0.869711
H	-6.762999	-0.091919	2.679803
H	-7.616414	1.450762	0.923457

C5-TS7Spri

C	-0.100401	-0.302826	0.015642
C	-0.595144	-1.195894	1.205925
H	-1.403826	-0.658467	1.702257
O	-0.949624	-0.671628	-1.063709
B	-1.514187	-1.980674	-0.926047
N	-1.202696	-2.421395	0.576348
C	0.419238	-1.719312	2.220744
H	1.215985	-1.003413	2.428807
H	-0.106564	-1.928817	3.154580
C	0.957667	-3.021460	1.603471
H	1.879692	-2.828485	1.051883
H	1.178034	-3.765980	2.371982
C	-0.142297	-3.507565	0.641000
H	0.252774	-3.681867	-0.359238
H	-0.628561	-4.420761	0.981471
B	-2.475553	-2.903649	1.448833
H	-2.901658	-3.927686	0.970700
H	-2.132436	-3.009584	2.604724
H	-3.333542	-2.008594	1.374231
O	-3.067211	-1.969141	-1.093428
C	-3.928722	-1.337503	-0.425356
C	-3.749521	0.082243	0.021442
H	-2.715193	0.341632	0.214030
H	-4.087225	0.712319	-0.813144
H	-4.379256	0.298207	0.885145
H	-1.180927	-2.801050	-1.739166
C	-5.324815	-1.898146	-0.405447
H	-5.708021	-1.778747	0.613138
H	-5.929518	-1.218164	-1.026185
C	-5.443286	-3.339464	-0.887443
H	-5.088439	-3.439846	-1.915491
H	-4.841473	-3.997170	-0.257776
H	-6.486406	-3.663604	-0.845627
C	1.366630	-0.550277	-0.369481
C	1.671399	-1.417315	-1.451659
C	2.405230	0.031870	0.328709
C	2.974860	-1.714910	-1.773486
H	0.857026	-1.834855	-2.028749
H	2.202192	0.738385	1.126931
C	3.759634	-0.255656	0.025408
C	4.056516	-1.158420	-1.044536
H	3.191403	-2.382006	-2.603534
C	4.833917	0.323426	0.752022
C	5.413300	-1.450807	-1.341950
C	6.138707	0.019162	0.440880
H	4.605065	1.011538	1.561255
C	6.431679	-0.877568	-0.616078
H	5.633120	-2.136561	-2.155609
H	6.951354	0.466607	1.004897
H	7.465897	-1.109082	-0.851549
C	-0.311791	1.182232	0.309040
C	-0.165870	1.715011	1.617303
C	-0.632723	2.032327	-0.728134
C	-0.338233	3.059218	1.856404
H	0.076533	1.056835	2.444635
H	-0.756264	1.622291	-1.724370
C	-0.823140	3.419837	-0.515645
C	-0.671352	3.950666	0.805747
H	-0.227634	3.452800	2.863055
C	-1.162172	4.304652	-1.574159
C	-0.864255	5.341334	1.016223
C	-1.341165	5.647785	-1.338521
H	-1.274072	3.899012	-2.575747
C	-1.191276	6.171637	-0.030530
H	-0.748266	5.738746	2.020839
H	-1.597804	6.314376	-2.156130
H	-1.335428	7.233848	0.141267

C6-B5-TS1R

C	-0.990618	-0.189830	0.207797
C	-0.458787	-1.103488	-0.980493
H	0.466560	-0.673687	-1.360119
C	-0.201636	1.124141	0.259066

C	0.098150	1.684424	1.503776
C	0.229816	1.778417	-0.897049
C	0.831355	2.868447	1.604835
H	-0.224146	1.160061	2.395817
C	0.972702	2.963831	-0.827526
H	0.015735	1.358993	-1.875955
C	1.266712	3.495326	0.429360
H	1.847515	4.412622	0.497855
C	-2.493472	0.114547	0.137625
C	-3.001975	1.068960	-0.752601
C	-3.381896	-0.576960	0.959578
C	-4.372820	1.309162	-0.852336
H	-2.322614	1.645237	-1.371739
C	-4.762405	-0.348537	0.889413
H	-2.982492	-1.286983	1.673561
C	-5.243537	0.588368	-0.025555
H	-6.313975	0.770783	-0.090820
O	-0.748426	-0.940606	1.394284
B	0.189575	-1.982896	1.155200
N	-0.099770	-2.416881	-0.344011
C	-1.421218	-1.465514	-2.110351
H	-2.088028	-0.643139	-2.369817
H	-0.834220	-1.722614	-2.994013
C	-2.186101	-2.705966	-1.597643
H	-3.194708	-2.436203	-1.277300
H	-2.271525	-3.462594	-2.380778
C	-1.374390	-3.227953	-0.393735
H	-1.920852	-3.072361	0.537374
H	-1.105454	-4.280227	-0.470165
B	1.058867	-3.175075	-1.135316
H	1.197738	-4.278798	-0.661803
H	0.833632	-3.172241	-2.323269
H	2.107635	-2.517453	-0.965143
O	1.584803	-1.299886	1.062296
C	2.685738	-1.832435	0.711219
C	3.695000	-0.901846	0.162920
C	4.945103	-1.349182	-0.292393
C	3.388016	0.468589	0.108674
C	5.868942	-0.441208	-0.799754
H	5.190782	-2.404394	-0.269938
C	4.318688	1.371977	-0.391857
C	5.558081	0.920006	-0.849888
H	4.070331	2.427718	-0.418633
H	6.282586	1.625601	-1.245114
C	3.089199	-3.179850	1.239620
H	3.647945	-3.003050	2.168038
H	2.213464	-3.784614	1.456382
H	3.731040	-3.717359	0.544204
H	2.429063	0.810983	0.471193
H	6.830226	-0.794043	-1.158912
C	1.493024	3.609879	-2.087825
H	0.749285	3.579857	-2.890074
H	1.768352	4.654485	-1.918505
H	2.384825	3.086549	-2.454698
C	1.134578	3.472223	2.953978
H	1.148003	2.708556	3.736220
H	2.102572	3.982462	2.956286
H	0.375418	4.213207	3.232791
C	-4.910476	2.313484	-1.842407
H	-5.349141	1.814714	-2.715290
H	-5.695908	2.932590	-1.397265
H	-4.120554	2.977175	-2.204487
C	-5.700694	-1.088301	1.811649
H	-5.429047	-2.145601	1.891810
H	-5.668296	-0.670497	2.825109
H	-6.735285	-1.028099	1.462744
H	0.178223	-2.879948	1.951898

C6-B5-TS1S

C	-0.973403	-0.170999	0.115151
C	-0.245371	-0.679538	-1.202851
H	0.218143	0.173782	-1.692811
C	-0.982564	1.361685	0.166750
C	-0.830839	1.997167	1.400921
C	-1.146397	2.144283	-0.979531
C	-0.823048	3.389809	1.500679
H	-0.684439	1.383669	2.282370
C	-1.147598	3.543148	-0.910291
H	-1.264886	1.673574	-1.951485
C	-0.975130	4.150710	0.335401
H	-0.957612	5.236423	0.400566
C	-2.400109	-0.706775	0.282402
C	-3.469791	-0.159088	-0.436854
C	-2.646165	-1.774856	1.143578
C	-4.758839	-0.681797	-0.327792
H	-3.302248	0.694728	-1.084849
C	-3.932373	-2.312867	1.281769
H	-1.823028	-2.175455	1.722840
C	-4.975092	-1.763223	0.535457

H	-5.976969	-2.175837	0.632541
O	-0.183247	-0.674826	1.193093
B	1.097201	-1.062479	0.737742
N	0.872164	-1.562664	-0.727145
C	-1.028634	-1.536814	-2.195430
H	-2.067160	-1.219031	-2.293716
H	-0.548395	-1.452412	-3.172092
C	-0.899729	-2.979448	-1.661981
H	-1.814070	-3.284283	-1.148277
H	-0.719058	-3.683670	-2.476934
C	0.278608	-2.953506	-0.666320
H	-0.070559	-3.146735	0.349559
H	1.069028	-3.663224	-0.905259
B	2.123021	-1.571686	-1.750117
H	2.995435	-2.242349	-1.245247
H	1.739815	-2.017426	-2.811309
H	2.482476	-0.407747	-1.907092
O	1.886015	0.334956	0.525423
C	2.955391	0.718775	-0.006842
C	4.245785	0.047932	0.199312
C	5.340927	0.332210	-0.633379
C	4.409200	-0.839174	1.275146
C	6.568043	-0.278689	-0.408039
H	5.223325	1.002645	-1.476293
C	5.643692	-1.434727	1.506952
H	3.568453	-1.047451	1.922642
C	6.721953	-1.161692	0.663638
H	7.404160	-0.070945	-1.067510
H	5.764338	-2.115443	2.343165
H	7.682461	-1.635722	0.840394
C	2.895900	2.053675	-0.697969
H	3.251596	1.963360	-1.726669
H	1.865714	2.409415	-0.698389
H	3.542948	2.770325	-0.181347
C	-1.349391	4.370476	-2.156521
H	-0.909448	3.886656	-3.033914
H	-2.416324	4.514543	-2.366971
H	-0.901415	5.363058	-2.054524
C	-0.670481	4.061594	2.843624
H	-1.646950	4.219833	3.317670
H	-0.073173	3.452155	3.527700
H	-0.192113	5.041198	2.750350
C	-5.896720	-0.105479	-1.134699
H	-5.634241	0.870662	-1.551500
H	-6.162206	-0.762321	-1.972011
H	-6.797327	0.017610	-0.524506
C	-4.179219	-3.449248	2.244008
H	-4.206399	-3.090271	3.279954
H	-5.132459	-3.945376	2.041619
H	-3.385094	-4.200286	2.185355
H	1.705074	-1.795211	1.459160

C6-B5-TS1Spri

C	-0.931621	0.002459	0.049514
C	-0.198241	-0.420529	-1.267643
H	0.390685	0.435613	-1.598113
C	-1.236937	1.499464	0.072729
C	-1.282838	2.149518	1.312126
C	-1.498069	2.229330	-1.086359
C	-1.579272	3.508465	1.400771
H	-1.063883	1.575874	2.205693
C	-1.804659	3.596910	-1.026478
H	-1.461732	1.746283	-2.058208
C	-1.837516	4.219975	0.219997
H	-2.065067	5.282169	0.277450
C	-2.225255	-0.785167	0.309075
C	-3.414632	-0.460961	-0.356538
C	-2.220757	-1.862653	1.193527
C	-4.569102	-1.225872	-0.188699
H	-3.448140	0.407274	-1.006441
C	-3.366519	-2.646087	1.384780
H	-1.313222	-2.092982	1.737823
C	-4.525971	-2.326165	0.677439
H	-5.417937	-2.934536	0.810723
O	0.019118	-0.273884	1.069795
B	0.972246	-1.286860	0.713061
N	0.775772	-1.495992	-0.865721
C	-1.002300	-1.016334	-2.421459
H	-1.985508	-0.557339	-2.532697
H	-0.443526	-0.857583	-3.346509
C	-1.094702	-2.518606	-2.102216
H	-2.024081	-2.738181	-1.573459
H	-1.075915	-3.122292	-3.012819
C	0.112960	-2.821957	-1.194882
H	-0.195919	-3.304555	-0.268504
H	0.857690	-3.452800	-1.679476
B	2.116479	-1.366891	-1.731163
H	2.872784	-2.262483	-1.442225
H	1.838734	-1.282306	-2.905459

H	2.658699	-0.288530	-1.390209
O	2.427792	-0.834520	0.947459
C	3.048636	0.123774	0.373887
C	4.514472	-0.035695	0.270813
C	5.352412	1.045275	-0.041137
C	5.076702	-1.301666	0.500956
C	6.730297	0.863643	-0.116518
H	4.934807	2.028571	-0.222276
C	6.452475	-1.479114	0.421955
C	7.281844	-0.397627	0.113813
H	6.881059	-2.460657	0.596621
H	8.356583	-0.538263	0.051344
C	2.432082	1.491761	0.256469
H	1.349358	1.436637	0.279714
H	2.751582	2.063288	1.137591
H	2.772960	2.015767	-0.635832
H	4.415956	-2.128068	0.729357
H	7.373102	1.704603	-0.355596
C	-2.097167	4.364782	-2.292432
H	-2.138041	5.441114	-2.105956
H	-1.333321	4.182118	-3.055714
H	-3.060053	4.066107	-2.723707
C	-1.637000	4.203790	2.738917
H	-1.085027	5.149525	2.722500
H	-2.671105	4.439553	3.017126
H	-1.216230	3.577902	3.529947
C	-5.847387	-0.865120	-0.905436
H	-6.580394	-0.431533	-0.214702
H	-5.667199	-0.133249	-1.697387
H	-6.313750	-1.746558	-1.357788
C	-3.341403	-3.800723	2.356374
H	-2.400385	-4.355527	2.291030
H	-3.435159	-3.447235	3.390330
H	-4.163077	-4.498106	2.170884
H	0.912168	-2.320903	1.327387

C6-TS4R

C	-1.066782	-0.178243	0.174565
C	-0.749691	-1.071077	-1.106224
H	0.166976	-0.703648	-1.562867
C	-0.126741	1.033992	0.201229
C	0.464392	1.417296	1.406775
C	0.175982	1.755760	-0.957358
C	1.360750	2.487058	1.463592
H	0.241186	0.840168	2.296331
C	1.055956	2.843853	-0.928466
H	-0.252886	1.461446	-1.911247
C	1.643095	3.195207	0.289246
H	2.338615	4.030878	0.323611
C	-2.529789	0.277894	0.274181
C	-3.024529	1.335918	-0.498416
C	-3.402522	-0.386133	1.136007
C	-4.368523	1.708470	-0.443402
H	-2.356536	1.891183	-1.147251
C	-4.753640	-0.027078	1.220468
H	-3.011250	-1.180798	1.759267
C	-5.224388	1.014270	0.419862
H	-6.272864	1.298984	0.474559
O	-0.797262	-1.021122	1.291614
B	-0.009628	-2.137670	0.908003
N	-0.456164	-2.446261	-0.573351
C	-1.851058	-1.276654	-2.144111
H	-2.453721	-0.380622	-2.295529
H	-1.382538	-1.540466	-3.094063
C	-2.681808	-2.463247	-1.610661
H	-3.610897	-2.112265	-1.156547
H	-2.939433	-3.154172	-2.416413
C	-1.799146	-3.143012	-0.543566
H	-2.233649	-3.022344	0.449964
H	-1.632201	-4.203240	-0.725609
B	0.554958	-3.271254	-1.515902
H	0.633154	-4.397053	-1.076678
H	0.157001	-3.234727	-2.658363
H	1.662764	-2.719299	-1.468091
O	1.450194	-1.582977	0.728489
C	2.491505	-2.196115	0.381172
C	2.759213	-3.620139	0.753286
H	3.421844	-3.597295	1.630075
H	1.847607	-4.156290	0.999874
H	3.285145	-4.130260	-0.053715
C	1.356725	3.620022	-2.187140
H	1.329317	2.973646	-3.069580
H	0.622224	4.418990	-2.346575
H	2.343529	4.089372	-2.139902
C	2.052898	2.841618	2.757127
H	1.469352	2.516831	3.622630
H	3.034861	2.355577	2.823334
H	2.220130	3.919651	2.841136
C	-4.894399	2.826050	-1.310928

H	-5.441649	2.432830	-2.176435
H	-5.585990	3.469714	-0.758159
H	-4.081412	3.450401	-1.691610
C	-5.669061	-0.740966	2.185115
H	-5.522354	-0.378281	3.209706
H	-6.720693	-0.586097	1.928309
H	-5.475457	-1.818114	2.194881
H	-0.029196	-3.071405	1.659719
C	3.626468	-1.331039	-0.096426
C	3.181365	-0.289955	-1.138764
C	4.213010	-0.612637	1.153555
H	4.401827	-1.979930	-0.520766
C	4.325155	0.654882	-1.518719
H	2.356667	0.288183	-0.716249
H	2.795208	-0.809086	-2.020816
C	5.361997	0.322576	0.761079
H	3.404821	-0.034672	1.616289
H	4.554675	-1.348042	1.891177
C	4.914881	1.348859	-0.285785
H	3.954231	1.400115	-2.231032
H	5.115401	0.091880	-2.035009
H	5.738834	0.827415	1.658071
H	6.194926	-0.272355	0.361856
H	5.757683	1.987974	-0.572308
H	4.149896	2.001864	0.152391

C6-TS4S

C	1.082774	-0.030145	-0.024141
C	0.449537	-0.564577	1.302857
H	-0.252177	0.192168	1.653357
C	1.173994	1.494201	-0.044165
C	1.141021	2.145602	-1.283863
C	1.318149	2.252164	1.116658
C	1.238583	3.533077	-1.370331
H	1.020839	1.547873	-2.180721
C	1.426068	3.649986	1.058972
H	1.344724	1.767223	2.087737
C	1.379407	4.272987	-0.186927
H	1.452811	5.356885	-0.242407
C	2.467545	-0.620711	-0.330050
C	3.613768	-0.139172	0.316216
C	2.599086	-1.662681	-1.246137
C	4.863613	-0.719265	0.100081
H	3.536269	0.710622	0.986513
C	3.843421	-2.259423	-1.489444
H	1.721974	-2.007307	-1.780527
C	4.961456	-1.787448	-0.801394
H	5.931211	-2.247014	-0.979505
O	0.154398	-0.438168	-1.027720
B	-0.651368	-1.551586	-0.630887
N	-0.363145	-1.767479	0.910759
C	1.359909	-1.045591	2.431436
H	2.274471	-0.456973	2.513970
H	0.812362	-0.962098	3.371926
C	1.646875	-2.525350	2.108311
H	2.619487	-2.629379	1.622702
H	1.657884	-3.131190	3.017095
C	0.529274	-2.969442	1.143557
H	0.944412	-3.300081	0.191952
H	-0.093372	-3.768054	1.544644
B	-1.660552	-1.903805	1.888571
H	-2.326798	-2.834990	1.493385
H	-1.251737	-2.055861	3.019185
H	-2.289659	-0.849494	1.812041
O	-2.199091	-1.241277	-0.758470
C	-2.891493	-0.300404	-0.306391
C	-2.381349	1.096415	-0.147437
H	-1.362066	1.192843	-0.507339
H	-3.043106	1.764823	-0.710569
H	-2.446943	1.395080	0.901221
C	6.085884	-0.217176	0.829287
H	6.400263	-0.921941	1.608513
H	6.934131	-0.091111	0.148609
H	5.893414	0.744794	1.311896
C	3.963181	-3.375743	-2.498036
H	3.146750	-4.097015	-2.391811
H	3.917785	-2.987792	-3.522749
H	4.909198	-3.913265	-2.389722
C	1.595050	4.450077	2.327425
H	2.584038	4.284830	2.771046
H	1.491150	5.522006	2.140207
H	0.853864	4.164411	3.081407
C	1.208226	4.231617	-2.707661
H	0.497495	5.065008	-2.707466
H	2.191167	4.647581	-2.958338
H	0.925386	3.543933	-3.508640
H	-0.554793	-2.550948	-1.290466
C	-4.353082	-0.598748	-0.133115
C	-5.050118	0.235212	0.952818
C	-5.061092	-0.424460	-1.504238

H	-4.401316	-1.658705	0.137429
C	-6.531280	-0.148430	1.056671
H	-4.974872	1.302852	0.708923
H	-4.541485	0.081335	1.909350
C	-6.545411	-0.792961	-1.391391
H	-4.967487	0.619773	-1.832319
H	-4.562713	-1.047257	-2.253376
C	-7.247512	0.010929	-0.290233
H	-7.018724	0.463280	1.823454
H	-6.609552	-1.191559	1.390570
H	-7.036396	-0.631501	-2.357521
H	-6.629397	-1.864870	-1.169212
H	-8.294180	-0.301003	-0.204585
H	-7.258052	1.073709	-0.570097

C6-TS7R

C	-0.365891	-0.172725	0.155445
C	0.109582	-1.051227	-1.081479
H	0.970092	-0.565622	-1.537504
C	0.337412	1.188726	0.141317
C	0.730359	1.761845	1.352792
C	0.583696	1.882403	-1.045693
C	1.370710	3.002396	1.389964
H	0.546487	1.211379	2.268180
C	1.227311	3.125654	-1.040363
H	0.290721	1.454288	-2.000543
C	1.616398	3.671275	0.184337
H	2.121978	4.634230	0.201785
C	-1.886099	0.028175	0.233485
C	-2.542967	0.946868	-0.594612
C	-2.641915	-0.725108	1.130953
C	-3.930594	1.091048	-0.560255
H	-1.968158	1.571505	-1.269809
C	-4.034953	-0.593281	1.195889
H	-2.128764	-1.407199	1.797816
C	-4.665799	0.309708	0.339459
H	-5.747578	0.417226	0.378768
O	0.038625	-0.906266	1.310715
B	0.978479	-1.909592	0.976308
N	0.599579	-2.344161	-0.490683
C	-0.926235	-1.462600	-2.126201
H	-1.660020	-0.679054	-2.316000
H	-0.405718	-1.679505	-3.060506
C	-1.569415	-2.748474	-1.561890
H	-2.560471	-2.540635	-1.152837
H	-1.677197	-3.504944	-2.342186
C	-0.627089	-3.227789	-0.438566
H	-1.101421	-3.112050	0.537138
H	-0.303214	-4.261130	-0.550195
B	1.723392	-3.051585	-1.406004
H	1.965240	-4.133654	-0.917694
H	1.314368	-3.134610	-2.541706
H	2.732413	-2.336951	-1.398926
O	2.360257	-1.152935	0.765901
C	3.472403	-1.653373	0.479123
C	3.896321	-3.013012	0.941711
H	4.347503	-2.880556	1.935027
H	3.055307	-3.694861	1.020812
H	4.651072	-3.429243	0.274266
C	1.532000	3.832748	-2.338457
H	2.442646	3.433552	-2.801984
H	0.720476	3.707363	-3.061923
H	1.686099	4.904133	-2.183427
C	1.765718	3.623806	2.707494
H	1.943023	2.860095	3.469828
H	2.672537	4.228489	2.610223
H	0.974458	4.283196	3.084755
C	-4.628366	2.056807	-1.486766
H	-5.086818	1.532639	-2.334311
H	-5.428582	2.597672	-0.971776
H	-3.929468	2.792573	-1.893979
C	-4.826815	-1.396850	2.198593
H	-4.749176	-0.961649	3.202306
H	-5.887736	-1.431456	1.935744
H	-4.458337	-2.425289	2.265091
H	1.101673	-2.797661	1.771099
C	4.511896	-0.732186	-0.094978
H	5.035172	-1.282700	-0.883903
H	5.257953	-0.586997	0.702453
C	3.970406	0.603271	-0.595030
H	3.264042	0.446632	-1.413126
H	3.443331	1.144748	0.192149
H	4.790161	1.226036	-0.962412

C6-TS7Rpri

C	0.234070	-0.102285	0.011855
C	-0.091623	-1.092274	1.184693
H	-1.033701	-0.785716	1.634915
C	-0.428682	1.271551	0.158332
C	-0.407066	2.102460	-0.968562

C	-1.071853	1.719422	1.310719
C	-1.023923	3.352317	-0.960529
H	0.091978	1.749693	-1.865192
C	-1.694168	2.975201	1.353521
H	-1.082406	1.111550	2.209572
C	-1.666650	3.775467	0.211546
H	-2.147972	4.750560	0.232719
C	1.746027	0.039254	-0.263756
C	2.613324	0.892885	0.459156
C	2.293973	-0.761852	-1.272159
C	3.983701	0.844757	0.174974
C	3.659647	-0.781479	-1.540669
H	1.629855	-1.383288	-1.856659
C	4.535630	0.018716	-0.807512
H	4.647627	1.491789	0.744018
H	4.043177	-1.427345	-2.326077
O	-0.398650	-0.703893	-1.121585
B	-0.635186	-2.106523	-0.983354
N	-0.356781	-2.436203	0.547320
C	0.957568	-1.365904	2.262867
H	1.545264	-0.486634	2.521900
H	0.437737	-1.705779	3.161654
C	1.824432	-2.498504	1.691779
H	2.675135	-2.087527	1.145339
H	2.214279	-3.144389	2.482538
C	0.899704	-3.270028	0.734805
H	1.369653	-3.433887	-0.234403
H	0.594294	-4.235270	1.136950
B	-1.569629	-3.172905	1.334771
H	-1.750248	-4.257331	0.832223
H	-1.282498	-3.213825	2.510971
H	-2.582741	-2.470858	1.195182
O	-2.127184	-2.477528	-1.271856
C	-3.168205	-2.024196	-0.727107
C	-4.378411	-2.903143	-0.779606
H	-5.048961	-2.525770	-1.562783
H	-4.091008	-3.929242	-1.003321
H	-4.917804	-2.855438	0.168083
C	-2.351028	3.453217	2.625264
H	-2.944250	2.659922	3.091335
H	-1.602285	3.769918	3.361317
H	-3.010162	4.305179	2.438533
C	-1.032107	4.215628	-2.197931
H	-1.933586	4.035829	-2.796577
H	-1.017178	5.279777	-1.944538
H	-0.169878	4.002988	-2.835654
C	2.169013	1.909531	1.489766
H	1.671008	2.758737	1.011904
H	1.465965	1.514371	2.223735
H	3.037899	2.290739	2.032588
C	6.017904	0.028645	-1.085463
H	6.292314	0.863144	-1.742410
H	6.597136	0.139099	-0.163592
H	6.336298	-0.893960	-1.578770
H	-0.049583	-2.830696	-1.744318
C	-3.350014	-0.577724	-0.359693
H	-4.252392	-0.481837	0.249439
H	-2.506997	-0.218410	0.220564
C	-3.452571	0.282981	-1.635653
H	-4.286157	-0.024182	-2.275310
H	-3.604632	1.325277	-1.345287
H	-2.519126	0.216888	-2.195210

C6-TS7S

C	-0.386321	-0.182924	0.094607
C	0.144761	-0.952326	-1.190051
H	0.824423	-0.299628	-1.732936
C	0.137173	1.260717	0.110482
C	0.494388	1.835788	1.331707
C	0.261096	2.022801	-1.054647
C	0.988775	3.139527	1.400241
H	0.413391	1.232594	2.228555
C	0.748746	3.335484	-1.017004
H	-0.012463	1.601309	-2.017864
C	1.118375	3.875748	0.216346
H	1.514450	4.888098	0.257258
C	-1.913204	-0.176603	0.232906
C	-2.708627	0.676810	-0.542294
C	-2.533624	-1.046077	1.128449
C	-4.100407	0.650759	-0.450647
H	-2.240724	1.382688	-1.220190
C	-3.929216	-1.085473	1.249875
H	-1.913532	-1.686899	1.743857
C	-4.698069	-0.238839	0.451107
H	-5.782301	-0.263376	0.535918
O	0.153094	-0.898899	1.203629
B	1.283855	-1.653848	0.813569
N	0.972459	-2.098040	-0.666729
C	-0.870465	-1.599029	-2.128360
H	-1.755402	-0.979700	-2.279678

H	-0.388776	-1.758002	-3.095678
C	-1.197582	-2.948191	-1.466830
H	-2.076673	-2.851318	-0.826413
H	-1.407514	-3.721528	-2.209735
C	0.041013	-3.297293	-0.618003
H	-0.232627	-3.493316	0.419890
H	0.590776	-4.156700	-0.998698
B	2.202603	-2.421169	-1.646441
H	2.801189	-3.376111	-1.205110
H	1.780611	-2.610738	-2.765530
H	2.923389	-1.416798	-1.676169
O	2.455481	-0.583350	0.618747
C	3.575213	-0.555120	0.057264
C	3.930648	0.734046	-0.630171
H	4.682988	0.580929	-1.405526
H	3.034605	1.202503	-1.037044
H	4.350521	1.408345	0.127739
C	-4.948518	1.551876	-1.314811
H	-5.417039	0.991433	-2.132979
H	-5.755937	2.014093	-0.737835
H	-4.351315	2.351271	-1.761793
C	-4.576346	-2.016363	2.246206
H	-5.653417	-2.097948	2.076665
H	-4.146171	-3.021597	2.189432
H	-4.427335	-1.661209	3.272831
C	0.851617	4.149760	-2.283739
H	-0.085958	4.680920	-2.489102
H	1.642040	4.902470	-2.212539
H	1.061490	3.516387	-3.150925
C	1.360796	3.750523	2.729292
H	1.647562	2.983050	3.453530
H	2.191967	4.455237	2.628850
H	0.516388	4.303938	3.158112
H	1.611270	-2.520858	1.564740
C	4.694228	-1.529309	0.323050
H	5.045667	-1.892168	-0.647120
H	5.510554	-0.901340	0.713990
C	4.408930	-2.681000	1.279408
H	5.336716	-3.224469	1.478894
H	4.011110	-2.319314	2.230446
H	3.687700	-3.374645	0.848307

C6-TS7Spri

C	0.200050	0.004085	-0.042766
C	-0.530359	-0.441993	1.269111
H	-1.122066	0.404813	1.617133
C	0.488938	1.504153	-0.051206
C	0.532640	2.166510	-1.284642
C	0.739027	2.225153	1.115458
C	0.815363	3.528722	-1.359302
H	0.324854	1.598816	-2.184651
C	1.032230	3.596706	1.069582
H	0.706301	1.731878	2.082294
C	1.063288	4.231511	-0.170528
H	1.282467	5.295978	-0.217322
C	1.500954	-0.767294	-0.312014
C	2.687232	-0.436541	0.355932
C	1.507495	-1.836192	-1.206735
C	3.849881	-1.186859	0.179686
H	2.711540	0.425326	1.014634
C	2.661818	-2.604847	-1.406818
H	0.602578	-2.071936	-1.753061
C	3.818251	-2.278784	-0.697551
H	4.716864	-2.875660	-0.837791
O	-0.747521	-0.272410	-1.068811
B	-1.685948	-1.292899	-0.716834
N	-1.502422	-1.515448	0.853674
C	0.280221	-1.050501	2.411624
H	1.262137	-0.589076	2.524633
H	-0.275858	-0.906182	3.340345
C	0.376672	-2.547765	2.071765
H	1.305365	-2.757025	1.537515
H	0.362656	-3.163485	2.974301
C	-0.833046	-2.843110	1.164811
H	-0.524563	-3.314462	0.232329
H	-1.575085	-3.480822	1.643649
B	-2.846056	-1.402110	1.743487
H	-3.581730	-2.313120	1.446559
H	-2.536207	-1.386119	2.913533
H	-3.381351	-0.315206	1.465375
O	-3.161599	-0.828971	-0.948922
C	-3.760619	0.158332	-0.445325
C	-3.127902	1.506335	-0.266975
H	-2.062217	1.456984	-0.076880
H	-3.257987	2.034676	-1.221643
H	-3.642521	2.074643	0.508617
C	5.124390	-0.820293	0.900114
H	5.590412	-1.698875	1.358445
H	5.859554	-0.388649	0.210553
H	4.939347	-0.085458	1.688136

C	2.648455	-3.750221	-2.389506
H	1.715046	-4.317992	-2.326396
H	2.734161	-3.385603	-3.420284
H	3.479713	-4.438548	-2.213363
C	1.310314	4.355054	2.344419
H	2.249381	4.026044	2.804692
H	1.389667	5.429567	2.160334
H	0.517979	4.196291	3.083866
C	0.862546	4.240719	-2.689081
H	0.202136	5.114908	-2.697810
H	1.874120	4.600737	-2.909543
H	0.559343	3.579219	-3.504439
H	-1.643046	-2.315137	-1.348501
C	-5.262513	0.086285	-0.379143
H	-5.566962	0.517375	0.580003
H	-5.629859	0.788052	-1.144659
C	-5.845925	-1.307372	-0.581803
H	-5.562226	-1.713508	-1.555161
H	-5.473499	-1.986181	0.187459
H	-6.936928	-1.268789	-0.524662

C7-B5-TS1R

C	-0.642839	-0.564188	0.040815
C	-0.192114	-1.161811	-1.363524
H	0.745765	-0.690377	-1.653260
C	0.232312	0.643890	0.387770
C	0.589743	1.587221	-0.579908
C	0.695834	0.805350	1.693320
C	1.395508	2.685519	-0.263035
H	0.250559	1.464645	-1.603681
C	1.527564	1.875956	2.035562
H	0.432901	0.049370	2.422904
C	1.860773	2.810918	1.048716
H	2.502326	3.650850	1.307069
C	-2.124994	-0.169781	0.101446
C	-3.047493	-1.027349	0.701998
C	-2.584351	1.025226	-0.459383
C	-4.413413	-0.724429	0.720063
H	-2.677337	-1.931539	1.168086
C	-3.941096	1.357084	-0.455808
H	-1.876201	1.718888	-0.895617
C	-4.844852	0.467781	0.132671
H	-5.904715	0.712995	0.140558
O	-0.426426	-1.611393	0.981101
B	0.442625	-2.604483	0.448738
N	0.106825	-2.611522	-1.104168
C	-1.200619	-1.165658	-2.511135
H	-1.826110	-0.272532	-2.518907
H	-0.650881	-1.207473	-3.453330
C	-2.021810	-2.459165	-2.313930
H	-3.004103	-2.236394	-1.892050
H	-2.172097	-2.974699	-3.265115
C	-1.209719	-3.322120	-1.326145
H	-1.725872	-3.402954	-0.368531
H	-0.997228	-4.324721	-1.693454
B	1.212450	-3.185660	-2.099970
H	1.287861	-4.383210	-1.954242
H	0.983614	-2.837690	-3.234983
H	2.302733	-2.663154	-1.776918
O	1.875663	-2.006197	0.502984
C	2.935258	-2.498891	-0.003313
C	3.243220	-3.963272	0.137139
H	3.823589	-4.081201	1.061534
H	2.327164	-4.541658	0.212221
H	3.833698	-4.337923	-0.696896
C	-5.404855	-1.678784	1.363483
C	-4.412321	2.660287	-1.078453
C	2.108188	1.987046	3.434378
C	1.750267	3.713761	-1.323085
H	0.382070	-3.678543	0.980508
C	-4.114606	2.701342	-2.586617
H	-3.035253	2.676593	-2.772767
H	-4.561377	1.844907	-3.100428
H	-4.508719	3.618171	-3.037733
C	-3.801554	3.878240	-0.365194
H	-4.031421	3.863896	0.704144
H	-2.711454	3.889435	-0.470806
H	-4.188087	4.811196	-0.789167
C	-5.143316	-1.830128	2.870927
H	-4.157171	-2.268796	3.054810
H	-5.174854	-0.859398	3.374375
H	-5.892437	-2.482548	3.332211
C	-5.399869	-3.047406	0.662390
H	-4.425879	-3.537020	0.766872
H	-6.154970	-3.711212	1.096690
H	-5.608578	-2.942823	-0.406744
C	1.016972	2.006530	4.515451
H	0.305859	2.819819	4.343193
H	0.454261	1.067731	4.525412
H	1.459319	2.140059	5.508202

C	3.119735	0.854548	3.686174
H	3.915611	0.863743	2.935330
H	3.576751	0.951296	4.676811
H	2.627248	-0.122062	3.633353
C	2.484388	3.079918	-2.516185
H	1.839666	2.370164	-3.044864
H	2.789940	3.847587	-3.234729
H	3.375500	2.535388	-2.191722
C	0.498708	4.477830	-1.788924
H	-0.012343	4.948711	-0.943854
H	0.762111	5.257751	-2.511161
H	-0.213161	3.800932	-2.274158
H	2.430064	4.439139	-0.857658
H	2.651272	2.939565	3.488534
H	-6.404765	-1.243415	1.238894
H	-5.501425	2.708281	-0.951418
C	4.010962	-1.527669	-0.295284
C	3.774238	-0.164307	-0.049772
C	5.262848	-1.934234	-0.782870
C	4.778347	0.770081	-0.276629
C	6.260052	-0.992979	-1.019252
C	6.021176	0.359488	-0.764319
H	2.811331	0.145978	0.329307
H	5.458141	-2.980223	-0.987698
H	4.583363	1.816657	-0.068297
H	7.223677	-1.313335	-1.401741
H	6.803166	1.090520	-0.945725

C7-B5-TS1S

C	-0.973403	-0.170999	0.115151
C	-0.245371	-0.679538	-1.202851
H	0.218143	0.173782	-1.692811
C	-0.982564	1.361685	0.166750
C	-0.830839	1.997167	1.400921
C	-1.146397	2.144283	-0.979531
C	-0.823048	3.389809	1.500679
H	-0.684439	1.383669	2.282370
C	-1.147598	3.543148	-0.910291
H	-1.264886	1.673574	-1.951485
C	-0.975130	4.150710	0.335401
H	-0.957612	5.236423	0.400566
C	-2.400109	-0.706775	0.282402
C	-3.469791	-0.159088	-0.436854
C	-2.646165	-1.774856	1.143578
C	-4.758839	-0.681797	-0.327792
H	-3.302248	0.694728	-1.084849
C	-3.932373	-2.312867	1.281769
H	-1.823028	-2.175455	1.722840
C	-4.975092	-1.763223	0.535457
H	-5.976969	-2.175837	0.632541
O	-0.183247	-0.674826	1.193093
B	1.097201	-1.062479	0.737742
N	0.872164	-1.562664	-0.727145
C	-1.028634	-1.536814	-2.195430
H	-2.067160	-1.219031	-2.293716
H	-0.548395	-1.452412	-3.172092
C	-0.899729	-2.979448	-1.661981
H	-1.814070	-3.284283	-1.148277
H	-0.719058	-3.683670	-2.476934
C	0.278608	-2.953506	-0.666320
H	-0.070559	-3.146735	0.349559
H	1.069028	-3.663224	-0.905259
B	2.123021	-1.571686	-1.750117
H	2.995435	-2.242349	-1.245247
H	1.739815	-2.017426	-2.811309
H	2.482476	-0.407747	-1.907092
O	1.886015	0.334956	0.525423
C	2.955391	0.718775	-0.006842
C	4.245785	0.047932	0.199312
C	5.340927	0.332210	-0.633379
C	4.409200	-0.839174	1.275146
C	6.568043	-0.278689	-0.408039
H	5.223325	1.002645	-1.476293
C	5.643692	-1.434727	1.506952
H	3.568453	-1.047451	1.922642
C	6.721953	-1.161692	0.663638
H	7.404160	-0.070945	-1.067510
H	5.764338	-2.115443	2.343165
H	7.682461	-1.635722	0.840394
C	2.895900	2.053675	-0.697969
H	3.251596	1.963360	-1.726669
H	1.865714	2.409415	-0.698389
H	3.542948	2.770325	-0.181347
C	-1.349391	4.370476	-2.156521
H	-0.909448	3.886656	-3.033914
H	-2.416324	4.514543	-2.366971
H	-0.901415	5.363058	-2.054524
C	-0.670481	4.061594	2.843624
H	-1.646950	4.219833	3.317670
H	-0.073173	3.452155	3.527700

H	-0.192113	5.041198	2.750350
C	-5.896720	-0.105479	-1.134699
H	-5.634241	0.870662	-1.551500
H	-6.162206	-0.762321	-1.972011
H	-6.797327	0.017610	-0.524506
C	-4.179219	-3.449248	2.244008
H	-4.206399	-3.090271	3.279954
H	-5.132459	-3.945376	2.041619
H	-3.385094	-4.200286	2.185355
H	1.705074	-1.795211	1.459160

C7-B5-TS1Spri

C	-0.265532	0.322649	-0.134095
C	-0.626693	0.838875	-1.569354
H	-1.535609	0.321436	-1.878755
C	-0.734967	-1.114702	0.076237
C	-1.184446	-1.499634	1.340987
C	-0.683567	-2.064431	-0.946620
C	-1.584953	-2.814345	1.592831
H	-1.229103	-0.746114	2.118472
C	-1.052893	-3.394393	-0.717504
H	-0.346673	-1.774777	-1.936299
C	-1.504887	-3.749730	0.555429
H	-1.803087	-4.778765	0.744094
C	1.233499	0.422899	0.185707
C	2.137296	-0.533624	-0.285722
C	1.718887	1.505092	0.920485
C	3.511716	-0.397100	-0.080127
H	1.765847	-1.405787	-0.810601
C	3.090704	1.671063	1.140929
H	1.012208	2.221523	1.318601
C	3.973712	0.717684	0.626719
H	5.042497	0.838799	0.789573
O	-1.005987	1.172019	0.730425
B	-1.323702	2.448266	0.155424
N	-0.978094	2.294017	-1.404229
C	0.430442	0.802540	-2.671820
H	1.072924	-0.077193	-2.615017
H	-0.081423	0.791611	-3.636847
C	1.222622	2.109388	-2.499637
H	2.099728	1.941590	-1.872024
H	1.567146	2.498562	-3.460839
C	0.254684	3.086205	-1.806194
H	0.701076	3.527615	-0.916152
H	-0.070785	3.892414	-2.463086
B	-2.175285	2.679144	-2.396431
H	-2.414771	3.858758	-2.302455
H	-1.918806	2.290433	-3.512911
H	-3.186265	2.053843	-1.997797
O	-2.822494	2.788093	0.272855
C	-3.808052	2.164800	-0.249718
C	-3.932870	0.669313	-0.138795
H	-2.961967	0.201613	-0.021272
H	-4.508879	0.461839	0.772563
H	-4.465282	0.242654	-0.988082
C	4.474436	-1.442651	-0.616433
C	3.604163	2.870385	1.918865
C	-0.950252	-4.425124	-1.828293
C	-2.100414	-3.216688	2.963807
H	-0.792563	3.408059	0.653633
C	3.111619	2.847324	3.375175
H	2.019869	2.917176	3.418851
H	3.407087	1.920412	3.875606
H	3.524908	3.690577	3.938809
C	3.221755	4.190716	1.229211
H	3.648367	5.046682	1.762824
H	3.584151	4.214778	0.196833
H	2.134620	4.320334	1.206894
C	4.416797	-1.522473	-2.151125
H	4.609692	-0.545563	-2.604467
H	5.157706	-2.232624	-2.533356
H	3.430025	-1.858565	-2.488140
C	4.216422	-2.821044	0.014771
H	4.280049	-2.772432	1.105690
H	3.218094	-3.189886	-0.244043
H	4.947127	-3.554924	-0.341609
C	-1.865775	-4.077492	-3.013396
H	-2.906182	-3.977432	-2.690300
H	-1.568142	-3.130924	-3.476922
H	-1.817631	-4.854632	-3.783365
C	0.507009	-4.602064	-2.288586
H	0.894244	-3.677251	-2.730177
H	1.155590	-4.866988	-1.448088
H	0.584914	-5.389516	-3.045577
C	-1.031772	-3.016743	4.050650
H	-0.120493	-3.573280	3.812916
H	-0.761472	-1.960095	4.145239
H	-1.399745	-3.357133	5.024356
C	-3.393364	-2.461251	3.314430

H	-4.163087	-2.626146	2.554134
H	-3.788179	-2.791702	4.281002
H	-3.210386	-1.383435	3.377847
H	-2.339558	-4.287136	2.921706
H	-1.287896	-5.383959	-1.414825
H	5.488005	-1.128869	-0.335755
H	4.699821	2.805248	1.933824
C	-5.002991	2.985800	-0.535425
C	-6.249972	2.402431	-0.805525
C	-4.882636	4.384927	-0.525077
C	-7.358805	3.205481	-1.055983
C	-5.991439	5.182716	-0.778850
C	-7.231224	4.595316	-1.044116
H	-6.360495	1.324634	-0.817484
H	-3.911588	4.819892	-0.326320
H	-8.321085	2.747864	-1.261273
H	-5.891444	6.263259	-0.774106
H	-8.096436	5.220186	-1.243309

C7-TS4S

C	-0.172134	-0.585560	-0.051338
C	0.118965	-1.193915	-1.491195
H	1.068256	-0.797190	-1.846321
C	0.806879	0.551264	0.250955
C	1.172529	1.481010	-0.725352
C	1.327230	0.681510	1.538484
C	2.040556	2.538092	-0.432881
H	0.782752	1.385423	-1.734210
C	2.213646	1.714357	1.856790
H	1.044608	-0.056553	2.279412
C	2.556155	2.636318	0.861624
H	3.241803	3.446521	1.100087
C	-1.612152	-0.090373	0.134792
C	-2.543043	-0.885965	0.803426
C	-2.025788	1.141360	-0.380063
C	-3.877082	-0.484481	0.934631
H	-2.205551	-1.821377	1.231218
C	-3.349378	1.571400	-0.263153
H	-1.304754	1.785353	-0.868679
C	-4.265397	0.743207	0.392065
H	-5.300126	1.065027	0.488252
O	0.047407	-1.662730	0.860068
B	0.750442	-2.717994	0.235193
N	0.316062	-2.666888	-1.270456
C	-0.968387	-1.096754	-2.559769
H	-1.516092	-0.154932	-2.513535
H	-0.493928	-1.171714	-3.539739
C	-1.877575	-2.320477	-2.312028
H	-2.801874	-2.023304	-1.812002
H	-2.143060	-2.805879	-3.253497
C	-1.067040	-3.265109	-1.400646
H	-1.520971	-3.327956	-0.410054
H	-0.959462	-4.272083	-1.800251
B	1.283000	-3.336078	-2.385822
H	1.348579	-4.519828	-2.129586
H	0.798877	-3.135091	-3.477747
H	2.383921	-2.795355	-2.316905
O	2.279183	-2.227250	0.174719
C	3.298121	-2.846088	-0.190340
C	3.421220	-4.335989	-0.142469
H	4.050641	-4.582105	0.723450
H	2.455004	-4.820847	-0.045887
H	3.926057	-4.698040	-1.039025
C	-4.881377	-1.372553	1.649059
C	-3.772097	2.913231	-0.836469
C	2.810951	1.819099	3.249635
C	2.416752	3.546630	-1.504217
H	0.684312	-3.785163	0.774006
C	4.517732	-2.034036	-0.521328
H	5.262674	-2.264230	0.255822
H	4.931655	-2.447766	-1.448145
C	4.272596	-0.532416	-0.632413
H	5.211854	-0.015705	-0.846027
H	3.570165	-0.316539	-1.439721
H	3.848538	-0.123982	0.286077
C	-3.598928	2.950202	-2.364030
H	-2.543702	2.848503	-2.640264
H	-4.149484	2.135209	-2.843325
H	-3.960131	3.898895	-2.775043
C	-3.013961	4.073491	-0.169985
H	-3.154005	4.063330	0.914836
H	-1.938791	4.004094	-0.367022
H	-3.362992	5.037793	-0.554484
C	-4.510537	-1.565906	3.128486
H	-3.548222	-2.078823	3.226915
H	-4.428758	-0.603395	3.641987
H	-5.266318	-2.168533	3.643508
C	-5.035907	-2.726871	0.937212
H	-4.096438	-3.289293	0.957885

H	-5.801855	-3.339133	1.424973
H	-5.320658	-2.590634	-0.110553
C	1.724994	1.965471	4.327219
H	1.085791	2.830007	4.125734
H	1.084099	1.078745	4.363144
H	2.175489	2.093061	5.317228
C	3.726475	0.619105	3.546732
H	4.526394	0.542861	2.803301
H	4.186022	0.712586	4.536505
H	3.158870	-0.317139	3.525335
C	3.200098	2.883529	-2.649402
H	2.587663	2.133807	-3.161634
H	3.509088	3.625904	-3.392810
H	4.095588	2.380493	-2.272487
C	1.179703	4.289123	-2.036988
H	0.631119	4.773386	-1.223505
H	1.467719	5.056662	-2.762970
H	0.492147	3.599777	-2.539102
H	3.074866	4.289178	-1.035300
H	3.430110	2.725172	3.272702
H	-5.852683	-0.862676	1.611063
H	-4.840092	3.040476	-0.617835

C7-TS4R

C	-0.548969	-0.361625	-0.068473
C	0.024798	-0.640450	-1.527589
H	0.453317	0.283476	-1.911705
C	-0.487771	1.138620	0.237944
C	-0.826225	2.097685	-0.720241
C	-0.092481	1.561479	1.506461
C	-0.784408	3.465002	-0.426153
H	-1.126379	1.784337	-1.715655
C	-0.008615	2.919921	1.820805
H	0.185716	0.805558	2.230623
C	-0.365047	3.858849	0.846869
H	-0.310317	4.919256	1.083847
C	-1.974536	-0.884687	0.144476
C	-2.176959	-2.109591	0.781438
C	-3.086051	-0.174491	-0.317321
C	-3.461667	-2.641811	0.936316
H	-1.312832	-2.635407	1.167086
C	-4.381183	-0.678315	-0.176482
H	-2.946126	0.793496	-0.782460
C	-4.552125	-1.917166	0.448134
H	-5.555473	-2.321647	0.563126
O	0.317978	-1.071256	0.814877
B	1.554736	-1.343592	0.176212
N	1.163748	-1.604716	-1.333886
C	-0.888381	-1.306493	-2.555287
H	-1.921863	-0.968270	-2.472871
H	-0.523731	-1.056471	-3.553348
C	-0.740415	-2.821292	-2.297063
H	-1.602650	-3.206815	-1.749035
H	-0.663761	-3.373097	-3.236577
C	0.536149	-2.977165	-1.445008
H	0.290848	-3.331454	-0.442478
H	1.272383	-3.649841	-1.881828
B	2.293005	-1.437489	-2.463914
H	3.110241	-2.320380	-2.315114
H	1.774291	-1.452950	-3.556787
H	2.829323	-0.331537	-2.300426
O	2.303733	0.038675	0.097400
C	3.424656	0.282244	-0.421395
C	3.669994	1.715134	-0.786989
H	4.104028	1.786926	-1.785541
H	2.733399	2.270496	-0.735938
H	4.391994	2.145653	-0.082558
C	-3.661205	-3.984387	1.618630
C	-5.571023	0.113378	-0.691732
C	0.503511	3.365975	3.179444
C	-1.180494	4.491818	-1.472894
H	2.232866	-2.186952	0.692041
C	-5.504962	0.296182	-2.217234
H	-4.629212	0.889538	-2.502076
H	-5.434502	-0.669095	-2.727528
H	-6.394348	0.817544	-2.586852
C	-5.693315	1.471831	0.018741
H	-5.763801	1.343875	1.102809
H	-4.820781	2.101484	-0.185643
H	-6.582782	2.011178	-0.324102
C	-3.206982	-3.942897	3.086820
H	-2.134079	-3.736798	3.158661
H	-3.733932	-3.159132	3.638898
H	-3.398751	-4.901459	3.580831
C	-2.950089	-5.112419	0.852724
H	-1.866093	-4.956548	0.843931
H	-3.143449	-6.083926	1.319928
H	-3.289757	-5.157437	-0.186569
C	-0.335060	2.789497	4.330629

H	-1.389231	3.061113	4.223073
H	-0.273478	1.696857	4.354593
H	0.021011	3.165217	5.295749
C	1.990178	3.002814	3.341620
H	2.591132	3.451019	2.543929
H	2.377952	3.354269	4.303835
H	2.131044	1.917820	3.294831
C	-0.236492	4.453806	-2.686114
H	-0.291350	3.486703	-3.197115
H	-0.501138	5.229960	-3.411962
H	0.802402	4.609954	-2.379841
C	-2.645483	4.314357	-1.906704
H	-3.318387	4.356772	-1.044996
H	-2.941253	5.096571	-2.613763
H	-2.795696	3.347727	-2.399855
H	-1.088260	5.481970	-1.008639
H	0.420187	4.459841	3.218970
H	-4.737460	-4.199821	1.606778
H	-6.473896	-0.467451	-0.463455
C	4.558221	-0.699714	-0.336853
C	5.661394	-0.499415	-1.382372
C	5.132707	-0.613055	1.105276
H	4.134881	-1.697427	-0.462172
C	6.775183	-1.535091	-1.189144
H	6.089075	0.507542	-1.288388
H	5.225598	-0.583220	-2.382077
C	6.256160	-1.641226	1.286848
H	5.525959	0.396270	1.286804
H	4.330980	-0.786121	1.829521
C	7.354797	-1.476511	0.229807
H	7.564703	-1.374197	-1.931164
H	6.366600	-2.536749	-1.376935
H	6.675541	-1.548869	2.294964
H	5.827762	-2.649363	1.213451
H	8.122165	-2.247444	0.360713
H	7.853881	-0.508228	0.376341

C7-TS4S

C	-0.172134	-0.585560	-0.051338
C	0.118965	-1.193915	-1.491195
H	1.068256	-0.797190	-1.846321
C	0.806879	0.551264	0.250955
C	1.172529	1.481010	-0.725352
C	1.327230	0.681510	1.538484
C	2.040556	2.538092	-0.432881
H	0.782752	1.385423	-1.734210
C	2.213646	1.714357	1.856790
H	1.044608	-0.056553	2.279412
C	2.556155	2.636318	0.861624
H	3.241803	3.446521	1.100087
C	-1.612152	-0.090373	0.134792
C	-2.543043	-0.885965	0.803426
C	-2.025788	1.141360	-0.380063
C	-3.877082	-0.484481	0.934631
H	-2.205551	-1.821377	1.231218
C	-3.349378	1.571400	-0.263153
H	-1.304754	1.785353	-0.868679
C	-4.265397	0.743207	0.392065
H	-5.300126	1.065027	0.488252
O	0.047407	-1.662730	0.860068
B	0.750442	-2.717994	0.235193
N	0.316062	-2.666888	-1.270456
C	-0.968387	-1.096754	-2.559769
H	-1.516092	-0.154932	-2.513535
H	-0.493928	-1.171714	-3.539739
C	-1.877575	-2.320477	-2.312028
H	-2.801874	-2.023304	-1.812002
H	-2.143060	-2.805879	-3.253497
C	-1.067040	-3.265109	-1.400646
H	-1.520971	-3.327956	-0.410054
H	-0.959462	-4.272083	-1.800251
B	1.283000	-3.336078	-2.385822
H	1.348579	-4.519828	-2.129586
H	0.798877	-3.135091	-3.477747
H	2.383921	-2.795355	-2.316905
O	2.279183	-2.227250	0.174719
C	3.298121	-2.846088	-0.190340
C	3.421220	-4.335989	-0.142469
H	4.050641	-4.582105	0.723450
H	2.455004	-4.820847	-0.045887
H	3.926057	-4.698040	-1.039025
C	-4.881377	-1.372553	1.649059
C	-3.772097	2.913231	-0.836469
C	2.810951	1.819099	3.249635
C	2.416752	3.546630	-1.504217
H	0.684312	-3.785163	0.774006
C	4.517732	-2.034036	-0.521328
H	5.262674	-2.264230	0.255822
H	4.931655	-2.447766	-1.448145

C	4.272596	-0.532416	-0.632413
H	5.211854	-0.015705	-0.846027
H	3.570165	-0.316539	-1.439721
H	3.848538	-0.123982	0.286077
C	-3.598928	2.950202	-2.364030
H	-2.543702	2.848503	-2.640264
H	-4.149484	2.135209	-2.843325
H	-3.960131	3.898895	-2.775043
C	-3.013961	4.073491	-0.169985
H	-3.154005	4.063330	0.914836
H	-1.938791	4.004094	-0.367022
H	-3.362992	5.037793	-0.554484
C	-4.510537	-1.565906	3.128486
H	-3.548222	-2.078823	3.226915
H	-4.428758	-0.603395	3.641987
H	-5.266318	-2.168533	3.643508
C	-5.035907	-2.726871	0.937212
H	-4.096438	-3.289293	0.957885
H	-5.801855	-3.339133	1.424973
H	-5.320658	-2.590634	-0.110553
C	1.724994	1.965471	4.327219
H	1.085791	2.830007	4.125734
H	1.084099	1.078745	4.363144
H	2.175489	2.093061	5.317228
C	3.726475	0.619105	3.546732
H	4.526394	0.542861	2.803301
H	4.186022	0.712586	4.536505
H	3.158870	-0.317139	3.525335
C	3.200098	2.883529	-2.649402
H	2.587663	2.133807	-3.161634
H	3.509088	3.625904	-3.392810
H	4.095588	2.380493	-2.272487
C	1.179703	4.289123	-2.036988
H	0.631119	4.773386	-1.223505
H	1.467719	5.056662	-2.762970
H	0.492147	3.599777	-2.539102
H	3.074866	4.289178	-1.035300
H	3.430110	2.725172	3.272702
H	-5.852683	-0.862676	1.611063
H	-4.840092	3.040476	-0.617835

C7-TS7Rpri

C	-0.088231	-0.572282	-0.321857
C	0.212608	-1.428433	-1.606676
H	0.805653	-0.842781	-2.300664
C	0.759300	0.706970	-0.212796
C	0.443938	1.591856	0.826712
C	1.884670	0.970099	-0.994342
C	1.216290	2.724278	1.082145
H	-0.424102	1.380963	1.441358
C	2.695329	2.086791	-0.747679
H	2.185188	0.282657	-1.773209
C	2.344198	2.955934	0.285351
H	2.961549	3.830559	0.478948
C	-1.569756	-0.213776	-0.226338
C	-2.079884	0.874308	-0.939483
C	-2.431616	-0.995521	0.539928
C	-3.438749	1.193265	-0.891173
C	-3.801207	-0.710681	0.594224
H	-2.014461	-1.823498	1.100535
C	-4.285790	0.385903	-0.123530
O	0.267989	-1.391573	0.790969
B	0.737009	-2.690264	0.436662
N	1.054263	-2.604983	-1.138982
C	-0.979463	-2.041945	-2.382140
H	-1.922597	-1.538192	-2.179528
H	-0.778356	-1.948081	-3.454363
C	-0.987078	-3.522972	-1.990222
H	-1.505661	-3.664631	-1.037175
H	-1.463870	-4.161065	-2.738860
C	0.498672	-3.815406	-1.835406
H	0.737811	-4.701151	-1.246744
H	0.983184	-3.903435	-2.812322
B	2.617017	-2.466554	-1.506292
H	3.121754	-3.562971	-1.454347
H	2.717118	-1.932445	-2.590571
H	3.165108	-1.741942	-0.657079
O	2.076846	-3.062686	1.098074
C	3.108254	-2.332836	1.211584
C	4.417765	-3.056463	1.262016
H	4.693645	-3.181544	2.317863
H	4.333680	-4.031955	0.786430
H	5.198701	-2.463037	0.783884
C	3.955974	2.308975	-1.564926
C	0.836118	3.681738	2.198353
H	0.016072	-3.614060	0.722218
C	3.058209	-0.916520	1.716490
H	4.074259	-0.513233	1.715414
H	2.447666	-0.302642	1.062510

C	2.445325	-0.876739	3.129387
H	2.976471	-1.525508	3.833508
H	2.490717	0.147291	3.507649
H	1.398278	-1.178896	3.081544
C	4.986195	1.200178	-1.283850
H	5.229413	1.156231	-0.217280
H	4.596574	0.220678	-1.579279
H	5.912917	1.379524	-1.839392
C	3.658030	2.414248	-3.068947
H	2.930202	3.205410	-3.272652
H	4.572504	2.635293	-3.629121
H	3.251549	1.474678	-3.456901
C	0.928952	3.002760	3.574798
H	0.684953	3.708392	4.375964
H	0.232152	2.161126	3.644562
H	1.937658	2.617516	3.754130
C	-0.562089	4.279873	1.968314
H	-0.618162	4.785745	0.999883
H	-1.330391	3.499957	1.978560
H	-0.808803	5.005174	2.750846
H	4.390983	3.263434	-1.241446
H	1.560015	4.506641	2.183035
H	-5.346436	0.623651	-0.078367
H	-1.400530	1.489396	-1.521204
C	-3.974278	2.407729	-1.629447
H	-5.058915	2.438491	-1.464516
C	-3.733765	2.306555	-3.144221
C	-3.375471	3.705288	-1.059293
H	-3.576090	3.792465	0.012692
H	-2.289030	3.727035	-1.194943
H	-3.795579	4.583238	-1.561541
H	-2.662872	2.291182	-3.372463
H	-4.172701	3.163699	-3.665611
H	-4.175168	1.392559	-3.552663
C	-4.735909	-1.573203	1.424437
H	-5.751253	-1.178784	1.288820
C	-4.395043	-1.487742	2.921422
C	-4.730985	-3.032643	0.940302
H	-4.993124	-3.096251	-0.120095
H	-3.741694	-3.484337	1.068041
H	-5.448489	-3.633634	1.508955
H	-3.389415	-1.874839	3.115597
H	-5.102109	-2.076216	3.515795
H	-4.427693	-0.452331	3.273458

C7-TS7S

C	-0.085632	-0.504538	-0.123004
C	0.240724	-0.977484	-1.607522
H	1.054945	-0.366143	-1.992699
C	0.679424	0.782558	0.199733
C	0.807578	1.813101	-0.735104
C	1.241893	0.944015	1.465562
C	1.475514	3.001220	-0.419318
H	0.381800	1.698252	-1.727360
C	1.940092	2.106491	1.801727
H	1.147796	0.128320	2.172157
C	2.042085	3.127971	0.851285
H	2.576733	4.040644	1.105787
C	-1.581819	-0.302039	0.146894
C	-2.315081	-1.300650	0.788734
C	-2.242708	0.858094	-0.266135
C	-3.692844	-1.168666	0.994449
H	-1.788336	-2.179613	1.137811
C	-3.616461	1.020613	-0.073510
H	-1.679128	1.656294	-0.733448
C	-4.329268	-0.005450	0.553653
H	-5.400140	0.107604	0.708461
O	0.375425	-1.563555	0.716490
B	1.290816	-2.385343	0.015112
N	0.770000	-2.379306	-1.473563
C	-0.909132	-1.081400	-2.608153
H	-1.647064	-0.289615	-2.475556
H	-0.497463	-1.002083	-3.615838
C	-1.506249	-2.488247	-2.384999
H	-2.443287	-2.429929	-1.827173
H	-1.710801	-2.980574	-3.338189
C	-0.454443	-3.263650	-1.565499
H	-0.820673	-3.461193	-0.556751
H	-0.155544	-4.206829	-2.019510
B	1.792589	-2.763165	-2.657683
H	2.066635	-3.938403	-2.540753
H	1.279756	-2.505697	-3.722823
H	2.809872	-2.071786	-2.526677
O	2.624375	-1.534059	-0.091217
C	3.711471	-1.874077	-0.615306
C	4.647662	-0.766456	-0.986881
H	5.032418	-0.929180	-1.996129
H	4.138285	0.194920	-0.920209
H	5.504477	-0.780090	-0.301782

C	-4.478228	-2.274185	1.679111
C	-4.309218	2.291394	-0.535204
C	2.602398	2.242752	3.161897
C	1.578835	4.122890	-1.438224
H	1.505664	-3.463474	0.492389
C	4.214332	-3.288263	-0.581421
H	3.408372	-3.969521	-0.847121
H	5.012130	-3.402020	-1.319159
C	4.733536	-3.597128	0.839247
H	5.126398	-4.616263	0.868149
H	5.537175	-2.917893	1.140263
H	3.923683	-3.519187	1.568407
C	-4.210983	2.462332	-2.060285
H	-3.168225	2.582779	-2.373761
H	-4.617937	1.590971	-2.582036
H	-4.761669	3.349775	-2.389820
C	-3.756416	3.528507	0.192295
H	-3.846745	3.419995	1.277013
H	-2.696609	3.678726	-0.039621
H	-4.296742	4.431678	-0.110868
C	-4.005095	-2.489100	3.126002
H	-2.960896	-2.817411	3.151760
H	-4.077139	-1.563310	3.704355
H	-4.610058	-3.254694	3.623505
C	-4.413982	-3.584679	0.877061
H	-3.386290	-3.958938	0.820790
H	-5.026332	-4.361129	1.347838
H	-4.772643	-3.437825	-0.146313
C	1.589338	2.111372	4.309841
H	0.785829	2.847001	4.210699
H	1.131271	1.117197	4.318841
H	2.078157	2.263052	5.278077
C	3.746626	1.225525	3.314883
H	4.486893	1.346562	2.517640
H	4.254340	1.347969	4.277645
H	3.364157	0.200761	3.261969
C	2.375582	3.688112	-2.679398
H	1.871246	2.869860	-3.204273
H	2.484852	4.518992	-3.384344
H	3.375120	3.339693	-2.401927
C	0.189053	4.652223	-1.831182
H	-0.371561	4.976821	-0.949431
H	0.273906	5.501422	-2.517415
H	-0.399604	3.877126	-2.334016
H	2.125995	4.946118	-0.961423
H	3.039828	3.248185	3.214326
H	-5.527948	-1.955229	1.713845
H	-5.371799	2.196944	-0.277069

C8-B5-TS1R

C	-0.529893	-0.754378	-0.011268
C	-0.138013	-1.399726	-1.412185
H	0.821232	-0.989518	-1.724141
C	0.425036	0.400755	0.303935
C	0.920369	0.549713	1.597213
C	0.826463	1.303668	-0.682611
C	1.831750	1.563085	1.913047
H	0.616253	-0.179052	2.337346
C	1.709222	2.352213	-0.401413
H	0.458554	1.183447	-1.695771
C	2.205427	2.460611	0.903104
H	2.902387	3.252521	1.135734
C	-1.983193	-0.266851	0.063395
C	-2.371107	0.950461	-0.499021
C	-2.950786	-1.059687	0.677901
C	-3.702708	1.373778	-0.484605
H	-1.620439	1.589443	-0.944993
C	-4.296411	-0.674861	0.710371
H	-2.627339	-1.981062	1.143280
C	-4.653827	0.542643	0.119582
H	-5.688321	0.853132	0.136945
O	-0.365420	-1.799714	0.941995
B	0.436441	-2.850165	0.414222
N	0.079344	-2.860433	-1.134054
C	-1.163734	-1.360889	-2.543696
H	-1.735048	-0.432145	-2.555747
H	-0.633357	-1.449843	-3.493736
C	-2.057065	-2.599538	-2.312356
H	-3.015365	-2.311539	-1.875201
H	-2.257196	-3.117944	-3.252746
C	-1.279252	-3.496643	-1.327408
H	-1.783393	-3.538032	-0.360878
H	-1.130403	-4.513639	-1.686348
B	1.135837	-3.512293	-2.135204
H	1.143355	-4.710093	-1.973168
H	0.912276	-3.166960	-3.272091
H	2.259234	-3.050123	-1.833585
O	1.902886	-2.337334	0.441210
C	2.924487	-2.899316	-0.071131

C	4.052742	-1.998472	-0.389612
C	5.270320	-2.485436	-0.890352
C	3.903305	-0.620933	-0.155068
C	6.319560	-1.609206	-1.150191
H	5.398434	-3.543256	-1.087306
C	4.959592	0.247897	-0.404389
C	6.167726	-0.242334	-0.905161
H	4.831795	1.306214	-0.203552
H	6.990323	0.437685	-1.104539
C	3.146973	-4.377320	0.087237
H	3.731478	-4.516163	1.006105
H	2.199577	-4.899585	0.182196
H	3.703493	-4.798020	-0.748196
H	2.966708	-0.248626	0.233533
H	7.256064	-1.991466	-1.543053
C	2.098806	3.323055	-1.524932
C	2.424191	1.619982	3.327626
C	-4.058682	2.725128	-1.120894
C	-5.319842	-1.599300	1.385298
C	2.733913	2.535825	-2.691885
H	2.041760	1.800055	-3.109853
H	3.022145	3.218027	-3.498940
H	3.626866	1.997565	-2.360854
C	0.828526	4.045672	-2.025452
H	1.076975	4.748946	-2.827778
H	0.092014	3.338588	-2.418025
H	0.354429	4.605745	-1.213234
C	3.105482	4.385938	-1.056889
H	3.350598	5.053388	-1.888596
H	2.700740	4.999561	-0.246254
H	4.040092	3.933871	-0.709035
C	3.219316	0.318878	3.578270
H	2.582447	-0.564393	3.481547
H	4.035981	0.219653	2.856006
H	3.649285	0.320707	4.585989
C	3.375095	2.811952	3.520015
H	2.866093	3.767016	3.355503
H	3.761988	2.812928	4.543616
H	4.233690	2.759542	2.842974
C	1.284940	1.736366	4.361974
H	1.694355	1.764896	5.377615
H	0.706183	2.651224	4.200353
H	0.596180	0.890001	4.301769
C	-5.559732	3.040645	-1.025029
H	-5.895515	3.094892	0.015199
H	-5.764059	4.009535	-1.491075
H	-6.165062	2.289027	-1.541492
C	-3.662338	2.706273	-2.613293
H	-2.589483	2.537414	-2.743181
H	-4.193779	1.911732	-3.146696
H	-3.909536	3.662338	-3.087631
C	-3.278448	3.846773	-0.400694
H	-3.542023	3.881550	0.660921
H	-2.197643	3.696440	-0.470966
H	-3.512630	4.820667	-0.844757
C	-6.750984	-1.043153	1.314023
H	-7.084913	-0.915222	0.279449
H	-7.441203	-1.737991	1.802385
H	-6.836194	-0.078156	1.823373
C	-5.300570	-2.974461	0.682797
H	-5.560264	-2.872874	-0.375939
H	-4.314896	-3.443738	0.743308
H	-6.023874	-3.652934	1.148348
C	-4.941509	-1.778053	2.871318
H	-3.942566	-2.207583	2.983493
H	-4.951710	-0.815137	3.391537
H	-5.654017	-2.444674	3.369726
H	0.319834	-3.910818	0.963351

C8-B5-TS1Rpri

C	0.145049	0.755697	-0.098171
C	-0.248918	1.537735	-1.391011
H	-1.329258	1.445858	-1.510491
C	-0.714596	-0.481335	0.137471
C	-0.769254	-1.006526	1.429784
C	-1.361076	-1.150880	-0.897072
C	-1.473721	-2.179353	1.707396
H	-0.246422	-0.473593	2.215504
C	-2.070055	-2.337022	-0.664009
H	-1.313073	-0.752382	-1.903260
C	-2.117979	-2.830589	0.643504
H	-2.660317	-3.743787	0.839852
C	1.611772	0.296398	-0.057272
C	2.021448	-0.821131	-0.787821
C	2.547059	0.971751	0.722604
C	3.353378	-1.239532	-0.797807
H	1.280882	-1.387608	-1.339304
C	3.893617	0.586785	0.738521
H	2.205288	1.804039	1.323924
C	4.278553	-0.513694	-0.035647

H	5.313959	-0.821609	-0.034254
O	-0.092248	1.700227	0.946392
B	-0.077758	3.053159	0.486831
N	0.037313	2.980037	-1.081786
C	0.504005	1.257432	-2.690609
H	0.765711	0.205267	-2.807269
H	-0.135169	1.544529	-3.527051
C	1.751681	2.166905	-2.632387
H	2.640304	1.588239	-2.370408
H	1.929944	2.643026	-3.598602
C	1.463567	3.210842	-1.534118
H	2.140021	3.082768	-0.690531
H	1.536365	4.239767	-1.883076
B	-0.912417	3.966445	-1.960366
H	-0.721766	5.098240	-1.565954
H	-0.612894	3.825751	-3.126086
H	-2.069944	3.617568	-1.781930
O	-1.445340	3.827077	0.841362
C	-2.641622	3.816392	0.455649
C	-3.440155	2.594581	0.303364
C	-4.573912	2.586943	-0.532558
C	-3.116903	1.447872	1.045419
C	-5.355441	1.445378	-0.635155
H	-4.808297	3.459297	-1.131587
C	-3.920053	0.314154	0.954975
C	-5.034173	0.310887	0.118886
H	-3.653661	-0.573646	1.514442
H	-5.653562	-0.577926	0.048352
C	-3.276476	5.169868	0.312330
H	-2.920994	5.798242	1.132209
H	-2.901842	5.606986	-0.618178
H	-4.364725	5.138665	0.306493
H	-2.237127	1.454364	1.674165
H	-6.213582	1.434785	-1.299046
C	-2.735133	-3.052652	-1.847655
C	-1.500424	-2.717996	3.145059
C	3.738113	-2.474908	-1.624012
C	4.884896	1.380729	1.601010
C	3.383478	-2.227180	-3.106302
H	2.312767	-2.049082	-3.240229
H	3.919210	-1.354671	-3.493328
H	3.656450	-3.095431	-3.716061
C	5.238256	-2.796219	-1.534015
H	5.544909	-3.010211	-0.505444
H	5.463707	-3.680149	-2.138583
H	5.851710	-1.971201	-1.909595
C	2.946309	-3.695943	-1.106296
H	3.198498	-4.589582	-1.687887
H	3.181846	-3.892700	-0.055733
H	1.866533	-3.538885	-1.181258
C	4.874165	2.859507	1.156078
H	3.884489	3.309346	1.271804
H	5.579705	3.444375	1.756314
H	5.164193	2.949976	0.104288
C	6.322829	0.851471	1.480811
H	6.690562	0.908975	0.451408
H	6.990101	1.452714	2.105983
H	6.401945	-0.187659	1.815502
C	4.454726	1.288639	3.081009
H	3.448968	1.689362	3.232779
H	4.456310	0.248013	3.420633
H	5.143574	1.857336	3.715356
C	-3.725303	-2.094680	-2.543916
H	-3.230375	-1.192142	-2.911701
H	-4.197288	-2.590504	-3.398959
H	-4.511725	-1.779797	-1.852066
C	-1.639471	-3.475809	-2.851479
H	-1.088283	-2.611489	-3.232734
H	-0.919090	-4.149956	-2.377130
H	-2.082909	-3.995234	-3.707966
C	-3.505991	-4.309704	-1.414626
H	-4.302996	-4.069615	-0.703562
H	-3.969535	-4.777534	-2.288468
H	-2.846966	-5.051128	-0.952521
C	-2.103786	-1.649430	4.082083
H	-1.533984	-0.717059	4.052147
H	-3.137131	-1.420741	3.800509
H	-2.107502	-2.007693	5.117154
C	-2.336239	-4.001054	3.276936
H	-3.379167	-3.837068	2.987080
H	-1.933545	-4.812578	2.662936
H	-2.330279	-4.339101	4.317631
C	-0.054658	-3.030527	3.589010
H	0.396229	-3.783530	2.935177
H	0.577732	-2.139514	3.556058
H	-0.044305	-3.415449	4.614586
H	0.720800	3.783254	1.006357

C8-B5-TS1S

C	-0.281373	-0.522797	-0.078788

C	0.261907	-0.970403	-1.504902
H	0.895915	-0.177542	-1.895881
C	0.131868	0.926488	0.198069
C	0.599516	1.277848	1.461778
C	0.060373	1.907976	-0.792231
C	1.022886	2.579627	1.744362
H	0.667790	0.495070	2.205977
C	0.438934	3.232196	-0.540396
H	-0.290699	1.640168	-1.783159
C	0.926840	3.545224	0.733389
H	1.241834	4.557416	0.940786
C	-1.796250	-0.687874	0.082074
C	-2.687505	0.267274	-0.408890
C	-2.307509	-1.832156	0.692215
C	-4.071212	0.090894	-0.322551
H	-2.297881	1.172383	-0.855515
C	-3.686475	-2.048847	0.796217
H	-1.603454	-2.547080	1.096737
C	-4.552403	-1.077996	0.279408
H	-5.619230	-1.231069	0.352546
O	0.360505	-1.393897	0.852778
B	1.510092	-1.981535	0.273606
N	1.154706	-2.149519	-1.243107
C	-0.736909	-1.450381	-2.556640
H	-1.675873	-0.896124	-2.520849
H	-0.290181	-1.308125	-3.542600
C	-0.927189	-2.954657	-2.270835
H	-1.864667	-3.130625	-1.739167
H	-0.952886	-3.530457	-3.198561
C	0.265221	-3.367072	-1.383767
H	-0.077150	-3.672423	-0.393190
H	0.866406	-4.170589	-1.806290
B	2.355055	-2.244716	-2.316671
H	3.054786	-3.180287	-2.000514
H	1.867923	-2.363644	-3.421173
H	2.978300	-1.184097	-2.266273
O	2.606566	-0.803350	0.268331
C	3.708712	-0.554194	-0.280962
C	3.908930	0.873624	-0.711498
H	4.624489	1.355889	-0.035706
H	4.316597	0.925641	-1.722334
H	2.955232	1.398652	-0.669069
C	-4.994408	1.182116	-0.883495
C	-4.190508	-3.336034	1.464699
C	0.316437	4.271269	-1.663744
C	1.615769	2.887012	3.126442
H	1.926892	-2.955629	0.827087
C	-3.638312	-4.556009	0.695502
H	-3.977368	-4.544286	-0.345435
H	-2.545002	-4.568859	0.692216
H	-3.983137	-5.488139	1.156498
C	-5.724606	-3.430060	1.477369
H	-6.176170	-2.604858	2.036871
H	-6.139172	-3.424513	0.464321
H	-6.032778	-4.364057	1.957399
C	-3.689062	-3.381286	2.924106
H	-4.070639	-2.526223	3.490840
H	-4.029197	-4.298792	3.417055
H	-2.597419	-3.355446	2.975519
C	-4.712581	1.361707	-2.391188
H	-4.894863	0.429031	-2.934433
H	-5.362008	2.136710	-2.812909
H	-3.676074	1.659007	-2.574242
C	-6.481999	0.838053	-0.708587
H	-7.098328	1.642601	-1.121584
H	-6.747888	-0.086093	-1.231392
H	-6.748644	0.723334	0.346666
C	-4.713212	2.512351	-0.150838
H	-4.904740	2.411704	0.921973
H	-3.674485	2.830442	-0.275506
H	-5.358110	3.307209	-0.541743
C	-1.153821	4.342140	-2.132237
H	-1.811436	4.626353	-1.304661
H	-1.266965	5.084199	-2.930026
H	-1.502153	3.380985	-2.520830
C	0.744396	5.676373	-1.211085
H	0.630471	6.381757	-2.039925
H	0.131144	6.037048	-0.379513
H	1.792991	5.699215	-0.898219
C	1.212753	3.849239	-2.848227
H	2.261732	3.796105	-2.539070
H	0.930247	2.868521	-3.240695
H	1.134374	4.573289	-3.666540
C	1.974746	4.371648	3.297441
H	2.383001	4.539501	4.298863
H	2.730872	4.693369	2.574306
H	1.096570	5.015059	3.183473
C	2.903272	2.051172	3.302998
H	2.702087	0.980981	3.206061
H	3.644328	2.322213	2.543241

H	3.345989	2.228164	4.289481
C	0.600659	2.505704	4.224495
H	0.339805	1.445268	4.184658
H	1.016976	2.712329	5.216501
H	-0.323307	3.081830	4.114148
C	4.834328	-1.498224	-0.278202
C	4.810716	-2.606435	0.583846
C	5.963338	-1.275505	-1.083924
C	5.893892	-3.475969	0.633003
C	7.037730	-2.155406	-1.041839
C	7.005570	-3.256112	-0.182232
H	3.944542	-2.771074	1.210202
H	5.988728	-0.433877	-1.765535
H	5.870241	-4.327960	1.304380
H	7.898789	-1.987382	-1.680222
H	7.846867	-3.941419	-0.148967

C8-B5-TS1Spri

C	0.032539	-0.289559	-0.115383
C	0.706075	-0.577384	-1.500160
H	1.320769	0.289177	-1.746734
C	-0.207086	1.203267	0.096227
C	-0.127926	1.716662	1.392261
C	-0.564293	2.045298	-0.958786
C	-0.401029	3.062234	1.650068
H	0.160623	1.038544	2.182867
C	-0.867880	3.393125	-0.736521
H	-0.614937	1.644688	-1.962088
C	-0.775083	3.876047	0.572613
H	-1.002008	4.920092	0.760782
C	-1.290922	-1.044142	0.082959
C	-2.476102	-0.576095	-0.487456
C	-1.316333	-2.233383	0.808784
C	-3.668849	-1.296318	-0.390031
H	-2.470540	0.374679	-1.005321
C	-2.490064	-2.987635	0.926976
H	-0.402121	-2.564959	1.281676
C	-3.653079	-2.507836	0.313179
H	-4.565500	-3.080472	0.393664
O	0.994150	-0.735076	0.830993
B	1.884844	-1.737734	0.319963
N	1.642818	-1.733543	-1.266437
C	-0.160114	-0.995409	-2.687329
H	-1.124042	-0.485346	-2.710162
H	0.377733	-0.752057	-3.606485
C	-0.314283	-2.518639	-2.543154
H	-1.226247	-2.757050	-1.992716
H	-0.372077	-3.009660	-3.517659
C	0.920626	-2.980912	-1.747448
H	0.638802	-3.584835	-0.886072
H	1.620815	-3.554240	-2.354429
B	2.968359	-1.544903	-2.145967
H	3.690807	-2.500958	-1.997963
H	2.664395	-1.298580	-3.290711
H	3.565013	-0.543290	-1.685691
O	3.365882	-1.388029	0.569218
C	4.018672	-0.388798	0.113742
C	5.472295	-0.596960	-0.050482
C	6.354294	0.478750	-0.232603
C	5.978282	-1.906421	-0.012816
C	7.720041	0.248459	-0.369754
H	5.980660	1.495335	-0.264206
C	7.341988	-2.131952	-0.153386
C	8.215373	-1.055870	-0.331491
H	7.726769	-3.146298	-0.128186
H	9.280611	-1.233969	-0.442311
C	3.463732	1.007911	0.191861
H	2.379923	0.998825	0.222751
H	3.816015	1.439945	1.137708
H	3.821092	1.632313	-0.626231
H	5.283888	-2.726563	0.116870
H	8.397163	1.085175	-0.507433
C	-0.311111	3.664857	3.059057
C	0.731592	4.803387	3.060182
H	0.810076	5.249068	4.057800
H	1.718613	4.424407	2.776459
H	0.464493	5.598075	2.358027
C	-1.690298	4.229752	3.461723
H	-1.645977	4.665630	4.465771
H	-2.025588	5.009954	2.772677
H	-2.446246	3.438482	3.464665
C	0.109430	2.624578	4.109682
H	-0.607972	1.800991	4.172910
H	1.094108	2.201483	3.888541
H	0.162800	3.097309	5.095286
C	-1.307316	4.333403	-1.867659
C	-0.323954	5.519164	-1.964403
C	-1.345987	3.626804	-3.232677
C	-2.724956	4.862311	-1.559272
H	-0.283710	6.087969	-1.031458

H	0.688286	5.165894	-2.184834
H	-0.628590	6.204862	-2.762494
H	-2.055490	2.792985	-3.237336
H	-1.662755	4.334214	-4.004999
H	-0.362456	3.241186	-3.518809
H	-3.063482	5.536477	-2.353629
H	-3.438597	4.035404	-1.484604
H	-2.753915	5.413471	-0.615420
C	-4.934692	-0.728725	-1.047899
C	-5.254236	0.649513	-0.428433
C	-4.689492	-0.562344	-2.563500
C	-6.155628	-1.641913	-0.854632
H	-5.434663	0.558306	0.647109
H	-4.431131	1.356168	-0.568166
H	-6.149753	1.078481	-0.891536
H	-4.443214	-1.523782	-3.025152
H	-5.583972	-0.164779	-3.055469
H	-3.864242	0.126868	-2.764812
H	-7.030283	-1.197010	-1.339108
H	-5.996257	-2.629705	-1.298415
H	-6.395310	-1.777177	0.204602
C	-2.452954	-4.303322	1.717263
C	-1.399123	-5.242407	1.090401
C	-2.063751	-4.004108	3.181008
C	-3.807886	-5.028819	1.713939
H	-1.643020	-5.455563	0.044488
H	-0.398479	-4.803062	1.121029
H	-1.363483	-6.193376	1.633145
H	-2.798602	-3.341837	3.649508
H	-2.018903	-4.932014	3.761870
H	-1.086377	-3.518659	3.244943
H	-3.727992	-5.961300	2.281149
H	-4.592733	-4.424252	2.179262
H	-4.128230	-5.283687	0.698675
H	1.786364	-2.838588	0.799877

C8-TS4S

C	0.263240	0.872300	-0.353641
C	0.113796	1.432540	-1.831989
H	-0.703016	0.922824	-2.337048
C	-0.845434	-0.141000	-0.026141
C	-1.393666	-0.142433	1.256881
C	-1.289328	-1.080660	-0.958405
C	-2.404272	-1.038270	1.610752
H	-1.032126	0.600222	1.953077
C	-2.282887	-2.013280	-0.635759
H	-0.862496	-1.086541	-1.952556
C	-2.832684	-1.964008	0.648701
H	-3.616190	-2.667393	0.910004
C	1.613759	0.210485	-0.058924
C	1.923081	-1.047479	-0.593152
C	2.546400	0.851297	0.746987
C	3.150235	-1.656805	-0.347135
H	1.184003	-1.555036	-1.195356
C	3.796787	0.270767	1.017252
H	2.278031	1.811984	1.166132
C	4.077884	-0.975686	0.460048
H	5.035030	-1.441841	0.656059
O	0.134993	2.019105	0.483357
B	-0.647029	3.007330	-0.155805
N	-0.296354	2.885551	-1.684155
C	1.374063	1.468877	-2.694692
H	1.974271	0.562780	-2.606176
H	1.074198	1.588504	-3.739947
C	2.097426	2.725826	-2.203130
H	2.636966	2.509170	-1.278192
H	2.815728	3.113703	-2.930405
C	0.956823	3.715222	-1.952291
H	1.142911	4.376229	-1.105479
H	0.756029	4.326824	-2.831144
B	-1.404749	3.296711	-2.787516
H	-1.587427	4.492004	-2.696095
H	-0.968648	2.988618	-3.877097
H	-2.431057	2.662614	-2.571450
O	-2.161233	2.452280	-0.077233
C	-3.308032	2.576549	-0.551601
C	-4.012656	1.284930	-0.865963
H	-4.961824	1.424584	-1.379216
H	-3.348325	0.651996	-1.452935
H	-4.182305	0.758083	0.079697
C	3.507287	-3.035566	-0.919364
C	4.792892	1.028229	1.905919
C	-2.783186	-3.060863	-1.640500
C	-3.060998	-1.027249	2.998622
H	-0.589939	4.099589	0.318654
C	-4.089876	3.879938	-0.511861
C	-3.212952	5.072181	-0.114869
H	-3.839940	5.966109	-0.044629
H	-2.732835	4.912232	0.852593
H	-2.437495	5.249414	-0.860285

C	4.750386	-2.905210	-1.825269
H	5.026306	-3.880582	-2.240928
H	4.553167	-2.221738	-2.657212
H	5.612163	-2.519599	-1.273546
C	2.362065	-3.634444	-1.753782
H	1.453093	-3.764985	-1.157623
H	2.118308	-3.005912	-2.616407
H	2.656810	-4.617660	-2.133087
C	3.815362	-4.006593	0.240676
H	4.075486	-4.997059	-0.148618
H	4.652821	-3.655773	0.849805
H	2.945958	-4.113190	0.896828
C	6.114385	0.265173	2.088821
H	6.790392	0.845324	2.724490
H	5.958107	-0.705540	2.569849
H	6.620149	0.096733	1.132761
C	5.108594	2.395461	1.261281
H	5.551252	2.263020	0.268857
H	4.208050	3.005000	1.148280
H	5.817749	2.955817	1.880420
C	4.163654	1.250969	3.298028
H	3.934282	0.293617	3.776481
H	4.854637	1.801334	3.946101
H	3.234572	1.823266	3.232841
C	-4.289984	-2.842776	-1.896442
H	-4.671249	-3.588480	-2.602458
H	-4.871980	-2.925151	-0.974331
H	-4.471215	-1.849458	-2.319762
C	-2.557787	-4.472447	-1.057265
H	-1.494054	-4.646022	-0.866088
H	-3.093152	-4.611479	-0.114149
H	-2.908901	-5.236654	-1.759115
C	-2.048009	-2.977397	-2.988296
H	-2.193838	-2.006624	-3.472379
H	-0.972447	-3.146122	-2.873493
H	-2.433212	-3.745473	-3.665727
C	-2.449772	0.039080	3.921634
H	-1.381782	-0.132569	4.084878
H	-2.572631	1.046407	3.511907
H	-2.946343	0.011628	4.896526
C	-2.879389	-2.408157	3.664091
H	-3.340028	-2.417067	4.657899
H	-3.339372	-3.206332	3.074907
H	-1.817102	-2.645258	3.776709
C	-4.567712	-0.725469	2.845885
H	-5.066294	-1.459450	2.206655
H	-5.062974	-0.738347	3.822882
H	-4.721574	0.265422	2.404453
C	-4.794190	4.168020	-1.851616
H	-4.058278	4.346797	-2.635882
H	-5.446408	3.349100	-2.165401
H	-5.415444	5.061324	-1.735548
C	-5.159537	3.648159	0.595408
H	-5.863706	2.854507	0.333698
H	-4.692812	3.400255	1.554016
H	-5.726952	4.573943	0.725070

C8-TS4R

C	-0.295630	-0.731490	-0.084380
C	0.053611	-1.371088	-1.497991
H	0.981669	-0.928953	-1.855329
C	0.614374	0.469226	0.186569
C	1.127180	0.663473	1.462831
C	0.927878	1.387167	-0.823648
C	1.963536	1.751297	1.753127
H	0.882943	-0.070244	2.220395
C	1.740611	2.492066	-0.571444
H	0.539927	1.226408	-1.821292
C	2.252011	2.652106	0.726360
H	2.894260	3.499488	0.928959
C	-1.764151	-0.310709	0.062834
C	-2.233807	0.881808	-0.490171
C	-2.662050	-1.135335	0.737980
C	-3.578855	1.249841	-0.405925
H	-1.535646	1.544805	-0.983922
C	-4.019165	-0.806637	0.840540
H	-2.275748	-2.036490	1.194956
C	-4.459633	0.387543	0.258409
H	-5.503760	0.655023	0.330150
O	-0.034329	-1.761760	0.866827
B	0.757553	-2.789495	0.297573
N	0.335673	-2.822533	-1.223874
C	-1.024783	-1.373603	-2.580123
H	-1.630135	-0.466835	-2.565453
H	-0.537151	-1.445403	-3.553776
C	-1.859650	-2.644010	-2.305398
H	-2.811872	-2.390279	-1.834769
H	-2.072847	-3.178427	-3.233665
C	-1.010897	-3.501544	-1.344443
H	-1.471645	-3.543736	-0.356302

H	-0.845966	-4.518421	-1.696164
B	1.349942	-3.461285	-2.302750
H	1.401978	-4.655228	-2.097796
H	0.961305	-3.196747	-3.417362
H	2.463912	-2.953233	-2.132840
O	2.231895	-2.217859	0.234613
C	3.269697	-2.810447	-0.150743
C	4.467170	-1.931658	-0.479060
C	-4.024719	2.577650	-1.034976
C	-4.964035	-1.764902	1.579830
C	2.099513	3.513820	-1.658873
C	2.540450	1.893991	3.168137
H	0.732109	-3.841920	0.869851
C	-3.716148	2.553650	-2.547903
H	-4.028272	3.492724	-3.017954
H	-2.646943	2.423863	-2.739039
H	-4.246268	1.731863	-3.039650
C	-3.248504	3.738315	-0.374565
H	-3.451482	3.778033	0.700089
H	-2.168079	3.627810	-0.504348
H	-3.545246	4.696327	-0.815699
C	-5.528751	2.836184	-0.853555
H	-5.804688	2.889113	0.204187
H	-5.798031	3.791036	-1.315576
H	-6.133315	2.055883	-1.326504
C	-6.418918	-1.269140	1.583932
H	-7.051349	-1.986578	2.115934
H	-6.515977	-0.303044	2.089071
H	-6.814719	-1.166254	0.568583
C	-4.926195	-3.145758	0.889323
H	-5.248525	-3.066693	-0.153984
H	-3.919729	-3.572839	0.898188
H	-5.593125	-3.848594	1.400687
C	-4.496910	-1.911602	3.043951
H	-4.518163	-0.944241	3.555526
H	-5.152455	-2.601280	3.587157
H	-3.476351	-2.298724	3.103994
C	3.630424	3.526540	-1.859031
H	3.910953	4.254460	-2.628166
H	4.155386	3.792547	-0.937471
H	3.986163	2.540516	-2.174496
C	1.623404	4.914876	-1.218701
H	0.538687	4.925721	-1.071022
H	2.090827	5.225084	-0.280280
H	1.872813	5.660848	-1.981174
C	1.440796	3.186398	-3.008883
H	1.762743	2.213336	-3.392799
H	0.348368	3.181787	-2.935715
H	1.719736	3.943023	-3.748488
C	1.385276	1.957883	4.189840
H	0.740314	2.819340	3.990694
H	0.763398	1.059925	4.153784
H	1.780590	2.053412	5.207044
C	3.397851	3.159270	3.329336
H	3.778987	3.218912	4.353457
H	4.260090	3.154039	2.654960
H	2.817626	4.067151	3.136787
C	3.427136	0.666025	3.470423
H	4.266859	0.618076	2.769278
H	3.833713	0.724844	4.486076
H	2.862238	-0.266348	3.384558
C	3.423831	-4.290453	0.032557
H	3.581232	-4.463130	1.104968
H	2.520645	-4.813067	-0.270541
H	4.275825	-4.683498	-0.517023
C	5.305702	-2.510630	-1.633248
H	4.697502	-2.632994	-2.532286
H	6.121997	-1.817777	-1.856896
H	5.755116	-3.474956	-1.383249
C	4.009234	-0.508034	-0.827784
H	3.435856	-0.053614	-0.020423
H	4.886344	0.114543	-1.027278
H	3.383368	-0.515862	-1.723698
C	5.318274	-1.900746	0.819903
H	4.741155	-1.498623	1.656887
H	5.688713	-2.893852	1.090224
H	6.183480	-1.251516	0.657252

C8-TS4S

C	0.263240	0.872300	-0.353641
C	0.113796	1.432540	-1.831989
H	-0.703016	0.922824	-2.337048
C	-0.845434	-0.141000	-0.026141
C	-1.393666	-0.142433	1.256881
C	-1.289328	-1.080660	-0.958405
C	-2.404272	-1.038270	1.610752
H	-1.032126	0.600222	1.953077
C	-2.282887	-2.013280	-0.635759
H	-0.862496	-1.086541	-1.952556
C	-2.832684	-1.964008	0.648701

H	-3.616190	-2.667393	0.910004
C	1.613759	0.210485	-0.058924
C	1.923081	-1.047479	-0.593152
C	2.546400	0.851297	0.746987
C	3.150235	-1.656805	-0.347135
H	1.184003	-1.555036	-1.195356
C	3.796787	0.270767	1.017252
H	2.278031	1.811984	1.166132
C	4.077884	-0.975686	0.460048
H	5.035030	-1.441841	0.656059
O	0.134993	2.019105	0.483357
B	-0.647029	3.007330	-0.155805
N	-0.296354	2.885551	-1.684155
C	1.374063	1.468877	-2.694692
H	1.974271	0.562780	-2.606176
H	1.074198	1.588504	-3.739947
C	2.097426	2.725826	-2.203130
H	2.636966	2.509170	-1.278192
H	2.815728	3.113703	-2.930405
C	0.956823	3.715222	-1.952291
H	1.142911	4.376229	-1.105479
H	0.756029	4.326824	-2.831144
B	-1.404749	3.296711	-2.787516
H	-1.587427	4.492004	-2.696095
H	-0.968648	2.988618	-3.877097
H	-2.431057	2.662614	-2.571450
O	-2.161233	2.452280	-0.077233
C	-3.308032	2.576549	-0.551601
C	-4.012656	1.284930	-0.865963
H	-4.961824	1.424584	-1.379216
H	-3.348325	0.651996	-1.452935
H	-4.182305	0.758083	0.079697
C	3.507287	-3.035566	-0.919364
C	4.792892	1.028229	1.905919
C	-2.783186	-3.060863	-1.640500
C	-3.060998	-1.027249	2.998622
H	-0.589939	4.099589	0.318654
C	-4.089876	3.879938	-0.511861
C	-3.212952	5.072181	-0.114869
H	-3.839940	5.966109	-0.044629
H	-2.732835	4.912232	0.852593
H	-2.437495	5.249414	-0.860285
C	4.750386	-2.905210	-1.825269
H	5.026306	-3.880582	-2.240928
H	4.553167	-2.221738	-2.657212

H	5.612163	-2.519599	-1.273546
C	2.362065	-3.634444	-1.753782
H	1.453093	-3.764985	-1.157623
H	2.118308	-3.005912	-2.616407
H	2.656810	-4.617660	-2.133087
C	3.815362	-4.006593	0.240676
H	4.075486	-4.997059	-0.148618
H	4.652821	-3.655773	0.849805
H	2.945958	-4.113190	0.896828
C	6.114385	0.265173	2.088821
H	6.790392	0.845324	2.724490
H	5.958107	-0.705540	2.569849
H	6.620149	0.096733	1.132761
C	5.108594	2.395461	1.261281
H	5.551252	2.263020	0.268857
H	4.208050	3.005000	1.148280
H	5.817749	2.955817	1.880420
C	4.163654	1.250969	3.298028
H	3.934282	0.293617	3.776481
H	4.854637	1.801334	3.946101
H	3.234572	1.823266	3.232841
C	-4.289984	-2.842776	-1.896442
H	-4.671249	-3.588480	-2.602458
H	-4.871980	-2.925151	-0.974331
H	-4.471215	-1.849458	-2.319762
C	-2.557787	-4.472447	-1.057265
H	-1.494054	-4.646022	-0.866088
H	-3.093152	-4.611479	-0.114149
H	-2.908901	-5.236654	-1.759115
C	-2.048009	-2.977397	-2.988296
H	-2.193838	-2.006624	-3.472379
H	-0.972447	-3.146122	-2.873493
H	-2.433212	-3.745473	-3.665727
C	-2.449772	0.039080	3.921634
H	-1.381782	-0.132569	4.084878
H	-2.572631	1.046407	3.511907
H	-2.946343	0.011628	4.896526
C	-2.879389	-2.408157	3.664091
H	-3.340028	-2.417067	4.657899
H	-3.339372	-3.206332	3.074907
H	-1.817102	-2.645258	3.776709
C	-4.567712	-0.725469	2.845885
H	-5.066294	-1.459450	2.206655
H	-5.062974	-0.738347	3.822882
H	-4.721574	0.265422	2.404453

C	-4.794190	4.168020	-1.851616
H	-4.058278	4.346797	-2.635882
H	-5.446408	3.349100	-2.165401
H	-5.415444	5.061324	-1.735548
C	-5.159537	3.648159	0.595408
H	-5.863706	2.854507	0.333698
H	-4.692812	3.400255	1.554016
H	-5.726952	4.573943	0.725070

C8-TS7Rpri

C	-0.031170	-0.832172	-0.339007
C	0.184523	-1.729890	-1.612397
H	0.859561	-1.229196	-2.297945
C	0.961415	0.337553	-0.225332
C	0.728089	1.269201	0.792090
C	2.123953	0.457603	-0.984574
C	1.610237	2.319194	1.045539
H	-0.171090	1.161901	1.386850
C	3.052050	1.481435	-0.743320
H	2.353503	-0.276311	-1.743927
C	2.773176	2.406156	0.266484
H	3.471977	3.208711	0.452616
C	-1.459386	-0.294354	-0.269335
C	-1.816824	0.847837	-0.997744
C	-2.417137	-0.952146	0.489523
C	-3.118588	1.341784	-0.964305
C	-3.745815	-0.498413	0.530478
H	-2.107802	-1.819568	1.058652
C	-4.070596	0.645726	-0.197486
O	0.205157	-1.678454	0.785543
B	0.513448	-3.028619	0.449256
N	0.862939	-2.999031	-1.121735
C	-1.060722	-2.194968	-2.406627
H	-1.935492	-1.572956	-2.226656
H	-0.829171	-2.139544	-3.475400
C	-1.264131	-3.658627	-2.002270
H	-1.812670	-3.723071	-1.057590
H	-1.805741	-4.238823	-2.753929
C	0.169448	-4.136139	-1.818259
H	0.283812	-5.039031	-1.218313
H	0.654984	-4.295143	-2.785646
B	2.435636	-3.062301	-1.464167
H	2.797950	-4.212772	-1.395597
H	2.619618	-2.554879	-2.550317
H	3.056951	-2.404676	-0.611200
O	1.786003	-3.556887	1.136875
C	2.898079	-2.958738	1.259958
C	4.106594	-3.838517	1.340693
H	4.348147	-3.983255	2.402435
H	3.909424	-4.802114	0.874362
H	4.962643	-3.352784	0.869650
C	4.343100	1.521025	-1.572767
C	1.266371	3.333108	2.145472
H	-0.321332	-3.853057	0.729571
C	3.017454	-1.540144	1.747286
H	4.076464	-1.269294	1.763718
H	2.504488	-0.863261	1.071845
C	2.385961	-1.399721	3.145326
H	2.815866	-2.098806	3.870064
H	2.553710	-0.383445	3.509989
H	1.310215	-1.566944	3.078039
C	5.227344	2.728203	-1.222332
H	6.129118	2.716144	-1.842084
H	4.710360	3.675743	-1.404897
H	5.545713	2.707820	-0.175274
C	5.147566	0.229895	-1.300762
H	5.414817	0.161198	-0.240699
H	4.577971	-0.665455	-1.562555
H	6.073956	0.227509	-1.885781
C	3.992765	1.597747	-3.074282
H	3.409184	2.498016	-3.291640
H	4.907183	1.628634	-3.676442
H	3.410596	0.731495	-3.398966
C	-0.086954	3.998167	1.807252
H	-0.362564	4.721720	2.582267
H	-0.027697	4.527475	0.850715
H	-0.891342	3.261577	1.730702
C	2.327137	4.436952	2.279637
H	3.307046	4.027211	2.544541
H	2.434726	5.009564	1.353135
H	2.035179	5.135387	3.069923
C	1.154777	2.600011	3.499461
H	0.887034	3.303427	4.295457
H	0.394020	1.815514	3.473257
H	2.109172	2.133453	3.765159
H	-1.051361	1.355177	-1.570437
H	-5.085239	1.021921	-0.168388
C	-4.771093	-1.268992	1.372838
C	-6.178558	-0.657673	1.288599
C	-4.323465	-1.259024	2.850413

H	-3.339379	-1.718319	2.975515
H	-4.265970	-0.234099	3.230478
H	-5.036548	-1.814506	3.469469
H	-6.197016	0.369789	1.665557
H	-6.873275	-1.245896	1.895998
H	-6.556738	-0.652863	0.261376
C	-3.531724	2.621006	-1.704570
C	-2.363472	3.242848	-2.486838
C	-4.027021	3.660097	-0.675010
H	-4.886490	3.290584	-0.108935
H	-3.234120	3.902727	0.039770
H	-4.328538	4.584705	-1.179429
H	-1.536649	3.519223	-1.825124
H	-2.702066	4.151010	-2.994985
H	-1.977224	2.559340	-3.249425
C	-4.666681	2.298587	-2.699546
H	-5.545392	1.889719	-2.193216
H	-4.977626	3.204355	-3.231467
H	-4.335225	1.563878	-3.440118
C	-4.844357	-2.727225	0.869666
H	-5.154313	-2.759856	-0.179726
H	-3.876843	-3.229983	0.949800
H	-5.569807	-3.298824	1.458843

C8-TS7S

C	-0.103225	-0.710459	-0.216615
C	0.120735	-1.237161	-1.702966
H	1.028800	-0.784304	-2.096506
C	0.911915	0.392575	0.104341
C	1.511740	0.420242	1.361097
C	1.257635	1.369075	-0.831020
C	2.469301	1.383828	1.688729
H	1.242526	-0.358818	2.062680
C	2.191720	2.369084	-0.533860
H	0.800535	1.351084	-1.814824
C	2.791679	2.353829	0.730293
H	3.526700	3.107793	0.971289
C	-1.529120	-0.218118	0.055085
C	-1.944287	1.057476	-0.349509
C	-2.443041	-1.058019	0.680730
C	-3.257144	1.484921	-0.161676
H	-1.223071	1.721394	-0.803083
C	-3.774843	-0.665714	0.886722
H	-2.094693	-2.025119	1.017549
C	-4.160418	0.602682	0.454664
H	-5.182752	0.926601	0.602369
O	0.139522	-1.843207	0.619324
B	0.897557	-2.813819	-0.080438
N	0.390181	-2.711448	-1.570492
C	-1.035694	-1.131680	-2.695635
H	-1.618065	-0.220067	-2.557485
H	-0.623018	-1.126034	-3.706120
C	-1.874596	-2.408188	-2.469606
H	-2.778820	-2.183174	-1.900227
H	-2.176631	-2.850744	-3.421350
C	-0.971340	-3.365284	-1.665213
H	-1.362096	-3.507982	-0.656348
H	-0.844576	-4.340794	-2.131485
B	1.332766	-3.267070	-2.752085
H	1.397463	-4.472332	-2.636890
H	0.876851	-2.921064	-3.818193
H	2.455394	-2.764105	-2.615985
O	2.358372	-2.210588	-0.179979
C	3.373762	-2.723098	-0.707248
C	4.480270	-1.782164	-1.070887
H	4.132725	-0.750725	-1.008469
H	5.316027	-1.932843	-0.376113
H	4.844576	-2.006700	-2.075799
C	-3.735678	2.876752	-0.597784
C	-4.742476	-1.643336	1.567754
C	2.531318	3.419052	-1.601463
C	3.157812	1.314943	3.059024
H	0.915459	-3.914209	0.394143
C	3.634113	-4.201260	-0.677992
H	2.725647	-4.737007	-0.946790
H	4.403024	-4.444810	-1.414955
C	4.091158	-4.596189	0.742748
H	4.307375	-5.666853	0.769987
H	4.996566	-4.061924	1.047006
H	3.304300	-4.384810	1.470639
C	-6.162497	-1.069764	1.696006
H	-6.595029	-0.837123	0.717726
H	-6.814133	-1.802666	2.181672
H	-6.177335	-0.158975	2.302811
C	-4.220865	-1.971559	2.983177
H	-4.891898	-2.678601	3.483399
H	-3.223608	-2.418184	2.951211
H	-4.161752	-1.064922	3.593442
C	-4.818656	-2.941994	0.735567
H	-3.840424	-3.422591	0.648405

H	-5.503150	-3.657764	1.203902
H	-5.182173	-2.733611	-0.275896
C	-2.613939	3.699616	-1.252643
H	-2.998011	4.682260	-1.543351
H	-1.774649	3.858077	-0.568914
H	-2.229974	3.213595	-2.155264
C	-4.883758	2.725613	-1.618678
H	-5.242041	3.709707	-1.940251
H	-4.544854	2.178549	-2.504167
H	-5.732731	2.182047	-1.195037
C	-4.244012	3.651523	0.637140
H	-5.074295	3.133576	1.125036
H	-3.444286	3.770573	1.374699
H	-4.594104	4.648078	0.346220
C	1.244976	4.171420	-2.006244
H	0.801770	4.674832	-1.141410
H	1.466976	4.928254	-2.766475
H	0.492040	3.494993	-2.419389
C	3.554570	4.452061	-1.102955
H	3.183910	4.999574	-0.230856
H	4.505944	3.982726	-0.833602
H	3.756898	5.181564	-1.893170
C	3.122336	2.711519	-2.840383
H	3.367076	3.441701	-3.619517
H	4.038772	2.173145	-2.577532
H	2.421169	1.989009	-3.267021
C	2.095175	1.362103	4.177172
H	1.386529	0.533976	4.097516
H	2.573548	1.302136	5.160927
H	1.525315	2.295294	4.130036
C	4.143772	2.472527	3.282793
H	4.953275	2.465586	2.546048
H	3.643152	3.444562	3.231794
H	4.598407	2.383692	4.274340
C	3.940086	-0.014568	3.148895
H	4.706664	-0.062812	2.368021
H	4.436366	-0.105038	4.121570
H	3.279440	-0.876462	3.021994

C8-TS7Spri

C	-0.007771	0.667364	-0.173484
C	-0.045949	1.324517	-1.596675
H	-1.093787	1.430736	-1.878541
C	-1.213598	-0.236995	0.065918
C	-1.719871	-0.346865	1.367113
C	-1.784518	-0.989603	-0.955386
C	-2.788265	-1.194136	1.651102
H	-1.260171	0.258364	2.135547
C	-2.851677	-1.867703	-0.705727
H	-1.396874	-0.904666	-1.964042
C	-3.336591	-1.948918	0.598342
H	-4.163507	-2.613191	0.814163
C	1.281963	-0.122160	0.092782
C	1.449190	-1.414680	-0.422409
C	2.319148	0.460098	0.811560
C	2.653220	-2.099406	-0.277692
H	0.618865	-1.884303	-0.930207
C	3.549337	-0.195723	0.976805
H	2.159936	1.440113	1.240078
C	3.697294	-1.464524	0.417222
H	4.638831	-1.987424	0.527259
O	-0.090573	1.775316	0.715194
B	0.371945	3.001217	0.142307
N	0.508310	2.715030	-1.423125
C	0.766782	0.706163	-2.733118
H	0.784639	-0.383814	-2.696829
H	0.317118	1.012004	-3.680223
C	2.171663	1.314097	-2.586401
H	2.805891	0.662355	-1.982458
H	2.654300	1.446096	-3.557838
C	1.959544	2.661618	-1.872103
H	2.608310	2.760495	-1.002723
H	2.131023	3.514920	-2.527247
B	-0.283133	3.735718	-2.394353
H	0.219377	4.829623	-2.294889
H	-0.266095	3.290161	-3.519781
H	-1.464066	3.774501	-2.007177
O	-0.676246	4.147760	0.312857
C	-1.870344	4.178297	-0.088918
C	-2.800248	3.003691	-0.013237
H	-2.304608	2.052154	-0.164359
H	-3.200286	2.993748	1.010092
H	-3.633753	3.128178	-0.705354
C	2.867937	-3.507469	-0.849735
C	4.671264	0.508973	1.751132
C	-3.427090	-2.693605	-1.864127
C	-3.379842	-1.329441	3.060839
H	1.360872	3.489620	0.622900
C	-2.489539	5.535131	-0.289278
H	-3.090279	5.485830	-1.203226

H	-3.215421	5.656939	0.530390
C	-1.498207	6.692243	-0.327019
H	-0.927675	6.750239	0.602528
H	-0.791316	6.555728	-1.147445
H	-2.029945	7.636736	-0.469205
C	4.983076	1.861861	1.074316
H	5.297734	1.712428	0.036316
H	4.112361	2.523014	1.070768
H	5.791155	2.377121	1.604988
C	5.965034	-0.319553	1.790541
H	6.735857	0.225982	2.343312
H	5.815359	-1.281045	2.291654
H	6.351274	-0.513309	0.784718
C	4.205840	0.758098	3.201763
H	3.985200	-0.188308	3.705544
H	4.986465	1.275453	3.770351
H	3.302437	1.372897	3.233520
C	4.031636	-3.470752	-1.863761
H	4.963376	-3.139077	-1.397277
H	4.203569	-4.466779	-2.286319
H	3.807114	-2.784072	-2.686154
C	3.215208	-4.479973	0.297572
H	3.379152	-5.489963	-0.093750
H	4.122343	-4.173743	0.825853
H	2.400907	-4.522569	1.027476
C	1.617747	-4.039984	-1.569667
H	0.757149	-4.102115	-0.896636
H	1.339408	-3.408690	-2.419663
H	1.813573	-5.045410	-1.954794
C	-3.951291	-1.741941	-2.961011
H	-4.746494	-1.099891	-2.569435
H	-3.159666	-1.095023	-3.349031
H	-4.356689	-2.315569	-3.801429
C	-2.311509	-3.587023	-2.450367
H	-1.474839	-2.994382	-2.830537
H	-1.918516	-4.267758	-1.688685
H	-2.698802	-4.187970	-3.280244
C	-4.584904	-3.600232	-1.417623
H	-5.420815	-3.019643	-1.014917
H	-4.958784	-4.170431	-2.273438
H	-4.266317	-4.317157	-0.654476
C	-2.651450	-0.443322	4.083925
H	-1.592652	-0.706623	4.165231
H	-2.718932	0.617000	3.821648
H	-3.104687	-0.571626	5.071737
C	-3.266533	-2.797452	3.525171
H	-3.685814	-2.913630	4.530536
H	-3.804498	-3.476677	2.858111
H	-2.219403	-3.114340	3.552346
C	-4.866363	-0.912947	3.030222
H	-5.448642	-1.539720	2.349038
H	-5.309471	-1.000206	4.028292
H	-4.970399	0.125715	2.700116

C9-B5-TS1R

C	-0.059896	-0.907103	-0.242115
C	0.317274	-1.198058	-1.759566
H	1.086044	-0.491506	-2.065192
C	0.646640	0.365588	0.234351
C	1.202809	0.414292	1.514357
C	0.722800	1.499286	-0.575978
C	1.842683	1.563253	1.980946
H	1.155299	-0.475080	2.131955
C	1.320775	2.682092	-0.122278
H	0.302015	1.476727	-1.576225
C	1.887698	2.693806	1.152454
H	2.370344	3.602231	1.503380
C	-1.567404	-0.785089	0.005308
C	-2.248904	0.397900	-0.278896
C	-2.293766	-1.875877	0.491025
C	-3.632614	0.505513	-0.111148
H	-1.697189	1.262582	-0.622910
C	-3.677853	-1.801326	0.671269
H	-1.761319	-2.785213	0.743458
C	-4.336040	-0.604009	0.359725
H	-5.412144	-0.527989	0.493547
O	0.424220	-2.034702	0.485501
B	1.401426	-2.726515	-0.268217
N	0.952616	-2.558852	-1.759907
C	-0.802624	-1.281383	-2.794934
H	-1.597645	-0.558243	-2.607894
H	-0.374291	-1.076826	-3.778139
C	-1.300813	-2.739506	-2.724403
H	-2.222270	-2.802501	-2.141417
H	-1.505351	-3.134488	-3.721964
C	-0.178905	-3.531470	-2.021284
H	-0.528196	-3.934154	-1.068813
H	0.218609	-4.349327	-2.620182
B	2.057212	-2.708296	-2.926170
H	2.560936	-3.803102	-2.813549

H	1.506097	-2.543710	-3.994185
H	2.888722	-1.815372	-2.763053
O	2.714820	-1.797466	-0.177440
C	3.818894	-1.676040	-0.763970
C	4.730270	-2.804949	-0.995865
C	5.849779	-2.665987	-1.833095
C	4.516422	-4.021510	-0.327944
C	6.725681	-3.729397	-2.013081
H	6.019277	-1.738615	-2.366768
C	5.403024	-5.077933	-0.500153
H	3.658967	-4.123906	0.323121
C	6.504677	-4.936605	-1.345839
H	7.578410	-3.620660	-2.675071
H	5.233242	-6.013713	0.022053
H	7.191150	-5.765871	-1.485922
C	4.282893	-0.261231	-0.983321
H	4.640354	-0.119668	-2.004579
H	3.458058	0.421714	-0.782322
H	5.114576	-0.043589	-0.303423
C	-4.325096	1.815814	-0.426239
C	-3.825776	2.954643	0.488837
C	-4.160838	2.210420	-1.909228
H	-5.400153	1.685394	-0.236734
C	-4.501113	4.292375	0.164996
H	-2.739981	3.059526	0.365800
H	-3.992457	2.679454	1.536238
C	-4.837779	3.548670	-2.227451
H	-3.089812	2.287162	-2.140246
H	-4.559012	1.412462	-2.546298
C	-4.321953	4.665530	-1.311734
H	-4.098268	5.081621	0.810443
H	-5.574584	4.221424	0.390135
H	-4.676147	3.810598	-3.279621
H	-5.923842	3.446133	-2.093710
H	-4.834510	5.608831	-1.533118
H	-3.254320	4.831726	-1.513434
C	1.318634	3.920757	-0.993236
C	2.066649	3.709497	-2.324969
C	-0.119004	4.418089	-1.259550
H	1.843396	4.715933	-0.444816
C	2.047823	4.972719	-3.194339
H	1.595141	2.884968	-2.876013
H	3.096893	3.396306	-2.119458
C	-0.137883	5.678645	-2.131320
H	-0.683239	3.622287	-1.764648

H	-0.626164	4.597743	-0.304757
C	0.614220	5.455692	-3.449347
H	2.561661	4.783699	-4.143866
H	2.610692	5.768388	-2.686361
H	-1.172337	5.984137	-2.327742
H	0.333369	6.505204	-1.581415
H	0.622204	6.375807	-4.044717
H	0.080903	4.700886	-4.044194
C	2.503387	1.566343	3.343751
C	4.040665	1.632012	3.223161
C	1.989168	2.693640	4.260195
H	2.256216	0.612421	3.830120
C	4.722068	1.602855	4.596397
H	4.316182	2.556048	2.696719
H	4.391333	0.801392	2.599129
C	2.669084	2.658486	5.634466
H	2.186278	3.664520	3.786556
H	0.901458	2.612989	4.363853
C	4.196329	2.721285	5.505161
H	5.809313	1.683436	4.480662
H	4.528958	0.631489	5.072673
H	2.304457	3.484043	6.256560
H	2.389228	1.729933	6.151355
H	4.666037	2.661490	6.493707
H	4.481552	3.694019	5.080068
C	-4.443326	-3.001810	1.188748
C	-5.164090	-2.709696	2.520303
C	-5.442707	-3.543539	0.145918
H	-3.711673	-3.798793	1.382727
C	-5.916273	-3.940885	3.039882
H	-5.875911	-1.886989	2.370247
H	-4.435938	-2.359744	3.260451
C	-6.196032	-4.773588	0.666414
H	-6.163622	-2.753077	-0.102596
H	-4.909923	-3.780665	-0.782212
C	-6.903237	-4.476225	1.994746
H	-6.440724	-3.697082	3.971143
H	-5.190680	-4.728650	3.286501
H	-6.918371	-5.118433	-0.082621
H	-5.482464	-5.596180	0.814984
H	-7.405501	-5.375708	2.369243
H	-7.688236	-3.725975	1.824028
H	1.636561	-3.842463	0.089367

C9-B5-TS1Rpri

C	0.275909	1.182245	-0.308803
C	-0.020460	1.611560	-1.786960
H	-1.102885	1.639044	-1.913199
C	-0.808560	0.287648	0.270827
C	-1.082206	0.332405	1.640856
C	-1.448496	-0.663205	-0.520831
C	-1.985087	-0.558748	2.222232
H	-0.583763	1.078244	2.250636
C	-2.327896	-1.598110	0.039240
H	-1.251883	-0.698043	-1.586562
C	-2.588204	-1.530197	1.408066
H	-3.275600	-2.248468	1.847015
C	1.621971	0.466500	-0.112294
C	1.758683	-0.869325	-0.490758
C	2.726303	1.122663	0.437216
C	2.979295	-1.540101	-0.385738
H	0.895654	-1.410567	-0.855728
C	3.962767	0.480623	0.556980
H	2.618741	2.143045	0.786039
C	4.077462	-0.848412	0.128867
H	5.031899	-1.361655	0.211391
O	0.312281	2.434599	0.403203
B	0.433468	3.514817	-0.439214
N	0.469918	3.028619	-1.885834
C	0.683452	0.886072	-2.931838
H	0.779103	-0.186506	-2.761077
H	0.097229	1.039397	-3.839192
C	2.057627	1.580690	-3.058843
H	2.841954	0.975672	-2.598980
H	2.318376	1.736636	-4.107201
C	1.925849	2.924179	-2.312190
H	2.574383	2.942163	-1.434342
H	2.147329	3.793252	-2.928186
B	-0.315950	3.866808	-3.065667
H	-0.038556	5.034075	-2.900831
H	0.104371	3.440089	-4.120528
H	-1.500373	3.631055	-2.947158
O	-1.334729	4.536723	-0.274286
C	-2.405506	4.647274	-0.880307
C	-3.393736	3.541581	-0.888195
C	-4.460444	3.503961	-1.799596
C	-3.256585	2.517924	0.059044
C	-5.364168	2.446605	-1.771257
H	-4.566447	4.284247	-2.544796
C	-4.168322	1.467654	0.094237
C	-5.220694	1.430868	-0.821990
H	-4.047356	0.675883	0.823277
H	-5.931911	0.610510	-0.796597
C	-2.739793	5.947130	-1.564511
H	-3.751808	6.282963	-1.321789
H	-2.004311	6.696675	-1.275421
H	-2.670569	5.796285	-2.646115
H	-2.430987	2.561583	0.757422
H	-6.179020	2.412038	-2.487432
C	3.072621	-2.992171	-0.807337
C	2.177561	-3.891149	0.073124
C	2.722323	-3.181595	-2.298241
H	4.112478	-3.320089	-0.667903
C	2.234621	-5.361289	-0.358011
H	1.141408	-3.534850	0.006212
H	2.473617	-3.782956	1.122520
C	2.785069	-4.653615	-2.721579
H	1.706833	-2.803014	-2.476783
H	3.393515	-2.570654	-2.912481
C	1.879858	-5.523875	-1.841139
H	1.559831	-5.960189	0.264617
H	3.247657	-5.751165	-0.185912
H	2.504180	-4.752920	-3.776430
H	3.820908	-5.011234	-2.638351
H	1.954185	-6.576078	-2.138794
H	0.833243	-5.225602	-1.995769
C	5.153143	1.223833	1.127084
C	5.737251	0.535400	2.376847
C	6.254212	1.438026	0.067582
H	4.802382	2.218302	1.436908
C	6.929497	1.313931	2.946283
H	6.062602	-0.479016	2.110411
H	4.952029	0.421925	3.132584
C	7.447848	2.213396	0.638044
H	6.594676	0.458920	-0.295608
H	5.831416	1.960590	-0.798190
C	8.017080	1.526125	1.885569
H	7.340948	0.788890	3.816057
H	6.581979	2.292180	3.306762
H	8.225415	2.323434	-0.126638
H	7.123343	3.229375	0.902852
H	8.844758	2.113119	2.299900
H	8.435731	0.550845	1.599616
C	-2.939343	-2.681303	-0.823232
C	-3.749931	-2.117300	-2.006940

C	-1.856902	-3.659436	-1.330953
H	-3.632352	-3.258376	-0.194658
C	-4.348043	-3.237467	-2.866657
H	-3.095004	-1.495439	-2.631051
H	-4.534672	-1.451835	-1.631221
C	-2.451947	-4.775110	-2.196818
H	-1.120093	-3.096914	-1.920882
H	-1.313723	-4.078787	-0.476374
C	-3.263088	-4.200967	-3.365125
H	-4.895815	-2.808758	-3.713580
H	-5.081616	-3.798346	-2.270581
H	-1.653681	-5.428259	-2.568531
H	-3.106203	-5.403025	-1.576145
H	-3.712163	-5.010420	-3.951725
H	-2.586080	-3.660638	-4.041632
C	-2.304379	-0.470061	3.699719
C	-3.775973	-0.074408	3.943280
C	-1.964604	-1.769263	4.457199
H	-1.678677	0.327771	4.123303
C	-4.092247	0.041217	5.439130
H	-4.428549	-0.833436	3.490476
H	-3.991267	0.869355	3.429182
C	-2.280003	-1.649474	5.953162
H	-2.545730	-2.597982	4.031146
H	-0.908677	-2.017335	4.302289
C	-3.743011	-1.251867	6.186680
H	-5.149569	0.292364	5.581838
H	-3.512977	0.871969	5.865453
H	-2.054606	-2.593832	6.461814
H	-1.624167	-0.889166	6.399713
H	-3.940690	-1.137437	7.258597
H	-4.396801	-2.060830	5.831201
H	1.004343	4.496526	-0.107695

C9-B5-TS1S

C	-0.064152	-0.970284	-0.443144
C	0.360202	-1.141106	-1.964453
H	1.036127	-0.335234	-2.240301
C	0.601908	0.267035	0.174795
C	1.033625	0.220934	1.503303
C	0.735835	1.460755	-0.533021
C	1.614645	1.331710	2.114663
H	0.936898	-0.712871	2.045104
C	1.280708	2.606311	0.062500
H	0.405439	1.518757	-1.564786
C	1.728435	2.522446	1.380756
H	2.167817	3.402520	1.843016
C	-1.574922	-0.856752	-0.215119
C	-2.251617	0.322622	-0.525403
C	-2.300199	-1.928713	0.310530
C	-3.629878	0.448773	-0.333843
H	-1.697317	1.172051	-0.902990
C	-3.681107	-1.836319	0.511422
H	-1.769973	-2.836816	0.572160
C	-4.333582	-0.641195	0.181774
H	-5.405672	-0.550106	0.336804
O	0.404690	-2.150687	0.204953
B	1.485655	-2.705302	-0.518685
N	1.162567	-2.419260	-2.023212
C	-0.746561	-1.326633	-2.999635
H	-1.595668	-0.661682	-2.835812
H	-0.327981	-1.122327	-3.988505
C	-1.113962	-2.809845	-2.871192
H	-1.843527	-2.944422	-2.069313
H	-1.546762	-3.213291	-3.790369
C	0.210495	-3.502204	-2.515947
H	0.082616	-4.256865	-1.738623
H	0.678184	-3.971360	-3.380191
B	2.384356	-2.294462	-3.085859
H	2.986842	-3.345405	-3.064680
H	1.891131	-2.059119	-4.170274
H	3.093092	-1.356756	-2.742961
O	2.733107	-1.729230	-0.192239
C	3.899313	-1.463621	-0.562393
C	4.898345	-2.483706	-0.898604
C	6.116708	-2.112238	-1.492777
C	4.663302	-3.834751	-0.595129
C	7.071911	-3.075413	-1.793372
H	6.305850	-1.075377	-1.743992
C	5.628704	-4.791952	-0.881228
H	3.726276	-4.119098	-0.136708
C	6.830272	-4.416212	-1.485311
H	8.003057	-2.784005	-2.267869
H	5.442335	-5.833311	-0.640597
H	7.578816	-5.167646	-1.716908
C	4.286741	-0.010556	-0.499260
H	4.450000	0.356620	-1.517078
H	3.476775	0.558050	-0.045379
H	5.213554	0.126107	0.064298
C	-4.313074	1.762550	-0.654952

C	-3.793272	2.898563	0.253030
C	-4.156212	2.149062	-2.140509
H	-5.387843	1.644104	-0.456324
C	-4.447926	4.245927	-0.072604
H	-2.706530	2.984945	0.125677
H	-3.959851	2.629798	1.302143
C	-4.815971	3.496107	-2.457908
H	-3.086646	2.209794	-2.382357
H	-4.571761	1.354720	-2.770945
C	-4.273429	4.608645	-1.552396
H	-4.026516	5.031131	0.565972
H	-5.520790	4.194980	0.160673
H	-4.661218	3.750796	-3.512853
H	-5.902068	3.411237	-2.312450
H	-4.771038	5.559688	-1.774747
H	-3.204492	4.755082	-1.763339
C	1.347970	3.907721	-0.708791
C	2.202170	3.794913	-1.987473
C	-0.064689	4.429949	-1.050702
H	1.825061	4.656424	-0.060459
C	2.249523	5.119638	-2.758713
H	1.780766	3.015154	-2.635416
H	3.213414	3.464513	-1.722820
C	-0.016592	5.751611	-1.825322
H	-0.585881	3.675986	-1.656217
H	-0.645973	4.540810	-0.128340
C	0.839425	5.624185	-3.091529
H	2.838619	5.000303	-3.675223
H	2.767064	5.873769	-2.149249
H	-1.032888	6.074148	-2.080631
H	0.406860	6.533642	-1.179694
H	0.892329	6.585615	-3.614986
H	0.357898	4.916770	-3.781212
C	2.133527	1.236435	3.534305
C	3.672377	1.341035	3.583829
C	1.494790	2.276562	4.475476
H	1.863137	0.241949	3.915709
C	4.206045	1.215302	5.015805
H	3.976858	2.309077	3.163087
H	4.109873	0.569546	2.939156
C	2.027166	2.144738	5.907590
H	1.713561	3.285679	4.101737
H	0.404904	2.166658	4.455381
C	3.557232	2.247127	5.947203
H	5.296796	1.325542	5.023573
H	3.990685	0.205430	5.391877
H	1.576582	2.910754	6.549200
H	1.720329	1.172618	6.318105
H	3.922334	2.117523	6.972527
H	3.858316	3.256070	5.631545
C	-4.448256	-3.014678	1.075422
C	-5.127641	-2.682608	2.419439
C	-5.483593	-3.565081	0.073121
H	-3.722378	-3.817401	1.267240
C	-5.882405	-3.890724	2.987236
H	-5.831496	-1.852905	2.270202
H	-4.374378	-2.327007	3.131266
C	-6.239069	-4.772241	0.641934
H	-6.200608	-2.770344	-0.172979
H	-4.980092	-3.829865	-0.863651
C	-6.905212	-4.434866	1.981873
H	-6.377485	-3.618586	3.926710
H	-5.161352	-4.683320	3.231731
H	-6.986527	-5.123362	-0.079030
H	-5.533182	-5.601566	0.789822
H	-7.409618	-5.318192	2.390433
H	-7.683926	-3.677131	1.815478
H	1.762534	-3.833754	-0.238527

C9-B5-TS1Spri

C	0.514810	-0.520517	-0.161200
C	1.011619	-0.732876	-1.633158
H	1.666435	0.104908	-1.876170
C	0.425189	0.962263	0.188464
C	0.736439	1.380314	1.485970
C	-0.036631	1.901870	-0.733314
C	0.587281	2.714351	1.868151
H	1.104145	0.645223	2.193152
C	-0.235106	3.239510	-0.368802
H	-0.272219	1.593754	-1.745900
C	0.088743	3.630248	0.930638
H	-0.056760	4.668699	1.216883
C	-0.843215	-1.184473	0.115337
C	-2.032192	-0.571634	-0.280440
C	-0.907043	-2.439515	0.726891
C	-3.268294	-1.206338	-0.130486
H	-2.003924	0.424088	-0.703787
C	-2.127081	-3.101432	0.892248
H	0.007783	-2.903410	1.075119
C	-3.300090	-2.476974	0.447152

H	-4.256915	-2.980100	0.560646
O	1.526183	-1.121662	0.632578
B	2.276966	-2.133146	-0.055708
N	1.866274	-1.972123	-1.599064
C	-0.005763	-0.976689	-2.747053
H	-0.922744	-0.400110	-2.618945
H	0.450935	-0.687933	-3.696384
C	-0.268758	-2.491851	-2.716759
H	-1.144550	-2.712779	-2.103722
H	-0.452943	-2.885588	-3.719297
C	0.990647	-3.114574	-2.085159
H	0.739018	-3.754536	-1.240319
H	1.574333	-3.698241	-2.796496
B	3.098920	-1.811114	-2.609075
H	3.750177	-2.827818	-2.617463
H	2.694964	-1.446831	-3.689627
H	3.824387	-0.899646	-2.146234
O	3.800238	-1.909044	0.047977
C	4.479116	-0.927009	-0.406244
C	5.884709	-1.227052	-0.750267
C	6.826736	-0.207709	-0.954256
C	6.282922	-2.568794	-0.864920
C	8.146172	-0.525648	-1.262808
H	6.536221	0.832742	-0.869892
C	7.600329	-2.881593	-1.176305
C	8.534389	-1.861574	-1.375223
H	7.901306	-3.920137	-1.268207
H	9.563197	-2.107743	-1.619551
C	4.052439	0.495495	-0.163419
H	2.981292	0.563599	-0.010416
H	4.538235	0.821897	0.765440
H	4.368905	1.157176	-0.968945
H	5.541823	-3.343541	-0.715826
H	8.870537	0.267588	-1.416529
C	-4.530535	-0.506915	-0.591653
C	-4.777358	0.800087	0.191817
C	-4.504060	-0.224089	-2.108713
H	-5.380218	-1.176459	-0.395812
C	-6.039692	1.527265	-0.286166
H	-3.909844	1.460614	0.064338
H	-4.840650	0.576537	1.262589
C	-5.768440	0.502133	-2.581486
H	-3.627960	0.397231	-2.338759
H	-4.368693	-1.165175	-2.653877
C	-5.991349	1.799657	-1.794713

H	-6.164409	2.465059	0.267655
H	-6.921169	0.910841	-0.060287
H	-5.702171	0.711844	-3.655443
H	-6.637398	-0.156590	-2.444126
H	-6.913049	2.293043	-2.124096
H	-5.167599	2.495342	-2.008021
C	-0.845755	4.216310	-1.351986
C	-0.029720	4.351350	-2.652626
C	-2.307547	3.833753	-1.674374
H	-0.865122	5.205238	-0.872355
C	-0.679626	5.339248	-3.629409
H	0.047212	3.367824	-3.134862
H	0.993847	4.660459	-2.412682
C	-2.956043	4.817444	-2.654480
H	-2.321726	2.825603	-2.111088
H	-2.883529	3.775166	-0.743791
C	-2.130137	4.948153	-3.940186
H	-0.092920	5.395019	-4.553498
H	-0.664804	6.345604	-3.187908
H	-3.978681	4.497612	-2.886826
H	-3.037803	5.803040	-2.175376
H	-2.586828	5.681671	-4.614378
H	-2.137325	3.985865	-4.471295
C	0.951758	3.153673	3.271057
C	2.124176	4.156180	3.275006
C	-0.254015	3.735531	4.036449
H	1.284620	2.260719	3.818414
C	2.505558	4.578421	4.698952
H	1.836811	5.044338	2.696193
H	2.984179	3.714078	2.758858
C	0.129138	4.156247	5.460476
H	-0.638197	4.608722	3.492384
H	-1.064722	2.998778	4.054817
C	1.298976	5.148412	5.455457
H	3.319787	5.311867	4.669575
H	2.890864	3.704358	5.242425
H	-0.738103	4.590619	5.971095
H	0.415807	3.264140	6.034661
H	1.581498	5.410769	6.481496
H	0.977833	6.080716	4.969760
C	-2.162639	-4.481516	1.515553
C	-3.061202	-4.549036	2.766204
C	-2.585566	-5.556166	0.492273
H	-1.139439	-4.722697	1.835828
C	-3.064008	-5.952348	3.384910

H	-4.087807	-4.275087	2.488646
H	-2.727168	-3.804278	3.497158
C	-2.592760	-6.959035	1.111018
H	-3.590191	-5.316253	0.118401
H	-1.914652	-5.518813	-0.373802
C	-3.481149	-7.015014	2.360292
H	-3.729553	-5.979080	4.255555
H	-2.055527	-6.184861	3.754568
H	-2.926310	-7.695977	0.371140
H	-1.565548	-7.235330	1.387028
H	-3.444390	-8.014018	2.809933
H	-4.526014	-6.840786	2.066688
H	2.146490	-3.262039	0.342229

C9-TS4S

C	-0.511829	-0.742776	-0.656665
C	-0.276869	-0.610983	-2.224940
H	0.743786	-0.272019	-2.392277
C	0.644478	-0.088364	0.104923
C	1.145609	-0.691863	1.258494
C	1.166342	1.145065	-0.296425
C	2.149205	-0.077612	2.015746
H	0.736639	-1.652196	1.548725
C	2.150878	1.798081	0.452500
H	0.790505	1.620647	-1.196805
C	2.636585	1.167365	1.601170
H	3.400464	1.663557	2.195840
C	-1.841916	-0.141107	-0.177002
C	-1.991543	1.234504	0.014203
C	-2.941179	-0.970582	0.047820
C	-3.214812	1.791812	0.393420
H	-1.139720	1.888532	-0.117693
C	-4.182958	-0.443658	0.421783
H	-2.808753	-2.038974	-0.063484
C	-4.305273	0.939139	0.586322
H	-5.265686	1.358658	0.877864
O	-0.545649	-2.143281	-0.397009
B	-0.009840	-2.872465	-1.490244
N	-0.371634	-2.011972	-2.761509
C	-1.280577	0.196595	-3.046353
H	-1.644303	1.075111	-2.512321
H	-0.787538	0.530211	-3.961237
C	-2.412331	-0.799626	-3.382279
H	-3.288933	-0.618611	-2.756732
H	-2.717033	-0.705429	-4.426829
C	-1.836392	-2.199988	-3.087876
H	-2.333323	-2.648458	-2.226190
H	-1.908577	-2.887531	-3.928781
B	0.500168	-2.224601	-4.098924
H	0.272824	-3.340474	-4.511453
H	0.220884	-1.359410	-4.898023
H	1.695716	-2.115500	-3.801557
O	1.550347	-2.697942	-1.384306
C	2.448925	-3.161657	-2.130839
C	2.338375	-4.486882	-2.814692
H	2.794303	-4.440485	-3.803742
H	2.914103	-5.201289	-2.209078
H	1.308721	-4.822163	-2.895490
H	-0.314391	-4.030759	-1.543402
C	2.682716	-0.728837	3.275925
C	1.569485	-0.983937	4.312535
C	3.446743	-2.034537	2.977122
H	3.399772	-0.030757	3.731431
C	2.118595	-1.630832	5.589573
H	0.812077	-1.643227	3.869624
H	1.062530	-0.040308	4.542716
C	3.988858	-2.686943	4.254467
H	2.769920	-2.733468	2.467761
H	4.265387	-1.824317	2.280154
C	2.872566	-2.929564	5.277678
H	1.301604	-1.823262	6.294394
H	2.802041	-0.928038	6.086731
H	4.495807	-3.627804	4.009922
H	4.748386	-2.028941	4.699660
H	3.285132	-3.362564	6.196204
H	2.166080	-3.666879	4.871485
C	2.622210	3.184388	0.064386
C	3.193839	3.251883	-1.364797
C	1.489595	4.220997	0.236733
H	3.430488	3.466597	0.753869
C	3.648808	4.669856	-1.731061
H	2.427604	2.922829	-2.078972
H	4.026672	2.546835	-1.456144
C	1.946847	5.637161	-0.129833
H	0.646858	3.934611	-0.407328
H	1.116769	4.186837	1.266633
C	2.514630	5.686996	-1.553382
H	4.022543	4.689544	-2.761204
H	4.491983	4.955295	-1.086486
H	1.112814	6.340799	-0.025390

H	2.720374	5.961794	0.580058
H	2.868099	6.697292	-1.788854
H	1.711580	5.459994	-2.268666
C	-5.372066	-1.358529	0.629249
C	-5.752600	-2.104133	-0.666930
C	-5.143285	-2.363468	1.776544
H	-6.230681	-0.731452	0.909427
C	-6.964723	-3.020594	-0.462966
H	-4.894555	-2.705465	-0.995992
H	-5.946915	-1.376748	-1.463599
C	-6.356412	-3.279380	1.979665
H	-4.260917	-2.974439	1.545423
H	-4.910704	-1.818706	2.698436
C	-6.728859	-4.011274	0.684100
H	-7.191730	-3.557318	-1.391442
H	-7.846568	-2.406143	-0.232963
H	-6.153911	-3.999880	2.780613
H	-7.213191	-2.675808	2.311103
H	-7.617491	-4.634063	0.839144
H	-5.911454	-4.692232	0.408317
C	-3.331587	3.288465	0.596957
C	-2.425837	3.778304	1.746933
C	-3.024657	4.071765	-0.696618
H	-4.371112	3.511048	0.877065
C	-2.524887	5.294694	1.950712
H	-1.386006	3.510753	1.519168
H	-2.687042	3.245709	2.668170
C	-3.127915	5.587081	-0.488097
H	-2.007835	3.824108	-1.030161
H	-3.701276	3.742172	-1.493260
C	-2.218313	6.054932	0.654513
H	-1.843660	5.611141	2.749226
H	-3.539972	5.550122	2.285924
H	-2.876888	6.112562	-1.416873
H	-4.168527	5.849666	-0.251326
H	-2.324353	7.134790	0.810104
H	-1.170780	5.878147	0.374031
C	3.795558	-2.502737	-2.011355
C	3.727956	-0.969375	-2.141603
C	4.364078	-2.895409	-0.617293
H	4.452504	-2.910273	-2.788844
C	5.099668	-0.336244	-1.894503
H	3.010959	-0.587647	-1.408496
H	3.347988	-0.709783	-3.133675
C	5.720995	-2.233711	-0.356239
H	3.642746	-2.567183	0.137068
H	4.449907	-3.985362	-0.534885
C	5.642046	-0.710669	-0.511231
H	5.021386	0.751047	-1.993295
H	5.805916	-0.669585	-2.668011
H	6.062143	-2.502466	0.650257
H	6.465425	-2.634593	-1.057636
H	6.629175	-0.263079	-0.350228
H	4.972787	-0.299279	0.256210

C9-TS4R

C	-0.017092	0.554880	-0.331445
C	-0.333893	0.465342	-1.890844
H	-0.620518	-0.558899	-2.122995
C	-0.047882	-0.852734	0.272517
C	-0.720645	-1.077362	1.471690
C	0.585518	-1.926952	-0.358398
C	-0.792893	-2.356466	2.030370
H	-1.226696	-0.239621	1.934729
C	0.584494	-3.206806	0.206062
H	1.095777	-1.771772	-1.304022
C	-0.122197	-3.407153	1.395097
H	-0.155768	-4.402778	1.832273
C	1.314510	1.246720	-0.013441
C	2.526389	0.554519	-0.062154
C	1.335135	2.608608	0.291232
C	3.746598	1.197531	0.159203
H	2.527577	-0.508566	-0.260152
C	2.540595	3.284170	0.513479
H	0.390770	3.132202	0.366859
C	3.738368	2.566535	0.439351
H	4.680649	3.082350	0.611017
O	-1.067915	1.347998	0.217251
B	-2.186310	1.355178	-0.655530
N	-1.551764	1.323105	-2.104476
C	0.706915	0.992414	-2.878123
H	1.726887	0.809057	-2.537926
H	0.567222	0.481884	-3.832836
C	0.395920	2.497937	-3.028522
H	1.124328	3.102875	-2.484503
H	0.424946	2.798690	-4.078133
C	-1.008826	2.697858	-2.425233
H	-0.953287	3.275028	-1.500842
H	-1.708360	3.188266	-3.100186
B	-2.448925	0.799468	-3.329766

H	-3.331403	1.607348	-3.525060
H	-1.744533	0.624006	-4.297738
H	-2.939526	-0.291010	-3.000036
O	-2.827413	-0.075941	-0.533695
C	-3.821832	-0.536679	-1.153097
C	-3.911585	-2.031836	-1.209497
H	-4.169191	-2.363908	-2.216404
H	-2.963725	-2.466682	-0.892513
H	-4.710703	-2.366534	-0.536901
H	-2.993137	2.220488	-0.460871
C	-1.633021	-2.604456	3.265612
C	-1.215578	-1.726686	4.461475
C	-3.133784	-2.410368	2.954452
H	-1.493581	-3.653801	3.563090
C	-2.094599	-1.986922	5.690810
H	-1.296504	-0.669695	4.177575
H	-0.160066	-1.905282	4.694963
C	-4.012838	-2.660891	4.185184
H	-3.288074	-1.384806	2.593041
H	-3.421486	-3.075669	2.131378
C	-3.580607	-1.785405	5.368157
H	-1.791162	-1.331955	6.515609
H	-1.938003	-3.018543	6.036418
H	-5.066220	-2.479788	3.940060
H	-3.936458	-3.718690	4.473688
H	-4.196084	-2.003460	6.248583
H	-3.753632	-0.729649	5.116440
C	1.350745	-4.335055	-0.452318
C	0.845016	-4.644110	-1.875883
C	2.867812	-4.046962	-0.474710
H	1.198880	-5.239983	0.153084
C	1.636786	-5.785608	-2.525565
H	0.938979	-3.742044	-2.495019
H	-0.223535	-4.885390	-1.839854
C	3.658617	-5.186970	-1.126158
H	3.045270	-3.118976	-1.035254
H	3.220264	-3.862776	0.546552
C	3.141461	-5.487082	-2.538501
H	1.273805	-5.962938	-3.544485
H	1.458843	-6.713330	-1.963750
H	4.725579	-4.936473	-1.154653
H	3.565991	-6.090410	-0.507350
H	3.693108	-6.326583	-2.976842
H	3.330007	-4.616497	-3.182428
C	2.541698	4.767651	0.819701
C	1.945577	5.593664	-0.339194
C	1.809006	5.089957	2.138090
H	3.588156	5.082660	0.940719
C	1.956564	7.096214	-0.033800
H	0.911561	5.267218	-0.513700
H	2.498963	5.382317	-1.261535
C	1.820491	6.592965	2.442241
H	0.770599	4.741519	2.063275
H	2.267139	4.525416	2.957873
C	1.230140	7.403755	1.281678
H	1.501174	7.652283	-0.861527
H	2.997402	7.441528	0.040097
H	1.268829	6.793776	3.367971
H	2.855692	6.917974	2.618128
H	1.277709	8.476516	1.501947
H	0.165933	7.151931	1.171916
C	5.035675	0.403489	0.100448
C	5.077065	-0.695010	1.184225
C	5.266413	-0.213545	-1.295275
H	5.868149	1.094132	0.296984
C	6.369497	-1.517377	1.120439
H	4.217035	-1.363178	1.046726
H	4.957367	-0.236650	2.172199
C	6.559288	-1.035427	-1.354420
H	4.416295	-0.864164	-1.541009
H	5.276645	0.581500	-2.049586
C	6.578041	-2.122809	-0.273123
H	6.349656	-2.306639	1.880967
H	7.224149	-0.870234	1.363044
H	6.675526	-1.482092	-2.348800
H	7.419824	-0.367473	-1.208653
H	7.519707	-2.682668	-0.310283
H	5.773949	-2.844105	-0.475052
C	-5.010150	0.323638	-1.474584
C	-5.901525	-0.204679	-2.604330
C	-5.818966	0.493965	-0.157591
H	-4.632693	1.307441	-1.757720
C	-7.092810	0.734089	-2.828016
H	-6.276823	-1.204614	-2.349495
H	-5.305281	-0.296276	-3.516757
C	-7.017179	1.421892	-0.392027
H	-6.175331	-0.486207	0.186847
H	-5.166329	0.896651	0.622940
C	-7.904368	0.926525	-1.540629
H	-7.731069	0.340529	-3.626489

H	-6.719594	1.706802	-3.174494
H	-7.598310	1.508195	0.532942
H	-6.644648	2.427553	-0.626754
H	-8.728001	1.628804	-1.710231
H	-8.361863	-0.031369	-1.255533

C9-TS7R

C	-0.435272	-0.691737	-0.724642
C	-0.251556	-0.535084	-2.297211
H	0.814143	-0.517880	-2.518483
C	0.908840	-0.504913	-0.018051
C	1.247091	-1.331917	1.051955
C	1.792355	0.508252	-0.397099
C	2.455149	-1.166504	1.737021
H	0.558316	-2.123063	1.321942
C	2.990186	0.724626	0.291479
H	1.547180	1.150115	-1.237490
C	3.310083	-0.128063	1.351081
H	4.245214	0.018413	1.887114
C	-1.468307	0.270848	-0.123885
C	-1.140089	1.600512	0.149397
C	-2.769636	-0.162877	0.130165
C	-2.085719	2.502353	0.641229
H	-0.125897	1.942885	-0.007319
C	-3.743623	0.716933	0.617809
H	-3.008704	-1.203653	-0.047390
C	-3.388023	2.046555	0.864348
H	-4.137744	2.737203	1.244226
O	-0.904930	-2.025409	-0.533258
B	-0.676539	-2.808092	-1.689851
N	-0.804024	-1.800238	-2.892209
C	-0.992782	0.596283	-3.008275
H	-1.044512	1.502425	-2.403835
H	-0.461182	0.829335	-3.932463
C	-2.390205	0.019521	-3.327311
H	-3.144297	0.424906	-2.649339
H	-2.687683	0.264141	-4.349190
C	-2.268775	-1.504838	-3.127424
H	-2.837041	-1.825018	-2.252599
H	-2.595201	-2.086123	-3.988050
B	-0.108712	-2.171098	-4.302747
H	-0.687309	-3.139785	-4.747016
H	-0.184517	-1.212054	-5.037274
H	1.079304	-2.427869	-4.093348
O	0.885311	-3.136470	-1.682413
C	1.529853	-3.850600	-2.481636
C	0.927967	-5.023938	-3.189754
H	1.495064	-5.265025	-4.089091
H	0.992156	-5.876641	-2.499646
H	-0.113512	-4.850323	-3.443456
C	3.019531	-3.666063	-2.526893
H	3.450240	-4.576222	-2.079979
H	3.313771	-3.696639	-3.581745
C	3.530708	-2.409052	-1.830555
H	3.230801	-2.378727	-0.782167
H	4.621704	-2.368909	-1.885295
H	3.130112	-1.514006	-2.310988
H	-1.315008	-3.817768	-1.775546
C	2.838163	-2.107537	2.860191
C	1.809630	-2.102435	4.008765
C	3.056620	-3.544882	2.341617
H	3.795509	-1.760971	3.275317
C	2.211831	-3.061617	5.135494
H	0.829527	-2.398824	3.613504
H	1.694186	-1.082099	4.391021
C	3.452977	-4.505462	3.469149
H	2.128661	-3.895088	1.870241
H	3.821535	-3.535822	1.555886
C	2.425079	-4.485849	4.607467
H	1.449869	-3.056732	5.923353
H	3.143134	-2.705985	5.598509
H	3.568386	-5.522243	3.075475
H	4.434737	-4.210319	3.865449
H	2.742479	-5.150891	5.418910
H	1.468386	-4.877968	4.234890
C	3.898417	1.872588	-0.095293
C	4.413538	1.754640	-1.543991
C	3.205631	3.236170	0.117802
H	4.774742	1.843869	0.567708
C	5.327267	2.927605	-1.919590
H	3.555994	1.731499	-2.229631
H	4.936470	0.799568	-1.669332
C	4.117899	4.408247	-0.260752
H	2.296508	3.273724	-0.497756
H	2.876613	3.320261	1.159822
C	4.626956	4.275071	-1.701450
H	5.654111	2.831339	-2.961340
H	6.235278	2.889603	-1.301532
H	3.584763	5.357185	-0.129195
H	4.976106	4.433967	0.425174

H	5.305185	5.100953	-1.944518
H	3.775568	4.353812	-2.392086
C	-5.159175	0.234971	0.858853
C	-5.831357	-0.239949	-0.446339
C	-5.225150	-0.874235	1.928330
H	-5.742400	1.087822	1.234886
C	-7.269339	-0.715306	-0.207118
H	-5.242426	-1.064626	-0.869740
H	-5.809101	0.569816	-1.184787
C	-6.663919	-1.348684	2.166733
H	-4.611985	-1.723707	1.600139
H	-4.777908	-0.510432	2.860166
C	-7.323721	-1.813491	0.862351
H	-7.709020	-1.072858	-1.145474
H	-7.880977	0.137158	0.120382
H	-6.676015	-2.154940	2.909319
H	-7.250828	-0.522500	2.592366
H	-8.361123	-2.116324	1.045815
H	-6.797113	-2.703627	0.490487
C	-1.680083	3.934277	0.925318
C	-0.591579	4.010940	2.017435
C	-1.207326	4.658882	-0.352811
H	-2.564255	4.469462	1.299979
C	-0.153862	5.454010	2.294553
H	0.279017	3.425929	1.693721
H	-0.960127	3.534940	2.932941
C	-0.771320	6.101125	-0.069775
H	-0.360439	4.106238	-0.781546
H	-2.005450	4.634417	-1.103495
C	0.314562	6.152220	1.011939
H	0.641675	5.465936	3.048722
H	-0.997846	6.014184	2.721097
H	-0.415570	6.574040	-0.992571
H	-1.641171	6.683397	0.265230
H	0.596190	7.190444	1.222383
H	1.218017	5.649762	0.638613

C9-TS7Rpri

C	0.061978	-1.281031	-0.618091
C	-0.406325	-2.641769	0.011940
H	-1.056624	-2.455178	0.860472
C	-0.788753	-0.062460	-0.214264
C	-0.368726	1.189814	-0.685597
C	-1.994316	-0.142889	0.478922
C	-1.124700	2.340882	-0.473286
H	0.566858	1.259494	-1.230580
C	-2.785084	0.995018	0.699065
H	-2.367345	-1.098669	0.819365
C	-2.336537	2.226049	0.224724
H	-2.945101	3.109663	0.398202
C	1.542395	-0.974456	-0.336269
C	2.014499	-0.625266	0.946569
C	2.447654	-1.025653	-1.400087
C	3.373250	-0.324048	1.095833
C	3.795930	-0.729354	-1.221888
H	2.069071	-1.292321	-2.378491
C	4.282902	-0.365281	0.036953
H	3.741227	-0.045373	2.079686
H	4.467883	-0.777980	-2.073932
O	-0.126658	-1.429801	-2.026656
B	-0.685601	-2.681610	-2.421698
N	-1.227461	-3.341200	-1.058645
C	0.685365	-3.655689	0.441589
H	1.671589	-3.202229	0.519898
H	0.424659	-4.050887	1.428413
C	0.632990	-4.778769	-0.601283
H	1.255543	-4.528650	-1.464897
H	0.966470	-5.741833	-0.206116
C	-0.836217	-4.789971	-0.995887
H	-1.052522	-5.254299	-1.958214
H	-1.445454	-5.277132	-0.228668
B	-2.820814	-3.238772	-0.841481
H	-3.348904	-4.124905	-1.470082
H	-3.057770	-3.276874	0.347503
H	-3.221205	-2.151224	-1.289799
O	-1.927704	-2.554834	-3.324816
C	-2.920920	-1.778700	-3.180022
C	-4.224732	-2.278810	-3.720130
H	-4.352753	-1.863523	-4.728974
H	-4.226814	-3.366258	-3.767904
H	-5.053076	-1.920438	-3.106881
H	0.038463	-3.413035	-3.048022
C	-2.776377	-0.303300	-2.925463
H	-3.775151	0.135405	-2.855413
H	-2.265186	-0.132110	-1.984065
C	-1.955556	0.357287	-4.048527
H	-2.380206	0.172846	-5.040681
H	-1.933385	1.437318	-3.882409
H	-0.931169	-0.016549	-4.020743
C	5.742103	-0.026593	0.256682

C	6.668947	-1.221722	-0.045914
C	6.179957	1.209495	-0.555504
H	5.867963	0.221728	1.320445
C	8.142636	-0.873926	0.196853
H	6.534445	-1.521816	-1.093689
H	6.368325	-2.080960	0.564129
C	7.653872	1.556188	-0.312616
H	6.025312	1.007722	-1.623869
H	5.535620	2.059310	-0.303515
C	8.566777	0.359889	-0.609738
H	8.778306	-1.731248	-0.053228
H	8.294035	-0.673852	1.266953
H	7.943628	2.419185	-0.923322
H	7.784945	1.857186	0.736328
H	9.611375	0.614693	-0.396645
H	8.513178	0.124325	-1.681992
C	1.117704	-0.559300	2.171016
C	1.608828	-1.449182	3.330693
C	0.915422	0.889674	2.661868
H	0.127458	-0.930482	1.901303
C	0.626408	-1.404405	4.507899
H	2.594672	-1.106393	3.669898
H	1.741063	-2.478274	2.979603
C	-0.059196	0.940958	3.843351
H	1.887413	1.304468	2.961762
H	0.545781	1.505894	1.837929
C	0.396707	0.033227	4.992246
H	0.992380	-2.030797	5.329767
H	-0.333861	-1.834868	4.189644
H	-0.174450	1.973493	4.193542
H	-1.047993	0.616786	3.494207
H	-0.338097	0.047471	5.805716
H	1.335387	0.424104	5.409916
C	-0.630967	3.683141	-0.969753
C	-1.617260	4.352782	-1.947093
C	-0.301336	4.635528	0.198514
H	0.303906	3.506453	-1.519865
C	-1.090633	5.703824	-2.446146
H	-2.580425	4.503871	-1.441890
H	-1.811133	3.679581	-2.790564
C	0.221914	5.987441	-0.300813
H	-1.209030	4.793310	0.796445
H	0.429014	4.158549	0.861898
C	-0.762679	6.642257	-1.277827
H	-1.822388	6.169173	-3.116644
H	-0.181619	5.538144	-3.041079
H	0.417376	6.652560	0.548230
H	1.185112	5.835519	-0.807629
H	-0.356337	7.588843	-1.652233
H	-1.690749	6.888327	-0.742786
C	-4.127874	0.862800	1.386485
C	-5.111158	0.041359	0.523535
C	-4.016203	0.255679	2.799290
H	-4.548697	1.872630	1.496005
C	-6.480904	-0.101321	1.196871
H	-4.684527	-0.953560	0.345589
H	-5.211866	0.517505	-0.459438
C	-5.388913	0.112156	3.467472
H	-3.545174	-0.733516	2.729876
H	-3.352955	0.876147	3.412336
C	-6.350386	-0.708628	2.599116
H	-7.142132	-0.715917	0.575018
H	-6.953281	0.888269	1.274983
H	-5.278050	-0.348119	4.456068
H	-5.816105	1.111258	3.633451
H	-7.333529	-0.776872	3.078966
H	-5.969132	-1.735262	2.511157

C9-TS7S

C	-0.326044	-0.681857	-0.636514
C	-0.208373	-0.598785	-2.223529
H	0.828437	-0.386299	-2.478926
C	0.997443	-0.240745	-0.004611
C	1.545234	-0.978018	1.043151
C	1.670141	0.899017	-0.453678
C	2.759159	-0.607300	1.628826
H	1.023126	-1.870611	1.364595
C	2.856577	1.329335	0.149244
H	1.265324	1.471734	-1.282350
C	3.394406	0.556256	1.181887
H	4.329482	0.864278	1.644754
C	-1.489915	0.135342	-0.061068
C	-1.374585	1.509098	0.162300
C	-2.709616	-0.485755	0.211698
C	-2.452420	2.269363	0.621937
H	-0.426595	2.000985	-0.007743
C	-3.810850	0.247661	0.668426
H	-2.782502	-1.557039	0.074835
C	-3.668447	1.624780	0.864049
H	-4.518258	2.203577	1.219335

O	-0.570667	-2.058850	-0.351911
B	-0.186110	-2.864940	-1.451570
N	-0.509835	-1.985895	-2.721583
C	-1.171282	0.316716	-2.978485
H	-1.394955	1.228057	-2.422581
H	-0.708899	0.597810	-3.926402
C	-2.428924	-0.543414	-3.233151
H	-3.241359	-0.255008	-2.562923
H	-2.780445	-0.424256	-4.260314
C	-2.005502	-1.998717	-2.949988
H	-2.493804	-2.374152	-2.049267
H	-2.215287	-2.682283	-3.770850
B	0.243629	-2.313508	-4.106867
H	-0.158499	-3.387096	-4.501418
H	0.036493	-1.416443	-4.891960
H	1.459077	-2.371400	-3.879440
O	1.396860	-2.868578	-1.435898
C	2.175172	-3.464016	-2.218306
C	3.582178	-2.954872	-2.271726
H	3.911545	-2.876378	-3.310086
H	3.652166	-1.989425	-1.770682
H	4.238693	-3.677407	-1.770829
C	1.848501	-4.804119	-2.811149
H	0.826528	-4.799164	-3.185287
H	2.517647	-4.996282	-3.653143
C	2.016598	-5.883074	-1.719824
H	1.318293	-5.708392	-0.897912
H	1.807283	-6.866854	-2.146670
H	3.032445	-5.901789	-1.313006
H	-0.619344	-3.982486	-1.464135
C	3.393557	-1.479023	2.692199
C	2.480735	-1.686861	3.916568
C	3.822942	-2.841434	2.104905
H	4.304218	-0.972540	3.043206
C	3.141278	-2.582671	4.971479
H	1.539925	-2.147591	3.589275
H	2.216914	-0.713485	4.344845
C	4.476025	-3.740967	3.160796
H	2.936735	-3.341560	1.691722
H	4.505718	-2.673905	1.263082
C	3.559409	-3.932839	4.375621
H	2.460121	-2.733385	5.816847
H	4.030140	-2.075286	5.372307
H	4.736892	-4.711258	2.721546
H	5.419371	-3.283436	3.490537

H	4.056327	-4.548264	5.134438
H	2.659838	-4.482156	4.064260
C	3.520639	2.615127	-0.297254
C	3.941124	2.582264	-1.780422
C	2.618222	3.838379	-0.024529
H	4.434643	2.743869	0.299806
C	4.607379	3.894547	-2.211771
H	3.053350	2.407048	-2.402539
H	4.612251	1.732780	-1.951520
C	3.283432	5.149574	-0.457711
H	1.675094	3.713980	-0.573972
H	2.354258	3.868549	1.038734
C	3.701738	5.100838	-1.932451
H	4.869239	3.851679	-3.275237
H	5.550794	4.017815	-1.661553
H	2.605033	5.992298	-0.280139
H	4.171648	5.326653	0.164707
H	4.206850	6.030923	-2.217062
H	2.801533	5.027583	-2.558835
C	-5.132625	-0.441768	0.936640
C	-5.712912	-1.088329	-0.338413
C	-5.024536	-1.487657	2.065026
H	-5.847465	0.325087	1.268103
C	-7.056711	-1.775674	-0.068469
H	-4.997280	-1.829318	-0.719160
H	-5.817862	-0.326471	-1.119530
C	-6.368934	-2.174596	2.333905
H	-4.280090	-2.242917	1.781008
H	-4.646813	-1.006368	2.974056
C	-6.938434	-2.809129	1.058813
H	-7.428301	-2.249465	-0.984509
H	-7.799475	-1.016684	0.214867
H	-6.254606	-2.931225	3.118809
H	-7.083621	-1.432325	2.716591
H	-7.914223	-3.265311	1.262051
H	-6.273072	-3.621124	0.733353
C	-2.277922	3.756106	0.855559
C	-1.231494	4.039522	1.954559
C	-1.905151	4.502386	-0.442916
H	-3.239964	4.159437	1.202575
C	-1.033940	5.542097	2.186321
H	-0.274380	3.590527	1.658845
H	-1.533305	3.540491	2.882142
C	-1.708203	6.004314	-0.206649
H	-0.974635	4.077373	-0.842808

H	-2.677231	4.326269	-1.200579
C	-0.662971	6.264176	0.885117
H	-0.262266	5.705891	2.947528
H	-1.963149	5.973271	2.584730
H	-1.415895	6.496633	-1.141514
H	-2.665168	6.451593	0.096794
H	-0.554715	7.340501	1.062179
H	0.315130	5.901401	0.539684

C9-TS7Spri

C	0.005035	0.990674	-0.425794
C	0.044224	1.308577	-1.958096
H	-0.981318	1.481857	-2.283441
C	-1.298453	0.316605	-0.012800
C	-1.760527	0.489537	1.297170
C	-2.018125	-0.499739	-0.882579
C	-2.934731	-0.121836	1.735085
H	-1.191181	1.121704	1.970685
C	-3.192421	-1.144267	-0.466455
H	-1.667504	-0.651357	-1.897427
C	-3.639865	-0.938027	0.837633
H	-4.554767	-1.427817	1.160669
C	1.188693	0.134111	0.045238
C	1.192384	-1.250654	-0.145288
C	2.300976	0.740612	0.627373
C	2.310448	-2.015337	0.185427
H	0.314868	-1.746284	-0.547906
C	3.447143	0.006673	0.964908
H	2.255168	1.803688	0.809050
C	3.431278	-1.372009	0.724982
H	4.300565	-1.970776	0.968805
O	0.067475	2.278793	0.188925
B	0.622465	3.277189	-0.666504
N	0.770681	2.618567	-2.086380
C	0.768149	0.354403	-2.906651
H	0.656227	-0.691885	-2.618447
H	0.347930	0.484553	-3.905577
C	2.239711	0.811524	-2.881472
H	2.819613	0.194363	-2.191731
H	2.694994	0.730199	-3.871027
C	2.215534	2.271318	-2.386378
H	2.810855	2.385203	-1.480828
H	2.573935	2.982748	-3.128921
B	0.172900	3.462781	-3.360931
H	0.730013	4.540096	-3.365979
H	0.422446	2.812989	-4.354245
H	-1.035398	3.566655	-3.202127
O	-0.380535	4.516180	-0.824480
C	-1.618161	4.602963	-0.912257
C	-2.558106	3.485399	-0.600809
H	-2.558214	2.800619	-1.454996
H	-2.214241	2.915637	0.261075
H	-3.573417	3.859758	-0.459386
H	1.607298	3.842163	-0.274109
C	-2.183792	5.915816	-1.358414
H	-2.860202	5.672830	-2.190372
H	-2.843770	6.273958	-0.554249
C	-1.144594	6.952877	-1.766254
H	-0.499060	7.214024	-0.924162
H	-0.511514	6.556214	-2.562720
H	-1.638504	7.861508	-2.119537
C	4.662585	0.692328	1.594212
C	5.990064	-0.018493	1.257717
C	4.764647	2.186753	1.224763
H	4.537982	0.639565	2.688443
C	7.205499	0.672237	1.887944
H	6.105317	-0.046496	0.165321
H	5.963402	-1.058730	1.595248
C	5.983512	2.868250	1.857207
H	4.821897	2.274888	0.130698
H	3.861688	2.720978	1.532013
C	7.285461	2.149583	1.494634
H	8.121683	0.145029	1.597642
H	7.134487	0.595618	2.982208
H	6.022114	3.918044	1.544928
H	5.867146	2.871418	2.950243
H	8.140719	2.631453	1.982395
H	7.453833	2.228278	0.411468
C	2.313404	-3.509240	-0.065585
C	3.301304	-3.894934	-1.186244
C	2.600296	-4.328534	1.208146
H	1.306762	-3.786414	-0.410139
C	3.284254	-5.402533	-1.465266
H	4.313427	-3.589455	-0.888401
H	3.061326	-3.329678	-2.094094
C	2.579485	-5.835976	0.926686
H	3.586398	-4.049180	1.602128
H	1.870387	-4.068090	1.982758
C	3.561612	-6.209907	-0.190828
H	4.016135	-5.650847	-2.242599

H	2.298580	-5.684176	-1.861621
H	2.811078	-6.393701	1.841492
H	1.564148	-6.132463	0.627901
H	3.509525	-7.284434	-0.400980
H	4.586957	-6.005287	0.148065
C	-3.943950	-2.054252	-1.415382
C	-4.451579	-1.308462	-2.666441
C	-3.092049	-3.274054	-1.825642
H	-4.825499	-2.436446	-0.881410
C	-5.212068	-2.242843	-3.615368
H	-3.594374	-0.874156	-3.198067
H	-5.085072	-0.468506	-2.359296
C	-3.852920	-4.205850	-2.775920
H	-2.176934	-2.920525	-2.319873
H	-2.771230	-3.813321	-0.927231
C	-4.356212	-3.451899	-4.013208
H	-5.533399	-1.692183	-4.506846
H	-6.126712	-2.595817	-3.118641
H	-3.213188	-5.044838	-3.073011
H	-4.711523	-4.639534	-2.244290
H	-4.925853	-4.124634	-4.664429
H	-3.493474	-3.103885	-4.598309
C	-3.438983	0.105473	3.145058
C	-4.767523	0.890090	3.155646
C	-3.583584	-1.207628	3.939880
H	-2.692451	0.721270	3.666052
C	-5.268960	1.141047	4.582751
H	-5.522417	0.319347	2.598224
H	-4.635905	1.837088	2.619296
C	-4.084176	-0.953439	5.367044
H	-4.292468	-1.866912	3.421685
H	-2.622515	-1.733454	3.953998
C	-5.403287	-0.170641	5.366158
H	-6.227949	1.671508	4.557736
H	-4.559835	1.800055	5.103026
H	-4.204702	-1.904457	5.898422
H	-3.325804	-0.380719	5.918805
H	-5.728265	0.030663	6.393349
H	-6.187154	-0.786873	4.903681

C10-B5-TS1R

C	0.508045	0.728026	0.007831
C	0.094240	1.437448	-1.355900
H	-0.871841	1.044797	-1.671709
C	-0.419370	-0.457513	0.293208
C	-0.857895	-0.670902	1.601224
C	-0.844734	-1.329946	-0.711837
C	-1.715186	-1.729662	1.893959
H	-0.541233	0.015742	2.375226
C	-1.694949	-2.395727	-0.410783
H	-0.535228	-1.185649	-1.740596
C	-2.139057	-2.602163	0.892906
H	-2.796197	-3.430312	1.126715
C	1.968021	0.255665	0.044020
C	2.388738	-0.900041	-0.620513
C	2.915603	1.008358	0.737014
C	3.732253	-1.270128	-0.621475
H	1.678189	-1.525118	-1.146308
C	4.260662	0.636256	0.729443
H	2.589395	1.881014	1.286712
C	4.683018	-0.500822	0.047479
H	5.727542	-0.783936	0.038716
O	0.346530	1.710559	1.014595
B	-0.467957	2.792804	0.547211
N	-0.108675	2.881675	-0.997647
C	1.102370	1.455868	-2.503952
H	1.658403	0.522285	-2.598354
H	0.554611	1.615135	-3.434181
C	2.014933	2.666574	-2.208523
H	2.975858	2.347691	-1.798562
H	2.215172	3.232901	-3.120226
C	1.254013	3.517309	-1.169994
H	1.766978	3.510360	-0.207261
H	1.108968	4.551379	-1.476301
B	-1.169619	3.602900	-1.949263
H	-1.142837	4.789848	-1.732140
H	-0.983517	3.296962	-3.102872
H	-2.297334	3.158928	-1.638411
O	-1.920981	2.277390	0.549474
C	-2.938719	2.897662	0.082549
C	-4.078260	2.048801	-0.316177
C	-5.325220	2.600519	-0.649972
C	-3.906788	0.654699	-0.363392
C	-6.378140	1.771030	-1.023169
H	-5.476404	3.672954	-0.623703
C	-4.957165	-0.169645	-0.745592
C	-6.195223	0.387774	-1.075354
H	-4.804613	-1.241038	-0.800095
H	-7.016367	-0.255399	-1.376090
C	-3.134339	4.360191	0.366626

H	-3.700484	4.433522	1.304191
H	-2.178276	4.860260	0.488807
H	-3.696342	4.854463	-0.423560
H	-2.946798	0.235438	-0.099206
H	-7.339709	2.203551	-1.279146
C	-2.203465	-3.281315	-1.513719
F	-2.402222	-4.545992	-1.091608
F	-3.395740	-2.836583	-1.992069
F	-1.354542	-3.321576	-2.560968
C	-2.248069	-1.884492	3.293177
F	-2.597925	-3.161888	3.557653
F	-1.343324	-1.505537	4.218464
F	-3.351100	-1.123780	3.480450
C	4.143021	-2.546139	-1.304054
F	4.021916	-3.610595	-0.479685
F	3.374698	-2.798427	-2.387956
F	5.428230	-2.504587	-1.714052
C	5.258972	1.533545	1.407885
F	6.441788	0.917735	1.612375
F	5.504437	2.635863	0.659682
F	4.807728	1.966042	2.604233
H	-0.333067	3.816931	1.153425

C10-B5-TS1S

C	-0.304237	-0.453775	-0.092340
C	0.230106	-1.009701	-1.484003
H	0.888162	-0.263544	-1.925117
C	0.141163	0.998434	0.107630
C	0.561284	1.410048	1.374276
C	0.149388	1.924276	-0.936640
C	1.000477	2.715195	1.582331
H	0.567414	0.689313	2.181393
C	0.579359	3.235731	-0.720400
H	-0.170002	1.639369	-1.932831
C	1.013940	3.640411	0.537898
H	1.348625	4.656196	0.704817
C	-1.822883	-0.569822	0.063516
C	-2.699879	0.377892	-0.471700
C	-2.356981	-1.680717	0.715195
C	-4.079305	0.199596	-0.379048
H	-2.321032	1.270748	-0.952942
C	-3.738078	-1.860560	0.794379
H	-1.683459	-2.397795	1.165322
C	-4.611982	-0.926544	0.246577
H	-5.683058	-1.064741	0.316498
O	0.301786	-1.272265	0.898109
B	1.446299	-1.934334	0.363491
N	1.088034	-2.189269	-1.135132
C	-0.779123	-1.536016	-2.504599
H	-1.702863	-0.955245	-2.524810
H	-0.320594	-1.475811	-3.492930
C	-1.009653	-3.012252	-2.117189
H	-1.952527	-3.139000	-1.579864
H	-1.048566	-3.645306	-3.005706
C	0.173379	-3.394587	-1.204673
H	-0.173510	-3.640972	-0.199254
H	0.760271	-4.230528	-1.580820
B	2.295242	-2.387244	-2.206897
H	2.976179	-3.301744	-1.801009
H	1.787912	-2.622352	-3.282906
H	2.918065	-1.335364	-2.258480
O	2.565909	-0.789261	0.309144
C	3.735632	-0.636233	-0.123818
C	4.769012	-1.669446	-0.076990
C	5.911313	-1.554923	-0.889324
C	4.654203	-2.755760	0.806566
C	6.903199	-2.525213	-0.839639
H	6.002910	-0.728906	-1.584646
C	5.661707	-3.710238	0.869867
H	3.783731	-2.835597	1.442647
C	6.781042	-3.602253	0.042189
H	7.770640	-2.445301	-1.486011
H	5.572535	-4.542175	1.560248
H	7.560520	-4.356487	0.084799
C	4.067820	0.752354	-0.593119
H	4.111348	0.747751	-1.687208
H	3.277133	1.434449	-0.281639
H	5.035268	1.085789	-0.212357
C	0.648294	4.184308	-1.885099
F	0.608747	5.473376	-1.491459
F	1.796328	4.015665	-2.584272
F	-0.369780	3.984887	-2.750132
C	1.551054	3.101856	2.927596
F	1.450341	4.429087	3.152053
F	0.918417	2.464574	3.933466
F	2.865353	2.782880	3.022097
C	-4.995589	1.202656	-1.024972
F	-4.479835	2.450560	-0.982000
F	-5.204284	0.908168	-2.329379
F	-6.205262	1.236993	-0.427895

C	-4.257725	-3.127646	1.414075
F	-3.627937	-3.412157	2.573964
F	-5.580906	-3.069711	1.668111
F	-4.051648	-4.183233	0.588710
H	1.813386	-2.882186	0.986616

C10-B5-TS1Spri

C	-0.030680	-0.227902	-0.086572
C	0.614940	-0.558875	-1.475036
H	1.265534	0.278240	-1.732408
C	-0.221409	1.273894	0.107359
C	-0.094522	1.802890	1.395267
C	-0.537918	2.130275	-0.947449
C	-0.277405	3.165466	1.616658
H	0.163599	1.140014	2.211341
C	-0.733130	3.494286	-0.716291
H	-0.624219	1.754843	-1.960296
C	-0.603704	4.021774	0.564319
H	-0.749936	5.079632	0.740481
C	-1.370690	-0.945886	0.134571
C	-2.562422	-0.470583	-0.422010
C	-1.401989	-2.130042	0.869267
C	-3.743271	-1.199359	-0.296323
H	-2.586091	0.476950	-0.946292
C	-2.584951	-2.861811	0.982174
H	-0.496661	-2.484897	1.343610
C	-3.762695	-2.410606	0.395344
H	-4.678695	-2.979295	0.487040
O	0.920949	-0.680477	0.855154
B	1.775695	-1.735772	0.351391
N	1.498677	-1.749756	-1.229820
C	-0.277460	-0.948581	-2.652780
H	-1.224289	-0.405738	-2.672727
H	0.259125	-0.720007	-3.575648
C	-0.479481	-2.468519	-2.513569
H	-1.419956	-2.687985	-2.004041
H	-0.514985	-2.953482	-3.491307
C	0.710031	-2.971187	-1.672764
H	0.374455	-3.522170	-0.794580
H	1.385595	-3.611842	-2.238078
B	2.819204	-1.639129	-2.133635
H	3.492557	-2.627823	-1.983302
H	2.510608	-1.384346	-3.274037
H	3.470239	-0.664324	-1.687703
O	3.256287	-1.448432	0.575774
C	3.962847	-0.501249	0.079644
C	5.396897	-0.798111	-0.092362
C	6.335569	0.218781	-0.326885
C	5.828503	-2.132557	-0.010646
C	7.683508	-0.094143	-0.471552
H	6.020699	1.253380	-0.393220
C	7.175019	-2.440147	-0.158580
C	8.104556	-1.422524	-0.388775
H	7.502659	-3.472741	-0.098022
H	9.156306	-1.664874	-0.505288
C	3.490295	0.926718	0.137569
H	2.408014	0.976038	0.172069
H	3.870325	1.354492	1.074179
H	3.875050	1.516804	-0.693201
H	5.092375	-2.907403	0.160627
H	8.404825	0.696551	-0.649545
C	-1.140724	4.376536	-1.864705
F	-0.869909	5.675886	-1.625706
F	-0.504094	4.026611	-3.005294
F	-2.466522	4.280414	-2.110727
C	-0.050567	3.726755	2.994665
F	-0.734969	4.873791	3.188795
F	-0.422852	2.856959	3.956060
F	1.258507	4.005527	3.198505
C	-4.987273	-0.701556	-0.979972
F	-5.055287	0.647425	-0.970243
F	-5.019057	-1.086913	-2.277515
F	-6.108003	-1.174873	-0.397311
C	-2.534301	-4.174914	1.713298
F	-2.033443	-4.031367	2.958524
F	-3.749718	-4.748717	1.823269
F	-1.728405	-5.051627	1.066689
H	1.618098	-2.816937	0.854302

C10-TS7R

C	0.195970	0.748400	-0.096005
C	-0.000116	1.380842	-1.542829
H	-0.967515	1.064730	-1.929820
C	-0.910140	-0.272519	0.183462
C	-1.496738	-0.309142	1.449153
C	-1.368071	-1.154328	-0.796185
C	-2.534525	-1.198370	1.717953
H	-1.156967	0.388686	2.203187

C	-2.403154	-2.049016	-0.518654
H	-0.943250	-1.145110	-1.793857
C	-2.997710	-2.076556	0.738472
H	-3.808770	-2.761510	0.949762
C	1.570970	0.103046	0.113030
C	1.872455	-1.171773	-0.374812
C	2.567037	0.814192	0.781365
C	3.151600	-1.704756	-0.225936
H	1.113065	-1.770985	-0.861238
C	3.847562	0.278270	0.923442
H	2.327961	1.782849	1.199463
C	4.154365	-0.981138	0.417389
H	5.146479	-1.397105	0.534890
O	0.082234	1.832328	0.811792
B	-0.544295	2.954285	0.190649
N	-0.081286	2.864005	-1.319738
C	1.109785	1.193077	-2.576474
H	1.570924	0.205603	-2.528081
H	0.673882	1.312109	-3.569530
C	2.112125	2.333031	-2.294266
H	2.987506	1.966258	-1.753537
H	2.462700	2.781296	-3.225975
C	1.347620	3.355498	-1.428492
H	1.775627	3.416127	-0.426575
H	1.326331	4.356404	-1.854901
B	-0.986980	3.584378	-2.440031
H	-0.851854	4.779409	-2.322843
H	-0.680906	3.169646	-3.532785
H	-2.169329	3.268614	-2.224634
O	-2.070674	2.617902	0.119913
C	-2.966235	3.348627	-0.387228
C	-2.931389	4.843762	-0.299688
H	-1.923152	5.238451	-0.376001
H	-3.563442	5.288955	-1.068221
H	-3.343723	5.105641	0.684742
H	-0.348959	4.005741	0.727299
C	-4.271684	2.692868	-0.742060
H	-4.579216	3.090389	-1.714918
H	-5.007609	3.086776	-0.023831
C	-4.260494	1.168333	-0.727213
H	-3.527967	0.771598	-1.433114
H	-4.017289	0.784914	0.264983
H	-5.243729	0.786064	-1.011864
C	-2.949888	-2.895152	-1.633585
F	-3.604217	-3.980996	-1.175908
F	-3.825984	-2.187589	-2.388204
F	-1.972729	-3.320065	-2.463973
C	-3.249666	-1.112230	3.036861
F	-3.856981	-2.271874	3.363246
F	-2.418029	-0.783300	4.045409
F	-4.214666	-0.157813	2.992977
C	3.461297	-3.047832	-0.828611
F	2.394243	-3.874916	-0.778724
F	3.807479	-2.931540	-2.131979
F	4.485633	-3.659074	-0.198514
C	4.909958	1.122597	1.571309
F	5.998996	0.402785	1.910372
F	5.321405	2.104349	0.732899
F	4.453350	1.729241	2.687558

C10-TS7Rpri

C	0.058894	0.675495	-0.277253
C	0.082742	1.187748	-1.758436
H	-0.953276	1.253046	-2.092927
C	-1.146476	-0.221856	-0.013183
C	-1.747670	-0.185358	1.247880
C	-1.665320	-1.079239	-0.984246
C	-2.862265	-0.974614	1.518631
H	-1.349054	0.481095	2.001140
C	-2.767639	-1.888404	-0.697617
H	-1.233400	-1.118151	-1.977239
C	-3.377983	-1.837970	0.551280
H	-4.241836	-2.453872	0.766295
C	1.347881	-0.054293	0.124540
C	1.572912	-1.391067	-0.219514
C	2.348029	0.637882	0.805698
C	2.794253	-1.999302	0.062188
C	3.573074	0.026592	1.076676
H	2.172486	1.659417	1.115619
C	3.811867	-1.291459	0.701915
O	-0.085391	1.854644	0.495919
B	0.404132	3.031627	-0.178959
N	0.612073	2.592677	-1.691743
C	0.948641	0.470670	-2.793597
H	0.989299	-0.610311	-2.645674
H	0.521884	0.659786	-3.780203
C	2.334686	1.128622	-2.675180
H	2.982294	0.546173	-2.016410
H	2.827471	1.192205	-3.647549

C	2.080976	2.524313	-2.074092
H	2.695217	2.694655	-1.189796
H	2.270555	3.329182	-2.782649
B	-0.165613	3.501857	-2.801384
H	0.267444	4.627706	-2.732755
H	-0.000224	2.976920	-3.879613
H	-1.365514	3.474811	-2.511011
O	-0.639353	4.186046	-0.165707
C	-1.845353	4.188134	-0.519520
C	-2.430476	5.509028	-0.898453
H	-3.212731	5.781108	-0.179935
H	-1.657271	6.274853	-0.927603
H	-2.903989	5.418011	-1.879333
H	1.369915	3.554755	0.305608
C	-2.772255	3.026771	-0.308989
H	-3.652383	3.154098	-0.943617
H	-2.283300	2.092962	-0.568460
C	-3.175287	2.982273	1.182184
H	-3.703469	3.889720	1.489139
H	-3.831849	2.129100	1.360261
H	-2.286175	2.865055	1.804268
H	4.762058	-1.762883	0.915721
H	0.795999	-1.975139	-0.697508
C	-3.257968	-2.853223	-1.743359
F	-4.544046	-3.205569	-1.540892
F	-3.165919	-2.324799	-2.983904
F	-2.525002	-3.988682	-1.743811
C	-3.572004	-0.825977	2.835946
F	-4.209060	-1.957496	3.198038
F	-2.727593	-0.488081	3.830210
F	-4.514748	0.151486	2.769585
C	3.032097	-3.412323	-0.396142
F	1.905256	-4.154169	-0.327440
F	3.446212	-3.445154	-1.684337
F	3.977540	-4.029886	0.341552
C	4.637518	0.848859	1.749643
F	4.206805	1.348297	2.927949
F	5.758418	0.139131	1.990592
F	4.983633	1.907837	0.979621

C10-TS7S

C	0.086907	0.712298	-0.044950
C	-0.197593	1.451278	-1.425083
H	-1.123938	1.061163	-1.843857
C	-0.889693	-0.450120	0.160553
C	-1.364827	-0.710390	1.448063
C	-1.303695	-1.271952	-0.888181
C	-2.245235	-1.765092	1.675088
H	-1.050756	-0.068130	2.260581
C	-2.182097	-2.332955	-0.654147
H	-0.951873	-1.102039	-1.899617
C	-2.662029	-2.586204	0.626844
H	-3.338467	-3.411557	0.808086
C	1.525814	0.203194	0.091585
C	1.949828	-0.980194	-0.520041
C	2.453218	0.950327	0.816317
C	3.280152	-1.386060	-0.433535
H	1.247385	-1.605988	-1.056543
C	3.785383	0.542102	0.895611
H	2.121590	1.845301	1.325668
C	4.212945	-0.624165	0.268681
H	5.245188	-0.941695	0.337659
O	-0.127649	1.690943	0.961793
B	-0.905961	2.769007	0.446383
N	-0.454178	2.885317	-1.057669
C	0.917239	1.502735	-2.469170
H	1.493216	0.577674	-2.520381
H	0.460290	1.669111	-3.445833
C	1.778669	2.720364	-2.070211
H	2.693335	2.408584	-1.560817
H	2.070355	3.295117	-2.951332
C	0.901894	3.557304	-1.115730
H	1.328958	3.580402	-0.111677
H	0.752039	4.582011	-1.450294
B	-1.446162	3.607492	-2.107210
H	-1.523947	4.773665	-1.790872
H	-1.010284	3.453605	-3.224600
H	-2.546902	3.055262	-2.026477
O	-2.372973	2.199980	0.329838
C	-3.408528	2.789088	-0.070343
C	-4.540114	1.910861	-0.503203
H	-5.383390	2.036848	0.186253
H	-4.884449	2.210306	-1.495422
H	-4.220846	0.869124	-0.515139
H	-0.872337	3.777990	1.086659
C	-3.633774	4.257127	0.140238
H	-4.054445	4.307857	1.158904
H	-2.672751	4.766371	0.169381
C	-4.583827	4.928356	-0.851383

H	-5.580467	4.478563	-0.841336
H	-4.694031	5.984257	-0.593848
H	-4.173983	4.864179	-1.861495
C	-2.807936	-1.973395	3.054892
F	-3.184050	-3.254062	3.257068
F	-1.917576	-1.649318	4.014765
F	-3.903877	-1.202503	3.256251
C	-2.661545	-3.154837	-1.818601
F	-3.089662	-4.374642	-1.435747
F	-3.694535	-2.549540	-2.451953
F	-1.687095	-3.328966	-2.738346
C	3.718844	-2.625166	-1.165068
F	2.744464	-3.560495	-1.191128
F	4.032599	-2.348621	-2.452390
F	4.810159	-3.183352	-0.601488
C	4.764534	1.429739	1.612828
F	4.271963	1.865392	2.791948
F	5.934224	0.805858	1.861027
F	5.044290	2.531301	0.874970

C10-TS7Spri

C	-0.000940	0.603313	-0.168953
C	-0.007019	1.230612	-1.605391
H	-1.049852	1.343313	-1.904486
C	-1.210986	-0.297645	0.060684
C	-1.779154	-0.343845	1.336613
C	-1.759669	-1.084386	-0.953065
C	-2.882144	-1.156797	1.586266
H	-1.361074	0.275835	2.119715
C	-2.855563	-1.910122	-0.691756
H	-1.355301	-1.055440	-1.958016
C	-3.426241	-1.950832	0.576686
H	-4.281431	-2.583895	0.774783
C	1.286965	-0.174361	0.138935
C	1.486116	-1.480367	-0.320149
C	2.310334	0.445578	0.854042
C	2.705080	-2.124173	-0.118615
H	0.690918	-2.013994	-0.826078
C	3.533018	-0.199990	1.044009
H	2.154209	1.438552	1.254234
C	3.746183	-1.484201	0.554073
H	4.694639	-1.982657	0.705248
O	-0.106882	1.718097	0.697022
B	0.380524	2.941631	0.110340
N	0.559416	2.614246	-1.441186
C	0.814907	0.580428	-2.717580
H	0.832376	-0.509389	-2.657197
H	0.368543	0.856677	-3.674470
C	2.220303	1.194498	-2.585385
H	2.876338	0.537119	-2.011041
H	2.679782	1.342828	-3.564749
C	2.023412	2.529866	-1.842471
H	2.647235	2.584685	-0.950541
H	2.242013	3.396225	-2.464775
B	-0.191177	3.626231	-2.460164
H	0.328236	4.711401	-2.367980
H	-0.146211	3.151678	-3.571757
H	-1.378859	3.686665	-2.103516
O	-0.648055	4.092297	0.234639
C	-1.833246	4.151755	-0.192289
C	-2.792590	3.001437	-0.125105
H	-2.310390	2.039621	-0.250722
H	-3.224567	3.015911	0.885083
H	-3.601810	3.131124	-0.844248
H	1.365360	3.412926	0.610126
C	-2.406778	5.520291	-0.428193
H	-2.983166	5.472733	-1.358026
H	-3.152855	5.672994	0.367842
C	-1.383731	6.649988	-0.454487
H	-0.838876	6.709209	0.490180
H	-0.658429	6.483160	-1.252834
H	-1.886925	7.604769	-0.626342
C	-3.379468	-2.797247	-1.788483
F	-4.658860	-3.165131	-1.572439
F	-3.325768	-2.179977	-2.990102
F	-2.648010	-3.928820	-1.895116
C	-3.531467	-1.113478	2.943214
F	-4.258391	-2.222397	3.191198
F	-2.618918	-0.988928	3.928919
F	-4.370043	-0.054685	3.046479
C	2.914377	-3.495851	-0.699900
F	1.778554	-4.226394	-0.673986
F	3.305668	-3.423439	-1.993782
F	3.863722	-4.186214	-0.035455
C	4.624031	0.552503	1.754802
F	4.222495	0.976000	2.972204
F	5.734638	-0.192830	1.925310
F	4.978533	1.656584	1.054405

C11-B5-TS1R

C	-0.654650	-0.409232	0.146949
C	-0.010772	-1.374929	-0.943211
H	0.931988	-0.957833	-1.276304
C	-0.021154	1.002312	0.166531
C	-0.185144	1.772719	1.327305
C	0.738729	1.585777	-0.849329
C	0.442798	3.001884	1.505433
C	1.388665	2.808123	-0.687592
C	1.252368	3.517088	0.498069
C	-2.181098	-0.235755	0.012941
C	-2.704972	0.573767	-1.001902
C	-3.123638	-0.806419	0.879396
C	-4.062874	0.808910	-1.174044
C	-4.494206	-0.584395	0.725910
C	-4.969903	0.230099	-0.293197
O	-0.363689	-1.006740	1.390078
B	0.566994	-2.080993	1.266199
N	0.309276	-2.633176	-0.191454
C	-0.881332	-1.834839	-2.111945
H	-1.522626	-1.046275	-2.498990
H	-0.219085	-2.139542	-2.922999
C	-1.656030	-3.060894	-1.571343
H	-2.710378	-2.827456	-1.402872
H	-1.611431	-3.887842	-2.282662
C	-0.971731	-3.431656	-0.237332
H	-1.589299	-3.152496	0.616402
H	-0.720258	-4.487447	-0.155088
B	1.487018	-3.474680	-0.872265
H	1.588169	-4.526913	-0.287930
H	1.299780	-3.584273	-2.060285
H	2.536398	-2.817958	-0.729982
O	1.968881	-1.410710	1.154530
C	3.075634	-1.987465	0.893922
C	4.103862	-1.136399	0.261831
C	5.388510	-1.614236	-0.037819
C	3.769220	0.188018	-0.063832
C	6.312606	-0.780800	-0.661271
C	4.689097	1.015552	-0.693979
C	5.964178	0.529932	-0.995577
C	3.446367	-3.276583	1.567474
H	3.990405	-3.013255	2.484039
H	2.557253	-3.842764	1.829205
H	4.091239	-3.891741	0.942700
F	-0.985720	1.359272	2.314443
F	0.268021	3.691204	2.637788
F	1.878123	4.684913	0.662474
F	2.171440	3.286749	-1.667203
F	0.893690	0.995703	-2.048605
F	-5.356561	-1.156178	1.572929
F	-6.279786	0.440854	-0.436503
F	-4.494773	1.585487	-2.173420
F	-1.877577	1.147496	-1.899854
F	-2.793532	-1.628448	1.881861
H	2.784970	0.550179	0.194739
H	4.404696	2.027781	-0.961542
H	6.685707	1.171120	-1.492103
H	7.303302	-1.156154	-0.895458
H	5.664017	-2.635360	0.197720
H	0.521517	-2.898525	2.139530

C11-B5-TS1Rpri

C	0.499873	-0.153786	0.046055
C	-0.302156	-0.604683	1.319239
H	-0.943607	0.221963	1.608938
C	0.772691	1.361722	-0.024849
C	1.156326	1.898537	-1.261494
C	0.667008	2.265325	1.032923
C	1.363181	3.261458	-1.450134
C	0.865053	3.634706	0.868271
C	1.209888	4.137915	-0.380056
C	1.865573	-0.873293	-0.110334
C	2.964112	-0.480406	0.665592
C	2.117168	-1.901104	-1.027182
C	4.218638	-1.070630	0.575425
C	3.368192	-2.511345	-1.136638
C	4.427327	-2.094822	-0.341491
O	-0.374594	-0.432534	-1.019043
B	-1.362694	-1.453364	-0.733261
N	-1.200685	-1.705798	0.836251
C	0.430742	-1.185620	2.527284
H	1.384608	-0.699525	2.715856
H	-0.196422	-1.022098	3.404727
C	0.552038	-2.697625	2.240169
H	1.563358	-2.959431	1.918647
H	0.333338	-3.282804	3.135679
C	-0.455027	-2.994012	1.109705
H	0.047299	-3.309653	0.195728

H	-1.187907	-3.756518	1.370898
B	-2.572902	-1.692147	1.657366
H	-3.275117	-2.597933	1.283122
H	-2.352512	-1.654342	2.844133
H	-3.144645	-0.616628	1.345094
O	-2.798355	-0.981765	-0.997304
C	-3.472761	-0.092082	-0.371616
C	-4.934005	-0.298723	-0.335721
C	-5.811406	0.725754	0.050861
C	-5.451340	-1.551512	-0.703033
C	-7.184666	0.501768	0.064251
H	-5.428328	1.697165	0.340669
C	-6.823208	-1.770690	-0.687066
C	-7.691969	-0.745527	-0.304037
H	-7.218067	-2.741039	-0.969715
H	-8.763515	-0.919147	-0.290791
C	-2.904605	1.286615	-0.154416
H	-1.821350	1.263587	-0.202019
H	-3.260377	1.914495	-0.980834
H	-3.241159	1.728057	0.783230
H	-4.761090	-2.334851	-0.989114
H	-7.858537	1.298267	0.362227
F	2.842504	0.508959	1.576167
F	5.220534	-0.650337	1.355043
F	5.623978	-2.675270	-0.446345
F	3.549414	-3.498770	-2.019436
F	1.170037	-2.399212	-1.827721
F	0.386257	1.854416	2.283434
F	0.738162	4.461461	1.912483
F	1.403494	5.447649	-0.548883
F	1.719263	3.730860	-2.650449
F	1.367861	1.100610	-2.313482
H	-1.283151	-2.457955	-1.377541

C11-B5-TS1S

C	-0.501393	-0.155512	-0.140614
C	0.248527	-0.459132	-1.506130
H	0.669854	0.464817	-1.883857
C	-0.855400	1.333822	0.051534
C	-1.130873	1.780619	1.351553
C	-0.937473	2.296372	-0.956299
C	-1.393852	3.115497	1.643059
C	-1.195648	3.639623	-0.688938
C	-1.419057	4.055063	0.617520
C	-1.790998	-0.976749	0.070565
C	-2.957388	-0.641771	-0.627728
C	-1.910385	-2.048434	0.966193
C	-4.162346	-1.316981	-0.479641
C	-3.110887	-2.742257	1.131735
C	-4.244135	-2.375424	0.418101
O	0.432559	-0.485416	0.867986
B	1.683729	-0.891253	0.342424
N	1.400652	-1.331357	-1.119522
C	-0.479073	-1.250841	-2.590977
H	-1.522739	-0.964634	-2.701245
H	0.019389	-1.044023	-3.538967
C	-0.273163	-2.734419	-2.201343
H	-1.188196	-3.171983	-1.793762
H	0.015952	-3.324459	-3.072588
C	0.841240	-2.733829	-1.131385
H	0.444715	-2.974337	-0.144232
H	1.658605	-3.417766	-1.352939
B	2.631438	-1.264307	-2.190270
H	3.556650	-1.848516	-1.669403
H	2.266656	-1.815710	-3.205774
H	2.874798	-0.091049	-2.409227
O	2.501931	0.513716	0.151430
C	3.692355	0.766933	-0.144116
C	4.829359	-0.060459	0.274513
C	5.992392	-0.112912	-0.512238
C	4.788350	-0.742270	1.501715
C	7.080852	-0.868385	-0.094948
H	6.023606	0.397259	-1.467761
C	5.892414	-1.472690	1.927021
H	3.903231	-0.676682	2.120515
C	7.033536	-1.545600	1.126315
H	7.965542	-0.931443	-0.719515
H	5.861499	-1.987822	2.881222
H	7.888715	-2.127883	1.454738
C	3.941149	2.048325	-0.881030
H	4.245285	1.802557	-1.903431
H	3.018175	2.625616	-0.926543
H	4.743143	2.624923	-0.413657
F	-1.182353	0.919695	2.374634
F	-1.637779	3.496059	2.902530
F	-1.669721	5.339383	0.884581
F	-1.247495	4.525225	-1.691461
F	-0.798337	1.973026	-2.255024
F	-2.944611	0.367608	-1.522062

F	-5.235673	-0.949123	-1.187498
F	-5.390976	-3.038698	0.579400
F	-3.172175	-3.764620	1.991870
F	-0.885681	-2.513999	1.691021
H	2.313387	-1.650493	1.008459

C11-B5-TS1Spri

C	0.499893	-0.153840	0.046038
C	-0.302111	-0.604762	1.319235
H	-0.943526	0.221887	1.609003
C	0.772630	1.361688	-0.024861
C	1.156170	1.898539	-1.261520
C	0.666995	2.265270	1.032935
C	1.362942	3.261475	-1.450153
C	0.864960	3.634664	0.868291
C	1.209687	4.137908	-0.380052
C	1.865643	-0.873254	-0.110357
C	2.964169	-0.480266	0.665536
C	2.117273	-1.901130	-1.027123
C	4.218732	-1.070411	0.575373
C	3.368339	-2.511286	-1.136580
C	4.427467	-2.094646	-0.341485
O	-0.374541	-0.432649	-1.019063
B	-1.362717	-1.453399	-0.733258
N	-1.200684	-1.705850	0.836263
C	0.430835	-1.185756	2.527217
H	1.384622	-0.699542	2.715894
H	-0.196394	-1.022470	3.404660
C	0.552366	-2.697676	2.239851
H	1.563597	-2.959160	1.917798
H	0.334273	-3.283058	3.135378
C	-0.455180	-2.994155	1.109833
H	0.046715	-3.310209	0.195762
H	-1.188138	-3.756398	1.371574
B	-2.572931	-1.692084	1.657283
H	-3.275193	-2.597841	1.283079
H	-2.352649	-1.654101	2.844064
H	-3.144618	-0.616565	1.344831
O	-2.798331	-0.981721	-0.997268
C	-3.472747	-0.092096	-0.371479
C	-4.933998	-0.298779	-0.335611
C	-5.811435	0.725633	0.051059
C	-5.451295	-1.551539	-0.703065
C	-7.184690	0.501610	0.064397
H	-5.428391	1.697022	0.340988
C	-6.823160	-1.770754	-0.687155
C	-7.691956	-0.745656	-0.304036
H	-7.217986	-2.741081	-0.969924
H	-8.763498	-0.919304	-0.290835
C	-2.904661	1.286671	-0.154504
H	-1.821402	1.263670	-0.202048
H	-3.260409	1.914364	-0.981074
H	-3.241265	1.728302	0.783035
H	-4.761020	-2.334823	-0.989233
H	-7.858587	1.298063	0.362441
F	2.842499	0.509090	1.576112
F	5.220613	-0.650021	1.354958
F	5.624154	-2.675017	-0.446337
F	3.549603	-3.498753	-2.019323
F	1.170160	-2.399355	-1.827606
F	0.386344	1.854328	2.283458
F	0.738104	4.461396	1.912526
F	1.403219	5.447654	-0.548871
F	1.718929	3.730911	-2.650484
F	1.367698	1.100641	-2.313529
H	-1.283242	-2.458005	-1.377532

C11-TS7R

C	-0.136251	-0.424548	0.063083
C	0.354340	-1.412868	-1.083284
H	1.275763	-1.036437	-1.507332
C	0.551653	0.959197	0.039747
C	0.472950	1.738528	1.203562
C	1.262853	1.522126	-1.020234
C	1.127406	2.959171	1.334303
C	1.934307	2.738427	-0.912041
C	1.871343	3.462012	0.271319
C	-1.658402	-0.190208	0.074310
C	-2.237237	0.641344	-0.891255
C	-2.540044	-0.728404	1.020657
C	-3.595226	0.927743	-0.942415
C	-3.909287	-0.454826	0.989346
C	-4.441740	0.379988	0.015503
O	0.253094	-1.043028	1.274168
B	1.086692	-2.166592	1.049415
N	0.696331	-2.684925	-0.368009
C	-0.639745	-1.836564	-2.163626
H	-1.278436	-1.022215	-2.498581

H	-0.065139	-2.175433	-3.026270
C	-1.413900	-3.024977	-1.546166
H	-2.429012	-2.736808	-1.261277
H	-1.492231	-3.846071	-2.260806
C	-0.607697	-3.444104	-0.297143
H	-1.131281	-3.171501	0.620070
H	-0.376172	-4.507265	-0.265062
B	1.779083	-3.584202	-1.184223
H	1.997216	-4.558897	-0.497773
H	1.293508	-3.884753	-2.250814
H	2.784235	-2.905796	-1.358916
O	2.551710	-1.537929	0.808230
C	3.655201	-2.109947	0.706893
C	3.944203	-3.462868	1.270850
H	3.041545	-3.972515	1.592334
H	4.459201	-4.069511	0.523959
H	4.624750	-3.326854	2.121526
H	1.126163	-2.970879	1.931662
C	4.785663	-1.327778	0.103090
H	5.214851	-1.963957	-0.680642
H	5.567876	-1.251263	0.872168
C	4.397951	0.043374	-0.441396
H	3.679003	-0.049309	-1.258080
H	3.946314	0.663737	0.335804
H	5.282638	0.558020	-0.823606
F	2.632205	3.211241	-1.951801
F	1.351649	0.916709	-2.220470
F	2.507679	4.630332	0.384857
F	1.030844	3.658588	2.470467
F	-0.268899	1.339883	2.243235
F	-1.468931	1.184263	-1.858111
F	-4.084890	1.722653	-1.899665
F	-5.750280	0.640216	-0.011393
F	-4.714358	-0.995828	1.909794
F	-2.149737	-1.564178	1.989970

C11-TS7Rpri

C	0.022500	-0.377922	0.058321
C	-0.286176	-1.297260	1.296650
H	-1.282010	-1.050612	1.651190
C	-0.721690	0.972425	0.079728
C	-0.849707	1.675072	-1.125799
C	-1.303759	1.565654	1.201262
C	-1.572854	2.859818	-1.225985
C	-2.035568	2.748991	1.126486
C	-2.175720	3.399158	-0.093803
C	1.531616	-0.081332	-0.129731
C	2.170413	0.874129	0.671559
C	2.343507	-0.697724	-1.090103
C	3.517442	1.194849	0.558093
C	3.699457	-0.392781	-1.224282
C	4.292084	0.559575	-0.406111
O	-0.511126	-1.089065	-1.033585
B	-0.647519	-2.506759	-0.807021
N	-0.356112	-2.690684	0.741450
C	0.697365	-1.373945	2.462534
H	1.162392	-0.416767	2.686548
H	0.135331	-1.679037	3.346290
C	1.702358	-2.475525	2.071543
H	2.632293	-2.042857	1.693455
H	1.958916	-3.095383	2.933016
C	1.013627	-3.299200	0.963997
H	1.574301	-3.259755	0.030051
H	0.871260	-4.345681	1.230090
B	-1.465290	-3.544896	1.561147
H	-1.511469	-4.655942	1.089975
H	-1.182217	-3.511952	2.736543
H	-2.553753	-2.968910	1.392064
O	-2.099192	-3.002256	-1.073610
C	-3.182590	-2.633038	-0.549449
C	-4.299406	-3.627261	-0.558527
H	-4.988495	-3.367673	-1.373114
H	-3.908904	-4.631573	-0.714216
H	-4.859203	-3.574262	0.376955
H	-0.002388	-3.226034	-1.508567
C	-3.506156	-1.191753	-0.264074
H	-4.417370	-1.145549	0.336769
H	-2.703484	-0.725959	0.298254
C	-3.679763	-0.426867	-1.593427
H	-4.457600	-0.869452	-2.222679
H	-3.968374	0.604799	-1.377135
H	-2.736807	-0.418194	-2.142084
F	-1.175011	1.026423	2.426779
F	-2.586381	3.268121	2.228987
F	-2.871836	4.534568	-0.176921
F	-1.681427	3.486132	-2.402335
F	-0.264857	1.228437	-2.243034
F	1.487100	1.529117	1.633575
F	4.063186	2.112454	1.362904

F	5.586968	0.852950	-0.534192
F	4.430812	-1.018650	-2.151659
F	1.890898	-1.646880	-1.915206

C11-TS7S

C	0.042148	-0.306156	-0.049658
C	-0.725125	-1.062482	1.121294
H	-1.483295	-0.404848	1.529215
C	-0.169722	1.223937	-0.037517
C	0.101634	1.930006	-1.218197
C	-0.607076	1.989725	1.044727
C	-0.124341	3.297609	-1.340321
C	-0.845041	3.359348	0.946008
C	-0.609201	4.018285	-0.253790
C	1.562339	-0.568628	-0.082058
C	2.400163	0.054003	0.851033
C	2.207513	-1.373952	-1.030939
C	3.780027	-0.103598	0.870653
C	3.593416	-1.547372	-1.030893
C	4.386884	-0.906913	-0.088387
O	-0.535444	-0.791397	-1.242737
B	-1.679569	-1.594827	-0.997446
N	-1.446009	-2.188104	0.438918
C	0.101950	-1.738586	2.213749
H	0.966024	-1.150741	2.514428
H	-0.533152	-1.853407	3.092549
C	0.462209	-3.127912	1.634839
H	1.517656	-3.185528	1.357021
H	0.271449	-3.911752	2.370101
C	-0.428712	-3.304062	0.385705
H	0.151793	-3.209904	-0.532224
H	-0.964211	-4.251370	0.363195
B	-2.730499	-2.696585	1.273531
H	-3.175183	-3.663369	0.696674
H	-2.400210	-2.942050	2.409273
H	-3.560293	-1.781766	1.293510
O	-2.864486	-0.563659	-0.762758
C	-4.068095	-0.836870	-0.520310
C	-4.883934	0.255948	0.099134
H	-4.238860	1.085450	0.386889
H	-5.634768	0.600251	-0.622323
H	-5.419811	-0.123122	0.971375
H	-1.951742	-2.379158	-1.857554
C	-4.754994	-2.042801	-1.093856
H	-5.061705	-1.708076	-2.099259
H	-4.025564	-2.836964	-1.235722
C	-5.970998	-2.543812	-0.315560
H	-5.669534	-2.856509	0.686399
H	-6.753206	-1.784220	-0.231708
H	-6.401875	-3.407618	-0.827211
F	-0.799187	1.448792	2.262815
F	-1.280702	4.042770	2.011517
F	-0.830845	5.330941	-0.358613
F	0.137618	3.924383	-2.492821
F	0.625744	1.310096	-2.281416
F	1.872470	0.830925	1.819008
F	4.518750	0.513169	1.799064
F	5.710814	-1.073607	-0.091349
F	4.162579	-2.332087	-1.951980
F	1.552989	-2.063756	-1.972610

C11-TS7Spri

C	-0.112267	-0.200622	0.030824
C	-0.868510	-0.786214	1.277285
H	-1.630551	-0.068187	1.564031
C	-0.066272	1.339162	-0.014172
C	0.251065	1.949379	-1.235524
C	-0.315207	2.197705	1.057494
C	0.259055	3.331252	-1.397917
C	-0.316708	3.584212	0.918936
C	-0.033256	4.155697	-0.315479
C	1.347142	-0.707488	-0.106251
C	2.360588	-0.169611	0.698186
C	1.766755	-1.668139	-1.034626
C	3.690836	-0.563682	0.623925
C	3.096857	-2.081883	-1.128744
C	4.067283	-1.526778	-0.305545
O	-0.912731	-0.591464	-1.060630
B	-1.735855	-1.748451	-0.803927
N	-1.584980	-1.999543	0.757915
C	-0.081922	-1.271717	2.494057
H	0.792305	-0.659738	2.700675
H	-0.738764	-1.203306	3.361819
C	0.252634	-2.750450	2.200415
H	1.302971	-2.872552	1.923409
H	0.073348	-3.370358	3.080987
C	-0.655710	-3.163854	1.024978
H	-0.077458	-3.367813	0.124015
H	-1.269271	-4.038478	1.236030
B	-2.959750	-2.216826	1.574974

H	-3.479746	-3.230129	1.177470
H	-2.723566	-2.216171	2.760131
H	-3.683116	-1.236586	1.319739
O	-3.243824	-1.478061	-1.096106
C	-4.035564	-0.662461	-0.551864
C	-3.660876	0.759321	-0.248876
H	-2.587858	0.894751	-0.177488
H	-4.015936	1.360327	-1.097005
H	-4.167520	1.115442	0.648768
H	-1.520791	-2.717745	-1.467607
C	-5.501062	-1.000840	-0.580127
H	-5.939032	-0.652034	0.360558
H	-5.936205	-0.358007	-1.361954
C	-5.815693	-2.469257	-0.843375
H	-5.399603	-2.792887	-1.799699
H	-5.384412	-3.094043	-0.059513
H	-6.897873	-2.620970	-0.864566
F	-0.549071	1.726398	2.295767
F	-0.575541	4.363707	1.974649
F	-0.030587	5.482340	-0.459336
F	0.557236	3.869912	-2.584750
F	0.589764	1.211063	-2.297763
F	2.076359	0.773653	1.621067
F	4.604130	-0.012982	1.430357
F	5.339024	-1.919041	-0.395606
F	3.439452	-3.013590	-2.023869
F	0.919612	-2.287518	-1.862532

C12-TS1R

C	1.864907	-1.239923	1.545637
C	1.540560	-1.326966	0.032281
H	0.495458	-1.589419	-0.123077
O	1.882281	0.128721	1.910344
B	1.430273	0.949276	0.844757
N	1.740134	0.079440	-0.467868
C	2.489084	-2.209987	-0.790398
H	2.919665	-2.997774	-0.168473
H	1.951294	-2.691952	-1.606222
C	3.566071	-1.242892	-1.347925
H	4.577826	-1.538487	-1.065614
H	3.517863	-1.215289	-2.436165
C	3.216665	0.130638	-0.759699
H	3.746578	0.298735	0.179077
H	3.418546	0.961531	-1.431514
B	0.912648	0.377655	-1.789325

H	1.200772	1.472602	-2.206001
H	1.053362	-0.511740	-2.593227
H	-0.292413	0.360025	-1.470134
O	-0.181808	0.815712	0.890605
C	-1.047082	1.142459	0.027331
C	1.941673	2.455806	0.873355
H	1.918335	2.946891	-0.103035
H	1.351652	3.061199	1.568173
H	2.975737	2.488663	1.227828
C	-2.273135	0.303771	-0.004354
C	-3.340219	0.577989	-0.868043
C	-2.361293	-0.791131	0.866061
C	-4.470456	-0.230541	-0.864208
H	-3.288763	1.411731	-1.554783
C	-3.492897	-1.594616	0.870234
C	-4.549402	-1.317673	0.003460
H	-3.554243	-2.436443	1.549550
H	-5.431560	-1.947138	0.004240
C	-1.072068	2.517950	-0.576414
H	-1.679709	3.145702	0.086442
H	-0.073091	2.931336	-0.638009
H	-1.519731	2.521031	-1.566756
H	-1.537267	-0.985561	1.538783
H	-5.288814	-0.014336	-1.540299
H	1.118554	-1.790827	2.129806
H	2.846166	-1.675976	1.765822

C12-TS1S

C	-2.890077	0.521371	-1.423855
C	-2.564078	-0.633171	-0.444246
H	-2.068918	-1.449728	-0.965463
O	-1.887049	1.515576	-1.269602
B	-0.887103	1.094384	-0.378467
N	-1.571288	-0.027250	0.511011
C	-3.740985	-1.116413	0.411470
H	-4.687052	-0.977082	-0.116283
H	-3.639669	-2.178612	0.631628
C	-3.662364	-0.283828	1.717337
H	-4.569537	0.296143	1.894862
H	-3.513172	-0.940478	2.573455
C	-2.455119	0.646500	1.533193
H	-2.769193	1.613429	1.136937
H	-1.880874	0.808781	2.441910
B	-0.654478	-1.110674	1.248272

H	0.070444	-0.536817	2.022148
H	-1.354417	-1.947545	1.769764
H	0.013318	-1.680727	0.384954
O	0.085433	0.073140	-1.379099
C	0.979163	-0.746891	-1.071772
C	-0.024852	2.203342	0.341370
H	0.738334	1.795092	1.004864
H	0.462957	2.856671	-0.387030
H	-0.677847	2.842729	0.944137
C	2.186312	-0.353813	-0.302035
C	2.813225	-1.223604	0.595903
C	2.760642	0.891290	-0.574680
C	3.987373	-0.841017	1.228615
H	2.346653	-2.169805	0.837364
C	3.948898	1.262770	0.045412
H	2.276783	1.553574	-1.279508
C	4.560025	0.400861	0.951219
H	4.453686	-1.505938	1.945573
H	4.391482	2.226837	-0.174470
H	5.479108	0.695146	1.444295
C	1.038210	-2.035949	-1.844895
H	1.510991	-2.831213	-1.271132
H	0.040675	-2.332059	-2.163489
H	1.656347	-1.853402	-2.732052
H	-2.913261	0.159877	-2.456673
H	-3.865224	0.968899	-1.200504

C12-TS1Spri

C	-2.906564	-1.515379	-0.227907
C	-2.530871	-0.532228	0.882705
H	-1.896736	-1.030244	1.614992
O	-1.777115	-1.590367	-1.068373
B	-1.175378	-0.280242	-1.189031
N	-1.676399	0.494536	0.171075
C	-3.700127	0.218616	1.544388
H	-4.651626	-0.192063	1.194468
H	-3.679320	0.115311	2.629101
C	-3.538806	1.693621	1.113155
H	-4.493058	2.168692	0.877613
H	-3.058439	2.268801	1.903675
C	-2.632516	1.620527	-0.113113
H	-3.217119	1.381234	-1.001640
H	-2.067922	2.530230	-0.305434
B	-0.515556	1.021822	1.136257

H	0.081988	1.920604	0.598281
H	-0.963906	1.280954	2.226822
H	0.279500	0.076187	1.296090
O	0.374876	-0.367445	-1.071052
C	1.070920	-0.838633	-0.119049
C	-1.417224	0.482469	-2.570672
H	-1.046425	1.512473	-2.570348
H	-0.886594	-0.055234	-3.363063
H	-2.472295	0.504208	-2.859976
C	2.422516	-0.261922	0.037310
C	3.387189	-0.860436	0.856992
C	2.744260	0.909353	-0.661841
C	4.652664	-0.298255	0.973267
H	3.156462	-1.763649	1.405634
C	4.007917	1.468248	-0.542364
C	4.964453	0.866309	0.275183
H	4.249087	2.376308	-1.081696
H	5.950698	1.305708	0.369586
C	0.746679	-2.171037	0.498168
H	-0.300193	-2.405646	0.343250
H	1.341915	-2.921455	-0.035790
H	1.011112	-2.205571	1.552850
H	1.987357	1.365909	-1.283368
H	5.394518	-0.766786	1.608617
H	-3.146271	-2.507916	0.164329
H	-3.788303	-1.152731	-0.779559

C12-TS4R

C	2.232512	-0.442003	1.954600
C	1.964842	-1.184102	0.621647
H	0.957330	-1.595895	0.608140
O	2.070043	0.946565	1.717053
B	1.595852	1.190608	0.407676
N	2.030577	-0.101250	-0.422140
C	3.024014	-2.220560	0.227574
H	3.494974	-2.649873	1.114613
H	2.567911	-3.036838	-0.331552
C	4.032384	-1.451465	-0.665138
H	5.045042	-1.471543	-0.258978
H	4.064009	-1.891626	-1.661080
C	3.503588	-0.010861	-0.727265
H	3.977884	0.609124	0.035045
H	3.634906	0.463066	-1.697097
B	1.230630	-0.482666	-1.755224

H	1.383336	0.403952	-2.561571
H	1.603633	-1.558644	-2.159164
H	0.037832	-0.593416	-1.457649
O	-0.026990	0.899808	0.518679
C	-0.904955	0.817665	-0.370715
C	1.928249	2.623874	-0.194336
H	1.905139	2.656670	-1.286513
H	1.239770	3.386485	0.182290
H	2.931504	2.927118	0.118243
C	-1.010267	1.784135	-1.508581
H	-1.768991	2.524342	-1.222811
H	-0.072765	2.291325	-1.697161
H	-1.349845	1.286941	-2.413853
C	-2.084007	-0.064068	-0.032756
C	-2.990825	0.690352	0.973810
C	-2.902403	-0.562282	-1.230201
H	-1.663343	-0.924994	0.496031
C	-4.146844	-0.205586	1.431986
H	-3.394336	1.590120	0.494017
H	-2.391589	1.015952	1.826845
C	-4.047719	-1.466737	-0.761106
H	-3.325252	0.292543	-1.769791
H	-2.249161	-1.093976	-1.926558
C	-4.952617	-0.746880	0.245617
H	-4.796504	0.354076	2.111283
H	-3.739096	-1.045380	2.007419
H	-4.630207	-1.803562	-1.623164
H	-3.628435	-2.367141	-0.295683
H	-5.738427	-1.420311	0.599740
H	-5.457472	0.087395	-0.257600
H	3.253814	-0.621458	2.310369
H	1.541748	-0.785294	2.732256

C12-TS4S

C	-3.092256	-1.435328	0.489636
C	-2.757243	0.045144	0.801611
H	-2.262571	0.130316	1.767306
O	-2.112409	-1.921868	-0.411322
B	-1.075917	-0.976672	-0.602495
N	-1.759437	0.430331	-0.259360
C	-3.930124	1.024763	0.660461
H	-4.879899	0.518521	0.846109
H	-3.841547	1.834145	1.384365
C	-3.828264	1.577657	-0.784962
H	-4.736764	1.400881	-1.363041
H	-3.649917	2.652227	-0.764261
C	-2.634722	0.845412	-1.413042
H	-2.963462	-0.053500	-1.936670
H	-2.053167	1.458371	-2.097569
B	-0.840212	1.654989	0.187404
H	-0.141930	1.982282	-0.742840
H	-1.523340	2.554360	0.614328
H	-0.130126	1.261909	1.124059
O	-0.133364	-1.145756	0.725285
C	0.816293	-0.430134	1.134573
C	-0.281256	-1.109627	-1.974770
H	0.264626	-0.208659	-2.263936
H	0.430860	-1.939391	-1.944444
H	-0.984928	-1.334938	-2.781204
C	1.053681	-0.437026	2.618294
H	1.327600	0.552393	2.980372
H	0.163986	-0.796045	3.131129
H	1.888196	-1.113427	2.834165
C	1.911083	0.046924	0.216598
C	2.713523	1.249137	0.725932
C	2.852092	-1.155436	-0.063314
H	1.445222	0.323276	-0.726244
C	3.779628	1.648250	-0.300345
H	3.206693	0.999168	1.672460
H	2.034043	2.082584	0.916353
C	3.924742	-0.756246	-1.082856
H	3.334280	-1.471405	0.869502
H	2.269516	-2.002197	-0.431164
C	4.713050	0.475895	-0.624746
H	4.355215	2.499499	0.074185
H	3.282271	1.985019	-1.217578
H	4.600080	-1.600183	-1.252398
H	3.438458	-0.543379	-2.041969
H	5.436071	0.766800	-1.392369
H	5.292775	0.219112	0.271130
H	-4.078730	-1.528144	0.020705
H	-3.101972	-2.028817	1.410370

C12-TS4Spri

C	2.920459	0.717274	1.505233
C	2.645546	-0.638229	0.849832
H	1.910046	-1.189642	1.433225
O	1.862973	1.565967	1.105887

B	1.409177	1.215618	-0.214081
N	1.996647	-0.272194	-0.464533
C	3.885977	-1.488583	0.530500
H	4.758472	-1.091496	1.056675
H	3.750844	-2.520010	0.854753
C	4.062015	-1.406782	-1.004592
H	5.096214	-1.213994	-1.296048
H	3.747506	-2.339187	-1.469807
C	3.146236	-0.261436	-1.435714
H	3.672487	0.690841	-1.360963
H	2.757343	-0.361170	-2.446786
B	0.938754	-1.416375	-0.907008
H	0.424988	-1.082539	-1.949292
H	1.517426	-2.475446	-0.968075
H	0.092502	-1.490328	-0.019802
O	-0.185835	1.026334	-0.223154
C	-0.927295	0.320536	0.492078
C	1.628512	2.295303	-1.364397
H	1.327543	1.933527	-2.352128
H	1.023712	3.180406	-1.141402
H	2.666641	2.633908	-1.427108
C	-0.686516	0.107229	1.953714
H	0.259192	0.543995	2.254808
H	-1.502795	0.605862	2.489627
H	-0.739855	-0.952389	2.200802
C	-2.241704	-0.070306	-0.122614
C	-2.888701	-1.327536	0.473110
C	-3.206451	1.141610	-0.033116
H	-2.022922	-0.242899	-1.179917
C	-4.210402	-1.639180	-0.237789
H	-3.086604	-1.175645	1.540556
H	-2.196679	-2.168502	0.385124
C	-4.533691	0.822495	-0.729587
H	-3.395388	1.380738	1.020591
H	-2.733002	2.015283	-0.486494
C	-5.177202	-0.451378	-0.170358
H	-4.668018	-2.527979	0.205668
H	-4.004296	-1.882796	-1.286791
H	-5.214531	1.672359	-0.624727
H	-4.349843	0.695757	-1.802916
H	-6.097269	-0.679143	-0.716199
H	-5.466059	-0.280724	0.874581
H	2.949479	0.645457	2.596704
H	3.888890	1.120391	1.172861

C13-B5-TS1R

C	-0.447598	-0.815138	-0.476126
C	-0.134755	-0.973701	-2.023937
H	0.892611	-0.657926	-2.198594
C	0.605406	0.068673	0.195188
C	1.087808	-0.283972	1.455041
C	1.082628	1.231162	-0.414097
C	2.054930	0.496848	2.102743
H	0.708123	-1.185518	1.917996
C	2.013325	2.060066	0.229969
H	0.759036	1.494446	-1.415208
C	2.499481	1.673490	1.484600
H	3.234843	2.293504	1.986200
C	-1.840213	-0.245950	-0.169017
C	-2.109319	1.114095	-0.328587
C	-2.862909	-1.083671	0.272865
C	-3.379931	1.645392	-0.082089
H	-1.326493	1.783312	-0.658453
C	-4.151713	-0.585827	0.520026
H	-2.635671	-2.124938	0.458563
C	-4.398811	0.779970	0.334445
H	-5.391154	1.174246	0.525377
O	-0.374909	-2.132301	0.054887
B	0.167465	-3.056504	-0.881782
N	-0.192480	-2.449316	-2.283500
C	-1.098419	-0.361008	-3.038704
H	-1.496765	0.601716	-2.717378
H	-0.557338	-0.212766	-3.974510
C	-2.204293	-1.424676	-3.224452
H	-3.123551	-1.128757	-2.713597
H	-2.434013	-1.563876	-4.282325
C	-1.645982	-2.717552	-2.595022
H	-2.175643	-2.948035	-1.669480
H	-1.695474	-3.583341	-3.253380
B	0.695479	-2.882591	-3.578431
H	0.685060	-4.098595	-3.608184
H	0.162844	-2.415906	-4.561933
H	1.817086	-2.427043	-3.439901
O	1.789995	-2.733263	-0.795219
C	2.851421	-3.215521	-1.249084
C	-0.164308	-4.584746	-0.591041
H	0.018056	-5.232970	-1.452681
H	0.403620	-4.971165	0.262848
H	-1.222693	-4.686521	-0.328195

C	4.031318	-2.336672	-1.244254
C	5.239502	-2.724060	-1.849659
C	3.931372	-1.064924	-0.650723
C	6.323400	-1.852989	-1.863247
H	5.328868	-3.691722	-2.328779
C	5.020548	-0.203991	-0.658044
C	6.216248	-0.594855	-1.265420
H	4.929223	0.772423	-0.196234
H	7.065535	0.081465	-1.276044
C	2.946022	-4.622387	-1.749309
H	3.909427	-5.066466	-1.495425
H	2.138152	-5.223387	-1.342347
H	2.827560	-4.600216	-2.836761
H	3.000885	-0.770754	-0.187646
H	7.250342	-2.152151	-2.341115
C	-5.228611	-1.496347	0.976855
C	-6.185312	-1.074347	1.913157
C	-5.317600	-2.805688	0.478281
C	-7.198433	-1.932246	2.335474
H	-6.115763	-0.075070	2.331108
C	-6.329088	-3.664551	0.901597
H	-4.599950	-3.141953	-0.263390
C	-7.274928	-3.231260	1.831666
H	-7.923703	-1.588574	3.066970
H	-6.383767	-4.671134	0.497913
H	-8.063802	-3.900182	2.161521
C	-3.617265	3.096358	-0.269582
C	-4.833223	3.574134	-0.780928
C	-2.615727	4.028952	0.049252
C	-5.041000	4.939022	-0.970106
H	-5.609112	2.866969	-1.056816
C	-2.821713	5.392809	-0.144073
H	-1.680229	3.681534	0.476744
C	-4.035886	5.854191	-0.655015
H	-5.986554	5.287484	-1.374685
H	-2.035957	6.096228	0.114975
H	-4.197786	6.917129	-0.805027
C	2.471495	3.312595	-0.416032
C	3.800772	3.747603	-0.300390
C	1.575077	4.101408	-1.155344
C	4.222695	4.927752	-0.908528
H	4.512178	3.147715	0.258792
C	1.996516	5.281155	-1.764253
H	0.534178	3.802072	-1.229978
C	3.322621	5.698865	-1.644971
H	5.257730	5.241947	-0.813315
H	1.284942	5.879292	-2.325297
H	3.651322	6.617950	-2.119881
C	2.607473	0.073732	3.410801
C	2.907010	1.011037	4.411046
C	2.854131	-1.283550	3.670261
C	3.437132	0.603895	5.633413
H	2.696809	2.061632	4.236700
C	3.383389	-1.690613	4.892588
H	2.648432	-2.016601	2.896626
C	3.677685	-0.748495	5.879246
H	3.653523	1.343078	6.398832
H	3.573060	-2.744528	5.072527
H	4.089716	-1.065671	6.832215

C13-B5-TS1Rpri

C	-0.270522	-0.891505	-0.425476
C	0.052616	-1.433758	-1.850917
H	1.123076	-1.633797	-1.884872
C	0.857566	-0.048546	0.161372
C	0.858688	0.168648	1.541569
C	1.824012	0.568929	-0.627350
C	1.821930	0.983868	2.142718
H	0.085940	-0.297218	2.141843
C	2.803112	1.396957	-0.053845
H	1.858188	0.386379	-1.694268
C	2.785641	1.598830	1.328969
H	3.538100	2.233306	1.784737
C	-1.545276	-0.034313	-0.335394
C	-1.546343	1.282983	-0.801696
C	-2.709675	-0.549623	0.228423
C	-2.703665	2.068655	-0.759330
H	-0.635506	1.712421	-1.201151
C	-3.888841	0.208906	0.281493
H	-2.696764	-1.557534	0.617981
C	-3.873894	1.514224	-0.223439
H	-4.762207	2.130452	-0.136792
O	-0.434780	-2.078061	0.353625
B	-0.704258	-3.239822	-0.430639
N	-0.640195	-2.761979	-1.931928
C	-0.428127	-0.663767	-3.079471
H	-0.389480	0.417235	-2.943201
H	0.218089	-0.922976	-3.919528
C	-1.864972	-1.174717	-3.332853

H	-2.604078	-0.431529	-3.024931
H	-2.018155	-1.385963	-4.392516
C	-2.016974	-2.449137	-2.477734
H	-2.711028	-2.273727	-1.658168
H	-2.356919	-3.316109	-3.041839
B	0.097222	-3.724722	-3.028113
H	-0.329400	-4.848795	-2.872269
H	-0.154906	-3.284608	-4.128105
H	1.298253	-3.653865	-2.817831
O	0.563119	-4.360749	-0.287876
C	1.764441	-4.433408	-0.617573
C	-1.902179	-4.181780	0.029723
H	-2.001192	-5.053211	-0.624621
H	-1.715468	-4.551260	1.044368
H	-2.868944	-3.668996	0.049957
C	2.734685	-3.356943	-0.360620
C	3.817513	-3.137264	-1.229392
C	2.635668	-2.628287	0.833786
C	4.770301	-2.176278	-0.916734
H	3.877209	-3.688677	-2.161365
C	3.633926	-1.719061	1.172909
C	4.688814	-1.481755	0.294710
H	3.566134	-1.169189	2.103391
H	5.445040	-0.744822	0.542806
C	2.240480	-5.752080	-1.158510
H	2.014562	-5.765178	-2.229111
H	3.307791	-5.908505	-1.002249
H	1.657757	-6.549321	-0.693290
H	1.784479	-2.790182	1.482770
H	5.582178	-1.972372	-1.606922
C	-5.121259	-0.367977	0.868918
C	-6.383783	-0.058324	0.339450
C	-5.055082	-1.246482	1.962063
C	-7.542214	-0.605623	0.886161
H	-6.452435	0.596060	-0.523829
C	-6.212932	-1.795949	2.507316
H	-4.089931	-1.480986	2.399183
C	-7.461955	-1.477524	1.972569
H	-8.508385	-0.358831	0.456302
H	-6.139825	-2.468498	3.356650
H	-8.364272	-1.906113	2.397604
C	-2.675521	3.466214	-1.251749
C	-3.774976	4.013996	-1.930158
C	-1.543595	4.273238	-1.054378
C	-3.744680	5.326325	-2.396786
H	-4.648328	3.395759	-2.112838
C	-1.512170	5.584542	-1.523034
H	-0.694802	3.875108	-0.507094
C	-2.612613	6.117095	-2.196183
H	-4.603082	5.728981	-2.926093
H	-0.630063	6.194745	-1.353525
H	-2.588391	7.139412	-2.560645
C	3.855303	2.005128	-0.900996
C	5.170357	2.142999	-0.428765
C	3.567911	2.444226	-2.202912
C	6.164808	2.697691	-1.231202
H	5.416614	1.794478	0.569211
C	4.561908	2.997482	-3.006706
H	2.551883	2.371292	-2.577685
C	5.865331	3.125872	-2.525079
H	7.176845	2.788357	-0.848172
H	4.315836	3.336403	-4.008418
H	6.639990	3.556545	-3.151746
C	1.838986	1.177982	3.611770
C	2.181279	2.416811	4.175522
C	1.522325	0.116778	4.474584
C	2.207357	2.588620	5.557579
H	2.401841	3.255864	3.523214
C	1.547924	0.288503	5.856808
H	1.272093	-0.852954	4.055648
C	1.891508	1.525098	6.404242
H	2.465649	3.557509	5.974153
H	1.305666	-0.546603	6.507082
H	1.911330	1.659535	7.481231

C13-B5-TS1S

C	-0.033627	-0.627919	-0.316356
C	0.410282	-0.783820	-1.830237
H	1.124441	0.004399	-2.056279
C	0.412389	0.730360	0.227921
C	0.956718	0.818731	1.507613
C	0.261731	1.894908	-0.528413
C	1.350546	2.053167	2.041346
H	1.112034	-0.095592	2.065306
C	0.608672	3.149865	-0.007166
H	-0.115891	1.843453	-1.544206
C	1.160233	3.212901	1.277325
H	1.393615	4.180620	1.708160
C	-1.539992	-0.799763	-0.080131

C	-2.434868	0.223759	-0.394451
C	-2.043312	-1.997039	0.426538
C	-3.815247	0.070932	-0.225806
H	-2.064765	1.161355	-0.784789
C	-3.422862	-2.186512	0.603213
H	-1.350091	-2.788555	0.678052
C	-4.299091	-1.145286	0.271828
H	-5.361086	-1.261628	0.459472
O	0.645053	-1.671541	0.381623
B	1.618445	-2.291164	-0.414080
N	1.156522	-2.082781	-1.887229
C	-0.660932	-0.892392	-2.913931
H	-1.528613	-0.263583	-2.711884
H	-0.220388	-0.573136	-3.859970
C	-1.020143	-2.393186	-2.973376
H	-1.985620	-2.584267	-2.499352
H	-1.076359	-2.738704	-4.007252
C	0.095305	-3.121341	-2.197442
H	-0.297237	-3.526108	-1.263426
H	0.568832	-3.926686	-2.756248
B	2.253245	-2.109713	-3.086362
H	2.919626	-3.109082	-2.938072
H	1.648289	-2.104662	-4.137170
H	2.923033	-1.090245	-2.996538
O	2.916204	-1.034902	-0.333488
C	3.922704	-0.710325	-0.990445
C	2.207981	-3.674230	0.058291
H	3.007271	-4.028946	-0.595680
H	2.591204	-3.611249	1.081857
H	1.422629	-4.439063	0.062771
C	5.048099	-1.642049	-1.212893
C	5.870888	-1.550675	-2.344883
C	5.328853	-2.598740	-0.227506
C	6.942749	-2.422414	-2.498165
H	5.637943	-0.836542	-3.126032
C	6.415962	-3.455329	-0.374142
H	4.701599	-2.646040	0.653512
C	7.219727	-3.372479	-1.511608
H	7.560218	-2.366887	-3.388720
H	6.634101	-4.187382	0.396661
H	8.061607	-4.047356	-1.631317
C	4.085563	0.741479	-1.351035
H	4.461444	0.862219	-2.368139
H	3.134821	1.260846	-1.231858
H	4.825688	1.182982	-0.672475

C	-3.937855	-3.467911	1.141860
C	-5.142294	-4.013727	0.671042
C	-3.230469	-4.169472	2.130865
C	-5.625272	-5.219851	1.173532
H	-5.688482	-3.498670	-0.112973
C	-3.711967	-5.376591	2.632091
H	-2.309607	-3.750679	2.523852
C	-4.911774	-5.907113	2.156205
H	-6.555389	-5.628477	0.790006
H	-3.153088	-5.899783	3.402191
H	-5.286983	-6.847827	2.547124
C	-4.721904	1.194173	-0.561069
C	-5.985530	0.963135	-1.124945
C	-4.324462	2.523390	-0.337543
C	-6.823502	2.025302	-1.458699
H	-6.299525	-0.056181	-1.326770
C	-5.159536	3.585323	-0.676302
H	-3.365197	2.722491	0.129923
C	-6.413298	3.340816	-1.238909
H	-7.794636	1.825164	-1.901423
H	-4.833579	4.604269	-0.490035
H	-7.065508	4.167805	-1.502018
C	0.361407	4.380369	-0.794779
C	1.275637	5.444765	-0.781437
C	-0.806116	4.512795	-1.564068
C	1.032633	6.603708	-1.515326
H	2.192065	5.349560	-0.207274
C	-1.048693	5.671060	-2.299021
H	-1.542484	3.714956	-1.563012
C	-0.130098	6.721347	-2.278168
H	1.756122	7.413285	-1.497992
H	-1.961576	5.756264	-2.880792
H	-0.319080	7.624337	-2.850323
C	1.946294	2.130724	3.396330
C	2.969542	3.048542	3.680786
C	1.504266	1.286216	4.426855
C	3.531656	3.121452	4.953310
H	3.341216	3.691916	2.889375
C	2.067107	1.357369	5.698925
H	0.697786	0.586917	4.231531
C	3.082969	2.275577	5.968281
H	4.327094	3.834014	5.150299
H	1.705177	0.700376	6.484073
H	3.520769	2.331482	6.960135

C13-B5-TS1Spri

C	0.251473	-0.430381	-0.244586
C	0.777986	-0.561406	-1.715203
H	1.463222	0.267387	-1.889641
C	0.231722	1.027626	0.216090
C	0.576760	1.327067	1.534479
C	-0.171413	2.059550	-0.633886
C	0.518130	2.642179	2.015251
H	0.927375	0.522951	2.169086
C	-0.278614	3.378692	-0.170563
H	-0.400091	1.855567	-1.673919
C	0.075978	3.656267	1.154262
H	-0.045212	4.664494	1.535586
C	-1.160131	-1.011862	-0.041548
C	-2.293330	-0.317363	-0.468729
C	-1.326500	-2.259308	0.554874
C	-3.576800	-0.860032	-0.345048
H	-2.189925	0.664875	-0.908364
C	-2.597765	-2.838967	0.686817
H	-0.451813	-2.784375	0.908805
C	-3.715729	-2.130642	0.228315
H	-4.707732	-2.543150	0.377425
O	1.188777	-1.153695	0.532784
B	2.029663	-2.063090	-0.207062
N	1.588155	-1.828106	-1.739176
C	-0.212500	-0.691890	-2.871714
H	-1.118901	-0.104649	-2.723693
H	0.275610	-0.336146	-3.780837
C	-0.509569	-2.199161	-2.976209
H	-1.456692	-2.438077	-2.487495
H	-0.581578	-2.515608	-4.019059
C	0.647764	-2.906677	-2.244581
H	0.270491	-3.481499	-1.402427
H	1.220745	-3.575006	-2.886476
B	2.795013	-1.686749	-2.776260
H	3.457528	-2.694924	-2.769211
H	2.379978	-1.342722	-3.857792
H	3.518671	-0.754243	-2.340025
O	3.549097	-1.659486	-0.133235
C	4.173330	-0.682457	-0.664773
C	2.087822	-3.573857	0.325961
H	2.689626	-4.211748	-0.331279
H	2.569281	-3.582570	1.310667
H	1.113755	-4.056853	0.446865
C	5.582390	-0.945124	-1.040981
C	6.464800	0.095624	-1.364314
C	6.040417	-2.271437	-1.064282
C	7.785656	-0.186041	-1.701749
H	6.126148	1.125011	-1.354500
C	7.359589	-2.548622	-1.402327
C	8.234756	-1.507338	-1.721548
H	7.707643	-3.576377	-1.420498
H	9.264694	-1.725534	-1.986746
C	3.720880	0.735102	-0.427737
H	2.671128	0.770969	-0.158749
H	4.291677	1.113804	0.429395
H	3.916682	1.381928	-1.282745
H	5.344960	-3.063974	-0.819023
H	8.463668	0.624189	-1.949704
C	-2.744224	-4.178037	1.305586
C	-3.717581	-5.079874	0.847732
C	-1.904299	-4.582035	2.355719
C	-3.850246	-6.341818	1.422467
H	-4.356506	-4.796560	0.017244
C	-2.034706	-5.844846	2.928609
H	-1.159326	-3.891765	2.737792
C	-3.008827	-6.730327	2.465534
H	-4.605171	-7.026688	1.047966
H	-1.378630	-6.134892	3.743746
H	-3.110200	-7.714525	2.912308
C	-4.749320	-0.080251	-0.807295
C	-5.852243	-0.712647	-1.400525
C	-4.772814	1.318865	-0.678788
C	-6.941997	0.028272	-1.853470
H	-5.840971	-1.790314	-1.530345
C	-5.859599	2.060071	-1.136337
H	-3.944478	1.824746	-0.192643
C	-6.949506	1.417808	-1.726088
H	-7.782430	-0.480079	-2.316531
H	-5.858447	3.139917	-1.021841
H	-7.797598	1.994679	-2.081723
C	-0.794272	4.443901	-1.062200
C	-0.246395	5.735417	-1.040161
C	-1.858361	4.184381	-1.941219
C	-0.743609	6.735583	-1.872795
H	0.589259	5.945968	-0.379957
C	-2.355021	5.184108	-2.774549
H	-2.318924	3.201132	-1.949448
C	-1.799202	6.463777	-2.744294

H	-0.301032	7.726817	-1.846846
H	-3.183914	4.965220	-3.440925
H	-2.186320	7.243225	-3.393184
C	0.897412	2.950310	3.414537
C	1.554856	4.148555	3.734231
C	0.610217	2.050254	4.452782
C	1.912589	4.438811	5.048893
H	1.807548	4.844478	2.940361
C	0.969145	2.339327	5.767151
H	0.081090	1.129993	4.227600
C	1.621433	3.534976	6.071299
H	2.427131	5.368220	5.273932
H	0.730984	1.633103	6.556774
H	1.900373	3.760276	7.095976

C13-TS7R

C	-0.247912	-0.920115	-0.572624
C	-0.089516	-1.024825	-2.153142
H	0.936889	-0.771046	-2.412237
C	0.988778	-0.248474	0.029672
C	1.543667	-0.760871	1.200465
C	1.572428	0.872686	-0.563483
C	2.687917	-0.186323	1.770303
H	1.076137	-1.623199	1.657760
C	2.695801	1.491991	0.001938
H	1.181373	1.265614	-1.496265
C	3.251327	0.944212	1.163255
H	4.134513	1.401337	1.596613
C	-1.517249	-0.180542	-0.128689
C	-1.583190	1.214021	-0.138047
C	-2.638779	-0.902070	0.278671
C	-2.756566	1.893871	0.210088
H	-0.703142	1.793688	-0.382288
C	-3.829653	-0.251791	0.636477
H	-2.564156	-1.978866	0.350054
C	-3.878530	1.146679	0.588933
H	-4.810752	1.656225	0.807785
O	-0.337089	-2.266650	-0.115357
B	0.101411	-3.171988	-1.113635
N	-0.279377	-2.480044	-2.476600
C	-1.093257	-0.287083	-3.038010
H	-1.391604	0.673700	-2.617193
H	-0.628920	-0.106067	-4.008634
C	-2.284106	-1.260123	-3.187897
H	-3.129930	-0.940852	-2.575042
H	-2.619054	-1.306432	-4.226104
C	-1.767321	-2.629791	-2.702548
H	-2.238468	-2.908803	-1.758508
H	-1.919935	-3.434137	-3.419974
B	0.504856	-2.917037	-3.818805
H	0.197910	-4.063743	-4.061649
H	0.206639	-2.157110	-4.711669
H	1.716827	-2.825523	-3.602348
O	1.689373	-3.081131	-1.109783
C	2.496745	-3.734297	-1.811127
C	2.228945	-5.135212	-2.265553
H	1.175057	-5.302787	-2.465281
H	2.817946	-5.372321	-3.151715
H	2.549754	-5.796989	-1.448920
H	-0.255877	-4.307243	-0.981756
C	3.886617	-3.184356	-1.957992
H	4.171443	-3.312440	-3.008002
H	4.543201	-3.866804	-1.395408
C	4.058642	-1.741610	-1.494689
H	3.424055	-1.070641	-2.077404
H	3.786788	-1.620950	-0.445160
H	5.097780	-1.429027	-1.624377
C	-2.792160	3.374674	0.169908
C	-2.089216	4.083207	-0.818461
C	-3.516466	4.106685	1.123137
C	-2.104303	5.475624	-0.848294
H	-1.549619	3.534738	-1.584391
C	-3.535497	5.499398	1.091507
H	-4.044757	3.577358	1.909736
C	-2.827921	6.190385	0.107183
H	-1.559393	6.003063	-1.625588
H	-4.095592	6.046486	1.843895
H	-2.840899	7.275617	0.084394
C	-5.019295	-1.038227	1.040124
C	-5.882631	-0.576484	2.045881
C	-5.313097	-2.264119	0.422377
C	-7.002878	-1.314674	2.420783
H	-5.656338	0.355980	2.553257
C	-6.431792	-3.003921	0.798081
H	-4.669821	-2.626185	-0.373347
C	-7.282444	-2.531994	1.798581
H	-7.653396	-0.943186	3.206918
H	-6.644439	-3.946434	0.302519
H	-8.154824	-3.108052	2.091257

C	3.297786	-0.777964	2.984676
C	3.325120	-2.170604	3.160710
C	3.867884	0.031162	3.979448
C	3.904187	-2.735596	4.294529
H	2.904919	-2.810038	2.390645
C	4.448392	-0.533798	5.112904
H	3.830778	1.110730	3.873377
C	4.469433	-1.919573	5.275431
H	3.919829	-3.815317	4.409666
H	4.876394	0.110043	5.875406
H	4.920519	-2.359523	6.159458
C	3.283671	2.693689	-0.634792
C	4.674224	2.855301	-0.728744
C	2.456185	3.700498	-1.156984
C	5.221391	3.987163	-1.329176
H	5.325864	2.074610	-0.348653
C	3.003439	4.831687	-1.758219
H	1.378366	3.608835	-1.061157
C	4.388213	4.979405	-1.847651
H	6.300013	4.090674	-1.400022
H	2.347267	5.604224	-2.147800
H	4.814616	5.861070	-2.315983

C13-TS7Rpri

C	-0.214114	-0.862242	-0.651129
C	-0.133140	-1.072449	-2.201500
H	0.892713	-1.351745	-2.441309
C	1.118601	-0.400568	-0.067245
C	1.392128	-0.694333	1.270470
C	2.041952	0.338289	-0.806346
C	2.588782	-0.285028	1.869992
H	0.651856	-1.239697	1.842596
C	3.247978	0.770737	-0.231659
H	1.829781	0.608652	-1.834359
C	3.509895	0.443564	1.102429
H	4.465185	0.717757	1.536560
C	-1.323186	0.110278	-0.229893
C	-1.133784	1.492198	-0.313200
C	-2.545434	-0.381179	0.225825
C	-2.169313	2.384350	-0.007365
C	-3.601463	0.486340	0.542976
H	-2.664899	-1.447987	0.356456
C	-3.403444	1.865352	0.406380
O	-0.491765	-2.163617	-0.143801
B	-1.131186	-3.015588	-1.101482
N	-0.991140	-2.272304	-2.500042
C	-0.630747	0.027050	-3.137469
H	-0.417740	1.029479	-2.762580
H	-0.129677	-0.092044	-4.100386
C	-2.139094	-0.235636	-3.282477
H	-2.699560	0.353103	-2.553814
H	-2.498665	0.035088	-4.277912
C	-2.321097	-1.739565	-3.003078
H	-3.087202	-1.914906	-2.248064
H	-2.583379	-2.305332	-3.896122
B	-0.305211	-3.138067	-3.695650
H	-1.005203	-4.099341	-3.915402
H	-0.169227	-2.403070	-4.649025
H	0.808357	-3.496780	-3.296623
O	-0.388008	-4.390346	-1.231950
C	0.825178	-4.615384	-1.467228
C	1.157425	-5.955262	-2.042241
H	1.721595	-6.536077	-1.302380
H	0.248560	-6.484075	-2.324546
H	1.801092	-5.821897	-2.914942
H	-2.254890	-3.363301	-0.853282
C	1.949463	-3.766022	-0.946371
H	2.860142	-4.009841	-1.498924
H	1.733726	-2.710365	-1.076006
C	2.134606	-4.042074	0.561183
H	2.358506	-5.093916	0.763898
H	2.961537	-3.435564	0.936552
H	1.228054	-3.757103	1.097109
H	-4.229805	2.543764	0.588360
H	-0.161745	1.887084	-0.585036
C	-4.905050	-0.053991	0.996588
C	-5.681283	0.633534	1.942825
C	-5.395792	-1.267852	0.489449
C	-6.908465	0.127502	2.364969
H	-5.305316	1.558511	2.368362
C	-6.621587	-1.775535	0.913070
H	-4.819427	-1.807539	-0.255020
C	-7.384510	-1.079484	1.851568
H	-7.489290	0.671918	3.103520
H	-6.984973	-2.713003	0.503038
H	-8.340385	-1.474857	2.181063
C	-1.960688	3.846697	-0.126205
C	-2.549348	4.734976	0.787433
C	-1.166604	4.379279	-1.154017
C	-2.351482	6.109358	0.676836

H	-3.144396	4.339456	1.604440
C	-0.965517	5.753170	-1.263531
H	-0.723858	3.710892	-1.885707
C	-1.557977	6.624670	-0.348851
H	-2.809851	6.778451	1.398885
H	-0.352673	6.144262	-2.070083
H	-1.402516	7.695663	-0.434450
C	2.884015	-0.626781	3.281993
C	3.595536	0.261373	4.103896
C	2.460113	-1.846998	3.831941
C	3.877760	-0.060925	5.429201
H	3.905287	1.222467	3.706116
C	2.740809	-2.169179	5.157697
H	1.923162	-2.554384	3.209204
C	3.452326	-1.278390	5.962104
H	4.422326	0.644340	6.049745
H	2.408691	-3.120995	5.561200
H	3.671064	-1.529343	6.995352
C	4.226566	1.542417	-1.033112
C	4.965685	2.585829	-0.454301
C	4.434272	1.251352	-2.390643
C	5.883517	3.313908	-1.207813
H	4.797186	2.841942	0.586910
C	5.350288	1.980792	-3.145162
H	3.891190	0.430735	-2.848970
C	6.079299	3.015000	-2.556788
H	6.439447	4.122716	-0.743286
H	5.501411	1.734895	-4.191901
H	6.793516	3.583280	-3.144475

C13-TS7S

C	-0.247899	-0.920127	-0.572599
C	-0.089513	-1.024853	-2.153117
H	0.936884	-0.771049	-2.412223
C	0.988784	-0.248450	0.029669
C	1.543744	-0.760852	1.200424
C	1.572361	0.872751	-0.563483
C	2.687994	-0.186270	1.770227
H	1.076273	-1.623214	1.657715
C	2.695723	1.492098	0.001914
H	1.181254	1.265681	-1.496242
C	3.251324	0.944310	1.163191
H	4.134507	1.401465	1.596525
C	-1.517250	-0.180576	-0.128661
C	-1.583223	1.213985	-0.138028
C	-2.638761	-0.902126	0.278714
C	-2.756611	1.893812	0.210116
H	-0.703192	1.793672	-0.382279
C	-3.829646	-0.251870	0.636526
H	-2.564114	-1.978920	0.350099
C	-3.878554	1.146598	0.588975
H	-4.810786	1.656125	0.807834
O	-0.337047	-2.266656	-0.115316
B	0.101453	-3.172009	-1.113582
N	-0.279342	-2.480079	-2.476558
C	-1.093279	-0.287145	-3.037984
H	-1.391623	0.673650	-2.617194
H	-0.628966	-0.106162	-4.008626
C	-2.284123	-1.260199	-3.187805
H	-3.129900	-0.940952	-2.574875
H	-2.619154	-1.306488	-4.225986
C	-1.767281	-2.629870	-2.702524
H	-2.238423	-2.908955	-1.758505
H	-1.919856	-3.434183	-3.419997
B	0.504930	-2.917069	-3.818731
H	0.197993	-4.063768	-4.061602
H	0.206785	-2.157121	-4.711601
H	1.716894	-2.825560	-3.602216
O	1.689406	-3.081183	-1.109723
C	2.496763	-3.734277	-1.811158
C	2.229011	-5.135174	-2.265668
H	1.175042	-5.302994	-2.464748
H	2.817506	-5.371971	-3.152248
H	2.550544	-5.797011	-1.449365
H	-0.255864	-4.307253	-0.981689
C	3.886638	-3.184319	-1.957980
H	4.171487	-3.312357	-3.007989
H	4.543215	-3.866776	-1.395403
C	4.058651	-1.741593	-1.494606
H	3.424011	-1.070599	-2.077234
H	3.786863	-1.621011	-0.445052
H	5.097775	-1.428978	-1.624333
C	-2.792231	3.374615	0.169931
C	-2.089301	4.083157	-0.818442
C	-3.516544	4.106616	1.123162
C	-2.104407	5.475573	-0.848276
H	-1.549700	3.534695	-1.584374
C	-3.535594	5.499329	1.091530
H	-4.044824	3.577282	1.909764
C	-2.828032	6.190325	0.107204

H	-1.559508	6.003020	-1.625572
H	-4.095694	6.046409	1.843921
H	-2.841024	7.275557	0.084415
C	-5.019268	-1.038331	1.040184
C	-5.882605	-0.576604	2.045947
C	-5.313049	-2.264230	0.422443
C	-7.002834	-1.314816	2.420860
H	-5.656327	0.355866	2.553320
C	-6.431726	-3.004055	0.798157
H	-4.669772	-2.626285	-0.373285
C	-7.282380	-2.532143	1.798663
H	-7.653354	-0.943340	3.207000
H	-6.644357	-3.946574	0.302599
H	-8.154745	-3.108219	2.091347
C	3.297951	-0.777929	2.984547
C	3.325347	-2.170574	3.160530
C	3.868073	0.031183	3.979317
C	3.904498	-2.735585	4.294298
H	2.905125	-2.809998	2.390469
C	4.448664	-0.533795	5.112721
H	3.830921	1.110753	3.873286
C	4.469766	-1.919575	5.275198
H	3.920186	-3.815309	4.409397
H	4.876684	0.110035	5.875222
H	4.920917	-2.359540	6.159185
C	3.283498	2.693852	-0.634798
C	4.674038	2.855551	-0.728799
C	2.455931	3.700632	-1.156920
C	5.221114	3.987469	-1.329211
H	5.325740	2.074886	-0.348761
C	3.003094	4.831876	-1.758135
H	1.378122	3.608904	-1.061052
C	4.387856	4.979680	-1.847616
H	6.299727	4.091047	-1.400095
H	2.346861	5.604389	-2.147661
H	4.814188	5.861387	-2.315933

C13-TS7Spri

C	-0.215850	-0.811709	-0.535577
C	-0.206557	-1.029222	-2.089071
H	0.791773	-1.372198	-2.362509
C	1.164126	-0.415382	-0.016086
C	1.585386	-0.900888	1.222708
C	2.002889	0.438054	-0.734839
C	2.833269	-0.545258	1.751103
H	0.941223	-1.592013	1.751584
C	3.246068	0.833622	-0.219564
H	1.712001	0.793874	-1.716623
C	3.649759	0.330411	1.021733
H	4.588708	0.665074	1.449426
C	-1.252026	0.227042	-0.078356
C	-0.998842	1.595967	-0.191686
C	-2.477448	-0.191276	0.438588
C	-1.972921	2.546201	0.136968
H	-0.021305	1.939732	-0.504818
C	-3.473233	0.735397	0.783734
H	-2.646004	-1.248085	0.593873
C	-3.213237	2.100581	0.611803
H	-3.995989	2.823540	0.814333
O	-0.548011	-2.090193	-0.014868
B	-1.283887	-2.905468	-0.934814
N	-1.155949	-2.170244	-2.347161
C	-0.669225	0.096841	-3.012165
H	-0.376793	1.085213	-2.654604
H	-0.216030	-0.058673	-3.993351
C	-2.196434	-0.062576	-3.094297
H	-2.686366	0.571420	-2.352960
H	-2.575540	0.221022	-4.078862
C	-2.469691	-1.546585	-2.787280
H	-3.204078	-1.660225	-1.990463
H	-2.820264	-2.099671	-3.657618
B	-0.594812	-3.077481	-3.562558
H	-1.407363	-3.937722	-3.804618
H	-0.352552	-2.354757	-4.502563
H	0.468521	-3.592285	-3.178325
O	-0.649269	-4.319974	-1.087906
C	0.518126	-4.621525	-1.455949
C	1.742184	-3.872835	-1.020968
H	1.569310	-2.811828	-0.886427
H	2.018293	-4.277777	-0.037533
H	2.568882	-4.053138	-1.708888
H	-2.420416	-3.165662	-0.639667
C	0.717654	-5.995457	-2.035057
H	1.406417	-5.894133	-2.880036
H	1.273459	-6.563709	-1.272413
C	-0.565247	-6.714542	-2.435786
H	-1.229758	-6.838997	-1.577993
H	-1.098285	-6.138074	-3.193980
H	-0.329919	-7.702337	-2.840460
C	4.099426	1.781573	-0.973360

C	5.492714	1.621213	-1.017224
C	3.530045	2.867160	-1.657762
C	6.291875	2.515506	-1.725901
H	5.945592	0.774810	-0.510313
C	4.328732	3.760600	-2.367754
H	2.457520	3.027894	-1.604555
C	5.712969	3.588067	-2.405524
H	7.367533	2.369955	-1.754611
H	3.870504	4.599365	-2.883079
H	6.335573	4.284664	-2.958286
C	3.274488	-1.077105	3.062368
C	4.620991	-1.400940	3.291207
C	2.355335	-1.273256	4.105129
C	5.036009	-1.903164	4.522531
H	5.341059	-1.279378	2.488028
C	2.769586	-1.777100	5.335845
H	1.314663	-1.005265	3.954103
C	4.111698	-2.093870	5.550326
H	6.081274	-2.153788	4.676808
H	2.044030	-1.913935	6.131978
H	4.434406	-2.485484	6.510031
C	-1.685377	3.990719	-0.024758
C	-2.178572	4.932780	0.890973
C	-0.903266	4.451343	-1.096355
C	-1.897450	6.289106	0.742219
H	-2.763515	4.592428	1.739517
C	-0.618577	5.806727	-1.243809
H	-0.540151	3.741958	-1.833653
C	-1.114746	6.732094	-0.324662
H	-2.281887	7.000462	1.466952
H	-0.017423	6.142194	-2.083627
H	-0.893939	7.788760	-0.439094
C	-4.781331	0.270284	1.303322
C	-5.481528	1.014910	2.265576
C	-5.352703	-0.927451	0.844444
C	-6.713053	0.579969	2.749754
H	-5.042521	1.928219	2.654371
C	-6.582824	-1.364090	1.330152
H	-4.836099	-1.510971	0.089264
C	-7.269681	-0.611675	2.283780
H	-7.234119	1.167796	3.499521
H	-7.009167	-2.290318	0.956861
H	-8.228936	-0.951718	2.661628

C8-TS6R

C	-0.211536	-0.737698	-0.038024
C	0.123813	-1.390275	-1.449255
H	1.077367	-0.992623	-1.791350
C	0.762404	0.409053	0.242120
C	1.313960	0.546803	1.513077
C	1.115922	1.322999	-0.751858
C	2.226805	1.566631	1.801453
H	1.041891	-0.187171	2.260523
C	2.000038	2.376590	-0.496503
H	0.707701	1.207929	-1.750408
C	2.549110	2.477512	0.786944
H	3.247749	3.273828	0.997842
C	-1.659456	-0.245018	0.090895
C	-2.062693	0.976540	-0.451021
C	-2.608600	-1.038284	0.733384
C	-3.391796	1.403586	-0.389004
H	-1.325562	1.616185	-0.917711
C	-3.951182	-0.650040	0.813417
H	-2.273829	-1.963427	1.182926
C	-4.324685	0.571798	0.241926
H	-5.357058	0.885103	0.295885
O	-0.016386	-1.782923	0.912617
B	0.720149	-2.854644	0.351955
N	0.330207	-2.853912	-1.176701
C	-0.936315	-1.335602	-2.547863
H	-1.497147	-0.400363	-2.537548
H	-0.436982	-1.426249	-3.514215
C	-1.836120	-2.564929	-2.293927
H	-2.778935	-2.267499	-1.830160
H	-2.067001	-3.079461	-3.229198
C	-1.041525	-3.472705	-1.332902
H	-1.521786	-3.512469	-0.353925
H	-0.912537	-4.489908	-1.698808
B	1.342083	-3.534808	-2.227034
H	1.369201	-4.722985	-1.996140
H	0.977477	-3.282380	-3.353072
H	2.461105	-3.033861	-2.057377
O	2.231937	-2.381547	0.326457
C	3.231894	-3.016235	-0.091682
C	3.351108	-4.504280	0.046312
H	3.661497	-4.955063	-0.894855
H	4.131686	-4.701891	0.792842
H	2.412769	-4.947700	0.366484
C	2.345289	3.354439	-1.628257
C	2.867455	1.621664	3.195224

C	-3.764558	2.760178	-1.004172
C	-4.954154	-1.575821	1.516701
C	3.017253	2.578388	-2.781910
H	2.362712	1.797837	-3.179623
H	3.269322	3.255977	-3.604993
H	3.938972	2.097986	-2.438909
C	1.048768	4.018360	-2.141827
H	1.272980	4.724823	-2.948409
H	0.342971	3.280398	-2.533653
H	0.547836	4.565666	-1.337059
C	3.304756	4.464465	-1.170633
H	3.512201	5.140523	-2.005765
H	2.876085	5.059604	-0.358344
H	4.261211	4.056826	-0.828759
C	3.689135	0.330556	3.408632
H	3.062849	-0.561154	3.319259
H	4.487454	0.255672	2.662411
H	4.149068	0.326510	4.403103
C	3.806630	2.826282	3.365151
H	3.276654	3.774032	3.227441
H	4.229491	2.824896	4.374536
H	4.640638	2.794283	2.657001
C	1.764056	1.712533	4.270401
H	2.208515	1.742259	5.271246
H	1.165356	2.618626	4.134782
H	1.086946	0.855693	4.229287
C	-5.260819	3.078763	-0.857509
H	-5.562505	3.126795	0.193418
H	-5.477480	4.051326	-1.310137
H	-5.884655	2.332196	-1.359086
C	-3.416570	2.751014	-2.508688
H	-2.348763	2.581604	-2.674159
H	-3.966454	1.961133	-3.030191
H	-3.677147	3.710808	-2.968093
C	-2.958197	3.874558	-0.301708
H	-3.187786	3.903080	0.767963
H	-1.880757	3.720957	-0.407286
H	-3.203052	4.852128	-0.731714
C	-6.384928	-1.014474	1.498278
H	-6.753685	-0.878726	0.476628
H	-7.060414	-1.710230	2.005575
H	-6.449176	-0.052543	2.016418
C	-4.963895	-2.946011	0.804338
H	-5.258472	-2.836000	-0.244367
H	-3.978575	-3.419332	0.828361
H	-5.673767	-3.625185	1.289162
C	-4.526115	-1.766461	2.987669
H	-5.223631	-2.434253	3.505353
H	-3.525466	-2.200120	3.062865
H	-4.515148	-0.807280	3.514707
C	4.461508	-2.200086	-0.428725
H	5.020446	-2.199050	0.524310
C	4.119881	-0.751350	-0.786649
H	3.547280	-0.719666	-1.717710
H	3.530018	-0.267073	-0.009601
H	5.042123	-0.182338	-0.933595
H	0.610265	-3.907761	0.913536
C	5.350865	-2.866996	-1.488676
H	6.229765	-2.242882	-1.669511
H	5.702848	-3.854261	-1.179953
H	4.803254	-2.972049	-2.429245

C8-TS6Rpri

C	-0.152938	-0.866398	-0.413323
C	0.038128	-1.662716	-1.749739
H	0.632868	-1.078792	-2.444049
C	0.834654	0.300521	-0.224176
C	0.678453	1.062761	0.940256
C	1.866027	0.626742	-1.100054
C	1.515044	2.138865	1.233057
H	-0.126440	0.798621	1.616582
C	2.729571	1.703847	-0.845387
H	2.032337	0.033185	-1.987345
C	2.537555	2.446163	0.321895
H	3.188362	3.283790	0.526059
C	-1.571605	-0.311450	-0.286685
C	-1.918990	0.875798	-0.932120
C	-2.531759	-0.996052	0.452151
C	-3.211741	1.397831	-0.842256
C	-3.843937	-0.515746	0.550410
H	-2.229517	-1.904997	0.956863
C	-4.161633	0.682088	-0.100463
O	0.074164	-1.808278	0.635089
B	0.478272	-3.101364	0.182131
N	0.827183	-2.903307	-1.377169
C	-1.225985	-2.164307	-2.496891
H	-2.139182	-1.681626	-2.153924
H	-1.113600	-1.933772	-3.560904
C	-1.243997	-3.684787	-2.292040

H	-1.758476	-3.940074	-1.361288
H	-1.734444	-4.218277	-3.110446
C	0.237125	-4.021886	-2.184966
H	0.459996	-4.968320	-1.691642
H	0.712959	-4.021702	-3.170317
B	2.400383	-2.795800	-1.706402
H	2.858833	-3.912349	-1.752378
H	2.542998	-2.171454	-2.736354
H	2.957686	-2.173425	-0.786251
O	1.782485	-3.623228	0.817493
C	2.868300	-2.989792	0.998923
C	4.114771	-3.819786	0.963085
H	4.436591	-4.008020	1.995378
H	3.916861	-4.764020	0.459754
H	4.920308	-3.285886	0.458835
C	3.826277	2.033036	-1.868736
C	1.266209	2.956245	2.508348
H	-0.303083	-3.996689	0.384331
C	2.891249	-1.628607	1.647655
H	2.103823	-1.038953	1.191415
C	2.484259	-1.825036	3.126591
H	3.192856	-2.461412	3.668017
H	2.464969	-0.848116	3.615865
H	1.486938	-2.263765	3.194832
C	4.671395	3.246878	-1.451078
H	5.440024	3.437618	-2.206257
H	4.063950	4.152884	-1.359344
H	5.179538	3.076597	-0.496596
C	4.767393	0.819842	-2.031622
H	5.301222	0.619966	-1.097391
H	4.224439	-0.087217	-2.309471
H	5.512965	1.020659	-2.808801
C	3.164166	2.350064	-3.228005
H	2.477628	3.197852	-3.137036
H	3.926073	2.603879	-3.972948
H	2.596391	1.496726	-3.608344
C	1.392545	2.034010	3.739658
H	1.192882	2.592965	4.660367
H	0.688036	1.199397	3.693150
H	2.403678	1.618701	3.807666
C	-0.159557	3.548749	2.453268
H	-0.271311	4.205851	1.585131
H	-0.919430	2.766728	2.375976
H	-0.365861	4.134260	3.355973
C	2.264921	4.113344	2.668963

H	2.048218	4.662380	3.590453
H	3.296531	3.752509	2.732132
H	2.200469	4.822074	1.837527
C	4.221627	-0.891498	1.507403
H	4.519504	-0.824235	0.458713
H	4.104763	0.128426	1.882126
H	5.026562	-1.372135	2.073586
H	-1.155445	1.409734	-1.487418
H	-5.166154	1.071560	-0.022253
C	-3.525234	2.737942	-1.522016
C	-4.992089	3.159736	-1.341535
C	-3.235832	2.628974	-3.034332
H	-2.190397	2.374836	-3.228786
H	-3.859760	1.855759	-3.493708
H	-3.447401	3.580897	-3.533480
H	-5.680077	2.425619	-1.772697
H	-5.165892	4.115136	-1.846189
H	-5.248859	3.291029	-0.285792
C	-4.870639	-1.311441	1.368399
C	-4.996922	-2.732120	0.776277
C	-6.262539	-0.659735	1.359678
H	-6.243566	0.342406	1.799462
H	-6.666480	-0.581184	0.345394
H	-6.957463	-1.266793	1.948090
H	-5.335589	-2.689410	-0.263857
H	-5.721602	-3.321966	1.348251
H	-4.041306	-3.262773	0.796205
C	-4.388446	-1.407721	2.832015
H	-4.292392	-0.410880	3.274016
H	-5.102676	-1.981429	3.432920
H	-3.415387	-1.900779	2.903313
C	-2.625108	3.831072	-0.903556
H	-2.816065	3.926642	0.169985
H	-1.564150	3.599522	-1.031549
H	-2.820301	4.800682	-1.374985

C8-TS6S

C	-0.128953	-0.647655	-0.134223
C	0.219245	-1.204630	-1.584883
H	1.054004	-0.630742	-1.983055
C	0.693027	0.614979	0.146492
C	1.266903	0.792609	1.402732
C	0.882481	1.596332	-0.827765
C	2.048606	1.914305	1.692657

H	1.122617	0.008761	2.135049
C	1.624953	2.753925	-0.566253
H	0.448847	1.460175	-1.812965
C	2.208411	2.888919	0.698613
H	2.800980	3.766355	0.912458
C	-1.621029	-0.366753	0.077935
C	-2.211173	0.810477	-0.384580
C	-2.421912	-1.314002	0.713600
C	-3.581691	1.046288	-0.246689
H	-1.590019	1.565185	-0.848413
C	-3.799302	-1.118371	0.868966
H	-1.943248	-2.204775	1.097905
C	-4.361643	0.065812	0.378421
H	-5.423152	0.231177	0.491337
O	0.257024	-1.686621	0.765665
B	1.182900	-2.558438	0.139504
N	0.716871	-2.608022	-1.368443
C	-0.909644	-1.335449	-2.605622
H	-1.631395	-0.521125	-2.531329
H	-0.473353	-1.317966	-3.606125
C	-1.544048	-2.714353	-2.324309
H	-2.480220	-2.604704	-1.773131
H	-1.760775	-3.243105	-3.255144
C	-0.516018	-3.480540	-1.466671
H	-0.903490	-3.653546	-0.461198
H	-0.218154	-4.436570	-1.893944
B	1.777865	-3.051894	-2.492374
H	2.045386	-4.220663	-2.313948
H	1.310454	-2.838366	-3.587878
H	2.791463	-2.354513	-2.352606
O	2.531931	-1.741415	0.050843
C	3.631313	-2.091085	-0.445111
C	4.558968	-0.975016	-0.819875
H	5.419651	-0.975546	-0.140485
H	4.941270	-1.123589	-1.831374
H	4.032578	-0.023218	-0.747288
C	-4.164657	2.364032	-0.777112
C	-4.632705	-2.204902	1.563623
C	1.775712	3.811122	-1.669238
C	2.730600	2.012998	3.064329
H	1.346830	-3.619330	0.672326
C	4.129206	-3.511465	-0.345942
H	3.266224	-4.163908	-0.477530
C	5.188476	-3.867692	-1.388498
H	4.795173	-3.726876	-2.397713
H	6.096997	-3.267980	-1.271865
H	5.470456	-4.918099	-1.276467
C	-4.489129	-3.529786	0.783227
H	-4.847430	-3.415171	-0.244951
H	-3.448071	-3.861369	0.741004
H	-5.074221	-4.322461	1.262387
C	-6.125865	-1.846763	1.631980
H	-6.296080	-0.927883	2.201701
H	-6.557280	-1.716318	0.634460
H	-6.675720	-2.651891	2.129131
C	-4.113693	-2.400690	3.004348
H	-4.208241	-1.473864	3.578784
H	-4.688705	-3.180995	3.515205
H	-3.060928	-2.694904	3.016389
C	-3.895209	2.466122	-2.294242
H	-4.360052	1.631466	-2.828589
H	-4.304988	3.400067	-2.694250
H	-2.824081	2.450474	-2.514859
C	-5.680847	2.468084	-0.548581
H	-6.049202	3.420602	-0.941938
H	-6.223124	1.665765	-1.058807
H	-5.932607	2.428485	0.515778
C	-3.481474	3.547665	-0.057563
H	-3.657983	3.498874	1.021418
H	-2.399959	3.548226	-0.218581
H	-3.877818	4.499720	-0.427692
C	0.375149	4.305820	-2.093536
H	-0.155308	4.745038	-1.242769
H	0.458020	5.067959	-2.876019
H	-0.240313	3.491684	-2.486747
C	2.594204	5.027317	-1.207226
H	2.665439	5.755077	-2.021383
H	2.127428	5.527315	-0.352934
H	3.613305	4.745786	-0.924178
C	2.487679	3.181503	-2.886157
H	3.485544	2.826517	-2.608926
H	1.929715	2.330359	-3.286041
H	2.598099	3.917652	-3.689883
C	3.513495	3.323787	3.241046
H	3.972901	3.348311	4.234076
H	4.315896	3.420911	2.502870
H	2.861175	4.198457	3.154396
C	3.718705	0.833523	3.206680
H	3.208840	-0.129025	3.112230
H	4.488892	0.881628	2.429344

H	4.215647	0.862501	4.182770
C	1.667556	1.930331	4.180035
H	1.104249	0.995006	4.133862
H	2.143088	1.988498	5.165277
H	0.953259	2.755207	4.095447
C	4.652559	-3.688188	1.100333
H	5.507343	-3.034986	1.304637
H	3.868843	-3.477450	1.831823
H	4.982192	-4.721850	1.234693

C8-TS6Spri

C	0.164204	-0.505424	-0.130769
C	0.531178	-1.105170	-1.531796
H	1.526433	-0.742858	-1.790496
C	0.845068	0.840642	0.101125
C	1.216019	1.188434	1.405814
C	1.059365	1.744767	-0.934152
C	1.800223	2.422990	1.679607
H	1.044724	0.459531	2.185237
C	1.626697	3.007022	-0.695410
H	0.778097	1.475604	-1.945744
C	1.990861	3.319957	0.612919
H	2.436900	4.284051	0.821016
C	-1.348557	-0.357796	0.086304
C	-2.047990	0.726021	-0.461485
C	-2.048987	-1.327544	0.793838
C	-3.433617	0.819442	-0.356665
H	-1.491251	1.505591	-0.961527
C	-3.445975	-1.270601	0.920244
H	-1.492748	-2.137134	1.245907
C	-4.116263	-0.199135	0.330639
H	-5.194013	-0.137638	0.411584
O	0.697448	-1.446707	0.793450
B	0.833856	-2.761365	0.248491
N	0.644421	-2.594035	-1.327377
C	-0.435476	-0.930492	-2.701868
H	-0.936080	0.038827	-2.696259
H	0.132443	-1.019174	-3.630716
C	-1.427895	-2.097036	-2.566912
H	-2.299878	-1.787747	-1.987598
H	-1.778322	-2.441106	-3.543119
C	-0.658095	-3.201767	-1.819745
H	-1.223503	-3.577914	-0.967980
H	-0.404341	-4.043631	-2.462949
B	1.850030	-3.165949	-2.240923
H	1.908222	-4.365924	-2.101064
H	1.660962	-2.818632	-3.385667
H	2.892878	-2.636088	-1.833364
O	2.266385	-3.337451	0.482298
C	3.383276	-2.857789	0.152503
C	3.694096	-1.393370	0.246392
H	2.854476	-0.762591	-0.020773
H	3.911182	-1.195105	1.304839
H	4.576331	-1.136185	-0.337980
C	-4.223411	1.987871	-0.962408
C	-4.169232	-2.384601	1.689092
C	1.813580	3.976420	-1.870360
C	2.237124	2.831549	3.093182
H	0.137819	-3.622270	0.720303
C	4.535692	-3.834339	0.040539
H	5.202118	-3.541403	0.869946
C	4.089143	-5.283822	0.236559
H	3.581439	-5.423612	1.192998
H	3.396582	-5.572571	-0.557440
H	4.959715	-5.944684	0.202318
C	-3.853578	-3.744150	1.027785
H	-4.182819	-3.753068	-0.016383
H	-2.782485	-3.962461	1.045915
H	-4.368837	-4.554402	1.555120
C	-5.694277	-2.195038	1.699105
H	-6.164198	-3.014567	2.251441
H	-5.982869	-1.258425	2.186527
H	-6.108795	-2.196323	0.685920
C	-3.669665	-2.398385	3.149763
H	-3.885901	-1.444953	3.642005
H	-4.164091	-3.196118	3.714981
H	-2.590775	-2.566228	3.203501
C	-5.225085	1.439025	-2.001037
H	-5.931503	0.737128	-1.549361
H	-5.802344	2.256827	-2.446172
H	-4.699654	0.913596	-2.804849
C	-4.992296	2.721739	0.157236
H	-5.566233	3.557771	-0.257301
H	-5.692265	2.056260	0.669863
H	-4.299429	3.119086	0.905442
C	-3.309923	3.006094	-1.664904
H	-2.583066	3.443685	-0.973802
H	-2.759031	2.551537	-2.494486
H	-3.913284	3.821437	-2.075673

C	2.739577	3.330728	-2.923698
H	3.722902	3.114383	-2.494444
H	2.327584	2.392301	-3.304932
H	2.878758	4.005503	-3.775253
C	0.437328	4.269509	-2.507510
H	-0.036842	3.359106	-2.884424
H	-0.240985	4.720328	-1.776399
H	0.544802	4.962941	-3.348622
C	2.436134	5.311914	-1.433568
H	3.429463	5.171253	-0.995968
H	2.545858	5.969151	-2.301539
H	1.808955	5.830025	-0.701394
C	1.951231	1.733039	4.129612
H	0.883437	1.502883	4.190975
H	2.485476	0.807029	3.895307
H	2.278821	2.065758	5.119388
C	1.475236	4.106012	3.516127
H	1.776140	4.413974	4.523426
H	1.673481	4.940607	2.837946
H	0.395600	3.927279	3.520764
C	3.754766	3.115547	3.094845
H	4.017245	3.920800	2.403129
H	4.087474	3.410994	4.095862
H	4.315703	2.223552	2.797749
C	5.340925	-3.667144	-1.262553
H	4.739188	-3.985159	-2.115319
H	5.655693	-2.635083	-1.433463
H	6.239193	-4.288376	-1.209476

C8-TS4R

C	-0.295630	-0.731490	-0.084380
C	0.053611	-1.371088	-1.497991
H	0.981669	-0.928953	-1.855329
C	0.614374	0.469226	0.186569
C	1.127180	0.663473	1.462831
C	0.927878	1.387167	-0.823648
C	1.963536	1.751297	1.753127
H	0.882943	-0.070244	2.220395
C	1.740611	2.492066	-0.571444
H	0.539927	1.226408	-1.821292
C	2.252011	2.652106	0.726360
H	2.894260	3.499488	0.928959
C	-1.764151	-0.310709	0.062834
C	-2.233807	0.881808	-0.490171
C	-2.662050	-1.135335	0.737980
C	-3.578855	1.249841	-0.405925
H	-1.535646	1.544805	-0.983922
C	-4.019165	-0.806637	0.840540
H	-2.275748	-2.036490	1.194956
C	-4.459633	0.387543	0.258409
H	-5.503760	0.655023	0.330150
O	-0.034329	-1.761760	0.866827
B	0.757553	-2.789495	0.297573
N	0.335673	-2.822533	-1.223874
C	-1.024783	-1.373603	-2.580123
H	-1.630135	-0.466835	-2.565453
H	-0.537151	-1.445403	-3.553776
C	-1.859650	-2.644010	-2.305398
H	-2.811872	-2.390279	-1.834769
H	-2.072847	-3.178427	-3.233665
C	-1.010897	-3.501544	-1.344443
H	-1.471645	-3.543736	-0.356302
H	-0.845966	-4.518421	-1.696164
B	1.349942	-3.461285	-2.302750
H	1.401978	-4.655228	-2.097796
H	0.961305	-3.196747	-3.417362
H	2.463912	-2.953233	-2.132840
O	2.231895	-2.217859	0.234613
C	3.269697	-2.810447	-0.150743
C	4.467170	-1.931658	-0.479060
C	-4.024719	2.577650	-1.034976
C	-4.964035	-1.764902	1.579830
C	2.099513	3.513820	-1.658873
C	2.540450	1.893991	3.168137
H	0.732109	-3.841920	0.869851
C	-3.716148	2.553650	-2.547903
H	-4.028272	3.492724	-3.017954
H	-2.646943	2.423863	-2.739039
H	-4.246268	1.731863	-3.039650
C	-3.248504	3.738315	-0.374565
H	-3.451482	3.778033	0.700089
H	-2.168079	3.627810	-0.504348
H	-3.545246	4.696327	-0.815699
C	-5.528751	2.836184	-0.853555
H	-5.804688	2.889113	0.204187
H	-5.798031	3.791036	-1.315576
H	-6.133315	2.055883	-1.326504
C	-6.418918	-1.269140	1.583932
H	-7.051349	-1.986578	2.115934

H	-6.515977	-0.303044	2.089071
H	-6.814719	-1.166254	0.568583
C	-4.926195	-3.145758	0.889323
H	-5.248525	-3.066693	-0.153984
H	-3.919729	-3.572839	0.898188
H	-5.593125	-3.848594	1.400687
C	-4.496910	-1.911602	3.043951
H	-4.518163	-0.944241	3.555526
H	-5.152455	-2.601280	3.587157
H	-3.476351	-2.298724	3.103994
C	3.630424	3.526540	-1.859031
H	3.910953	4.254460	-2.628166
H	4.155386	3.792547	-0.937471
H	3.986163	2.540516	-2.174496
C	1.623404	4.914876	-1.218701
H	0.538687	4.925721	-1.071022
H	2.090827	5.225084	-0.280280
H	1.872813	5.660848	-1.981174
C	1.440796	3.186398	-3.008883
H	1.762743	2.213336	-3.392799
H	0.348368	3.181787	-2.935715
H	1.719736	3.943023	-3.748488
C	1.385276	1.957883	4.189840
H	0.740314	2.819340	3.990694
H	0.763398	1.059925	4.153784
H	1.780590	2.053412	5.207044
C	3.397851	3.159270	3.329336
H	3.778987	3.218912	4.353457
H	4.260090	3.154039	2.654960
H	2.817626	4.067151	3.136787
C	3.427136	0.666025	3.470423
H	4.266859	0.618076	2.769278
H	3.833713	0.724844	4.486076
H	2.862238	-0.266348	3.384558
C	3.423831	-4.290453	0.032557
H	3.581232	-4.463130	1.104968
H	2.520645	-4.813067	-0.270541
H	4.275825	-4.683498	-0.517023
C	5.305702	-2.510630	-1.633248
H	4.697502	-2.632994	-2.532286
H	6.121997	-1.817777	-1.856896
H	5.755116	-3.474956	-1.383249
C	4.009234	-0.508034	-0.827784
H	3.435856	-0.053614	-0.020423
H	4.886344	0.114543	-1.027278
H	3.383368	-0.515862	-1.723698
C	5.318274	-1.900746	0.819903
H	4.741155	-1.498623	1.656887
H	5.688713	-2.893852	1.090224
H	6.183480	-1.251516	0.657252

C8-TS4Rpri

C	0.208477	0.745024	-0.215792
C	-0.056974	1.464180	-1.576702
H	-1.118362	1.364106	-1.804545
C	-0.692707	-0.464441	0.012823
C	-0.808665	-0.944264	1.319213
C	-1.293498	-1.169300	-1.027839
C	-1.509808	-2.117386	1.602751
H	-0.319604	-0.384407	2.107574
C	-2.004770	-2.354650	-0.786397
H	-1.185685	-0.815452	-2.046510
C	-2.106413	-2.803839	0.533702
H	-2.644790	-3.718004	0.735221
C	1.661795	0.277674	-0.049693
C	2.092801	-0.907979	-0.648689
C	2.569252	1.029045	0.692450
C	3.420414	-1.330311	-0.559168
H	1.371393	-1.521031	-1.175665
C	3.911935	0.644816	0.800090
H	2.211775	1.919944	1.190892
C	4.319316	-0.531726	0.160776
H	5.351587	-0.840906	0.236388
O	-0.105567	1.735326	0.760395
B	0.040877	3.071891	0.261361
N	0.198373	2.924117	-1.316173
C	0.800161	1.111961	-2.790815
H	1.059123	0.052901	-2.830117
H	0.235906	1.360376	-3.691860
C	2.043343	2.013500	-2.672200
H	2.867554	1.467740	-2.208286
H	2.378061	2.356547	-3.653886
C	1.619977	3.192234	-1.773266
H	2.268877	3.275197	-0.902552
H	1.621384	4.148022	-2.295555
B	-0.799033	3.830348	-2.217622
H	-0.577950	4.994323	-1.974965
H	-0.630802	3.533056	-3.380028
H	-1.952761	3.539357	-1.889270
O	-1.237874	3.936885	0.524242

C	-2.428516	3.737975	0.163526
C	-3.288984	4.959259	0.056821
H	-4.100903	4.909544	0.790708
H	-2.684226	5.849770	0.219628
H	-3.743849	4.998681	-0.934977
C	-2.580767	-3.139291	-1.974240
C	-1.548218	-2.642532	3.045335
C	3.827524	-2.649161	-1.231075
C	4.874500	1.521984	1.612990
C	3.527911	-2.566198	-2.743572
H	2.463600	-2.403285	-2.935564
H	4.082077	-1.742654	-3.204845
H	3.818199	-3.496926	-3.243165
C	5.321344	-2.961888	-1.051321
H	5.588790	-3.064029	0.004982
H	5.563455	-3.906508	-1.548014
H	5.953063	-2.183498	-1.490771
C	3.009234	-3.803360	-0.610876
H	3.276898	-4.756916	-1.079421
H	3.204125	-3.882306	0.463261
H	1.934124	-3.652386	-0.743404
C	4.884821	2.948402	1.021196
H	3.894065	3.408998	1.060147
H	5.573539	3.589736	1.581953
H	5.208896	2.932141	-0.024434
C	6.313967	0.983467	1.598524
H	6.717948	0.935291	0.582278
H	6.960816	1.645606	2.182233
H	6.377718	-0.015877	2.040454
C	4.390919	1.580329	3.078103
H	3.381682	1.994074	3.151465
H	4.376764	0.579622	3.521506
H	5.058341	2.210771	3.675924
C	-3.556905	-2.250299	-2.772811
H	-3.075442	-1.337536	-3.134369
H	-3.939250	-2.792887	-3.644028
H	-4.410076	-1.956892	-2.153969
C	-1.413883	-3.570176	-2.891536
H	-0.870832	-2.706415	-3.285606
H	-0.700012	-4.193103	-2.343119
H	-1.788815	-4.147375	-3.743664
C	-3.337023	-4.401907	-1.530990
H	-4.179738	-4.158653	-0.876207
H	-3.736132	-4.919152	-2.408760
H	-2.683113	-5.102441	-1.002681
C	-2.141687	-1.569910	3.982389
H	-1.582505	-0.631990	3.932326
H	-3.181070	-1.355397	3.718251
H	-2.118862	-1.919490	5.020144
C	-2.392251	-3.919647	3.183539
H	-3.430239	-3.753156	2.878005
H	-1.986407	-4.742287	2.586632
H	-2.400844	-4.243507	4.228737
C	-0.102272	-2.963107	3.485739
H	0.344480	-3.716003	2.828781
H	0.533202	-2.074161	3.453110
H	-0.091699	-3.350745	4.510335
C	-3.061434	2.375952	0.141304
C	-3.475307	2.035541	1.602960
C	-4.253624	2.242123	-0.816254
H	-2.299121	1.647627	-0.124959
C	-4.115360	0.642708	1.666288
H	-4.185381	2.787222	1.972837
H	-2.585815	2.072580	2.237829
C	-4.837691	0.827600	-0.742797
H	-5.034799	2.965914	-0.550457
H	-3.926630	2.476446	-1.834352
C	-5.280580	0.487690	0.684176
H	-4.449870	0.451150	2.692159
H	-3.350775	-0.105469	1.436962
H	-5.676604	0.734533	-1.441331
H	-4.074117	0.108462	-1.062337
H	-5.670421	-0.535504	0.728317
H	-6.104874	1.153476	0.976860
H	0.902498	3.744867	0.763210

C8-TS4S

C	0.263240	0.872300	-0.353641
C	0.113796	1.432540	-1.831989
H	-0.703016	0.922824	-2.337048
C	-0.845434	-0.141000	-0.026141
C	-1.393666	-0.142433	1.256881
C	-1.289328	-1.080660	-0.958405
C	-2.404272	-1.038270	1.610752
H	-1.032126	0.600222	1.953077
C	-2.282887	-2.013280	-0.635759
H	-0.862496	-1.086541	-1.952556
C	-2.832684	-1.964008	0.648701
H	-3.616190	-2.667393	0.910004

C	1.613759	0.210485	-0.058924
C	1.923081	-1.047479	-0.593152
C	2.546400	0.851297	0.746987
C	3.150235	-1.656805	-0.347135
H	1.184003	-1.555036	-1.195356
C	3.796787	0.270767	1.017252
H	2.278031	1.811984	1.166132
C	4.077884	-0.975686	0.460048
H	5.035030	-1.441841	0.656059
O	0.134993	2.019105	0.483357
B	-0.647029	3.007330	-0.155805
N	-0.296354	2.885551	-1.684155
C	1.374063	1.468877	-2.694692
H	1.974271	0.562780	-2.606176
H	1.074198	1.588504	-3.739947
C	2.097426	2.725826	-2.203130
H	2.636966	2.509170	-1.278192
H	2.815728	3.113703	-2.930405
C	0.956823	3.715222	-1.952291
H	1.142911	4.376229	-1.105479
H	0.756029	4.326824	-2.831144
B	-1.404749	3.296711	-2.787516
H	-1.587427	4.492004	-2.696095
H	-0.968648	2.988618	-3.877097
H	-2.431057	2.662614	-2.571450
O	-2.161233	2.452280	-0.077233
C	-3.308032	2.576549	-0.551601
C	-4.012656	1.284930	-0.865963
H	-4.961824	1.424584	-1.379216
H	-3.348325	0.651996	-1.452935
H	-4.182305	0.758083	0.079697
C	3.507287	-3.035566	-0.919364
C	4.792892	1.028229	1.905919
C	-2.783186	-3.060863	-1.640500
C	-3.060998	-1.027249	2.998622
H	-0.589939	4.099589	0.318654
C	-4.089876	3.879938	-0.511861
C	-3.212952	5.072181	-0.114869
H	-3.839940	5.966109	-0.044629
H	-2.732835	4.912232	0.852593
H	-2.437495	5.249414	-0.860285
C	4.750386	-2.905210	-1.825269
H	5.026306	-3.880582	-2.240928
H	4.553167	-2.221738	-2.657212
H	5.612163	-2.519599	-1.273546
C	2.362065	-3.634444	-1.753782
H	1.453093	-3.764985	-1.157623
H	2.118308	-3.005912	-2.616407
H	2.656810	-4.617660	-2.133087
C	3.815362	-4.006593	0.240676
H	4.075486	-4.997059	-0.148618
H	4.652821	-3.655773	0.849805
H	2.945958	-4.113190	0.896828
C	6.114385	0.265173	2.088821
H	6.790392	0.845324	2.724490
H	5.958107	-0.705540	2.569849
H	6.620149	0.096733	1.132761
C	5.108594	2.395461	1.261281
H	5.551252	2.263020	0.268857
H	4.208050	3.005000	1.148280
H	5.817749	2.955817	1.880420
C	4.163654	1.250969	3.298028
H	3.934282	0.293617	3.776481
H	4.854637	1.801334	3.946101
H	3.234572	1.823266	3.232841
C	-4.289984	-2.842776	-1.896442
H	-4.671249	-3.588480	-2.602458
H	-4.871980	-2.925151	-0.974331
H	-4.471215	-1.849458	-2.319762
C	-2.557787	-4.472447	-1.057265
H	-1.494054	-4.646022	-0.866088
H	-3.093152	-4.611479	-0.114149
H	-2.908901	-5.236654	-1.759115
C	-2.048009	-2.977397	-2.988296
H	-2.193838	-2.006624	-3.472379
H	-0.972447	-3.146122	-2.873493
H	-2.433212	-3.745473	-3.665727
C	-2.449772	0.039080	3.921634
H	-1.381782	-0.132569	4.084878
H	-2.572631	1.046407	3.511907
H	-2.946343	0.011628	4.896526
C	-2.879389	-2.408157	3.664091
H	-3.340028	-2.417067	4.657899
H	-3.339372	-3.206332	3.074907
H	-1.817102	-2.645258	3.776709
C	-4.567712	-0.725469	2.845885
H	-5.066294	-1.459450	2.206655
H	-5.062974	-0.738347	3.822882
H	-4.721574	0.265422	2.404453
C	-4.794190	4.168020	-1.851616

H	-4.058278	4.346797	-2.635882
H	-5.446408	3.349100	-2.165401
H	-5.415444	5.061324	-1.735548
C	-5.159537	3.648159	0.595408
H	-5.863706	2.854507	0.333698
H	-4.692812	3.400255	1.554016
H	-5.726952	4.573943	0.725070

C8-TS4Spri

C	0.130661	-0.399161	-0.180709
C	0.591165	-0.861904	-1.606750
H	1.446261	-0.247901	-1.890727
C	0.452905	1.072695	0.063809
C	0.771818	1.482075	1.364318
C	0.390836	2.018516	-0.954340
C	1.032077	2.820147	1.650682
H	0.821662	0.720753	2.129836
C	0.626616	3.379750	-0.701786
H	0.149890	1.703134	-1.963025
C	0.947704	3.753668	0.601744
H	1.140692	4.796152	0.820058
C	-1.361635	-0.646071	0.083595
C	-2.333250	0.231382	-0.416208
C	-1.765893	-1.773469	0.788733
C	-3.692283	-0.033770	-0.266125
H	-2.011979	1.134958	-0.914328
C	-3.125754	-2.077043	0.959876
H	-1.005478	-2.420959	1.203115
C	-4.067686	-1.203176	0.417542
H	-5.121931	-1.419908	0.533716
O	0.915475	-1.186703	0.705696
B	1.373466	-2.411657	0.127071
N	1.096513	-2.272618	-1.441787
C	-0.430938	-0.928322	-2.740248
H	-1.162868	-0.120373	-2.698468
H	0.105710	-0.858000	-3.689143
C	-1.083649	-2.312187	-2.595149
H	-1.976303	-2.246203	-1.970468
H	-1.381199	-2.720352	-3.564242
C	-0.019535	-3.191402	-1.913650
H	-0.428208	-3.727706	-1.058089
H	0.420702	-3.917600	-2.595900
B	2.376057	-2.499060	-2.402931
H	2.711448	-3.657536	-2.332500
H	2.083731	-2.144410	-3.523254
H	3.287746	-1.767814	-1.983620
O	2.907819	-2.597361	0.316420
C	3.850315	-1.839293	-0.043589
C	3.747995	-0.344573	0.054814
H	2.776109	0.028092	-0.243635
H	3.860576	-0.091141	1.116402
H	4.537022	0.150469	-0.507669
C	-4.775016	0.903953	-0.817663
C	-3.512933	-3.350810	1.723234
C	0.517332	4.383943	-1.857166
C	1.402944	3.303371	3.059579
H	0.934519	-3.430917	0.593442
C	5.223786	-2.490865	-0.166034
C	5.089761	-4.017444	-0.251991
H	4.600820	-4.425367	0.634769
H	4.498251	-4.300897	-1.124331
H	6.085627	-4.462238	-0.336990
C	-2.888297	-4.574115	1.017036
H	-3.244943	-4.649650	-0.015371
H	-1.797029	-4.512309	0.993492
H	-3.159784	-5.497406	1.540418
C	-5.034649	-3.555610	1.788338
H	-5.259798	-4.476301	2.335295
H	-5.532430	-2.730964	2.308234
H	-5.473866	-3.646404	0.789735
C	-2.970602	-3.261327	3.165875
H	-3.402399	-2.402489	3.689361
H	-3.224679	-4.167804	3.726289
H	-1.883171	-3.150287	3.179830
C	-5.644115	0.133980	-1.835082
H	-6.131745	-0.731015	-1.377128
H	-6.426508	0.784873	-2.240522
H	-5.034228	-0.228533	-2.668686
C	-5.661500	1.402075	0.343975
H	-6.443686	2.071028	-0.031403
H	-6.150181	0.573148	0.863301
H	-5.063867	1.951201	1.078177
C	-4.177008	2.130955	-1.525925
H	-3.559611	2.729392	-0.849092
H	-3.560367	1.843221	-2.383428
H	-4.982566	2.771621	-1.897650
C	1.539252	4.014320	-2.953959
H	2.559988	4.051262	-2.560614
H	1.367979	3.007541	-3.344834
H	1.470108	4.715321	-3.792810

C	-0.909995	4.325480	-2.444737
H	-1.149126	3.330670	-2.830621
H	-1.654366	4.574889	-1.682132
H	-1.013359	5.037679	-3.270541
C	0.793360	5.826937	-1.406091
H	1.804609	5.938140	-1.002387
H	0.700630	6.504582	-2.260247
H	0.081142	6.155003	-0.642547
C	1.450026	2.150608	4.075219
H	0.480622	1.651707	4.166129
H	2.194814	1.397936	3.798324
H	1.719654	2.539376	5.062053
C	0.354802	4.329860	3.540504
H	0.604471	4.688470	4.545049
H	0.304684	5.198341	2.877916
H	-0.641088	3.877591	3.575865
C	2.794964	3.970119	3.019679
H	2.814574	4.827956	2.341801
H	3.078219	4.323871	4.017001
H	3.555114	3.258833	2.680805
C	6.000109	-1.969073	-1.390197
H	5.479506	-2.224158	-2.314654
H	6.148452	-0.886518	-1.363102
H	6.989484	-2.435856	-1.403915
C	5.991513	-2.108595	1.128933
H	6.177086	-1.033203	1.195395
H	5.444715	-2.426538	2.021831
H	6.959384	-2.617766	1.124792

C8-TS7Rpri

C	-0.031170	-0.832172	-0.339007
C	0.184523	-1.729890	-1.612397
H	0.859561	-1.229196	-2.297945
C	0.961415	0.337553	-0.225332
C	0.728089	1.269201	0.792090
C	2.123953	0.457603	-0.984574
C	1.610237	2.319194	1.045539
H	-0.171090	1.161901	1.386850
C	3.052050	1.481435	-0.743320
H	2.353503	-0.276311	-1.743927
C	2.773176	2.406156	0.266484
H	3.471977	3.208711	0.452616
C	-1.459386	-0.294354	-0.269335
C	-1.816824	0.847837	-0.997744
C	-2.417137	-0.952146	0.489523
C	-3.118588	1.341784	-0.964305
C	-3.745815	-0.498413	0.530478
H	-2.107802	-1.819568	1.058652
C	-4.070596	0.645726	-0.197486
O	0.205157	-1.678454	0.785543
B	0.513448	-3.028619	0.449256
N	0.862939	-2.999031	-1.121735
C	-1.060722	-2.194968	-2.406627
H	-1.935492	-1.572956	-2.226656
H	-0.829171	-2.139544	-3.475400
C	-1.264131	-3.658627	-2.002270
H	-1.812670	-3.723071	-1.057590
H	-1.805741	-4.238823	-2.753929
C	0.169448	-4.136139	-1.818259
H	0.283812	-5.039031	-1.218313
H	0.654984	-4.295143	-2.785646
B	2.435636	-3.062301	-1.464167
H	2.797950	-4.212772	-1.395597
H	2.619618	-2.554879	-2.550317
H	3.056951	-2.404676	-0.611200
O	1.786003	-3.556887	1.136875
C	2.898079	-2.958738	1.259958
C	4.106594	-3.838517	1.340693
H	4.348147	-3.983255	2.402435
H	3.909424	-4.802114	0.874362
H	4.962643	-3.352784	0.869650
C	4.343100	1.521025	-1.572767
C	1.266371	3.333108	2.145472
H	-0.321332	-3.853057	0.729571
C	3.017454	-1.540144	1.747286
H	4.076464	-1.269294	1.763718
H	2.504488	-0.863261	1.071845
C	2.385961	-1.399721	3.145326
H	2.815866	-2.098806	3.870064
H	2.553710	-0.383445	3.509989
H	1.310215	-1.566944	3.078039
C	5.227344	2.728203	-1.222332
H	6.129118	2.716144	-1.842084
H	4.710360	3.675743	-1.404897
H	5.545713	2.707820	-0.175274
C	5.147566	0.229895	-1.300762
H	5.414817	0.161198	-0.240699
H	4.577971	-0.665455	-1.562555
H	6.073956	0.227509	-1.885781

C	3.992765	1.597747	-3.074282
H	3.409184	2.498016	-3.291640
H	4.907183	1.628634	-3.676442
H	3.410596	0.731495	-3.398966
C	-0.086954	3.998167	1.807252
H	-0.362564	4.721720	2.582267
H	-0.027697	4.527475	0.850715
H	-0.891342	3.261577	1.730702
C	2.327137	4.436952	2.279637
H	3.307046	4.027211	2.544541
H	2.434726	5.009564	1.353135
H	2.035179	5.135387	3.069923
C	1.154777	2.600011	3.499461
H	0.887034	3.303427	4.295457
H	0.394020	1.815514	3.473257
H	2.109172	2.133453	3.765159
H	-1.051361	1.355177	-1.570437
H	-5.085239	1.021921	-0.168388
C	-4.771093	-1.268992	1.372838
C	-6.178558	-0.657673	1.288599
C	-4.323465	-1.259024	2.850413
H	-3.339379	-1.718319	2.975515
H	-4.265970	-0.234099	3.230478
H	-5.036548	-1.814506	3.469469
H	-6.197016	0.369789	1.665557
H	-6.873275	-1.245896	1.895998
H	-6.556738	-0.652863	0.261376
C	-3.531724	2.621006	-1.704570
C	-2.363472	3.242848	-2.486838
C	-4.027021	3.660097	-0.675010
H	-4.886490	3.290584	-0.108935
H	-3.234120	3.902727	0.039770
H	-4.328538	4.584705	-1.179429
H	-1.536649	3.519223	-1.825124
H	-2.702066	4.151010	-2.994985
H	-1.977224	2.559340	-3.249425
C	-4.666681	2.298587	-2.699546
H	-5.545392	1.889719	-2.193216
H	-4.977626	3.204355	-3.231467
H	-4.335225	1.563878	-3.440118
C	-4.844357	-2.727225	0.869666
H	-5.154313	-2.759856	-0.179726
H	-3.876843	-3.229983	0.949800
H	-5.569807	-3.298824	1.458843

C8-TS7S

C	-0.103225	-0.710459	-0.216615
C	0.120735	-1.237161	-1.702966
H	1.028800	-0.784304	-2.096506
C	0.911915	0.392575	0.104341
C	1.511740	0.420242	1.361097
C	1.257635	1.369075	-0.831020
C	2.469301	1.383828	1.688729
H	1.242526	-0.358818	2.062680
C	2.191720	2.369084	-0.533860
H	0.800535	1.351084	-1.814824
C	2.791679	2.353829	0.730293
H	3.526700	3.107793	0.971289
C	-1.529120	-0.218118	0.055085
C	-1.944287	1.057476	-0.349509
C	-2.443041	-1.058019	0.680730
C	-3.257144	1.484921	-0.161676
H	-1.223071	1.721394	-0.803083
C	-3.774843	-0.665714	0.886722
H	-2.094693	-2.025119	1.017549
C	-4.160418	0.602682	0.454664
H	-5.182752	0.926601	0.602369
O	0.139522	-1.843207	0.619324
B	0.897557	-2.813819	-0.080438
N	0.390181	-2.711448	-1.570492
C	-1.035694	-1.131680	-2.695635
H	-1.618065	-0.220067	-2.557485
H	-0.623018	-1.126034	-3.706120
C	-1.874596	-2.408188	-2.469606
H	-2.778820	-2.183174	-1.900227
H	-2.176631	-2.850744	-3.421350
C	-0.971340	-3.365284	-1.665213
H	-1.362096	-3.507982	-0.656348
H	-0.844576	-4.340794	-2.131485
B	1.332766	-3.267070	-2.752085
H	1.397463	-4.472332	-2.636890
H	0.876851	-2.921064	-3.818193
H	2.455394	-2.764105	-2.615985
O	2.358372	-2.210588	-0.179979
C	3.373762	-2.723098	-0.707248
C	4.480270	-1.782164	-1.070887
H	4.132725	-0.750725	-1.008469
H	5.316027	-1.932843	-0.376113
H	4.844576	-2.006700	-2.075799
C	-3.735678	2.876752	-0.597784

C	-4.742476	-1.643336	1.567754
C	2.531318	3.419052	-1.601463
C	3.157812	1.314943	3.059024
H	0.915459	-3.914209	0.394143
C	3.634113	-4.201260	-0.677992
H	2.725647	-4.737007	-0.946790
H	4.403024	-4.444810	-1.414955
C	4.091158	-4.596189	0.742748
H	4.307375	-5.666853	0.769987
H	4.996566	-4.061924	1.047006
H	3.304300	-4.384810	1.470639
C	-6.162497	-1.069764	1.696006
H	-6.595029	-0.837123	0.717726
H	-6.814133	-1.802666	2.181672
H	-6.177335	-0.158975	2.302811
C	-4.220865	-1.971559	2.983177
H	-4.891898	-2.678601	3.483399
H	-3.223608	-2.418184	2.951211
H	-4.161752	-1.064922	3.593442
C	-4.818656	-2.941994	0.735567
H	-3.840424	-3.422591	0.648405
H	-5.503150	-3.657764	1.203902
H	-5.182173	-2.733611	-0.275896
C	-2.613939	3.699616	-1.252643
H	-2.998011	4.682260	-1.543351
H	-1.774649	3.858077	-0.568914
H	-2.229974	3.213595	-2.155264
C	-4.883758	2.725613	-1.618678
H	-5.242041	3.709707	-1.940251
H	-4.544854	2.178549	-2.504167
H	-5.732731	2.182047	-1.195037
C	-4.244012	3.651523	0.637140
H	-5.074295	3.133576	1.125036
H	-3.444286	3.770573	1.374699
H	-4.594104	4.648078	0.346220
C	1.244976	4.171420	-2.006244
H	0.801770	4.674832	-1.141410
H	1.466976	4.928254	-2.766475
H	0.492040	3.494993	-2.419389
C	3.554570	4.452061	-1.102955
H	3.183910	4.999574	-0.230856
H	4.505944	3.982726	-0.833602
H	3.756898	5.181564	-1.893170
C	3.122336	2.711519	-2.840383
H	3.367076	3.441701	-3.619517
H	4.038772	2.173145	-2.577532
H	2.421169	1.989009	-3.267021
C	2.095175	1.362103	4.177172
H	1.386529	0.533976	4.097516
H	2.573548	1.302136	5.160927
H	1.525315	2.295294	4.130036
C	4.143772	2.472527	3.282793
H	4.953275	2.465586	2.546048
H	3.643152	3.444562	3.231794
H	4.598407	2.383692	4.274340
C	3.940086	-0.014568	3.148895
H	4.706664	-0.062812	2.368021
H	4.436366	-0.105038	4.121570
H	3.279440	-0.876462	3.021994

C8-TS7Spri

C	-0.007771	0.667364	-0.173484
C	-0.045949	1.324517	-1.596675
H	-1.093787	1.430736	-1.878541
C	-1.213598	-0.236995	0.065918
C	-1.719871	-0.346865	1.367113
C	-1.784518	-0.989603	-0.955386
C	-2.788265	-1.194136	1.651102
H	-1.260171	0.258364	2.135547
C	-2.851677	-1.867703	-0.705727
H	-1.396874	-0.904666	-1.964042
C	-3.336591	-1.948918	0.598342
H	-4.163507	-2.613191	0.814163
C	1.281963	-0.122160	0.092782
C	1.449190	-1.414680	-0.422409
C	2.319148	0.460098	0.811560
C	2.653220	-2.099406	-0.277692
H	0.618865	-1.884303	-0.930207
C	3.549337	-0.195723	0.976805
H	2.159936	1.440113	1.240078
C	3.697294	-1.464524	0.417222
H	4.638831	-1.987424	0.527259
O	-0.090573	1.775316	0.715194
B	0.371945	3.001217	0.142307
N	0.508310	2.715030	-1.423125
C	0.766782	0.706163	-2.733118
H	0.784639	-0.383814	-2.696829
H	0.317118	1.012004	-3.680223
C	2.171663	1.314097	-2.586401
H	2.805891	0.662355	-1.982458

H	2.654300	1.446096	-3.557838
C	1.959544	2.661618	-1.872103
H	2.608310	2.760495	-1.002723
H	2.131023	3.514920	-2.527247
B	-0.283133	3.735718	-2.394353
H	0.219377	4.829623	-2.294889
H	-0.266095	3.290161	-3.519781
H	-1.464066	3.774501	-2.007177
O	-0.676246	4.147760	0.312857
C	-1.870344	4.178297	-0.088918
C	-2.800248	3.003691	-0.013237
H	-2.304608	2.052154	-0.164359
H	-3.200286	2.993748	1.010092
H	-3.633753	3.128178	-0.705354
C	2.867937	-3.507469	-0.849735
C	4.671264	0.508973	1.751132
C	-3.427090	-2.693605	-1.864127
C	-3.379842	-1.329441	3.060839
H	1.360872	3.489620	0.622900
C	-2.489539	5.535131	-0.289278
H	-3.090279	5.485830	-1.203226
H	-3.215421	5.656939	0.530390
C	-1.498207	6.692243	-0.327019
H	-0.927675	6.750239	0.602528
H	-0.791316	6.555728	-1.147445
H	-2.029945	7.636736	-0.469205
C	4.983076	1.861861	1.074316
H	5.297734	1.712428	0.036316
H	4.112361	2.523014	1.070768
H	5.791155	2.377121	1.604988
C	5.965034	-0.319553	1.790541
H	6.735857	0.225982	2.343312
H	5.815359	-1.281045	2.291654
H	6.351274	-0.513309	0.784718
C	4.205840	0.758098	3.201763
H	3.985200	-0.188308	3.705544
H	4.986465	1.275453	3.770351
H	3.302437	1.372897	3.233520
C	4.031636	-3.470752	-1.863761
H	4.963376	-3.139077	-1.397277
H	4.203569	-4.466779	-2.286319
H	3.807114	-2.784072	-2.686154
C	3.215208	-4.479973	0.297572
H	3.379152	-5.489963	-0.093750
H	4.122343	-4.173743	0.825853
H	2.400907	-4.522569	1.027476
C	1.617747	-4.039984	-1.569667
H	0.757149	-4.102115	-0.896636
H	1.339408	-3.408690	-2.419663
H	1.813573	-5.045410	-1.954794
C	-3.951291	-1.741941	-2.961011
H	-4.746494	-1.099891	-2.569435
H	-3.159666	-1.095023	-3.349031
H	-4.356689	-2.315569	-3.801429
C	-2.311509	-3.587023	-2.450367
H	-1.474839	-2.994382	-2.830537
H	-1.918516	-4.267758	-1.688685
H	-2.698802	-4.187970	-3.280244
C	-4.584904	-3.600232	-1.417623
H	-5.420815	-3.019643	-1.014917
H	-4.958784	-4.170431	-2.273438
H	-4.266317	-4.317157	-0.654476
C	-2.651450	-0.443322	4.083925
H	-1.592652	-0.706623	4.165231
H	-2.718932	0.617000	3.821648
H	-3.104687	-0.571626	5.071737
C	-3.266533	-2.797452	3.525171
H	-3.685814	-2.913630	4.530536
H	-3.804498	-3.476677	2.858111
H	-2.219403	-3.114340	3.552346
C	-4.866363	-0.912947	3.030222
H	-5.448642	-1.539720	2.349038
H	-5.309471	-1.000206	4.028292
H	-4.970399	0.125715	2.700116

C8-B5-TS1R

C	-0.529893	-0.754378	-0.011268
C	-0.138013	-1.399726	-1.412185
H	0.821232	-0.989518	-1.724141
C	0.425036	0.400755	0.303935
C	0.920369	0.549713	1.597213
C	0.826463	1.303668	-0.682611
C	1.831750	1.563085	1.913047
H	0.616253	-0.179052	2.337346
C	1.709222	2.352213	-0.401413
H	0.458554	1.183447	-1.695771
C	2.205427	2.460611	0.903104
H	2.902387	3.252521	1.135734
C	-1.983193	-0.266851	0.063395
C	-2.371107	0.950461	-0.499021

C	-2.950786	-1.059687	0.677901	H	2.041760	1.800055	-3.109853
C	-3.702708	1.373778	-0.484605	H	3.022145	3.218027	-3.498940
H	-1.620439	1.589443	-0.944993	H	3.626866	1.997565	-2.360854
C	-4.296411	-0.674861	0.710371	C	0.828526	4.045672	-2.025452
H	-2.627339	-1.981062	1.143280	H	1.076975	4.748946	-2.827778
C	-4.653827	0.542643	0.119582	H	0.092014	3.338588	-2.418025
H	-5.688321	0.853132	0.136945	H	0.354429	4.605745	-1.213234
O	-0.365420	-1.799714	0.941995	C	3.105482	4.385938	-1.056889
B	0.436441	-2.850165	0.414222	H	3.350598	5.053388	-1.888596
N	0.079344	-2.860433	-1.134054	H	2.700740	4.999561	-0.246254
C	-1.163734	-1.360889	-2.543696	H	4.040092	3.933871	-0.709035
H	-1.735048	-0.432145	-2.555747	C	3.219316	0.318878	3.578270
H	-0.633357	-1.449843	-3.493736	H	2.582447	-0.564393	3.481547
C	-2.057065	-2.599538	-2.312356	H	4.035981	0.219653	2.856006
H	-3.015365	-2.311539	-1.875201	H	3.649285	0.320707	4.585989
H	-2.257196	-3.117944	-3.252746	C	3.375095	2.811952	3.520015
C	-1.279252	-3.496643	-1.327408	H	2.866093	3.767016	3.355503
H	-1.783393	-3.538032	-0.360878	H	3.761988	2.812928	4.543616
H	-1.130403	-4.513639	-1.686348	H	4.233690	2.759542	2.842974
B	1.135837	-3.512293	-2.135204	C	1.284940	1.736366	4.361974
H	1.143355	-4.710093	-1.973168	H	1.694355	1.764896	5.377615
H	0.912276	-3.166960	-3.272091	H	0.706183	2.651224	4.200353
H	2.259234	-3.050123	-1.833585	H	0.596180	0.890001	4.301769
O	1.902886	-2.337334	0.441210	C	-5.559732	3.040645	-1.025029
C	2.924487	-2.899316	-0.071131	H	-5.895515	3.094892	0.015199
C	4.052742	-1.998472	-0.389612	H	-5.764059	4.009535	-1.491075
C	5.270320	-2.485436	-0.890352	H	-6.165062	2.289027	-1.541492
C	3.903305	-0.620933	-0.155068	C	-3.662338	2.706273	-2.613293
C	6.319560	-1.609206	-1.150191	H	-2.589483	2.537414	-2.743181
H	5.398434	-3.543256	-1.087306	H	-4.193779	1.911732	-3.146696
C	4.959592	0.247897	-0.404389	H	-3.909536	3.662338	-3.087631
C	6.167726	-0.242334	-0.905161	C	-3.278448	3.846773	-0.400694
H	4.831795	1.306214	-0.203552	H	-3.542023	3.881550	0.660921
H	6.990323	0.437685	-1.104539	H	-2.197643	3.696440	-0.470966
C	3.146973	-4.377320	0.087237	H	-3.512630	4.820667	-0.844757
H	3.731478	-4.516163	1.006105	C	-6.750984	-1.043153	1.314023
H	2.199577	-4.899585	0.182196	H	-7.084913	-0.915222	0.279449
H	3.703493	-4.798020	-0.748196	H	-7.441203	-1.737991	1.802385
H	2.966708	-0.248626	0.233533	H	-6.836194	-0.078156	1.823373
H	7.256064	-1.991466	-1.543053	C	-5.300570	-2.974461	0.682797
C	2.098806	3.323055	-1.524932	H	-5.560264	-2.872874	-0.375939
C	2.424191	1.619982	3.327626	H	-4.314896	-3.443738	0.743308
C	-4.058682	2.725128	-1.120894	H	-6.023874	-3.652934	1.148348
C	-5.319842	-1.599300	1.385298	C	-4.941509	-1.778053	2.871318
C	2.733913	2.535825	-2.691885	H	-3.942566	-2.207583	2.983493

H	-4.951710	-0.815137	3.391537
H	-5.654017	-2.444674	3.369726
H	0.319834	-3.910818	0.963351

C8-B5-TS1Rpri

C	0.145049	0.755697	-0.098171
C	-0.248918	1.537735	-1.391011
H	-1.329258	1.445858	-1.510491
C	-0.714596	-0.481335	0.137471
C	-0.769254	-1.006526	1.429784
C	-1.361076	-1.150880	-0.897072
C	-1.473721	-2.179353	1.707396
H	-0.246422	-0.473593	2.215504
C	-2.070055	-2.337022	-0.664009
H	-1.313073	-0.752382	-1.903260
C	-2.117979	-2.830589	0.643504
H	-2.660317	-3.743787	0.839852
C	1.611772	0.296398	-0.057272
C	2.021448	-0.821131	-0.787821
C	2.547059	0.971751	0.722604
C	3.353378	-1.239532	-0.797807
H	1.280882	-1.387608	-1.339304
C	3.893617	0.586785	0.738521
H	2.205288	1.804039	1.323924
C	4.278553	-0.513694	-0.035647
H	5.313959	-0.821609	-0.034254
O	-0.092248	1.700227	0.946392
B	-0.077758	3.053159	0.486831
N	0.037313	2.980037	-1.081786
C	0.504005	1.257432	-2.690609
H	0.765711	0.205267	-2.807269
H	-0.135169	1.544529	-3.527051
C	1.751681	2.166905	-2.632387
H	2.640304	1.588239	-2.370408
H	1.929944	2.643026	-3.598602
C	1.463567	3.210842	-1.534118
H	2.140021	3.082768	-0.690531
H	1.536365	4.239767	-1.883076
B	-0.912417	3.966445	-1.960366
H	-0.721766	5.098240	-1.565954
H	-0.612894	3.825751	-3.126086
H	-2.069944	3.617568	-1.781930
O	-1.445340	3.827077	0.841362
C	-2.641622	3.816392	0.455649
C	-3.440155	2.594581	0.303364
C	-4.573912	2.586943	-0.532558
C	-3.116903	1.447872	1.045419
C	-5.355441	1.445378	-0.635155
H	-4.808297	3.459297	-1.131587
C	-3.920053	0.314154	0.954975
C	-5.034173	0.310887	0.118886
H	-3.653661	-0.573646	1.514442
H	-5.653562	-0.577926	0.048352
C	-3.276476	5.169868	0.312330
H	-2.920994	5.798242	1.132209
H	-2.901842	5.606986	-0.618178
H	-4.364725	5.138665	0.306493
H	-2.237127	1.454364	1.674165
H	-6.213582	1.434785	-1.299046
C	-2.735133	-3.052652	-1.847655
C	-1.500424	-2.717996	3.145059
C	3.738113	-2.474908	-1.624012
C	4.884896	1.380729	1.601010
C	3.383478	-2.227180	-3.106302
H	2.312767	-2.049082	-3.240229
H	3.919210	-1.354671	-3.493328
H	3.656450	-3.095431	-3.716061
C	5.238256	-2.796219	-1.534015
H	5.544909	-3.010211	-0.505444
H	5.463707	-3.680149	-2.138583
H	5.851710	-1.971201	-1.909595
C	2.946309	-3.695943	-1.106296
H	3.198498	-4.589582	-1.687887
H	3.181846	-3.892700	-0.055733
H	1.866533	-3.538885	-1.181258
C	4.874165	2.859507	1.156078
H	3.884489	3.309346	1.271804
H	5.579705	3.444375	1.756314
H	5.164193	2.949976	0.104288
C	6.322829	0.851471	1.480811
H	6.690562	0.908975	0.451408
H	6.990101	1.452714	2.105983
H	6.401945	-0.187659	1.815502
C	4.454726	1.288639	3.081009
H	3.448968	1.689362	3.232779
H	4.456310	0.248013	3.420633
H	5.143574	1.857336	3.715356
C	-3.725303	-2.094680	-2.543916
H	-3.230375	-1.192142	-2.911701
H	-4.197288	-2.590504	-3.398959

H	-4.511725	-1.779797	-1.852066
C	-1.639471	-3.475809	-2.851479
H	-1.088283	-2.611489	-3.232734
H	-0.919090	-4.149956	-2.377130
H	-2.082909	-3.995234	-3.707966
C	-3.505991	-4.309704	-1.414626
H	-4.302996	-4.069615	-0.703562
H	-3.969535	-4.777534	-2.288468
H	-2.846966	-5.051128	-0.952521
C	-2.103786	-1.649430	4.082083
H	-1.533984	-0.717059	4.052147
H	-3.137131	-1.420741	3.800509
H	-2.107502	-2.007693	5.117154
C	-2.336239	-4.001054	3.276936
H	-3.379167	-3.837068	2.987080
H	-1.933545	-4.812578	2.662936
H	-2.330279	-4.339101	4.317631
C	-0.054658	-3.030527	3.589010
H	0.396229	-3.783530	2.935177
H	0.577732	-2.139514	3.556058
H	-0.044305	-3.415449	4.614586
H	0.720800	3.783254	1.006357

C8-B5-TS1S

C	-0.281373	-0.522797	-0.078788
C	0.261907	-0.970403	-1.504902
H	0.895915	-0.177542	-1.895881
C	0.131868	0.926488	0.198069
C	0.599516	1.277848	1.461778
C	0.060373	1.907976	-0.792231
C	1.022886	2.579627	1.744362
H	0.667790	0.495070	2.205977
C	0.438934	3.232196	-0.540396
H	-0.290699	1.640168	-1.783159
C	0.926840	3.545224	0.733389
H	1.241834	4.557416	0.940786
C	-1.796250	-0.687874	0.082074
C	-2.687505	0.267274	-0.408890
C	-2.307509	-1.832156	0.692215
C	-4.071212	0.090894	-0.322551
H	-2.297881	1.172383	-0.855515
C	-3.686475	-2.048847	0.796217
H	-1.603454	-2.547080	1.096737
C	-4.552403	-1.077996	0.279408
H	-5.619230	-1.231069	0.352546
O	0.360505	-1.393897	0.852778
B	1.510092	-1.981535	0.273606
N	1.154706	-2.149519	-1.243107
C	-0.736909	-1.450381	-2.556640
H	-1.675873	-0.896124	-2.520849
H	-0.290181	-1.308125	-3.542600
C	-0.927189	-2.954657	-2.270835
H	-1.864667	-3.130625	-1.739167
H	-0.952886	-3.530457	-3.198561
C	0.265221	-3.367072	-1.383767
H	-0.077150	-3.672423	-0.393190
H	0.866406	-4.170589	-1.806290
B	2.355055	-2.244716	-2.316671
H	3.054786	-3.180287	-2.000514
H	1.867923	-2.363644	-3.421173
H	2.978300	-1.184097	-2.266273
O	2.606566	-0.803350	0.268331
C	3.708712	-0.554194	-0.280962
C	3.908930	0.873624	-0.711498
H	4.624489	1.355889	-0.035706
H	4.316597	0.925641	-1.722334
H	2.955232	1.398652	-0.669069
C	-4.994408	1.182116	-0.883495
C	-4.190508	-3.336034	1.464699
C	0.316437	4.271269	-1.663744
C	1.615769	2.887012	3.126442
H	1.926892	-2.955629	0.827087
C	-3.638312	-4.556009	0.695502
H	-3.977368	-4.544286	-0.345435
H	-2.545002	-4.568859	0.692216
H	-3.983137	-5.488139	1.156498
C	-5.724606	-3.430060	1.477369
H	-6.176170	-2.604858	2.036871
H	-6.139172	-3.424513	0.464321
H	-6.032778	-4.364057	1.957399
C	-3.689062	-3.381286	2.924106
H	-4.070639	-2.526223	3.490840
H	-4.029197	-4.298792	3.417055
H	-2.597419	-3.355446	2.975519
C	-4.712581	1.361707	-2.391188
H	-4.894863	0.429031	-2.934433
H	-5.362008	2.136710	-2.812909
H	-3.676074	1.659007	-2.574242
C	-6.481999	0.838053	-0.708587
H	-7.098328	1.642601	-1.121584

H	-6.747888	-0.086093	-1.231392
H	-6.748644	0.723334	0.346666
C	-4.713212	2.512351	-0.150838
H	-4.904740	2.411704	0.921973
H	-3.674485	2.830442	-0.275506
H	-5.358110	3.307209	-0.541743
C	-1.153821	4.342140	-2.132237
H	-1.811436	4.626353	-1.304661
H	-1.266965	5.084199	-2.930026
H	-1.502153	3.380985	-2.520830
C	0.744396	5.676373	-1.211085
H	0.630471	6.381757	-2.039925
H	0.131144	6.037048	-0.379513
H	1.792991	5.699215	-0.898219
C	1.212753	3.849239	-2.848227
H	2.261732	3.796105	-2.539070
H	0.930247	2.868521	-3.240695
H	1.134374	4.573289	-3.666540
C	1.974746	4.371648	3.297441
H	2.383001	4.539501	4.298863
H	2.730872	4.693369	2.574306
H	1.096570	5.015059	3.183473
C	2.903272	2.051172	3.302998
H	2.702087	0.980981	3.206061
H	3.644328	2.322213	2.543241
H	3.345989	2.228164	4.289481
C	0.600659	2.505704	4.224495
H	0.339805	1.445268	4.184658
H	1.016976	2.712329	5.216501
H	-0.323307	3.081830	4.114148
C	4.834328	-1.498224	-0.278202
C	4.810716	-2.606435	0.583846
C	5.963338	-1.275505	-1.083924
C	5.893892	-3.475969	0.633003
C	7.037730	-2.155406	-1.041839
C	7.005570	-3.256112	-0.182232
H	3.944542	-2.771074	1.210202
H	5.988728	-0.433877	-1.765535
H	5.870241	-4.327960	1.304380
H	7.898789	-1.987382	-1.680222
H	7.846867	-3.941419	-0.148967

C8-B5-TS1Spri

C	0.032539	-0.289559	-0.115383
C	0.706075	-0.577384	-1.500160
H	1.320769	0.289177	-1.746734
C	-0.207086	1.203267	0.096227
C	-0.127926	1.716662	1.392261
C	-0.564293	2.045298	-0.958786
C	-0.401029	3.062234	1.650068
H	0.160623	1.038544	2.182867
C	-0.867880	3.393125	-0.736521
H	-0.614937	1.644688	-1.962088
C	-0.775083	3.876047	0.572613
H	-1.002008	4.920092	0.760782
C	-1.290922	-1.044142	0.082959
C	-2.476102	-0.576095	-0.487456
C	-1.316333	-2.233383	0.808784
C	-3.668849	-1.296318	-0.390031
H	-2.470540	0.374679	-1.005321
C	-2.490064	-2.987635	0.926976
H	-0.402121	-2.564959	1.281676
C	-3.653079	-2.507836	0.313179
H	-4.565500	-3.080472	0.393664
O	0.994150	-0.735076	0.830993
B	1.884844	-1.737734	0.319963
N	1.642818	-1.733543	-1.266437
C	-0.160114	-0.995409	-2.687329
H	-1.124042	-0.485346	-2.710162
H	0.377733	-0.752057	-3.606485
C	-0.314283	-2.518639	-2.543154
H	-1.226247	-2.757050	-1.992716
H	-0.372077	-3.009660	-3.517659
C	0.920626	-2.980912	-1.747448
H	0.638802	-3.584835	-0.886072
H	1.620815	-3.554240	-2.354429
B	2.968359	-1.544903	-2.145967
H	3.690807	-2.500958	-1.997963
H	2.664395	-1.298580	-3.290711
H	3.565013	-0.543290	-1.685691
O	3.365882	-1.388029	0.569218
C	4.018672	-0.388798	0.113742
C	5.472295	-0.596960	-0.050482
C	6.354294	0.478750	-0.232603
C	5.978282	-1.906421	-0.012816
C	7.720041	0.248459	-0.369754
H	5.980660	1.495335	-0.264206
C	7.341988	-2.131952	-0.153386
C	8.215373	-1.055870	-0.331491
H	7.726769	-3.146298	-0.128186

H	9.280611	-1.233969	-0.442311
C	3.463732	1.007911	0.191861
H	2.379923	0.998825	0.222751
H	3.816015	1.439945	1.137708
H	3.821092	1.632313	-0.626231
H	5.283888	-2.726563	0.116870
H	8.397163	1.085175	-0.507433
C	-0.311111	3.664857	3.059057
C	0.731592	4.803387	3.060182
H	0.810076	5.249068	4.057800
H	1.718613	4.424407	2.776459
H	0.464493	5.598075	2.358027
C	-1.690298	4.229752	3.461723
H	-1.645977	4.665630	4.465771
H	-2.025588	5.009954	2.772677
H	-2.446246	3.438482	3.464665
C	0.109430	2.624578	4.109682
H	-0.607972	1.800991	4.172910
H	1.094108	2.201483	3.888541
H	0.162800	3.097309	5.095286
C	-1.307316	4.333403	-1.867659
C	-0.323954	5.519164	-1.964403
C	-1.345987	3.626804	-3.232677
C	-2.724956	4.862311	-1.559272
H	-0.283710	6.087969	-1.031458
H	0.688286	5.165894	-2.184834
H	-0.628590	6.204862	-2.762494
H	-2.055490	2.792985	-3.237336
H	-1.662755	4.334214	-4.004999
H	-0.362456	3.241186	-3.518809
H	-3.063482	5.536477	-2.353629
H	-3.438597	4.035404	-1.484604
H	-2.753915	5.413471	-0.615420
C	-4.934692	-0.728725	-1.047899
C	-5.254236	0.649513	-0.428433
C	-4.689492	-0.562344	-2.563500
C	-6.155628	-1.641913	-0.854632
H	-5.434663	0.558306	0.647109
H	-4.431131	1.356168	-0.568166
H	-6.149753	1.078481	-0.891536
H	-4.443214	-1.523782	-3.025152
H	-5.583972	-0.164779	-3.055469
H	-3.864242	0.126868	-2.764812
H	-7.030283	-1.197010	-1.339108
H	-5.996257	-2.629705	-1.298415
H	-6.395310	-1.777177	0.204602
C	-2.452954	-4.303322	1.717263
C	-1.399123	-5.242407	1.090401
C	-2.063751	-4.004108	3.181008
C	-3.807886	-5.028819	1.713939
H	-1.643020	-5.455563	0.044488
H	-0.398479	-4.803062	1.121029
H	-1.363483	-6.193376	1.633145
H	-2.798602	-3.341837	3.649508
H	-2.018903	-4.932014	3.761870
H	-1.086377	-3.518659	3.244943
H	-3.727992	-5.961300	2.281149
H	-4.592733	-4.424252	2.179262
H	-4.128230	-5.283687	0.698675
H	1.786364	-2.838588	0.799877

C8-TS4R

C	-0.295630	-0.731490	-0.084380
C	0.053611	-1.371088	-1.497991
H	0.981669	-0.928953	-1.855329
C	0.614374	0.469226	0.186569
C	1.127180	0.663473	1.462831
C	0.927878	1.387167	-0.823648
C	1.963536	1.751297	1.753127
H	0.882943	-0.070244	2.220395
C	1.740611	2.492066	-0.571444
H	0.539927	1.226408	-1.821292
C	2.252011	2.652106	0.726360
H	2.894260	3.499488	0.928959
C	-1.764151	-0.310709	0.062834
C	-2.233807	0.881808	-0.490171
C	-2.662050	-1.135335	0.737980
C	-3.578855	1.249841	-0.405925
H	-1.535646	1.544805	-0.983922
C	-4.019165	-0.806637	0.840540
H	-2.275748	-2.036490	1.194956
C	-4.459633	0.387543	0.258409
H	-5.503760	0.655023	0.330150
O	-0.034329	-1.761760	0.866827
B	0.757553	-2.789495	0.297573
N	0.335673	-2.822533	-1.223874
C	-1.024783	-1.373603	-2.580123
H	-1.630135	-0.466835	-2.565453
H	-0.537151	-1.445403	-3.553776
C	-1.859650	-2.644010	-2.305398

H	-2.811872	-2.390279	-1.834769
H	-2.072847	-3.178427	-3.233665
C	-1.010897	-3.501544	-1.344443
H	-1.471645	-3.543736	-0.356302
H	-0.845966	-4.518421	-1.696164
B	1.349942	-3.461285	-2.302750
H	1.401978	-4.655228	-2.097796
H	0.961305	-3.196747	-3.417362
H	2.463912	-2.953233	-2.132840
O	2.231895	-2.217859	0.234613
C	3.269697	-2.810447	-0.150743
C	4.467170	-1.931658	-0.479060
C	-4.024719	2.577650	-1.034976
C	-4.964035	-1.764902	1.579830
C	2.099513	3.513820	-1.658873
C	2.540450	1.893991	3.168137
H	0.732109	-3.841920	0.869851
C	-3.716148	2.553650	-2.547903
H	-4.028272	3.492724	-3.017954
H	-2.646943	2.423863	-2.739039
H	-4.246268	1.731863	-3.039650
C	-3.248504	3.738315	-0.374565
H	-3.451482	3.778033	0.700089
H	-2.168079	3.627810	-0.504348
H	-3.545246	4.696327	-0.815699
C	-5.528751	2.836184	-0.853555
H	-5.804688	2.889113	0.204187
H	-5.798031	3.791036	-1.315576
H	-6.133315	2.055883	-1.326504
C	-6.418918	-1.269140	1.583932
H	-7.051349	-1.986578	2.115934
H	-6.515977	-0.303044	2.089071
H	-6.814719	-1.166254	0.568583
C	-4.926195	-3.145758	0.889323
H	-5.248525	-3.066693	-0.153984
H	-3.919729	-3.572839	0.898188
H	-5.593125	-3.848594	1.400687
C	-4.496910	-1.911602	3.043951
H	-4.518163	-0.944241	3.555526
H	-5.152455	-2.601280	3.587157
H	-3.476351	-2.298724	3.103994
C	3.630424	3.526540	-1.859031
H	3.910953	4.254460	-2.628166
H	4.155386	3.792547	-0.937471
H	3.986163	2.540516	-2.174496
C	1.623404	4.914876	-1.218701
H	0.538687	4.925721	-1.071022
H	2.090827	5.225084	-0.280280
H	1.872813	5.660848	-1.981174
C	1.440796	3.186398	-3.008883
H	1.762743	2.213336	-3.392799
H	0.348368	3.181787	-2.935715
H	1.719736	3.943023	-3.748488
C	1.385276	1.957883	4.189840
H	0.740314	2.819340	3.990694
H	0.763398	1.059925	4.153784
H	1.780590	2.053412	5.207044
C	3.397851	3.159270	3.329336
H	3.778987	3.218912	4.353457
H	4.260090	3.154039	2.654960
H	2.817626	4.067151	3.136787
C	3.427136	0.666025	3.470423
H	4.266859	0.618076	2.769278
H	3.833713	0.724844	4.486076
H	2.862238	-0.266348	3.384558
C	3.423831	-4.290453	0.032557
H	3.581232	-4.463130	1.104968
H	2.520645	-4.813067	-0.270541
H	4.275825	-4.683498	-0.517023
C	5.305702	-2.510630	-1.633248
H	4.697502	-2.632994	-2.532286
H	6.121997	-1.817777	-1.856896
H	5.755116	-3.474956	-1.383249
C	4.009234	-0.508034	-0.827784
H	3.435856	-0.053614	-0.020423
H	4.886344	0.114543	-1.027278
H	3.383368	-0.515862	-1.723698
C	5.318274	-1.900746	0.819903
H	4.741155	-1.498623	1.656887
H	5.688713	-2.893852	1.090224
H	6.183480	-1.251516	0.657252

C8-TS4Rpri

C	0.208477	0.745024	-0.215792
C	-0.056974	1.464180	-1.576702
H	-1.118362	1.364106	-1.804545
C	-0.692707	-0.464441	0.012823
C	-0.808665	-0.944264	1.319213
C	-1.293498	-1.169300	-1.027839
C	-1.509808	-2.117386	1.602751

H	-0.319604	-0.384407	2.107574
C	-2.004770	-2.354650	-0.786397
H	-1.185685	-0.815452	-2.046510
C	-2.106413	-2.803839	0.533702
H	-2.644790	-3.718004	0.735221
C	1.661795	0.277674	-0.049693
C	2.092801	-0.907979	-0.648689
C	2.569252	1.029045	0.692450
C	3.420414	-1.330311	-0.559168
H	1.371393	-1.521031	-1.175665
C	3.911935	0.644816	0.800090
H	2.211775	1.919944	1.190892
C	4.319316	-0.531726	0.160776
H	5.351587	-0.840906	0.236388
O	-0.105567	1.735326	0.760395
B	0.040877	3.071891	0.261361
N	0.198373	2.924117	-1.316173
C	0.800161	1.111961	-2.790815
H	1.059123	0.052901	-2.830117
H	0.235906	1.360376	-3.691860
C	2.043343	2.013500	-2.672200
H	2.867554	1.467740	-2.208286
H	2.378061	2.356547	-3.653886
C	1.619977	3.192234	-1.773266
H	2.268877	3.275197	-0.902552
H	1.621384	4.148022	-2.295555
B	-0.799033	3.830348	-2.217622
H	-0.577950	4.994323	-1.974965
H	-0.630802	3.533056	-3.380028
H	-1.952761	3.539357	-1.889270
O	-1.237874	3.936885	0.524242
C	-2.428516	3.737975	0.163526
C	-3.288984	4.959259	0.056821
H	-4.100903	4.909544	0.790708
H	-2.684226	5.849770	0.219628
H	-3.743849	4.998681	-0.934977
C	-2.580767	-3.139291	-1.974240
C	-1.548218	-2.642532	3.045335
C	3.827524	-2.649161	-1.231075
C	4.874500	1.521984	1.612990
C	3.527911	-2.566198	-2.743572
H	2.463600	-2.403285	-2.935564
H	4.082077	-1.742654	-3.204845
H	3.818199	-3.496926	-3.243165
C	5.321344	-2.961888	-1.051321
H	5.588790	-3.064029	0.004982
H	5.563455	-3.906508	-1.548014
H	5.953063	-2.183498	-1.490771
C	3.009234	-3.803360	-0.610876
H	3.276898	-4.756916	-1.079421
H	3.204125	-3.882306	0.463261
H	1.934124	-3.652386	-0.743404
C	4.884821	2.948402	1.021196
H	3.894065	3.408998	1.060147
H	5.573539	3.589736	1.581953
H	5.208896	2.932141	-0.024434
C	6.313967	0.983467	1.598524
H	6.717948	0.935291	0.582278
H	6.960816	1.645606	2.182233
H	6.377718	-0.015877	2.040454
C	4.390919	1.580329	3.078103
H	3.381682	1.994074	3.151465
H	4.376764	0.579622	3.521506
H	5.058341	2.210771	3.675924
C	-3.556905	-2.250299	-2.772811
H	-3.075442	-1.337536	-3.134369
H	-3.939250	-2.792887	-3.644028
H	-4.410076	-1.956892	-2.153969
C	-1.413883	-3.570176	-2.891536
H	-0.870832	-2.706415	-3.285606
H	-0.700012	-4.193103	-2.343119
H	-1.788815	-4.147375	-3.743664
C	-3.337023	-4.401907	-1.530990
H	-4.179738	-4.158653	-0.876207
H	-3.736132	-4.919152	-2.408760
H	-2.683113	-5.102441	-1.002681
C	-2.141687	-1.569910	3.982389
H	-1.582505	-0.631990	3.932326
H	-3.181070	-1.355397	3.718251
H	-2.118862	-1.919490	5.020144
C	-2.392251	-3.919647	3.183539
H	-3.430239	-3.753156	2.878005
H	-1.986407	-4.742287	2.586632
H	-2.400844	-4.243507	4.228737
C	-0.102272	-2.963107	3.485739
H	0.344480	-3.716003	2.828781
H	0.533202	-2.074161	3.453110
H	-0.091699	-3.350745	4.510335
C	-3.061434	2.375952	0.141304
C	-3.475307	2.035541	1.602960

C	-4.253624	2.242123	-0.816254
H	-2.299121	1.647627	-0.124959
C	-4.115360	0.642708	1.666288
H	-4.185381	2.787222	1.972837
H	-2.585815	2.072580	2.237829
C	-4.837691	0.827600	-0.742797
H	-5.034799	2.965914	-0.550457
H	-3.926630	2.476446	-1.834352
C	-5.280580	0.487690	0.684176
H	-4.449870	0.451150	2.692159
H	-3.350775	-0.105469	1.436962
H	-5.676604	0.734533	-1.441331
H	-4.074117	0.108462	-1.062337
H	-5.670421	-0.535504	0.728317
H	-6.104874	1.153476	0.976860
H	0.902498	3.744867	0.763210

C8-TS4S

C	0.263240	0.872300	-0.353641
C	0.113796	1.432540	-1.831989
H	-0.703016	0.922824	-2.337048
C	-0.845434	-0.141000	-0.026141
C	-1.393666	-0.142433	1.256881
C	-1.289328	-1.080660	-0.958405
C	-2.404272	-1.038270	1.610752
H	-1.032126	0.600222	1.953077
C	-2.282887	-2.013280	-0.635759
H	-0.862496	-1.086541	-1.952556
C	-2.832684	-1.964008	0.648701
H	-3.616190	-2.667393	0.910004
C	1.613759	0.210485	-0.058924
C	1.923081	-1.047479	-0.593152
C	2.546400	0.851297	0.746987
C	3.150235	-1.656805	-0.347135
H	1.184003	-1.555036	-1.195356
C	3.796787	0.270767	1.017252
H	2.278031	1.811984	1.166132
C	4.077884	-0.975686	0.460048
H	5.035030	-1.441841	0.656059
O	0.134993	2.019105	0.483357
B	-0.647029	3.007330	-0.155805
N	-0.296354	2.885551	-1.684155
C	1.374063	1.468877	-2.694692
H	1.974271	0.562780	-2.606176
H	1.074198	1.588504	-3.739947
C	2.097426	2.725826	-2.203130
H	2.636966	2.509170	-1.278192
H	2.815728	3.113703	-2.930405
C	0.956823	3.715222	-1.952291
H	1.142911	4.376229	-1.105479
H	0.756029	4.326824	-2.831144
B	-1.404749	3.296711	-2.787516
H	-1.587427	4.492004	-2.696095
H	-0.968648	2.988618	-3.877097
H	-2.431057	2.662614	-2.571450
O	-2.161233	2.452280	-0.077233
C	-3.308032	2.576549	-0.551601
C	-4.012656	1.284930	-0.865963
H	-4.961824	1.424584	-1.379216
H	-3.348325	0.651996	-1.452935
H	-4.182305	0.758083	0.079697
C	3.507287	-3.035566	-0.919364
C	4.792892	1.028229	1.905919
C	-2.783186	-3.060863	-1.640500
C	-3.060998	-1.027249	2.998622
H	-0.589939	4.099589	0.318654
C	-4.089876	3.879938	-0.511861
C	-3.212952	5.072181	-0.114869
H	-3.839940	5.966109	-0.044629
H	-2.732835	4.912232	0.852593
H	-2.437495	5.249414	-0.860285
C	4.750386	-2.905210	-1.825269
H	5.026306	-3.880582	-2.240928
H	4.553167	-2.221738	-2.657212
H	5.612163	-2.519599	-1.273546
C	2.362065	-3.634444	-1.753782
H	1.453093	-3.764985	-1.157623
H	2.118308	-3.005912	-2.616407
H	2.656810	-4.617660	-2.133087
C	3.815362	-4.006593	0.240676
H	4.075486	-4.997059	-0.148618
H	4.652821	-3.655773	0.849805
H	2.945958	-4.113190	0.896828
C	6.114385	0.265173	2.088821
H	6.790392	0.845324	2.724490
H	5.958107	-0.705540	2.569849
H	6.620149	0.096733	1.132761
C	5.108594	2.395461	1.261281
H	5.551252	2.263020	0.268857

H	4.208050	3.005000	1.148280
H	5.817749	2.955817	1.880420
C	4.163654	1.250969	3.298028
H	3.934282	0.293617	3.776481
H	4.854637	1.801334	3.946101
H	3.234572	1.823266	3.232841
C	-4.289984	-2.842776	-1.896442
H	-4.671249	-3.588480	-2.602458
H	-4.871980	-2.925151	-0.974331
H	-4.471215	-1.849458	-2.319762
C	-2.557787	-4.472447	-1.057265
H	-1.494054	-4.646022	-0.866088
H	-3.093152	-4.611479	-0.114149
H	-2.908901	-5.236654	-1.759115
C	-2.048009	-2.977397	-2.988296
H	-2.193838	-2.006624	-3.472379
H	-0.972447	-3.146122	-2.873493
H	-2.433212	-3.745473	-3.665727
C	-2.449772	0.039080	3.921634
H	-1.381782	-0.132569	4.084878
H	-2.572631	1.046407	3.511907
H	-2.946343	0.011628	4.896526
C	-2.879389	-2.408157	3.664091
H	-3.340028	-2.417067	4.657899
H	-3.339372	-3.206332	3.074907
H	-1.817102	-2.645258	3.776709
C	-4.567712	-0.725469	2.845885
H	-5.066294	-1.459450	2.206655
H	-5.062974	-0.738347	3.822882
H	-4.721574	0.265422	2.404453
C	-4.794190	4.168020	-1.851616
H	-4.058278	4.346797	-2.635882
H	-5.446408	3.349100	-2.165401
H	-5.415444	5.061324	-1.735548
C	-5.159537	3.648159	0.595408
H	-5.863706	2.854507	0.333698
H	-4.692812	3.400255	1.554016
H	-5.726952	4.573943	0.725070

C8-TS4Spri

C	0.130661	-0.399161	-0.180709
C	0.591165	-0.861904	-1.606750
H	1.446261	-0.247901	-1.890727
C	0.452905	1.072695	0.063809
C	0.771818	1.482075	1.364318
C	0.390836	2.018516	-0.954340
C	1.032077	2.820147	1.650682
H	0.821662	0.720753	2.129836
C	0.626616	3.379750	-0.701786
H	0.149890	1.703134	-1.963025
C	0.947704	3.753668	0.601744
H	1.140692	4.796152	0.820058
C	-1.361635	-0.646071	0.083595
C	-2.333250	0.231382	-0.416208
C	-1.765893	-1.773469	0.788733
C	-3.692283	-0.033770	-0.266125
H	-2.011979	1.134958	-0.914328
C	-3.125754	-2.077043	0.959876
H	-1.005478	-2.420959	1.203115
C	-4.067686	-1.203176	0.417542
H	-5.121931	-1.419908	0.533716
O	0.915475	-1.186703	0.705696
B	1.373466	-2.411657	0.127071
N	1.096513	-2.272618	-1.441787
C	-0.430938	-0.928322	-2.740248
H	-1.162868	-0.120373	-2.698468
H	0.105710	-0.858000	-3.689143
C	-1.083649	-2.312187	-2.595149
H	-1.976303	-2.246203	-1.970468
H	-1.381199	-2.720352	-3.564242
C	-0.019535	-3.191402	-1.913650
H	-0.428208	-3.727706	-1.058089
H	0.420702	-3.917600	-2.595900
B	2.376057	-2.499060	-2.402931
H	2.711448	-3.657536	-2.332500
H	2.083731	-2.144410	-3.523254
H	3.287746	-1.767814	-1.983620
O	2.907819	-2.597361	0.316420
C	3.850315	-1.839293	-0.043589
C	3.747995	-0.344573	0.054814
H	2.776109	0.028092	-0.243635
H	3.860576	-0.091141	1.116402
H	4.537022	0.150469	-0.507669
C	-4.775016	0.903953	-0.817663
C	-3.512933	-3.350810	1.723234
C	0.517332	4.383943	-1.857166
C	1.402944	3.303371	3.059579
H	0.934519	-3.430917	0.593442
C	5.223786	-2.490865	-0.166034
C	5.089761	-4.017444	-0.251991

H	4.600820	-4.425367	0.634769
H	4.498251	-4.300897	-1.124331
H	6.085627	-4.462238	-0.336990
C	-2.888297	-4.574115	1.017036
H	-3.244943	-4.649650	-0.015371
H	-1.797029	-4.512309	0.993492
H	-3.159784	-5.497406	1.540418
C	-5.034649	-3.555610	1.788338
H	-5.259798	-4.476301	2.335295
H	-5.532430	-2.730964	2.308234
H	-5.473866	-3.646404	0.789735
C	-2.970602	-3.261327	3.165875
H	-3.402399	-2.402489	3.689361
H	-3.224679	-4.167804	3.726289
H	-1.883171	-3.150287	3.179830
C	-5.644115	0.133980	-1.835082
H	-6.131745	-0.731015	-1.377128
H	-6.426508	0.784873	-2.240522
H	-5.034228	-0.228533	-2.668686
C	-5.661500	1.402075	0.343975
H	-6.443686	2.071028	-0.031403
H	-6.150181	0.573148	0.863301
H	-5.063867	1.951201	1.078177
C	-4.177008	2.130955	-1.525925
H	-3.559611	2.729392	-0.849092
H	-3.560367	1.843221	-2.383428
H	-4.982566	2.771621	-1.897650
C	1.539252	4.014320	-2.953959
H	2.559988	4.051262	-2.560614
H	1.367979	3.007541	-3.344834
H	1.470108	4.715321	-3.792810
C	-0.909995	4.325480	-2.444737
H	-1.149126	3.330670	-2.830621
H	-1.654366	4.574889	-1.682132
H	-1.013359	5.037679	-3.270541
C	0.793360	5.826937	-1.406091
H	1.804609	5.938140	-1.002387
H	0.700630	6.504582	-2.260247
H	0.081142	6.155003	-0.642547
C	1.450026	2.150608	4.075219
H	0.480622	1.651707	4.166129
H	2.194814	1.397936	3.798324
H	1.719654	2.539376	5.062053
C	0.354802	4.329860	3.540504
H	0.604471	4.688470	4.545049
H	0.304684	5.198341	2.877916
H	-0.641088	3.877591	3.575865
C	2.794964	3.970119	3.019679
H	2.814574	4.827956	2.341801
H	3.078219	4.323871	4.017001
H	3.555114	3.258833	2.680805
C	6.000109	-1.969073	-1.390197
H	5.479506	-2.224158	-2.314654
H	6.148452	-0.886518	-1.363102
H	6.989484	-2.435856	-1.403915
C	5.991513	-2.108595	1.128933
H	6.177086	-1.033203	1.195395
H	5.444715	-2.426538	2.021831
H	6.959384	-2.617766	1.124792

B5-TS1R

C	-1.050678	-0.965683	-0.038126
C	-0.475485	-0.603937	1.393570
H	0.552689	-0.952082	1.446977
C	-0.164250	-2.011010	-0.718314
C	0.052855	-1.926297	-2.094500
C	0.430389	-3.055740	-0.007623
C	0.859445	-2.856329	-2.742496
H	-0.398673	-1.109270	-2.640225
C	1.232399	-3.993234	-0.655648
H	0.289269	-3.138071	1.063675
C	1.453428	-3.894520	-2.026078
H	1.027546	-2.769252	-3.809944
H	1.690763	-4.792867	-0.085110
H	2.081574	-4.618493	-2.532162
C	-2.506550	-1.459673	-0.031821
C	-2.841560	-2.756466	0.368379
C	-3.531878	-0.602190	-0.434568
C	-4.169314	-3.170987	0.407239
H	-2.063018	-3.458544	0.637573
C	-4.861087	-1.017400	-0.403075
H	-3.272814	0.386482	-0.785649
C	-5.187511	-2.299855	0.027187
H	-4.406452	-4.179878	0.725169
H	-5.641606	-0.335243	-0.720833
H	-6.221739	-2.622959	0.053573
O	-1.011949	0.249547	-0.768955
B	-0.272967	1.269911	-0.101787
N	-0.433435	0.901846	1.443038

C	-1.279339	-1.016851	2.622280
H	-1.759143	-1.985429	2.498926
H	-0.605241	-1.077665	3.475789
C	-2.302604	0.121024	2.824799
H	-3.296613	-0.184679	2.497482
H	-2.364806	0.403729	3.875928
C	-1.802662	1.293205	1.958048
H	-2.458714	1.458028	1.106250
H	-1.710874	2.224149	2.509861
B	0.692224	1.411974	2.449678
H	0.621893	2.606802	2.572475
H	0.643438	0.807754	3.490864
H	1.793020	1.122668	1.932846
O	1.243749	0.884525	-0.361150
C	2.296559	1.317329	0.206063
C	3.423467	0.354182	0.272406
C	4.631166	0.670061	0.908783
C	3.272418	-0.901725	-0.330858
C	5.665389	-0.256003	0.944279
H	4.760037	1.627423	1.395394
C	4.311836	-1.821809	-0.298650
C	5.508687	-1.502712	0.339345
H	4.179691	-2.787023	-0.771359
H	6.318328	-2.222824	0.367751
C	2.571554	2.789133	0.313748
H	3.068789	3.080838	-0.619155
H	1.652987	3.355792	0.406895
H	3.231105	3.022119	1.145612
H	2.343394	-1.139769	-0.826388
H	6.592922	-0.008020	1.446156
C	-0.645342	2.727785	-0.622784
C	-0.860847	2.883929	-2.001065
C	-0.779178	3.868503	0.180362
C	-1.182993	4.119775	-2.554621
H	-0.780993	2.015433	-2.645116
C	-1.105933	5.109382	-0.364140
H	-0.614207	3.795632	1.248370
C	-1.306380	5.240181	-1.735435
H	-1.341816	4.209743	-3.623847
H	-1.203999	5.973584	0.283913
H	-1.559368	6.204544	-2.161684

B5-TS1Rpri

C	-0.213874	1.049117	0.096562
C	-0.209145	0.347309	1.488577
H	-1.103526	-0.268243	1.549164
C	-1.615426	1.367244	-0.412112
C	-1.773037	1.647526	-1.773238
C	-2.718426	1.481290	0.430437
C	-3.008128	2.029758	-2.279094
H	-0.913484	1.565949	-2.426359
C	-3.959881	1.867357	-0.075458
H	-2.626672	1.268312	1.487610
C	-4.109062	2.144971	-1.428858
H	-3.113831	2.245831	-3.336285
H	-4.808571	1.948199	0.593854
H	-5.072349	2.449819	-1.821213
C	0.600742	2.353344	0.042636
C	0.067492	3.543364	0.550985
C	1.876539	2.384297	-0.519560
C	0.811880	4.717712	0.546802
H	-0.944160	3.559373	0.936105
C	2.621565	3.561895	-0.531212
H	2.291134	1.486832	-0.952358
C	2.098333	4.730904	0.011204
H	0.381466	5.626476	0.951720
H	3.613040	3.559836	-0.969529
H	2.678242	5.646462	0.004217
O	0.390997	0.072916	-0.762662
B	1.144130	-0.891416	-0.072197
N	0.963171	-0.598372	1.456197
C	-0.000863	1.192934	2.739844
H	-0.484208	2.165865	2.670781
H	-0.424829	0.661346	3.590611
C	1.531002	1.310462	2.888499
H	1.879175	2.285112	2.545742
H	1.828962	1.191269	3.929911
C	2.133973	0.201807	2.005454
H	2.709991	0.621812	1.184942
H	2.773735	-0.483379	2.554182
B	0.702397	-1.828770	2.509256
H	1.555091	-2.663215	2.332063
H	0.748439	-1.358205	3.621078
H	-0.411689	-2.267078	2.290169
O	0.364715	-2.518627	-0.259465
C	-0.656565	-3.076013	0.166936
C	-2.000948	-2.492036	-0.021605
C	-3.049842	-2.738121	0.874776
C	-2.241058	-1.745975	-1.182035

C	-4.317647	-2.237694	0.614106
H	-2.860922	-3.282798	1.790615
C	-3.522349	-1.288521	-1.459608
C	-4.557757	-1.526915	-0.562117
H	-3.705114	-0.717926	-2.359376
H	-5.550511	-1.146834	-0.771577
C	-0.534368	-4.479805	0.681734
H	-0.813167	-5.159821	-0.131654
H	0.493664	-4.665699	0.981733
H	-1.212590	-4.667530	1.512760
H	-1.419609	-1.540282	-1.854103
H	-5.120172	-2.405057	1.322474
C	2.552769	-1.276358	-0.678410
C	2.901858	-0.815075	-1.954717
C	3.479109	-2.082481	-0.001831
C	4.129649	-1.131945	-2.532580
H	2.194314	-0.201491	-2.501205
C	4.710057	-2.397943	-0.569003
H	3.227243	-2.472687	0.976796
C	5.040665	-1.921798	-1.836835
H	4.374523	-0.763445	-3.522861
H	5.412717	-3.019109	-0.024169
H	5.999601	-2.168132	-2.279168

B5-TS1S

C	1.846244	0.135618	-0.009082
C	1.253543	0.294637	1.452269
H	1.011631	1.341339	1.609853
C	2.196098	1.504541	-0.597437
C	2.000258	1.728351	-1.960753
C	2.733470	2.532069	0.180902
C	2.326483	2.953689	-2.531626
H	1.573892	0.935312	-2.559594
C	3.065578	3.759897	-0.389894
H	2.895278	2.387109	1.242942
C	2.860958	3.975842	-1.748983
H	2.161776	3.112789	-3.591425
H	3.480908	4.545868	0.230689
H	3.114486	4.930818	-2.194475
C	3.066261	-0.792821	-0.094289
C	4.341229	-0.365313	0.287507
C	2.912600	-2.102138	-0.553367
C	5.426768	-1.234839	0.247245
H	4.496029	0.657541	0.606029
C	3.999531	-2.971902	-0.601085
H	1.936743	-2.425182	-0.886809
C	5.259767	-2.545687	-0.193035
H	6.406274	-0.883473	0.551040
H	3.858585	-3.984207	-0.962883
H	6.105703	-3.222329	-0.229012
O	0.801046	-0.458208	-0.767922
B	-0.439269	-0.437717	-0.087363
N	-0.052179	-0.456981	1.445946
C	2.042484	-0.283694	2.623360
H	3.117257	-0.188569	2.483420
H	1.767404	0.255587	3.528908
C	1.592716	-1.757124	2.720191
H	2.362390	-2.426846	2.335786
H	1.392195	-2.032762	3.755676
C	0.322003	-1.867463	1.856448
H	0.510174	-2.453608	0.958958
H	-0.522257	-2.304079	2.382264
B	-1.068336	0.157692	2.529625
H	-2.063489	-0.515956	2.577218
H	-0.510186	0.244528	3.596559
H	-1.353699	1.298314	2.162427
O	-0.919288	1.132818	-0.262621
C	-1.829534	1.788348	0.303461
C	-3.238340	1.337563	0.309102
C	-4.097103	1.611158	1.379736
C	-3.732707	0.697255	-0.831970
C	-5.427442	1.224477	1.317069
H	-3.703816	2.070496	2.277367
C	-5.072551	0.332855	-0.899461
H	-3.068196	0.489149	-1.657950
C	-5.918022	0.588157	0.175136
H	-6.082584	1.409671	2.159716
H	-5.444566	-0.169124	-1.783715
H	-6.958452	0.288024	0.129486
C	-1.535076	3.229324	0.602311
H	-2.125895	3.597076	1.439618
H	-0.470385	3.368018	0.780652
H	-1.821916	3.805677	-0.285432
C	-1.498415	-1.465134	-0.663542
C	-2.524493	-2.054955	0.085191
C	-1.425333	-1.784990	-2.028581
C	-3.448559	-2.914621	-0.503364
H	-2.620592	-1.818576	1.136637
C	-2.352802	-2.633045	-2.627521

H	-0.626885	-1.356172	-2.623567
C	-3.372108	-3.199980	-1.863847
H	-4.236247	-3.352816	0.099623
H	-2.278288	-2.858164	-3.686030
H	-4.095095	-3.863796	-2.325110

B5-TS1Spri

C	-1.624449	-0.546231	-0.009268
C	-0.869103	-0.655327	1.357872
H	-0.343734	-1.606135	1.367788
C	-1.959585	-1.928090	-0.571348
C	-2.006071	-2.094604	-1.957660
C	-2.240334	-3.023123	0.248143
C	-2.316864	-3.331612	-2.510925
H	-1.776211	-1.247943	-2.590399
C	-2.555819	-4.263186	-0.305646
H	-2.207697	-2.922648	1.326446
C	-2.593518	-4.421750	-1.686995
H	-2.342610	-3.446449	-3.588637
H	-2.767355	-5.103725	0.345409
H	-2.835087	-5.385829	-2.119193
C	-2.911603	0.291642	0.070254
C	-4.104073	-0.260178	0.550682
C	-2.910240	1.631821	-0.316640
C	-5.249691	0.518386	0.681981
H	-4.148154	-1.310207	0.809713
C	-4.057142	2.412203	-0.190774
H	-2.010123	2.066018	-0.722336
C	-5.229946	1.862660	0.317652
H	-6.161601	0.070027	1.059640
H	-4.029204	3.452140	-0.495927
H	-6.122643	2.469319	0.416366
O	-0.687661	0.077461	-0.877151
B	0.434122	0.694199	-0.232267
N	0.179935	0.424080	1.336888
C	-1.644256	-0.450820	2.656275
H	-2.666956	-0.817359	2.590992
H	-1.137824	-0.996662	3.451962
C	-1.587568	1.065717	2.924118
H	-2.530267	1.541218	2.652614
H	-1.400784	1.268112	3.979035
C	-0.455088	1.612731	2.036546
H	-0.842180	2.297499	1.287779
H	0.320731	2.128037	2.597243
B	1.451236	-0.053266	2.173608
H	2.303395	0.795262	2.162729
H	1.116918	-0.436848	3.266482
H	1.912150	-1.050065	1.562118
O	1.801445	-0.009940	-0.563483
C	2.294272	-1.115974	-0.172383
C	3.766074	-1.136193	-0.001239
C	4.466450	-2.327510	0.221514
C	4.468390	0.073890	-0.072557
C	5.847693	-2.308562	0.373049
H	3.939243	-3.270332	0.283250
C	5.847750	0.088060	0.077341
C	6.540016	-1.101508	0.302024
H	6.385674	1.026809	0.021034
H	7.617144	-1.087986	0.421620
C	1.554974	-2.405299	-0.409234
H	0.490233	-2.233968	-0.513468
H	1.918434	-2.806057	-1.362225
H	1.744056	-3.144026	0.367126
H	3.915502	0.986269	-0.248065
H	6.383719	-3.233776	0.547211
C	0.733809	2.198713	-0.690425
C	0.134931	2.716554	-1.845098
C	1.649993	3.019643	-0.015807
C	0.419280	4.003212	-2.301537
H	-0.559527	2.093558	-2.397562
C	1.935544	4.308100	-0.458792
H	2.146787	2.644249	0.872191
C	1.317709	4.806214	-1.605123
H	-0.058466	4.376751	-3.200957
H	2.641556	4.924777	0.087281
H	1.539056	5.809083	-1.953104

B5-C12-TS1R

C	-0.374806	2.150086	1.735211
C	0.139889	2.348994	0.287994
H	1.187481	2.056397	0.206244
O	-1.098551	0.936568	1.774838
B	-0.981466	0.233942	0.544442
N	-0.671556	1.376404	-0.534829
C	-0.126561	3.737745	-0.308599
H	-0.163292	4.495750	0.479313
H	0.671454	4.017525	-0.998030
C	-1.474009	3.602073	-1.064866

H	-2.228302	4.307368	-0.706854
H	-1.325632	3.785956	-2.130491
C	-1.929117	2.154054	-0.829780
H	-2.590028	2.079413	0.037295
H	-2.432421	1.711643	-1.687098
B	0.083391	0.999235	-1.888692
H	-0.642874	0.345191	-2.597657
H	0.517738	1.995807	-2.417846
H	1.074367	0.316080	-1.567700
O	0.453864	-0.479899	0.635335
C	1.146625	-1.039896	-0.270298
C	2.617477	-0.955983	-0.093663
C	3.506183	-1.616197	-0.954927
C	3.129141	-0.199935	0.973672
C	4.880010	-1.517466	-0.754583
H	3.130725	-2.199426	-1.786937
C	4.501553	-0.105057	1.171902
C	5.380260	-0.761996	0.307074
H	4.888701	0.478513	2.000991
H	6.452212	-0.685852	0.460652
C	0.556958	-2.079906	-1.179671
H	0.704386	-3.051917	-0.691006
H	-0.507553	-1.920091	-1.315090
H	1.049852	-2.097857	-2.150042
H	2.433390	0.293458	1.641349
H	5.560572	-2.028159	-1.428005
C	-2.171063	-0.795513	0.300074
C	-2.669397	-1.483899	1.419969
C	-2.770922	-1.067600	-0.940256
C	-3.707362	-2.407749	1.308755
H	-2.238731	-1.276037	2.394929
C	-3.814268	-1.987756	-1.061382
H	-2.410645	-0.562993	-1.831090
C	-4.284677	-2.663841	0.063746
H	-4.070307	-2.925292	2.192591
H	-4.259415	-2.174821	-2.034756
H	-5.095420	-3.381104	-0.027140
H	0.465827	2.133210	2.442328
H	-1.038795	2.972228	2.035498

B5-C12-TS1Rpri

C	-0.373770	1.642874	-1.470311
C	-0.645945	1.898797	0.015772
H	-1.609711	1.474372	0.297029
O	0.221654	0.359083	-1.556265
B	0.762922	-0.062192	-0.307195
N	0.425045	1.107587	0.719787
C	-0.486721	3.353343	0.473912
H	-0.683166	4.043335	-0.353026
H	-1.198943	3.580422	1.268718
C	0.968399	3.459178	1.001141
H	1.546395	4.223329	0.474663
H	0.965515	3.714132	2.061336
C	1.585033	2.070538	0.781050
H	2.134339	2.031852	-0.162954
H	2.260139	1.749916	1.573223
B	-0.038021	0.726486	2.229712
H	0.770911	-0.050537	2.690929
H	-0.112264	1.752982	2.868422
H	-1.147297	0.219118	2.146574
O	-0.030433	-1.381080	0.289164
C	-1.175178	-1.642754	0.721467
C	-2.408717	-1.155790	0.084270
C	-3.581683	-0.994841	0.842262
C	-2.428676	-0.894780	-1.295822
C	-4.746214	-0.541804	0.235099
H	-3.562287	-1.177392	1.910657
C	-3.607371	-0.467660	-1.901235
C	-4.761713	-0.283255	-1.138827
H	-3.623422	-0.276868	-2.969321
H	-5.675905	0.059526	-1.613529
C	-1.233071	-2.611470	1.867528
H	-0.474951	-3.380030	1.700579
H	-0.950813	-2.064070	2.771919
H	-2.213576	-3.068454	1.994211
H	-1.518697	-1.013813	-1.869549
H	-5.641979	-0.391173	0.828374
C	2.266774	-0.580082	-0.355981
C	3.029961	-0.378572	-1.515361
C	2.891338	-1.205908	0.735453
C	4.364590	-0.781282	-1.588626
H	2.558699	0.096642	-2.370933
C	4.224892	-1.607700	0.673540
H	2.322128	-1.371741	1.644699
C	4.966484	-1.396210	-0.490980
H	4.933526	-0.617214	-2.499871
H	4.688163	-2.086190	1.532231
H	6.005386	-1.710140	-0.541520
H	0.308124	2.398540	-1.887889
H	-1.300375	1.669107	-2.055358

B5-C12-TS1S

C	-3.141572	0.358120	-1.466519
C	-3.077051	-0.626303	-0.277002
H	-2.872378	-1.638699	-0.625468
O	-1.874288	0.987677	-1.566757
B	-0.988533	0.501778	-0.594555
N	-1.879226	-0.169735	0.518967
C	-4.273461	-0.558267	0.677594
H	-5.186612	-0.288643	0.138458
H	-4.438874	-1.529087	1.146234
C	-3.877647	0.496162	1.743958
H	-4.550468	1.357660	1.744590
H	-3.904839	0.052127	2.739995
C	-2.447019	0.933296	1.384643
H	-2.452202	1.863669	0.810826
H	-1.799372	1.066112	2.249086
B	-1.324484	-1.355737	1.479313
H	-0.419329	-0.918861	2.149570
H	-2.246937	-1.738678	2.164937
H	-0.947815	-2.275993	0.771262
O	-0.392076	-0.961642	-1.387637
C	0.379221	-1.852732	-0.975353
C	1.667355	-1.546832	-0.319128
C	2.125855	-2.282894	0.781849
C	2.466364	-0.532700	-0.865388
C	3.360548	-1.982611	1.347091
H	1.487531	-3.037906	1.226209
C	3.713435	-0.257353	-0.314039
H	2.102858	0.036283	-1.711260
C	4.157286	-0.975232	0.796221
H	3.700762	-2.529769	2.220249
H	4.321993	0.534702	-0.736207
H	5.122257	-0.747322	1.238505
C	0.109789	-3.271397	-1.389260
H	0.393687	-3.972240	-0.602977
H	-0.939441	-3.397382	-1.654040
H	0.736096	-3.490406	-2.263702
C	0.209167	1.460654	-0.223213
C	0.878216	1.455938	1.010268
C	0.644071	2.378207	-1.196287
C	1.945669	2.318239	1.259111
H	0.579659	0.746578	1.773332
C	1.720336	3.232284	-0.961477
H	0.125056	2.414253	-2.149589
C	2.377173	3.202835	0.270990
H	2.449949	2.288907	2.220820
H	2.042308	3.926574	-1.732868
H	3.213922	3.869599	0.460091
H	-3.385338	-0.166472	-2.397881
H	-3.908318	1.128162	-1.305931

B5-C12-TS1Spri

C	2.093625	-1.961555	1.378463
C	1.476553	-2.455686	0.067419
H	0.586311	-3.051151	0.277093
O	1.311455	-0.856991	1.781423
B	0.880522	-0.114444	0.622119
N	1.019556	-1.182996	-0.609265
C	2.442716	-3.170613	-0.895793
H	3.394302	-3.369307	-0.390807
H	2.041751	-4.131253	-1.224515
C	2.630755	-2.199000	-2.083932
H	3.667779	-2.148154	-2.426655
H	2.009024	-2.506963	-2.925779
C	2.156144	-0.852526	-1.541614
H	2.950260	-0.357219	-0.977874
H	1.803178	-0.159401	-2.303926
B	-0.316520	-1.441738	-1.459959
H	-0.619348	-0.424028	-2.040567
H	-0.169099	-2.410886	-2.168775
H	-1.215196	-1.716272	-0.647248
O	-0.623874	0.250768	0.702709
C	-1.634310	-0.508255	0.834931
C	-2.903188	0.008126	0.293514
C	-4.123132	-0.642316	0.535356
C	-2.885965	1.184130	-0.475032
C	-5.305735	-0.125409	0.016233
H	-4.152325	-1.551603	1.124009
C	-4.069703	1.697165	-0.989934
C	-5.280525	1.043444	-0.746934
H	-4.051369	2.605269	-1.583541
H	-6.204230	1.444465	-1.152633
C	-1.631029	-1.652933	1.809871
H	-0.609834	-1.920084	2.063510
H	-2.128067	-1.300660	2.722901
H	-2.183428	-2.510860	1.427930
H	-1.938246	1.676298	-0.651623
H	-6.245883	-0.633144	0.205016
C	1.609931	1.286168	0.379011

C	2.681909	1.675819	1.192601
C	1.219146	2.163089	-0.647138
C	3.344859	2.888899	0.993137
H	2.986851	1.016582	2.000602
C	1.872956	3.376182	-0.855623
H	0.396965	1.880383	-1.300382
C	2.941443	3.742451	-0.033326
H	4.172213	3.169832	1.639300
H	1.553362	4.036945	-1.657033
H	3.453673	4.687188	-0.192306
H	2.081773	-2.732966	2.156871
H	3.143326	-1.664418	1.220175

C12h-TS1S

C	-2.942958	-1.181424	-0.894642
C	-2.621771	-0.446405	0.430044
H	-2.251275	-1.144435	1.179140
O	-1.838316	-0.996373	-1.770533
B	-0.803193	-0.296374	-1.123556
N	-1.492409	0.479941	0.066299
C	-3.746138	0.448487	0.961985
H	-4.724999	0.043901	0.695264
H	-3.698651	0.510509	2.048510
C	-3.485427	1.840933	0.329623
H	-4.314963	2.172282	-0.297208
H	-3.339915	2.586283	1.110730
C	-2.206746	1.678004	-0.507783
H	-2.446983	1.471833	-1.552224
H	-1.539563	2.534996	-0.463994
B	-0.610878	0.943243	1.323703
H	0.186489	1.762605	0.938899
H	-1.342848	1.358630	2.193047
H	-0.027790	-0.045501	1.766071
O	0.055159	-1.444256	-0.318122
C	1.027263	-1.273383	0.455804
C	2.144655	-0.358710	0.143612
C	2.877703	0.270846	1.156507
C	2.527097	-0.191860	-1.192447
C	3.964488	1.070258	0.835222
H	2.557129	0.182232	2.186057
C	3.630359	0.592154	-1.508051
H	1.966837	-0.688529	-1.972003
C	4.345762	1.228606	-0.497264
H	4.510340	1.578343	1.620823
H	3.926696	0.710448	-2.543225
H	5.197654	1.850704	-0.745695
C	1.209525	-2.284659	1.548315
H	1.701673	-1.856208	2.419281
H	0.249126	-2.715135	1.823448
H	1.860159	-3.076052	1.157452
H	-3.117087	-2.247483	-0.716135
H	-3.841258	-0.768881	-1.368521
H	-0.071151	0.353463	-1.800847

C12h-TS1Spri

C	-3.011889	1.507185	0.312368
C	-2.695609	0.390759	-0.688112
H	-2.140119	0.799648	-1.531287
O	-1.822014	1.707730	1.045248
B	-1.203613	0.429011	1.284330
N	-1.753517	-0.512802	0.077268
C	-3.888216	-0.472461	-1.139260
H	-4.822480	-0.037845	-0.772392
H	-3.956289	-0.523589	-2.225740
C	-3.643265	-1.868371	-0.521655
H	-4.559206	-2.329220	-0.147139
H	-3.204479	-2.536663	-1.262324
C	-2.645098	-1.602316	0.602036
H	-3.153957	-1.241193	1.498474
H	-2.035742	-2.461083	0.875840
B	-0.638785	-1.124317	-0.889362
H	-0.015790	-1.974424	-0.303109
H	-1.136021	-1.476341	-1.932177
H	0.148651	-0.196994	-1.160698
O	0.323633	0.485717	1.158288
C	1.004162	0.852897	0.147278
C	2.345452	0.250362	0.024570
C	3.302439	0.760915	-0.861624
C	2.667700	-0.855755	0.823417
C	4.560522	0.177208	-0.944728
H	3.071690	1.612987	-1.486605
C	3.923788	-1.436771	0.735986
C	4.872445	-0.922085	-0.147719
H	4.165080	-2.294621	1.351936
H	5.852828	-1.378973	-0.216676
C	0.669534	2.111086	-0.603054
H	-0.380917	2.348922	-0.479395
H	1.253100	2.919010	-0.144992
H	0.940146	2.039604	-1.654163
H	1.916738	-1.246378	1.495095

H	5.296538	0.577791	-1.631218
H	-3.302812	2.436607	-0.184880
H	-3.838305	1.207579	0.975242
H	-1.396792	-0.082167	2.355171

h-TS7R

C	0.645044	0.110558	-0.198506
C	0.068538	-0.800312	0.969862
H	-0.710186	-0.243520	1.487957
C	0.148116	1.551521	-0.042825
C	-0.183822	2.284729	-1.186076
C	0.032099	2.168204	1.207760
C	-0.634114	3.599366	-1.080583
H	-0.103344	1.800141	-2.151487
C	-0.413741	3.486478	1.315899
H	0.278311	1.620282	2.112396
C	-0.752532	4.206452	0.171476
H	-1.105590	5.229873	0.253422
C	2.176869	0.099947	-0.307098
C	2.980047	0.828055	0.579684
C	2.801030	-0.657234	-1.303171
C	4.370058	0.771812	0.495647
H	2.520377	1.456639	1.334035
C	4.192440	-0.709659	-1.393026
H	2.182321	-1.189037	-2.014370
C	4.984008	-0.002224	-0.489537
H	6.066774	-0.043827	-0.557921
O	0.119444	-0.447101	-1.401142
B	-0.944057	-1.342130	-1.129703
N	-0.604039	-1.955224	0.281485
C	1.050678	-1.442823	1.947691
H	1.887518	-0.788069	2.191541
H	0.515756	-1.673148	2.870492
C	1.504421	-2.745766	1.253514
H	2.508356	-2.636683	0.837697
H	1.520279	-3.578102	1.960261
C	0.488187	-2.989195	0.118681
H	0.958594	-2.852567	-0.856251
H	0.026342	-3.974474	0.148987
B	-1.800614	-2.584743	1.162896
H	-2.195823	-3.574253	0.586544
H	-1.386726	-2.828286	2.273328
H	-2.700617	-1.742411	1.249103
O	-2.204755	-0.428607	-0.816670
C	-3.372098	-0.798627	-0.551319
C	-3.980996	-2.042303	-1.120517
H	-3.242584	-2.823239	-1.273166
H	-4.781691	-2.408114	-0.477506
H	-4.416014	-1.763448	-2.090464
H	-1.198104	-2.129087	-1.996308
C	-4.269818	0.196425	0.127728
H	-4.850685	-0.349620	0.878623
H	-5.001449	0.510234	-0.633710
C	-3.547391	1.397213	0.729711
H	-2.856341	1.074853	1.511492
H	-2.965535	1.935212	-0.020456
H	-4.270731	2.085910	1.173544
H	4.656554	-1.302755	-2.175645
H	4.972959	1.341394	1.196587
H	-0.894606	4.151845	-1.978744
H	-0.502145	3.945167	2.296200

h-TS7Rpri

C	0.470359	0.171123	-0.047572
C	-0.001623	-0.716189	1.153760
H	-0.788767	-0.171433	1.675012
C	0.297088	1.661822	0.237858
C	0.117486	2.530184	-0.845943
C	0.342309	2.195896	1.529119
C	-0.033724	3.898777	-0.641021
H	0.088573	2.113225	-1.845746
C	0.196699	3.569983	1.735961
H	0.489649	1.545834	2.385099
C	0.003696	4.424778	0.652985
H	-0.113623	5.492130	0.813137
C	1.919750	-0.098861	-0.477123
C	2.997274	0.457808	0.223890
C	2.187579	-0.926859	-1.571329
C	4.310497	0.154473	-0.129619
C	3.502410	-1.227126	-1.929552
H	1.361219	-1.329632	-2.142512
C	4.568987	-0.696846	-1.205032
H	5.132173	0.591834	0.429632
H	3.690816	-1.875161	-2.780419
O	-0.418626	-0.182336	-1.103231
B	-0.984055	-1.489226	-0.948495
N	-0.647556	-1.930506	0.541094
C	1.030286	-1.258210	2.140303
H	1.841473	-0.554205	2.332642
H	0.522796	-1.463524	3.085290

C	1.529190	-2.567418	1.506082
H	2.431487	-2.386193	0.918726
H	1.770841	-3.313128	2.267205
C	0.384934	-3.042623	0.590388
H	0.740094	-3.249117	-0.418894
H	-0.113837	-3.933915	0.968848
B	-1.920726	-2.378797	1.452428
H	-2.429955	-3.348252	0.938944
H	-1.514300	-2.577794	2.576365
H	-2.713245	-1.431268	1.467562
O	-2.548356	-1.459775	-1.079315
C	-3.383012	-0.754207	-0.461290
C	-4.783287	-1.275601	-0.396803
H	-5.444679	-0.612082	-0.966846
H	-4.830545	-2.288947	-0.792176
H	-5.121158	-1.262903	0.642294
H	-0.684486	-2.310234	-1.773343
C	-3.133487	0.668815	-0.052214
H	-3.878612	0.957118	0.693371
H	-2.144118	0.781911	0.379194
C	-3.228588	1.574349	-1.299011
H	-4.210258	1.511623	-1.778770
H	-3.054474	2.610163	-1.000266
H	-2.457766	1.292071	-2.017328
H	5.591816	-0.932287	-1.482783
H	2.809748	1.145211	1.041758
H	0.230803	3.967060	2.745961
H	-0.177949	4.558041	-1.491938

h-TS7S

C	0.696932	0.102488	-0.127432
C	0.099274	-0.698922	1.109274
H	-0.501346	-0.015125	1.706688
C	0.492464	1.610414	0.053937
C	0.240580	2.398706	-1.073618
C	0.585838	2.234562	1.302517
C	0.075999	3.776557	-0.954534
H	0.158797	1.910477	-2.037239
C	0.424749	3.616379	1.423777
H	0.782896	1.647875	2.194725
C	0.166721	4.392582	0.295449
H	0.037695	5.466684	0.388102
C	2.180500	-0.181164	-0.401165
C	3.189617	0.386599	0.386898
C	2.550162	-1.027452	-1.450693
C	4.530661	0.087046	0.154837
H	2.929839	1.079907	1.179222
C	3.892895	-1.323243	-1.688899
H	1.774338	-1.435295	-2.085737
C	4.888986	-0.774326	-0.882819
H	5.933788	-1.005318	-1.066777
O	-0.049576	-0.344354	-1.258847
B	-1.230759	-1.010204	-0.850546
N	-0.847386	-1.704018	0.512194
C	1.051968	-1.522966	1.973789
H	2.017595	-1.036649	2.112437
H	0.594399	-1.660991	2.954753
C	1.174212	-2.880070	1.245467
H	2.130638	-2.959176	0.724145
H	1.106038	-3.708551	1.953630
C	0.015645	-2.912856	0.227341
H	0.396430	-2.839075	-0.792318
H	-0.609848	-3.800402	0.304540
B	-2.025089	-2.124905	1.529285
H	-2.650443	-3.025305	1.012009
H	-1.539050	-2.443987	2.589854
H	-2.754424	-1.142337	1.703502
O	-2.223469	0.134574	-0.378646
C	-3.401324	-0.009735	0.028579
C	-3.981963	1.121933	0.817656
H	-4.753938	1.616970	0.215585
H	-4.466779	0.734216	1.716450
H	-3.203920	1.838171	1.080957
H	-1.747409	-1.713708	-1.671512
C	-4.311827	-1.082914	-0.494898
H	-3.779919	-2.031408	-0.529275
H	-5.159247	-1.194099	0.185693
C	-4.792607	-0.684314	-1.906639
H	-3.946245	-0.611160	-2.593526
H	-5.478030	-1.446826	-2.284236
H	-5.322176	0.273458	-1.905866
H	0.498442	4.081805	2.402231
H	-0.124815	4.372411	-1.840224
H	5.296433	0.535228	0.780904
H	4.158682	-1.981697	-2.510774

h-TS7Spri

C	0.637030	0.160904	-0.107220
C	-0.111697	-0.399505	1.149158
H	-0.664978	0.427156	1.595017

C	0.999950	1.635748	0.055721
C	1.078596	2.435168	-1.091193
C	1.288455	2.207847	1.298762
C	1.431361	3.778505	-0.995051
H	0.845794	1.988922	-2.050914
C	1.647363	3.554261	1.395446
H	1.233297	1.609811	2.202413
C	1.719019	4.343771	0.249682
H	1.995505	5.391014	0.324096
C	1.897847	-0.635862	-0.474760
C	3.104113	-0.443313	0.211236
C	1.854457	-1.586039	-1.499294
C	4.223385	-1.219218	-0.083315
H	3.175559	0.328863	0.969472
C	2.975953	-2.360480	-1.798634
H	0.939009	-1.709251	-2.063329
C	4.161526	-2.188845	-1.085356
H	5.033421	-2.793020	-1.316725
O	-0.324325	0.052319	-1.151189
B	-1.313759	-0.954476	-0.910762
N	-1.129260	-1.372923	0.617875
C	0.672274	-1.170587	2.209425
H	1.675806	-0.772165	2.365103
H	0.127782	-1.104516	3.153426
C	0.695406	-2.624632	1.704495
H	1.621481	-2.825204	1.162191
H	0.632210	-3.333697	2.533152
C	-0.511358	-2.753166	0.754854
H	-0.204655	-3.106582	-0.228964
H	-1.282089	-3.422203	1.135335
B	-2.461535	-1.317185	1.531966
H	-3.238010	-2.149411	1.127511
H	-2.142916	-1.469318	2.689755
H	-2.946702	-0.180803	1.403817
O	-2.766899	-0.393857	-1.065266
C	-3.318555	0.549799	-0.439901
C	-2.623694	1.834919	-0.101770
H	-1.554937	1.718511	0.034103
H	-2.767892	2.498227	-0.965909
H	-3.084442	2.306845	0.766775
H	-1.327290	-1.892909	-1.661621
C	-4.821269	0.536326	-0.363250
H	-5.097968	0.863847	0.644116
H	-5.162114	1.339817	-1.035280
C	-5.469941	-0.795626	-0.721912
H	-5.212575	-1.095927	-1.739983
H	-5.124604	-1.577258	-0.042997
H	-6.557485	-0.714254	-0.647092
H	1.866080	3.982990	2.368791
H	1.483697	4.386882	-1.893164
H	5.147455	-1.058586	0.463960
H	2.921324	-3.096550	-2.595290

14. References

[1] E. J. Corey, C. J. Helal, *Angew. Chem. Int. Ed.* **1998**, *37*, 1986-2012.
[2] R. Pollice, P. Chen, *Angew. Chem. Int. Ed.* **2019**, *58*, 9758-9769.
[3] G. B. Stone, *Tetrahedron: Asymmetry* **1994**, *5*, 465-472.
[4] a) E. J. Corey, R. K. Bakshi, S. Shibata, C. P. Chen, V. K. Singh, *J. Am. Chem. Soc.* **1987**, *109*, 7925-7926; b) E. J. Corey, R. K. Bakshi, S. Shibata, *J. Am. Chem. Soc.* **1987**, *109*, 5551-5553; c) E. J. Corey, J. O. Link, *Tetrahedron Lett.* **1989**, *30*, 6275-6278.
[5] a) M. C. Sherman, M. R. Ams, K. D. Jordan, *J. Phys. Chem. A* **2016**, *120*, 9292-9298; b) J. W. G. Bloom, R. K. Raju, S. E. Wheeler, *J. Chem. Theory. Comput.* **2012**, *8*, 3167-3174; c) S. Tsuzuki, K. Honda, T. Uchimaru, M. Mikami, K. Tanabe, *J. Phys. Chem. A* **2002**, *106*, 4423-4428.
[6] E. J. Corey, J. O. Link, R. K. Bakshi, *Tetrahedron Lett.* **1992**, *33*, 7107-7110.
[7] M. P. Gamble, A. R. C. Smith, M. Wills, *J. Org. Chem.* **1998**, *63*, 6068-6071.
[8] T. Touge, H. Nara, M. Fujiwhara, Y. Kayaki, T. Ikariya, *J. Am. Chem. Soc.* **2016**, *138*, 10084-10087.
[9] A. C. Albéniz, P. Espinet, R. Manrique, A. Pérez-Mateo, *Angew. Chem. Int. Ed.* **2002**, *41*, 2363-2366.
[10] G. A. Hiegel, K. B. Peyton, *Synth. Commun.* **1985**, *15*, 385-392.
[11] A. L.-F. Chow, M.-H. So, W. Lu, N. Zhu, C.-M. Che, *Chem. Asian J.* **2011**, *6*, 544-553.
[12] V. Diemer, H. Chaumeil, A. Defoin, A. Fort, A. Boeglin, C. Carré, *Eur. J. Org. Chem.* **2006**, *2006*, 2727-2738.
[13] S. Poplata, T. Bach, *J. Am. Chem. Soc.* **2018**, *140*, 3228-3231.
[14] D. J. Mathre, T. K. Jones, L. C. Xavier, T. J. Blacklock, R. A. Reamer, J. J. Mohan, E. T. T. Jones, K. Hoogsteen, M. W. Baum, E. J. J. Grabowski, *J. Org. Chem.* **1991**, *56*, 751-762.
[15] A. Lattanzi, A. Russo, *Tetrahedron* **2006**, *62*, 12264-12269.
[16] Y.-k. Liu, C. Ma, K. Jiang, T.-Y. Liu, Y.-C. Chen, *Org. Lett.* **2009**, *11*, 2848-2851.
[17] C. Ó Dálaigh, S. J. Connon, *J. Org. Chem.* **2007**, *72*, 7066-7069.
[18] D. Enders, P. Fey, H. Kipphardt, *Org. Prep. Proced. Int.* **1985**, *17*, 1-9.
